# Synthesis of branched and linear galactooligosaccharides related to glucuronoxylomannogalactan of *Cryptococcus neoformans*

**DOI:** 10.3389/fchem.2024.1501766

**Published:** 2024-11-14

**Authors:** Vera S. Dorokhova, Bozhena S. Komarova, José O. Previato, Lúcia Mendonça Previato, Vadim B. Krylov, Nikolay E. Nifantiev

**Affiliations:** ^1^ Laboratory of Glycoconjugate Chemistry, N.D. Zelinsky Institute of Organic Chemistry, Russian Academy of Sciences, Moscow, Russia; ^2^ Laboratório de Glicobiologia, Instituto de Biofísica Carlos Chagas Filho, Universidade Federal do Rio de Janeiro, Rio de Janeiro, Brazil; ^3^ Laboratory of Synthetic Glycovaccines, N.D. Zelinsky Institute of Organic Chemistry, Russian Academy of Sciences, Moscow, Russia

**Keywords:** *Cryptococcus neoformans*, oligosaccharides, glucuronoxylomannogalactan, stereoselective glycosylation, orthogonal protecting groups

## Abstract

This study focuses on the synthesis of a series of oligo-*α*-(1→6)-D-galactopyranosides bearing *β*-D-galactofuranosyl residues at O-2 and/or O-3, which relate structurally to fragments of glucuronoxylomannogalactan (GXMGal) from the fungal pathogen *Cryptococcus neoformans* that causes severe diseases in immunocompromised patients. The preparation of target compounds is based on the use of a selectively O-protected N-phenyltrifluoroacetimidoyl galactopyranoside donor with an allyl group at O-2, levulinoyl group (Lev) at O-3, pentafluorobenzoyl (PFB) group at O-4, and fluorenylmethoxycarbonyl (Fmoc) group at O-6. The choice of protecting groups for this donor ensures the stereospecific formation of *α-*(1→6)-glycosidic bonds due to the stereodirecting effect of acyls at O-3, O-4, and O-6. At the same time, this combination of O-substituents permits the selective recovery of free OH groups at O-2, O-3, and O-6 for chain elongation *via* the introduction of *β-*D-galactofuranosyl and *α-*D-galactopyranosyl residues. The reported compounds are obtained as aminopropyl glycosides, which are transformed into biotinylated conjugates for further use as coating antigens in immunological studies. The obtained oligosaccharides were subjected to detailed ^13^C NMR analysis to show the spatial similarity of the obtained hexasaccharide with the corresponding fragment in the GXMGal chain, making this compound suitable for further immunological studies of *C. neoformans*.

## Introduction

*Cryptococcus neoformans* is a human fungal pathogen capable of causing severe diseases in patients with a weakened immune system (especially in patients with HIV/AIDS) (Bermas and Geddes-McAlister, 2020; Zhao et al., 2023). This fungus can attack the central nervous system, thus causing cryptococcal meningitis, a fatal disease if untreated (Chen et al., 2022). In recent years, serious concerns have arisen about the increasing cases of cryptococcal meningitis in HIV-seronegative individuals (Paccoud et al., 2023). This fungus can also attack the lungs, skin, and other organs, which also leads to serious complications (Rivera et al., 1998). This pathogen spreads through bird droppings and enters the human body through inhaled dust (Maziarz and Perfect, 2016). *C. neoformans* is most commonly found in territories of Africa and Southern and Southeastern Asia, but the affected area is expanding every year (Rajasingham et al., 2022).

One of the main factors contributing to the virulence of *C. neoformans* is its bulk polysaccharide capsule (Vecchiarelli, 2000; Doering, 2009). It is composed mainly of glucuronoxylomannan (GXM), with minor components—glucuronoxylomannogalactan (GXMGal) and mannoprotein. The structure and immunological properties of GXM were studied in detail (Cherniak et al., 1998; McFadden and Casadevall, 2004; Oscarson et al., 2005; Nakouzi et al., 2009; Hargett et al., 2024), and their heterogeneity was shown for different serotypes. In contrast, the minor polysaccharide GXMGal of the *C. neoformans* capsule, which has not attracted significant attention until recently, is of great interest from an immunological point of view due to its immunomodulatory effect (Villena et al., 2008; Vecchiarelli et al., 2011; Decote-Ricardo et al., 2019). Unlike GXM (Cherniak et al., 1980; Skelton et al., 1991a; 1991b; James and Cherniak, 1992), GXMGal is a conserved polysaccharide that seems to be structurally similar in all *C. neoformans* serotypes studied to date (Cherniak et al., 1982; James and Cherniak, 1992; Vaishnav et al., 1998; Heiss et al., 2009).

**FIGURE 1:**
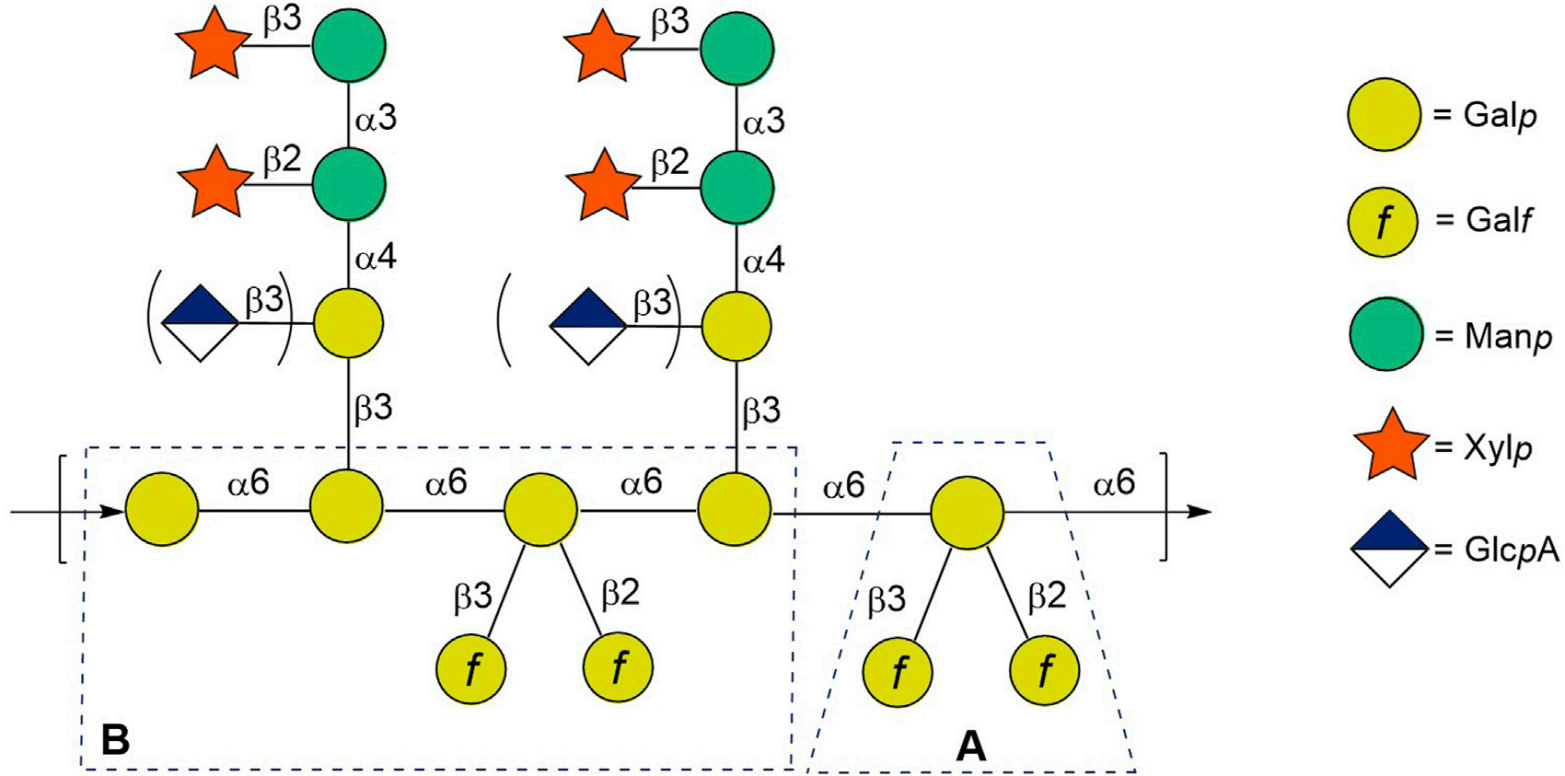
Structure of glucuronoxylomannogalactan of *Cryptococcus neoformans*. Presumed immunodominant vicinal branched fragment is framed.

Generally, GXMGal consists of a poly-*α-*(1→6)-D-galactopyranan backbone bearing *β-*Xyl*p*-(1→3)-*α-*Man*p*-(1→3)[*β-*Xyl*p*-(1→2)-]-*α-*Man*p*-1→4)[*β-*Glc*p*A-1→3)]-*β-*Gal*p* and β-D-galactofuranosyl residues (Figure 1) (Heiss et al., 2013; Previato et al., 2017). However, *C. neoformans* GXMGal does not contain a regular and defined repeating unit due to the variable addition of *β-*Glc*p*A, *β-*Xyl*p*, and O-acetyl groups on the *β-*Gal*p* side chains and a variable number of *β-*Gal*f* branches on the polysaccharide backbone. Given the high immunological activity of galactofuranosyl-bearing epitopes demonstrated on a number of other polysaccharide antigens (Turco and Pedersen, 2003; Peltier et al., 2008; Richards and Lowary, 2009; Tefsen et al., 2012; Krylov et al., 2021; Argunov et al., 2024), we started the systematic synthesis of spacer-armed oligosaccharides related to GXMGal fragments bearing galactofuranosyl residues for their immunological studies toward the development of potential immunomodulators, diagnostic kits, and vaccines.

**FIGURE 2:**
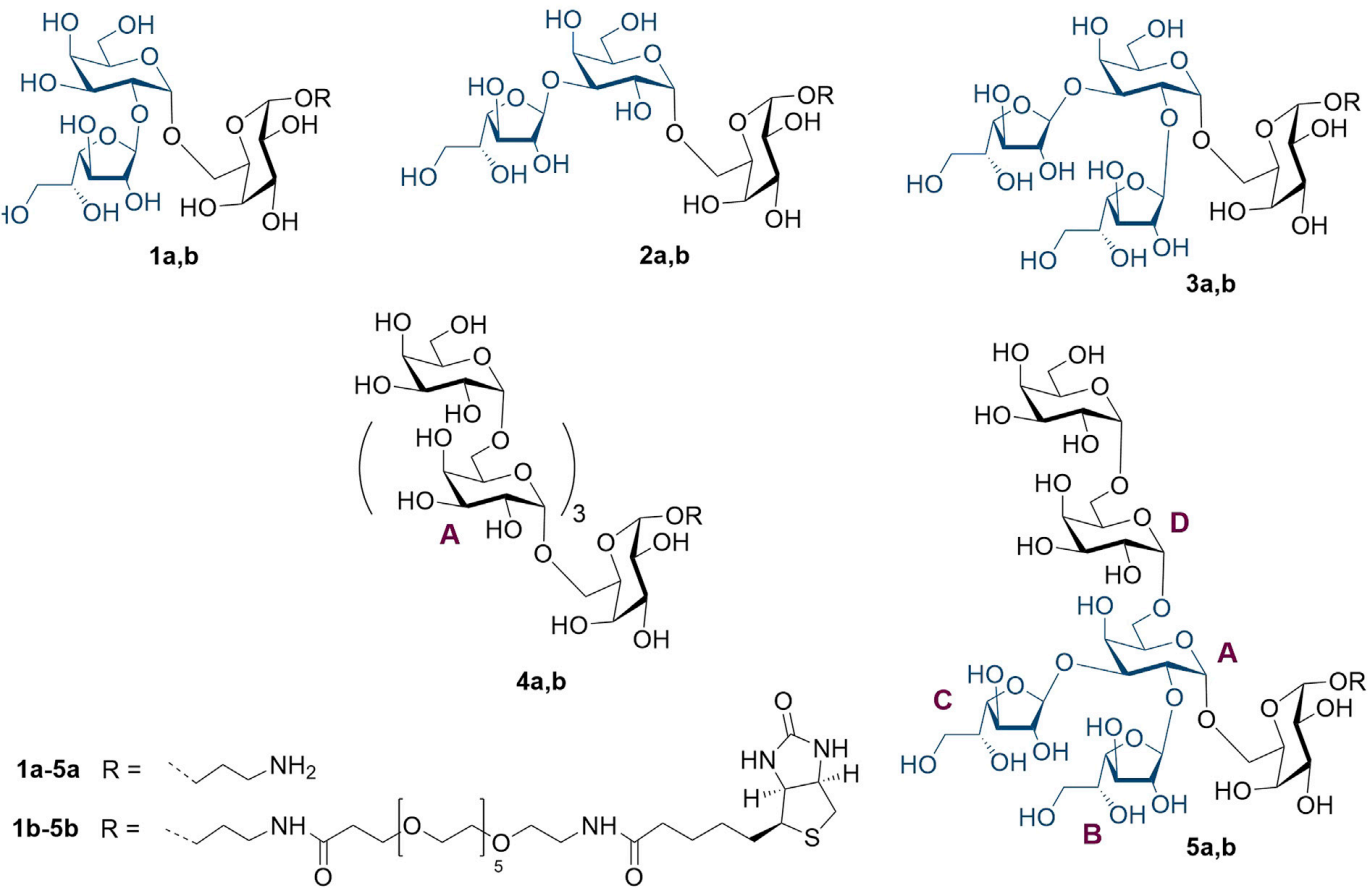
Studied set of oligosaccharides related to the vicinally branched galactoside fragment of GXMGal.

Previously (Dorokhova et al., 2021), we described the preparation, nuclear magnetic resonance (NMR), and conformational studies of the model trisaccharide with two *β-*D-galactofuranosyl residues at O-2 and **O**-3, which relates to branch point **A** (Figure 1), as well as of its constituent monofuranosylated disaccharides. In this study, we report on the synthesis and NMR studies of spacered hexasaccharide **5a** related to fragment **B** (Figure 1) of the GXMGal chain (Figure 1), which includes not only 2,3-vicinal branching but also 1,2-*cis*-pseudo-branching. These elements may influence the 3D structure of oligo and polysaccharides and, therefore, should be taken into account during the selection of the oligosaccharide, which is spatially equivalent to the target antigenic polysaccharide GXMGal. In addition to **5a**, the synthesis of a series of its constituting oligosaccharide derivatives **1a–4a** is also described, along with the preparation of corresponding biotinylated glycoconjugates **1b**–**5b** required for use as molecular probes and coating antigens in a variety of immunological investigations (Figure 2).

## Results and discussion

The galactopyranosyl units in target compounds have an *α-*anomeric configuration and, thus, are connected to other parts of the molecules through 1,2-*cis*-glycosidic bonds. Their stereoselective construction can be accomplished by the remote anchimeric assistance of remote acyl groups at O-3, O-4, and O-6 of the glycosyl donor. The stereocontrolling participation of a single acyl group, as well as the combined effect of two or three acyls, was previously applied for stereoselective 1,2-*cis*-glycosylation by gluco- and galacto-glycosyl donors [for reviews, see Nigudkar and Demchenko (2015); Komarova et al. (2016); Hettikankanamalage et al. (2020); Tokatly et al. (2021)] toward the synthesis of biologically relevant oligosaccharides (Gerbst et al., 2001; Calin et al., 2013; Komarova et al., 2014; 2015; 2018a; 2018b; Vinnitskiy et al., 2015; Zou et al., 2018; Zhang et al., 2022). In the present work, we explored this approach in the case of galactosylation and used a stereodirecting acyl group at O-6 as the temporary substituent needed for the selective liberation of OH to be glycosylated.

It was also shown that the introduction of fluoro-substituted benzoates at O-6 in glucosyl donors also favors the selectivity of 1,2-*cis*-glycosylation (Cato et al., 2005; Vohra et al., 2009; Komarova et al., 2018a; 2023). In this work, we report an integrated approach of both participating and withdrawing acceptor groups to achieve high *α-*selectivity in the synthesis of target structures.

### Synthesis of compounds 1–5

In order to obtain target compounds **1–5**, universal synthetic blocks **13**, **21**, and **33** were designed. They, on one hand, would ensure the stereoselective building of 1,2-*cis*-glycosidic bonds between galactopyranose residues and, on the other hand, would allow the regioselective deprotection of hydroxyl groups at C-2, C-3, and C-6 for the efficient synthesis of branched fragments and chain extension. Both goals were achieved by the rational selection of protecting groups in the galactopyranosyl donors. Thus, donor **13**, which is the precursor of the branched unit, carries a non-participating allyl (All)-protecting group at O-2 and participating acyl groups at O-3 (levulinoyl, Lev) (Komarova et al., 2014; 2015; 2023), O-4 (pentafluorobenzoyl, PFB) (Komarova et al., 2018a), and O-6 atoms (fluorenylmethyloxycarbonyl, Fmoc), which favored the *α-*stereocontrol of the glycosylation reaction. According to the literature data, each of the above groups can be selectively removed under orthogonal conditions without affecting the other protecting groups (Prabhu et al., 2003; Ágoston et al., 2016).

**SCHEME 1:**
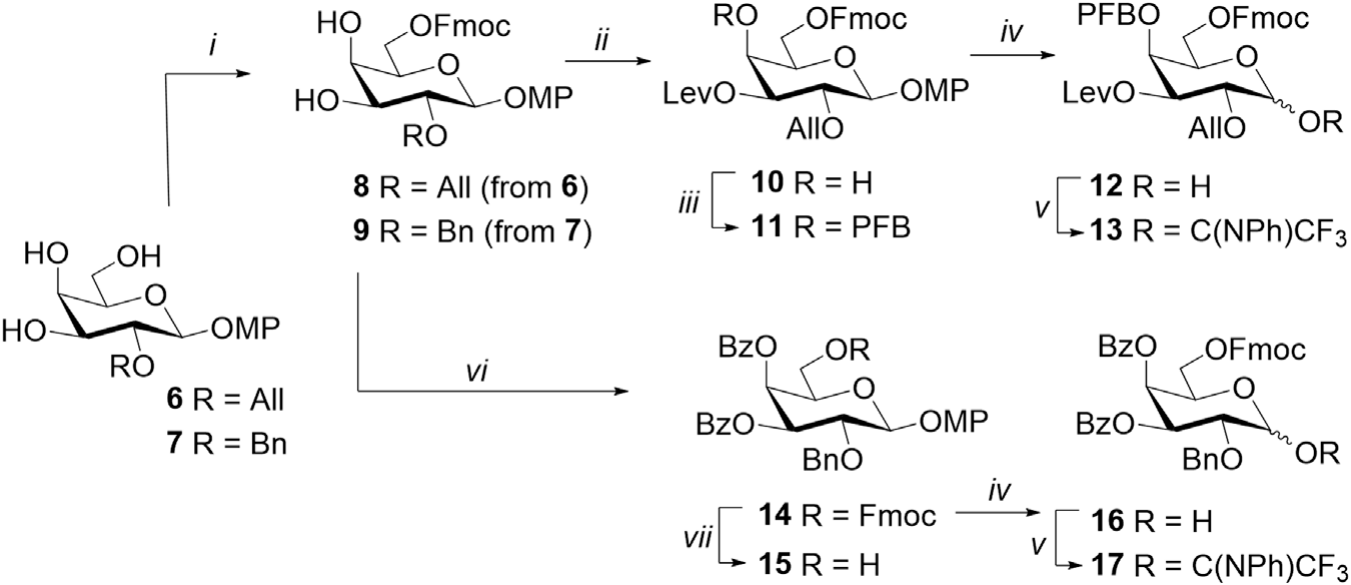
Synthesis of the monosaccharide donors **13** and **17** and acceptor **15**. Reagents and conditions: (i) FmocCl, 2,6-lutidine, MeCN, 2–3 days, 65% for **8** and 64% for **9**; (ii) LevOH, CMPI, DMAP, CH_2_Cl_2_, -18°C, 20 h, 87%; (iii) pentafluorobenzoyl chloride, Py, DMAP, 12 h, 82%; (iv) CAN, MeCN, benzene, H_2_O, 0 °C, 10–12 min, 88% for **12** and 74% for **16**; (v) ClC(NPh)CF_3_, K_2_CO_3_, acetone, 12 h, 82% for **13** and 84% for **17**; (vi) BzCl, Py, DMAP, CH_2_Cl_2_, 12 h, 99%; and (vii) piperidine, CH_2_Cl_2_, 0 °C, 15 min, 69%.

Galactosyl donor **13** was synthesized from the well-known monosaccharide precursor **6** (Thijssen et al., 1998) (Scheme 1). Its primary hydroxyl group at C-6 was regioselectively protected by Fmoc to form 3,4-diol **8** (Argunov et al., 2016). The introduction of the levulinoyl group using 1-ethyl-3-(3-dimethylaminopropyl) carbodiimide (EDC) hydrochloride and dimethylaminopyridine (DMAP) at −18^о^С proceeded exclusively at O-3 (Hirose et al., 2015), and the subsequent treatment of product **10** with pentafluorobenzoyl chloride in the presence of pyridine (Py) allowed obtaining a fully protected monosaccharide **11**. The *p*-methoxyphenyl-protecting group was removed from the anomeric center by ceric ammonium nitrate (CAN) in a mixture of acetonitrile, benzene, and water to form the corresponding hemiacetal **12**, which was then converted to *N*-phenyltrifluoroacetimidate **13**. It is important to note that glycosyl donors of this type are usually purified on silica gel with the addition of triethylamine to the eluent in order to neutralize the silica gel and reduce the cleavage of the acid-labile leaving group. In the case of Fmoc-containing donors, the presence of triethylamine led to a loss of yield as a result of partial Fmoc removal (Oberli et al., 2008). Chromatography of donor **13** on neutral aluminum oxide without triethylamine has made it possible to mitigate side reactions and isolate the product with a sufficiently high yield of 82%.

Monosaccharide blocks **15** and **17** were obtained based on 2-O-benzylated triol **7** (Zhu and Yang, 2012) (Scheme 1). Protecting groups (6-O-Fmoc and two benzoate groups at C-3 and C-4) were introduced sequentially with considerably high yields at each step. The resulting monosaccharide **14** was partially converted to acceptor **15** after Fmoc removal under the action of piperidine in tetrahydrofuran (THF). Hemiacetal **16** was also obtained from monosaccharide **14** and then treated with N-phenyltrifluoroacetimidoyl chloride in acetone. As in the case of donor **13**, chromatographic purification of the resulting 6-O-Fmoc-bearing donor was performed on neutral Al_2_O_3_, yielding compound **17** with a considerably high yield of 84%.

**SCHEME 2:**
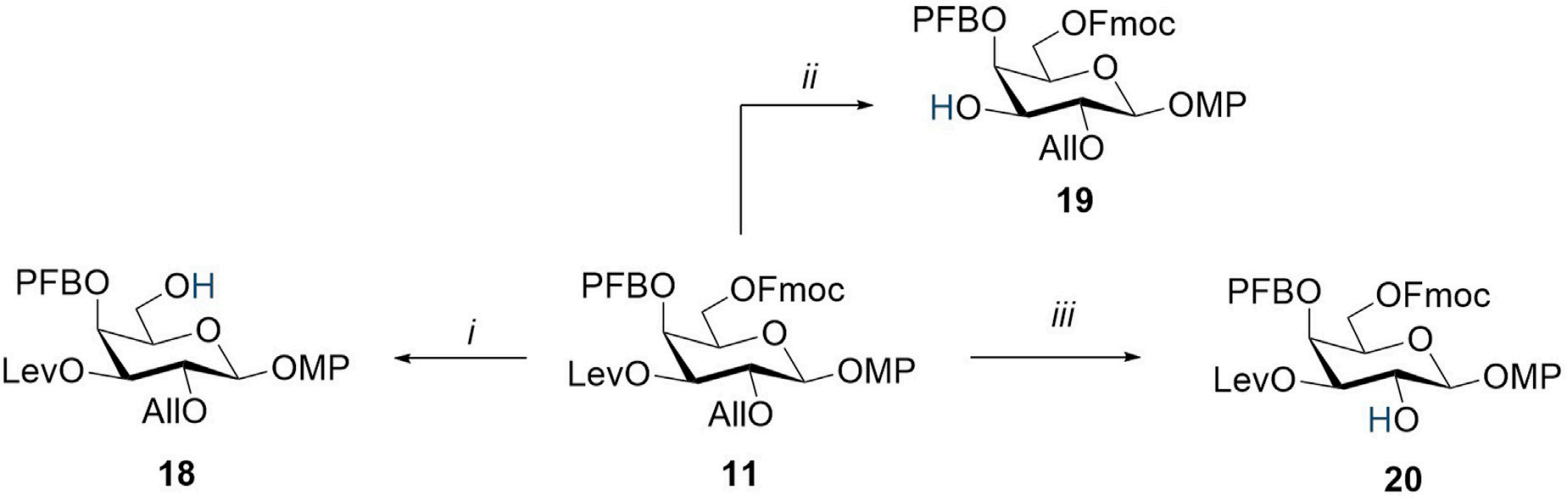
Regioselective removal of orthogonal-protecting groups in monosaccharide **11**. Reagents and conditions: (i) *N*-methylmorpholine, CH_2_Cl_2_, 2 days, 65%; (ii) NH_2_NH_2_∙H_2_O, AcOH, Py, 20 min, 96%; and (iii) [Ir(COD) (PMePh_2_)_2_]PF_6_, H_2_, I_2_, THF, 2 h, 99%.

The conditions for the selective removal of the chosen protecting groups (Fmoc, Lev, and All) were optimized using the model monosaccharide **11** (Scheme 2). The use of the standard Fmoc-removal procedure in the piperidine/THF system led to a rapid cleavage of the Fmoc group (Pennington and Dunn, 1994; Werz, 2012). However, under these conditions, a side reaction was observed, which consisted of the substitution of a fluorine atom in 4-O-pentafluorobenzoate by piperidine. This process was confirmed by HRMS data and the emergence of piperidine ring signals in ^1^H NMR spectra at 3.31 ppm 1.65 ppm and ^13^C NMR at 52.1 ppm and 23.9 ppm, respectively. Thus, these conditions can be used only for compounds without a pentafluorobenzoyl group or only at the last synthetic steps of the complete deprotection. Nevertheless, the Fmoc group in the presence of pentafluorobenzoate was successfully removed under milder conditions under the action of N-methylmorpholine in methylene chloride for 2 days. The levulinoyl group was efficiently and selectively removed by hydrazine acetate in pyridine, resulting in compound **19** with a 96% yield. The allyl substituent at O-2 was selectively removed using (1,5-cyclooctadiene)bis (methyldiphenylphosphine)iridium(I) hexafluorophosphate ([Ir(COD) (PMePh_2_)_2_]PF_6_), which was pre-reduced with hydrogen (Laroussarie et al., 2015). This resulted in compound **20** with an almost quantitative yield.

In order to increase the efficiency of the *α-*(1→6)-glycoside bond formation, the conditions for the glycosylation of the spacer-containing acceptor **21** by donor **13** were optimized. Originally, the coupling was carried out in the presence of trimethylsilyl trifluoromethane sulfonate (TMSOTf) at −35°C (Table 1, entry 1). The desired disaccharide **23** was obtained with an insufficient yield of 28%; however, full *α-*stereospecificity was achieved that can be explained by the presence of three *α-*directing protecting groups in donor **13**. The low yield may be attributed to the presence of three electron-withdrawing groups in the donor, which lower its activity; hence, the side processes of its destruction occur before the glycosylation reaction is completed. An increase in the reaction yield to 47% was achieved by replacing the promoter with triflic acid, along with a gradual increase in temperature to −15°C (Table 1, entry 2). One of the reasons for the low yield of disaccharide **23**, in this case, is the removal of Fmoc from the O-6 product by triethylamine, which is used to neutralize unreacted acid after the reaction is completed (Oberli et al., 2008). This is confirmed by an increase in the yield of disaccharide **23** up to 64% when the addition of triethylamine was omitted, and the reaction mixture was immediately filtered and washed with a saturated NaHCO_3_ solution (Table 1, entry 3).

**TABLE 1:** Optimization of acceptor **21** glycosylation by donor **13**.

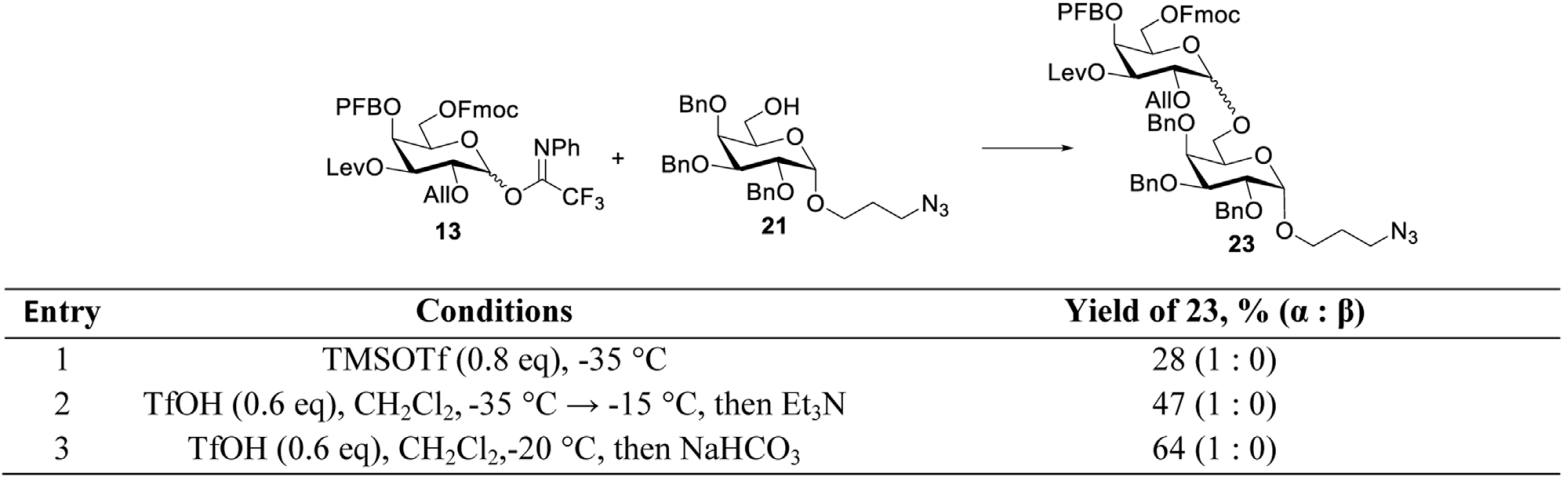

**SCHEME 3:**
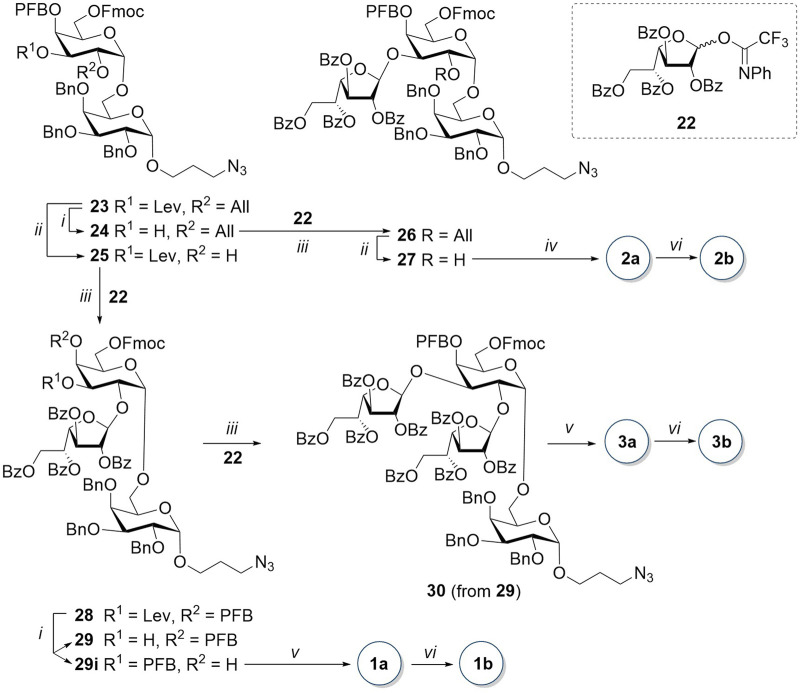
Synthesis of tri- and tetrasaccharides **1**, **2**, and **3**. Reagents and conditions: (i) NH_2_NH_2_∙H_2_O, AcOH, Py, 10–11 min, 92% for **24**, 27% for **29**, and 29% for **29i**; (ii) [Ir(COD) (PMePh_2_)_2_]PF_6_, H_2_, I_2_, THF, 2 h, 83% for **25** and 78% for **27**; (iii) TMSOTf, AW300, CH_2_Cl_2_, -20°C, only β, 1.5 h, 86% for **26**, 3 h, 87% for **28**, and 17 min, 70% for **30**; (iv) 1) piperidine, THF, 0 C, 40 min; 2) NaOCH_3_, MeOH, 12 h; 3) H_2_, Pd(OH)_2_/C, HCl, MeOH, EtOAc, 25 min, 79% in three steps; (v) 1) piperidine, THF, 0 C, 25–60 min; 2) NaOCH_3_, MeOH, 3.5–12 h; and 3) Na, NH_3_, THF, 50–60 min, 56% for **1a**, 35% for **3a** in three steps; (vi) AEB, Et_3_N, DMF, 30 min, 50% for **1b**, 85% for **2b**, and 2.5 h, 44% for **3b**.

The disaccharide acceptor **25** was obtained from **23** by the removal of the 2-O-allyl group with the iridium complex [Ir(COD) (PMePh_2_)_2_]PF_6_ pre-reduced with hydrogen (Scheme 3). Glycosylation of acceptor **25** by galactofuranosyl donor **22**, previously obtained by us (B. Krylov et al., 2018), in the presence of TMSOTf, resulted in trisaccharide **28** with a high yield as a pure *β-*isomer. In the next step, the hydroxyl group at the C-3 atom of the non-reducing residue was recovered by hydrazine acetate in pyridine. However, in addition to the expected disaccharide **29**, we observed the formation of a migration product of pentafluorobenzoate from O-4 to O-3 (compound **29i**). Both regioisomers **29** and **29i** were successfully separated by column chromatography and found applications in the synthetic scheme.

The target trisaccharide **1a** was synthesized from the pentafluorobenzoyl migration product **29i** in three steps. First, the Fmoc-protecting group was removed with piperidine in THF. Then, without intermediate purification, benzoyl substituents were removed in the presence of sodium methylate in methanol. The following reduction of the azide group in the spacer to the amino group and the simultaneous removal of benzyl groups by treatment with sodium in liquid ammonia yielded the unprotected (1→2)-trisaccharide **1a** with a high yield of 98%. Tetrasaccharide **30** was obtained by coupling galactofuranosyl donor **22** and trisaccharide acceptor **29** with a fairly high yield of 87% and exclusively as a pure *β-*isomer. The sequential removal of protecting groups using a scheme similar to that described above for trisaccharide **29i** resulted in unprotected tetrasaccharide **3a**.

Unlike in trisaccharide **28**, the removal of the 3-O-levulinoyl group in disaccharide **23** did not result in the migration of the pentafluorobenzoyl group from O-4 to O-3. The furanosyl residue was introduced by glycosylation with donor **22**, resulting in the formation of *β-*(1→3)-trisaccharide **26** with an 86% yield. The removal of all protecting groups in compound **26** included (1) 2-O-deallylation (→**27**); (2) removal of 6-O-Fmoc with piperidine in THF; (3) removal of 4-O-pentafluorobenzoyl and benzoyl groups with sodium methylate in methanol; and (4) hydrogenolysis on Pd(OH)_2_/C in the presence of a small amount of hydrochloric acid, which prevents the methylation of the amino group of the target (1→3)-trisaccharide **2a**.

**SCHEME 4:**
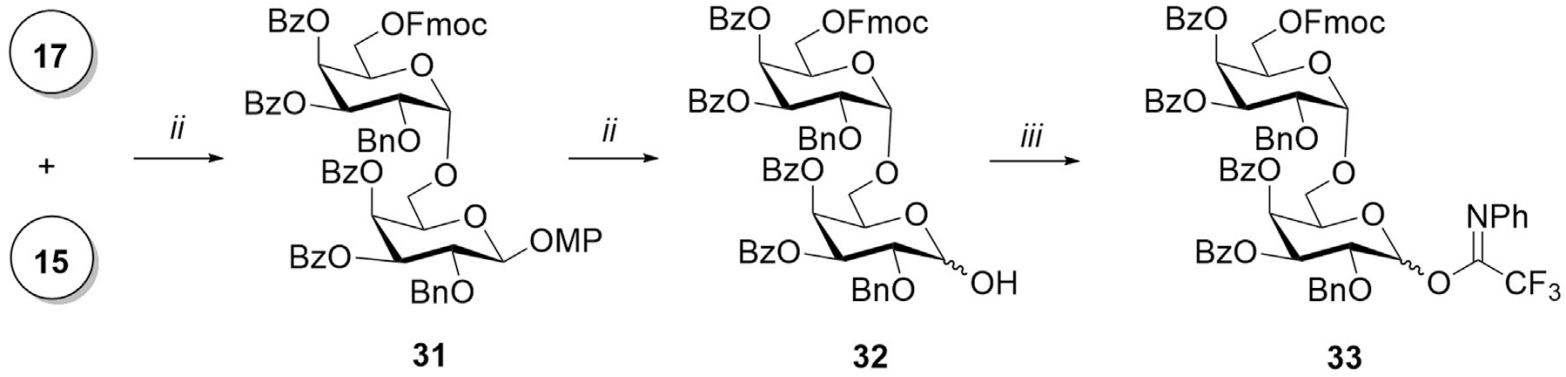
Synthesis of disaccharide donors **42** and **44**. Reagents and conditions: (i) TfOH, MS AW300, CH_2_Cl_2_, -20°C, 7 min, 79%, only α; (ii) CAN, MeCN, benzene, H_2_O, 0 °C, 10 min, 71%; and (iii) ClC(NPh)CF_3_, K_2_CO_3_, acetone, 12 h, 60%.

The *α-*(1→6)-linked galactopyranosyl chain in the synthesis of target compounds **4a** and **5a** was elongated with disaccharide donor **33** (Scheme 4). The glycosylation reaction of *p*-methoxygalactoside **15** by donor **17** in the presence of TfOH proceeded with the exclusive formation of *α-*isomer **33** due to the concerted action of three *α-*stereodirecting acyl groups at O-3, O-4, and O-6 (Baek et al., 2015). The absence of the *β-*isomer among the reaction products was confirmed by the NMR spectra of the untreated reaction mixture. The removal of the *p*-methoxyphenyl-protecting group of the anomeric center, followed by the addition of the N-phenyltrifluoroacetimidoyl-leaving group to the hemiacetal **32**, yielded disaccharide donor **33**.

**SCHEME 5:**
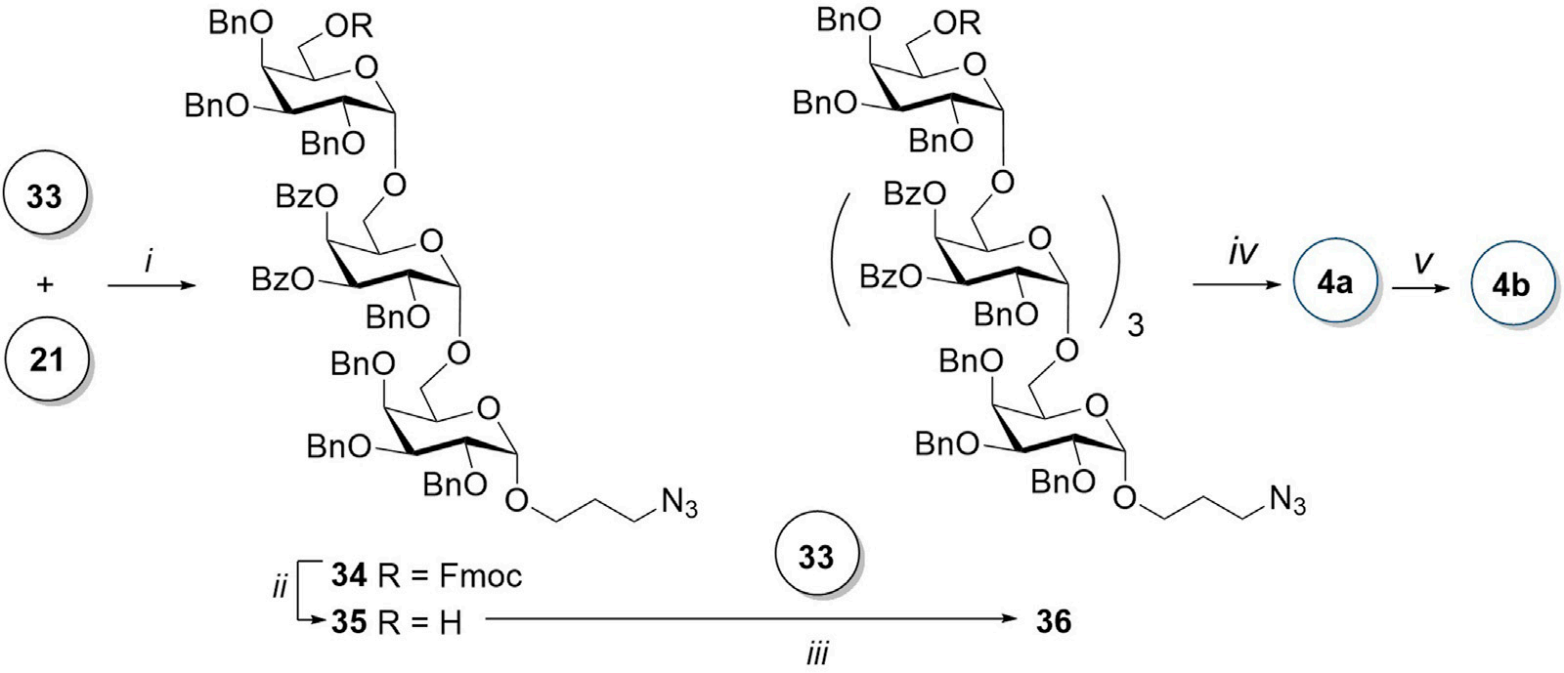
Synthesis of the linear pentasaccharides **4a** and **4b**. Reagents and conditions: (i) TfOH, MS AW300, CH_2_Cl_2_, -20°C, 6 min, α:β = 20:1, 75%; (ii) piperidine, THF, 0 C, 20 min, 95%; (iii) TMSOTf, MS AW300, CH_2_Cl_2_, -5°C, 2.5 h, only α, 55%; (iv) 1) piperidine, THF, 0 C, 35 min; 2) NaOMe, MeOH, 12 h; and 3) H_2_, Pd(OH)_2_/C, HCl, MeOH, EtOAc, 5 h, 59% for the three steps; and (v) AEB, Et_3_N, DMF, 12 h, 92%.

Glycosylation of prespacer-containing monosaccharide **21** by donor **33** in the presence of TfOH (Scheme 5) resulted in a mixture of *α-* and *β-*isomeric trisaccharides in the ratio of 20:1. Their ratio was determined by the integration of the ^1^H NMR spectrum of the reaction mixture. After the successful separation of the two isomers by column chromatography, the desired *α-*product **34** was isolated with a yield of 75%. The trisaccharide acceptor **35** was obtained after the removal of 6-O-Fmoc with piperidine in THF. An attempt of a TfOH-assisted glycosylation of acceptor **35** with a disaccharide donor **33** failed. After an optimization of conditions, it was found that in the presence of TMSOTf and with an increase in temperature from −20°C to −5°C, *α-*pentasaccharide **36** is formed with a sufficient yield of 55% without any *β-*isomer admixture.

Protecting groups in pentasaccharide **36** were removed according to a standardized algorithm: first, Fmoc was removed with piperidine; then, benzoate groups were removed in the presence of sodium methylate in methanol; and, in the last step, the azide group was reduced and benzyl groups removed in the course of catalytic hydrogenolysis. Unprotected pentasaccharide **4a** was isolated by gel permeation chromatography with a 70% yield after all stages of deprotection. The ^1^H NMR spectrum of the product contains five anomeric proton signals. For each of the monosaccharide residues, *α-*configuration of the C-1 atom is confirmed both by spin–spin coupling constants (less than 4 Hz) and chemical shifts of the related carbon atoms (signals at 99.4, 98.9, and 98.8 ppm and two more signals at 98.7 ppm in ^13^C NMR).

**SCHEME 6:**
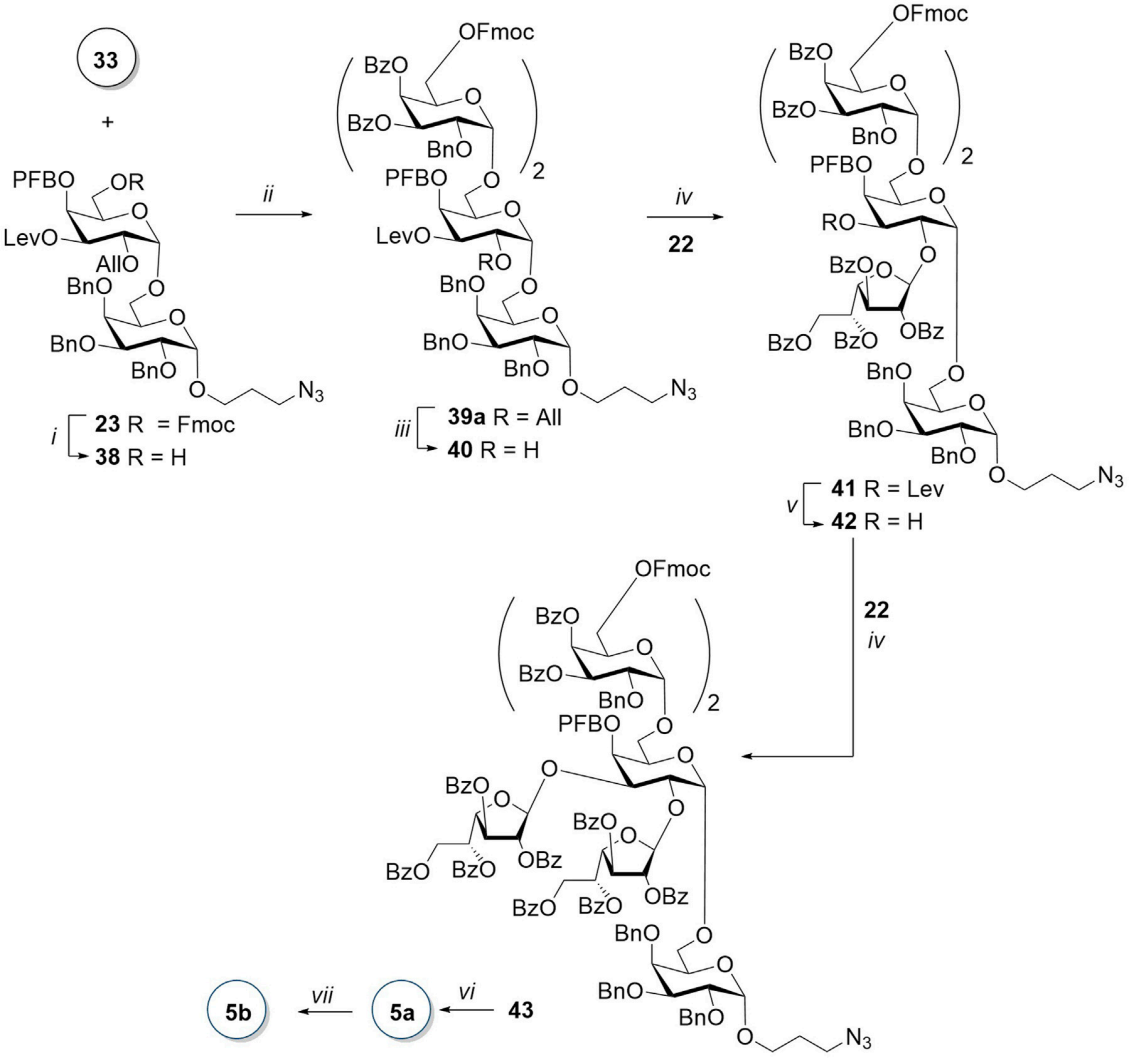
Synthesis of the branched hexasaccharide **5**. Reagents and conditions: (i) *N*-methylmorpholine, THF/CH_2_Cl_2_, 19 h, 78%; (ii) TfOH, MS AW300, CH_2_Cl_2_, -20 C → −8°C, 2 h, 55%, α:β = 13:1; (iii) [Ir(COD) (PMePh_2_)_2_]PF_6_, H_2_, I_2_, THF, 2 h, 86%; (iv) TMSOTf, AW300, CH_2_Cl_2_, -20°C, 84% for **41** and 87% for **43**; (v) NH_2_NH_2_∙H_2_O, AcOH, Py, 40 min, 89%; (vi) 1) *N*-methylmorpholine, THF/CH_2_Cl_2_, 48 h; 2) NaOMe MeOH, 48 h; and 3) H_2_, Pd(OH)_2_/C, HCl, MeOH, EtOAc, 7 h, 46% for the three steps; and (vii) AEB, Et_3_N, DMF, 1 h, 65%.

The synthesis of hexasaccharide **5a** began with the removal of 6-O-Fmoc in disaccharide **23** by N-methylmorpholine in a mixture of dichloromethane and THF (Scheme 6). The ^19^F NMR spectrum, as well as the absence of piperidine signals in the ^1^H spectrum, confirmed that the pentafluorobenzoyl group was not affected in this transformation. The TfOH-promoted glycosylation of the resulting acceptor **38** by disaccharide donor **33** was very slow and required a gradual increase in temperature from −20°C to −8°C. The low reaction rate and, accordingly, the accumulation of a large number of by-products of the destruction of the donor may be attributed to the presence of a strong electron-withdrawing PFB group in the immediate vicinity of the nucleophilic center in the acceptor. Attempts to vary the temperature regime of this reaction, as well as to change the promoter from TfOH to TMSOTf, *tert*-butyldimethylsilyl trifluoromethane sulfonate (TBDMSOTf), and C_4_F_9_SO_3_H, were not successful. After the separation of *α-* and *β-*isomers, obtained in a ratio of 13:1 in the reaction promoted with TfOH, tetrasaccharide **39a** was isolated by HPLC with a yield of 51%.

For the synthesis of the branched hexasaccharide, we first obtained acceptor **40**, which was then reacted with donor **22**. Monofuranosylated pentasaccharide **41** was isolated with a fairly high yield of 84% and only as a *β-*isomer. The introduction of a second galactofuranosyl residue after the removal of the levulinoyl-protecting group from O-3 resulted in a protected hexasaccharide **43** with an 87% yield and absolute *β-*stereoselectivity. The target hexasaccharide **5a** was obtained after the sequential removal of Fmoc and benzoate groups and hydrogenolysis, with a total yield of 46% for the three steps. Two singlets (δ 5.19 and 5.17 ppm) corresponding to H-1 of furanose rings are observed in the ^1^H NMR spectrum. Further notable are four doublets (δ 5.07–4.96 ppm) with *J* in the range 2.6–3.3 Hz, attributable to H-1 in pyranose residues of the main chain. Chemical shifts of the corresponding carbon atoms in the ^1^H–^13^C HSQC spectra also confirm the configurations of the anomeric centers of all six carbohydrate residues of compound **5a** (for ^13^C-NMR data of **1a–5a**, see Table 2).

**TABLE 2:** ^13^C-NMR chemical shifts (δ, ppm, D_2_O, 303 K) for oligosaccharides **1a–5a**.

Compound	Unit	C-1	C-2	C-3	C-4	C-5	C-6
**1a**	*β-D-Galf-(1→2)-*	109.98	82.00	77.21	83.28	71.18	63.25
	*→2)-α-D-Galp-(1→6)-*	99.21	77.06	69.02	69.84	71.41	61.70
	*→6)-α-D-Galp-Sp*	99.21	68.68	70.08	69.91	69.84	67.86
	*β-D-Galf-(1→3)-*	109.69	82.03	77.44	83.43	71.24	63.33
**2a**	*→3)-α-D-Galp-(1→6)-*	98.80	67.87	77.80	69.47	71.48	61.69
	*→6)-α-D-Galp-Sp*	99.18	68.70	70.04	69.86	69.86	67.13
**3a**	*β-D-Galf-(1→2)-*	109.85	82.14	77.40	83.26	71.31	63.21
	*β-D-Galf-(1→3)-*	109.58	82.05	77.72	83.62	71.31	63.34
	*→2) →3)-α-D-Galp-(1→6)-*	99.11	75.79	75.98	69.80	71.31	61.59
	*→6)-α-D-Galp-Sp*	99.19	68.68	70.08	69.88	70.01	67.76
**4a**	*α-D-Galp-(1→6)-*	98.38^*a*^	68.84^*b*^	70.05	70.05	71.55	61.71
	*→6)-α-D-Galp-(1→6)-*	98.38^*a*^	68.84^*b*^	70.05	68.88^*c*^	69.44^*d*^	67.06^*e*^
	*→6)-α-D-Galp-(1→6)-*	98.50^*a*^	68.84^*b*^	70.05	68.81^*c*^	69.22^*d*^	67.06^*e*^
	*→6)-α-D-Galp-(1→6)-*	98.60^*a*^	68.72^*b*^	70.05	68.88^*c*^	69.22^*d*^	67.06^*e*^
	*→6)-α-D-Galp-Sp*	99.13	68.75^*b*^	70.05	68.81^*c*^	69.55	67.12^*e*^
**5a**	*α-D-Galp-(1→6)-*	98.33	68.68^*f*^	69.96	70.06	71.59	61.79
	*→6)-α-D-Galp-(1→6)-*	98.63	68.94^*f*^	69.87^*f*^	68.87 ^*f*^	69.27^*f*^	67.00
	*β-D-Galf-(1→2)-*	109.59	82.07	77.46	83.61	71.28	63.21
	*β-D-Galf-(1→3)-*	109.87	82.17	77.70	83.38	71.28	63.39
	*→2) →3) →6)-α-D-Galp-(1→6)-*	98.66	76.01	75.79	70.27	69.87^*f*^	67.12
	*→6)-α-D-Galp-Sp*	99.19	69.96	69.16^*b*^	70.13	69.96	67.59

^*a-f*^The assignment is tentative within marked groups due to the overlap of signals and may be reversed.

Biotinylated oligosaccharides **1b**–**5b** were obtained by the treatment of aminopropyl glycosides **1a–5a** with an activated biotin ester and triethylamine in DMF according to the described procedure (Tsvetkov et al., 2012). Following purification on gel TSK-40 afforded **1b**–**5b** in good to excellent yields. The attachment of the biotin entity was confirmed by the presence of characteristic signals in the ^1^H NMR spectra (for NMR data of corresponding biotin conjugates, see the Supplementary Material) and by HRMS data.

### NMR analysis of obtained oligosaccharides 1a–5a

The NMR spectra of oligosaccharides **1a–5a** were totally assigned by applying 2D NMR experiments (Table 2. For ^1^H NMR shifts, see Supplementary Table S1 in Supplementary Material). The effects of glycosylation (Table 3, units are labeled A–D as in Figure 2) were calculated as the difference in the ^13^C chemical shifts between two structures, one with and one without a particular type of glycosylation, as described before (Dorokhova et al., 2021). Upon the introduction of a glycosylating residue, the most pronounced spectral effect was observed on the glycosylated carbons, which underwent a down-field shift by 5–8 ppm (*α-*effect), while the resonances of the adjacent carbon atoms moved up-field to a smaller extent (*β-*effect) (Lipkind et al., 1988; Shashkov et al., 1988; Kochetkov et al., 1991; Gerbst et al., 2015). The C-1 of the glycosylating residue also underwent a significant down-field shift. Other carbon resonances were much less affected and were excluded from consideration. The *α-* and *β-*glycosylation effects for *β-*(1→2)- and *β-*(1→3)-galactofuranosylation measured using trisaccharides **1a** and **2a** agreed well with previously reported data (Dorokhova et al., 2021). The deviations from additivity in the vicinally branched fragment of hexasaccharide **5a** (Table 4) were calculated as the difference between the experimental (δ_exp_) and calculated (δ_calc_) ^13^C chemical shifts, where δ_calc_ was calculated by the summation of all glycosylation effects.

**TABLE 3:** ^13^C NMR *α-* and *β-*glycosylation effects (Δδ, ppm) of a branched galactose fragment in hexasaccharide **5a** and related monoglycosylated oligosaccharides.

Glycosylation	Compound	Glycosylated Gal*p* (A)						Glycosylating unit		
		ΔδC-1	ΔδC-2	ΔδC-3	ΔδC-4	ΔδC-5	ΔδC-6	ΔδC-1B	ΔδC-1C	ΔδC-1D
*β-D-Galf-(1→2)-* ^*a*^		−0.56	7.87	−1.33	—	—	—	7.10	—	—
*β-D-Galf-(1→3)-* ^*a*^		—	−1.13	7.66	−0.17	—	—	—	6.93	—
*α-D-Galp-(1→6)-*	**4a**	—	—	—	—	−2.46	5.30	—	—	4.80
*β-D-Galf-(1→2)-* *β-D-Galf-(1→3)-* *α-D-Galp-(1→6)-*	**5a**	−0.49	7.30	5.93	0.27	−2.46	5.30	7.00	7.20	4.83

^*a*^Data from our previous paper (Dorokhova et al., 2021).

**TABLE 4:** Deviations from additivity (ΔΔδ, ppm) in the ^13^C-NMR spectra of a branched fragment in hexasaccharide **5a**.

Glycosylated Gal*p* (A)						Glycosylating unit		
ΔΔδC-1	ΔΔδC-2	ΔΔδC-3	ΔΔδC-4	ΔΔδC-5	ΔΔδC-6	ΔΔδC-1B	ΔΔδC-1C	ΔΔδC-1D
−0.07	−0.56	0.40	−0.44	0.00	0.00	−0.10	0.27	−0.03

In spite of the presence of 2,3-vicinal branching and 1,2-*cis*-pseudobranching in hexasaccharide **5a**, the good agreement between theoretical and experimental ^13^C chemical shifts was determined (deviation from additivity did not exceed 0.56 ppm; Table 4). It suggests the independence of conformational flexibility around corresponding interunit linkages connected with the branched Gal*p*-residue of **5a**. These suggest that the spatial similarity of **5a** to the corresponding fragment within the chain of GXMGal makes **5a** a the reliable model for future immunological studies of *C. neoformans*.

## Conclusion

In conclusion, the oligosaccharides **1a–5a** and their biotinylated derivatives **1b**–**5b** were synthesized according to the convergent scheme, achieving good to excellent yields at each step. The sequential introduction of *β-*galactofuranosyl residues at O-2 and/or O-3, along with the elongation of the *α-*(1→6)-galactopyranoside core chain, was achieved using a galactosyl donor bearing orthogonal groups: 2-O-allyl, 3-O-levulinoyl, and Fmoc group at O-6. High, nearly absolute *α-*stereoselectivity in each glycosylation step was achieved due to the joint stereoredirecting effects of O-protecting acyl groups in galactosyl donors, including the 4-O-pentafluorobenzoyl group. The analysis of ^13^C NMR shifts and corresponding glycosylation effects for oligosaccharides **1a–5a** confirms the spatial equivalence of synthetic hexasaccharide **5a** to the corresponding branched fragment of the polysaccharide GXMGal, supporting the use of **5a** as a reliable model hapten for further immunological studies of *C. neoformans*.

## Data Availability

The original contributions presented in the study are included in the article/Supplementary Material, further inquiries can be directed to the corresponding author.

## References

[B1] Ágoston K. Streicher H. Fügedi P. (2016). Orthogonal protecting group strategies in carbohydrate chemistry. Tetrahedron Asymmetry 27, 707–728. 10.1016/j.tetasy.2016.06.010

[B2] Argunov D. A. Aladysheva U. S. Krylov V. B. Nifantiev N. E. (2024). Acid-catalyzed transformation of pyranosides into furanosides as a tool for preparation of furanoside synthetic blocks. Org. Lett. 26, 8090–8094. 10.1021/acs.orglett.4c02984 39269779

[B3] Argunov D. A. Krylov V. B. Nifantiev N. E. (2016). The use of pyranoside-into-furanoside rearrangement and controlled O(5) → O(6) benzoyl migration as the basis of a synthetic strategy to assemble (1→5)- and (1→6)-linked galactofuranosyl chains. Org. Lett. 18, 5504–5507. 10.1021/acs.orglett.6b02735 27759393

[B4] Baek J. Y. Kwon H.-W. Myung S. J. Park J. J. Kim M. Y. Rathwell D. C. K. (2015). Directing effect by remote electron-withdrawing protecting groups at O-3 or O-4 position of donors in glucosylations and galactosylations. Tetrahedron 71, 5315–5320. 10.1016/j.tet.2015.06.014

[B5] Bermas A. Geddes-McAlister J. (2020). Combatting the evolution of antifungal resistance in Cryptococcus neoformans. Mol. Microbiol. 114, 721–734. 10.1111/mmi.14565 32697029

[B6] Calin O. Eller S. Hahm H. S. Seeberger P. H. (2013). Total synthesis of the *Escherichia coli* O111 O-specific polysaccharide repeating unit. Chem. – Eur. J. 19, 3995–4002. 10.1002/chem.201204394 23447496

[B7] Cato D. Buskas T. Boons G. (2005). Highly efficient stereospecific preparation of tn and TF building blocks using thioglycosyl donors and the Ph2SO/Tf2O promotor system. J. Carbohydr. Chem. 24, 503–516. 10.1081/CAR-200067091

[B8] Chen Y. Shi Z. W. Strickland A. B. Shi M. (2022). Cryptococcus neoformans infection in the central nervous system: the battle between host and pathogen. J. Fungi 8, 1069. 10.3390/jof8101069 PMC9605252 36294634

[B9] Cherniak R. Reiss E. Slodki M. E. Plattner R. D. Blumer S. O. (1980). Structure and antigenic activity of the capsular polysaccharide of Cryptococcus neoformans serotype A. Mol. Immunol. 17, 1025–1032. 10.1016/0161-5890(80)90096-6 6777664

[B10] Cherniak R. Reiss E. Turner S. H. (1982). A galactoxylomannan antigen of Cryptococcus neoformans serotype A. Carbohydr. Res. 103, 239–250. 10.1016/S0008-6215(00)80686-2

[B11] Cherniak R. Valafar H. Morris L. C. Valafar F. (1998). Cryptococcus neoformans chemotyping by quantitative analysis of ^1^H nuclear magnetic resonance spectra of glucuronoxylomannans with a computer-simulated artificial neural network. Clin. Diagn. Lab. Immunol. 5, 146–159. 10.1128/CDLI.5.2.146-159.1998 9521136 PMC121351

[B12] Decote-Ricardo D. LaRocque-de-Freitas I. F. Rocha J. D. B. Nascimento D. O. Nunes M. P. Morrot A. (2019). Immunomodulatory role of capsular polysaccharides constituents of Cryptococcus neoformans. Front. Med. 6, 129. 10.3389/fmed.2019.00129 PMC6593061 31275938

[B13] Doering T. L. (2009). How sweet it is! Cell wall biogenesis and polysaccharide capsule formation in Cryptococcus neoformans. Annu. Rev. Microbiol. 63, 223–247. 10.1146/annurev.micro.62.081307.162753 19575556 PMC2880894

[B14] Dorokhova V. S. Gerbst A. G. Komarova B. S. Previato J. O. Previato L. M. Dmitrenok A. S. (2021). Synthesis and conformational analysis of vicinally branched trisaccharide β-d-Gal*f*-(1 → 2)-[β-d-Gal*f*-(1 → 3)-]-α-Gal*p* from *Cryptococcus neoformans* galactoxylomannan. Org. Biomol. Chem. 19, 2923–2931. 10.1039/D0OB02071K 33471013

[B15] Gerbst A. G. Krylov V. B. Vinnitskiy D. Z. Dmitrenok A. S. Shashkov A. S. Nifantiev N. E. (2015). ^13^C-NMR glycosylation effects in (1→3)-linked furanosyl-pyranosides. Carbohydr. Res. 417, 1–10. 10.1016/j.carres.2015.08.014 26382080

[B16] Gerbst A. G. Ustuzhanina N. E. Grachev A. A. Khatuntseva E. A. Tsvetkov D. E. Whitfield D. M. (2001). Synthesis, nmr, and conformational studies of fucoidan fragments. III. Effect of benzoyl group at O-3 on stereoselectivity of glycosylation by 3-O- and 3,4-di-O-benzoylated 2-O-benzylfucosyl bromides. J. Carbohydr. Chem. 20, 821–831. 10.1081/CAR-100108659

[B17] Hargett A. A. Azurmendi H. F. Crawford C. J. Wear M. P. Oscarson S. Casadevall A. (2024). The structure of a *C. neoformans* polysaccharide motif recognized by protective antibodies: a combined NMR and MD study. Proc. Natl. Acad. Sci. 121, e2315733121. 10.1073/pnas.2315733121 38330012 PMC10873606

[B18] Heiss C. Skowyra M. L. Liu H. Klutts J. S. Wang Z. Williams M. (2013). Unusual galactofuranose modification of a capsule polysaccharide in the pathogenic yeast Cryptococcus neoformans. J. Biol. Chem. 288, 10994–11003. 10.1074/jbc.M112.441998 23408430 PMC3630864

[B19] Heiss C. Stacey Klutts J. Wang Z. Doering T. L. Azadi P. (2009). The structure of Cryptococcus neoformans galactoxylomannan contains β-d-glucuronic acid. Carbohydr. Res. 344, 915–920. 10.1016/j.carres.2009.03.003 19345342 PMC2695399

[B20] Hettikankanamalage A. A. Lassfolk R. Ekholm F. S. Leino R. Crich D. (2020). Mechanisms of stereodirecting participation and ester migration from near and far in glycosylation and related reactions. Chem. Rev. 120, 7104–7151. 10.1021/acs.chemrev.0c00243 32627532 PMC7429366

[B21] Hirose H. Tamai H. Gao C. Imamura A. Ando H. Ishida H. (2015). Total syntheses of disulphated glycosphingolipid SB1a and the related monosulphated SM1a. Org. Biomol. Chem. 13, 11105–11117. 10.1039/C5OB01744K 26399908 PMC4920060

[B22] James P. G. Cherniak R. (1992). Galactoxylomannans of Cryptococcus neoformans. Infect. Immun. 60, 1084–1088. 10.1128/iai.60.3.1084-1088.1992 1541523 PMC257597

[B23] Kochetkov N. K. Lipkind G. M. Shashkov A. S. Nifantiev N. E. (1991). N.m.r. and conformational analysis of some 2,3-disubstituted methyl α-L-rhamnopyranosides. Carbohydr. Res. 221, 145–168. 10.1016/0008-6215(91)80053-P 1816916

[B24] Komarova B. S. Dorokhova V. S. Tsvetkov Y. E. Nifantiev N. E. (2018a). Synthesis of a biotinylated penta-α-(1→6)-D-glucoside based on the rational design of an α-stereoselective glucosyl donor. Org. Chem. Front. 5, 909–928. 10.1039/C7QO01007A

[B25] Komarova B. S. Novikova N. S. Gerbst A. G. Sinitsyna O. A. Rubtsova E. A. Kondratyeva E. G. (2023). Combination of 3-O-levulinoyl and 6-O-trifluorobenzoyl groups ensures α-selectivity in glucosylations: synthesis of the oligosaccharides related to Aspergillus fumigatus α-(1 → 3)-D-glucan. J. Org. Chem. 88, 12542–12564. 10.1021/acs.joc.3c01283 37593939

[B26] Komarova B. S. Orekhova M. V. Tsvetkov Y. E. Beau R. Aimanianda V. Latgé J. (2015). Synthesis of a pentasaccharide and neoglycoconjugates related to fungal α‐(1→3)‐glucan and their use in the generation of antibodies to trace Aspergillus fumigatus cell wall. Chem. – Eur. J. 21, 1029–1035. 10.1002/chem.201404770 25376936

[B27] Komarova B. S. Orekhova M. V. Tsvetkov Y. E. Nifantiev N. E. (2014). Is an acyl group at O-3 in glucosyl donors able to control α-stereoselectivity of glycosylation? The role of conformational mobility and the protecting group at O-6. Carbohydr. Res. 384, 70–86. 10.1016/j.carres.2013.11.016 24368161

[B28] Komarova B. S. Tsvetkov Y. E. Nifantiev N. E. (2016). Design of α-selective glycopyranosyl donors relying on remote anchimeric assistance. Chem. Rec. 16, 488–506. 10.1002/tcr.201500245 26785933

[B29] Komarova B. S. Wong S. S. W. Orekhova M. V. Tsvetkov Y. E. Krylov V. B. Beauvais A. (2018b). Chemical synthesis and application of biotinylated oligo-α-(1→3)-D-glucosides to study the antibody and cytokine response against the cell wall α-(1→3)-D-glucan of Aspergillus fumigatus. J. Org. Chem. 83, 12965–12976. 10.1021/acs.joc.8b01142 30277398 PMC6461050

[B30] Krylov V. B. Argunov D. A. Solovev A. S. Petruk M. I. Gerbst A. G. Dmitrenok A. S. (2018). Synthesis of oligosaccharides related to galactomannans from Aspergillus fumigatus and their NMR spectral data. Org. Biomol. Chem. 16, 1188–1199. 10.1039/C7OB02734F 29376539

[B31] Krylov V. B. Solovev A. S. Puchkin I. A. Yashunsky D. V. Antonets A. V. Kutsevalova O. Y. (2021). Reinvestigation of carbohydrate specificity of EBCA-1 monoclonal antibody used for the detection of Candida mannan. J. Fungi 7, 504. 10.3390/jof7070504 PMC8303853 34202579

[B32] Laroussarie A. Barycza B. Andriamboavonjy H. Tamigney Kenfack M. Blériot Y. Gauthier C. (2015). Synthesis of the tetrasaccharide repeating unit of the β-kdo-containing exopolysaccharide from *Burkholderia pseudomallei* and *B. cepacia* complex. J. Org. Chem. 80, 10386–10396. 10.1021/acs.joc.5b01823 26375291

[B33] Lipkind G. M. Shashkov A. S. Knirel Y. A. Vinogradov E. V. Kochetkov N. K. (1988). A computer-assisted structural analysis of regular polysaccharides on the basis of ^13^C-n.m.r. data. Carbohydr. Res. 175, 59–75. 10.1016/0008-6215(88)80156-3 3378242

[B34] Maziarz E. K. Perfect J. R. (2016). Cryptococcosis. Infect. Dis. Clin. 30, 179–206. 10.1016/j.idc.2015.10.006 PMC5808417 26897067

[B35] McFadden D. C. Casadevall A. (2004). Unexpected diversity in the fine specificity of monoclonal antibodies that use the same V region gene to glucuronoxylomannan of Cryptococcus neoformans. J. Immunol. 172, 3670–3677. 10.4049/jimmunol.172.6.3670 15004170

[B36] Nakouzi A. Zhang T. Oscarson S. Casadevall A. (2009). The common Cryptococcus neoformans glucuronoxylomannan M2 motif elicits non-protective antibodies. Vaccine 27, 3513–3518. 10.1016/j.vaccine.2009.03.089 19464529 PMC2932445

[B37] Nigudkar S. Demchenko V. (2015). Stereocontrolled 1,2-cis-glycosylation as the driving force of progress in synthetic carbohydrate chemistry. Chem. Sci. 6, 2687–2704. 10.1039/C5SC00280J 26078847 PMC4465199

[B38] Oberli M. A. Bindschädler P. Werz D. B. Seeberger P. H. (2008). Synthesis of a hexasaccharide repeating unit from Bacillus anthracis vegetative cell walls. Org. Lett. 10, 905–908. 10.1021/ol7030262 18232704

[B39] Oscarson S. Alpe M. Svahnberg P. Nakouzi A. Casadevall A. (2005). Synthesis and immunological studies of glycoconjugates of Cryptococcus neoformans capsular glucuronoxylomannan oligosaccharide structures. Vaccine 23, 3961–3972. 10.1016/j.vaccine.2005.02.029 15917118

[B40] Paccoud O. Desnos-Ollivier M. Cassaing S. Boukris-Sitbon K. Alanio A. Bellanger A.-P. (2023). *Cryptococcus neoformans* infections differ among human immunodeficiency virus (HIV)–Seropositive and HIV-seronegative individuals: results from a nationwide surveillance program in France. Open Forum Infect. Dis. 11, ofad658. 10.1093/ofid/ofad658 38344129 PMC10854213

[B41] Peltier P. Euzen R. Daniellou R. Nugier-Chauvin C. Ferrières V. (2008). Recent knowledge and innovations related to hexofuranosides: structure, synthesis and applications. Carbohydr. Res. 343, 1897–1923. 10.1016/j.carres.2008.02.010 18440497

[B42] Pennington M. W. Dunn B. M. (1994). Peptide synthesis protocols (Totowa, N.J: Humana Press).

[B43] Prabhu A. Venot A. Boons G.-J. (2003). New set of orthogonal protecting groups for the modular synthesis of heparan sulfate fragments. Org. Lett. 5, 4975–4978. 10.1021/ol0359261 14682743

[B44] Previato J. O. Vinogradov E. Maes E. Fonseca L. M. Guerardel Y. Oliveira P. A. V. (2017). Distribution of the O-acetyl groups and β-galactofuranose units in galactoxylomannans of the opportunistic fungus Cryptococcus neoformans. Glycobiology 27, 582–592. 10.1093/glycob/cww127 27986834

[B45] Rajasingham R. Govender N. P. Jordan A. Loyse A. Shroufi A. Denning D. W. (2022). The global burden of HIV-associated cryptococcal infection in adults in 2020: a modelling analysis. Lancet Infect. Dis. 22, 1748–1755. 10.1016/S1473-3099(22)00499-6 36049486 PMC9701154

[B46] Richards M. R. Lowary T. L. (2009). Chemistry and biology of galactofuranose-containing polysaccharides. ChemBioChem 10, 1920–1938. 10.1002/cbic.200900208 19591187

[B47] Rivera J. Feldmesser M. Cammer M. Casadevall A. (1998). Organ-dependent variation of capsule thickness in Cryptococcus neoformans during experimental murine infection. Infect. Immun. 66, 5027–5030. 10.1128/iai.66.10.5027-5030.1998 9746613 PMC108624

[B48] Shashkov A. S. Lipkind G. M. Knirel Y. A. Kochetkov N. K. (1988). Stereochemical factors determining the effects of glycosylation on the ^13^C chemical shifts in carbohydrates. Magn. Reson. Chem. 26, 735–747. 10.1002/mrc.1260260904

[B49] Skelton M. A. Cherniak R. Poppe L. van Halbeek H. (1991a). Structure of the de-O-acetylated glucuronoxylomannan from Cryptococcus neoformans serotype D, as determined by 2D NMR spectroscopy. Magn. Reson. Chem. 29, 786–793. 10.1002/mrc.1260290808

[B50] Skelton M. A. van Halbeek H. Cherniak R. (1991b). Complete assignment of the ^1^H- and ^13^C-n.m.r. spectra of the O-deacetylated glucuronoxylomannan from Cryptococcus neoformans serotype B. Carbohydr. Res. 221, 259–268. 10.1016/0008-6215(91)80062-R 1816923

[B51] Tefsen B. Ram A. F. van Die I. Routier F. H. (2012). Galactofuranose in eukaryotes: aspects of biosynthesis and functional impact. Glycobiology 22, 456–469. 10.1093/glycob/cwr144 21940757

[B52] Tokatly A. I. Vinnitskiy D. Z. Ustuzhanina N. E. Nifantiev N. E. (2021). Protecting groups as a factor of stereocontrol in glycosylation reactions. Russ. J. Bioorg. Chem. 47, 53–70. 10.1134/S1068162021010258

[B53] Tsvetkov Y. E. Burg-Roderfeld M. Loers G. Ardá A. Sukhova E. V. Khatuntseva E. A. (2012). Synthesis and molecular recognition studies of the HNK-1 trisaccharide and related oligosaccharides. The specificity of monoclonal anti-HNK-1 antibodies as assessed by surface plasmon resonance and STD NMR. J. Am. Chem. Soc. 134, 426–435. 10.1021/ja2083015 22087768

[B54] Turco S. J. Pedersen L. L. (2003). Galactofuranose metabolism: a potential target for antimicrobial chemotherapy. Cell. Mol. Life Sci. CMLS 60, 259–266. 10.1007/s000180300021 12678491 PMC11138809

[B66] Thijssen M. J. van Rijswijk M. N. Kamerling J. P. Vliegenthart J. F. (1998). Synthesis of spacer-containing di-and tri-saccharides that represent parts of the capsular polysaccharide of Streptococcus pneumoniae type 6B. Carbohydrate. Research. 306, 93–109. 10.1016/S0008-6215(97)00271-1 9691442

[B55] Vaishnav V. V. Bacon B. E. O’Neill M. Cherniak R. (1998). Structural characterization of the galactoxylomannan of Cryptococcus neoformans Cap67. Carbohydr. Res. 306, 315–330. 10.1016/S0008-6215(97)10058-1 9691456

[B56] Vecchiarelli A. (2000). Immunoregulation by capsular components of Cryptococcus neoformans. Med. Mycol. 38, 407–417. 10.1080/mmy.38.6.407.417 11204878

[B57] Vecchiarelli A. Pericolini E. Gabrielli E. Chow S.-K. Bistoni F. Cenci E. (2011). Cryptococcus neoformans galactoxylomannan is a potent negative immunomodulator, inspiring new approaches in anti-inflammatory immunotherapy. Immunotherapy 3, 997–1005. 10.2217/imt.11.86 21843086

[B58] Villena S. N. Pinheiro R. O. Pinheiro C. S. Nunes M. P. Takiya C. M. DosReis G. A. (2008). Capsular polysaccharides galactoxylomannan and glucuronoxylomannan from Cryptococcus neoformans induce macrophage apoptosis mediated by Fas ligand. Cell. Microbiol. 10, 1274–1285. 10.1111/j.1462-5822.2008.01125.x 18284419

[B59] Vinnitskiy D. Z. Krylov V. B. Ustyuzhanina N. E. Dmitrenok A. S. Nifantiev N. E. (2015). The synthesis of heterosaccharides related to the fucoidan from Chordaria flagelliformis bearing an α-L-fucofuranosyl unit. Org. Biomol. Chem. 14, 598–611. 10.1039/C5OB02040A 26536063

[B60] Vohra Y. Buskas T. Boons G.-J. (2009). Rapid assembly of oligosaccharides: a highly convergent strategy for the assembly of a glycosylated amino acid derived from PSGL-1. J. Org. Chem. 74, 6064–6071. 10.1021/jo901135k 19606831 PMC2888837

[B61] Werz D. B. (2012). in Chemical synthesis of carbohydrates and their surface immobilization: a brief introduction. Editors Microarrays C. Chevolot Y. (Totowa, NJ: Humana Press), 13–29. 10.1007/978-1-61779-373-8_2 22057515

[B62] Zhang Y. Hu Y. Liu S. He H. Sun R. Lu G. (2022). Total synthesis of Lentinus giganteus glycans with antitumor activities via stereoselective α-glycosylation and orthogonal one-pot glycosylation strategies. Chem. Sci. 13, 7755–7764. 10.1039/D2SC02176E 35865907 PMC9258330

[B63] Zhao Y. Ye L. Zhao F. Zhang L. Lu Z. Chu T. (2023). Cryptococcus neoformans, a global threat to human health. Infect. Dis. Poverty 12, 20. 10.1186/s40249-023-01073-4 36932414 PMC10020775

[B64] Zhu S.-Y. Yang J.-S. (2012). Synthesis of tetra- and hexasaccharide fragments corresponding to the O-antigenic polysaccharide of *Klebsiella pneumoniae* . Tetrahedron 68, 3795–3802. 10.1016/j.tet.2012.03.074

[B65] Zou X. Qin C. Pereira C. L. Tian G. Hu J. Seeberger P. H. (2018). Synergistic glycosylation as key to the chemical synthesis of an outer core octasaccharide of Helicobacter pylori. Chem. – Eur. J. 24, 2868–2872. 10.1002/chem.201800049 29319212

