# Supplementary material for: Synthesis of branched and linear galactooligosaccharides related to glucuronoxylomannogalactan of Cryptococcus neoformans
Source: Front Chem. 2024 Nov 14;12:1501766. doi: 10.3389/fchem.2024.1501766 (PMC11602299; doi:10.3389/fchem.2024.1501766)

Supplementary Material

Synthesis of branched and linear galactooligosaccharides related to glucuronoxylomannogalactan (GXMGal) of *Cryptococcus neoformans*

Vera S. Dorokhova[1], Bozhena S. Komarova[1], José O. Previato[2], Lúcia Mendonça Previato[2], Vadim B. Krylov[1,3], Nikolay E. Nifantiev[1]*

[1] Laboratory of Glycoconjugate Chemistry, N.D. Zelinsky Institute of Organic Chemistry, Russian Academy of Sciences, Moscow, Russia.

[2] Laboratório de Glicobiologia, Instituto de Biofísica Carlos Chagas Filho, Universidade Federal do Rio de Janeiro, Rio de Janeiro, Brazil.

[3] Laboratory of Synthetic Glycovaccines, N.D. Zelinsky Institute of Organic Chemistry, Russian Academy of Sciences, Moscow, Russia

Table S1. ^{1}H-NMR data for target oligosaccharides **1a – 5a** (600 MHz, D_2O, 303K).

Unit	H-1	H-2	H-3	H-4	H-5	H-6A	H-6B
			Trisaccharide **1a**				
β-D-Galf-(1→2)-	5.09	3.82	3.91	4.01	3.99	3.76 – 3.71	
→2)-α-D-Galp-(1→6)-	4.95	3.83	3.83	4.01	4.09	3.86	3.71
→6)-α-D-Galp-Sp	5.13	4.15	4.07	3.98	3.83	3.71 – 3.64	
			Trisaccharide **2a**				
β-D-Galf-(1→3)-	4.99	3.95	3.88	4.11	4.00	3.75 – 3.71	
→3)-α-D-Galp-(1→6)-	4.96	3.84	3.85	4.03	4.11	3.87	3.73
→6)-α-D-Galp-Sp	5.18	4.18	4.06	4.03	3.83	3.69	3.64
			Tetrasaccharide **3a**				
β-D-Galf-(1→2)-	5.07	3.98	3.98	4.10	4.02	3.75 – 3.71	
β-D-Galf-(1→3)-	4.96	3.84	3.84	4.03	4.11	3.87	3.74
→2) →3)-α-D-Galp-(1→6)-	5.15	4.13	4.07	3.99	3.85 – 3.82	3.71 – 3.67	
→6)-α-D-Galp-Sp	5.19	4.16	4.06	4.02		3.69 – 3.62	
			Pentasaccharide **4a**				
α-D-Galp-(1→6)-	4.96	3.87 – 3.79	4.02 – 3.93	3.84 – 3.76	3.96	3.74 – 3.69	
→6)-α-D-Galp-(1→6)-	4.97 – 4.91				4.19 – 4.12	3.88 – 3.81	3.73 – 3.61
→6)-α-D-Galp-(1→6)-							
→6)-α-D-Galp-(1→6)-							
→6)-α-D-Galp-Sp	4.93				4.08		
			Hexasaccharide **5a**				
β-D-Galf-(1→2)-	5.19	4.17	4.06	4.03	3.85 – 3.81	3.71 – 3.66	
β-D-Galf-(1→3)-	5.17	4.14	4.05	3.98	3.85 – 3.81	3.67	3.64
α-D-Galp-(1→6)-	4.99	3.86 – 3.80	4.02	3.85	3.99	3.76 – 3.72	
→6)-α-D-Galp-(1→6)-	5.00		4.21	3.86 – 3.80	3.98	3.90	3.66
→2) →3) →6)-α-D-Galp-(1→6)-	5.07	4.01 – 3.96		4.12	4.18	3.86	3.73
→6)-α-D-Galp-Sp	4.96	3.85	3.83	3.99	4.11	3.93	3.66

Table S2. ^{13}C-NMR data for target oligosaccharides **1a – 5a** (150 MHz, D_2O, 303K).

Trisaccharide **1a**

Unit	C-1	C-2	C-3	C-4	C-5	C-6
β-D-Galf-(1→2)-	99.21	77.06	69.02	69.84	71.41	61.70
→2)-α-D-Galp-(1→6)-	99.21	68.68	70.08	69.91	69.84	67.86
→6)-α-D-Galp-Sp	109.98	82.00	77.21	83.28	71.18	63.25

Trisaccharide **2a**

Unit	C-1	C-2	C-3	C-4	C-5	C-6
β-D-Galf-(1→3)-	98.80	67.87	77.80	69.47	71.48	61.69
→3)-α-D-Galp-(1→6)-	99.18	68.70	70.04	69.86	69.86	67.13
→6)-α-D-Galp-Sp	109.69	82.03	77.44	83.43	71.24	63.33

Tetrasaccharide **3a**

Unit	C-1	C-2	C-3	C-4	C-5	C-6
β-D-Galf-(1→2)-	99.11	75.79	75.98	69.80	71.31	61.59
β-D-Galf-(1→3)-	99.19	68.68	70.08	69.88	70.01	67.76
→2) →3)-α-D-Galp-(1→6)-	109.85	82.14	77.40	83.26	71.31	63.21
→6)-α-D-Galp-Sp	109.58	82.05	77.72	83.62	71.31	63.34

Pentasaccharide **4a**

Unit	C-1	C-2	C-3	C-4	C-5	C-6
α-D-Galp-(1→6)-	98.38 [a]	68.84 [c]	70.05 [b]	70.05	71.55	61.71
→6)-α-D-Galp-(1→6)-	98.38 [a]	70.05	69.88 [b]	68.72 [c]	69.44 [d]	67.06 [e]
→6)-α-D-Galp-(1→6)-	98.50 [a]	70.05	69.88 [b]	68.84 [c]	69.22 [d]	67.06 [e]
→6)-α-D-Galp-(1→6)-	98.60 [a]	70.05	69.81 [b]	68.65 [c]	69.22 [d]	67.06 [e]
→6)-α-D-Galp-Sp	99.13	70.05	69.81 [b]	68.84 [c]	69.55	67.12 [e]

Hexasaccharide **5a**

Unit	C-1	C-2	C-3	C-4	C-5	C-6
β-D-Galf-(1→2)-	109.59	82.07	77.46	83.61	71.28	63.21
β-D-Galf-(1→3)-	109.87	82.17	77.70	83.38	71.28	63.39
α-D-Galp-(1→6)-	98.33	68.68 [f]	69.96	70.06	71.59	61.79
→6)-α-D-Galp-(1→6)-	98.63	68.94 [f]	69.87 [f]	68.87 [f]	69.27 [f]	67.00
→2) →3) →6)-α-D-Galp-(1→6)-	98.66	76.01	75.79	70.27	69.87 [f]	67.12
→6)-α-D-Galp-Sp	99.19	69.96	69.16 [b]	70.13	69.96	67.59

[a-f] The assignment is tentative within marked groups due to the overlap of signals and may be reversed.

I. General methods

All glycosylation reactions were carried out in an atmosphere of dry Ar. Molecular sieves for glycosylation reactions were activated prior to application at 150 °C under vacuum of an oil pump for 2 h. Dichloromethane was successively distilled from diethanolamine, P_2O_5, and CaH_2 under Ar. Analytical TLC was performed on Silica Gel 60 F254 aluminium sheets (Merck), and visualization was accomplished using UV light or by charring at 150 °C with 10% (v/v) H_3PO_4 in isopropyl alcohol. Column chromatography was performed on Silica Gel 60, 40–63 μm (Merck). Gel-permeation chromatography was carried out on TSK HW-40(S) columns in 0.1 M AcOH using a K-2401 (Knauer) refractometer to monitor the eluate. Optical rotations were measured using a JASCO P-2000 polarimeter in $CHCl_3$ (C = 10 mg/ml) unless otherwise specified. NMR spectra were recorded on a Bruker AV-400, Bruker AV-600, and Bruker Avance spectrometers. The spectra of the protected carbohydrate derivatives were recorded for solutions in $CDCl_3$ or in the mixture of $CDCl_3$ and CD_3OD (for partly protected compounds). Chemical shifts in ^{1}H- and ^{13}C-NMR spectra were referenced to the residual solvent signals. The NMR spectra of free oligosaccharide derivatives were recorded for solutions in D_2O using acetonitrile (δH 2.06, δC 1.47 and 119.68 ppm) as the internal standard. Signal assignment in the ^{1}H and ^{13}C NMR spectra was made using COSY, ^{1}H–^{13}C HSQC, HMBC, TOCSY and NOESY techniques. In the description of the NMR spectra of oligosaccharides galactofuranose residues are denoted as "I" for 2-O-Galf units and "II" for 3-O-Galf units. Galactopyranoside residues are labeled from "1" to "5" starting from the reducing end. The HRMS (ESI) were obtained on a MicrOTOF II (Bruker Daltonics) instrument. All the reagents were obtained from Merck and used as it is except for 2,2,2-trifluoro-*N*-phenylacetimidoyl chloride[1].

II. Synthesis of monosaccharide donors 13 and 17 and acceptor 15

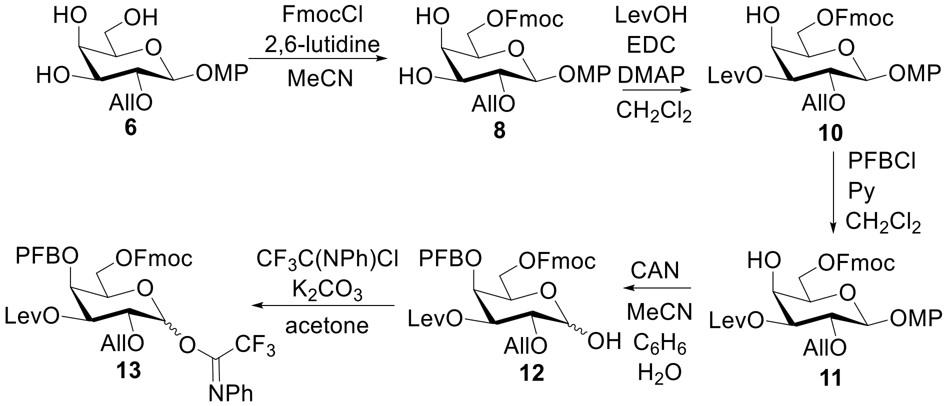

p-Methoxyphenyl 2-*O*-allyl-6-*O*-fluorenylmethyloxycarbonyl-β-D-galactopyranoside (8)

Compound **6**[2] (5.29 g, 16.21 mmol) was dissolved in dry acetonitrile (60 mL), then FmocCl (5.25 g, 20.27 mmol) and 2,6-lutidine (3.8 mL, 32.42 mmol) were added under an argon atmosphere and the mixture was stirred for 48 hours. Then the solvent was evaporated and the dry residue was purified by column chromatography (silica gel, toluene : acetone 30:1 → 9:1) to give white powder **8** (5.77 g, 65%). R_f = 0.40 (toluene : acetone 2 : 1). $[\alpha]_D^{23}$ = -2.0.

^{1}H NMR (300 Hz, $CDCl_3$): δ 7.80 (d, *J* = 7.6 Hz, 2H, Fmoc), 7.63 (d, *J* = 7.6 Hz, 2H, Fmoc), 7.44 (t, *J* = 7.4 Hz, 2H, Fmoc), 7.33 (d, *J* = 7.4 Hz, 2H, Fmoc), 7.05 (d, *J* = 9.2 Hz, 2H, Ph (MP)), 6.83 (d, *J* = 9.2 Hz, 2H, Ph (MP)), 6.00 (m, 1H, $CH_2CH{=}CH_2$), 5.36 (dd, *J* = 1.6 Hz, *J* = 17.2 Hz, $CH_2CH{=}CHH'$), 5.25 (dd, *J* = 1.6 Hz, *J* = 10.2 Hz, $CH_2CH{=}CHH'$), 4.82 (d, $J_{1,2}$ = 7.7 Hz, 1H, H-1), 4.57 (m, 1H, $CHH'CH{=}CH_2$), 4.52 – 4.42 (m, 4H, H-6A, H-6B, CH_2 (Fmoc)), 4.37 – 4.23 (m, 2H, CH (Fmoc),

CH*H'*CH=CH$_2$), 4.02 (br s, 1H, H-4), 3.83 (t, $J_{5,6A}$ = $J_{5,6B}$ = 6.5 Hz, 1H, H-5), 3.75 (s, 3H, OMe (MP)), 3.74 – 3.70 (m, 2H, H-2, H-3), 2.85 (br s, 1H, OH), 2.74 (br s, 1H, OH).

$^{13}C\{^{1}H\}$ NMR (75 Hz, $CDCl_3$): δ 155.5 (*ipso*-Ph (MP)), 155.1 (*ipso*-Fmoc), 151.3 (*ipso*-Ph (MP)), 143.3, 143.2, 141.3 (Fmoc), 134.6 (CH$_2$*C*H=CH$_2$), 128.0, 127.2, 125.1, 120.1 (Fmoc), 118.5 (Ph (MP)), 117.9 (CH$_2$CH=*C*H$_2$), 114.6 (Ph (MP)), 102.8 (C-1), 78.4 (C-2), 73.8 (*C*H$_2$CH=CH$_2$), 72.8 (C-3), 72.3 (C-5), 70.1 (CH$_2$ (Fmoc)), 68.1 (C-4), 66.2 (C-6), 55.6 (OMe (MP)), 46.8 (CH (Fmoc)).

HRMS ESI m/z calcd for $[M+Na]^+$ $C_{31}H_{32}O_9$ 571.1939; found 571.1936.

p-Methoxyphenyl 2-*O*-allyl-6-*O*-fluorenylmethyloxycarbonyl-3-*O*-levulinyl-β-D-galactopyranoside (10)

Compound **8** (1.50 g, 2.74 mmol) was dissolved in dry CH_2Cl_2 (150 mL) and the solution was cooled down to -18 °C. Then levulinic acid (390 µL, 3.83 mmol), 1-ethyl-3-(3-dimethylaminopropyl)carbodiimide hydrochloride (518 mg, 3.83mmol) and DMAP (20 mg) were added and the mixture was stirred for 20 hours at -18 °C under argon atmosphere. Then the reaction mixture was diluted with CH_2Cl_2 and washed with brine. The aqueous phase was washed with CH_2Cl_2 three times; the combined organic extracts were dried over anhydrous Na_2SO_4, filtered and concentrated under vacuum. Column chromatography of the residue (silica gel, toluene : EtOAc 30:1 → 3:1) provided **10** (1.54 g, 87%) as a colorless oil. R_f = 0.33 (toluene : EtOAc 2 : 1). $[\alpha]_D^{24}$ = 9.0.

^{1}H NMR (300 Hz, $CDCl_3$): δ 7.79 (d, J = 7.5 Hz, 2H, Fmoc), 7.64 (d, J = 7.5 Hz, 2H, Fmoc), 7.43 (t, J = 7.1 Hz, 2H, Fmoc), 7.34 (d, J = 7.1 Hz, 2H, Fmoc), 7.06 (d, J = 9.2 Hz, 2H, Ph (MP)), 6.83 (d, J = 9.2 Hz, 2H, Ph (MP)), 5.95 (m, 1H, CH$_2$C*H*=CH$_2$), 5.31 (dd, J = 1.5 Hz, J = 17.3 Hz, CH$_2$CH=C*H*H'), 5.20 (dd, J = 1.5 Hz, J = 10.3 Hz, CH$_2$CH=CH*H'*), 4.93 – 4.84 (m, 2H, H-1, H-3), 4.51 (dd, $J_{6A,6B}$ = 11.4 Hz, $J_{6A,5}$ = 6.3 Hz, 1H, H-6A), 4.48 – 4.37 (m, 4H, H-6B, C*H*H'CH=CH$_2$, CH$_2$ (Fmoc)), 4.33 – 4.24 (m, 2H, CH (Fmoc), CH*H'*CH=CH$_2$), 4.19 (br s, 1H, H-4), 3.92 (dd, $J_{2,3}$ = 10.0 Hz, $J_{2,1}$ = 7.7 Hz, 1H, H-2), 3.87 (t, $J_{5,6A}$ = $J_{5,6B}$ = 6.3 Hz, 1H, H-5), 3.75 (s, 3H, OMe (MP)), 2.88 (m, 2H, CH$_2$ (Lev)), 2.63 (m, 2H, CH$_2$ (Lev)), 2.24 (s, 3H, Me (Lev)).

$^{13}C\{^{1}H\}$ NMR (75 Hz, $CDCl_3$): δ 207.9 (C=O (Lev)), 172.1 (C=O (Lev)), 155.5 (*ipso*-Ph (MP)), 155.0 (*ipso*-Fmoc), 151.4 (*ipso*-Ph (MP)), 143.3, 141.3 (Fmoc), 134.8 (CH$_2$*C*H=CH$_2$), 127.9, 127.2, 125.2, 120.1 (Fmoc), 118.7 (Ph (MP)), 117.1 (CH$_2$CH=*C*H$_2$), 114.6 (Ph (MP)), 103.0 (C-1), 75.6 (C-2), 75.3 (C-3), 73.8 (*C*H$_2$CH=CH$_2$), 72.0 (C-5), 70.1 (CH$_2$ (Fmoc)), 66.4 (C-4), 66.2 (C-6), 55.6 (OMe (MP)), 46.8 (CH (Fmoc)), 38.5 (CH$_2$ (Lev)), 29.8 (Me (Lev)), 28.3 (CH$_2$ (Lev)).

HRMS ESI m/z calcd for $[M+Na]^+$ $C_{36}H_{38}O_{11}$ 669.2306; found 669.2301.

p-Methoxyphenyl 2-*O*-allyl-6-*O*-fluorenylmethyloxycarbonyl-3-*O*-levulinyl-4-*O*-pentafluorobenzoyl-β-D-galactopyranoside (11)

To a solution of **10** (2.87 g, 4.44 mmol) in dry CH_2Cl_2 (300 mL) pentafluorobenzoyl chloride (1.25 mL, 8.88 mmol) and pyridine (700 µL, 8.88 mmol) were added under argon atmosphere. The reaction mixture was stirred overnight, then diluted with CH_2Cl_2 and washed with saturated solution of $NaHCO_3$. The aqueous phase was washed with CH_2Cl_2 three times; the combined organic extracts were dried over anhydrous Na_2SO_4, filtered and concentrated under vacuum. The dry residue was purified by column chromatography (silica gel, toluene : EtOAc 20:1 → 3:1) to give colorless oil **11** (3.07 g, 82%). R_f = 0.67 (toluene : EtOAc 2 : 1). $[\alpha]^{23}$ = 2.2.

^1H NMR (300 Hz, $CDCl_3$): δ 7.80 (d, *J* = 7.3 Hz, 2H, Fmoc), 7.64 (dd, *J* = 7.3 Hz, *J* = 4.3 Hz, 2H, Fmoc), 7.45 (t, *J* = 7.1 Hz, 2H, Fmoc), 7.39 – 7.26 (m, 5H, Fmoc + $CHCl_3$), 7.24 – 7.16 (m, 6H, Fmoc + toluene), 7.05 (d, *J* = 9.2 Hz, 2H, Ph (MP)), 6.84 (d, *J* = 9.2 Hz, 2H, Ph (MP)), 5.97 (m, 1H, CH_2CH=CH_2), 5.75 (d, $J_{4,3}$ = 3.1 Hz, 1H, H-4), 5.33 (dd, *J* = 1.6 Hz, *J* = 17.2 Hz, CH_2CH=C*H*H'), 5.23 (dd, *J* = 1.6 Hz, *J* = 10.2 Hz, CH_2CH=CH*H'*), 5.18 (dd, $J_{3,4}$ = 3.1 Hz, $J_{3,2}$ = 10.2 Hz, 1H, H-3), 4.94 (d, $J_{1,2}$ = 7.7 Hz, 1H, H-1), 4.51 – 4.25 (m, 7H, H-6A, H-6B, CH, CH_2 (Fmoc), CH_2CH=CH_2), 4.11 (t, $J_{5,6A}$ = $J_{5,6B}$ = 6.5 Hz, 1H, H-5), 3.87 (dd, $J_{2,3}$ = 10.2 Hz, $J_{2,1}$ = 7.7 Hz, 1H, H-2), 3.77 (s, 3H, OMe (MP)), 2.95 – 2.50 (m, 4H, CH_2 (Lev)), 2.22 (s, 3H, Me (Lev)).

$^{13}C\{^1H\}$ NMR (75 Hz, $CDCl_3$): δ 172.0 (C=O (Lev)), 143.3 (Fmoc), 134.8 (CH_2CH=CH_2), 129.1, 128.2, 128.0, 127.2, 125.3, 125.2, 120.1 (Fmoc), 118.8 (Ph (MP)), 117.4 (CH_2CH=CH_2), 114.6 (Ph (MP)), 103.1 (C-1), 75.7 (C-2), 74.0 (CH_2CH=CH_2), 72.2 (C-3), 70.4 (C-5), 70.3 (CH_2 (Fmoc)), 69.9 (C-4), 64.8 (C-6), 55.6 (OMe (MP)), 46.7 (CH (Fmoc)), 37.8 (CH_2 (Lev)), 29.8 (Me (Lev)), 27.8 (CH_2 (Lev)).

HRMS ESI m/z calcd for $[M+Na]^+$ $C_{43}H_{37}F_5O_{12}$ 863.2097; found 863.2103.

2-*O*-Allyl-6-*O*-fluorenylmethyloxycarbonyl-3-*O*-levulinyl-4-*O*-pentafluorobenzoyl-D-galactopyranose (12)

Compound **11** (3.07 g, 3.65 mmol) was dissolved in acetonitrile (300 mL), then water (75 mL) and benzene (25 mL) were added. CAN (10.0 g, 18.25 mmol) was added to the solution at 0 °C and the mixture was stirred for 10 minutes, diluted with EtOAc and washed with saturated solution of $NaHCO_3$. The aqueous phase was washed with EtOAc three times; the combined organic extracts were dried over anhydrous Na_2SO_4, filtered and concentrated under vacuum. Column chromatography of the residue (silica gel, toluene : EtOAc 30:1 → 3:1) provided yellowish oil **12** (2.36 g, 88%) as a mixture of α- and β-isomers. R_f = 0.28 (toluene : EtOAc 4 : 1).

^1H NMR (300 Hz, $CDCl_3$): δ 7.79 (d, *J* = 7.6 Hz, 2H, Fmoc), 7.62 (dd, *J* = 2.3 Hz, *J* = 7.3 Hz, 2H, Fmoc), 7.43 (t, *J* = 7.3 Hz, 2H, Fmoc), 7.35 (m, 2H, Fmoc), 5.92 (m, 1H, CH_2CH=CH_2), 5.78 (d, $J_{4,3}$ = 2.5 Hz, 0.7H, H-4 α), 5.69 (d, $J_{4,3}$ = 3.1 Hz, 0.3H, H-4 β), 5.43 (m, 1.4H, H-1 α, H-3 α), 5.38 – 5.18 (m, 2H, CH_2CH=CH_2), 5.11 (dd, $J_{3,4}$ = 3.1 Hz, $J_{3,2}$ = 10.4 Hz, 0.3H, H-3 β), 4.82 (d, $J_{1,2}$ = 7.8 Hz, 0.3H, H-1 β), 4.63 (t, $J_{5,6A}$ = $J_{5,6B}$ = 6.5 Hz, 0.7H, H-5 α), 4.51 – 4.37 (m, 2H, CH_2 (Fmoc)), 4.37 – 4.12 (m, 5H, H-6A α, H-6B α, H-6A β, H-6B β, CH (Fmoc), CH_2CH=CH_2), 4.08 (t, $J_{5,6A}$ = $J_{5,6B}$ = 6.5 Hz, 0.3H, H-5 β), 3.86 (dd, $J_{2,3}$ = 10.2 Hz, $J_{2,1}$ = 3.6 Hz, 0.7H, H-2 α), 3.57 (dd, $J_{2,3}$ = 10.4 Hz, $J_{2,1}$ = 7.8 Hz, 0.3H, H-2 β), 3.27 (br s, 0.3H, OH β), 3.04 (br s, 0.7H, OH α), 2.95 – 2.44 (m, 4H, CH_2 (Lev)), 2.21 (s, 3H, Me (Lev)).

$^{13}C\{^1H\}$ NMR (75 Hz, $CDCl_3$): δ 172.2 (C=O (Lev)), 155.4 (*ipso*-Ph (MP)), 143.3, 143.2 (Fmoc), 134.5 (CH_2CH=CH_2 β), 134.1 (CH_2CH=CH_2 α), 129.0, 128.2, 127.9, 127.2, 125.3, 125.2, 120.1 (Fmoc), 118.2 (CH_2CH=CH_2 α), 117.5 (CH_2CH=CH_2 β), 97.5 (C-1 β), 91.9 (C-1 α), 76.9 (C-2 β), 73.9 (CH_2CH=CH_2 β), 73.4 (C-2 α), 72.7 (CH_2CH=CH_2 α), 72.3 (C-3 β), 71.4 (C-4 α), 70.4 (C-4 β), 70.3 (C-5 β, CH_2 (Fmoc) α), 70.2 (CH_2 (Fmoc) β), 69.5 (C-3 α), 66.1 (C-5 α), 65.1 (C-6 α), 65.0 (C-6 β), 46.7 (CH (Fmoc)), 37.8 (CH_2 (Lev)), 29.8 (Me (Lev)), 27.8 (CH_2 (Lev) α), 27.7 (CH_2 (Lev) β).

HRMS ESI m/z calcd for $[M+Na]^+$ $C_{36}H_{31}F_5O_{11}$ 757.1679; found 757.1683.

O-(2-*O*-Allyl-6-*O*-fluorenylmethyloxycarbonyl-3-*O*-levulinyl-4-*O*-pentafluorobenzoyl-D-galactopyranosyl *N*-phenyltrifluoroacetimidate (13)

To a solution of **12** (2.35 g, 3.20 mmol) in acetone (50 mL) *N*-phenyltrifluoroacetimidoyl chloride (616 μL, 3.84 mmol) and K_2CO_3 (663 mg, 4.80 mmol) were added. The reaction mixture was stirred overnight, then diluted with acetone and filtered through a pad of Celite. The filtrate was concentrated under vacuum and the dry residue was purified by column chromatography (Al_2O_3, toluene : EtOAc 1 : 0 → 10 : 1) to give yellowish oil **13** (2.37 g, 82%) as a mixture of α- and β-isomers. R_f = 0.62 (toluene : EtOAc 5 : 1).

^{1}H NMR (300 Hz, $CDCl_3$, 323 K): δ 7.79 (m, 4H, Fmoc), 7.62 (m, 4H, Fmoc), 7.48 – 7.28 (m, 12H, Fmoc, PTFAI), 7.12 (t, J = 7.3 Hz, 2H, PTFAI), 6.87 (t, J = 7.0 Hz, 4H, PTFAI), 6.00 – 5.84 (m, 3H, H-4 α, CH_2CH=CH_2), 5.73 (m, 2H, H-1 β, H-4 β), 5.47 (dd, $J_{3,4}$ = 3.1 Hz, $J_{3,2}$ = 10.4 Hz, 1H, H-3 α), 5.38 – 5.21 (m, 4H, CH_2CH=CH_2), 5.11 (dd, $J_{3,4}$ = 3.0 Hz, $J_{3,2}$ = 9.9 Hz, 1H, H-3 β), 4.53 (m, 1H, H-5 α), 4.50 – 4.37 (m, 4H, CH_2 (Fmoc)), 4.37 – 4.12 (m, 10H, H-6A α, H-6B α, H-6A β, H-6B β, CH (Fmoc), CH_2CH=CH_2), 4.12 – 3.98 (m, 2H, H-2 α, H-5 β), 3.85 (dd, $J_{2,3}$ = 9.9 Hz, $J_{2,1}$ = 8.0 Hz, 1H, H-2 β), 2.94 – 2.47 (m, 8H, CH_2 (Lev)), 2.21 (s, 6H, Me (Lev)).

^{13}C{^{1}H} NMR (75 Hz, $CDCl_3$, 323 K): δ 171.8, 171.7 (C=O (Lev)), 143.3, 141.3 (Fmoc), 134.0 (CH_2CH=CH_2 β), 133.9 (CH_2CH=CH_2 α), 128.8, 127.9, 127.6, 127.2, 125.2, 125.1 (Fmoc), 124.5, 124.4 (PTFAI), 120.0 (Fmoc), 119.3, 119.2 (PTFAI), 117.8 (CH_2CH=CH_2 α), 117.7 (CH_2CH=CH_2 β), 96.1 (C-1 β), 93.4 (C-1 α), 74.8 (C-2 β), 74.1 (CH_2CH=CH_2 β), 72.6 (CH_2CH=CH2 α), 72.3 (C-2 α), 72.1 (C-3 β), 71.1 (C-5 β), 70.5 (CH_2 (Fmoc)), 70.4 (C-4 α), 69.7 (C-4 β), 69.3 (C-3 α), 68.6 (C-5 α), 64.6 (C-6 β), 64.3 (C-6 α), 46.7 (CH (Fmoc)), 37.8, 37.7 (CH_2 (Lev)), 29.6 (Me (Lev)), 27.8, 27.7 (CH_2 (Lev)).

HRMS ESI m/z calcd for $[M+Na]^+$ $C_{44}H_{35}F_8NO_{11}$ 929.1975; found 928.1974.

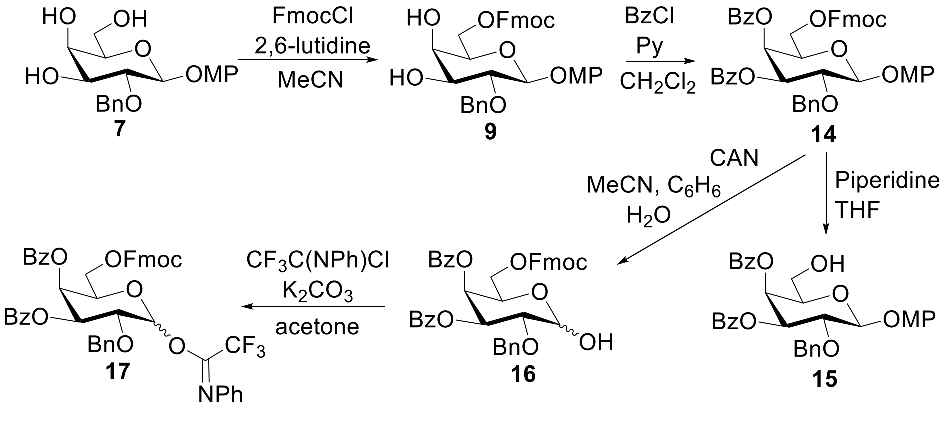

p-Methoxyphenyl 2-*O*-benzyl-6-*O*-fluorenylmethyloxycarbonyl-β-D-galactopyranoside (9)

To a solution of **7**[3] (3.45 g, 9.23 mmol) in dry acetonitrile (70 mL) FmocCl (3.0 g, 1.53 mmoL) and 2,6-lutidine (2.1 mL, 18.45 mmoL) were added and the reaction mixture was stirred for 3 days when TLC (toluene : acetone 1.5 : 1) showed it had completed. The mixture was diluted with toluene and the solvents were removed under vacuum. The dry residue was purified by column chromatography (silica gel, toluene : acetone 50 : 1 → 3 : 1) to give white solid **9** (3.52 g, 64%). R_f = 0.50 ($CHCl_3$: MeOH 15 : 1). $[\alpha]_D^{21}$ = 20.0.

^{1}H NMR (300 Hz, $CDCl_3$): δ 7.80 (d, J = 7.4 Hz, 2H, Fmoc), 7.64 (d, J = 7.4 Hz, 2H, Fmoc), 7.49 – 7.30 (m, 10H, Ph (Bn), Fmoc + $CHCl_3$), 7.07 (d, J = 9.2 Hz, 2H, Ph (MP)), 6.84 (d, J = 9.2 Hz, 2H, Ph (MP)), 5.11 (d, J = 11.5 Hz, 1H, BnA), 4.82 (d, $J_{1,2}$ = 7.4 Hz, 1H, H-1),4.81 (d, J = 11.5 Hz, 1H, BnB), 4.56 – 4.41 (m, 4H, H-6A, H-6B, CH_2 (Fmoc)), 4.28 (t, J = 7.3 Hz, 1H, CH (Fmoc)), 4.00 (br d, 1H, H-

4), 3.86 – 3.77 (m, 2H, H-2, H-5), 3.76 (s, 1H, OMe (MP)), 3.72 (dd, $J_{3,4}$ = 3.3 Hz, $J_{3,2}$ = 9.5 Hz, 1H, H-3).

$^{13}C\{^1H\}$ NMR (75 Hz, $CDCl_3$): δ 155.5 (*ipso*-Ph (MP)), 155.1 (*ipso*-Fmoc), 151.3 (*ipso*-Ph (MP)), 143.3, 143.2, 141.3 (Fmoc), 138.1 (*ipso*-Ph (Bn)), 128.7, 128.3, 128.2, 128.0, 127.2, 125.1 (Ph (Bn), Fmoc), 120.1 (Fmoc), 118.5 (Ph (MP)), 114.6 (Ph (MP)), 102.8 (C-1), 78.5 (C-2), 74.9 (CH_2 (Bn)), 72.8 (C-3), 72.2 (C-5), 70.1 (CH_2 (Fmoc)), 68.0 (C-4), 66.2 (C-6), 55.7 (OMe (MP)), 46.8 (CH (Fmoc)).

HRMS ESI m/z calcd for $[M+Na]^+$ $C_{35}H_{34}O_9$ 621.2095; found 621.2086.

_p_-Methoxyphenyl 3,4-di-_O_-benzoyl-2-_O_-benzyl-6-_O_-fluorenylmethyloxycarbonyl-β-D-galactopyranoside (14)

To a solution of **9** (3.50 g, 5.85 mmol) in dry CH_2Cl_2 (35 mL) benzoyl chloride (1.6 mL, 14.0 mmol) and pyridine (2.3 mL, 28.1 mmol) were added and the reaction mixture was stirred overnight. Then it was diluted with $CHCl_3$ and washed with saturated solution of $NaHCO_3$. The aqueous phase was washed with $CHCl_3$ three times; the combined organic extracts were dried over anhydrous Na_2SO_4, filtered, concentrated under vacuum and purified by column chromatography (silica gel, toluene : EtOAc 1 : 0 → 20 : 1) to give colorless syrup **14** (4.70 g, 99%). R_f = 0.50 (toluene : EtOAc 5 : 1). $[\alpha]_D^{25}$ = 78.1.

^1H NMR (600 Hz, $CDCl_3$): δ 8.02 (dd, J = 8.2 Hz, J= 1.3 Hz, 2H, Ph (Bz)), 7.80 (d, J = 7.4 Hz, 2H, Fmoc), 7.75 (d, J = 7.4 Hz, 2H, Fmoc), 7.60 (m, 3H, Ph (Bz)), 7.47 (m, 3H, Ph (Bz), Fmoc), 7.40 (t, J = 7.5 Hz, 2H, Fmoc), 7.31 (m, 5H, Ph (Bn)), 7.22 (m, 2H, Ph (Bz)), 7.16 (m, 2H, Ph (Bz)), 7.11 (d, J = 9.2 Hz, 2H, Ph (MP)), 6.82 (d, J = 9.2 Hz, 2H, Ph (MP)), 5.84 (d, $J_{4,3}$ = 3.2 Hz, 1H, H-4), 5.47 (dd, $J_{3,4}$ = 3.1 Hz, $J_{3,2}$ = 10.2 Hz, 1H, H-3), 5.11 (d, $J_{1,2}$ = 7.7 Hz, 1H, H-1), 4.96 (d, J = 11.6 Hz, 1H, BnA), 4.81 (d, J = 11.6 Hz, 1H, BnB), 4.43 (dd, $J_{6A,6B}$ = 11.2 Hz, $J_{6A,5}$ = 7.3 Hz, 1H, H-6A), 4.41 – 4.29 (m, 2H, CH_2 (Fmoc)), 4.31 (dd, $J_{6B,6A}$ = 11.2 Hz, $J_{6B,5}$ = 5.0 Hz, 1H, H-6B), 4.25 – 4.20 (m, 2H, H-5, CH (Fmoc)), 4.16 (dd, $J_{2,3}$ = 10.2 Hz, $J_{2,1}$ = 7.7 Hz, 1H, H-2), 3.74 (s, 3H, OMe (MP)).

^1H NMR spectra contains traces of BzCl: δ 8.11 (d, 2H), 7.52 (t, 2H), 7.16 (m, 1H).

$^{13}C\{^1H\}$ NMR (150 Hz, $CDCl_3$): δ 165.6 (C=O (Bz)), 155.9 (*ipso*-Ph (MP)), 154.9 (*ipso*-Fmoc), 151.5 (*ipso*-Ph (MP)), 143.5, 143.4 (Fmoc), 141.5 (*ipso*-Bz), 141.4 (*ipso*-Fmoc), 137.8 (*ipso*-Ph (Bn)), 133.9, 133.7, 133.4, 130.4, 130.2, 129.9, 129.5, 129.3, 128.7, 128.5, 128.4, 128.1, 127.9, 127.4, 125.4, 125.3 (Ph (Bn, Bz), Fmoc), 120.2 (Fmoc), 118.8 (Ph (MP)), 114.9 (Ph (MP)), 103.4 (C-1), 76.2 (C-2), 75.1 (CH_2 (Bn)), 72.9 (C-3), 71.5 (C-5), 70.4 (CH_2 (Fmoc)), 68.5 (C-4), 65.9 (C-6), 55.8 (OMe (MP)), 46.9 (CH (Fmoc)).

HRMS ESI m/z calcd for $[M+Na]^+$ $C_{49}H_{42}O_{11}$ 829.2619; found 829.2624.

_p_-Methoxyphenyl 3,4-di-_O_-benzoyl-2-_O_-benzyl-β-D-galactopyranoside (15)

A solution of **14** (807.0 mg, 1.00 mmol) in dry THF (8 mL) was cooled down to 0 °C and piperidine (1.0 mL, 10.0 mmol) was added. In 15 minutes TLC (toluene : EtOAc 5 : 1) indicated that the reaction had completed. The mixture was diluted with toluene and the solvents were removed under vacuum. The dry residue was purified by column chromatography (silica gel, toluene : EtOAc 30:1 → 6 : 1) to give **15** (406.1 mg, 69%) as a colorless syrup. R_f = 0.25 (toluene : EtOAc 5 : 1). $[\alpha]_D^{20}$ = 87.2.

^1H NMR (300 Hz, $CDCl_3$): δ 8.01 (d, J = 7.4 Hz, 2H, Ph (Bz)), 7.82 (d, J = 7.4 Hz, 2H, Ph (Bz)), 7.63 (t, J = 7.4 Hz, 1H, Ph (Bz)), 7.49 (m, 3H, Ph (Bz)), 7.32 (d, J = 7.4 Hz, 2H, Ph (Bz)), 7.20 (m, 5H, Ph (Bn)), 7.08 (d, J = 9.2 Hz, 2H, Ph (MP)), 6.87 (d, J = 9.2 Hz, 2H, Ph (MP)), 5.72 (d, $J_{4,3}$ = 3.1 Hz, 1H,

H-4), 5.49 (dd, $J_{3,4}$ = 3.1 Hz, $J_{3,2}$ = 10.1 Hz, 1H, H-3), 5.15 (d, $J_{1,2}$ = 7.8 Hz, 1H, H-1), 4.97 (d, J = 11.5 Hz, 1H, BnA), 4.83 (d, J = 11.5 Hz, 1H, BnB), 4.20 (dd, $J_{2,3}$ = 10.1 Hz, $J_{2,1}$ = 7.8 Hz, 1H, H-2), 4.01 (t, $J_{5,6A}$ = $J_{5,6B}$ = 6.5 Hz, 1H, H-5), 3.85 – 3.76 (m, 4H, H-6A, OMe (MP)), 3.63 (dd, $J_{6B,6A}$ =11.7 Hz, $J_{6B,5}$ = 6.5 Hz, 1H, H-6B).

$^{13}C\{^1H\}$ NMR (75 Hz, $CDCl_3$): δ 166.7, 165.4 (C=O (Bz)), 155.6, 151.5 (*ipso*-Ph (MP)), 137.6 (*ipso*-Ph (Bn)), 133.7, 133.3, 130.2, 130.1, 129.8, 129.3, 129.0, 128.6, 128.3, 127.8 (Ph (Bn, Bz)), 118.4 (Ph (MP)), 114.8 (Ph (MP)), 103.2 (C-1), 76.0 (C-2), 74.8 (CH_2 (Bn)), 73.9 (C-5), 72.7 (C-3), 69.2 (C-4), 60.7 (C-6), 55.7 (OMe (MP)).

HRMS ESI m/z calcd for $[M+Na]^+$ $C_{34}H_{32}O_9$ 607.1939; found 607.1937.

3,4-di-*O*-Benzoyl-2-*O*-benzyl-6-*O*-fluorenylmethyloxycarbonyl-D-galactopyranose (16)

Compound **14** (749.0 mg, 0.93 mmol) was dissolved in acetonitrile (75 mL), then water (19 mL) and benzene (6 mL) were added. CAN (2.54 g, 4.64 mmol) was added to the solution at 0 °C and the mixture was stirred for 12 minutes, diluted with EtOAc and washed with saturated solution of $NaHCO_3$. The aqueous phase was washed with EtOAc three times; the combined organic extracts were dried over anhydrous Na_2SO_4, filtered and concentrated under vacuum. Column chromatography of the residue (silica gel, toluene : EtOAc 20:1 → 4:1) provided yellowish oil **16** (478.2 mg, 74%) as a mixture of α- and β-isomers. R_f = 0.35 (toluene : EtOAc 5 : 1).

^1H NMR (300 Hz, $CDCl_3$): δ 7.98 (m, 2H, Ph (Bz))), 7.85 – 7.72 (m, 4H, Ph (Bz)), Fmoc), 7.65 – 7.14 (m, 19H, Ph (Bn, Bz), Fmoc + $CHCl_3$), 5.87 (br d, 0.7H, H-4 α), 5.81 – 5.72 (m, 1H, H-3 α, H-4 β), 5.49 (br t, 0.7H, H-1 α), 5.41 (dd, $J_{3,4}$ = 3.4 Hz, $J_{3,2}$ = 10.4 Hz, 0.3H, H-3 β), 4.97 (dd, $J_{1,2}$ = 7.5 Hz, $J_{1,OH}$ = 5.0 Hz, 0.3H, H-1 β), 4.88 (d, J = 11.7 Hz, 0.3H, BnA β), 4.78 – 4.62 (m, 2.4H, H-5 α, BnA α, BnB α, BnB β), 4.23 – 4.10 (m, 6 H, H-2 α, H-5 β, H-6A α, H-6B α, H-6A β, H-6B β, CH, CH_2 (Fmoc)), 3.88 (dd, $J_{2,3}$ = 10.4 Hz, $J_{2,1}$ = 7.5 Hz, 0.3H, H-2 β), 3.52 (d, $J_{OH,1}$ = 5.0 Hz, 0.3 H, OH β), 3.16 (d, $J_{OH,1}$ = 5.0 Hz, 0.3 H, OH α).

$^{13}C\{^1H\}$ NMR (75 Hz, $CDCl_3$): δ 165.5 (C=O (Bz)), 154.8 (*ipso*-Ph (Bz)), 143.3, 141.3 (Fmoc), 137.3 (*ipso*-Ph (Bn)), 133.5, 133.2, 130.0, 129.9, 129.7, 129.6, 129.4, 128.6, 128.5, 128.3, 128.1, 127.9, 127.2, 127.2, 125.2, 125.2 (Ph (Bn, Bz), Fmoc), 120.1, 120.0 (Fmoc), 97.8 (C-1 β), 91.8 (C-1 α), 77.2 (C-2 β), 74.7 (CH_2 (Bn) β), 73.7 (C-2 α), 73.3 (CH_2 (Bn) α), 72.8 (C-3 β), 71.3 (C-5 β), 70.1 (CH_2 (Fmoc)), 70.1 (C-3 α), 69.6 (C-4 α), 68.6 (C-4 β), 67.1 (C-5 α), 66.1 (C-6 α), 66.0 (C-6 β), 46.7 (CH (Fmoc)),

HRMS ESI m/z calcd for $[M+Na]^+$ $C_{42}H_{36}O_{10}$ 723.2201; found 723.2204.

***O*-(3,4-di-*O*-Benzoyl-2-*O*-benzyl-6-*O*-fluorenylmethyloxycarbonyl-D-galactopyranosyl) *N*-phenyltrifluoroacetimidate (17)**

To a solution of **16** (462.0 mg, 0.66 mmol) in acetone (25 mL) *N*-phenyltrifluoroacetimidoyl chloride (127 μL, 0.792 mmol) and K_2CO_3 (136.6 mg, 0.99 mmol) were added. The reaction mixture was stirred overnight, then diluted with acetone and filtered through a pad of Celite. The filtrate was concentrated under vacuum and the dry residue was purified by column chromatography (Al_2O_3, toluene : EtOAc 1: 0 → 10 : 1) to give yellowish oil **17** (485.9 mg, 84%) as a mixture of α- and β-isomers. R_f = 0.38 (toluene : EtOac 10 : 1).

^1H NMR (300 Hz, $CDCl_3$, 323 K): δ 8.02 – 7.90 (m, 2H, Ph (Bz)), 7.85 – 7.70 (m, 4H, Ph (Bz), Fmoc)), 7.65 – 7.04 (m, 25H, Ph (Bz), PTFAI, Fmoc + $CHCl_3$), 6.91 – 6.75 (m, 2H, PTFAI), 6.68 (br s, 0.4H, H-1 α), 5.87 – 5.76 (m, 1.6H, H-1 β, H-4 β, H-3 α), 5.44 (dd, $J_{3,4}$ = 3.3 Hz, $J_{3,2}$ = 10.0 Hz, 0.6H, H-3 β),

4.82 (d, J = 11.7 Hz, 0.36H, BnA β), 4.74 – 4.66 (m, 1.4H, BnA α, BnB α, BnB β), 4.60 (t, $J_{5,6A}$ = $J_{5,6B}$ = 6.2 Hz, 1H, H-5 α), 4.39 – 4.25 (m, 4.4H, H-2 α, H-6A α, H-6B α, H-6A β, H-6B β, CH_2 (Fmoc)), 4.24 – 4.06 (m, 2.2H, H-2 β, H-5 β, CH (Fmoc)).

$^{13}C\{^1H\}$ NMR (75 Hz, $CDCl_3$, 323 K): δ 143.3 (Fmoc), 133.5, 133.2, 129.9, 129.8, 129.7, 129.7, 128.8, 128.6, 128.4, 128.3, 128.1, 128.0, 127.8, 127.2, 125.2, 125.1 (Ph (Bn, Bz), Fmoc), 124.5, 124.4 (PTFAI), 120.0 (Fmoc), 119.4, 119.3 (PTFAI), 97.3 (C-1 β), 75.3 (C-2 β), 74.1 (CH_2 (Bn) β), 73.3 (CH_2 (Bn) α), 72.7 (C-3 β), 72.6 (C-2 α), 72.1 (C-5 β), 70.3 (CH_2 (Fmoc)), 69.9 (C-3 α), 69.6 (C-5 α), 69.0 (C-4 α), 68.2 (C-4 β), 65.6 (C-6 α), 65.2 (C-6 β), 46.8(CH (Fmoc)).

HRMS ESI m/z calcd for $[M+Na]^+$ $C_{50}H_{40}F_3NO_{10}$ 894.2497; found 894.2488.

III. Regioselective removal of protecting groups of monosaccharide 11

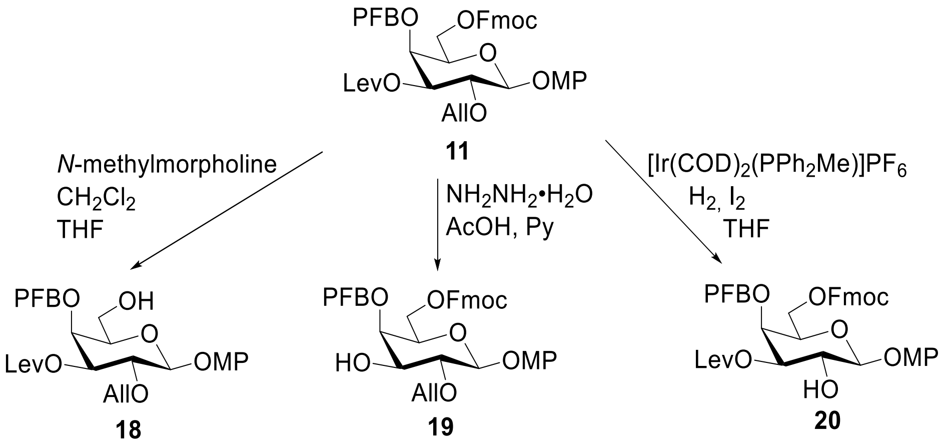

***p*-Methoxyphenyl 2-*O*-allyl-3-*O*-levulinyl-4-*O*-pentafluorobenzoyl-β-D-galactopyranoside (18)**

To a solution of **11** (16.5 mg, 0.02 mmol) in a mixture of dry THF (140 μL) and dry CH_2Cl_2 (100 μL) *N*-methylmorpholine (13 μL, 0.12 mmol) was added at +3 °C and the mixture was slowly warmed up to + 12 °C. After 48 hours TLC (toluene : EtOAc 3 : 1) indicated that the reaction had completed. The base was quenched with 0.1 M aqueous solution of H_2SO_4 (50 μL) and the mixture was washed with brine. The aqueous phase was washed with $CHCl_3$ three times; the combined organic extracts were dried over anhydrous Na_2SO_4, filtered and concentrated under vacuum. Column chromatography of the dry residue (silica gel, toluene : EtOAc 7 : 1 → 3 : 1) gave **18** (8.0 mg, 65%) as a colorless oil. R_f = 0.25 (toluene : EtOAc 3 : 1). $[\alpha]^{25}$ = 16.3.

^1H NMR (300 Hz, $CDCl_3$): δ 7.05 (d, J = 9.2 Hz, 2H, Ph (MP)), 6.85 (d, J = 9.2 Hz, 2H, Ph (MP)), 5.94 (m, 1H, $CH_2CH{=}CH_2$), 5.70 (d, $J_{4,3}$ = 3.3 Hz, 1H, H-4), 5.29 (dd, J = 1.6 Hz, J = 17.2 Hz, 1H, $CH_2CH{=}CHH'$), 5.21 (dd, J = 1.6 Hz, J = 10.2 Hz, 1H, $CH_2CH{=}CHH'$), 5.14 (dd, $J_{3,4}$ = 3.4 Hz, $J_{3,2}$ = 10.1 Hz, 1H, H-3), 4.97 (d, $J_{1,2}$ = 7.7 Hz, 1H, H-1), 4.42 (m, 1H, $CHH'CH{=}CH_2$), 4.27 (m, 1H, $CHH'CH{=}CH_2$), 3.96 – 3.82 (m, 3H, H-2, H-5, H-6A), 3.80 (s, 3H, OMe (MP)), 2.92 – 2.50 (m, 4H, CH_2 (Lev)), 2.20 (s, 3H, Me (Lev)), 2.05 (m, 1H, OH).

$^{13}C\{^1H\}$ NMR (75 Hz, $CDCl_3$): δ 134.8 ($CH_2CH{=}CH_2$), 118.4 (Ph (MP)), 117.3 ($CH_2CH{=}CH_2$), 114.7 (Ph (MP)), 102.7 (C-1), 75.9 (C-2), 73.9 ($CH_2CH{=}CH_2$), 73.3 (C-5), 72.4 (C-3), 70.6 (C-4), 60.6 (C-6), 55.7 (OMe (MP)), 37.7 (CH_2 (Lev)), 29.8 (Me (Lev)), 27.7 (CH_2 (Lev)).

HRMS ESI m/z calcd for $[M+Na]^+$ $C_{28}H_{27}F_5O_{10}$ 641.1417; found 641.1418.

***p*-Methoxyphenyl 2-*O*-allyl-6-*O*-fluorenylmethyloxycarbonyl-4-*O*-pentafluorobenzoyl-β-D-galactopyranoside (19)**

Compound **11** (36.0 mg, 0.043 mmol) was dissolved in a mixture of hydrazine monohydrate (29 μL), pyridine (343 μL) and AcOH (232 μL). The reaction mixture was stirred for 20 minutes, quenched with acetone (1 mL) and then the solvents were removed under vacuum. The dry residue was dissolved in toluene (5 mL) and concentrated under vacuum. The dry residue was purified by column chromatography (silica gel, toluene : EtOAc 25 : 1 → 5 : 1) to afford **19** (30.6 mg, 96%) as a white solid. R_f = 0.61 (toluene : EtOAc 2 : 1). $[\alpha]_D^{26}$ = 20.0.

^{1}H NMR (300 Hz, $CDCl_3$): δ 7.77 (d, *J* = 7.5 Hz, 2H, Fmoc), 7.60 (t, *J* = 6.0 Hz, 2H, Fmoc), 7.41 (t, *J* = 7.5 Hz, 2H, Fmoc), 7.32 (t, *J* = 7.5 Hz, 2H, Fmoc), 7.02 (d, *J* = 9.2 Hz, 2H, Ph (MP)), 6.81 (d, *J* = 9.2 Hz, 2H, Ph (MP)), 5.97 (m, 1H, $CH_2C\mathit{H}=CH_2$), 5.72 (d, $J_{4,3}$ = 3.0 Hz, 1H, H-4), 5.33 (dd, *J* = 1.5 Hz, *J* = 17.3 Hz, 1H, $CH_2CH=C\mathit{H}H'$), 5.23 (dd, *J* = 1.5 Hz, *J* = 10.3 Hz, 1H, $CH_2CH=CH\mathit{H'}$), 4.87 (d, $J_{1,2}$ = 7.6 Hz, 1H, H-1), 4.54 (m, 1H, $C\mathit{H}H'CH=CH_2$), 4.47 – 4.20 (m, 6H, H-6A, H-6B, CH, CH_2 (Fmoc), $CH\mathit{H'}CH=CH_2$), 4.06 (t, $J_{5,6A}$ = $J_{5,6B}$ = 6.5 Hz, 1H, H-5), 3.95 (dd, $J_{3,4}$ = 3.0 Hz, $J_{3,2}$ = 9.8 Hz, 1H, H-3), 3.78 – 3.68 (m, 4H, H-2, OMe (MP)), 2.56 (br s, 1H, OH).

$^{13}C\{^1H\}$ NMR (75 Hz, $CDCl_3$): δ 151.3 (*ipso*-Ph (MP)), 143.3 (Fmoc), 134.3 ($CH_2\mathit{C}H=CH_2$), 128.0, 127.2, 125.2, 125.1, 120.1 (Fmoc), 118.5 (Ph (MP)), 118.2 ($CH_2CH=\mathit{C}H_2$), 114.6 (Ph (MP)), 102.9 (C-1), 78.3 (C-2), 73.9 ($\mathit{C}H_2CH=CH_2$), 71.5 (C-4), 71.1 (C-3), 70.9 (C-5), 70.3 (CH_2 (Fmoc)), 65.1 (C-6), 55.6 (OMe (MP)), 46.7 (CH (Fmoc)).

HRMS ESI m/z calcd for $[M+Na]^+$ $C_{38}H_{31}F_5O_{10}$ 765.1730; found 765.1729.

***p*-Methoxyphenyl 6-*O*-fluorenylmethyloxycarbonyl-3-*O*-levulinyl-4-*O*-pentafluorobenzoyl-β-D-galactopyranoside (20)**

$[Ir(COD)(PPh_2Me)_2]PF_6$ (1.8 mg, 0.0021 mmol) was dissolved in anhydrous THF (410 μL). The resulting red suspension was bubbled through with hydrogen for 5 minutes until it became a light yellow solution. The solution was degassed under Ar and a solution of compound **11** (59.5 mg, 0.071 mmol) in anhydrous THF (410 μL) was added. The reaction mixture was stirred under argon atmosphere for 2 hours. Then, a solution of I_2 (36.1 mg, 0.142 mmol) in THF/H_2O 4/1 (1.3 mL) was added and the mixture was stirred for 1 hour. The excess of I_2 was quenched with 5.0 mL 10% aq. solution of $Na_2S_2O_3$. The mixture was diluted with EtOAc and aqueous phase was washed with EtOAc. Combined organic extracts were dried over anhydrous Na_2SO_4, filtered and the solvent was evaporated under reduced pressure. The dry residue was purified by column chromatography (silica gel, toluene : EtOAc 12 : 1 → 2 : 1) to give product **20** as a colorless oil (51.1 mg, 99%). R_f = 0.20 (petroleum ether : acetone 2 : 1). $[\alpha]_D^{22}$ = -4.7.

^{1}H NMR (400 Hz, $CDCl_3$): δ 7.76 (d, *J* = 7.5 Hz, 2H, Fmoc), 7.58 (t, *J* = 7.2 Hz, Fmoc), 7.40 (t, *J* = 7.5 Hz, 2H, Fmoc), 7.30 (m, 2H, Fmoc), 7.02 (d, *J* = 9.2 Hz, 2H, Ph (MP)), 6.76 (d, *J* = 9.2 Hz, 2H, Ph (MP)), 5.71 (d, $J_{4,3}$ = 3.3 Hz, 1H, H-4), 5.16 (dd, $J_{3,4}$ = 3.3 Hz, $J_{3,2}$ = 10.2 Hz, 1H, H-3), 4.88 (d, $J_{1,2}$ = 7.6 Hz, 1H, H-1), 4.46 – 4.33 (m, 3H, H-6A, CH_2 (Fmoc)), 4.30 (dd, $J_{6B,6A}$ = 11.3 Hz, $J_{6B,5}$ = 6,1 Hz, 1H, H-6B), 4.23 (t, *J* = 7.3 Hz, 1H, CH (Fmoc)), 4.11 (t, $J_{5,6A}$ = $J_{5,6B}$ = 6.1 Hz, 1H, H-5), 4.08 (dd, $J_{2,3}$ = 10.2 Hz, $J_{2,1}$ = 7.6 Hz, 1H, H-2), 3.72 (s, 3H, OMe (MP)), 2.90 – 2.47 (m, 4H, CH_2 (Lev)), 2.16 (s, 3H, Me (Lev)).

$^{13}C\{^1H\}$ NMR (100 Hz, $CDCl_3$): δ 206.8 (C=O (Lev)), 172.1 (C=O (Lev)), 155.7 (*ipso*-Ph (MP)), 143.2, 143.1, 141.1 (Fmoc), 127.9, 127.2, 125.23, 125.1, 120.1 (Fmoc), 118.8 (Ph (MP)), 114.5 (Ph (MP)),

102.5 (C-1), 72.5 (C-3), 70.6 (C-5), 70.3 (CH_2 (Fmoc)), 69.7 (C-4), 69.0 (C-2), 64.7 (C-6), 55.5 (OMe (MP)), 46.7 (CH (Fmoc)), 37.9 (CH_2 (Lev)), 29.7 (Me (Lev)), 27.8 (CH_2 (Lev)).

HRMS ESI m/z calcd for $[M+Na]^+$ $C_{40}H_{33}F_5O_{12}$ 823.1784; found 823.1790.

IV. Synthesis of acceptor 21

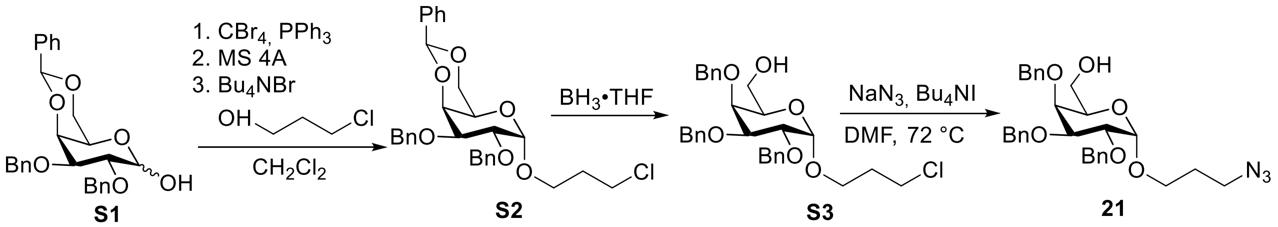

3-Chloropropyl 2,3-di-*O*-benzyl-4,6-*O*-benzylidene-α-D-galactopyranoside (S2)

To a solution of hemiacetal **S1**[4] (468 mg, 1.04 mmol) in anhydrous CH_2Cl_2 (10.0 mL) CBr_4 (1.03 g, 3.12 mmol) and PPh_3 (818 mg, 3.12 mmol) were added under argon atmosphere, the flask was covered with foil and the mixture was stirred for 1 hour. Then MS 4A (1.0 g) were added and the mixture was stirred for another 40 minutes. 3-Chloropropanol (243 μL, 2.91 mmol) and tetrabutylammonium bromide (486 mg, 1.51 mmol) were added. After 1.5 hours the TLC (toluene : EtOAc 2 :1) indicated that the reaction was complete. The reaction mixture was diluted with CH_2Cl_2 and filtered through a pad of Celite, the filtrate was washed with saturated solution of $NaHCO_3$ and the aqueous layer was extracted with CH_2Cl_2 three times. The combined organic extracts were dried over anhydrous Na_2SO_4, filtered and concentrated under vacuum. The dry residue was purified by column chromatography (silica gel, toluene : EtOAc 50 : 1 → 20 : 1) to give only α-isomer **S2** (299 mg, 55%) as a yellowish syrup. R_f = 0.48 (toluene : EtOAc 5 : 1). $[\alpha]_D^{27}$ = 71.0.

^{1}H NMR (400 MHz, $CDCl_3$): δ 7.56 (m, 2H, Ph), 7.47 – 7.26 (m, 13H, Ph), 5.51 (s, 1H, PhC*H*), 4.94 (d, $J_{1,2}$ = 3.3 Hz, 1H, H-1), 4.89 (d, J = 11.8 Hz, 1H, Bn_1A), 4.86 (d, J = 12.3 Hz, 1H, Bn_2A), 4.77 (d, J = 12.3 Hz, 1H, Bn_2B), 4.67 (d, J = 11.8 Hz, 1H, Bn_1B), 4.27 – 4.22 (m, 2H, H-4, H-6A), 4.11 (dd, $J_{2,3}$ = 10.3 Hz, $J_{2,1}$ = 3.2 Hz, 1H, H-2), 4.04 (dd, $J_{6B,6A}$ = 12.4 Hz, $J_{6B,5}$ = 2.0 Hz, 1H, H-6B), 4.00 (dd, $J_{3,4}$ = 3.5 Hz, $J_{3,2}$ = 10.3 Hz, 1H, H-3), 3.88 (m, 1H, OC*H*H'CH_2CH_2N), 3.74 – 3.63 (m, 3H, H-5, OCH_2CH_2C*H_2*N), 3.58 (m, 1H, OCH*H'*CH_2CH_2N), 2.19 – 1.98 (m, 2H, OCH_2C*H_2*CH_2N).

^{13}C{^{1}H} NMR (100 MHz, $CDCl_3$): δ 138.8, 138.7, 137.8 (*ipso*-Ph), 128.9, 128.4, 128.3, 128.1, 127.9, 127.7, 127.6, 127.5, 127.6 (Ph), 101.1 (PhC*H*), 98.4 (C-1), 76.0 (C-3), 75.7 (C-2), 74.7 (C-4), 73.7 (CH_2 (Bn_1)), 69.5 (CH_2 (Bn_2), C-6), 64.4 (O*C*$H_2CH_2CH_2$N), 62.7 (C-5), 41.8 (OCH_2CH_2*C*H_2N), 32.2 (OCH_2*C*H_2CH_2N).

HRMS ESI m/z calcd for $[M+Na]^+$ $C_{30}H_{33}Cl_5O_6$ 547.1858; found 547.1857.

3-Chloropropyl 2,3,4-tri-*O*-benzyl-α-D-galactopyranoside (S3)

The reaction was carried out at 0 °C under argon atmosphere. To a solution of **S2** (280.8 mg, 0.536 mmol) 1M solution of BH_3 in THF (3.2 mL) was added. After 1 hour of stirring the reaction was quenched with MeOH (43 μL) and Et_3N (150 μL) and concentrated in vacuum. The pure product **S3** (240 mg, 85%) was obtained after a column chromatography (toluene : EtOAc 40 : 1 → 5 : 1) as a colorless syrup. R_f = 0.33 (toluene : EtOAc 5 : 1), $[\alpha]_D^{27}$ = 28.0.

^{1}H NMR (500 MHz, $CDCl_3$): δ 7.45 – 7.27 (m, 15H, Ph (Bn)), 4.99 (d, J = 11.6 Hz, 1H, Bn_1A), 4.90 (d, J = 11.8 Hz, 1H, Bn_2A), 4.86 (d, $J_{1,2}$ = 3.6 Hz, 1H, H-1), 4.83 (d, J = 11.8 Hz, 1H, Bn_3A), 4.77 (d, J = 11.8 Hz, 1H, Bn_2B), 4.69 (d, J = 11.8 Hz, 1H, Bn_3B), 4.67 (d, J = 11.6 Hz, 1H, Bn_1B), 4.08 (dd, $J_{2,3}$ = 9.4 Hz, $J_{2,1}$ = 3.6 Hz, 1H, H-2), 3.96 – 3.90 (m, 2H, H-3, H-5), 3.82 (m, 1H, OC*H*H'CH_2CH_2N), 3.77 (m, 1H, H-4), 3.75 – 3.61 (m, 3H, H-6A, OCH_2CH_2C*H_2*N), 3.58 – 3.47 (m, 2H, H-6B, OCH*H'*CH_2CH_2N), 2.06 (m, 2H, OCH_2C*H_2*CH_2N).

$^{13}C\{^{1}H\}$ NMR (100 MHz, $CDCl_3$): δ 138.6, 138.4, 138.0 (*ipso*-Ph (Bn)), 128.5, 128.4, 128.3, 128.2, 128.1, 127.9, 127.8, 127.6, 127.5, 127.4 (Ph (Bn)), 97.7 (C-1), 78.9 (C-3), 76.6 (C-2), 74.8 (C-5), 74.3 (CH_2 (Bn_1)), 73.4 (CH_2 (Bn_2)), 73.3 (CH_2 (Bn_3)), 70.3 (C-4), 64.3 (O*C*$H_2CH_2CH_2$N), 62.3 (C-6), 41.7 (OCH_2CH_2*C*H_2N), 32.1 (OCH_2*C*H_2CH_2N).

HRMS ESI m/z calcd for $[M+NH_4]^+$ $C_{30}H_{35}Cl_5O_6$ 544.2460; found 544.2464.

3-Azidopropyl 2,3,4-tri-*O*-benzyl-α-D-galactopyranoside (21)

To a solution of **S3** (234.5 mg, 0.445 mmol) in anhydrous DMF (2.3 mL) sodium azide (289.3 mg, 4.45 mmol) and tetrabutylammonim iodide (8.1 mg, 0.022 mmol) were added and the reaction mixture was stirred for 24 hours at 72 °C. Then the mixture was cooled down to room temperature, diluted with EtOAc and washed with saturated solution of $NaHCO_3$. The aqueous layer was extracted with EtOAc three times. The combined organic extracts were dried over anhydrous Na_2SO_4 and filtered. The filtrate was concentrated under vacuum and purified on a chromatography column (silica gel, toluene : EtOAc 10 : 1 → 4 : 1) to give **21** (205.5 mg,87%) as a colorless syrup. R_f = 0.33 (toluene : EtOAc 5 : 1), $[\alpha]_D^{25}$ = 49.1.

^{1}H NMR (600 MHz, $CDCl_3$): δ 7.48 – 7.31 (m, 15H, Ph (Bn)), 5.01 (d, J= 11.5 Hz, 1H, Bn_1A), 4.93 (d, J = 11.7 Hz, 1H, Bn_2A), 4.88 (m, 2H, H-1, Bn_3A), 4.80 (d, J = 11.7 Hz, 1H, Bn_2B), 4.71 (d, J = 12.0 Hz, 1H, Bn_3B), 4.68 (d, J = 11.5 Hz, 1H, Bn_1B), 4.11 (dd, $J_{2,3}$ = 10.6 Hz, $J_{2,1}$ = 3.6 Hz, 1H, H-2), 3.98 (dd, $J_{3,4}$ = 2.5 Hz, $J_{3,2}$ = 10.6 Hz, 1H, H-3), 3.95 (m, 1H, H-5), 3.77 (m, 3H, H-4, H-6A, OC*H*H'CH_2CH_2N), 3.52 (m, 2H, H-6B, OCH*H'*CH_2CH_2N), 3.43 (m, 2H, H-6B, OCH_2CH_2C*H_2*N), 1.92 (m, 2H, OCH_2C*H_2*CH_2N).

$^{13}C\{^{1}H\}$ NMR (150 MHz, $CDCl_3$): δ 138.7, 138.6, 138.2 (ipso Ph (Bn)), 129.1, 128.6, 128.5, 128.4, 128.3, 128.0, 127.9, 127.8, 127.6, (Ph (Bn)), 97.9 (C-1), 79.0 (C-3), 76.7 (C-2), 75.0 (C-5), 74.5 (CH_2 (Bn_1)), 73.5 (CH_2 (Bn_2, Bn_3)), 70.6 (C-4), 64.8 (O*C*$H_2CH_2CH_2$N), 62.4 (C-6), 48.4 (OCH_2CH_2*C*H_2N), 28.8 (OCH_2*C*H_2CH_2N).

HRMS ESI m/z calcd for $[M+NH_4]^+$ $C_{30}H_{35}N_3O_6$ 556.2418; found 556.2422.

V. Synthesis of trisaccharides 1 and 2 and tetrasaccharide 3

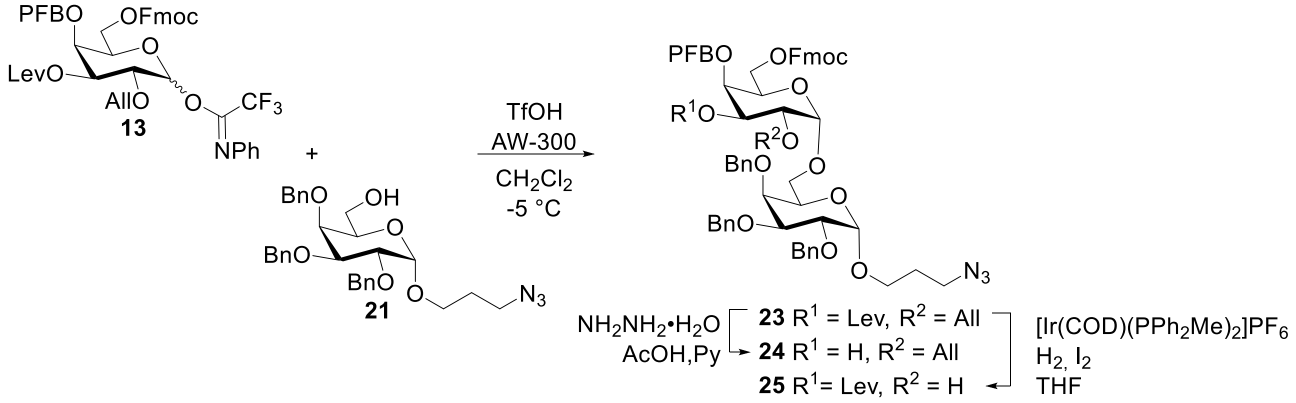

3-Azidopropyl 2-*O*-allyl-6-*O*-fluorenylmethyloxycarbonyl-3-*O*-levulinyl-4-*O*-pentafluorobenzoyl-α-D-galactopyranosyl-(1→6)-2,3,4-tri-*O*-benzyl-α-D-galactopyranoside (23)

Molecular sieves AW-300 (2.60 g) were added to a solution of donor **13** (1.09 g, 1.20 mmol) and acceptor **21** (428 mg, 0.80 mmol) in dry CH_2Cl_2 (15.2 mL) under argon atmosphere at –5 °C. The mixture was stirred for 40 minutes and TfOH (53 µL, 0.60 mmol) was added. In 7 minutes the reaction mixture was diluted with CH_2Cl_2 and filtered through the pad of Celite. The filtrate was washed with saturated solution of $NaHCO_3$ and the aqueous layer was extracted with CH_2Cl_2 three times. The combined organic extracts were dried over anhydrous Na_2SO_4, filtered and concentrated under vacuum. The residue was purified by column chromatography (toluene : EtOAc 30 : 1 → 7 : 1) to give only α-disaccharide **23** (638 mg, 64%). R_f = 0.43 (toluene : EtOAc 4:1). $[\alpha]_D^{23}$ = 63.8.

^{1}H NMR (400 Hz, $CDCl_3$): δ 7.75 (d, J = 7.5 Hz, 2H, Fmoc), 7.57 (t, J = 7.0 Hz, 2H, Fmoc), 7.42 – 7.20 (m, 22H, Ph (Bn), Fmoc + $CHCl_3$), 5.81 (m, 1H, CH_2CH=CH_2), 5.73 (d, $J_{4,3}$ = 3.3 Hz, 1H, H-4^2), 5.38 (dd, $J_{3,4}$ = 3.3 Hz, $J_{3,2}$ = 10.5 Hz, 1H, H-3^2), 5.22 (dd, J =1.7 Hz, J = 17.2 Hz, 1H, CH_2CH=C*H*H'), 5.12 (d, J = 10.3 Hz, 1H, CH_2CH=CH*H'*), 4.96 (d, J = 11.5 Hz, 1H, Bn_1A), 4.90 – 4.84 (m, 2H, H-1^2, Bn_2A), 4.83 – 4.73 (m, 3H, H-1^1, Bn_2B, Bn_3A), 4.66 – 4.56 (m, 2H, Bn_1B, Bn_3B), 4.45 – 4.35 (m, 2H, H-5^2, CH_2 (Fmoc)), 4.33 – 4.16 (m, 4H, H-6A^2, H-6B^2, CH_2, CH(Fmoc)), 4.12 (dd, J = 12.9 Hz, J = 5.3 Hz, 1H, C*H*H'-CH=CH_2), 4.02 (m, 2H, H-2, CH*H'*-CH=CH_2), 3.98 – 3.91 (m, 3H, H-3^1, H-4^1, H-5^1), 3.84 – 3.70 (m, 3H, H-2^2, H-6A^1, OC*H*H'$CH_2CH_2N_3$), 3.51 – 3.35 (m, 4H, H-6B^1, OCH*H'*$CH_2CH_2N_3$, $OCH_2CH_2CH_2N_3$), 2.88 – 2.77 (m, 1H, CH_2 (Lev)), 2.75 – 2.57 (m, 2H, CH_2(Lev)), 2.52 – 2.41 (m, 1H, CH_2 (Lev)), 2.17 (s, 3H, Me (Lev)), 1.89 (m, 2H, $OCH_2CH_2CH_2N_3$).

^{13}C{^{1}H} NMR (100 Hz, $CDCl_3$): δ 134.5 (CH_2CH=CH_2), 128.3, 128.1, 127.8, 127.7, 127.6, 127.2, 125.2, 125.1 (Ph (Bn)), 120.0 (Fmoc), 117.7 (CH_2CH=CH_2), 97.7 (C-1^1, C-1^2), 78.9 (C-3^1), 76.6 (C-2^1), 75.1 (C-4^1), 74.6 (CH_2 (Bn_1)), 73.4 (CH_2 (Bn_2)), 73.3 (CH_2 (Bn_3)), 73.1 (H-2^2), 72.8 (CH_2CH=CH_2), 71.1 (C-4^2), 70.3 (CH_2 (Fmoc)), 69.7 (C-3^2), 69.3 (C-5^1), 67.4 (C-6^1), 65.7 (C-5^2), 65.0 ($OCH_2CH_2CH_2N_3$), 64.7 (C-6^2), 48.4 ($OCH_2CH_2CH_2N_3$), 46.6 (CH (Fmoc)), 37.8 (CH_2 (Lev)), 29.7 (Me (Lev)), 28.8 ($OCH_2CH_2CH_2N_3$), 27.8 (CH_2 (Lev)).

HRMS ESI m/z calcd for $[M+Na]^+$ $C_{51}H_{54}F_5N_3O_{17}$ 1272.4099; found 1272.4084.

3-Azidopropyl 2-*O*-allyl-6-*O*-fluorenylmethyloxycarbonyl-4-*O*-pentafluorobenzoyl-α-D-galactopyranosyl-(1→6)-2,3,4-tri-*O*-benzyl-α-D-galactopyranoside (24)

Disaccharide **23** (242.0 mg, 0.19 mmol) was dissolved in a mixture of hydrazine monohydrate (240 µL), pyridine (2.85 mL) and AcOH (1.90 mL). The reaction mixture was stirred for 11 minutes, quenched with acetone (5 mL) and then the solvents were removed in vacuum. The dry residue was dissolved in toluene (5 mL) and concentrated under vacuum several times. Column chromatography of the residue (silica gel, toluene : EtOAc 30 : 1 → 6 : 1) provided **24** (204.5 g, 92 %) as a colorless oil. R_f = 0.38 (toluene : EtOAc 4 : 1). $[\alpha]_D^{25}$ = 29.7.

^{1}H-NMR (300 MHz, $CDCl_3$): δ 7.75 (d, J = 7.5 Hz, 2H, Fmoc), 7.58 (t, J = 6.5 Hz, 2H, Fmoc), 7.44 – 7.21 (m, 21 H, Ph (Bn), Fmoc), 5.84 (m, 1H, CH_2CH=CH_2), 5.72 (d, $J_{4,3}$ = 3.3 Hz, 1H, H-4^2), 5.25 (dd, J = 1.4 Hz, J = 17.2 Hz, 1H, CH_2CH=C*H*H'), 5.19 (m, 1H, CH_2CH=CH*H'*), 4.99 (d, J = 11.6 Hz, 1H, Bn_1A), 4.92 (d, $J_{1,2}$ = 3.3Hz, 1H, H-1^2), 4.87 (d, J = 11.8 Hz, 1H, Bn_2A), 4.83 – 4.71 (m, 3H, H-1^1, Bn_3A, Bn_3B), 4.66 – 4.56 (m, 2H, Bn_1B, Bn_2B), 4.42 (dd, J = 7.3 Hz, J = 10.4 Hz, 1H, CH_2 (Fmoc)), 4.38 – 4.27 (m, 3H, H-4^1, H-6A^2, CH_2 (Fmoc)), 4.27 – 4.17 (m, 3H, H-3^2, H-6B^2, CH (Fmoc)), 4.08 (m, 2H, CH_2-CH=CH_2), 4.03 (m, 1H, H-2^1), 3.99 – 3.88 (m, 3H, H-3^1, H-5^1, H-5^2), 3.78 (m, 1H, H-6A^1), 3.74 – 3.63 (m, 2H, H-2^1, OC*H*H'$CH_2CH_2N_3$), 3.50 – 3.41 (m, 2H, H-6B^1, OCH*H'*$CH_2CH_2N_3$), 3.38 (t, J = 6.6 Hz, 2H, $OCH_2CH_2CH_2N_3$), 1.87 (m, 2H, $OCH_2CH_2CH_2N_3$).

$^{13}C\{^1H\}$ NMR (75 MHz,$CDCl_3$): δ 143.5, 143.1 (Fmoc), 138.8, 138.6 (*ipso*-Ph (Bn)), 134.2 (CH_2CH=CH_2), 128.4, 128.4, 128.3, 128.0, 127.9, 127.7, 127.6, 127.5, 127.2 (Ph (Bn), Fmoc), 125.3, 125.1 (Fmoc), 120.0 (Fmoc), 118.2 (CH_2CH=CH_2), 97.8 (C-1^1), 97.0 (C-1^2), 78.9 (C-3^1), 76.6 (C-2^1), 76.2 (C-2^2), 75.2 (C-4^1), 74.6 (CH_2 (Bn_1)), 73.5 (CH_2 (Bn_2)), 73.4 (CH_2 (Bn_3)), 72.2 (C-4^2), 72.2 (CH_2CH=CH_2), 70.2 (CH_2 (Fmoc)), 69.3 (C-5^1), 67.7 (C-3^2), 67.5 (C-6^1), 66.2 (C-5^2), 65.1 (C-6^{2}'), 64.9 ($OCH_2CH_2CH_2N_3$), 48.4 ($OCH_2CH_2CH_2N_3$), 46.8 (CH (Fmoc)), 28.8 ($OCH_2CH_2CH_2N_3$).

HRMS ESI m/z calcd for $[M+Na]^+$ $C_{61}H_{58}F_5N_3O_{23}$ 1174.3731; found 1174.3722.

3-Azidopropyl 6-*O*-fluorenylmethyloxycarbonyl-3-*O*-levulinyl-4-*O*-pentafluorobenzoyl-α-D-galactopyranosyl-(1→6)-2,3,4-tri-*O*-benzyl-α-D-galactopyranoside (25)

$[Ir(COD)(PPh_2Me)_2]PF_6$ (5.0 mg, 0.006 mmol) was dissolved in anhydrous THF (2.2 mL). The resulting red suspension was bubbled through with hydrogen for 5 minutes until it became a light yellow solution. The solution was degassed under Ar and a solution of disaccharide **23** (402.4 mg, 0.322 mmol) in anhydrous THF (2.2 mL) was added. The reaction mixture was stirred under Ar for 2 hours. Then, a solution of I_2 (165.1 mg, 0.650 mmol) in THF/H_2O 4/1 (6.0 mL) was added and the mixture was stirred for 1 hour. The excess of I_2 was quenched with 20 mL 10% aq. solution of $Na_2S_2O_3$. The mixture was diluted with EtOAc and aqueous phase was washed with EtOAc. Combined organic extracts were dried over anhydrous Na_2SO_4, filtered and the solvent was evaporated under reduced pressure. The dry residue was purified by column chromatography (silica gel, toluene:EtOAc 12 : 1 → 2 : 1) to give disaccharide **25** as a colorless oil (389.5 mg, 83%). R_f = 0.20 (toluene : EtOAc 5 : 1). $[\alpha]_D^{25}$ = 39.2.

^{1}H NMR (400 Hz, $CDCl_3$): δ 7.76 (d, J = 7.2 Hz, 2H, Fmoc), 7.57 (m, 2H, Fmoc), 7.45 – 7.26 (m, 19H, Ph (Bn), Fmoc), 5.73 (d, $J_{4,3}$ = 3.2 Hz, 1H, H-4^2), 5.22 (dd, $J_{3,4}$ = 3.2 Hz, $J_{3,2}$ = 10.4 Hz, 1H, H-3^2), 4.95 (d, J = 11.7 Hz, 1H, Bn_1A), 4.89 (d, J = 11.7 Hz, 1H, Bn_2A), 4.85 – 4.79 (m, 3H, H-1^1, H-1^2, Bn_3A), 4.75 (d, J = 11.7 Hz, 1H, Bn_2B), 4.64 (d, J = 11.7 Hz, 1H, Bn_3B), 4.59 (d, J = 11.7 Hz, 1H, Bn_1B), 4.44 – 4.16 (m, 6H, H-5^2, H-6A^2, H-6B^2, CH, CH_2 (Fmoc)), 4.04 (dd, $J_{2,3}$ = 10.0 Hz, $J_{2,1}$ = 3.6 Hz, 1H, H-2^1), 4.00 – 3.71 (m, 6H, H-2^2, H-3^1, H-4^1, H-5^1, H-6A^1, OC*H*H'$CH_2CH_2N_3$), 3.48 (m, 1H, OCH*H'*$CH_2CH_2N_3$), 3.40 (t, J = 6.7 Hz, 2H, $OCH_2CH_2CH_2N_3$), 3.31 (dd, $J_{6B,6A}$ = 9.7 Hz, $J_{6B,5}$ = 3.9 Hz, 1H, H-6B^1), 2.88 – 2.45 (m, 4H, CH_2 (Lev)), 2.17 (s, 3H, Me (Lev)), 1.09 (m, 2H, $OCH_2CH_2CH_2N_3$).

$^{13}C\{^1H\}$ NMR (100 Hz, $CDCl_3$): δ 128.5, 128.4, 127.9, 127.8, 127.7, 127.6, 127.2, 125.3, 125.2 (Ph (Bn), Fmoc)), 120.1 (Fmoc), 98.4 (C-1^2), 97.8 (C-1^1), 79.0 (C-3^1), 76.6 (C-2^1), 74.9 (C-4^1), 74.5 (CH_2 (Bn_1)), 73.6 (CH_2 (Bn_2)), 73.5 (CH_2 (Bn_3)), 70.7 (C-4^2), 70.5 (C-3^2), 70.3 (CH_2 (Fmoc)), 69.2 (C-5^1), 67.9 (C-6^1), 67.0 (C-2^2), 66.1 (C-5^2), 65.0 (O*C*$H_2CH_2CH_2N_3$), 64.6 (C-6^2), 48.5 (OCH_2CH_2*C*H_2N_3), 46.6 (CH (Fmoc)), 38.0 (CH_2 (Lev)), 29.7 (Me (Lev)), 28.8 (OCH_2*C*$H_2CH_2N_3$), 27.9 (CH_2 (Lev)).

HRMS ESI m/z calcd for $[M+Na]^+$ $C_{63}H_{86}F_5N_3O_{16}$ 1232.3786; found 1232.3784.

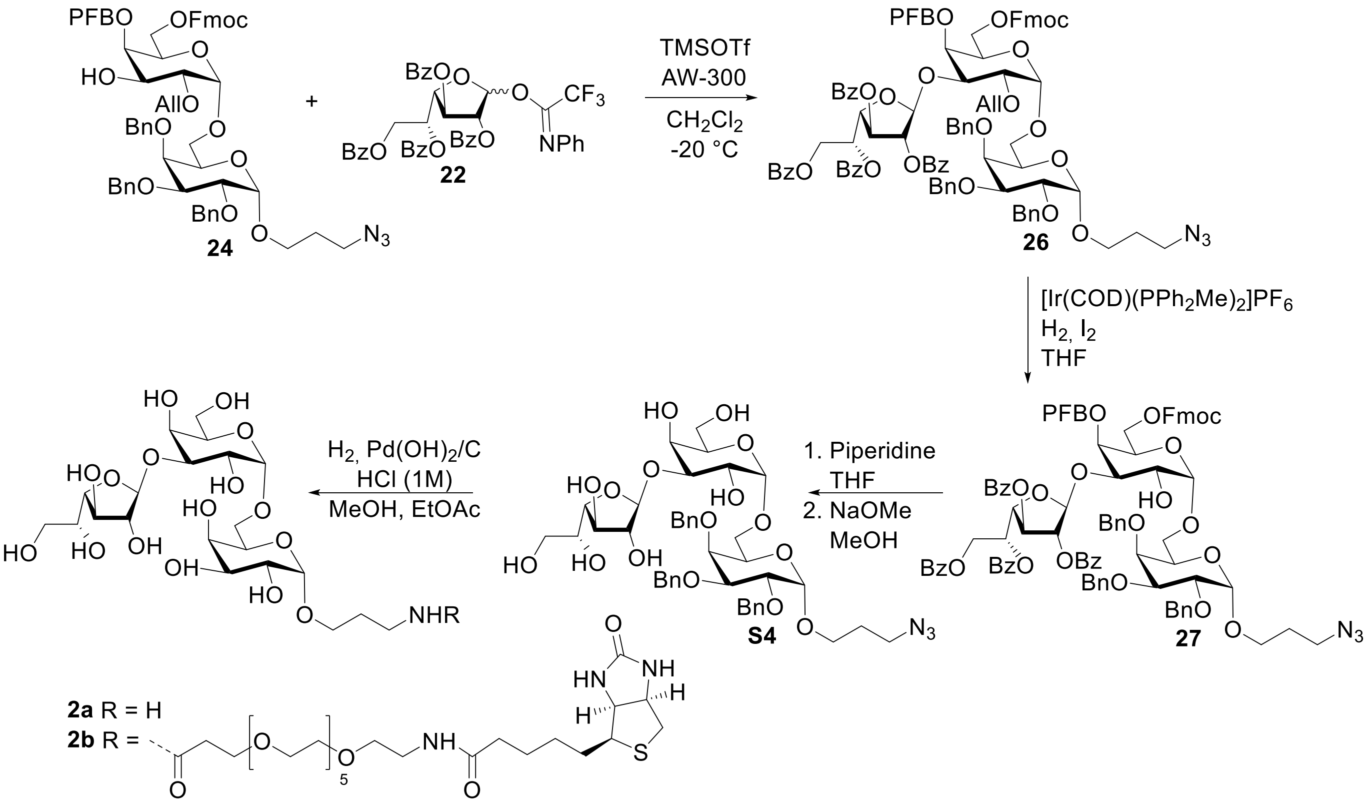

3-Azidopropyl 2,3,4,6-tetra-*O*-benzoyl-β-D-galactofuranosyl-(1→3)-2-*O*-allyl-6-*O*-fluorenylmethyloxycarbonyl-4-*O*-pentafluorobenzoyl-α-D-galactopyranosyl-(1→6)-2,3,4-tri-*O*-benzyl-α-D-galactopyranoside (26)

Molecular sieves AW-300 (370 mg) were added to a solution of donor **22** (184.4 mg, 0.240 mmol) and acceptor **24** (184.5 mg, 0.160 mmol) in dry CH_2Cl_2 (3.7 mL) under argon atmosphere at –20 °C. The mixture was stirred for 40 minutes and TMSOTf (12 μL, 0.064 mmol) was added. In 1 hour the mixture was slowly warmed up. In the next 30 minutes the temperature was –12 °C, the reaction mixture was diluted with CH_2Cl_2 and filtered through the pad of Celite. The filtrate was washed with saturated solution of $NaHCO_3$ and the aqueous layer was extracted with CH_2Cl_2 three times. The combined organic extracts were dried over anhydrous Na_2SO_4, filtered and concentrated under vacuum. The residue was purified by column chromatography (toluene : EtOAc 50 : 1→20 : 1) to give trisaccharide **26** (237.2 mg, 86%) as a β-isomer. R_f = 0.20 (toluene : EtOAc 25 : 1). $[\alpha]_D^{25}$ = 33.8.

^{1}H-NMR (600 MHz, $CDCl_3$): δ 8.08 (d, J = 8.1 Hz, 2H, Ph (Bz)), 8.01 (d, J = 8.1 Hz, 2H, Ph (Bz)), 7.86 (d, J = 8.1 Hz, 2H, Ph (Bz)), 7.83 (d, J = 8.1 Hz, 2H, Ph (Bz)), 7.70 (d, J = 7.5 Hz, 2H, Fmoc), 7.57 (d, J = 7.5 Hz, 1H, Fmoc), 7.54 (d, J = 7.5 Hz, 1H, Fmoc), 7.52 – 7.46 (m, 3H, Ph (Bz), Fmoc), 7.44 (t, J = 7.5 Hz, 1H, Fmoc), 7.39 – 7.26 (m, 21H, Ph (Bn, Bz)), 7.25 – 7.14 (m, 6H, Ph (Bn, Bz)) + toluene), 6.17 (m, 1H, H-5^{II}), 5.84 (m, 1H, CH_2*C*H=CH_2), 5.71 (d, $J_{4,3}$ = 3.2 Hz, 1H, H-4^2), 5.61 (s, 1H,

H-1^{II}), 5.52 (s, 1H, H-2^{II}), 5.40 (d, $J_{3,4}$ = 5.3 Hz, 1H, H-3^{II}), 5.23 (dd, J = 17.2 Hz, J = 1.1 Hz, 1H, $CH_2CH{=}C\mathit{H}H'$), 5.08 (dd, J = 1.1 Hz, J =10.6 Hz, 1H, $CH_2CH{=}CH\mathit{H'}$), 5.04 (dd, $J_{4,5}$ = 5.3 Hz, $J_{4,3}$ = 3.1 Hz, 1H, H-4^{II}), 4.98 (d, J = 11.6 Hz, 1H, Bn_1A), 4.86 (m, 2H, H-1^{2}, Bn_2A), 4.80 – 4.72 (m, 5H, H-1^{1}, Bn_2B, Bn_3A, H-6A^{II}, H-6B^{II}), 4.63 (d, J = 11.9 Hz, 1H, Bn_3B), 4.60 (d, J = 11.6 Hz, 1H, Bn_1B), 4.39 (dd, J = 7.3 Hz, J = 10.4 Hz, 1H, CH_2 (Fmoc)), 4.33 – 4.25 (m, 4H, H-3^{2}, H-5^{2}, CH_2 (Fmoc), $C\mathit{H}H'$-$CH{=}CH_2$), 4.18 (m, 1H, CH (Fmoc)), 4.16 – 4.05 (m, 3H, H-6A^{2}, H-6B^{2}, $CH\mathit{H'}$-$CH{=}CH_2$), 4.03 (dd, $J_{2,3}$ = 9.5 Hz, $J_{2,1}$ = 3.5 Hz, 1H, H-2^{1}), 3.99 – 3.93 (m, 3H, H-3^{1}, H-4^{1}, H-5^{1}), 3.81 (dd, $J_{2,3}$ = 10.1 Hz, $J_{2,1}$ = 3.5 Hz, 1H, H-2^{2}), 3.78 (dd, $J_{6A,6B}$=9.6 Hz, $J_{6A,5}$=6.2 Hz, 1H, H-6A^{1}), 3.73 (m, 1H, $OC\mathit{H}H'CH_2CH_2N_3$), 3.50 (dd, $J_{6B,6A}$ = 9.6 Hz, $J_{6B,5}$ = 6.8 Hz, 1H, H-6B^{1}), 3.50 (m, 1H, $OCH\mathit{H'}CH_2CH_2N_3$), 3.39 (t, J= 6.5 Hz, 2H, $OCH_2CH_2C\mathit{H}_2N_3$), 1.87 (m, 2H, $OCH_2C\mathit{H}_2CH_2N_3$).

$^{13}C\{^1H\}$ NMR (150.9 MHz, $CDCl_3$): δ 166.1, 165.8, 165.7, 165.1 (C=O (Bz)), 154.7, 143.4, 143.1, 141.2, 141.2 (*ipso*-Ph (Bz), Fmoc), 138.7, 138.6, (*ipso*-Ph (Bn)), 134.6 ($CH_2\mathit{C}H{=}CH_2$), 133.3, 133.2, 133.1, 132.9, 130.0, 129.8, 129.7, 129.5, 128.4, 128.3, 128.3, 127.9, 127.9, 127.8, 127.8, 127.6, 127.5, 127.4, 127.2, 127.1, 125.2, 125.1 (Ph (Bn, Bz), Fmoc), 120.0 (Fmoc), 117.6 ($CH_2CH{=}\mathit{C}H_2$), 107.5 (C-1^{II}), 98.0 (C-1^{2}), 97.8 (C-1^{1}), 81.8 (C-2^{II}), 81.5 (C-4^{II}), 78.9 (C-3^{1}), 78.7 (C-3^{II}), 76.6 (C-2^{1}), 75.3 (C-2^{2}), 75.1 (C-4^{1}), 74.6 (CH_2 (Bn_1)), 73.4 (C-3^{2}), 73.3 (CH_2 (Bn_2)), 73.2 ($\mathit{C}H_2CH{=}CH_2$), 73.1 (CH_2 (Bn_3)), 73.0 (C-4^{2}), 70.4 (C-5^{II}), 70.2 (CH_2 (Fmoc)), 69.1 (C-5^{1}), 67.2 (C-6^{1}), 66.3 (C-5^{2}), 65.3 (C-6^{2}), 64.8 ($O\mathit{C}H_2CH_2CH_2N_3$), 63.4 (C-6^{II}), 48.4 ($OCH_2CH_2\mathit{C}H_2N_3$), 46.8 (CH (Fmoc)), 28.8 ($OCH_2\mathit{C}H_2CH_2N_3$).

HRMS ESI m/z calcd for $[M+Na]^+$ $C_{95}H_{84}F_5N_3O_{23}$ 1752.5308; found 1752.5306.

3-Azidopropyl 2,3,4,6-tetra-*O*-benzoyl-β-D-galactofuranosyl-(1→3)-6-*O*-fluorenylmethyloxycarbonyl-4-*O*-pentafluorobenzoyl-α-D-galactopyranosyl-(1→6)-2,3,4-tri-*O*-benzyl-α-D-galactopyranoside (27)

$[Ir(COD)(PPh_2Me)_2]PF_6$ (3.7 mg, 0.0043 mmol) was dissolved in anhydrous THF (1.5 mL). The resulting red suspension was bubbled through with hydrogen for 8 minutes until it became a light yellow solution. The solution was degassed under Ar and a solution of trisaccharide **26** (142.3 mg, 0.082 mmol) in anhydrous THF (1.0 mL) was added. The reaction mixture was stirred under Ar for 2 hours. Then, a solution of I_2 (44.1 mg, 0.174 mmol) in THF/H_2O 4/1 (1.6 mL) was added and the mixture was stirred for 1 hour. The excess of I_2 was quenched with 10 mL 10% aq. solution of $Na_2S_2O_3$. The mixture was diluted with EtOAc and aqueous phase was washed with EtOAc. Combined organic extracts were dried over anhydrous Na_2SO_4, filtered and the solvent was evaporated under reduced pressure. The dry residue was purified by column chromatography (silica gel, toluene : EtOAc 12 : 1 → 2:1) to give trisaccharide **27** as a colorless oil (109.0 mg, 78 %). R_f = 0.47 (toluene : EtOAc 5 : 1). $[\alpha]_D^{20}$ = 42.2.

^1H-NMR (300 MHz, $CDCl_3$): δ 8.08 (d, J = 7.1 Hz, 2H, Ph (Bz)), 7.99 (d, J = 7.5 Hz, 2H, Ph (Bz)), 7.88 (t, J = 7.1 Hz, 4H, Ph (Bz)), 7.70 (d, J = 7.5 Hz, 2H, Fmoc), 7.61 – 7.42 (m, 6H, Ph (Bz), Fmoc), 7.41 – 7.21 (m, 32H, Ph (Bn, Bz), Fmoc + $CHCl_3$), 6.07 (m, 1H, H-5^{II}), 5.77 (br d, 1H, H-4^{2}), 5.60 (s, 1H, H-1^{II}), 5.55 (dd, $J_{3,4}$ = 5.3 Hz, $J_{3,2}$ = 1.6 Hz, 1H, H-3^{II}), 5.40 (d, $J_{2,3}$ = 1.6 Hz, 1H, H-2^{II}), 5.02 – 4.92 (m, 2H, H-4^{II}, Bn_1A), 4.86 (m, 2H, H-1^{2}, Bn_2A), 4.84 – 4.71 (m, 5H, H-1^{1}, H-6A^{II}, H-6B^{II}, Bn_2B, Bn_3A), 4.67 – 4.57 (m, 2H, Bn_1B, Bn_3B), 4.41 (dd, J = 7.3 Hz, J = 10.2 Hz, 1H, $C\mathit{H}H'$ (Fmoc)), 4.32 (m, 2H, H-5^{2}, $CH\mathit{H'}$ (Fmoc)), 4.24 – 4.14 (m, 3H, H-6A^{2}, H-6B^{2}, CH (Fmoc)), 4.14 – 3.99 (m, 3H, H-2^{1}, H-2^{2}, H-3^{2}), 3.98 – 3.87 (m, 3H, H-3^{1}, H-4^{1}, H-5^{1}), 3.81 (dd, $J_{6A,6B}$ = 10.0 Hz, $J_{6A,5}$ = 6.6 Hz, 1H, H-6A^{1}), 3.72 (m, 1H, $OC\mathit{H}H'CH_2CH_2N_3$), 3.43 (m, 2H, H-6B^{1}, $OCH\mathit{H'}CH_2CH_2N_3$), 3.36 (t, J = 6.6 Hz, 2H, $OCH_2CH_2C\mathit{H}_2N_3$), 2.78 (d, $J_{OH,2}$ = 5.8 Hz, 1H, OH), 1.86 (m, 2H, $OCH_2C\mathit{H}_2CH_2N_3$).

$^{13}C\{^1H\}$ NMR (75 MHz, $CDCl_3$): δ 165.7 (C=O (Bz)), 154.7 (*ipso*-Ph (Bz)), 143.5, 143.2, 141.2, (*ipso*-Ph (Bz), Fmoc), 138.8, 138.6, 138.4 (*ipso*-Ph (Bn)), 133.6, 133.3, 133.0 (*ipso*-Ph (Bz)), 130.0, 129.9, 129.8, 129.6, 128.9, 128.7, 128.5, 128.4, 128.3, 128.3, 128.2, 128.1, 127.9, 127.7, 127.7, 127.6, 127.5, 127.2, 125.3, 125.1 (Ph (Bn, Bz), Fmoc), 120.0 (Fmoc), 107.9 (C-1^{II}), 98.5 (C-1^2), 97.8 (C-1^1), 83.5 (C-2^{II}), 81.1 (C-4^{II}), 79.0 (C-3^1), 77.7 (C-3^{II}), 76.6 (C-2^1), 75.5 (C-3^2), 75.1 (C-4^1), 74.6 (CH_2 (Bn_1)), 73.5 (CH_2 (Bn_2, Bn_3), 72.4 (C-4^2), 70.3 (C-5^{II}), 70.2 (CH_2 (Fmoc)), 69.1 (C-5^1), 68.8 (C-2^2), 67.7 (C-6^1), 66.5 (C-5^2), 65.2 (C-6^2), 64.9 (O*C*$H_2CH_2CH_2N_3$), 63.4 (C-6^{II}), 48.4 (OCH_2CH_2*C*H_2N_3), 46.7 (CH (Fmoc)), 28.9 (OCH_2*C*$H_2CH_2N_3$).

HRMS ESI m/z calcd for $[M+Na]^+$ $C_{92}H_{80}F_5N_3O_{23}$ 1712.4995; found 1712.5010.

3-Azidopropyl β-D-galactofuranosyl-(1→3)-α-D-galactopyranosyl-(1→6)-2,3,4-tri-*O*-benzyl-α-D-galactopyranoside (S4)

Piperidine (83 μL, 0.852 mmol) was added to a solution of trisaccharide **27** (144.0 mg, 0.085 mmol) in anhydroud THF (1.4 mL) at 0 °C. In 40 minutes, the reaction was completed, the mixture was diluted with toluene, and the solvents were evaporated under vacuum. The dry residue was dissolved in anhydrous MeOH (1.5 mL), 1M solution of NaOMe in MeOH (13 μL) was added and the mixture was stirred overnight. Then NaOMe was quenched with AcOH (2.0 μL) and the solvent was evaporated under vacuum. The dry residue purified by column chromatography (silica gel, $CHCl_3$: MeOH 20 : 1 → 5 : 1) to give trisaccharide **S4** as a white powder (58.3 mg, 80%). R_f = 0.17 ($CHCl_3$: MeOH 8 : 1). $[\alpha]_D^{20}$ = 15.7.

^{1}H-NMR (300 MHz, $CDCl_3$ + CD_3OD): δ 7.43 – 7.22 (m, 15H, Ph (Bn)), 5.18 (s, 1H, H-1^{II}), 4.92 (d, J = 11.3 Hz, 1H, Bn_1A), 4.83 – 4.70 (m, 5H, H-1, H-1^2, Bn_2A, Bn_3A, Bn_3B), 4.71 – 3.59 (m, 2H, Bn_1B, Bn_2B), 4.11 (m, 1H, H-4^1), 4.10 – 4.00 (m, 4H, H-2^{II}, H-3^{II}, H-4^{II}, H-4^2), 4.00 – 3.92 (m, 3H, H-2^1, H-3^1, H-4^1), 3.89 (dd, $J_{2,3}$ = 10.0 Hz, $J_{2,1}$ = 3.7 Hz, 1H, H-2^2), 3.85 – 3.72 (m, 5H, H-3^2, H-5^2, H-5^{II}, H-$6A^1$, OC*H*H'$CH_2CH_2N_3$), 3.70 (m, 2H, H-$6A^2$, H-$6B^2$), 3.62 (m, 2H, H-$6A^{II}$, H-$6B^{II}$), 3.51 (m, 1H, H-$6B^1$), 3.48 – 3.39 (m, 3H, OCH*H'*$CH_2CH_2N_3$, OCH_2CH_2C*H_2*N_3), 1.87 (m, 2H, OCH_2C*H_2*CH_2N_3).

$^{13}C\{^1H\}$ NMR (75 MHz, $CDCl_3$ + CD_3OD): δ 139.5, 139.3, 139.2 (*ipso*-Ph (Bn)), 129.1, 129.0, 128.9, 128.8, 128.5, 128.3, 128.3 (Ph (Bn), 110.7 (C-1^{II}), 100.0 (C-1^2), 98.5 (C-1^1), 85.5 (C-4^{II}), 81.8 (C-2^{II}), 79.6 (C-3^1), 78.6 (C-3^{II}), 78.1 (C-3^2), 77.1 (C-2^1), 75.9 (C-4^1), 75.6 (CH_2 (Bn_1)), 74.1 (CH_2 (Bn_2)), 73.5 (CH_2 (Bn_3)), 72.1 (C-5^{II}), 71.3 (C-5^2), 70.3 (C-4^2), 69.9 (C-5^1), 68.6 (C-2^2), 67.5 (C-6^1), 65.6 (O*C*$H_2CH_2CH_2N_3$), 63.9 (C-6^{II}), 62.3 (C-6^2), 49.1 (OCH_2CH_2*C*H_2N_3), 29.5 (OCH_2*C*$H_2CH_2N_3$).

HRMS ESI m/z calcd for $[M+Na]^+$ $C_{42}H_{55}N_3O_{16}$ 880.3475; found 880.3469.

3-Aminopropyl β-D-galactofuranosyl-(1→3)-α-D-galactopyranosyl-(1→6)]-α-D-galactopyranoside (2a)

$Pd(OH)_2$/C (20 %wt, 27 mg) and 1M aq. solution of HCl (63 μL) were added to added to a solution of trisaccharide **S4** (44.5 mg, 0.052 mmol) in a mixture of anhydrous EtOAc (1.14 mL) and anhydrous MeOH (2.25 mL). The reaction mixture was intensively stirred under a hydrogen atmosphere for 25 minutes. Then, the mixture was diluted with MeOH and water, the catalyst was filtered and the filtrate was concentrated under vacuum. Trisaccharide **2a** was purified by gel-permeation chromatography on the TSK HW-40(S) column and isolated as a fluffy solid (28.8 mg, 99%). R_f = 0.19 (^{n}BuOH : EtOH : H_2O : NH_3 0.5 : 1 : 0.8 : 0.8). $[\alpha]_D^{25}$ = 39.6 (H_2O, C = 5mg/ml).

^{1}H-NMR (600 MHz, D_2O, 303 K): δ 5.18 (s, 1H, H-1^{II}), 4.99 (d, $J_{1,2}$ = 3.8 Hz, 1H, H-1^2), 4.96 (d, $J_{1,2}$ = 2.5 Hz, 1H, H-1^1), 4.18 (br s, 1H, H-2^{II}), 4.13 – 4.10 (m, 2H, H-4^2, H-5^1), 4.06 (dd, $J_{3,4}$ = 3.6 Hz, $J_{3,2}$ = 6.6 Hz, 1H, H-3^{II}), 4.03 (m, 2H, H-4^2, H-4^1), 4.00 (t, $J_{5,6A}$ = $J_{5,6B}$ = 6.2 Hz, 1H, H-5^2), 3.95 (dd, $J_{2,3}$ = 10.3 Hz, $J_{2,1}$ = 3.8 Hz, 1H, H-2^2), 3.92 (m, 1H, OC*H*H'$CH_2CH_2NH_2$), 3.87 (m, 2H, H-3^2, H-6A^1), 3.86 – 3.81 (m, 3H, H-5^{II}, H-2^1, H-3^1), 3.76 – 3.71 (m, 3H, H-6A^2, H-6B^2, H-6B^1), 3.69 (dd, $J_{6A,6B}$ = 11.6 Hz, $J_{6A,5}$ = 4.6 Hz, 1H, H-6A^{II}), 3.65 – 3.59 (m, 2H, H-6B^{II}, OCH*H'*$CH_2CH_2NH_2$), 3.16 (m, 2H, OCH_2CH_2C*H_2*NH_2), 2.01 (m, 2H, OCH_2C*H_2*CH_2NH_2).

^{13}C{^{1}H} NMR (150 MHz, D_2O, 303 K): δ 109.7 (C-1^{II}), 99.2 (C-1^1), 98.8 (C-1^2), 83.4 (C-4^{II}), 82.0 (C-2^{II}), 77.8 (C-3^2), 77.4 (C-3^{II}), 71.5 (C-5^2), 71.2 (C-5^{II}), 70.0 (C-2^1), 69.9 (C-4^2), 69.5 (C-5^1), 68.7 (C-3^1), 67.9 (C-2^2), 67.1 (C-6^1), 66.6 (O*C*$H_2CH_2CH_2NH_2$), 63.3 (C-6^{II}), 61.7 (C-6^2), 38.5 (OCH_2CH_2*C*H_2NH_2), 27.1 (OCH_2*C*$H_2CH_2NH_2$).

HRMS ESI m/z calcd for $[M+Na]^+$ $C_{21}H_{35}NO_{16}$ 584.2153; found 584.2161.

Conjugate of the (1→3)-trisaccharide with biotin (2b)

A solution of activated ester of biotin in DMF[5] (62 µmol/ml, 141 µL 0.0088 mmol) and dry Et_3N (8.8 µL) was added to a solution of trisaccharide **2a** (4.1 mg, 0.0073 mmol) in DMF (300 µL). The mixture was stirred for 30 minutes and then loaded onto the TSK HW-40(S) column and washed with a 0.1 M solution of AcOH. The fractions with the product were combined and dried by lyophilization to afford **2b** (7.0 mg, 85%). R_f = 0.55 (BPS : AMW 1 : 1).

Characteristic signals in ^{1}H-NMR (600 MHz, D_2O, 303 K): δ 5.19 (s, 1H, H-1^{II}), 5.00 (d, $J_{1,2}$ = 3.8 Hz, 1H, H-1^2), 4.95 (d, $J_{1,2}$ = 3.8 Hz, 1H, H-1^1), 4.62 (m, 1H, H-6a biotin), 4.43 (m, 1H, H-3a biotin), 3.72 (t, *J* = 6.2 Hz, 3H, OC*H_2*CH_2O biotin), 3.64 (t, *J* = 6.0 Hz, 3H, C*H_2*O biotin), 3.56 (m, 1H, OCH*H'*$CH_2CH_2NH_2$), 3.40 (t, *J* = 5.4 Hz, 2H, C*H_2*NH biotin), 3.33 (m, 3H, biotin, OCH_2CH_2C*H_2*NH_2), 3.01 (dd, *J* = 5.1 Hz, *J* = 13.1 Hz, 1H, H-6A biotin), 2.79 (d, *J* = 13.1 Hz, 1H, H-6B biotin), 2.53 (t, *J* = 6.2 Hz, 2H, C(O)CH_2C*H_2*O biotin), 2.28 (t, *J* = 7.1 Hz, 2H, Hα biotin), 1.90 – 1.52 (m, 7H, HΔ-A biotin, HΔ-B biotin, H-β biotin, H-γ biotin, OCH_2C*H_2*CH_2NH_2).

HRMS ESI m/z calcd for $[M+Na]^+$ $C_{46}H_{82}N_4O_{25}S$ 1145.4874; found 1145.4881.

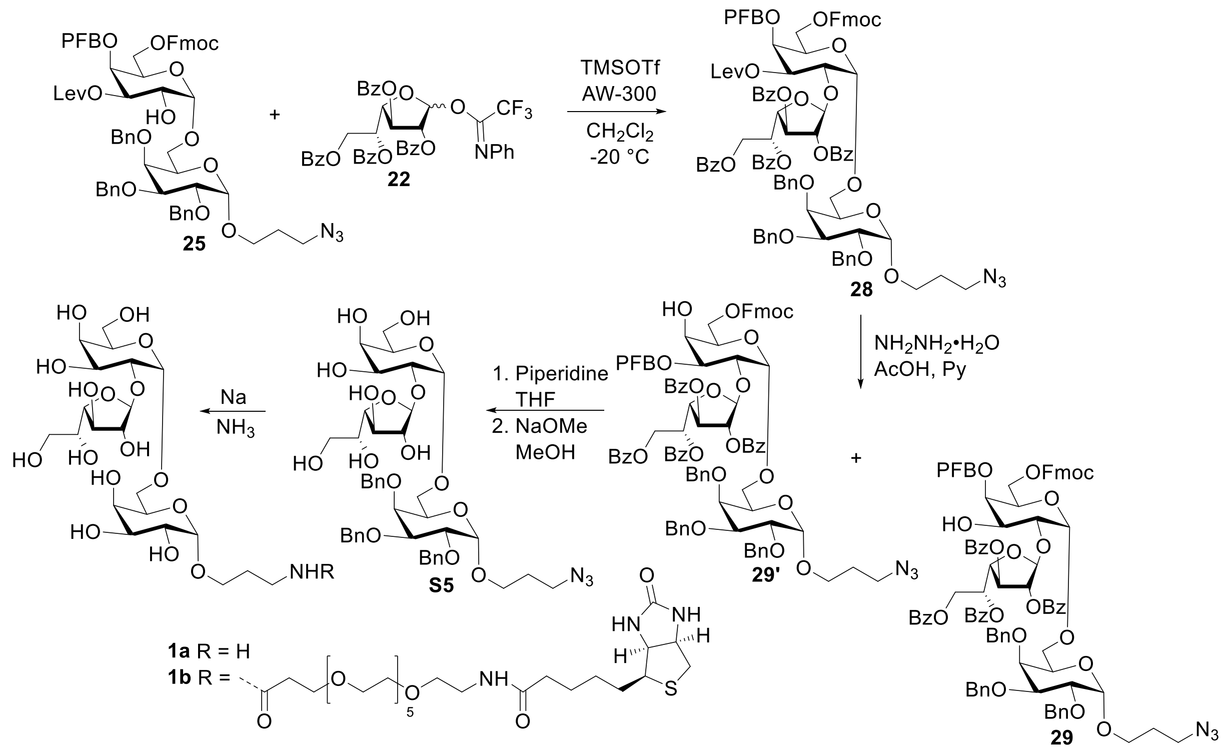

3-Azidopropyl 2,3,4,6-tetra-*O*-benzoyl-β-D-galactofuranosyl-(1→2)-6-*O*-fluorenylmethyloxycarbonyl-3-*O*-levulinyl-4-*O*-pentafluorobenzoyl-α-D-galactopyranosyl-(1→6)-2,3,4-tri-*O*-benzyl-α-D-galactopyranoside (28)

Molecular sieves AW-300 (870 mg) were added to a solution of donor **22** (423.8 mg, 0.552 mmol) and acceptor **25** (445.0 mg, 0.368 mmol) in dry CH_2Cl_2 (8.7 mL) under argon atmosphere at –20 °C. The mixture was stirred for 40 minutes and TMSOTf (26 µL, 0.147 mmol) was added. In 30 minutes the mixture was slowly warmed up. In the next 2.5 hours when the temperature was –5 °C, the reaction mixture was diluted with CH_2Cl_2 and filtered through the pad of Celite. The filtrate was washed with saturated solution of $NaHCO_3$ and the aqueous layer was extracted with CH_2Cl_2 three times. The combined organic extracts were dried over anhydrous Na_2SO_4, filtered and concentrated under vacuum. The residue was purified by column chromatography (toluene : EtOAc 50 : 1 → 12 : 1) to give trisaccharide **28** (572.5 mg, 87%) as a colorless oil. R_f = 0.47 (toluene : EtOAc 5 : 1). $[\alpha]_D^{22}$ = 39.5.

^{1}H-NMR (600 MHz, $CDCl_3$): δ 8.01 (m, 2H, Ph (Bz)), 7.97 (d, J = 7.5 Hz, 2H, Ph (Bz)), 7.83 (d, J = 7.5 Hz, 2H, Ph (Bz)), 7.74 (d, J = 7.5 Hz, 2H, Fmoc), 7.57 (d, J = 8.1 Hz, 2H, Fmoc), 7.52 – 7.45 (m, 4H, Ph (Bz), Fmoc), 7.41 – 7.33 (m, 11H, Ph (Bn, Bz)), 7.32 – 7.21 (m, 22H, Ph (Bn, Bz), Fmoc + $CHCl_3$), 7.19 – 7.14 (m, 3H, Ph (Bz)), 6.00 (m, 1H, H-5^{I}), 5.76 (d, $J_{4,3}$ = 3.3 Hz, 1H, H-4^{2}), 5.66 (dd, $J_{3,4}$ = 5.1 Hz, $J_{3,2}$ = 1.3 Hz, 1H, H-3^{I}), 5.50 (dd, $J_{3,4}$ = 3.4 Hz, $J_{3,2}$ = 10.5 Hz, 1H, H-3^{2}), 5.47 (d, $J_{2,3}$ = 1.3 Hz, 1H, H-2^{I}), 5.42 (s, 1H, H-1^{I}), 4.99 (d, $J_{1,2}$ = 3.5 Hz, 1H, H-1^{2}), 4.88 – 4.82 (m, 3H, H-1^{1}, Bn_1A, Bn_3A), 4.76 (d, J = 12.0 Hz, 1H, Bn_2A), 4.74 – 4.69 (m, H-6A^{I}, H-6B^{I}, Bn_3B), 4.61 (d, J = 11.7 Hz, 1H, Bn_2B), 4.53 (m, 3H, H-4^{I}, H-5^{2}, Bn_1B), 4.38 (dd, J = 7.2 Hz, J = 10.5 Hz, 1H, C*H*H' (Fmoc)), 4.33 (dd, $J_{6A,6B}$ = 11.0 Hz, $J_{6A,5}$ = 7.2 Hz, 1H, H-6A^{2}), 4.28 (dd, J = 7.8 Hz, J = 10.5 Hz, 1H, CH*H'* (Fmoc)), 4.21 (dd, $J_{6B,6A}$ = 11.0 Hz, $J_{6A,5}$ = 6.3 Hz, 1H, H-6B^{2}), 4.16 (t, J = 7.5 Hz, 1H, CH (Fmoc)), 4.08 (dd, $J_{2,3}$ = 10.5 Hz, $J_{2,1}$ = 3.3 Hz, 1H, H-2^{2}), 4.00 (dd, $J_{2,3}$ = 10.1 Hz, $J_{2,1}$ = 3.7 Hz, 1H, H-2^{1}), 3.90 – 3.82 (m, 3H, H-3^{1}, H-5^{1}, H-6A), 3.76 (m, 1H, OC*H*H'$CH_2CH_2N_3$), 3.71 (d, $J_{4,3}$ = 2.3 Hz, 1H, H-4^{1}), 3.54 (m, 1H, OCH*H'*$CH_2CH_2N_3$), 3.37 (t, J = 6.8 Hz, 2H, $OCH_2CH_2CH_2N_3$), 3.24 (m, 1H, H-6B^{1}), 2.81 – 2.47 (m, 4H, CH_2 (Lev)), 2.07 (s, 3H, Me (Lev)), 1.90 (m, 2H, $OCH_2CH_2CH_2N_3$).

^{13}C{^{1}H} NMR (150 MHz, $CDCl_3$): δ 172 (C=O (Lev)), 166.1, 165.7, 165.4, 165.3 (C=O (Bz)), 154.7, 143.5, 143.1, 141.2 (Fmoc), 138.1, (*ipso*-Ph (Bn)), 133.5, 133.4, 133.3, 133.1 (*ipso*-Ph (Bz)), 129.9, 129.8, 129.7, 129.7, 128.9, 128.4, 128.3, 128.3, 127.8, 127.8, 127.8, 127.7, 127.6, 127.5, 127.3, 127.2, 127.1, 125.2, 125.1 (Ph (Bn, Bz), Fmoc), 120.0 (Fmoc), 107.2 (C-1^{I}), 97.9 (C-1^{2}), 97.4 (C-1^{1}), 81.7 (C-2^{I}), 81.7 (C-4^{I}), 79.0 (C-3^{1}), 77.2 (C-3^{I}), 77.1 (C-2^{1}), 75.1 (C-4^{1}), 74.5 (CH_2 (Bn_1)), 73.3 (CH_2 (Bn_2)), 73.3 (CH_2 (Bn_3)), 73.0 (C-2^{2}), 72.9 (C-4^{2}), 70.3 (C-5^{I}), 70.2 (CH_2 (Fmoc)), 69.8 (C-5^{1}), 68.6 (C-6^{1}), 68.5 (C-3^{2}), 65.6 (C-5^{2}), 65.1 (O*C*H$_2$CH$_2$CH$_2$N$_3$), 64.(C-6^{2}), 63.5 (C-6^{I}), 48.3 (OCH$_2$CH$_2$*C*H$_2$N$_3$), 46.6 (CH (Fmoc)), 37.9 (CH_2 (Lev)), 29.5 (Me (Lev)), 28.7 (OCH$_2$*C*H$_2$CH$_2$N$_3$), 27.7 (CH_2 (Lev)).

HRMS ESI m/z calcd for $[M+Na]^+$ $C_{92}H_{80}F_5N_3O_{23}$ 1810.5353; found 1810.5382.

3-Azidopropyl 2,3,4,6-tetra-*O*-benzoyl-β-D-galactofuranosyl-(1→2)-6-*O*-fluorenylmethyloxycarbonyl-4-*O*-pentafluorobenzoyl-α-D-galactopyranosyl-(1→6)-2,3,4-tri-*O*-benzyl-α-D-galactopyranoside (29) and 3-azidopropyl 2,3,4,6-tetra-*O*-benzoyl-β-D-galactofuranosyl-(1→2)-6-*O*-fluorenylmethyloxycarbonyl-3-*O*-pentafluorobenzoyl-α-D-galactopyranosyl-(1→6)-2,3,4-tri-*O*-benzyl-α-D-galactopyranoside (29)

Trisaccharide **28** (550.0 mg, 0.308 mmol) was dissolved in a mixture of hydrazine monohydrate (150 μL), pyridine (1.83 mL) and AcOH (1.22 mL). The reaction mixture was stirred for 10 minutes, quenched with acetone (3 mL) and then the solvents were evaporated under reduced pressure. The dry residue was dissolved in toluene (5 mL) and concentrated under vacuum several times. Column chromatography of the residue (silica gel, toluene : MTBE 40 : 1 → 10 : 1) provided 3-OH-trisacchatide **29** (138.5 mg, 27%), 4-OH-trisaccharide **29i** (152.0 mg, 29%), and their mixture **29m** (148.4 mg, 29%).

Data for trisaccharide **29**. White powder. R_f = 0.38 (toluene : EtOAc 9 : 1). $[\alpha]_D^{29}$ = 55.6.

^{1}H-NMR (300 MHz, $CDCl_3$): δ 8.08 – 7.88 (m, 8H, Ph (Bz), Fmoc), 7.78 (d, J = 7.3 Hz, 2H, Ph (Bz)), 7.62 (t, J = 6.9 Hz, 2H, Fmoc), 7.52 (m, 4H, Fmoc), 7.45 – 7.15 (m, 12H, Ph (Bn, Bz) + toluene + $CHCl_3$), 5.93 (m, 1H, H-5^{II}), 5.82 (d, $J_{4,3}$ = 3.2 Hz, 1H, H-4^{2}), 5.76 (dd, $J_{3,4}$ = 6.6 Hz, $J_{3,2}$ = 2.5 Hz, 1H, H-3^{II}), 5.51 (m, 2H, H-1^{II}, H-2^{II}), 5.07 (d, $J_{1,2}$ = 3.4 Hz, 1H, H-1^{2}), 4.94 (d, J= 11.4 Hz, 1H, Bn_1A), 4.90 – 4.83 (m, 2H, H-1^{1}, Bn_3A), 4.80 (d, J= 11.7 Hz, 1H, Bn_2A), 4.78 – 4.70 (m, 3H, H-6A^{II}, H-6B^{II}, Bn_3B), 4.70 – 4.59 (m, 3H, H-4^{II}, Bn_1B, Bn_2B), 4.51 – 4.31 (m, 5H, H-3^{2}, H-5^{2}, H-6A^{2}, CH_2 (Fmoc)), 4.31 – 4.18 (m, 2H, H-6B^{1}, CH (Fmoc)), 4.05 (dd, $J_{2,3}$ = 10.0 Hz, $J_{2,1}$ = 3.6 Hz, 1H, H-2^{1}), 4.01 – 3.86 (m, 5H, H-2^{2}, H-3^{2}, H-4^{1}, H-5^{1}, H-6A^{1}), 3.77 (m, 1H, OC*H*H'CH$_2$CH$_2$N$_3$), 3.53 (m, 1H, OCH*H'*CH$_2$CH$_2$N$_3$), 3.45 (m, 1H, H-6B^{1}), 3.53 (t, J = 6.5 Hz, 2H, OCH$_2$CH$_2$C*H*$_2$N$_3$), 1.89 (m, 2H, OCH$_2$C*H*$_2$CH$_2$N$_3$).

^{13}C{^{1}H} NMR (75 MHz,$CDCl_3$): δ 166.2 (C=O (Bz)), 165.7 (C=O (Bz)), 154.7 (Fmoc), 143.2 (Fmoc), 138.5, 138.3, 133.7, 133.6, 133.4, 133.2, 130.0, 129.9, 129.8, 129.5, 128.8, 128.5, 128.4, 128.4, 128.3, 127.8, 127.8, 127.7, 127.5, 127.3, 127.2, 125.3, 125.2 (Ph (Bn, Bz)), 120.0 (Fmoc), 108.1 (C-1^{II}), 98.1 (C-1^{2}), 97.6 (C-1^{1}), 83.1 (C-2^{II}), 81.4 (C-4^{II}), 79.0 (C-3^{1}), 77.5 (C-2^{2}), 77.0 (C-3^{II}, C-2^{1}), 75.3 (C-4^{1}), 74.6 (CH_2 (Bn_1)), 73.5 (CH_2 (Bn_2)), 73.2 (CH_2 (Bn_3)), 72.2 (C-4^{2}), 70.2 (CH_2 (Fmoc)), 70.1 (C-5^{II}), 69.9 (C-5^{1}), 68.5 (C-6^{1}), 67.3 (C-3^{2}), 66.1 (C-5^{2}), 65.0 (C-6^{1}), 64.8 (O*C*H$_2$CH$_2$CH$_2$N$_3$), 63.4 (C-6^{II}), 48.3 (OCH$_2$CH$_2$*C*H$_2$N$_3$), 46.7 (CH (Fmoc), 28.7 (OCH$_2$*C*H$_2$CH$_2$N$_3$).

HRMS ESI m/z calcd for $[M+Na]^+$ $C_{92}H_{80}F_5N_3O_{23}$ 1712.4995; found 1712.4983.

Data for trisaccharide **29i**. White powder. R_f = 0.31 (toluene : EtOAc 9 : 1). $[\alpha]_D^{25}$ = 44.2.

^{1}H-NMR (600 MHz, $CDCl_3$): δ 8.02 (d, J = 7.1 Hz, 2H, Ph (Bz)), 7.99 (d, J = 7.1 Hz, 2H, Ph (Bz)), 7.96 (d, J = 7.1 Hz, 2H, Ph (Bz)), 7.76 (d, J =7.3 Hz, 2H, Fmoc), 7.73 (d, J =7.3 Hz, 2H, Fmoc), 7.60

(dd, J = 3.3 Hz, J=7.8 Hz, 2H, Fmoc), 7.54 – 7.45 (m, 5H, Ph (Bz), Fmoc), 7.42 – 7.23 (m, 31H, Ph (Bn, Bz)), 5.96 (m, 1H, H-5^{II}), 5.65 (dd, $J_{3,4}$ = 1.8 Hz, $J_{3,2}$ = 5.5 Hz, 1H, H-3^{II}), 5.63 (dd, $J_{3,4}$ = 3.1 Hz, $J_{3,2}$ = 10.4 Hz, 1H, H-3^{2}), 5.46 (d, J = 1.8 Hz, 1H, H-2^{II}), 5.40 (s, 1H, H-1^{II}), 5.06 (d, $J_{1,2}$ = 3.5 Hz, 1H, H-1^{2}), 4.89 (d, J = 11.5 Hz, 1H, Bn_1A), 4.87 (d, $J_{1,2}$ = 3.9 Hz, 1H, H-1^{1}), 4.83 (d, J =11.7 Hz, 1H, Bn_2A), 4.78 (d, J = 11.9 Hz, 1H, Bn_3A), 7.74 – 4.69 (m, 3H, H-6A^{II}, H-6B^{II}, Bn_3B), 4.63 (d, J = 11.7 Hz, 1H, Bn_2B), 4.59 – 4.55 (m, 2H, H-4^{II}, Bn_1B), 4.47 (dd, $J_{6A,6B}$ = 12.8 Hz, $J_{6A,5}$ = 9.0 Hz, 1H, H-6A^{2}), 4.41 (d, J = 7.4, 2H, CH_2 (Fmoc)), 4.32 – 4.23 (m, 4H, H-2^{2}, H-4^{2}, H-5^{2}, H-6B^{2}), 4.19 (m, J = 7.4 Hz, 1H, CH (Fmoc)), 4.02 (dd, $J_{2,3}$ = 10.4 Hz, $J_{2,1}$ = 3.8 Hz, 1H, H-2^{1}), 3.96 – 3.88 (m, 3H, H-3^{1}, H-5^{1}, H-6A^{1}), 3.81 (d, $J_{4,3}$ = 2.7 Hz, 1H, H-4^{1}), 3.79 (m, 1H, OC*H*H'$CH_2CH_2N_3$), 3.54 (m, 1H, OCH*H'*$CH_2CH_2N_3$), 3.38 (t, J = 6.6 Hz, 2H, $OCH_2CH_2CH_2N_3$), 3.23 (dd, $J_{6B,6A}$ = 9.5 Hz, $J_{6B,5}$=5.5 Hz, 1H, H-6B^{1}), 2.46 (br s, 1H, OH), 1.90 (m, 2H, $OCH_2CH_2CH_2N_3$).

$^{13}C\{^1H\}$ NMR (150 MHz,$CDCl_3$): δ 165.6 (C=O (Bz)), 165.3 (C=O (Bz)), 141.2 (Fmoc), 138.7 (*ipso*-Ph (Bn)), 133.4, 133.3, 133.2, 133.1, 129.8, 129.6, 129.4, 128.8, 128.5, 128.3, 128.2, 128.2, 127.8, 127.8, 127.7, 127.6, 127.5, 127.4, 127.2, 127.1, 125.0 (Ph (Bn, Bz), Fmoc), 119.9 (Fmoc), 107.5 (C-1^{II}), 98.0 (C-1^{2}), 97.4 (C-1^{1}), 81.6 (C-2^{II}), 81.2 (C-4^{II}), 79.0 (C-3^{1}), 76.7 (C-3^{II}), 76.6 (C-2^{1}), 75.1 (C-4^{1}), 74.4 (Bn_1), 73.6 (C-2^{2}), 73.3 (Bn_2), 73.1 (C-3^{2}), 73.0 (Bn_3), 70.1 (C-5^{II}), 70.0 (CH_2 (Fmoc)), 69.8 (C-5^{1}), 68.4 (C-6^{1}), 67.6 (C-4^{2}), 67.0 (C-5^{2}), 65.4 (C-6^{2}), 64.9 ($OCH_2CH_2CH_2N_3$), 63.4 (C-6^{II}), 48.2 ($OCH_2CH_2CH_2N_3$), 46.6 (CH (Fmoc), 28.6 ($OCH_2CH_2CH_2N_3$).

HRMS ESI m/z calcd for $[M+Na]^+$ $C_{92}H_{80}F_5N_3O_{23}$ 1712.4995; found 1712.4985.

3-Azidopropyl β-D-galactofuranosyl-(1→2)-α-D-galactopyranosyl-(1→6)-2,3,4-tri-*O*-benzyl-α-D-galactopyranoside (S5)

Piperidine (83 μL, 0.852 mmol) was added to a solution of trisaccharide **29m** (120.0 mg, 0.071 mmol) in anhydroud THF (1.2 mL) at 0 °C. In 25 minutes, the reaction was completed, the mixture was diluted with toluene, and the solvents were evaporated under vacuum. The dry residue was dissolved in a mixture of anhydrous MeOH (500 μL) and anhydrous CH_2Cl_2 (100 μL), 1M solution of NaOMe in MeOH (10 μL) was added and the mixture was stirred for 3.5 hours. Then NaOMe was quenched with AcOH (4.0 μL) and the solvent was evaporated under vacuum. Column chromatography of the residue (silica gel, $CHCl_3$: MeOH 10 : 1 → 5 : 1) provided trisaccharide **S5** as a white powder (18.2 mg, 58%). R_f = 0.29 ($CHCl_3$: MeOH 15 : 1). $[\alpha]_D^{29}$ = 62.4.

^1H-NMR (600 MHz, $CDCl_3$): δ 7.33 – 7.16 (m, 16H, Ph (Bn) + $CHCl_3$), 5.11 (s, 1H, H-1^{I}), 4.90 (s, 1H, H-1'), 4.87 – 4.77 (m, 2H, H-1^{1}, Bn_1A), 4.71 (m, 2H, Bn_3A, Bn_2A), 4.64 (d, J = 11.8 Hz, 1H, Bn_3B), 4.60 – 4.52 (m, 2H, Bn_1B, Bn_2B), 4.11 (br s, 1H, H-2^{I}), 4.03 (br t, 1H, H-3^{I}), 3.97 – 3.85 (m, 5H, H-2^{1}, H-3^{1}, H-4^{1}, H-5^{1}, H-4^{2}), 3.85 – 3.75 (m, 5H, H-2^{2}, H-3^{2}, H-5^{2}', H-4^{I}, H-6A^{1}), 3.73 (m, 1H, H-5^{I}), 3.64 (m, 3H, H-6A^{2}, H-6B^{2}, OC*H*H'$CH_2CH_2N_3$), 3.57 (m, 1H, H-6A^{I}), 3.51 (m, 1H, H-6B^{I}), 3.46 (m, 1H, H-6B^{1}), 3.39 (m, 1H, OCH*H'*$CH_2CH_2N_3$), 3.28 (m, 2H, $OCH_2CH_2CH_2N_3$), 1.76 (m, 2H, $OCH_2CH_2CH_2N_3$).

$^{13}C\{^1H\}$ NMR (150 MHz, $CDCl_3$): δ 138.6, 138.4, 138.3 (*ipso*-Ph (Bn)), 128.4, 128.3, 127.9, 127.7, 127.5, 127.4 (Ph (Bn), 109.1 (C-1^{I}), 98.5 (C-1^{2}), 97.6 (C-1^{1}), 83.0 (C-4^{I}), 80.8 (C-2^{I}), 78.8 (C-3^{1}), 76.4 (C-2^{1}), 76.3 (C-3^{I}), 75.9 (C-2^{2}), 75.4 (C-4^{1}), 74.5 (CH_2 (Bn_1)), 73.3 (CH_2 (Bn_2)), 73.0 (CH_2 (Bn_3)), 70.4 (C-5^{I}), 70.0 (C-5^{1}), 69.7 (C-3^{2}, C-4^{2}), 68.8 (C-5^{2}), 67.9 (C-6^{1}), 65.0 ($OCH_2CH_2CH_2N_3$), 63.6 (C-6^{I}), 61.5 (C-6^{2}), 48.4 ($OCH_2CH_2CH_2N_3$), 28.6 ($OCH_2CH_2CH_2N_3$).

HRMS ESI m/z calcd for $[M+Na]^+$ $C_{42}H_{55}N_3O_{16}$ 880.3475; found 880.3461.

3-Aminopropyl β-D-galactofuranosyl-(1→2)-α-D-galactopyranosyl-(1→6)]-α-D-galactopyranoside (1a)

Trisaccharide **S5** (12.8 mg, 0.015 mmol) was dissolved in anhydrous THF (500 µL) and added dropwise to 5 mL of a solution of Na (30 mg) in liquid ammonia at −60 °C. After 50 min, the mixture was quenched with MeOH, and the ammonia was removed with the stream of argon. Then AcOH (170 µL) was added until pH = 7 and the solvents were evaporated. The dry residue was purified to gel-permeation chromatography on a TSK-40 column in 0.1 M AcOH to give trisaccharide **1a** (8.2 mg, 98%) as a fluffy solid. R_f = 0.15 (^{n}BuOH : EtOH : H_2O : NH_3 0.5 :1 : 0.8 : 0.8). $[\alpha]_D^{25}$ = 7.4 (C = 5mg/ml, H_2O).

^{1}H-NMR (600 MHz, D_2O, 303 K): δ 5.13 (s, 1H, H-1^I), 5.09 (s, 1H, H-1^2), 4.95 (s, 1H, H-1^1), 4.15 (br s, 1H, H-2^I), 4.11 – 4.04 (m, 2H, H-3^I, H-5^1), 4.03 – 3.95 (m, 4H, H-4^I, H-4^2, H-5^2, H-4^1), 3.93 – 3.78 (m, 7H, H-5^I, H-2^2, H-3^2, H-2^1, H-3^1, H-6A^1, OC*H*H'$CH_2CH_2NH_2$), 3.77 – 3.63 (m, 5H, H-6A^I, H-6B^I, H-6A^2, H-6B^2, H-6B^1), 3.60 (m, 1H, OCH*H*'$CH_2CH_2NH_2$), 3.15 (m, 2H, $OCH_2CH_2C\mathit{H}_2NH_2$), 2.00 (m, 2H, $OCH_2C\mathit{H}_2CH_2NH_2$).

^{13}C{^{1}H} NMR (150 MHz, D_2O, 303 K): δ 110.0 (C-1^I), 99.2 (C-1^2, C-1^1), 83.3 (C-4^I), 82.0 (C-2^I), 77.2 (C-3^I), 77.1 (C-2^2), 71.4 (C-5^2), 71.2 (C-5^I), 70.1 (C-2^1), 69.9 (C-4^2, C-4^1), 69.8 (C-5^1), 69.0 (C-3^2), 68.7 (C-3^1), 67.9 (C-6^1), 66.7 ($O\mathit{C}H_2CH_2CH_2NH_2$), 63.2 (C-6^I), 61.7 (C-6^2), 38.6 ($OCH_2CH_2\mathit{C}H_2NH_2$), 27.1 ($OCH_2\mathit{C}H_2CH_2NH_2$).

HRMS ESI m/z calcd for $[M+Na]^+$ $C_{21}H_{35}NO_{16}$ 584.2153; found 584.2161.

Conjugate of the (1→2)-trisaccharide with biotin (1b)

A solution of activated ester of biotin in DMF[5] (62 µmol/ml, 141 µL 0.0088 mmol) and dry Et_3N (8.8 µL) was added to a solution of trisaccharide **1a** (4.1 mg, 0.0073 mmol) in DMF (300 µL). The mixture was stirred for 30 minutes and then loaded onto the TSK HW-40(S) column and washed with a 0.1 M solution of AcOH. The fractions with the product were combined and dried by lyophilization to afford **1b** (4.1 mg, 50%). R_f = 0.55 (BPS : AMW 1 : 1).

Characteristic signals in ^{1}H-NMR (600 MHz, D_2O, 303 K): δ 5.16 (s, 1H, H-1^I), 5.09 (d, $J_{1,2}$ = 3.6 Hz, 1H, H-1^2), 4.94 (d, $J_{1,2}$ = 3.7 Hz, 1H, H-1^1), 4.62 (m, 1H, H-6a biotin), 4.44 (m, 1H, H-3a biotin), 3.72 (t, J = 6.1 Hz, 3H, $OC\mathit{H}_2CH_2O$ biotin), 3.64 (t, J = 5.2 Hz, 3H, $C\mathit{H}_2O$ biotin), 3.56 (m, 1H, OCH*H*'$CH_2CH_2NH_2$), 3.40 (t, J = 5.2 Hz, 2H, $C\mathit{H}_2NH$ biotin), 3.33 (m, 3H, biotin, $OCH_2CH_2C\mathit{H}_2NH_2$), 3.01 (dd, J = 5.2 Hz, J = 12.9 Hz, 1H, H-6A biotin), 2.80 (d, J = 112.9 Hz, 1H, H-6B biotin), 2.53 (t, J = 6.1 Hz, 2H, $C(O)CH_2C\mathit{H}_2O$ biotin), 2.29 (t, J = 7.4 Hz, 2H, Hα biotin), 1.90–1.55 (m, 7H, HΔ-A biotin, HΔ-B biotin, H-β biotin, H-γ biotin, $OCH_2C\mathit{H}_2CH_2NH_2$).

HRMS ESI m/z calcd for $[M+Na]^+$ $C_{46}H_{82}N_4O_{25}S$ 1145.4874; found 1145.4881.

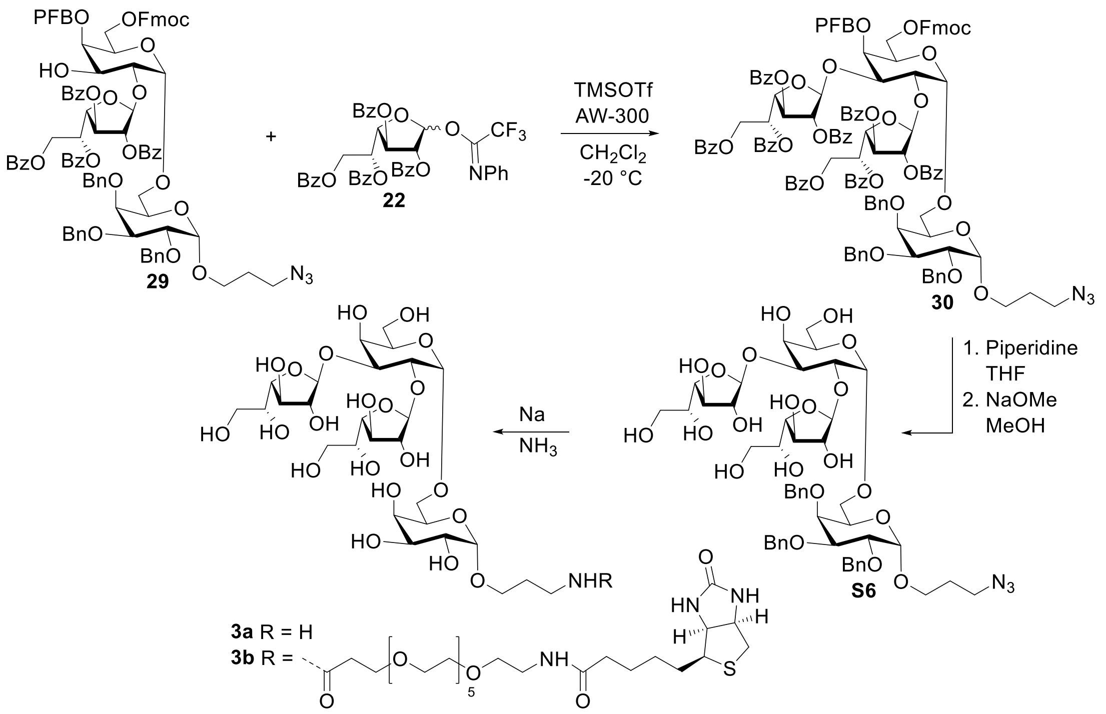

3-Azidopropyl 2,3-di-*O*-(2,3,4,6-tetra-*O*-benzoyl-β-D-galactofuranosyl)-6-*O*-fluorenylmethyloxycarbonyl-4-*O*-pentafluorobenzoyl-α-D-galactopyranosyl-(1→6)-2,3,4-tri-*O*-benzyl-α-D-galactopyranoside (30)

Molecular sieves AW-300 (140 mg) were added to a solution of donor **22** (52.5 mg, 0.068 mmol) and acceptor **29** (83.4 mg, 0.057 mmol) in dry CH_2Cl_2 (1.4 mL) under argon atmosphere at –10 °C. The mixture was stirred for 40 minutes and TMSOTf (6.2 μL, 0.147 mmol) was added. In 17 minutes the mixture was diluted with CH_2Cl_2 and filtered through the pad of Celite. The filtrate was washed with saturated solution of $NaHCO_3$ and the aqueous layer was extracted with CH_2Cl_2 three times. The combined organic extracts were dried over anhydrous Na_2SO_4, filtered and concentrated under vacuum. The residue was purified by column chromatography (toluene : EtOAc 50 : 1→15 : 1) to give tetrasaccharide **30** (90.3 mg, 70%) as a colorless oil. R_f = 0.36 (toluene : EtOAc 15 : 1). $[\alpha]_D^{31}$ = 32.3.

^{1}H-NMR (300 MHz, $CDCl_3$): δ 8.10 – 7.97 (m, 11H, Ph (Bz)), 7.91 – 7.82 (m, 4H, Ph (Bz)), 7.72 (m, 1H, Ph (Bz)), 7.70 (m, 1H, Ph (Bz)), 7.66 – 7.62 (m, 2H, Fmoc), 7.62 – 7.54 (m, 2H, Fmoc), 7.51 – 7.42 (m, 7H, Ph (Bz), Fmoc), 7.42 – 7.17 (m, 48 H, Ph (Bn, Bz) + $CHCl_3$), 7.08 (m, 2H, Ph (Bz)), 6.24 (m, 1H, H-5^{II}), 6.11 (m, 1H, H-5^{I}), 5.78 (d, $J_{4,3}$ = 3.2 Hz, 1H, H-4^2), 5.74 (m, 2H, H-1^{I}, H-1^{II}), 5.67 (d, $J_{3,4}$ = 6.6 Hz, 1H, H-3^{I}), 5.66 (d, $J_{2,3}$ = 1.0 Hz, 1H, H-2^{I}), 5.45 (d, $J_{2,3}$ = 1.0 Hz, 1H, H-2^{II}), 5.42 (d, $J_{3,4}$ = 5.3 Hz, 1H, H-3^{II}), 5.11 (dd, $J_{4,5}$ = 3.3 Hz, $J_{4,3}$ = 5.3 Hz, 1H, H-4^{II}), 4.98 (d, $J_{1,2}$ = 3.4 Hz, 1H, H-1^2), 4.88 – 4.81 (m, 3H, H-1^1, Bn_1A, Bn_2A), 4.81 – 4.66 (m, 6H, H-6A^{I}, H-6B^{I}, H-6A^{II}, H-6B^{II}, Bn_2B, Bn_3A), 4.63 (d, J = 11.9 Hz, 1H, Bn_3B), 4.56 – 4.44 (m, 2H, H-5^2, Bn_1B), 4.41 (m, 2H, H-3^2, C*H*H' (Fmoc)), 4.31 (dd, J = 7.7 Hz, J = 10.4 Hz, 1H, CH*H'* (Fmoc)), 4.23 – 4.09 (m, 4H, H-2^2, H-6A^2, H-6B^2, CH (Fmoc)), 4.01 (dd, $J_{2,3}$ = 9.8 Hz, $J_{2,1}$ = 3.4 Hz, 1H, H-2^1), 3.90 – 3.83 (m, 3H, H-3^1, H-5^1, H-6A^1), 3.77 (m, 1H, OC*H*H'$CH_2CH_2N_3$), 3.55 (d, $J_{4,3}$ = 3.0 Hz, 1H, H-4^1), 3.50 (m, 1H, OCH*H'*$CH_2CH_2N_3$), 3.41 (t, J = 6.8 Hz, 2H, $OCH_2CH_2CH_2N_3$), 3.16 (m, 1H, H-6B), 1.93 (m, 2H, $OCH_2CH_2CH_2N_3$).

^{13}C{^{1}H} NMR (75 MHz,$CDCl_3$): δ 166.2, 166.1, 165.9, 165.8, 165.6, 165.4, 165.3 (C=O (Bz)), 154.7 (Fmoc), 143.6, 143.1, 141.3, 141.2 (*ipso*-Ph (Bz), Fmoc), 138.8, 138.5, 138.1, 133.4, 133.2, 133.1 (*ipso*-Ph (Bn, Bz)), 130.0, 129.9, 129.8, 129.5, 128.8, 128.5, 128.4, 128.4, 128.3, 128.1, 127.8, 127.8, 127.7,

127.6, 127.3, 127.2, 127.1, 125.3, 125.2, 125.1 (Ph (Bn, Bz)), 120.0 (Fmoc), 107.6 (C-1^{II}), 107.4 (C-1^{I}), 98.2 (C-1^{2}), 97.5 (C-1^{1}), 82.7 (C-4^{I}), 82.3 (C-2^{II}), 81.7 (C-2^{I}), 81.6 (C-4^{II}), 79.1 (C-3^{1}), 78.8 (C-3^{II}), 77.6 (C-3^{I}), 76.7 (C-2^{1}), 75.1 (C-4^{1}), 74.8 (C-2^{2}), 74.4 (CH_2 (Bn_1)), 73.3 (CH_2 (Bn_2, Bn_3)),72.9 (C-3^{2}, C-4^{2}), 70.5 (C-5^{II}), 70.4 (C-5^{I}), 70.1 (CH_2 (Fmoc)), 69.9 (C-5^{1}), 69.1 (C-6^{1}), 66.0 (C-5^{2}), 65.1 (C-6^{2}), 64.8 (O*C*$H_2CH_2CH_2N_3$), 63.7 (C-6^{I}), 63.3 (C-6^{II}), 48.4 (OCH_2CH_2*C*H_2N_3), 46.6 (CH (Fmoc), 28.8 (OCH_2*C*$H_2CH_2N_3$).

HRMS ESI m/z calcd for $[M+Na]^+$ $C_{126}H_{106}F_5N_3O_{32}$ 2290.6572; found 2290.6578.

3-Azidopropyl 2,3-di-*O*-(β-D-galactofuranosyl)-α-D-galactopyranosyl-(1→6)-2,3,4-tri-*O*-benzyl-α-D-galactopyranoside (S6)

Piperidine (63 µL, 0.646 mmol) was added to a solution of tetrasaccharide **30** (146.5 mg, 0.065 mmol) in anhydroud THF (1.5 mL) at 0 °C. In 1 hour the reaction was completed, the mixture was diluted with toluene, and the solvents were evaporated under vacuum. The dry residue was dissolved in a mixture of anhydrous MeOH (1.2 mL) and anhydrous CH_2Cl_2 (300 µL), 1M solution of NaOMe in MeOH (16 µL) was added and the mixture was stirred overnight. Then NaOMe was quenched with AcOH (1.5 µL) and the solvent was evaporated under vacuum. Column chromatography of the residue (silica gel, $CHCl_3$: MeOH 10 : 1 → 4 : 1) provided tetrasaccharide **S6** as a white powder (43.0 mg, 65%). $[\alpha]_D^{29}$ = 24.1.

^{1}H-NMR (600 MHz, CD_3OD + $CDCl_3$, 303 K): δ 7.40 – 7.24 (m, 20H, Ph (Bn) + $CHCl_3$), 5.14 (s, 1H, H-1^{I}), 5.08 (s, 1H, H-1^{II}), 4.92 (d, J = 11.1 Hz, 1H, Bn_1A), 4.85 (d, $J_{1,2}$ = 3.8 Hz, 1H, H-1^{2}), 4.80 (d, J = 11.4 Hz, Bn_3A), 4.79 – 4.74 (m, 3H, H-1^{1}, Bn_2A, Bn_3B), 4.65 (d, J = 11.9 Hz, Bn_2B), 4.59 (d, J = 11.1 Hz, 1H, Bn_1B), 4.08 (m, 1H, H-4^{II}), 4.04 (d, $J_{4,3}$ = 3.0 Hz, 1H, H-4^{2}), 4.03 – 3.99 (m, 3H, H-2^{I}, H-2^{II}, H-3^{II}), 3.98 – 3.89 (m, 7H, H-2^{1}, H-2^{2}, H-3^{1}, H-4^{1}, H-5^{1}, H-3^{I}, H-4^{II} + H_2O), 3.81 (dd, $J_{3,4}$ = 3.0 Hz, $J_{3,2}$ = 9.9 Hz, 1H, H-3^{2}), 3.80 – 3.67 (m, 6H, H-5^{I}, H-5^{II}, H-6A^{1}, H-6A^{2}, H-6B^{2}, OC*H*H'$CH_2CH_2N_3$), 3.66 – 3.56 (m, 3H, H-6A^{I}, H-6A^{II}, H-6^{II}), 3.51 (dd, $J_{6B,6A}$ = 11.1 Hz, $J_{6B,5}$ = 5.6 Hz, H-6B^{II}), 3.46 (m, 1H, OCH*H'*$CH_2CH_2N_3$), 3.44 – 3.36 (m, 3H, H-6B^{1}, OCH_2CH_2C*H_2*N_3), 1.88 (m, 2H, OCH_2C*H_2*CH_2N_3).

^{13}C{^{1}H} NMR (150 MHz, CD_3OD + $CDCl_3$, 303 K): δ 128.3, 128.2, 127.9, 127.7, 127.7 (Ph (Bn)), 109.9 (C-1^{I}), 109.2 (C-1^{II}), 98.5 (C-1^{2}), 97.6 (C-1^{1}), 85.4 (C-4^{I}, C-4^{II}), 80.2 (C-2^{I}), 79.7 (C-2^{II}), 78.8 (C-3^{1}), 77.3 (C-3^{II}), 77.7 (C-3^{I}), 76.2 (C-2^{1}), 75.9 (C-3^{2}), 75.3 (C-4^{1}), 74.6 (CH_2 (Bn_1)), 74.0 (C-2^{2}), 73.4 (CH_2 (Bn_2)), 73.0 (CH_2 (Bn_3)), 71.3 (C-5^{II}), 71.0 (C-5^{I}), 69.9 (C-4^{2}), 69.6 (C-5^{1}), 69.5 (C-5^{2}), 67.4 (C-6^{1}), 64.9 (O*C*$H_2CH_2CH_2N_3$), 63.3 (C-6^{I}), 63.2 (C-6^{II}), 61.8 (C-6^{2}), 48.3 (OCH_2CH_2*C*H_2N_3), 28.6 (OCH_2*C*$H_2CH_3N_3$).

HRMS ESI m/z calcd for $[M+Na]^+$ $C_{48}H_{65}N_3O_{21}$ 1042.4003; found 1042.4003.

3-Aminopropyl 2,3-di-*O*-(β-D-galactofuranosyl)-α-D-galactopyranosyl-(1→6)-α-D-galactopyranoside (3a)

Trisaccharide **S6** (26.9 mg, 0.026 mmol) was dissolved in anhydrous THF (700 µL) and added dropwise to 5 mL of a solution of Na (30 mg) in liquid ammonia at −60 °C. After 60 min, the mixture was quenched with MeOH, and the ammonia was removed with the stream of argon. Then AcOH (170 µL) was added until pH = 7 and the solvents were evaporated. The dry residue was purified to gel-permeation chromatography on a TSK-40 column in 0.1 M AcOH and then reversed-phase C18 column chromatography to give trisaccharide **3a** (10.7 mg, 54%) as a fluffy solid. R_f = 0.15 (^{n}BuOH : EtOH : H_2O : NH_3 0.5 : 1 : 0.8 : 0.8). $[\alpha]_D^{24}$ = 2.6 (C = 5mg/ml, H_2O).

^{1}H-NMR (600 MHz, D_2O, 303 K): δ 5.19 (s, 1H, H-1^{II}), 5.15 (s, 1H, H-1^{I}), 5.07 (s, 1H, H-1^{2}), 4.96 (s, 1H, H-1), 4.16 (br s, 1H, H-2^{II}), 4.13 (br s, 1H, H-2^{I}), 4.10 (m, 2H, H-4^{2}, H-5^{1}), 4.07 (m, 2H, H-3^{I}, H-3^{II}), 4.04 – 4.00 (m, 3H, H-4^{II}, H-5^{2}, H-4^{1}), 3.99 – 3.96 (m, 3H, H-4^{I}, H-2^{2}, H-3^{2}), 3.92 (m, 1H, OC*H*H'$CH_2CH_2NH_2$), 3.87 (m, 1H, H-6A^{1}), 3.85 – 3.81 (m, 4H, H-5^{I}, H-5^{II}, H-2^{1}, H-3^{1}), 3.76 – 3.66 (m, 6H, H-6A^{I}, H-6B^{I}, H-6A^{II}, H-6A^{2}, H-6B^{2}, H-6B^{1}), 3.66 – 3.59 (m, 2H, H-6B^{II}, OCH*H'*$CH_2CH_2NH_2$), 3.17 (m, 2H, $OCH_2CH_2CH_2NH_2$ with $C\mathit{H}_2$), 2.02 (m, 2H, $OCH_2C\mathit{H}_2CH_2NH_2$).

^{13}C{^{1}H} NMR (150 MHz, D_2O, 303 K): δ 109.8 (C-1^{I}), 109.6 (C-1^{II}), 99.2 (C-1^{1}), 99.1 (C-1^{2}), 83.6 (C-4^{II}), 83.3 (C-4^{I}), 82.1 (C-2^{I}), 82.1 (C-2^{II}), 77.7 (C-3^{II}), 77.4 (C-3^{I}), 76.0 (C-3^{2}), 75.8 (C-2^{2}), 71.3 (C-5^{I}, C-5^{II}), 71.2 (C-5^{1}), 70.1 (C-2^{1}), 70.0 (C-4^{2}), 69.9 (C-4^{1}), 69.8 (C-5^{1}), 68.7 (C-3^{1}), 67.8 (C-6^{1}), 66.7 ($O\mathit{C}H_2CH_2CH_2NH_2$), 63.3 (C-6^{II}), 63.2 (C-6^{I}), 61.6(C-6^{2}), 38.6 ($OCH_2CH_2\mathit{C}H_2NH_2$), 27.1 ($OCH_2\mathit{C}H_2CH_2NH_2$).

HRMS ESI m/z calcd for $[M+Na]^{+}$ $C_{27}H_{49}NO_{21}$ 746.2684; found 746.2689.

Conjugate of the tetrasaccharide with biotin (3b)

A solution of activated ester of biotin in DMF[5] (62 µmol/ml, 150 µL 0.0093 mmol) and dry Et_3N (9.3 µL) was added to a solution of tetrasaccharide **3a** (4.5 mg, 0.0062 mmol) in DMF (300 µL). The mixture was stirred for 2.5 hours and then loaded onto the TSK HW-40(S) column and washed with a 0.1 M solution of AcOH. The fractions with the product were combined and dried by lyophilization to afford **3b** (3.5 mg, 44%). $R_f = 0.55$ (BPS : AMW 1 : 1).

Characteristic signals in ^{1}H-NMR (600 MHz, D_2O, 303 K): δ 5.16 (s, 1H, H-1^{II}), 5.12 (s, 1H, H-1^{I}), 5.04 (d, $J_{1,2}$ = 3.2 Hz, 1H, H-1^{2}), 4.91 (d, $J_{1,2}$ = 3.9 Hz, 1H, H-1^{1}), 4.58 (m, 1H, H-6a biotin), 4.40 (m, 1H, H-3a biotin), 3.69 (m, 3H, $OC\mathit{H}_2CH_2O$ biotin), 3.60 (t, J = 5.9 Hz, 3H, $C\mathit{H}_2O$ biotin), 3.53 (m, 1H, OCH*H'*$CH_2CH_2NH_2$), 3.37 (t, J = 5.5 Hz, 2H, $C\mathit{H}_2NH$ biotin), 3.29 (m, 3H, biotin, $OCH_2CH_2C\mathit{H}_2NH_2$), 2.97 (dd, J = 4.9 Hz, J = 13.2 Hz, 1H, H-6A biotin), 2.76 (d, J = 13.2Hz, 1H, H-6B biotin), 2.50 (t, J = 5.9 Hz, 2H, $C(O)CH_2C\mathit{H}_2O$ biotin), 2.25 (t, J = 7.4 Hz, 2H, Hα biotin), 1.88–1.53 (m, 7H, HΔ-A biotin, HΔ-B biotin, H-β biotin, H-γ biotin, $OCH_2C\mathit{H}_2CH_2NH_2$).

HRMS ESI m/z calcd for $[M+Na]^{+}$ $C_{52}H_{92}N_4O_{30}S$ 1307.5399; found 1307.5409.

VI. Synthesis of disaccharide donor 33

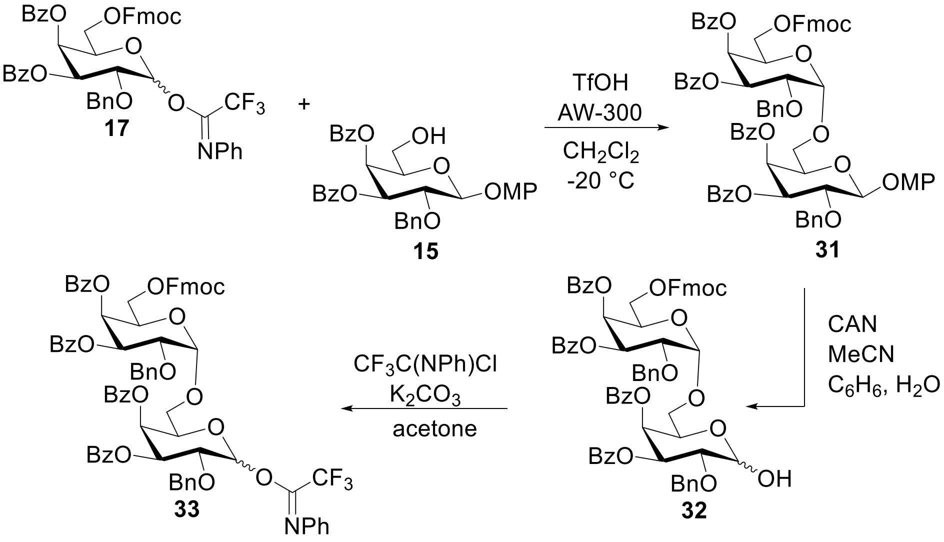

***p*-Methoxyphenyl 3,4-di-*O*-benzoyl-2-*O*-benzyl-6-*O*-fluorenylmethyloxycarbonyl-α-D-galactopyranosyl-(1→6)-3,4-di-*O*-benzoyl-2-*O*-benzyl-β-D-galactopyranoside (31)**

Molecular sieves AW-300 (5.1 g) were added to a solution of donor **17** (3.30 g, 3.80 mmol) and acceptor **15** (1.87 g, 3.20 mmol) in dry CH_2Cl_2 (51 mL) under argon atmosphere at –20 °C. The mixture was stirred for 40 minutes and TfOH (212 µL, 2.40 mmol) was added. In 7 minutes the reaction mixture was diluted with CH_2Cl_2 and filtered through the pad of Celite. The filtrate was washed with saturated solution of $NaHCO_3$ and the aqueous layer was extracted with CH_2Cl_2 three times. The combined organic extracts were dried over anhydrous Na_2SO_4, filtered and concentrated under vacuum. The residue was purified by column chromatography (toluene : EtOAc 90 : 1 → 7 : 1) to give only α-disaccharide **31** (3.18 mg, 79%). R_f = 0.38 (toluene : EtOAc 6 : 1). $[\alpha]_D^{20}$ = 123.0.

^{1}H NMR (300 Hz, $CDCl_3$): δ 8.00 (m, 2H, Ph (Bz)), 7.93 (m, 2H, Ph (Bz)), 7.81 (m, Ph (Bz)), 7.72 (d, J = 7.6 Hz, 2H, Fmoc), 7.61 – 6.99 (m, 37H, Ph (Bn, Bz), Fmoc + $CHCl_3$ + toluene), 5.82 (d, $J_{4,3}$ = 3.5 Hz, 1H, H-4^1), 5.77 (dd, $J_{3,4}$ = 3.4 Hz, $J_{3,2}$ = 10.4 Hz, 1H, H-3^2), 5.71 (d, $J_{4,3}$ = 3.4 Hz, 1H, H-4^2), 5.51 (dd, $J_{3,4}$ = 3.5 Hz, $J_{3,2}$ = 9.9 Hz, 1H, H-3^1), 5.25 (d, $J_{1,2}$ = 7.8 Hz, 1H, H-1^1), 4.98 (d, $J_{1,2}$ = 3.5 Hz, 1H, H-1^2), 4.94 (d, J = 11.5 Hz, 1H, Bn_1A), 4.77 (d, J = 11.5 Hz, 1H, Bn_1B), 4.67 (d, J = 12.4 Hz, 1H, Bn_2A), 4.62 (d, J = 12.4 Hz, 1H, Bn_2B), 4.46 (t, $J_{5,6A}$ = $J_{5,6B}$ = 6.0 Hz, H-5^2), 4.32 (m, 1H, H-5^1), 4.23 – 4.04 (m, 7H, H-2^1, H-2^2, H-6A^2, H-6B^2, CH, CH_2 (Fmoc)), 4.00 (dd, $J_{6A,6B}$ = 10.3 Hz, $J_{6A,5}$ = 7.8 Hz, 1H, H-6A^1), 3.80 (s, 3H, OMe (MP)), 3.73 (dd, $J_{6B,6A}$ = 10.3 Hz, $J_{6B,5}$ = 3.4 Hz, 1H, H-6B^1).

^{13}C{^{1}H} NMR (75 Hz, $CDCl_3$): δ 165.4 (C=O (Bz)), 155.6 (*ipso*-Ph (MP)), 150.1 (*ipso*-Ph (MP)), 133.4, 133.3, 133.1, 130.0, 129.8, 129.8, 129.7, 129.2, 128.5, 128.5, 128.3, 128.2, 128.1, 127.8, 127.7, 127.2, 125.8, 125.3 (Ph (Bn, Bz), Fmoc), 119.9 (Fmoc), 117.5 (Ph (MP)), 114.9 (Ph (MP)), 101.9 (C-1^1), 97.5 (C-1^2), 76.2 (C-2^1), 74.8 (CH_2 (Bn_1)), 73.3 (CH_2 (Bn_2)), 73.2 (C-2^2), 73.0 (C-3^1), 72.8 (C-5^1), 70.1 (CH_2 (Fmoc)), 70.0 (C-3^2), 69.6 (C-4^2), 69.3 (C-4^1), 67.4 (C-6^1), 66.9 (C-5^2), 65.8 (C-6^2), 55.7 (OMe (MP)), 46.6 (CH (Fmoc)).

HRMS ESI m/z calcd for $[M+Na]^+$ $C_{76}H_{66}O_{18}$ 1289.4141; found 1289.4154.

***p*-Methoxyphenyl 3,4-di-*O*-benzoyl-2-*O*-benzyl-6-*O*-fluorenylmethyloxycarbonyl-α-D-galactopyranosyl-(1→6)-3,4-di-*O*-benzoyl-2-*O*-benzyl-D-galactopyranose (32)**

Disaccharide **21** (3.16 g, 2.50 mmol) was dissolved in acetonitrile (160 mL), then water (40 mL) and benzene (6 mL) were added. CAN (6.85 g, 12.50 mmol) was added to the solution at 0 °C and the mixture was stirred for 10 minutes, diluted with EtOAc and washed with saturated solution of $NaHCO_3$. The aqueous phase was washed with EtOAc three times; the combined organic extracts were dried over anhydrous Na_2SO_4, filtered and concentrated under vacuum. Column chromatography of the residue (silica gel, toluene : EtOAc 20:1 → 4:1) provided yellowish syrup **32** (2.06 g, 71%) as a mixture of α- and β-isomers in ratio 1 : 1. R_f = 0.20 (toluene : EtOAc 5 : 1).

^{1}H NMR (300 Hz, $CDCl_3$): δ 8.08 – 7.93 (m, 4.5 H, Ph (Bz)), 7.91 – 7.72 (m, 6.5H, Ph (Bz), Fmoc), 7.70 – 7.08 (m, 42.5 H, Ph (Bn, Bz), Fmoc + $CHCl_3$), 5.95 – 5.72 (m, 3.5H, H-3^1 α, H-4^1 α, H-3^2 α, H-4^2 α, H-4^1 β, H-3^2 β, H-4^2 β), 5.55 (br s, 0.5H, H-1^1 α), 5.47 (dd, $J_{3,4}$ = 3.4 Hz, $J_{3,2}$ = 10.3 Hz, 0.5H, H-3^1 β), 5.05 (dd, $J_{1,2}$ = 7.6 Hz, $J_{1,OH}$ = 3.9 Hz, 0.5H, H-1^1 β), 4.48 (m, 1H, H-1^2 α, H-1^2 β), 4.93 (d, J = 11.7 Hz, 0.5H, Bn), 4.81 – 4.56 (m, 5H, H-5^1 α, H-5^2 β, 7 × Bn), 4.47 – 4.31 (m, 3.5H, H-6A^2 α, H-6A^2 β, OH β, CH_2 (Fmoc) α, β), 4.31 – 4.10 (m, 4H, H-2^1 α, H-2^2 α, H-2^2 β, H-5^1 β, H-6B^2 α, H-6B^2 β, CH (Fmoc) α, β), 3.96 – 3.77 (m, 2.5H, H-2^1 β, H-6A^1 α, H-6A^1 β, H-6B^1 α, H-6A^1 β), 3.61 (br s, 0.5H, OH α).

^{13}C{^{1}H} NMR (75 Hz, $CDCl_3$): δ 165.7, 165.6, 165.5, 165.5, 165.4, 165.3 (C=O (Bz)), 155.3, 155.0, 143.3 (*ipso*-Ph (Bz)), 141.2 (Fmoc), 138.0, 137.7, 137.6, 137.5, 136.5 (*ipso*-Ph (Bn)), 133.4, 133.4, 133.0 (*ipso*-Ph (Bz)),129.9, 129.8, 129.7, 129.6, 129.5, 129.1, 128.5, 128.5, 128.4, 128.3, 128.2, 128.1, 128.0, 127.9, 127.9, 127.4, 125.8, 125.4, 125.3 (Ph (Bn, Bz), Fmoc), 120.1, 120.0 (Fmoc), 98.4 (C-1^2 β), 98.3 (C-1^2 α), 97.8 (C-1^1 β), 91.8 (C-1^1 α), 77.3 (C-2^1 β), 74.5 (CH_2 (Bn)), 73.7 (C-2^1 α), 73.3 (C-2^2 α, C-2^2 β), 73.0 (CH_2 (Bn)), 72.9 (C-5^1 β), 72.8 (C-3^1 β), 70.6 (CH_2 (Bn), 70.4 (CH_2 (Fmoc) β), 70.3 (CH_2 (Fmoc) α), 70.2 (C-3^1 α), 70.1 (C-3^2 α, C-4^2 α), 69.7 (C-4^1 β), 69.3 (C-6^1 β), 69.1 (C-4^1 α), 68.9 (C-6^1 β), 68.1 (C-5^1 α), 66.6 (C-5^1 α), 66.4 (C-5^2 β), 65.6 (C-6^2 α), 65.4 (C-6^2 β), 46.6 (CH (Fmoc)).

HRMS ESI m/z calcd for $[M+Na]^+$ $C_{69}H_{60}O_{17}$ 1183.3725; found 1183.3723.

***O*-(3,4-di-*O*-benzoyl-2-*O*-benzyl-6-*O*-fluorenylmethyloxycarbonyl-α-D-galactopyranosyl-(1→6)-3,4-di-*O*-benzoyl-2-*O*-benzyl-D-galactopyranosyl) *N*-phenyltrifluoroacetimidate (33)**

To a solution of **32** (2.06 g, 1.77 mmol) in acetone (40 mL) *N*-phenyltrifluoroacetimidoyl chloride (340 μL, 2.12 mmol) and K_2CO_3 (366 mg, 2.66 mmol) were added. The reaction mixture was stirred overnight, then diluted with acetone and filtered through a pad of Celite. The filtrate was concentrated under vacuum and the dry residue was purified by column chromatography (Al_2O_3, toluene : EtOAc 60 : 1 → 30 : 1) to give yellowish oil **33** (1.44 g, 60%) as a mixture of α- and β-isomers in ratio 4 : 1. R_f = 0.52 (toluene : EtOAc 5 : 1).

^{1}H NMR (600 Hz, $CDCl_3$, 323 K): δ 8.04 (d, J = 7.1 Hz, Ph (Bz)), 7.96 (m, 2.4H, Ph (Bz)), 7.87 – 7.71 (m, 7.8H, Ph (Bn, Bz), Fmoc), 7.67 – 7.08 (m, 50H, Ph (Bn, Bz, PTFAI), Fmoc + $CHCl_3$ + toluene), 6.92 (m, 2H, Ph (PTFAI)), 6.06 – 5.95 (m, 1H, H-1^1 α, H-4^1 β), 5.92 (d, $J_{4,3}$ = 3.0 Hz, 0.8H, H-4^1 α), 5.88 (dd, $J_{3,4}$ = 3.4 Hz, $J_{3,2}$ = 10.4 Hz, 0.2H, H-3^1 β), 5.85 (d, $J_{4,3}$ = 2.7 Hz, 0.8H, H-4^2 α), 5.77 (dd, $J_{3,4}$ = 3.2 Hz, $J_{3,2}$ = 10.5 Hz, 0.2H, H-3^2 β), 5.70 (dd, $J_{3,4}$ = 3.4 Hz, $J_{3,2}$ = 10.5 Hz, 0.2H, H-3^2 α), 5.27 (br d, 0.8H, H-3^1 α), 5.04 (d, $J_{1,2}$ = 3.4 Hz, 0.2H, H-1^2 β), 5.02 (d, $J_{1,2}$ = 3.4 Hz, 0.2H, H-1^2 α), 4.84 (d, J = 11.4 Hz, 0.8H, Bn α), 4.76 – 4.61 (m, 2.4H, H-5^2 β, 2 × Bn α, 3 × Bn β), 4.58 (d, J = 12.1 Hz, 1H, Bn α, β), 4.53 (t, $J_{5,6A}$ = $J_{5,6B}$ = 6.2 Hz, 0.8H, H-5^2 α), 4.38 – 4.13 (m, 8.6H, H-2^1 α, H-2^1 β, H-2^2 β, H-5^1 α, H-5^1 β, H-6A^2 α, H-6B^2 α, CH, CH_2 (Fmoc) α, β), 4.12 (dd, $J_{2,3}$ = 10.5 Hz, $J_{2,1}$ = 3.4 Hz, 0.8H, H-2^2 α),

4.08 (m, 0.4H, H-6A[2] α, H-6B[2] β), 3.99 (dd, $J_{6A,6B}$ = 10.7 Hz, $J_{6A,5}$ = 5.5 Hz, 0.8H, H-6A[1] α), 3.95 (dd, $J_{6A,6B}$ = 10.9 Hz, $J_{6A,5}$ = 6.2Hz, 0.2H, H-6A[1] β), 3.79 (dd, $J_{6B,6A}$ = 10.7 Hz, $J_{6A,5}$ = 6.4 Hz, 0.8H, H-6B[1] α), 3.75 (dd, $J_{6B,6A}$ = 10.9 Hz, $J_{6A,5}$ = 5.7 Hz, 0.2H, H-6B[1] β).

$^{13}C\{^1H\}$ NMR (150 Hz, $CDCl_3$, 323 K): δ 165.6, 165.6, 165.2, 165.0 (C=O (Bz)), 155.0 (Fmoc), 143.6, 143.6, 143.3 (*ipso*-Ph (Bz)), 141.4 (Fmoc), 138.0, 137.9, 137.4, 136.6 (*ipso*-Ph (Bn)), 133.5, 133.4, 133.2, 133.0, 130.1, 130.0, 129.9, 129.8, 129.7, 129.6, 129.2, 129.0, 128.9, 128.7, 128.6, 128.5, 128.4, 128.3, 127.9, 127.7, 127.3, 127.2, 127.2, 125.9, 125.4, 124.9, 124.7 (Ph (Bn, Bz, PTFAI)), 120.2 (Fmoc β), 120.1 (Fmoc α, PTFAI β), 119.6 (PTFAI α), 98.5 (C-1[2] α, C-1[2] β), 97.6 (C-1[1] α), 75.9 (C-2[1] α), 75.1 (CH_2 (Bn)), 73.4 (C-2[2] α, C-2[1] β, C-2[2] β), 73.3 (C-3[1] α, CH_2 (Bn)), 73.1 (CH_2 (Bn)), 72.9 (CH_2 (Bn)), 70.4 (CH_2 (Fmoc)), 70.1 (C-3[2] α), 69.7 (C-4[2] α, C-4[1] β), 69.0 (C-4[1] α), 67.5 (C-5[2] α), 67.3 (C-6[1] α), 67.2 (C-6[1] β), 66.0 (C-6[2] α), 65.4 (C-6[2] β), 46.9 (CH (Fmoc)).

HRMS ESI m/z calcd for $[M+Na]^+$ $C_{77}H_{64}F_3NO_{17}$ 1354.4006; found 1354.4037.

VII. Synthesis of linear pentasaccharide 4

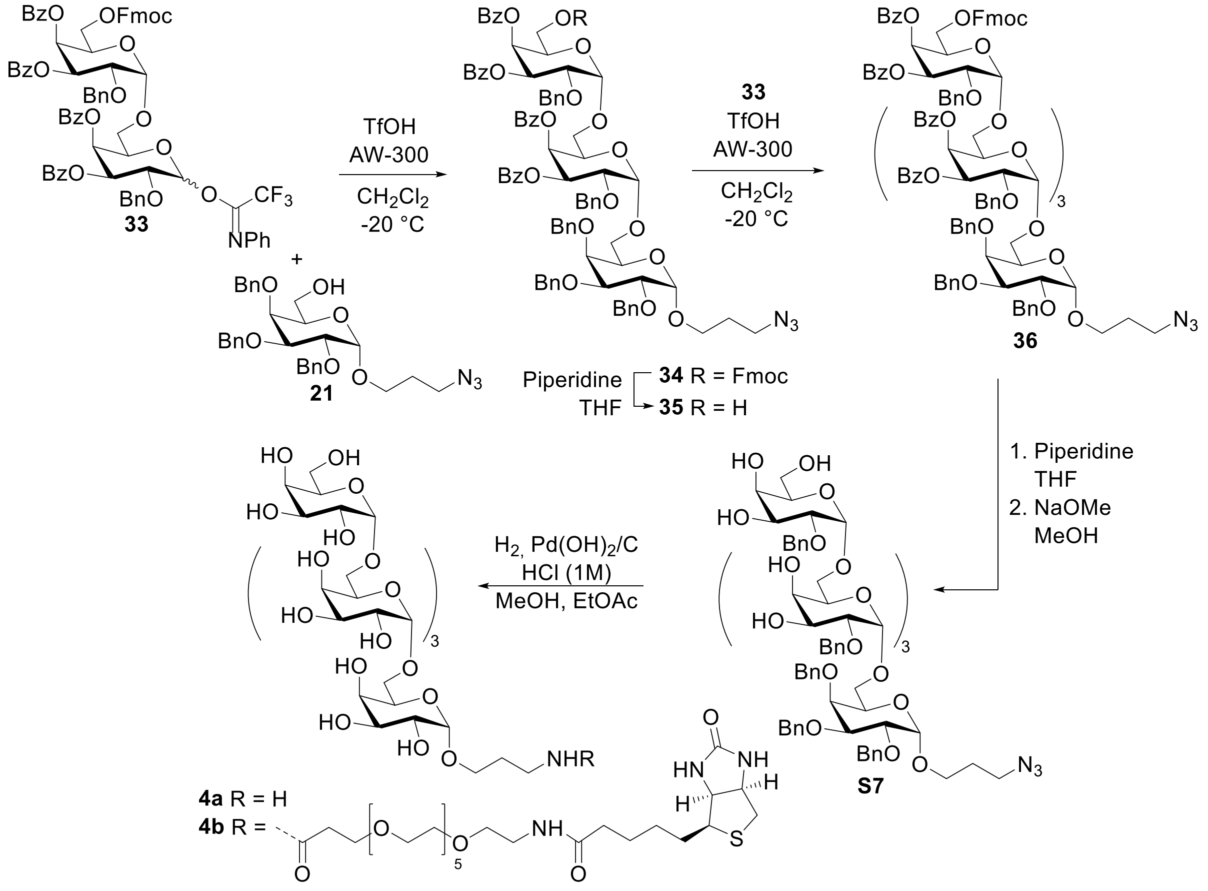

3-Azidopropyl 3,4-di-*O*-benzoyl-2-*O*-benzyl-6-*O*-fluorenylmethyloxycarbonyl-α-D-galactopyranosyl-(1→6)-3,4-di-*O*-benzoyl-2-*O*-benzyl- α-D-galactopyranosyl-(1→6)-2,3,4-tri-*O*-benzyl-α-D-galactopyranoside (34)

Molecular sieves AW-300 (885 mg) were added to a solution of donor **33** (520.0 mg, 0.390 mmol) and acceptor **21** (160.0 mg, 0.300 mmol) in dry CH_2Cl_2 (4.8 mL) under argon atmosphere at –20 °C. The mixture was stirred for 40 minutes and TfOH (16 µL, 0.180 mmol) was added. In 6 minutes the reaction mixture was diluted with CH_2Cl_2 and filtered through the pad of Celite. The filtrate was washed with

saturated solution of $NaHCO_3$ and the aqueous layer was extracted with CH_2Cl_2 three times. The combined organic extracts were dried over anhydrous Na_2SO_4, filtered and concentrated under vacuum. NMR spectra of the crude mixture indicated α- and β-isomers in ratio 20 : 1. Column chromatography (toluene : EtOAc 30 : 1 → 16 : 1) afforded α-disaccharide **34** (377.6 mg, 75%) as a white foam. R_f = 0.46 (toluene : EtOAc 5 : 1). $[\alpha]_D^{25}$ = 105.2.

^{1}H NMR (600 Hz, $CDCl_3$): δ 7.95 (d, J = 8.5 Hz, 2H, Ph (Bz)), 7.90 (d, J = 8.2 Hz, 2H, Ph (Bz)), 7.79 (d, J = 8.5 Hz, 2H, Ph (Bz)), 7.76 (d, J = 8.2 Hz, 2H, Ph (Bz)), 7.73 (d, J = 7.3 Hz, 2H, Fmoc)), 7.57 (td, J = 1.3 Hz, J = 7.6 Hz, 2H, Fmoc), 7.52 (m, 2H, Fmoc), 7.47 (m, 2H, Fmoc), 7.44 – 7.24 (m, 33H, Ph (Bn, Bz) + $CHCl_3$), 7.24 – 7.11 (m, 14H, Ph (Bz) + toluene), 5.92 (d, $J_{4,3}$ = 3.3 Hz, 1H, H-4^2), 5.83 (d, $J_{4,3}$ = 3.4 Hz, 1H, H-4^3), 5.80 (dd, $J_{3,4}$ = 3.3 Hz, $J_{3,2}$ = 10.4 Hz, 1H, H-3^2), 5.69 (dd, $J_{3,4}$ = 3.4 Hz, $J_{3,2}$ = 10.4 Hz, 1H, H-3^3), 5.01 (d, $J_{1,2}$ = 3.4 Hz, 1H, H-1^2), 4.99 (d, $J_{1,2}$ = 3.4 Hz, 1H, H-1^3), 4.98 (d, $J_{1,2}$ = 3.6 Hz, 1H, H-1^1), 4.93 (d, J = 11.7 Hz, 1H, Bn_1A), 4.86 (d, J= 11.7 Hz, 1H, Bn_2A), 4.80 (d, J = 11.9 Hz, 1H, Bn_3A), 4.75 (d, J = 11.6 Hz, 1H, Bn_3B), 4.72 (d, J = 11.9 Hz, 1H, Bn_3B), 4.69 (d, J = 12.0, 1H, Bn_5A), 4.60 (d, J = 11.7 Hz, 1H, Bn_1B), 4.59 – 5.50 (m, 5H, H-5^2, H-5^3, Bn_4A, Bn_4B, Bn_5B), 4.30 (dd, $J_{6A,6B}$ = 11.3 Hz, $J_{6A,5}$ = 7.0 Hz, 1H, H-6A^3), 4.25 (m, 2H, CH_2 (Fmoc)), 4.19 (dd, $J_{6B,6A}$ = 11.3 Hz, $J_{6B,5}$ = 5.3 Hz, 1H, H-6B^3), 4.16 – 4.05 (m, 5H, H-2^1, H-2^2, H-2^3, H-5^1, CH (Fmoc)), 4.03 (m, 1H, H-4^1), 4.01 (dd, $J_{3,4}$ = 2.8 Hz, $J_{3,2}$ = 10.0 Hz, 1H, H-3^1), 3.96 (dd, $J_{6A,6B}$ = 9.7 Hz, $J_{6A,5}$ = 6.8 Hz, 1H, H-6A^1), 3.87 (m, 2H, H-6A^2, OC*H*H'$CH_2CH_2N_3$), 3.68 (dd, $J_{6B,6A}$ = 10.5 Hz, $J_{6B,5}$ = 6.9 Hz, 1H, H-6B^2), 3.57 (m, 2H, OCH*H*'$CH_2CH_2N_3$, H-6B^1), 3.41 (m, 2H, $OCH_2CH_2C\mathit{H}_2N_3$), 1.90 (m, 2H, $OCH_2C\mathit{H}_2CH_2N_3$).

^{13}C{^{1}H} NMR (150 Hz, $CDCl_3$): δ 165.6, 165.4, 162.2, 165.2 (C=O (Bz)), 154.8, 143.4, 143.4 (*ipso*-Ph (Bz)), 141.2 (Fmoc), 138.9, 138.8, 137.6, 133.3, 133.3, 132.9, 129.8, 129.7, 129.6, 129.6, 129.5, 128.5, 128.5, 128.3, 128.2, 127.9, 127.9, 127.8, 127.5, 127.4, 127.2, 125.3 (Ph (Bn, Bz), Fmoc), 119.9 (Fmoc), 97.9 (C-1^3), 97.7 (C-1^1, C-1^2), 79.0 (C-3^1), 76.7 (C-2^1), 75.2 (C-4^1), 74.5 (CH_2 (Bn_1)), 73.6 (C-2^2), 73.2 (CH_2 (Bn_2, Bn_3)), 73.1 (CH_2 (Bn_4)), 73.0 (C-2^3), 72.6 (CH_2 (Bn_5)), 70.5 (C-3^2), 70.1 (CH_2 (Fmoc)), 70.0 (C-4^2), 69.8 (C-3^3), 69.6 (C-4^3), 69.4 (C-5^1), 67.9 (C-5^2), 67.5 (C-6^1), 67.1 (C-5^3), 66.7 (C-6^2), 65.9 (C-6^3), 65.2 ($O\mathit{C}H_2CH_2CH_2N_3$), 48.5 ($OCH_2CH_2\mathit{C}H_2N_3$), 46.6 (CH (Fmoc)), 28.8 ($OCH_2\mathit{C}H_2CH_2N_3$).

HRMS ESI m/z calcd for $[M+Na]^+$ $C_{99}H_{93}N_3O_{22}$ 1698.6143; found 1698.6173.

3-Azidopropyl 3,4-di-*O*-benzoyl-2-*O*-benzyl-α-D-galactopyranosyl-(1→6)-3,4-di-*O*-benzoyl-2-*O*-benzyl- α-D-galactopyranosyl-(1→6)-2,3,4-tri-*O*-benzyl-α-D-galactopyranoside (35)

Piperidine (208 μL, 2.13 mmol) was added to a solution of trisaccharide **34** (357.6 mg, 0.213 mmol) in anhydroud THF (6 mL) at 0 °C. In 20 minutes the reaction was completed, the mixture was diluted with toluene, and the solvents were evaporated under vacuum. Column chromatography of the dry residue (silica gel, toluene : EtOAc 30 : 1 → 4 : 1) provided trisaccharide **35** as a white foam (293.3 mg, 95%). R_f = 0.38 (toluene : EtOAc 4 : 1). $[\alpha]_D^{25}$ = 118.9.

^{1}H NMR (600 Hz, $CDCl_3$): δ 8.00 (d, J = 7.8 Hz, 2H, Ph (Bz)), 7.90 (d, J = 7.8 Hz, 2H, Ph (Bz)), 7.81 (d, J = 7.8 Hz, 2H, Ph (Bz)), 7.79 (d, J = 7.8 Hz, 2H, Ph (Bz)), 7.63 (t, J = 7.3 Hz, 2H, Ph (Bz)), 7.59 (t, J = 7.3 Hz, 2H, Ph (Bz)), 7.51 – 7.46 (m, 5H, Ph (Bz)), 7.45 – 7.37 (m, 7H, Ph (Bn, Bz)), 7.38 – 7.14 (m, 33H, Ph (Bn, Bz) + $CHCl_3$), 5.93 (d, $J_{4,3}$ = 3.0 Hz, 1H, H-4^2), 5.79 (dd, $J_{3,4}$ = 3.0 Hz, $J_{3,2}$ = 10.4 Hz, 1H, H-3^2), 5.76 (d, $J_{4,3}$ = 3.1 Hz, 1H, H-4^3), 5.69 (dd, $J_{3,4}$ = 3.1 Hz, $J_{3,2}$ = 10.5 Hz, 1H, H-3^3), 5.07 (d, $J_{1,2}$ = 3.5 Hz, 1H, H-1^1), 4.98 (d, $J_{1,2}$ = 3.2 Hz, 1H, H-1^2), 4.97 – 4.93 (m, 2H, H-1^3, Bn_1A), 4.89 (d, J = 11.6 Hz, 1H, Bn_2A), 4.82 (d, J = 11.9 Hz, 1H, Bn_3A), 4.77 (m, 2H, Bn_2B, Bn_3B), 4.68 (d, J = 12.2 Hz, 1H, Bn_4A), 4.65 – 4.54 (m, 5H, H-5^2, Bn_1B, Bn_4B, Bn_5A, Bn_5B), 4.34 (t, $J_{5,6A}$ = $J_{5,6B}$ = 6.7 Hz, 1H, H-

5^3), 4.16 – 4.07 (m, 4H, H-2^1, H-2^2, H-2^3, H-5^1), 4.04 – 3.99 (m, 2H, H-3^1, H-4^1), 3.94 (dd, $J_{6A,6B}$ = 9.2 Hz, $J_{6A,5}$ = 7.2 Hz, 1H, H-6A^1), 3.91 (m, 1H, OC*H*H'CH$_2$CH$_2$N$_3$), 3.79 (dd, $J_{6A,6B}$ = 10.6 Hz, $J_{6A,5}$ = 5.3 Hz, 1H, H-6A^2), 3.72 (dd, $J_{6B,6A}$ = 10.6 Hz, $J_{6B,5}$ = 6.9 Hz, 1H, H-6B^2), 3.67 – 3.58 (m, 2H, H-6A^3, OCH*H'*CH$_2$CH$_2$N$_3$), 3.56 – 3.48 (m, 2H, H-6B^1, H-6B^3), 3.45 (m, 2H, OCH$_2$CH$_2$C*H*$_2$N$_3$), 2.68 (br t, 1H, OH), 1.97 (m, 2H, OCH$_2$C*H*$_2$CH$_2$N$_3$).

^{13}C{^{1}H} NMR (150 Hz, CDCl$_3$): δ 166.2, 165.5, 165.3, 165.2 (C=O (Bz)), 138.8, 138.6, 137.7, 137.6 (*ipso*-Ph (Bn)), 133.4, 132.9, 129.8, 129.7, 129.6, 129.5, 129.3, 128.5, 128.4, 128.3, 128.2, 128.2, 128.1, 127.9, 127.9, 127.8, 127.8, 127.5, 127.4 (Ph (Bn, Bz)), 97.9 (C-1^3), 97.6 (C-1^2), 97.5 (C-1^1), 78.9 (C-3^1), 76.7 (C-2^1), 75.2 (C-4^1), 74.5 (CH$_2$ (Bn$_2$)), 73.4 (C-2^2), 73.3 (CH$_2$ (Bn$_2$)), 73.2 (C-2^3), 73.1 (CH$_2$ (Bn$_3$)), 73.1 (CH$_2$ (Bn$_4$)), 72.6 (CH$_2$ (Bn$_5$)), 70.5 (C-3^2), 70.1 (C-4^3), 69.9 (C-3^3), 69.8 (C-4^2), 69.6 (C-5^3), 69.3 (C-5^1), 68.0 (C-5^2), 67.6 (C-6^1), 66.9 (C-6^2), 65.2 (O*C*H$_2$CH$_2$CH$_2$N$_3$), 60.8 (C-6^3), 48.5 (OCH$_2$CH$_2$*C*H$_2$N$_3$), 28.8 (OCH$_2$*C*H$_2$CH$_2$N$_3$).

HRMS ESI m/z calcd for [M+Na]$^+$ $C_{84}H_{83}N_3O_{20}$ 1476.5462; found 1476.5469.

3-Azidopropyl 3,4-di-*O*-benzoyl-2-*O*-benzyl-6-*O*-fluorenylmethyloxycarbonyl-α-D-galactopyranosyl-(1→6)-3,4-di-*O*-benzoyl-2-*O*-benzyl- α-D-galactopyranosyl-(1→6)- 3,4-di-*O*-benzoyl-2-*O*-benzyl- α-D-galactopyranosyl-(1→6)-3,4-di-*O*-benzoyl-2-*O*-benzyl- α-D-galactopyranosyl-(1→6)-2,3,4-tri-*O*-benzyl-α-D-galactopyranoside (36)

Molecular sieves AW-300 (30 mg) were added to a solution of disaccharide donor **33** (30.0 mg, 0.023 mmol) and trisaccharide acceptor **35** (27.3 mg, 0.019 mmol) in dry CH$_2$Cl$_2$ (600 μL) under argon atmosphere at –5 °C. The mixture was stirred for 40 minutes and TBDMSOTf (2.6 μL, 0.011 mmol) was added. In 2.5 h the reaction mixture was diluted with CH$_2$Cl$_2$ and filtered through the pad of Celite. The filtrate was washed with saturated solution of NaHCO$_3$ and the aqueous layer was extracted with CH$_2$Cl$_2$ three times. The combined organic extracts were dried over anhydrous Na$_2$SO$_4$, filtered and concentrated under vacuum. The residue was purified by column chromatography (toluene : EtOAc 30 : 1 → 15 : 1) to give pentasaccharide **36** (32.0 mg, 55%). R_f = 0.20 (toluene : EtOAc 15 : 1). $[\alpha]_D^{22}$ = 159.2.

^{1}H NMR (600 Hz, CDCl$_3$): δ 8.08 (d, *J* = 7.5 Hz, 2H, Ph (Bz)), 8.03 (d, *J* = 7.5 Hz, 2H, Ph (Bz)), 7.93 (d, *J* = 7.5 Hz, 2H, Ph (Bz)), 7.88 (d, *J* = 7.5 Hz, 2H, Ph (Bz)), 7.76 (d, *J* = 7.5 Hz, 2H, Ph (Bz)), 7.74 – 7.70 (m, 9H, Ph (Bz), Fmoc)), 7.57 (t, *J* = 7.5 Hz, 1H, Ph (Bz)), 7.53 (t, *J* = 7.5 Hz, 1H, Ph (Bz)), 7.50 (t, *J* = 7.5 Hz, 1H, Ph (Bz)), 7.49 – 7.26 (m, 32H, Ph (Bn, Bz), Fmoc) + CHCl$_3$), 7.25 – 7.19 (m, 10H, Ph (Bn, Bz)), 7.17 – 7.00 (m, 26H, Ph (Bz) + toluene), 5.94 (d, $J_{4,3}$ = 3.6 Hz, 1H, H-4^4), 5.91 (d, $J_{4,3}$ = 3.7 Hz, 1H, H-4^2), 5.89 (d, $J_{4,3}$ = 3.3 Hz, 1H, H-4^3), 5.84 – 5.80 (m, 2H, H-3^4, H-4^5), 5.78 (dd, $J_{3,4}$ = 3.7 Hz, $J_{3,2}$ = 10.4 Hz, 1H, H-3^2), 5.72 (dd, $J_{3,4}$ = 3.3 Hz, $J_{3,2}$ = 10.4 Hz, 1H, H-3^3), 5.70 (dd, $J_{3,4}$ = 3.5 Hz, $J_{3,2}$ = 10.4 Hz, 1H, H-3^5), 5.44 (d, $J_{1,2}$ = 3.6 Hz, 1H, H-1^4), 5.24 (d, $J_{1,2}$ = 3.4 Hz, 1H, H-1^3), 5.02 (d, $J_{1,2}$ = 3.7 Hz, 1H, H-1^1), 4.99 (d, $J_{1,2}$ = 3.3 Hz, 1H, H-1^2), 4.88 (d, *J* = 11.5 Hz, 1H, Bn$_1$A), 4.82 (d, *J* = 11.6 Hz, 1H, Bn$_3$A), 4.79 (d, *J* = 11.8 Hz, 1H, Bn$_2$A), 4.76 – 4.58 (m, 10H, H-1^5, H-5^2, H-5^3, H-5^4, Bn$_1$B, Bn$_2$B, Bn$_3$B, Bn$_4$A, Bn$_4$B, Bn$_5$A), 4.57 – 4.50 (m, 4H, H-5^5, Bn$_5$B, Bn$_6$A, Bn$_7$A), 4.41 (d, *J* = 11.8 Hz, 1H, Bn$_6$B), 4.38 (d, *J* = 12.3 Hz, 1H, Bn$_7$B), 4.33 (dd, $J_{2,3}$ = 10.4 Hz, $J_{2,1}$ = 3.6 Hz, 1H, H-2^4), 4.25 (dd, $J_{2,3}$ = 10.4 Hz, $J_{2,1}$ = 3.4 Hz, 1H, H-2^3), 4.22 – 4.00 (m, 13H, H-2^2, H-2^1, H-3^1, H-4^1, H-5^1, H-6A^1, H-2^5, H-6A^5, H-6B^5, H-6A^4), 3.90 – 3.84 (m, 2H, H-6A^3, OC*H*H'CH$_2$CH$_2$N$_3$), 3.82 – 3.76 (m, 3H, H-6B^1, H-6B^3, H-6B^4), 3.75 (dd, $J_{6A,6B}$ = 10.5 Hz, $J_{6A,5}$ = 6.8 Hz, 1H, H-6A^2), 3.56 (m, 1H, OCH*H'*CH$_2$CH$_2$N$_3$), 3.45 (dd, $J_{6B,6A}$ = 10.5 Hz, $J_{6B,5}$ = 5.1 Hz, 1H, H-6B^2), 3.38 (t, *J* = 7.1 Hz, 2H, OCH$_2$CH$_2$C*H*$_2$N$_3$), 1.88 (m, 2H, OCH$_2$C*H*$_2$CH$_2$N$_3$).

$^{13}C\{^1H\}$ NMR (150 Hz, $CDCl_3$): δ 165.8, 165.7, 165.5, 165.2, 165.1, 165.0 (C=O (Bz)), 154.7, 143.3 (*ipso*-Ph (Bz)), 141.1 (Fmoc), 139.1, 138.0, 137.8, 137.5 (*ipso*-Ph (Bn)), 133.4, 133.2, 132.9, 132.8, 132.6, 130.0, 129.9, 129.8, 129.6, 128.6, 128.5, 128.3, 128.2, 128.1, 128.0, 127.9, 127.8, 127.6, 127.4, 127.3, 127.2, 127.1, 125.2 (Ph (Bn, Bz), Fmoc), 119.9 (Fmoc)), 97.8 ($C-1^2$), 97.6 ($C-1^1$), 97.6 ($C-1^3$, $C-1^4$), 97.1 ($C-1^5$), 79.0 ($C-3^1$), 76.8 ($C-2^1$), 75.1 ($C-4^1$), 74.7 (CH_2 (Bn_1)), 74.1 ($C-2^4$), 74.0 ($C-2^3$), 73.5 ($C-2^2$), 73.1 (CH_2 (Bn_2)), 73.0 (CH_2 (Bn_3)), 72.9 ($C-2^5$, CH_2 (Bn_4)), 72.5 (CH_2 (Bn_5, Bn_6)), 72.1 (CH_2 (Bn_7)), 70.7 ($C-3^4$), 70.6 ($C-4^3$, $C-4^4$), 70.3 ($C-3^2$, $C-4^2$), 70.0 ($C-3^3$, CH_2 (Fmoc)), 69.9 ($C-3^5$), 69.5 ($C-4^5$), 69.2 ($C-5^1$), 68.9 ($C-5^3$), 68.7 ($C-6^3$), 68.5 ($C-5^4$), 68.2 ($C-6^4$), 67.5 ($C-5^2$), 67.0 ($C-6^1$), 66.9 ($C-6^2$), 66.5 ($C-5^5$), 65.9 ($C-6^5$), 65.1 ($OCH_2CH_2CH_2N_3$), 48.6 ($OCH_2CH_2CH_2N_3$), 46.6 (CH (Fmoc)), 28.8 ($OCH_2CH_2CH_2N_3$).

HRMS ESI m/z calcd for $[M+NH_4]^+$ $C_{146}H_{134}N_3O_{35}$ 2613.9633; found 2613.9601.

3-Azidopropyl 2-*O*-benzyl-α-D-galactopyranosyl-(1→6)-2-*O*-benzyl-α-D-galactopyranosyl-(1→6)-2-*O*-benzyl-α-D-galactopyranosyl-(1→6)-2-*O*-benzyl-α-D-galactopyranosyl-(1→6)-2,3,4-tri-*O*-benzyl-α-D-galactopyranoside (S7)

Piperidine (8.3 μL, 0.085 mmol) was added to a solution of pentasaccharide **36** (22.0 mg, 0.0085 mmol) in anhydroud THF (300 μL) at 0 °C. In 35 minutes the reaction was completed, the mixture was diluted with toluene, and the solvents were evaporated. The crude product was dissolved in a mixture of anhydrous MeOH (200 μL) and anhydrous CH_2Cl_2 (100 μL) and NaOMe (2.3 μL, 1M solution in MeOH) was added. The mixture was stirred for 48 hours, then quenched with AcOH (1.5 μL) to neutral pH of the solution. The solvents were removed under vacuum. Column chromatography of the dry residue (silica gel, $CHCl_3$: MeOH 10 : 1 → 2.5 : 1) gave pentasaccharide **S7** (12.7 mg, 98%) as a colorless syrup. R_f = 0.10 ($CHCl_3$: MeOH 10 : 1). $[\alpha]_D^{24}$ = 86.2.

^1H NMR (600 Hz, $CDCl_3$): δ 7.36 – 7.23 (m, 44H, Ph (Bn) + $CHCl_3$), 4.93 – 4.89 (m, 3H, 2 × H-1, Bn), 4.86 (d, $J_{1,2}$ = 3.4 Hz, 1H, H-1), 4.81 (d, J = 11.7 Hz, 1H, Bn), 4.79 – 4.75 (m, 2H, $H-1^1$, Bn), 4.73 (d, $J_{1,2}$ = 3.6 Hz, 1H, H-1), 4.69 (d, J = 11.5 Hz, 1H, Bn), 4.65 – 4.58 (m, 8H, 8 × Bn), 4.54 (d, J = 12.1 Hz, 1H, Bn), 4.51 (d, J = 12.1 Hz, 1H, Bn), 4.04 – 3.85 (m, 15H, $H-2^1$, $H-3^1$, $H-4^1$, $H-5^1$, 4 × H-3, 4 × H-4, 3 × H-5), 3.85 – 3.77 (m, 5H, $H-5^5$, $H-6A^5$, $H-6B^5$, 3 × H-6A), 3.75 (m, 1H, $H-6B^5$), 3.71 – 3.60 (m, 9H, $H-6A^1$, 4 × H-2, 3 × H-6B, OC*H*H'$CH_2CH_2N_3$), 3.48 (br s, 0.5H, OH), 3.43 (m, 1H, OCH*H'*$CH_2CH_2N_3$), 3.36 – 3.31 (m, 3H, $H-6B^1$, $OCH_2CH_2CH_2N_3$), 3.17 (br s, 0.5H, OH), 3.04 (br s, 0.5H, OH), 2.93 (br s, 0.5H, OH), 2.89 (br s, 0.5H, OH), 2.78 (br s, 0.5H, OH), 2.67 (br s, 0.5H, OH), 2.58 (br s, 0.5H, OH), 1.81 (m, 2H, $OCH_2CH_2CH_2N_3$).

$^{13}C\{^1H\}$ NMR (150 Hz, $CDCl_3$): δ 128.6, 128.5, 128.4, 128.3, 128.2, 128.0, 127.9, 127.8, 127.7, 127.6, 127.5 (Ph (Bn)), 97.8 ($C-1^1$, C-1), 97.6, 97.5, 97.4 (3 × C-1), 78.9 ($C-3^1$), 76.6 (4 × C-2), 76.5 ($C-2^1$), 75.3 ($C-4^1$), 74.6 (CH_2 (Bn_1)), 73.4 (CH_2 (Bn_2)), 73.2 (CH_2 (Bn_3)), 72.9 (4 × CH_2 (Bn)), 70.4, 69.8 (4 × C-4), 69.6 ($C-5^5$), 69.5 ($C-5^1$, 3 × C-3), 69.0 (C-3), 68.6, 68.2 (3 × C-5), 67.8, 67.7 (3 × C-6), 67.2 ($C-6^1$), 65.0 ($OCH_2CH_2CH_2N_3$), 62.9 ($C-6^5$), 48.4 ($OCH_2CH_2CH_2N_3$), 28.8 ($OCH_2CH_2CH_2N_3$).

HRMS ESI m/z calcd for $[M+NH_4^+]^+$ $C_{82}H_{99}N_3O_{26}$ 1564.6409; found 1564.6406.

3-Aminopropyl α-D-galactopyranosyl-(1→6)-α-D-galactopyranosyl-(1→6)-α-D-galactopyranosyl-(1→6)-α-D-galactopyranosyl-(1→6)- α-D-galactopyranoside (4a)

$Pd(OH)_2/C$ (20 %wt, 6 mg) and 1M aq. solution of HCl (19 μL) were added to added to a solution of pentasaccharide **S7** (12.6 mg, 0.008 mmol) in a mixture of anhydrous EtOAc (570 μL) and anhydrous

MeOH (1.12 mL). The reaction mixture was intensively stirred under a hydrogen atmosphere for 5 hours. Then, the mixture was diluted with MeOH and water, the catalyst was filtered and the filtrate was concentrated under vacuum. Pentasaccharide **4a** was purified by gel-permeation chromatography on the TSK HW-40(S) column and isolated as a fluffy solid (4.3 mg, 60%). $R_f = 0.10$ ($CHCl_3$: MeOH 20 : 1). $[\alpha]_D^{20} = 95.1$ (H_2O).

^{1}H NMR (600 Hz, D_2O, 303 K): δ 4.96 (d, $J_{1,2} = 3.5$ Hz, 2H, H-1^{5}, H-1), 4.95 (d, $J_{1,2} = 3.5$ Hz, 1H, H-1), 4.93 (br s, 2H, H-1^{1}, H-1), 4.20 – 4.11 (m, 3H, H-5^{2}, H-5^{3}, H-5^{4}), 4.08 (m, 1H, H-5^{1}), 4.01 – 3.94 (m, 6H, 5 × H-4, H-5),^{5}3.91 – 3.76 (m, 15H, 5 × H-2, 5 × H-3, H-6A , H-6A^{1} , H-6A^{2} , H-6A^{3}, ^{4} OC*H*H'$CH_2CH_2N_3$), 3.73 – 3.61 (m, 6H, H-6A^{5}, 5 × H-6B), 3.58 (m, 1H, OCH*H*'$CH_2CH_2NH_2$), 3.10 (m, 2H, $OCH_2CH_2C\mathit{H}_2NH_2$), 1.97 (m, 2H, $OCH_2C\mathit{H}_2CH_2NH_2$).

^{13}C{^{1}H} NMR (150 Hz, D_2O, 303 K): δ 99.1 (C-1^{1}), 98.6, 98.5 (2 × C-1), 98.4 (C-1^{5}, C-1), 71.6 (C-5^{5}), 70.5 (5 × C-3, C-4^{5}), 69.9, 69.8 (4 × C-4), 69.5 (C-5), ^{1}69.4, 69.2 (3 × C-5), 68.8, 68.7, 68.6 (5 × C-2), 67.1 (4 × C-6), 66.7 ($O\mathit{C}H_2CH_2CH_2NH_2$), 61.7 (C-6^{5}), 38.5 ($OCH_2CH_2\mathit{C}H_2NH_2$), 27. (4$OCH_2\mathit{C}H_2CH_2NH_2$).

HRMS ESI m/z calcd for $[M+Na]^+$ $C_{33}H_{59}NO_2$ 908.3218; found 908.3217.

Conjugate of the pentasaccharide with biotin (4b)

A solution of activated ester of biotin in DMF[5] (62 μmol/ml, 32.0 μL 0.0020 mmol) and dry Et_3N (2.0 μL) was added to a solution of pentasaccharide **4a** (1.5 mg, 0.0017 mmol) in DMF (330 μL). The mixture was stirred for 12 hour and then loaded onto the TSK HW-40(S) column and washed with a 0.1 M solution of AcOH. The fractions with the product were combined and dried by lyophilization to afford **4b** (2.3 mg, 92%). $R_f = 0.33$ (BPS : AMW 1 : 1).

Characteristic signals in ^{1}H NMR (600 Hz, D_2O, 303 K): δ 5.02 – 4.96 (m, 4H, H-1^{2}, H-1^{3}, H-1^{4}, H-1^{5}), 4.94 (d, $J_{1,2} = 3.7$ Hz, 1H, H-1^{1}), 4.62 (m, 1H, H-6a biotin), 4.44 (m, 1H, H-3a biotin), 4.23 – 4.16 (m, 3H, H-5^{2}, H-5^{3}, H-5^{4}), 4.10 (m, 1H, H-5^{1}), 3.72 (t, $J = 6.0$ Hz, 3H, $O\mathit{CH}_2CH_2O$ biotin), 3.64 (t, $J = 6.0$ Hz, 3H, $C\mathit{H}_2O$ biotin), 3.54 (m, 1H, OCH*H*'$CH_2CH_2NH_2$), 3.41 (t, $J = 5.2$ Hz, 2H, $C\mathit{H}_2NH$ biotin), 3.33 (m, 3H, biotin, $OCH_2CH_2C\mathit{H}_2NH_2$), 2.96 (dd, $J = 5.1$ Hz, $J = 13.0$ Hz, 1H, H-6A biotin), 2.80 (d, $J = 13.0$ Hz, 1H, H-6B biotin), 2.54 (t, $J = 6.0$ Hz, 2H, $C(O)CH_2C\mathit{H}_2O$ biotin), 2.29 (t, $J = 7.1$ Hz, 2H, Hα biotin), 1.90 – 1.56 (m, 7H, HΔ-A biotin, HΔ-B biotin, H-β biotin, H-γ biotin, $OCH_2C\mathit{H}_2CH_2NH_2$).

Characteristic signals in ^{13}C{^{1}H} NMR (150 Hz, D_2O, 303 K): δ 99.2 (C-1^{1}), 98.9, 98.8, 98.7 (C-1^{2}, C-1^{3}, C-1^{4}, C-1^{5}), 70.2 (C-5^{1}, C-5^{2}, C-5^{3}, C5^{4}), 69.7 ($\mathit{C}H_2O$ biotin), 67.7 ($O\mathit{C}H_2CH_2O$ biotin), 66.7 ($O\mathit{C}H_2CH_2CH_2NH_2$), 63.4 (C3-a biotin), 61.6 (C6-a biotin), 56.1 (C-4 biotin), 40.5 (C-6 biotin), 39.8 ($\mathit{C}H_2NH$ biotin), 37.7 ($OCH_2CH_2\mathit{C}H_2NH_2$), 37.0 ($C(O)\mathit{C}H_2CH_2O$ biotin), 36.3 (C-α biotin), 29.1 ($OCH_2\mathit{C}H_2CH_2NH_2$), 28.5 (C-γ biotin), 28.8 (CΔ-A, CΔ-B biotin), 25.9 (C-β biotin).

HRMS ESI m/z calcd for $[M+Na]^+$ $C_{58}H_{102}N_4O_{35}S$ 1469.5938; found 1469.5926.

VIII. Synthesis of hexasaccharide 5

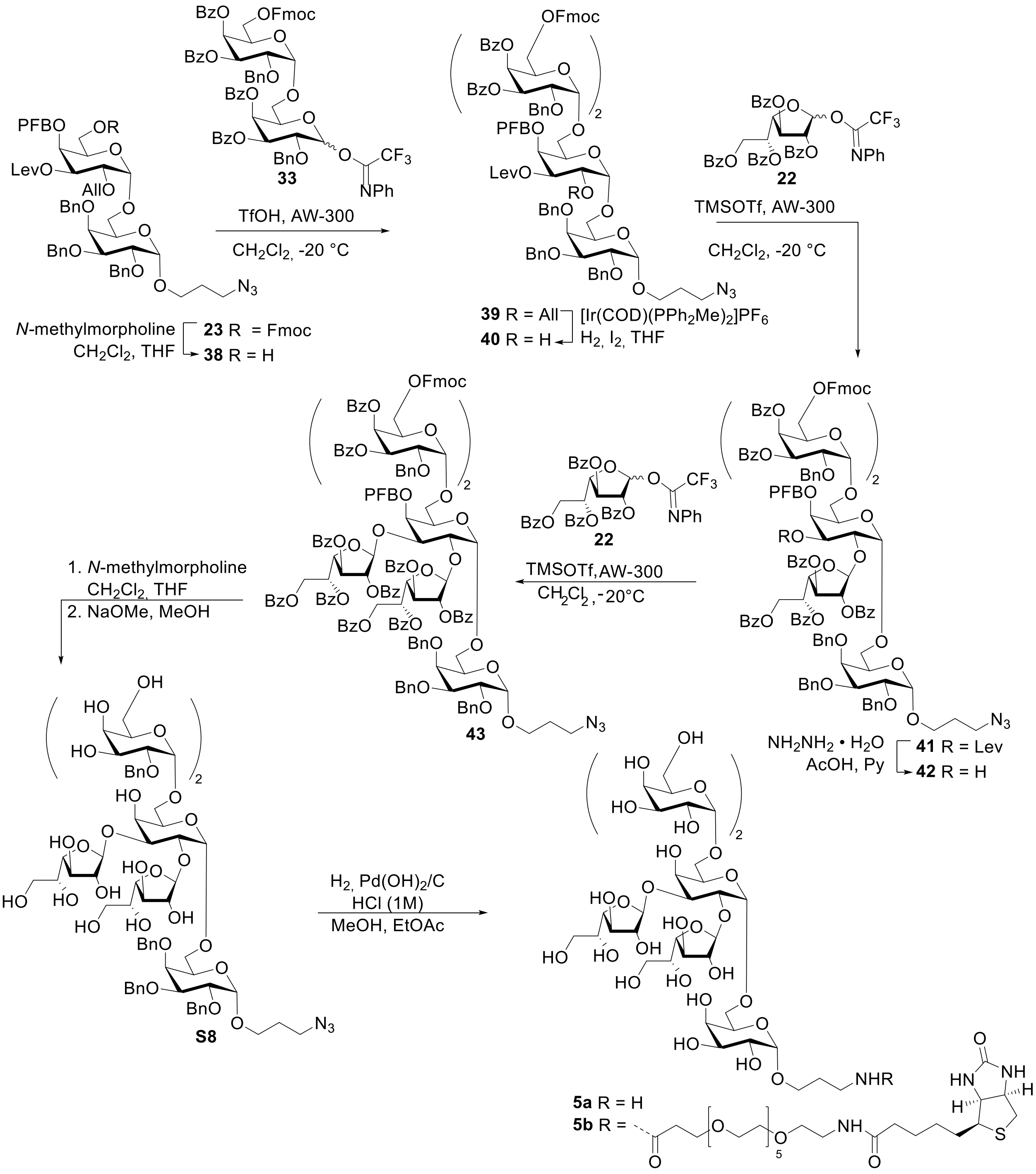

3-Azidopropyl 2-*O*-allyl-3-*O*-levulinyl-4-*O*-pentafluorobenzoyl-α-D-galactopyranosyl-(1→6)-2,3,4-tri-*O*-benzyl-α-D-galactopyranoside (38)

To a solution of **23** (54.4 mg, 0.044 mmol) in a mixture of dry THF (280 μL) and dry CH_2Cl_2 (200 μL) *N*-methylmorpholine (29 μL, 0.26 mmol) was added. After 19 hours TLC (toluene : EtOAc 4 : 1) indicated that the reaction had completed. The base was quenched with 0.1 M aqueous solution of H_2SO_4 (100 μL) and the mixture was washed with brine. The aqueous phase was washed with $CHCl_3$ three times; the combined organic extracts were dried over anhydrous Na_2SO_4, filtered and concentrated

under vacuum. Column chromatography of the dry residue (silica gel, toluene : EtOAc 10 : 1 → 4 : 1) gave **38** (35.0 mg, 78%) as a colorless oil. R_f = 0.22 (toluene : EtOAc 4 : 1). $[\alpha]_D^{23}$ = 88.1.

^{1}H NMR (300 Hz, $CDCl_3$): δ 7.44 – 7.22 (m, 17H, Ph (Bn) + $CHCl_3$), 5.80 (m, 1H, CH_2CH=CH_2), 5.69 (d, $J_{4,3}$ = 3.0 Hz, 1H, H-4^2), 5.36 (dd, $J_{3,4}$ = 3.0 Hz, $J_{3,2}$ = 10.4 Hz, 1H, H-3^2), 5.22 (dd, J = 1.4 Hz, J = 17.3 Hz, 1H, CH_2CH=C*H*H'), 5.15 (d, J = 10.4 Hz, 1H, CH_2CH=CH*H'*), 4.97 (d, J = 11.6 Hz, 1H, Bn_1A), 4.91 – 4.72 (m, 5H, H-1^1, H-1^2, Bn_2A, Bn_3A, Bn_3B), 4.66 (d, J = 12.0 Hz, 1H, Bn_2B), 4.59 (d, J = 11.6 Hz, 1H, Bn_1B), 4.20 (m, 2H, H-5^2, C*H*H'CH=CH_2), 4.08 – 4.00 (m, 2H, H-2^1, CH*H'*CH=CH_2), 3.95 (m, 3H, H-3^1, H-4^1, H-5^1), 3.85 – 3.65 (m, 4H, H-2^2, H-6A^1, H-6A^2, OC*H*H'$CH_2CH_2N_3$), 3.63 – 3.37 (m, 5H, H-6B^1, H-6B^2, OCH*H'*$CH_2CH_2N_3$, $OCH_2CH_2CH_2N_3$), 2.88 – 2.42 (m, 4H, 2 × CH_2 (Lev)), 2.16 (s, 3H, Me (Lev)), 1.91 (m, 2H, $OCH_2CH_2CH_2N_3$).

^{13}C{^{1}H} NMR (75 Hz, $CDCl_3$): δ 172.0 (C=O (Lev), 138.8, 138.7, 138.6 (*ipso*-Ph (Bn)), 134.4(CH_2CH=CH_2), 129.0, 128.4, 128.3, 128.1, 127.9, 127.7, 127.7, 127.6, 127.5 (Ph (Bn)), 117.7 (CH_2CH=CH_2), 97.9 (C-1^1, C-1^2), 78.9 (C-3^1), 76.6 (C-2^1), 75.1 (C-4^1), 74.6 (CH_2 (Bn_1)), 73.5 (CH_2 (Bn_2)), 73.4 (H-2^2, CH_2 (Bn_3)), 72.7 (CH_2CH=CH_2), 71.9 (C-4^2), 70.0 (C-3^2), 69.3 (C-5^1), 68.6 (C-5^2), 67.4 (C-6^1), 65.1 ($OCH_2CH_2CH_2N_3$), 60.9 (C-6^2), 48.5 ($OCH_2CH_2CH_2N_3$), 37.8 (CH_2 (Lev)), 29.8 (Me (Lev)), 28.8 ($OCH_2CH_2CH_2N_3$), 27.8 (CH_2 (Lev)).

HRMS ESI m/z calcd for $[M+Na]^+$ $C_{51}H_{54}F_5N_3O_{14}$ 1050.3418; found 1050.3419.

3-Azidopropyl 3,4-di-*O*-benzoyl-2-*O*-benzyl-6-*O*-fluorenylmethyloxycarbonyl-α-D-galactopyranosyl-(1→6)-3,4-di-*O*-benzoyl-2-*O*-benzyl-α-D-galactopyranosyl-(1→6)-2-*O*-allyl-3-*O*-levulinyl-4-*O*-pentafluorobenzoyl-α-D-galactopyranosyl-(1→6)-2,3,4-tri-*O*-benzyl-α-D-galactopyranoside (39a) and 3-azidopropyl 3,4-di-*O*-benzoyl-2-*O*-benzyl-6-*O*-fluorenylmethyloxycarbonyl-α-D-galactopyranosyl-(1→6)-3,4-di-*O*-benzoyl-2-*O*-benzyl-β-D-galactopyranosyl-(1→6)-2-*O*-allyl-3-*O*-levulinyl-4-*O*-pentafluorobenzoyl-α-D-galactopyranosyl-(1→6)-2,3,4-tri-*O*-benzyl-α-D-galactopyranoside (39b)

Molecular sieves AW-300 (660 mg) were added to a solution of donor **33** (390.9 mg, 0.337 mmol) and acceptor **38** (266.1 mg, 0.259 mmol) in dry CH_2Cl_2 (6.6 mL) under argon atmosphere at –20 °C. The mixture was stirred for 40 minutes and TfOH (15 μL, 0.169 mmol) was added. The conversion of the initial compounds was monitored with by TLC (toluene : EtOAc 6 : 1 and petroleum ether : acetone 2.5 : 1). In 20 minutes the reaction mixture was slowly warmed up to – 8 °C and stirred for another 1.5 hours. Then the mixture was diluted with $CHCl_3$ and filtered through the pad of Celite. The filtrate was washed with saturated solution of $NaHCO_3$ and the aqueous layer was extracted with $CHCl_3$ three times. The combined organic extracts were dried over anhydrous Na_2SO_4, filtered and concentrated under vacuum. Column chromatography (petroleum ether : acetone 10 : 1 → 3 : 1) gave a mixture of α- and β-isomers (307.4 mg, 55%, α : β = 13 : 1). Then 180 mg of the mixture was separated by HPLC (toluene : MeCN 13.5 : 1) to provide α-disaccharide **39a** (164.8 mg) and β-disaccharide **39b** (12.8 mg).

Data for α-disaccharide **39a**:

R_f = 0.18 (petroleum ether : acetone 2.8 : 1). Colorless syrup. $[\alpha]_D^{23}$ = 132.1.

^{1}H NMR (600 Hz, $CDCl_3$): δ 7.90 (t, J = 7.6 Hz, 4H, Ph (Bz)), 7.72 (m, 6H, Ph (Bz)), 7.57 (t, J = 7.6 Hz, 1H, Ph (Bz)), 7.54 – 7.43 (m, 5H, Ph (Bz), Fmoc), 7.43 – 7.09 (m, 45H, Ph (Bn, Bz), Fmoc + $CHCl_3$), 5.84 (br s, 2H, H-4^2, H-4^3), 5.80 – 5.73 (m, 2H, H-4^4, CH_2CH=CH_2), 5.71 (dd, $J_{3,4}$ = 3.1 Hz, $J_{3,2}$ = 10.2 Hz, 1H, H-3^3), 5.66 (dd, $J_{3,4}$ = 3.2 Hz, $J_{3,2}$ = 10.4 Hz, 1H, H-3^4), 5.45 (dd, $J_{3,4}$ = 3.2 Hz, $J_{3,2}$ = 10.4 Hz, 1H, H-3^2), 5.14 (dd, J = 1.4 Hz, 17.3 Hz, 1H, CH_2CH=C*H*H'), 5.08 – 5.04 (m, 2H, H-1^3,

CH_2CH=CH*H'*), 5.04 (d, $J_{1,2}$ = 3.3 Hz, 1H, H-1^2), 5.02 (d, $J_{1,2}$ = 3.5 Hz, 1H, H-1^1), 4.97 (d, J = 11.4 Hz, 1H, Bn_1A), 4.91 (d, $J_{1,2}$ = 3.3 Hz, 1H, H-1^4), 4.86 (d, J = 11.6 Hz, 1H, Bn_3A), 4.80 (d, J = 11.8 Hz, 1H, Bn_2A), 4.77 (d, J = 11.6 Hz, 1H, Bn_3B), 4.73 (d, J = 11.8 Hz, 1H, Bn_2B), 4.66 – 4.53 (m, 6H, H-5^3, H-5^4, Bn_1B, Bn_4A, Bn_4B, Bn_5A), 4.51 – 4.46 (m, 2H, H-5^2, Bn_5B), 4.30 (m, 4H, H-6A^4, H-6B^4, CH_2 (Fmoc)), 4.13 (m, 6H, H-2^1, H-2^3, H-2^4, H-5^1, C*H*H'-CH=CH_2, CH (Fmoc)), 4.02 – 3.94 (m, 5H, H-3^1, H-4^1, H-6A^1, H-6A^2, CH*H'*-CH=CH_2), 3.89 (dd, $J_{2,3}$ = 10.1 Hz, $J_{2,1}$ = 3.3 Hz, 1H, H-2^2), 3.87 – 3.80 (m, 2H, H-6A^3, OC*H*H'$CH_2CH_2N_3$), 3.74 (dd, $J_{6B,6A}$ = 10.4 Hz, $J_{6B,5}$ = 6.3 Hz, 1H, H-6B^2), 3.60 – 3.52 (m, 3H, H-6B^1, H-6B^3, OCH*H'*$CH_2CH_2N_3$), 3.40 (t, J = 7.0 Hz, 2H, OCH_2CH_2C*H_2*N_3), 2.81 (m, 1H, CH_2 (Lev)), 2.71 (m, 1H, CH_2 (Lev)), 2.63 (m, 1H, CH_2 (Lev)), 2.48 (m, 1H, CH_2 (Lev)), 2.15 (s, 3H, Me (Lev)), 1.90 (m, 2H, OCH_2C*H_2*CH_2N_3).

$^{13}C\{^1H\}$ NMR (150 Hz, $CDCl_3$): δ 171.7 (C=O (Lev)), 165.4, 165.0 (C=O (Bz)), 143.3, 141.1 (Fmoc), 137.6 (*ipso*-Ph (Bn)), 134.5 (CH_2*C*H=CH_2), 133.3, 133.2, 132.9, 132.8, 129.8, 129.7, 129.6, 129.5, 129.4, 128.5, 128.4, 128.3, 128.2, 128.1, 127.9, 127.8, 127.6, 127.4, 127.3, 127.1, 125.2 (Ph (Bn, Bz), Fmoc), 119.9 (Fmoc), 117.2 (CH_2CH=*C*H_2), 97.9 (C-1^3), 97.7 (C-1^1), 97.6 (C-1^2), 97.5 (C-1^4), 79.1 (C-3^1), 76.7 (C-2^1), 75.3 (C-4^1), 74.5 (CH_2 (Bn_1)), 73.5 (C-2^3), 73.5 (C-2^2), 73.2 (CH_2 (Bn_2)), 73.1 (CH_2 (Bn_3)), 73.0 (C-2^4), 72.7 (*C*H_2CH=CH_2), 72.6 (CH_2 (Bn_4)), 72.5 (CH_2 (Bn_5)), 72.3 (C-4^2), 70.1 (C-3^2, C-3^3, C-3^4, CH_2 (Fmoc)), 69.9 (C-3^2), 69.5 (C-4^4), 69.3 (C-5^1), 68.2 (C-5^3), 67.7 (C-6^1), 67.4 (C-5^2), 67.0 (C-6^2), 66.8 (C-5^4, C-6^3), 66.0 (C-6^4), 65.1 (O*C*$H_2CH_2CH_2N_3$), 48.5 (OCH_2CH_2*C*H_2N_3), 46.6 (CH (Fmoc)), 37.8 (CH_2 (Lev)), 29.8 (Me (Lev)), 28.8 (OCH_2*C*$H_2CH_2N_3$), 27.8 (CH_2 (Lev)).

HRMS ESI m/z calcd for $[M+Na]^+$ $C_{120}H_{112}F_5N_3O_{30}$ 2193.7146; found 2193.7176.

Data for β-disaccharide **39b**:

R_f = 0.18 (petroleum ether : acetone 2.8 : 1). Colorless syrup. $[\alpha]_D^{20}$ = 58.6.

^1H NMR (600 Hz, $CDCl_3$): δ 7.95 (d, J = 7.3 Hz, 2H, Ph (Bz)), 7.89 (d, J = 7.3 Hz, 2H, Ph (Bz)), 7.76 (d, J = 7.3 Hz, 2H, Ph (Bz)), 7.73 – 7.69 (m, 4H, Ph (Bz)), 7.57 (t, J = 7.5 Hz, 1H, Ph (Bz)), 7.55 – 7.44 (m, 5H, Ph (Bz), Fmoc), 7.44 – 7.26 (m, 22H, Ph (Bn, Bz), Fmoc), 7.25 – 7.10 (m, 12H, Ph (Bz)), 7.08 (t, J = 7.3 Hz, 1H, Ph (Bz)), 7.03 (m, 1H, Ph (Bz)), 5.84 (d, $J_{4,3}$ = 3.2 Hz, 1H, H-4^2), 5.81 (d, $J_{4,3}$ = 3.4 Hz, 1H, H-4^4), 5.78 (m, 1H, CH_2C*H*=CH_2), 5.07 (m, 2H, H-3^4, H-4^3), 5.43 (dd, $J_{3,4}$ = 3.4 Hz, $J_{3,2}$ = 10.2 Hz, 1H, H-3^3), 5.41 (dd, $J_{3,4}$ = 3.2 Hz, $J_{3,2}$ = 10.5 Hz, 1H, H-3^2), 5.19 (dd, J = 1.4 Hz, 17.2 Hz, 1H, CH_2CH=C*H*H'), 5.10 (d, J = 10.3 Hz, 1H, CH_2CH=CH*H'*), 4.97 (m, 2H, H-1^4, Bn_2A), 4.85 (m, 2H, H-1^2, Bn_4A), 4.82 – 4.71 (m, 5H, H-1^1, H-1^3, Bn_1A, Bn_3A, Bn_4B), 4.67 (d, J = 12.1 Hz, 1H, Bn_5A), 4.62 – 4.53 (m, 4H, Bn_1B, Bn_2B, Bn_3B, Bn_5B), 4.47 (t, $J_{5,6A}$ = $J_{5,6B}$ = 6.0 Hz, 1H, H-5^4), 4.43 (t, $J_{5,6A}$ = $J_{5,6B}$ = 6.0 Hz, 1H, H-5^2), 4.29 – 4.20 (m, 4H, H-6A^4, H-6B^4, CH_2 (Fmoc)), 4.13 (m, 1H, CH (Fmoc)), 4.11 – 3.95 (m, 7H, H-2^1, H-2^4, H-5^1, H-5^3, H-6A^2, C*H_2*-CH=CH_2), 3.93 (s, 2H, H-3^1, H-4^1), 3.89 (dd, $J_{6B,6A}$ = 9.5 Hz, $J_{6B,5}$ = 7.1 Hz, H-6A^1), 3.86 – 3.77 (m, 4H, H-2^2, H-2^3, H-6A^3, H-6B^2), 3.70 (m, 1H, OC*H*H'$CH_2CH_2N_3$), 3.54 (dd, $J_{6B,6A}$ = 10.4 Hz, $J_{6B,5}$ = 6.5 Hz, H-6B^3), 3.47 (dd, $J_{6B,6A}$ = 9.8 Hz, $J_{6B,5}$ = 5.6 Hz, H-6B^1), 3.37 (m, 1H, OCH*H'*$CH_2CH_2N_3$), 3.33 (m, 2H, OCH_2CH_2C*H_2*N_3), 2.81 (m, 1H, CH_2 (Lev)), 2.69 (m, 1H, CH_2 (Lev)), 2.62 (m, 1H, CH_2 (Lev)), 2,48 (m, 1H, CH_2 (Lev)), 2.16 (s, 3H, Me (Lev)), 1.82 (m, 2H, OCH_2C*H_2*CH_2N_3).

$^{13}C\{^1H\}$ NMR (150 Hz, $CDCl_3$): δ 165.4 (C=O (Bz)), 134.5 (CH_2*C*H=CH_2), 133.3, 132.9, 129.9, 129.8, 129.7, 129.6, 129.5, 129.4, 128.5, 128.3, 128.2, 128.1, 128.0, 127.8, 127.5, 127.4, 127.2, 125.2 (Ph (Bn, Bz), Fmoc), 119.9 (Fmoc), 117.5 (CH_2CH=*C*H_2), 102.8 (C-1^3), 98.0 (C-1^4), 97.7 (C-1^1, C-1^2), 79.0 (C-3^1), 76.8 (C-2^1), 76.7 (C-2^3), 75.4 (C-4^1), 74.8 (CH_2 (Bn_1)), 74.5 (CH_2 (Bn_2)), 73.3 (C-2^2), 72.8 (C-2^4, *C*H_2CH=CH_2), 72.7 (CH_2 (Bn_3)), 72.6 (CH_2 (Bn_4)), 72.5 (C-3^3, CH_2 (Bn_5)), 72.1 (C-5^3), 72.0 (C-4^2),

70.2 (CH_2 (Fmoc)), 69.9 (C-3^2, C-3^4), 69.5 (C-4^4, C-5^1), 69.1 (C-4^3), 67.6 (C-6^1), 67.1 (C-5^4), 66.9 (C-5^2), 66.8 (C-6^2, C-6^3), 65.9 (C-6^4), 64.7 (O*C*$H_2CH_2CH_2N_3$), 48.4 (OCH_2CH_2*C*H_2N_3), 46.5 (CH (Fmoc)), 37.8 (CH_2 (Lev)), 29.7 (Me (Lev)), 28.8 (OCH_2*C*$H_2CH_2N_3$), 27.8 (CH_2 (Lev)).

HRMS ESI m/z calcd for $[M+Na]^+$ $C_{120}H_{112}F_5N_3O_{30}$ 2193.7146; found 2193.7176.

3-Azidopropyl 3,4-di-*O*-benzoyl-2-*O*-benzyl-6-*O*-fluorenylmethyloxycarbonyl-α-D-galactopyranosyl-(1→6)-3,4-di-*O*-benzoyl-2-*O*-benzyl-α-D-galactopyranosyl-(1→6)-3-*O*-levulinyl-4-*O*-pentafluorobenzoyl-α-D-galactopyranosyl-(1→6)-2,3,4-tri-*O*-benzyl-α-D-galactopyranoside (40)

$[Ir(COD)(PPh_2Me)_2]PF_6$ (3.2 mg, 0.0038 mmol) was dissolved in anhydrous THF (1.7 mL). The resulting red suspension was bubbled through with hydrogen for 1.5 minutes until it became a light yellow solution. The solution was degassed under Ar and a solution of tetrasaccharide **39a** (158.3 mg, 0.073 mmol) in anhydrous THF (1.7 mL) was added. The reaction mixture was stirred under Ar for 3 hours. Then, a solution of I_2 (38.6 mg, 0.152 mmol) in THF/H_2O 4/1 (2.8 mL) was added and the mixture was stirred for 1 hour. The excess of I_2 was quenched with 8 mL 10% aq. solution of $Na_2S_2O_3$. The mixture was diluted with EtOAc and aqueous phase was washed with EtOAc. Combined organic extracts were dried over anhydrous Na_2SO_4, filtered and the solvent was evaporated under reduced pressure. The dry residue was purified by column chromatography (silica gel, toluene : EtOAc 10 : 1 → 3:1) to give tetrasaccharide **40** as a colorless sypup (134.1 mg, 86 %). R_f = 0.37 (toluene : EtOAc 3 : 1). $[\alpha]_D^{23}$ = 113.9.

^{1}H NMR (600 Hz, $CDCl_3$): δ 7.89 (m, 4H, Ph (Bz)), 7.72 (m, 7H, Ph (Bz)), 7.57 (t, J = 7.5 Hz, 1H, Ph (Bz)), 7.54 – 7.43 (m, 6H, Ph (Bz), Fmoc), 7.41 (t, J = 7.9 Hz, 2H, Fmoc), 7.38 – 7.32 (m, 12H, Ph (Bn, Bz), Fmoc), 7.32 – 7.08 (m, 50H, Ph (Bz)), 5.86 (d, $J_{4,3}$ = 3.3 Hz, 1H, H-4^3), 5.79 (d, $J_{4,3}$ = 3.4 Hz, 1H, H-4^4), 5.78 (d, $J_{4,3}$ = 3.1 Hz, 1H, H-4^2), 5.71 (dd, $J_{3,4}$ = 3.3 Hz, $J_{3,2}$ = 10.4 Hz, 1H, H-3^3), 5.67 (dd, $J_{3,4}$ = 3.4 Hz, $J_{3,2}$ = 10.5 Hz, 1H, H-3^4), 5.28 (dd, $J_{3,4}$ = 3.1 Hz, $J_{3,2}$ = 10.4 Hz, 1H, H-3^2), 5.06 (d, $J_{1,2}$ = 3.4 Hz, 1H, H-1^3), 4.99 (d, $J_{1,2}$ = 3.7 Hz, 1H, H-1^1), 4.95 (d, $J_{1,2}$ = 3.7 Hz, 1H, H-1^2), 4.94 – 4.91 (m, 2H, H-1^4, Bn_1A), 4.86 (d, J = 11.7 Hz, 1H, Bn_2A), 4.80 (d, J = 12.0 Hz, 1H, Bn_3A), 4.74 (d, J = 11.7 Hz, 1H, Bn_2B), 4.70 (d, J = 12.0 Hz, 1H, Bn_3B), 4.63 (m, 2H, Bn_4A, Bn_5A), 4.59 – 4.51 (m, 4H, H-5^3, H-5^4, Bn_1B, Bn_4B), 4.49 (d, J = 12.1 Hz, 1H, Bn_5B), 4.40 (t, $J_{5,6A}$ = 6.1 Hz, $J_{5,6B}$ = 6.8 Hz, 1H, H-5^2), 4.26 (dd, $J_{6A,6B}$ = 11.6 Hz, $J_{6A,5}$ = 7.1 Hz, 1H, H-6A^4), 4.22 (m, 2H, CH_2 (Fmoc)), 4.19 (dd, $J_{6B,6A}$ = 11.6 Hz, $J_{6B,5}$ = 5.0 Hz, 1H, H-6B^4), 4.10 – 4.05 (m, 4H, H-2^1, H-2^3, H-2^4, CH (Fmoc)), 4.03 – 3.93 (m, 6H, H-2^2, H-3^1, H-5^1, H-6A^1, H-6A^2), 3.89 (d, $J_{4,3}$ = 2.7 Hz, 1H, H-4^1), 3.86 – 3.80 (m, 2H, H-6A^3, OC*H*H'$CH_2CH_2N_3$), 3.69 (dd, $J_{6B,6A}$ = 10.5 Hz, $J_{6B,5}$ = 6.7 Hz, 1H, H-6B^2), 3.58 (dd, $J_{6B,6A}$ = 10.6 Hz, $J_{6B,5}$ = 5.8 Hz, 1H, H-6B^3), 3.55 (m, 1H, OCH*H'*$CH_2CH_2N_3$), 3.47 (dd, $J_{6B,6A}$ = 7.7 Hz, $J_{6B,5}$ = 3.4 Hz, 1H, H-6B^1), 3.39 (t, J = 6.9 Hz, 2H, OCH_2CH_2C*H_2*N_3), 2.79 (m, 1H, CH_2 (Lev)), 2.71 (m, 1H, CH_2 (Lev)), 2.63 (m, 1H, CH_2 (Lev)), 2.52 (m, 1H, CH_2 (Lev)), 2.14 (s, 3H, Me (Lev)), 1.91 (m, 2H, OCH_2C*H_2*CH_2N_3).

^{13}C{^{1}H} NMR (150 Hz, $CDCl_3$): δ 158.1 (Fmoc), 133.3, 129.8, 129.6, 128.5, 128.3, 128.2, 127.9, 127.8, 127.7, 127.6, 127.1, 125.2 (Ph (Bn, Bz), Fmoc), 119.9 (Fmoc), 98.2 (C-1^2), 98.0 (C-1^3), 97.7 (C-1^1, C-1^4), 79.1 (C-3^1), 76.7 (C-2^1), 75.3 (C-4^1), 74.5 (CH_2 (Bn_1)), 73.4 (CH_2 (Bn_2)), 73.3 (C-2^4, CH_2 (Bn_3)), 73.0 (C-2^3), 72.7 (CH_2 (Bn_4)), 72.6 (CH_2 (Bn_5)), 71.8 (C-4^2), 70.7 (C-3^2), 70.1 (C-3^4, CH_2 (Fmoc)), 69.9 (C-3^3, C-4^3), 69.5 (C-4^4), 68.9 (C-5^1), 68.1 (C-5^3), 67.9 (C-6^1), 67.7 (C-5^2), 67.2 (C-2^2), 67.0 (C-5^4), 66.7 (C-6^2, C-6^3), 66.0 (C-6^4), 65.1 (O*C*$H_2CH_2CH_2N_3$), 48.5 (OCH_2CH_2*C*H_2N_3), 46.6 (CH (Fmoc)), 38.0 (CH_2 (Lev)), 29.8 (Me (Lev)), 28.8 (OCH_2*C*$H_2CH_2N_3$), 27.9 (CH_2 (Lev)).

HRMS ESI m/z calcd for $[M+Na]^+$ $C_{117}H_{108}F_5N_3O_{30}$ 2153.6863; found 2153.6889.

3-Azidopropyl 3,4-di-*O*-benzoyl-2-*O*-benzyl-6-*O*-fluorenylmethyloxycarbonyl-α-D-galactopyranosyl-(1→6)-3,4-di-*O*-benzoyl-2-*O*-benzyl-α-D-galactopyranosyl-(1→6)-[2,3,4,6-tetra-*O*-benzoyl-β-D-galactofuranosyl-(1→2)]-3-*O*-levulinyl-4-*O*-pentafluorobenzoyl-α-D-galactopyranosyl-(1→6)-2,3,4-tri-*O*-benzyl-α-D-galactopyranoside (41)

Molecular sieves AW-300 (200 mg) were added to a solution of donor **22** (72.5 mg, 0.094 mmol) and acceptor **40** (134.1 mg, 0.063 mmol) in dry CH_2Cl_2 (2.0 mL) under argon atmosphere at –20 °C. The mixture was stirred for 40 minutes and TMSOTf (4.5 µL, 0.025 mmol) was added. In 1 hour the mixture was slowly warmed up. In the next 30 minutes the temperature was –12 °C, the reaction mixture was diluted with CH_2Cl_2 and filtered through the pad of Celite. The filtrate was washed with saturated solution of $NaHCO_3$ and the aqueous layer was extracted with CH_2Cl_2 three times. The combined organic extracts were dried over anhydrous Na_2SO_4, filtered and concentrated under vacuum. The residue was purified by column chromatography (toluene : EtOAc 20 : 1 → 8 : 1) to give pentasaccharide **41** (143.1 mg, 84%) as a β-isomer. R_f = 0.5 (toluene : EtOAc 6 : 1). $[\alpha]_D^{25}$ = 104.3.

^{1}H NMR (600 Hz, $CDCl_3$): δ 7.97 (m, 5H, Ph (Bz)), 7.95 – 7.90 (m, 5H, Ph (Bz)), 7.87 (d, *J* = 7.3 Hz, 2H, Ph (Bz)), 7.83 (d, *J* = 7.3 Hz, 2H, Ph (Bz)), 7.71 (m, 7H, Ph (Bz)), 7.58 – 7.42 (m, 11H, Ph (Bz), Fmoc), 7.42 – 7.26 (m, 28H, Ph (Bn, Bz), Fmoc), 7.25 – 7.17 (m, 15H, Ph (Bz)), 7.16 – 7.05 (m, 14H, Ph (Bz)), 5.93 (m, 1H, H-5^I), 5.88 (d, $J_{4,3}$ = 3.3 Hz, 1H, H-4^2), 5.87 (d, $J_{4,3}$ = 3.2 Hz, 1H, H-4^3), 5.81 (d, $J_{4,3}$ = 3.2 Hz, 1H, H-4^4), 5.75 (dd, $J_{3,4}$ = 3.2 Hz, $J_{3,2}$ = 10.4 Hz, 1H, H-3^3), 5.68 (m, 2H, H-3^4, H-3^I), 5.57 (dd, $J_{3,4}$ = 3.3 Hz, $J_{3,2}$ = 10.5 Hz, 1H, H-3^2), 5.47 (s, 1H, H-2^I), 5.42 (s, 1H, H-1^I), 5.16 (d, $J_{1,2}$ = 3.7 Hz, 1H, H-1^1), 5.09 (d, $J_{1,2}$ = 3.3 Hz, 2H, H-1^2, H-1^3), 4.93 (d, $J_{1,2}$ = 3.4 Hz, 1H, H-1^4), 4.88 (d, *J* = 11.6 Hz, 1H, Bn_1A), 4.83 (d, *J* = 11.6 Hz, 1H, Bn_2A), 4.78 (s, 2H, Bn_3A, Bn_3B), 4.70 – 4.53 (m, 11H, H-4^I, H-5^2, H-5^3, H-5^4, H-6A^I, H-6B^I, Bn_1B, Bn_2B, Bn_4A, Bn_5A, Bn_5B), 4.47 (d, *J* = 12.2 Hz, 1H, Bn_4B), 4.29 (dd, $J_{6A,6B}$ = 11.6 Hz, $J_{6A,5}$ = 7.1 Hz, 1H, H-6A^4), 4.23 (m, 3H, H-6B^4, CH_2 (Fmoc)), 4.19 (dd, $J_{2,3}$ = 10.5 Hz, $J_{2,1}$ = 3.3 Hz, 1H, H-2^2), 4.14 (dd, $J_{2,3}$ = 10.1 Hz, $J_{2,1}$ = 3.1 Hz, 1H, H-2^1), 4.12 – 3.99 (m, 5H, H-2^3, H-2^4, H-6A^1, H-6A^3, CH (Fmoc)), 3.98 (m, 1H, H-5^1), 3.91 (dd, $J_{3,4}$ = 2.9 Hz, $J_{3,2}$ = 10.3 Hz, 1H, H-3^1), 3.86 (m, 2H, H-6A^2, OC*H*H'$CH_2CH_2N_3$), 3.78 (dd, $J_{6B,6A}$ = 11.0 Hz, $J_{6B,5}$ = 7.7 Hz, 1H, H-6B^3), 7.46 (br s, 1H, H-4^1), 3.67 (m, 1H, OCH*H'*$CH_2CH_2N_3$), 3.57 (dd, $J_{6B,6A}$ = 10.6 Hz, $J_{6B,5}$ = 5.8 Hz, 1H, H-6B^2), 3.34 (m, 2H, $OCH_2CH_2CH_2N_3$), 2.81 – 2.62 (m, 3H, CH_2 (Lev)), 2.51 (m, 1H, CH_2 (Lev)), 2.03 (s, 3H, Me (Lev)), 1.95 (m, 2H, $OCH_2CH_2CH_2N_3$).

^{13}C{^{1}H} NMR (150 Hz, $CDCl_3$): δ 171.8 (C=O (Lev)), 166.2, 165.8, 165.5, 165.3, 165.1 (C=O (Bz)), 133.5, 133.4, 133.2, 132.9, 130.0, 139.9, 129.8, 129.6, 128.7, 128.6, 128.5, 128.4, 128.2, 128.1, 128.0, 127.9, 127.8, 127.7, 127.6, 127.4, 127.3, 125.3 (Ph (Bn, Bz), Fmoc), 120.0 (Fmoc), 107.3 (C-1^I), 98.1 (C-1^2, C-1^3), 97.8 (C-1^1), 97.5 (C-1^4), 81.8 (C-2^I), 81.6 (C-4^I), 79.3 (C-3^1), 77.1 (C-3^I), 76.9 (C-2^1), 75.5 (C-4^1), 74.5 (CH_2 (Bn_1)), 73.6 (C-2^3), 73.5 (C-2^2), 73.4 (CH_2 (Bn_2)), 73.1 (CH_2 (Bn_3)), 72.8 (C-2^4), 72.7 (CH_2 (Bn_4)), 72.5 (CH_2 (Bn_5)), 72.1 (C-4^2), 70.4 (C-5^I), 70.2 (C-5^1, CH_2 (Fmoc)), 70.1 (C-3^4, C-4^3), 70.0 (C-3^3), 69.6 (C-4^4), 69.4 (C-6^1), 69.0 (C-3^2), 68.2 (C-5^2), 67.4 (C-5^3), 67.1 (C-5^4), 66.8 (C-6^2), 66.6 (C-6^3), 66.1 (C-6^4), 65.5 ($OCH_2CH_2CH_2N_3$), 63.6 (C-6^I), 48.6 ($OCH_2CH_2CH_2N_3$), 46.7 (CH (Fmoc)) 38.1 (CH_2 (Lev)), 29.8 (Me (Lev)), 28.9 ($OCH_2CH_2CH_2N_3$), 27.9 (CH_2 (Lev)).

HRMS ESI m/z calcd for $[M+Na]^+$ $C_{151}H_{134}F_5N_3O_{39}$ 2731.8432; found 2731.8440.

3-Azidopropyl 3,4-di-*O*-benzoyl-2-*O*-benzyl-6-*O*-fluorenylmethyloxycarbonyl-α-D-galactopyranosyl-(1→6)-3,4-di-*O*-benzoyl-2-*O*-benzyl-α-D-galactopyranosyl-(1→6)-[2,3,4,6-tetra-*O*-benzoyl-β-D-galactofuranosyl-(1→2)]-4-*O*-pentafluorobenzoyl-α-D-galactopyranosyl-(1→6)-2,3,4-tri-*O*-benzyl-α-D-galactopyranoside (42)

Pentasaccharide **41** (143.1 mg, 0.053 mmol) was dissolved in a mixture of hydrazine monohydrate (100 μL), pyridine (1.2 mL) and AcOH (800 μL). The reaction mixture was monitored by TLC (CH_2Cl_2 : EtOAc 20 : 1). After 40 minutes the reaction was quenched with acetone (2 mL) and then the solvents were evaporated under reduced pressure. The dry residue was dissolved in toluene (3 mL) and concentrated under vacuum several times. Column chromatography of the residue (silica gel, toluene : EtOAc 25 : 1 → 8 : 1) provided pentasaccharide **42** (122.1 mg, 89%) as a colorless foam. R_f = 0.23 (toluene : EtOAc 10 : 1). $[\alpha]_D^{25}$ = 102.5.

^{1}H NMR (600 Hz, $CDCl_3$): δ 7.99 – 7.92 (m, 8H, Ph (Bz)), 7.88 (m, 4H, Ph (Bz)), 7.77 – 7.69 (m, 6H, Ph (Bz)), 7.58 – 7.07 (Ph (Bn, Bz), Fmoc + $CHCl_3$), 5.92 – 5.87 (m, 3H, H-4^{2}, H-4^{3}, H-5^{I}), 5.81 (d, $J_{4,3}$ = 3.1 Hz, 1H, H-4^{4}), 5.77 (dd, $J_{3,4}$ = 3.2 Hz, $J_{3,2}$ = 10.5 Hz, 1H, H-3^{3}), 5.74 (m, 1H, H-3^{I}), 5.69 (dd, $J_{3,4}$ = 3.1 Hz, $J_{3,2}$ = 10.5 Hz, 1H, H-3^{4}), 5.53 (s, 1H, H-1^{I}), 5.50 (m, 1H, H-2^{I}), 5.16 (d, $J_{1,2}$ = 3.7 Hz, 1H, H-1^{3}), 5.10 (d, $J_{1,2}$ = 3.6 Hz, 1H, H-1^{2}), 5.09 (d, $J_{1,2}$ = 3.6 Hz, 1H, H-1^{1}), 4.94 (m, 2H, H-1^{4}, Bn_1A), 4.82 (d, J = 11.7 Hz, 1H, Bn_2A), 4.80 – 4.73 (m, 3H, Bn_3A, Bn_3B, Bn_4A), 4.71 – 4.61 (m, 6H, H-4^{I}, H-6A^{I}, H-6B^{I}, Bn_1B, Bn_2B, Bn_5A), 4.59 – 4.52 (m, 4H, H-5^{3}, H-5^{4}, Bn_4B, Bn_5B), 4.49 (m, 1H, H-5^{2}), 4.36 (dd, $J_{3,4}$ = 3.0 Hz, $J_{3,2}$ = 9.9 Hz, 1H, H-3^{2}), 4.28 (dd, $J_{6A,6B}$ = 11.5 Hz, $J_{6A,5}$ = 7.1 Hz, 1H, H-6A^{4}), 4.22 (m, 3H, H-6B^{4}, CH_2 (Fmoc)), 4.14 – 3.98 (m, 8H, H-2^{1}, H-2^{2}, H-2^{3}, H-2^{4}, H-5^{1}, H-6A^{1}, H-6A^{2}, CH (Fmoc)), 3.94 (m, 2H, H-3^{1}, H-4^{1}), 3.88 (dd, $J_{6A,6B}$ = 10.4 Hz, $J_{6A,5}$ = 6.2 Hz, 1H, H-6A^{3}), 3.81 (m, 1H, OC*H*H'$CH_2CH_2N_3$), 3.77 (m, 1H, H-6B^{2}), 3.60 (m, 2H, H-6B^{3}, OCH*H*'$CH_2CH_2N_3$), 3.50 (m, 1H, H-6B^{1}), 3.32 (m, 2H, $OCH_2CH_2CH_2N_3$), 2.97 (br s, 0.5H, OH), 1.83 (m, 2H, $OCH_2CH_2CH_2N_3$).

$^{13}C\{^{1}H\}$ NMR (150 Hz, $CDCl_3$): δ 166.1, 165.6, 165.5, 165.2 (C=O (Bz)), 158.8, 154.9 (Fmoc), 143.4, 141.2 (*ipso*-Ph (Bz), Fmoc), 133.6, 133.3, 133.1, 132.9, 130.0, 129.9, 129.8, 129.6, 128.6, 128.5, 128.4, 128.3, 128.2, 128.0, 127.9, 127.8, 127.7, 127.6, 127.4, 127.2, 127.6, 127.4, 127.2, 125.3 (Ph (Bn, Bz), Fmoc), 120.0 (Fmoc), 108.0 (C-1^{I}), 98.3 (C-1^{2}), 98.2 (C-1^{3}), 97.8 (C-1^{4}), 97.7 (C-1^{1}), 83.0 (C-2^{I}), 80.6 (C-4^{I}), 79.2 (C-3^{1}), 77.4 (C-2^{2}), 76.9 (C-2^{1}, C-3^{I}), 75.8 (C-4^{1}), 74.6 (CH_2 (Bn_1)), 73.9 (C-2^{3}), 73.5 (C-4^{2}), 73.3 (CH_2 (Bn_2)), 73.2 (CH_2 (Bn_3)), 72.8 (C-2^{4}, CH_2 (Bn_4)), 72.6 (CH_2 (Bn_5)), 70.3 (C-5^{I}), 70.2 (C-3^{4}, C-5^{1}, CH_2 (Fmoc)), 70.0 (C-3^{3}, C-4^{3}), 69.7 (C-4^{4}), 69.1 (C-6^{1}), 68.2 (C-5^{3}), 67.8 (C-3^{2}), 67.7 (C-5^{2}), 67.1 (C-5^{4}), 66.8 (C-6^{2}, C-6^{3}), 66.2 (C-6^{4}), 65.7 ($OCH_2CH_2CH_2N_3$), 63.5 (C-6^{I}), 48.6 ($OCH_2CH_2CH_2N_3$), 46.7 (CH (Fmoc)), 28.8 ($OCH_2CH_2CH_2N_3$).

HRMS ESI m/z calcd for $[M+Na]^+$ $C_{146}H_{128}F_5N_3O_{37}$ 2632.8039; found 2632.8036.

3-Azidopropyl 3,4-di-*O*-benzoyl-2-*O*-benzyl-6-*O*-fluorenylmethyloxycarbonyl-α-D-galactopyranosyl-(1→6)-3,4-di-*O*-benzoyl-2-*O*-benzyl-α-D-galactopyranosyl-(1→6)-[2,3-di-*O*-(2,3,4,6-tetra-*O*-benzoyl-β-D-galactofuranosyl)]-4-*O*-pentafluorobenzoyl-α-D-galactopyranosyl-(1→6)-2,3,4-tri-*O*-benzyl-α-D-galactopyranoside (43)

Molecular sieves AW-300 (180 mg) were added to a solution of donor **22** (53.7 mg, 0.070 mmol) and acceptor **42** (121.7 mg, 0.047 mmol) in dry CH_2Cl_2 (1.8 mL) under argon atmosphere at –20 °C. The mixture was stirred for 40 minutes and TMSOTf (3.3 μL, 0.019 mmol) was added. In 1 hour the mixture was slowly warmed up. In the next 30 minutes the temperature was –10 °C, the reaction mixture was diluted with CH_2Cl_2 and filtered through the pad of Celite. The filtrate was washed with saturated solution of $NaHCO_3$ and the aqueous layer was extracted with CH_2Cl_2 three times. The combined

organic extracts were dried over anhydrous Na_2SO_4, filtered and concentrated under vacuum. The residue was purified by column chromatography (toluene : EtOAc 35 : 1 → 15 : 1) to give hexasaccharide **43** (128.9 mg, 87%) as a β-isomer. R_f = 0.19 (toluene : EtOAc 20 : 1). $[\alpha]_D^{25}$ = 74.3.

^{1}H NMR (600 Hz, $CDCl_3$): δ 8.00 – 7.94 (m, 4H, Ph (Bz)), 7.91 (m, 8H, Ph (Bz)), 7.85 (m, 4H, Ph (Bz)), 7.81 (d, J = 7.1 Hz, 2H, Ph (Bz)), 7.71 (m, 6H, Ph (Bz)), 7.58 (d, J = 7.5 Hz, 2H, Ph (Bz)), 7.57 – 7.26 (m, 39 H, Ph (Bn, Bz), Fmoc + $CHCl_3$), 7.25 – 7.15 (m, 18H, Ph (Bz) + toluene), 7.15 – 7.05 (m, 16H, Ph (Bz)), 7.03 (t, J = 7.5 Hz, 2H, Ph (Bz)), 6.12 (m, 1H, H-5^{II}), 5.98 (m, 1H, H-5^{I}), 5.91 (d, $J_{4,3}$ = 3.0 Hz, 1H, H-4^{2}), 5.88 (d, $J_{4,3}$ = 3.1 Hz, 1H, H-4^{3}), 5.78 (d, $J_{4,3}$ = 3.3 Hz, 1H, H-4^{4}), 5.75 (dd, $J_{3,4}$ = 3.1 Hz, $J_{3,2}$ = 10.6 Hz, 1H, H-3^{3}), 5.73 (s, 1H, H-1^{II}), 5.72 – 5.68 (m, 3H, H-1^{I}, H-3^{I}, H-3^{4}), 5.67 (s, 1H, H-2^{II}), 5.44 (s, 1H, H-2^{I}), 5.39 (d, $J_{3,4}$ = 5.1 Hz, 1H, H-3^{II}), 5.17 (d, $J_{1,2}$ = 3.7 Hz, 1H, H-1^{1}), 5.15 (d, $J_{1,2}$ = 3.3 Hz, 1H, H-1^{3}), 5.13 (d, $J_{1,2}$ = 3.6 Hz, H-1^{2}), 4.99 (m, 1H, H-4^{II}), 4.90 (d, $J_{1,2}$ = 3.3 Hz, 1H, H-1^{4}), 4.85 (d, J= 11.5 Hz, 1H, Bn_1A), 4.80 (d, J = 11.9 Hz, 1H, Bn_2A), 4.79 – 4.58 (m, 9H, H-6A^{I}, H-6B^{I}, H-6A^{II}, H-6B^{II}, Bn_2B, Bn_3A, Bn_3B, Bn_4A, Bn_5A), 4.58 – 4.51 (m, 6H, H-4^{I}, H-5^{2}, H-5^{3}, H-5^{4}, Bn_1B, Bn_4B), 4.48 (d, J = 12.6 Hz, 1H, Bn_5B), 4.04 (dd, $J_{3,4}$ = 3.0 Hz, $J_{3,2}$ = 10.0 Hz, 1H, H-3^{2}), 4.32 (dd, $J_{2,3}$ = 10.0 Hz, $J_{2,1}$ = 3.6 Hz, 1H, H-2^{2}), 4.25 (dd, $J_{6A,6B}$ = 11.5 Hz, $J_{6A,5}$ = 7.0 Hz, 1H, H-6A^{4}), 4.23 – 4.16 (m, 3H, H-6B^{4}, CH_2 (Fmoc)), 4.14 (dd, $J_{2,3}$ = 10.1 Hz, $J_{2,1}$ = 3.7 Hz, 1H, H-2^{1}), 4.10 – 4.02 (m, 4H, H-2^{3}, H-2^{4}, H-6A^{1}, CH (Fmoc)), 3.96 (m, 1H, H-5^{1}), 3.90 – 3.84 (m, 3H, H-3^{1}, H-6A^{2}, OC*H*H'$CH_2CH_2N_3$), 3.82 (dd, $J_{6A,6B}$ = 10.6 Hz, $J_{6A,5}$ = 6.5 Hz, 1H, H-6A^{3}), 3.74 (dd, $J_{6B,6A}$ = 11.2 Hz, $J_{6B,5}$ = 6.0 Hz, 1H, H-6B^{2}), 3.67 – 3.60 (m, 2H, H-4^{1}, OCH*H'*$CH_2CH_2N_3$), 3.55 (dd, $J_{6B,6A}$ = 10.6 Hz, $J_{6B,5}$ = 6.5 Hz, 1H, H-6B^{3}), 3.40 (m, 2H, $OCH_2CH_2CH_2N_3$), 3.34 (m, 1H, H-6B_1), 1.92 (m, 2H, $OCH_2CH_2CH_2N_3$).

^{13}C{^{1}H} NMR (150 Hz, $CDCl_3$): δ 166.0, 165.9, 165.8, 165.7, 165.6, 165.5, 165.3, 165.1 (C=O (Bz)), 158.7, 154.9 (Fmoc), 143.4, 141.2 (*ipso*-Ph (Bz), Fmoc), 139.1, 139.0, 138.5, 138.3, 137.8 (*ipso*-Ph (Bn)), 133.3, 133.3, 133.2, 133.1, 133.0, 132.9, 132.8, 130.0, 129.9, 129.8, 129.6, 128.6, 128.5, 128.4, 128.3, 128.2, 128.1, 127.9, 127.8, 127.7, 127.5, 127.4, 127.3, 127.2, 125.3 (Ph (Bn, Bz), Fmoc), 120.0 (Fmoc), 107.7 (C-1^{II}), 107.5 (C-1^{I}), 98.4 (C-1^{2}), 97.9 (C-1^{3}), 97.7 (C-1^{4}), 97.6 (C-1^{1}), 82.3 (C-2^{I}), 82.2 (C-4^{I}), 81.8 (C-2^{II}), 81.7 (C-4^{II}), 79.4 (C-3^{1}), 77.3 (C-3^{I}), 77.2 (C-2^{II}), 77.0 (C-2^{1}), 75.6 (C-2^{2}, C-4^{1}), 74.4 (CH_2 (Bn_1)), 74.2 (C-4^{2}), 73.8 (C-2^{3}), 73.3 (C-3^{2}, CH_2 (Bn_2)), 73.1 (CH_2 (Bn_3)), 72.2 (C-2^{3}), 74.4 (C-2^{4}, CH_2 (Bn_4)), 72.4 (CH_2 (Bn_5)), 70.7 (C-5^{II}), 70.4 (C-5^{I}), 70.3 (C-5^{1}), 70.2 (C-4^{3}), 70.2 (CH_2 (Fmoc)), 70.1 (C-3^{4}), 69.9 (C-3^{3}), 69.8 (C-6^{1}), 69.7 (C-4^{4}), 68.2 (C-5^{2}, C-5^{3}), 67.0 (C-5^{4}, C-6^{2}), 66.9 (C-6^{3}), 66.2 (C-6^{4}), 65.2 (O*C*$H_2CH_2CH_2N_3$), 63.5 (C-6^{I}, C-6^{II}), 48.6 (OCH_2CH_2*C*H_2N_3), 46.7 (CH (Fmoc)), 29.0 (OCH_2*C*$H_2CH_2N_3$).

HRMS ESI m/z calcd for $[M+Na]^+$ $C_{180}H_{154}F_5N_3O_{46}$ 3212.9683; found 3212.9681.

3-Azidopropyl 2-*O*-benzyl-α-D-galactopyranosyl-(1→6)-2-*O*-benzyl-α-D-galactopyranosyl-(1→6)-[2,3-di-*O*-(β-D-galactofuranosyl)]-α-D-galactopyranosyl-(1→6)-2,3,4-tri-*O*-benzyl-α-D-galactopyranoside (S8)

To a solution of **43** (128.5 mg, 0.040 mmol) in a mixture of dry THF (600 µL) and dry CH_2Cl_2 (1.45 mL) *N*-methylmorpholine (27 µL, 0.242 mmol) was added. After 48 hours TLC (toluene : EtOAc 10 : 1) indicated that the reaction had completed. The base was quenched with 0.1 M aqueous solution of H_2SO_4 (100 µL) and the mixture was washed with brine. The aqueous phase was washed with $CHCl_3$ three times; the combined organic extracts were dried over anhydrous Na_2SO_4, filtered and concentrated under vacuum. The dry residue was dissolved in a mixture of anhydrous MeOH (1.0 mL) and anhydrous CH_2Cl_2 (0.5 mL) and NaOMe (33 µL, 1M solution in MeOH) was added. After 48 hours of stirring the reaction was quenched with AcOH (5 µL) to neutral pH of the solution. The solvents were removed under vacuum. Column chromatography of the dry residue (silica gel, $CHCl_3$: MeOH 10 : 1 → 2.5 : 1)

gave hexasaccharide **S8** (43.4 mg, 81%) as a colorless syrup. R_f = 0.2 ($CHCl_3$: MeOH 2.5 : 1). $[\alpha]_D^{25}$ = 32.3 (C = 5 mg/ml).

^{1}H NMR (600 Hz, $CDCl_3$ + CD_3OD): δ 7.39 – 7.22 (m, 27H, Ph (Bn)), 5.17 (m, 2H, H-1^{I}, H-1^{II}), 4.90 (br d, 1H, Bn_1A), 4.85 (br d, 1H, H-1^{1}), 4.83 – 4.76 (4H, H-1^{2}, H-1^{3}, H-1^{4}, Bn_3A), 4.75 – 4.64 (m, 4H, Bn_2A, Bn_2B, Bn_3B, Bn_5A), 4.64 – 4.56 (m, 4H, Bn_1B, Bn_4A, Bn_4B, Bn_5B), 4.10 (m, 3H, H-2^{II}, H-4^{II}, H-4^{3} or H-4^{4}), 4.08 – 3.82 (m, 20H, H-2^{1}, H-2^{2}, H-2^{I}, 6 × H-3, H-4^{1}, H-4^{2}, H-4^{3} or H-4^{4}, H-4^{I}, H-5^{1}, H-5^{2}, H-5^{3}, H-5^{4} + EtOAc), 3.82 – 3.61 (m, 13H, H-2^{3}, H-2^{4}, H-5^{I}, H-5^{II}, H-6A^{1}, H-6A^{2} or H-6A^{3}, H-6B^{2} or H-6B^{3}, H-6A^{4}, H-6B^{4}, H-6A^{I}, H-6A^{II}, H-6B^{II}, OC*H*H'$CH_2CH_2N_3$), 3.61 – 3.52 (m, 3H, H-6A^{2} or H-6A^{3}, H-6B^{2} or H-6B^{3}, H-6B^{I}), 3.49 (m, 1H, H-6B^{1}), 3.45 (m, 1H, OCH*H'*$CH_2CH_2N_3$), 3.43 – 3.31 (m, 6H, $OCH_2CH_2CH_2N_3$ + CH_3OH), 1.82 (m, 2H, $OCH_2CH_2CH_2N_3$).

^{13}C{^{1}H} NMR (150 Hz, $CDCl_3$ + CD_3OD): δ 138.6, 138.5, 138.2, 138.1 (*ipso*-Ph (Bn)), 128.5, 128.3, 128.2, 128.0, 127.9, 127.7, 127.5 (Ph (Bn)), 109.9 (C-1^{II}), 109.3 (C-1^{I}), 98.5 (C-1^{2}), 97.5 (C-1^{1}), 97.0 (C-1^{3} or C-1^{4}), 96.8 (C-1^{3} or C-1^{4}), 85.1 (C-4^{I}, C-4^{II}), 80.8 (C-2^{II}), 80.2 (C-2^{I}), 78.8 (C-2^{1}), 77.8 (C-3^{II}), 77.4 (C-3^{I}), 76.7 (C-2^{3} or C-2^{4}), 76.5 (C-3^{2}), 76.4 (C-2^{1}), 76.3 (C-2^{3} or C-2^{4}), 75.5 (C-4^{1}), 74.7 (CH_2 (Bn_1)), 73.7 (C-2^{2}), 73.2 (CH_2 (Bn_2)), 73.1 (CH_2 (Bn_3, Bn_4)), 72.9 (CH_2 (Bn_5)), 71.2 (C-5^{II}), 70.9 (C-5^{I}), 70.3 (C-4^{3} or C-4^{4}), 69.7 (C-4^{3} or C-4^{4}), 69.5 (C-5^{1}, C-5^{4}), 69.4 (C-5^{3}), 69.4 (C-5^{2}), 69.0 (C-3^{3}, C-3^{4}), 68.6 (C-6^{1}), 67.5 (C-6^{2} or C-6^{3}), 66.6 (C-6^{2} or C-6^{3}), 64.9 ($OCH_2CH_2CH_2N_3$), 63.2 (C-6^{II}), 63.0 (C-6^{I}), 61.2 (C-6^{4}), 48.4 ($OCH_2CH_2CH_2N_3$), 28.7 ($OCH_2CH_2CH_2N_3$).

HRMS ESI m/z calcd for $[M+Na]^+$ $C_{74}H_{97}N_3O_{31}$ 1546.6004; found 1546.5996.

3-Aminopropyl α-D-galactopyranosyl-(1→6)-α-D-galactopyranosyl-(1→6)-[2,3-di-*O*-(β-D-galactofuranosyl)]-α-D-galactopyranosyl-(1→6)-α-D-galactopyranoside (5a)

$Pd(OH)_2/C$ (20 %wt, 15 mg) and 1M aq. solution of HCl (63 μL) were added to added to a solution of hexasaccharide **S8** (42.0 mg, 0.031 mmol) in a mixture of anhydrous EtOAc (1.14 mL) and anhydrous MeOH (2.25 mL). The reaction mixture was intensively stirred under a hydrogen atmosphere for 7 hours. Then, the mixture was diluted with MeOH and water, the catalyst was filtered and the filtrate was concentrated under vacuum. Hexasaccharide **5a** was purified by gel-permeation chromatography on the TSK HW-40(S) column and isolated as a fluffy solid (18.8 mg, 57%). R_f = 0.12 (^{n}BuOH : EtOH : H_2O : NH_3 0.5 : 1 : 0.8 : 0.8). $[\alpha]_D^{25}$ = 92.2 (H_2O).

^{1}H NMR (600 Hz, D_2O, 303 K): δ 5.19 (s, 1H, H-1^{I}), 5.17 (s, 1H, H-1^{II}), 5.07 (d, $J_{1,2}$ = 3.0 Hz, 1H, H-1^{2}), 5.00 (d, $J_{1,2}$ = 3.2 Hz, 1H, H-1^{3}), 4.99 (d, $J_{1,2}$ = 3.3 Hz, 1H, H-1^{4}), 4.96 (d, $J_{1,2}$ = 2.6 Hz, 1H, H-1^{1}), 4.22 – 4.16 (m, 3H, H-2^{I}, H-3^{3}, H-5^{3}), 4.14 – 4.09 (m, 3H, H-2^{II}, H-4^{2}, H-5^{1}), 4.08 – 4.05 (m, 2H, H-3^{I}, H-3^{II}), 4.04 – 3.96 (m, 8H, H-2^{2}, H-3^{2}, H-3^{4}, H-4^{1}, H-4^{I}, H-4^{II}, H-5^{2}, H-5^{4}), 3.96 – 3.80 (m, 12H, H-2^{1}, H-2^{3}, H-2^{4}, H-3^{1}, H-4^{3}, H-4^{4}, H-5^{I}, H-5^{II}, H-6A^{1}, H-6A^{2}, H-6A^{3}, OC*H*H'$CH_2CH_2N_3$), 3.75 – 3.72 (m, 3H, H-6B^{2}, H-6A^{4}, H-6B^{4}), 3.72 – 3.64 (m, 5H, H-6B^{1}, H-6B^{3}, H-6A^{I}, H-6B^{I}, H-6A^{II}), 3.64 – 3.60 (m, 2H, H-6B^{II}, OCH*H'*$CH_2CH_2NH_2$), 3.17 (m, 2H, $OCH_2CH_2CH_2NH_2$), 2.03 (m, 2H, $OCH_2CH_2CH_2NH_2$).

^{13}C{^{1}H} NMR (150 Hz, D_2O, 303 K): δ 109.9 (C-1^{II}), 109.6 (C-1^{I}), 99.2 (C-1^{1}), 98.7 (C-1^{2}), 98.6 (C-1^{3}), 98.3 (C-1^{4}), 83.6 (C-4^{I}), 83.4 (C-4^{II}), 82.2 (C-2^{II}), 82.1 (C-2^{I}), 77.7 (C-3^{II}), 77.5 (C-3^{I}), 76.0 (C-2^{2}), 75.8 (C-3^{2}), 71.6 (C-5^{2}), 71.3 (C-5^{I}, C-5^{II}), 70.3 (C-4^{2}), 70.1 (C-2^{1}, C-4^{4}), 70.0 (C-2^{1}, C-3^{4}, C-2^{1}), 69.9, 69.3, 69.2, 68.9, 68.9, 68.7, 67.6 (C-2^{3}, C-2^{4}, C-3^{3}, C-4^{1}, C-4^{3}, C-5^{3}, C-5^{4}), 67.6 (C-6^{1}), 67.2 (C-6^{2}), 67.0 (C-6^{3}), 66.7 ($OCH_2CH_2CH_2NH_2$), 63.4 (C-6^{II}), 63.2 (C-6^{I}), 61.8 (C-6^{4}), 38.6 ($OCH_2CH_2CH_2NH_2$), 27.1 ($OCH_2CH_2CH_2NH_2$).

HRMS ESI m/z calcd for $[M+Na]^+$ $C_{39}H_{69}NO_{31}$ 1070.3746 ; found 1070.3734.

Conjugate of the hexasaccharide with biotin (5b)

A solution of activated ester of biotin in DMF[5] (62 µmol/ml, 33.5 µL 0.0017 mmol) and dry Et_3N (1.7 µL) was added to a solution of hexasaccharide **5a** (1.5 mg, 0.0014 mmol) in DMF (330 µL). The mixture was stirred for 1 hour and then loaded onto the TSK HW-40(S) column and washed with a 0.1 M solution of AcOH. The fractions with the product were combined and dried by lyophilization to afford **5b** (1.5 mg, 65%). R_f = 0.33 (BPS : AMW 1 :1).

Characteristic signals in ^{1}H NMR (600 Hz, D_2O, 303 K): δ 5.15 (s, 1H, H-1^I), 5.12 (s, 1H, H-1^{II}), 5.02 (d, $J_{1,2}$ = 3.4 Hz, 1H, H-1^2), 4.96 (d, $J_{1,2}$ = 3.7 Hz, 1H, H-1^3), 4.95 (d, $J_{1,2}$ = 3.7 Hz, 1H, H-1^4), 4.90 (d, $J_{1,2}$ = 3.8 Hz, 1H, H-1^1), 4.57 (m, 1H, H-6a biotin), 4.39 (m, 1H, H-3a biotin), 3.75 (t, J = 6.0 Hz, 3H, OCH_2CH$_2$O biotin), 3.59 (t, J = 6.0 Hz, 3H, CH_2O biotin), 3.54 (m, 1H, OCHH'CH$_2$CH$_2$NH$_2$), 3.36 (t, J = 5.2 Hz, 2H, CH_2NH biotin), 3.29 (m, 3H, biotin, OCH$_2$CH$_2$CH_2NH$_2$), 2.96 (dd, J = 5.2 Hz, J = 13.0 Hz, 1H, H-6A biotin), 2.75 (d, J = 13.0 Hz, 1H, H-6B biotin), 2.50 (t, J = 6.0 Hz, 2H, C(O)CH$_2$CH_2O biotin), 2.24 (t, J = 7.1 Hz, 2H, Hα biotin), 1.88–1.51 (m, 7H, HΔ-A biotin, HΔ-B biotin, H-β biotin, H-γ biotin, OCH$_2$CH_2CH$_2$NH$_2$).

Characteristic signals in ^{13}C{^{1}H} NMR (150 Hz, D_2O, 303 K): δ 110.1 (C-1^{II}), 109.9(C-1^I), 99.3 (C-1^1), 98.9 (C-1^2), 98.8 (C-1^3, C-1^4), 69.7 (CH$_2$O biotin), 67.7 (OCH$_2$CH$_2$O biotin), 66.7 (OCH$_2$CH$_2$CH$_2$NH$_2$), 63.4 (C3-a biotin), 61.6 (C6-a biotin), 56.1 (C-4 biotin), 40.6 (C-6 biotin), 39.8 (CH$_2$NH biotin), 37.8 (OCH$_2$CH$_2$$CH_2NH_2$), 37.0 (C(O)$CH_2CH_2$O biotin), 36.3 (C-α biotin), 29.1 (OCH$_2$$CH_2CH_2NH_2$), 28.5 (C-γ biotin), 28.8 (CΔ-A, CΔ-B biotin), 25.9 (C-β biotin).

HRMS ESI m/z calcd for $[M+Na]^+$ $C_{64}H_{112}N_4O_{40}S$ 1631.6466 ; found 1631.6453.

IX. References

1. K. Tamura, H. Mizukami, K. Maeda, H. Watanabe and K. Uneyama, *J. Org. Chem.*, 1993, **58**, 32–35. *doi: 10.1021/jo00053a011*

2. M. J. L. Thijssen, M. N. van Rijswijk, J. P. Kamerling and J. F. G. Vliegenthart, *Carbohydrate Research*, 1998, **306**, 93–109. *doi: 10.1016/S0008-6215(97)00271-1*

3. S.-Y. Zhu and J.-S. Yang, *Tetrahedron*, 2012, **68**, 3795–3802. *doi: 10.1016/j.tet.2012.03.074*

4. V. S. Dorokhova, A. G. Gerbst, B. S. Komarova, J. O. Previato, L. M. Previato, A. S. Dmitrenok, A. S. Shashkov, V. B. Krylov and N. E. Nifantiev, *Org. Biomol. Chem.*, 2021, **19**, 2923–2931. *doi: 10.1039/D0OB02071K*

5. Y. E. Tsvetkov, M. Burg-Roderfeld, G. Loers, A. Ardá, E. V. Sukhova, E. A. Khatuntseva, A. A. Grachev, A. O. Chizhov, H.-C. Siebert, M. Schachner, J. Jiménez-Barbero and N. E. Nifantiev, *J. Am. Chem. Soc.*, 2012, **134**, 426–435. *doi: 10.1021/ja2083015*

^{1}H-NMR of **8** (300 MHz, $CDCl_3$,)

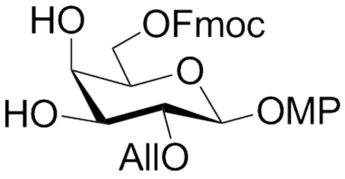

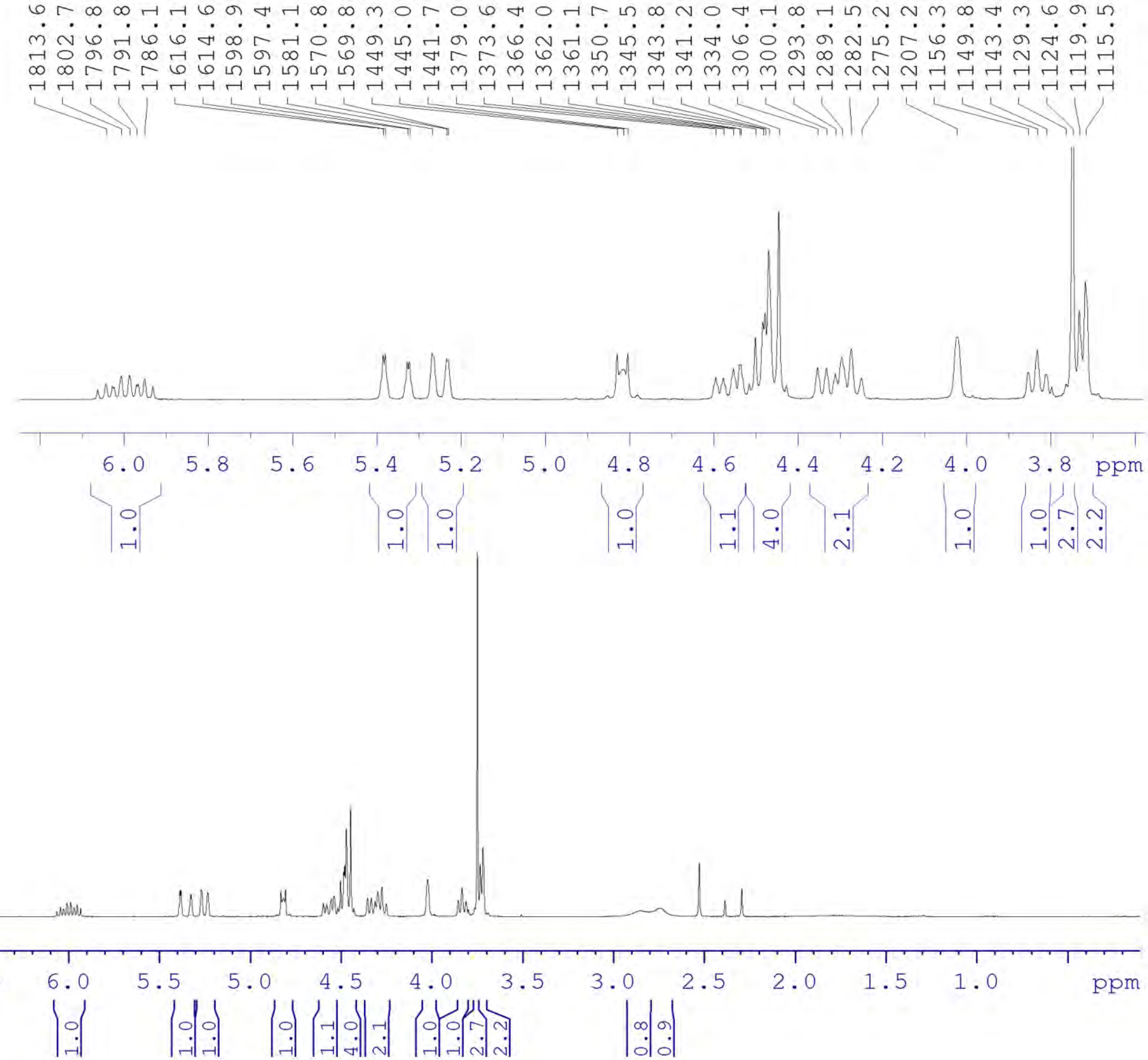

^{13}C-NMR of **8** (75 MHz, $CDCl_3$)

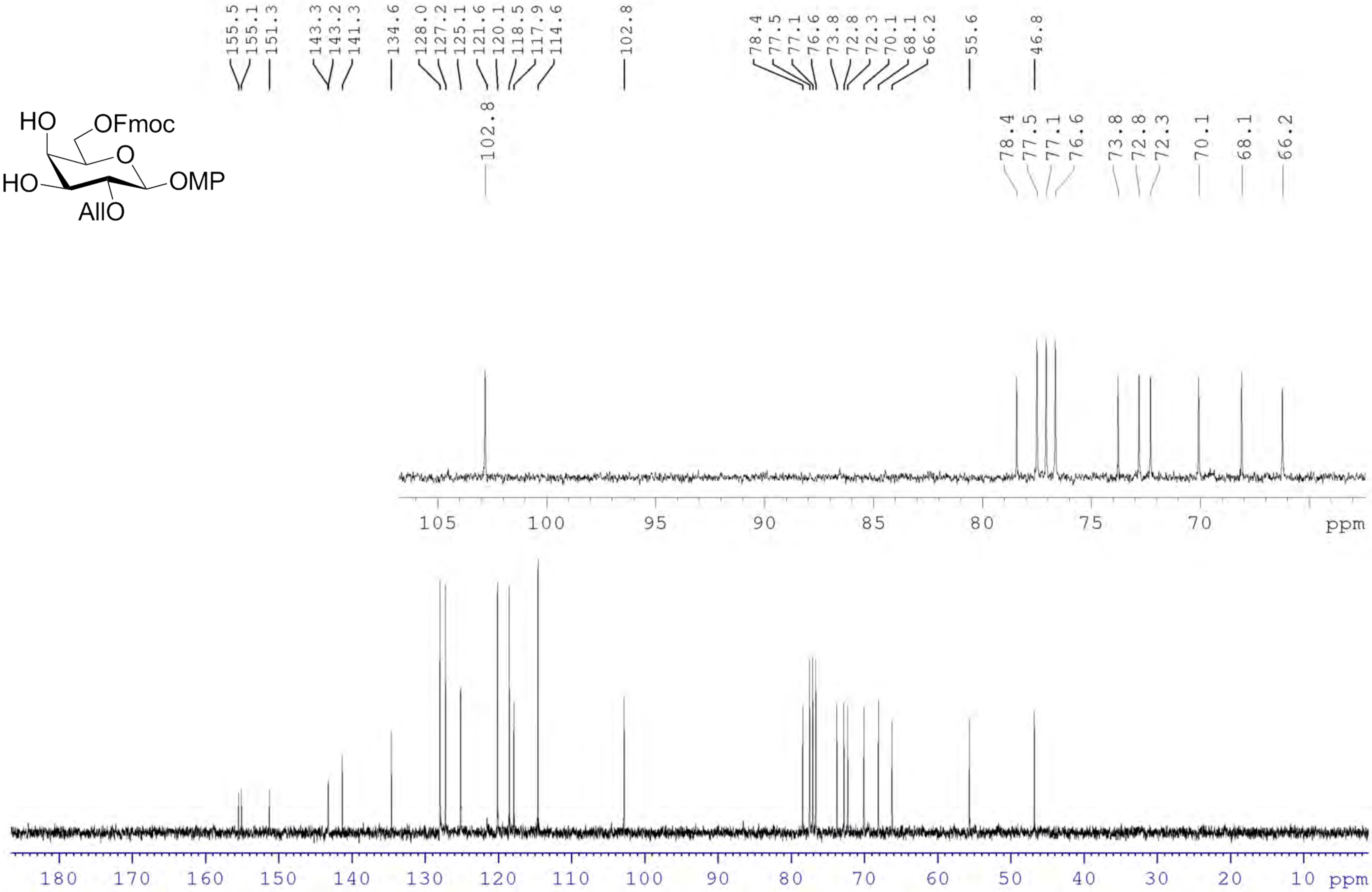

^{1}H-NMR of **10** (300 MHz, $CDCl_3$)

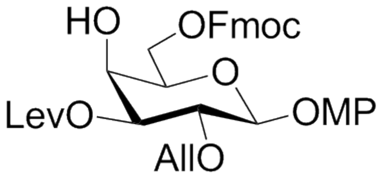

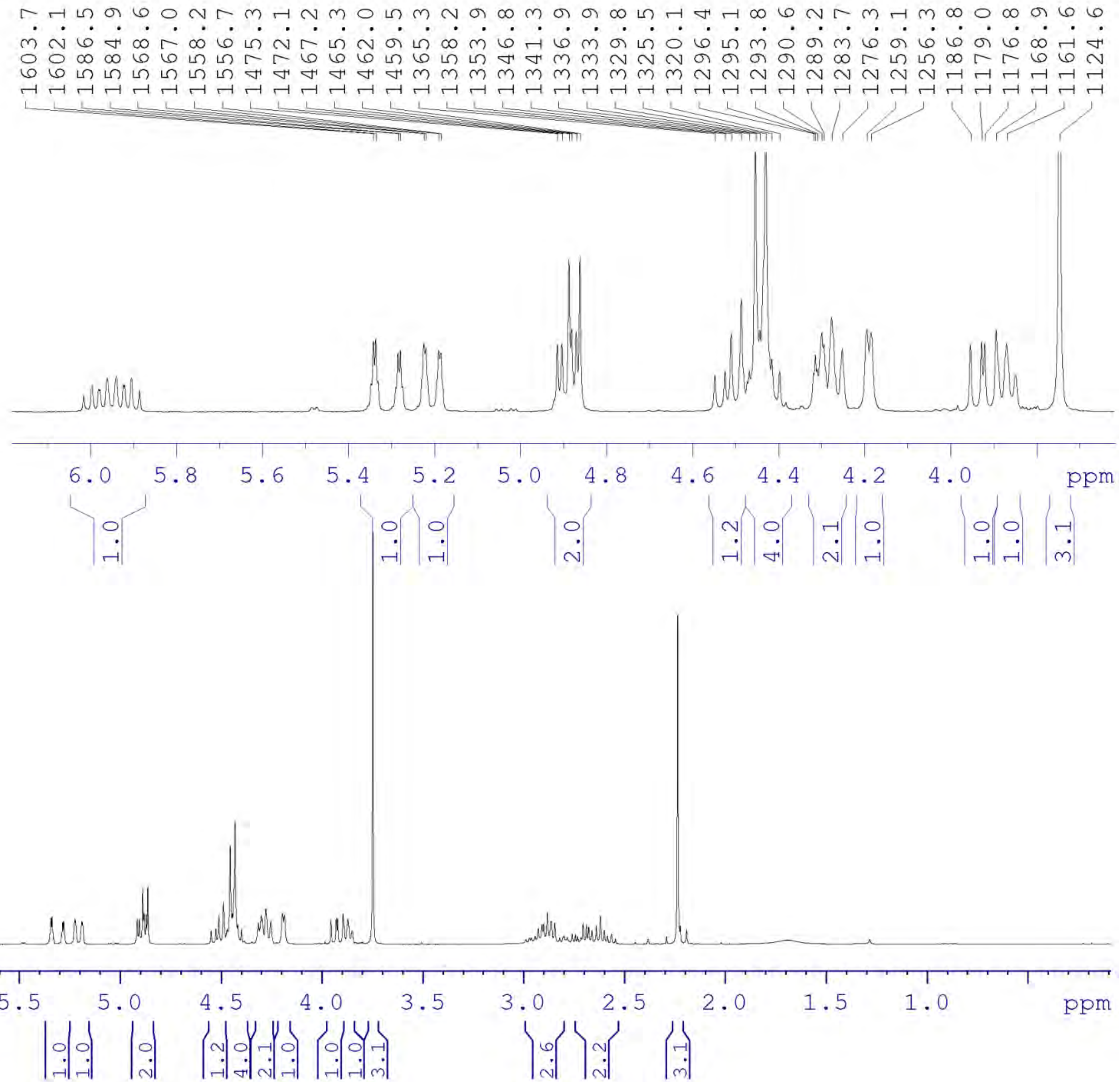

^{13}C-NMR of **10** (75 MHz, $CDCl_3$)

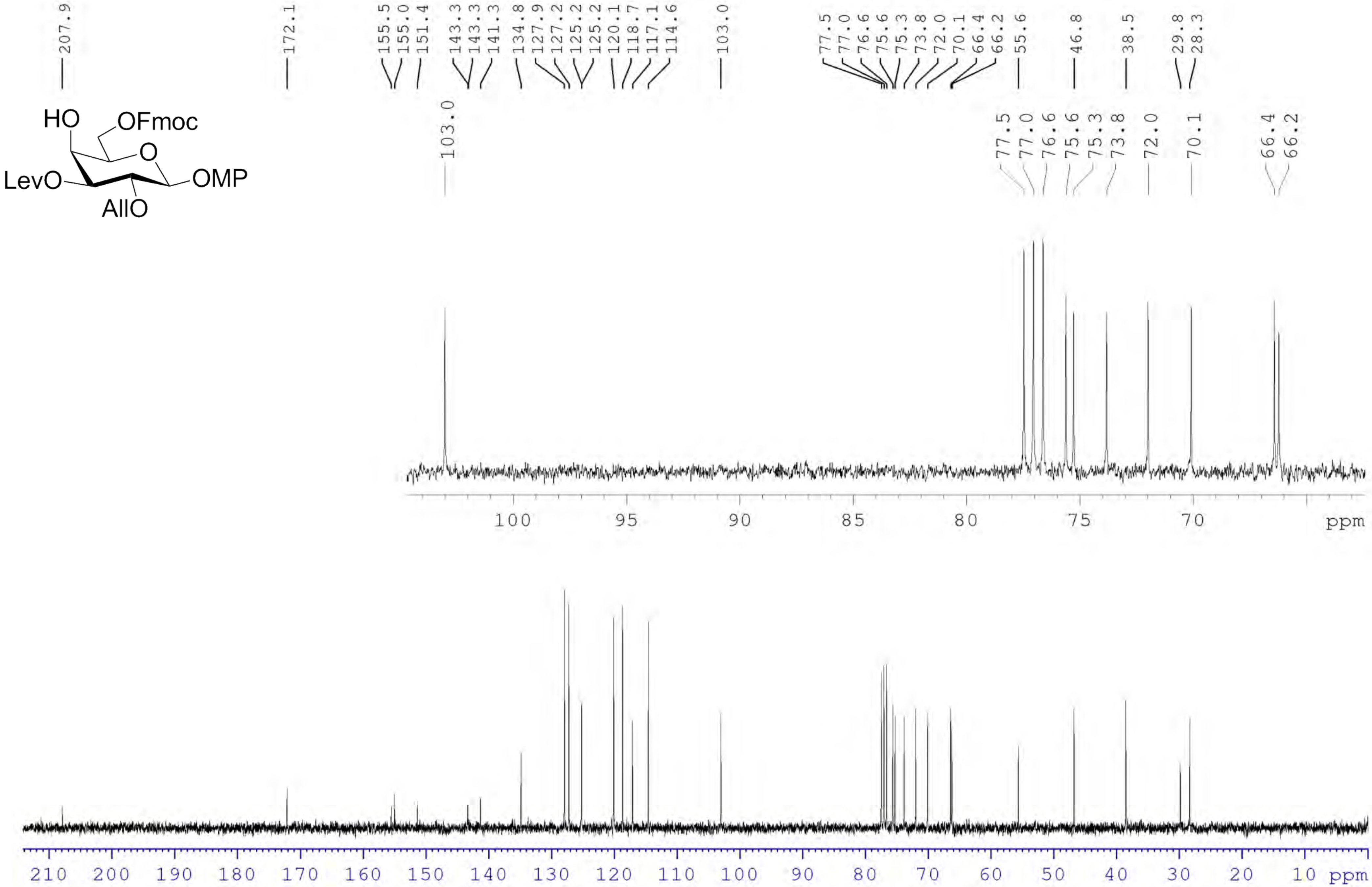

^{1}H-NMR of **11** (300 MHz, $CDCl_3$)

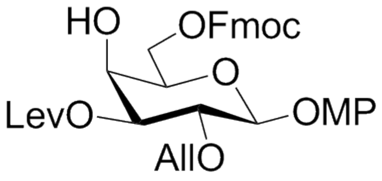

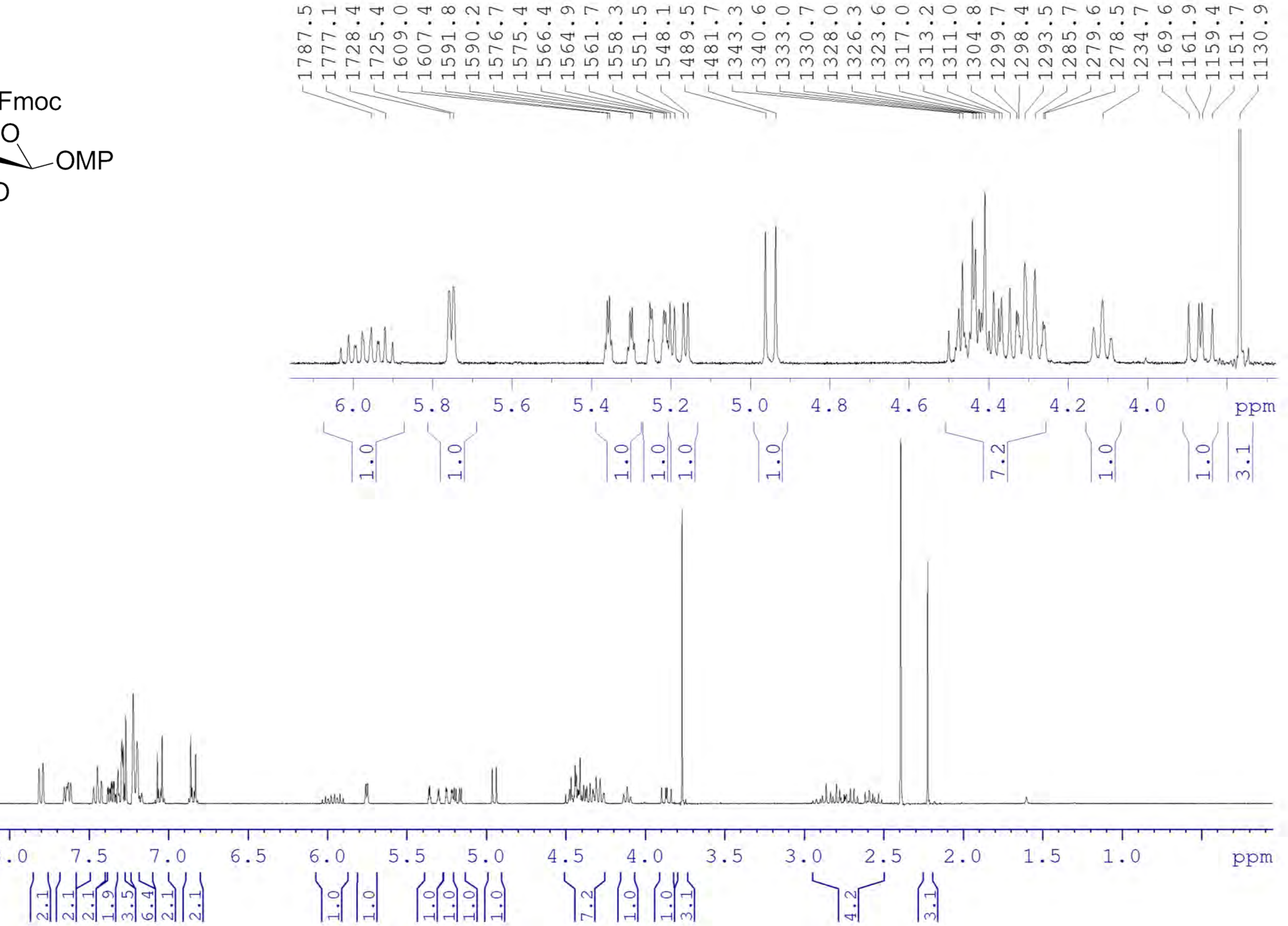

^{13}C-NMR of **11** (75 MHz, $CDCl_3$)

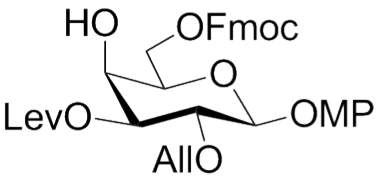

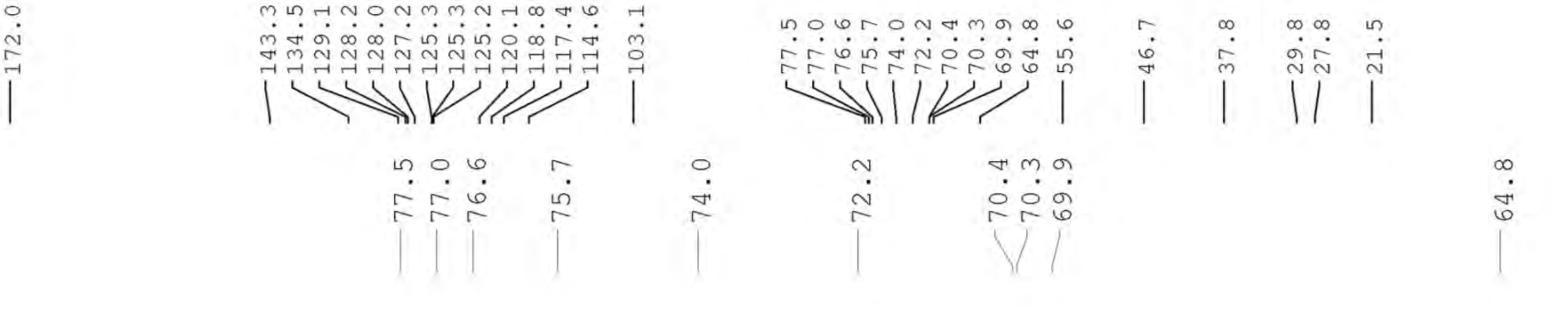
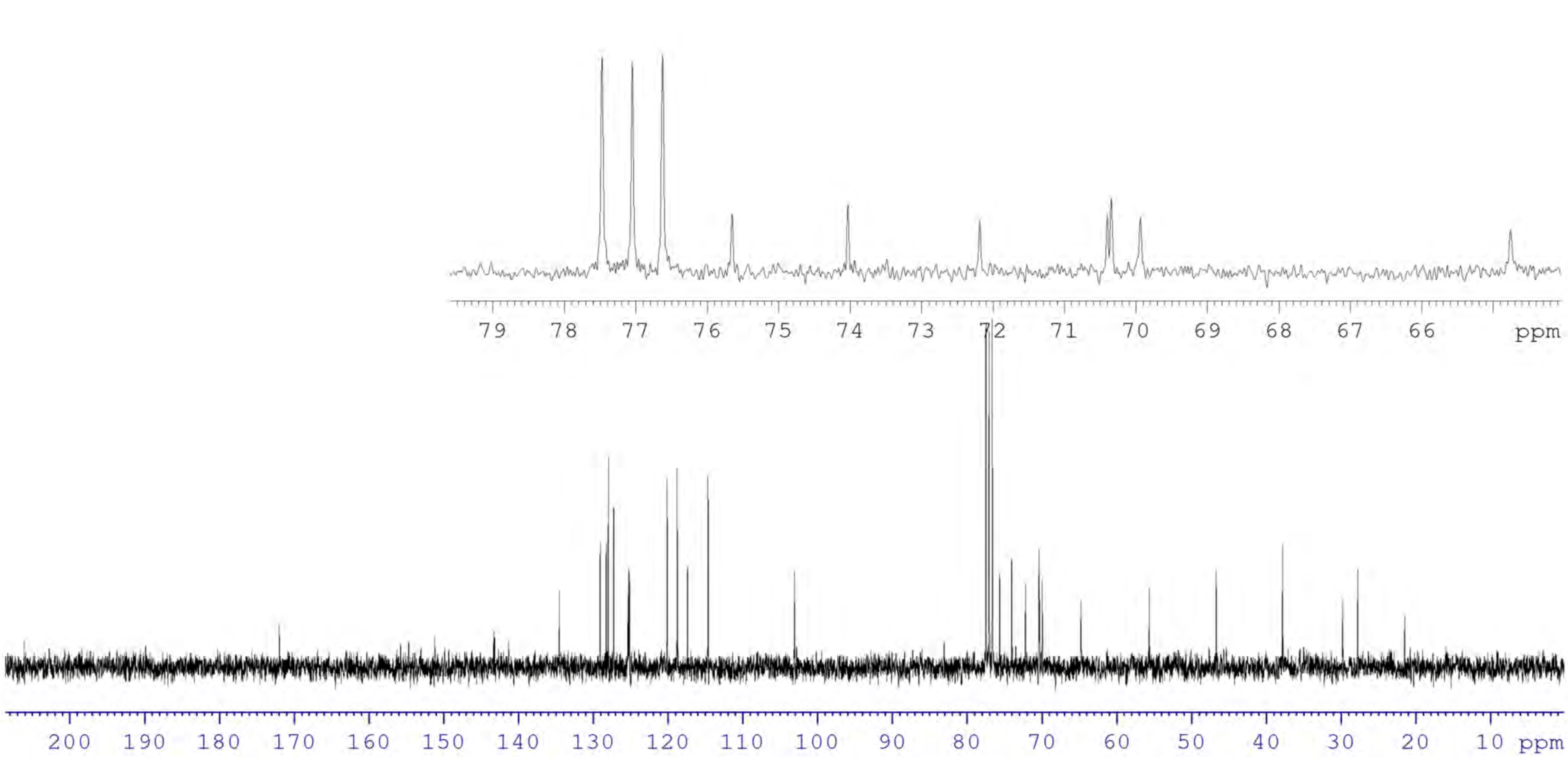

^{1}H-NMR of **12** (300 MHz, $CDCl_3$)

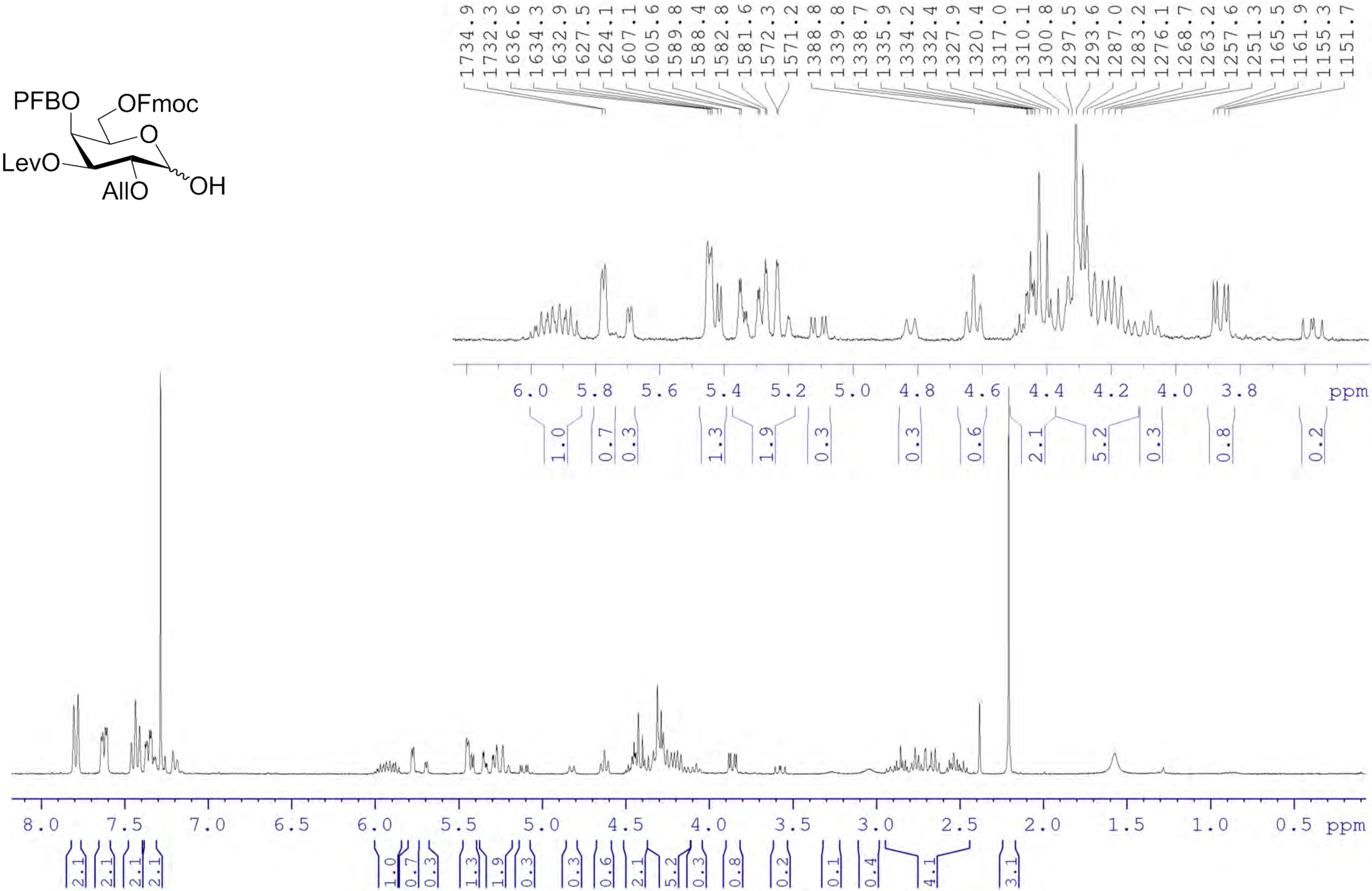

^{13}C-NMR of **12** (75 MHz, $CDCl_3$)

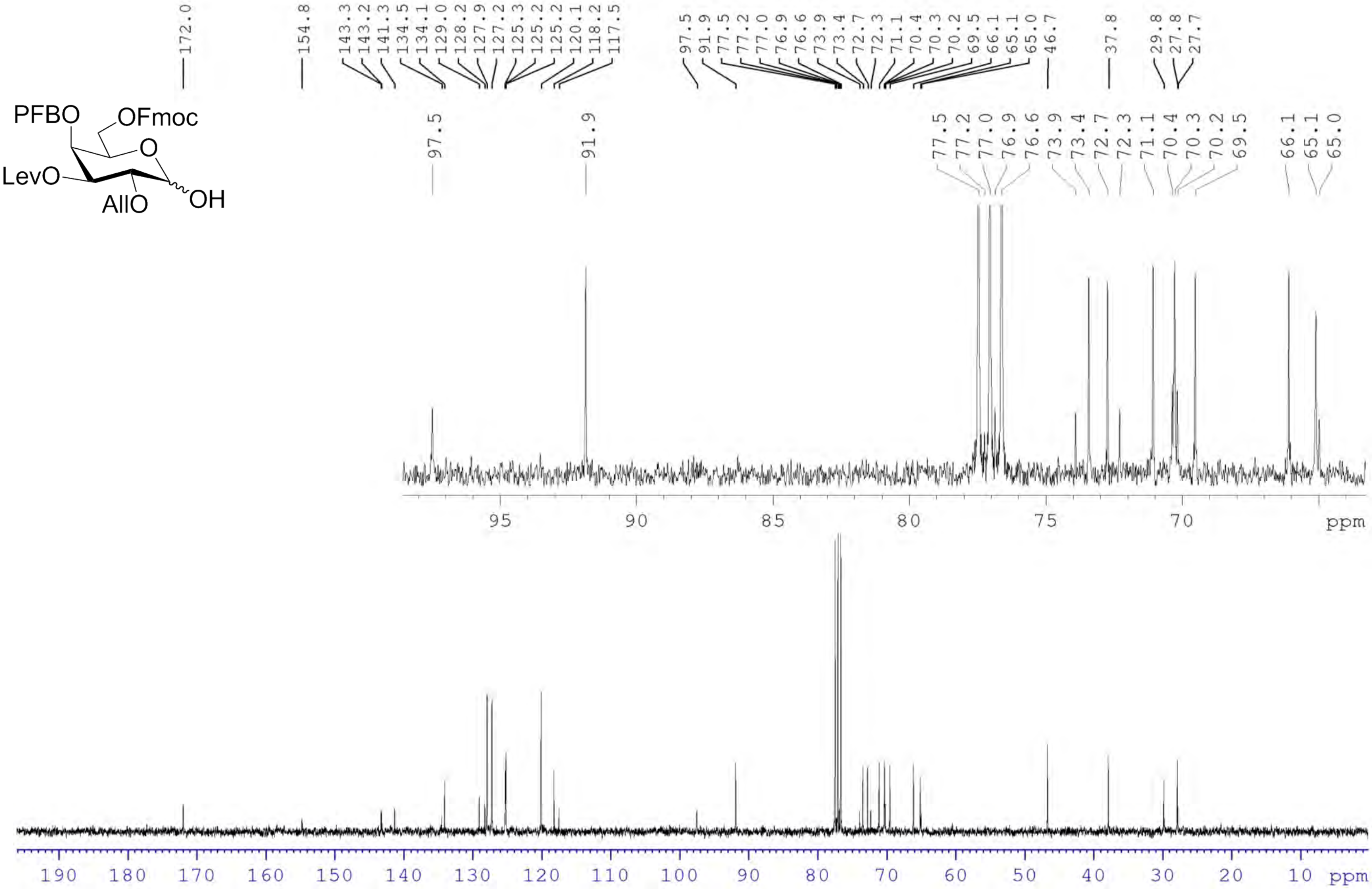

^{1}H-NMR of **13** (600 MHz, $CDCl_3$, 323K)

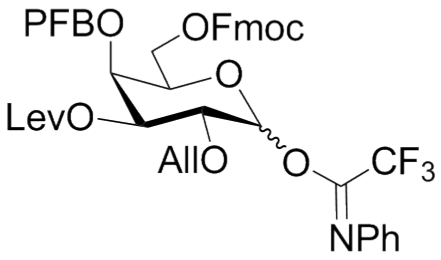

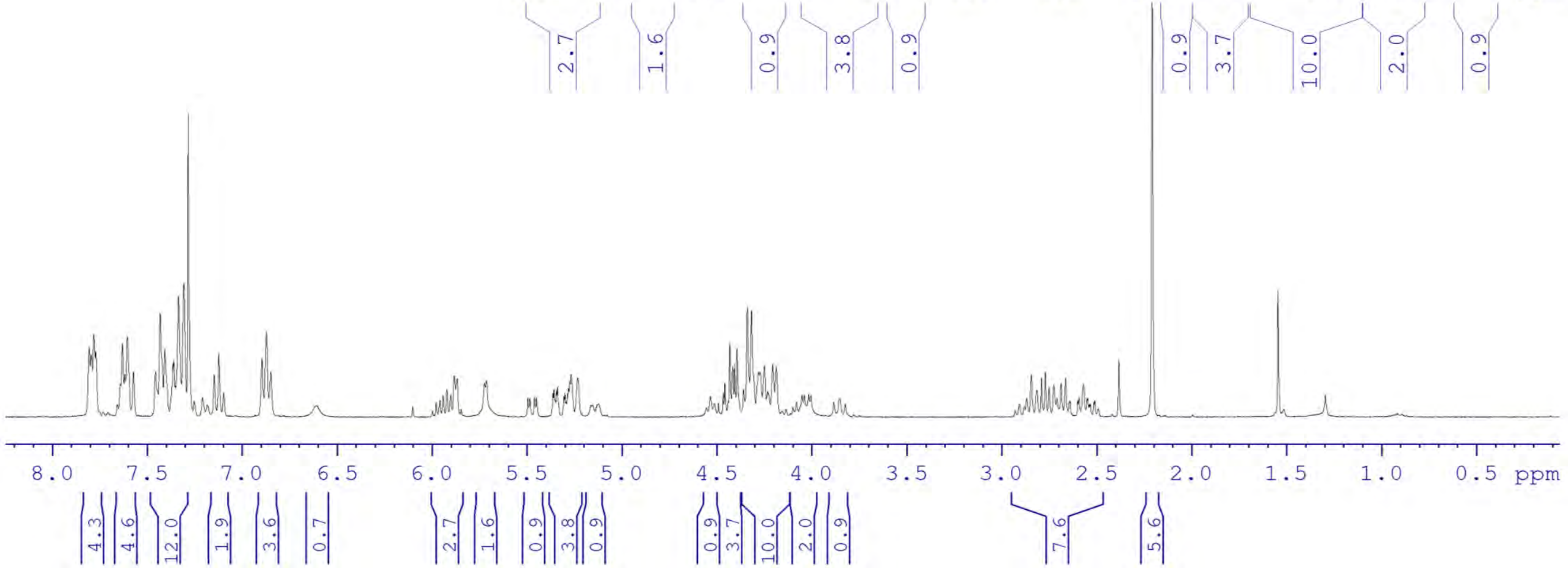

^{13}C-NMR of **13** (150 MHz, $CDCl_3$, 323K)

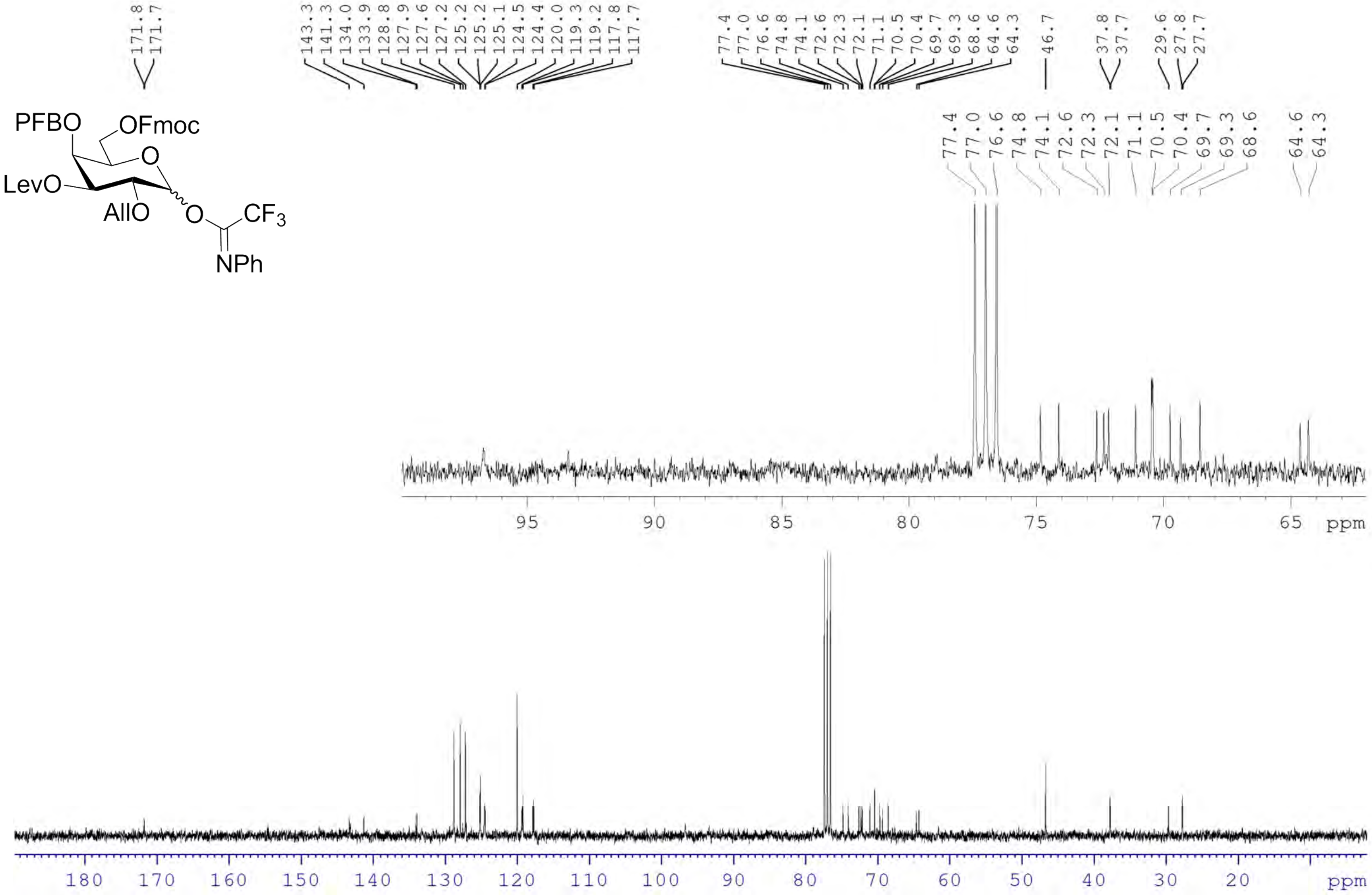

^{1}H-NMR of **9** (300 MHz, $CDCl_3$)

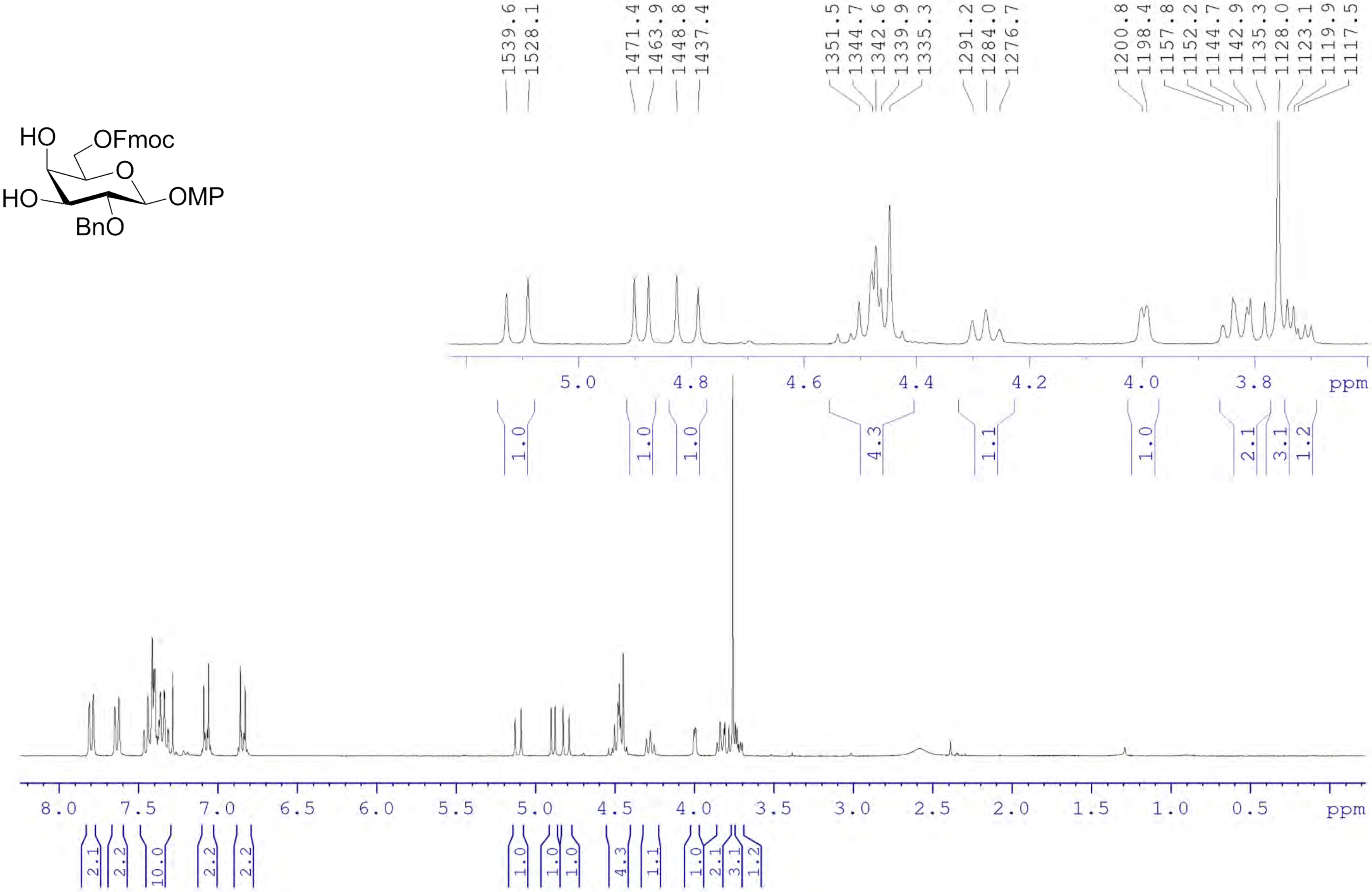

^{13}C-NMR of **9** (75 MHz, $CDCl_3$)

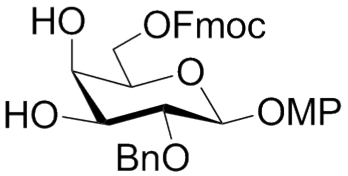

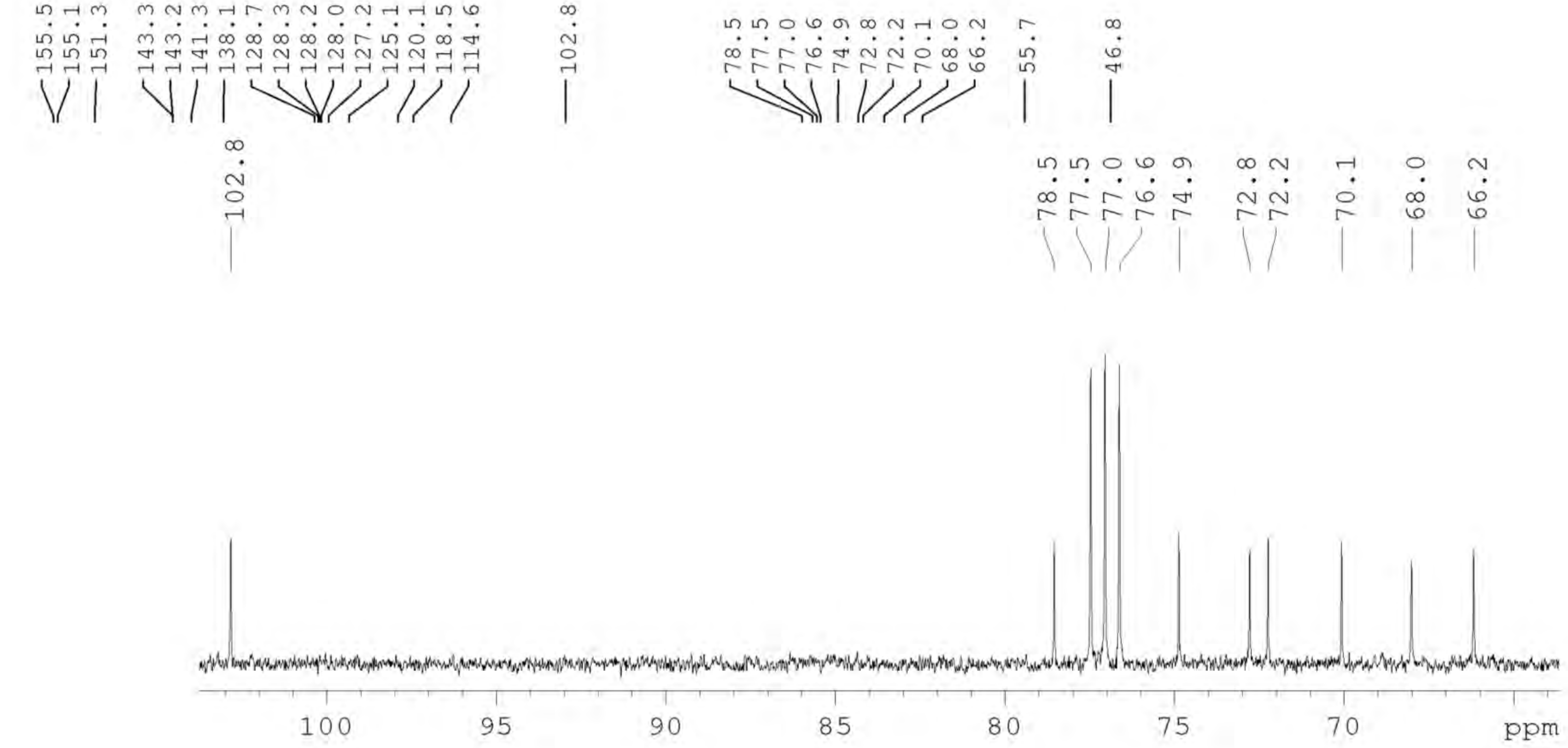

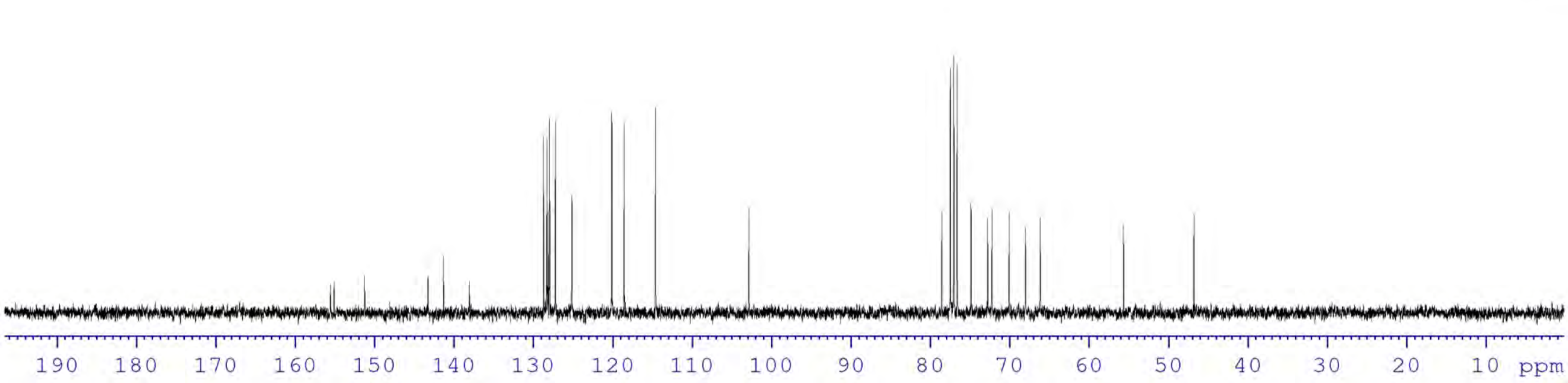

^{1}H-NMR of **14** (600 MHz, $CDCl_3$)

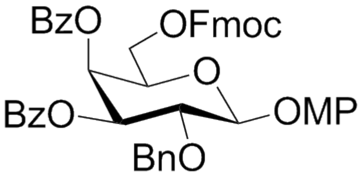

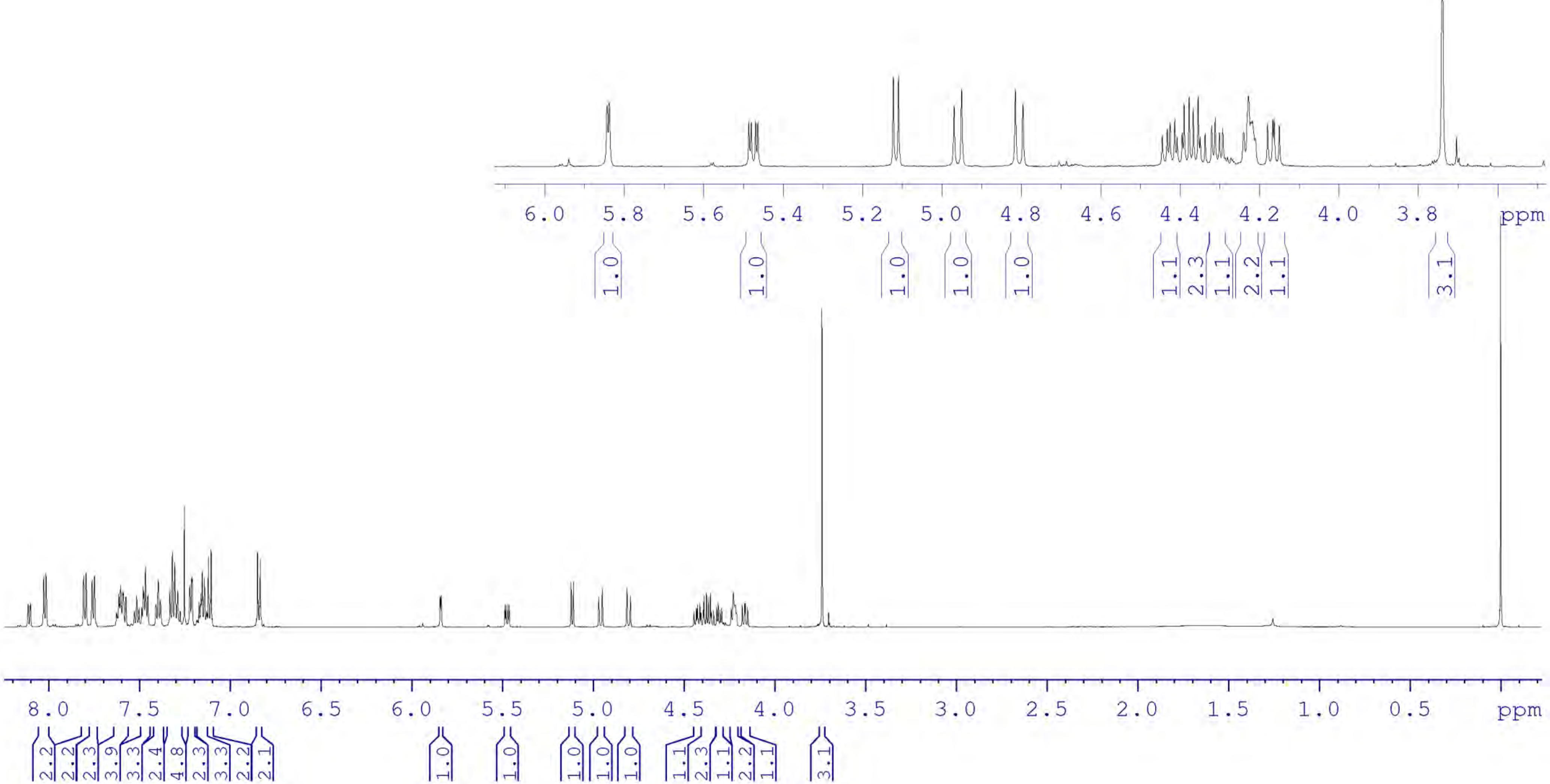

^{13}C-NMR of **14** (150 MHz, $CDCl_3$)

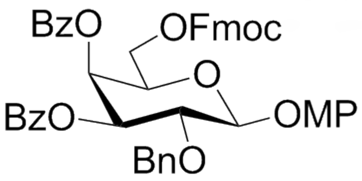

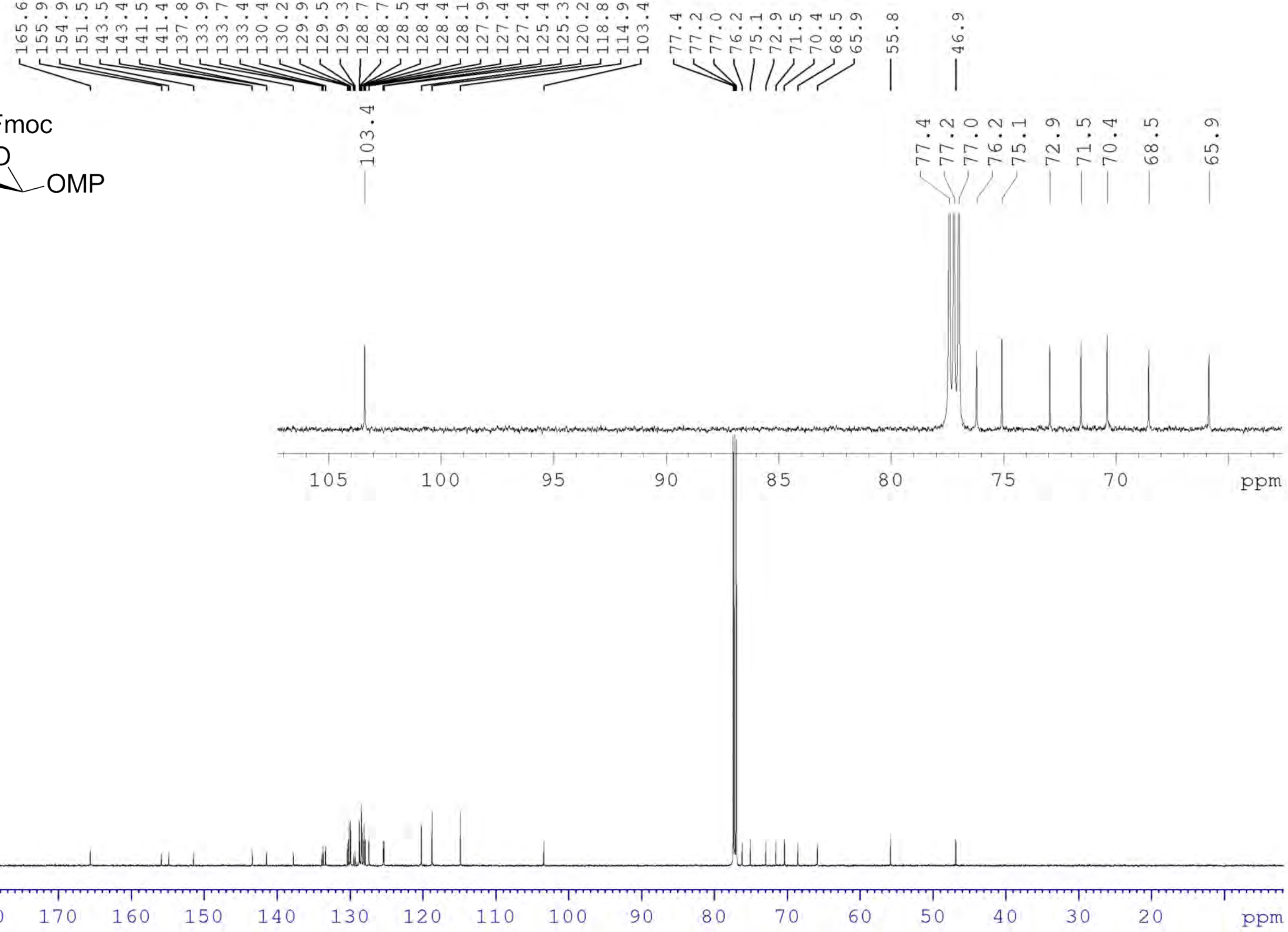

^{1}H-NMR of **15** (300 MHz, $CDCl_3$)

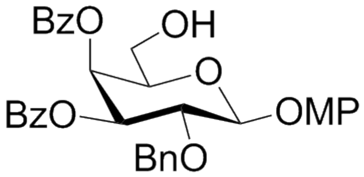

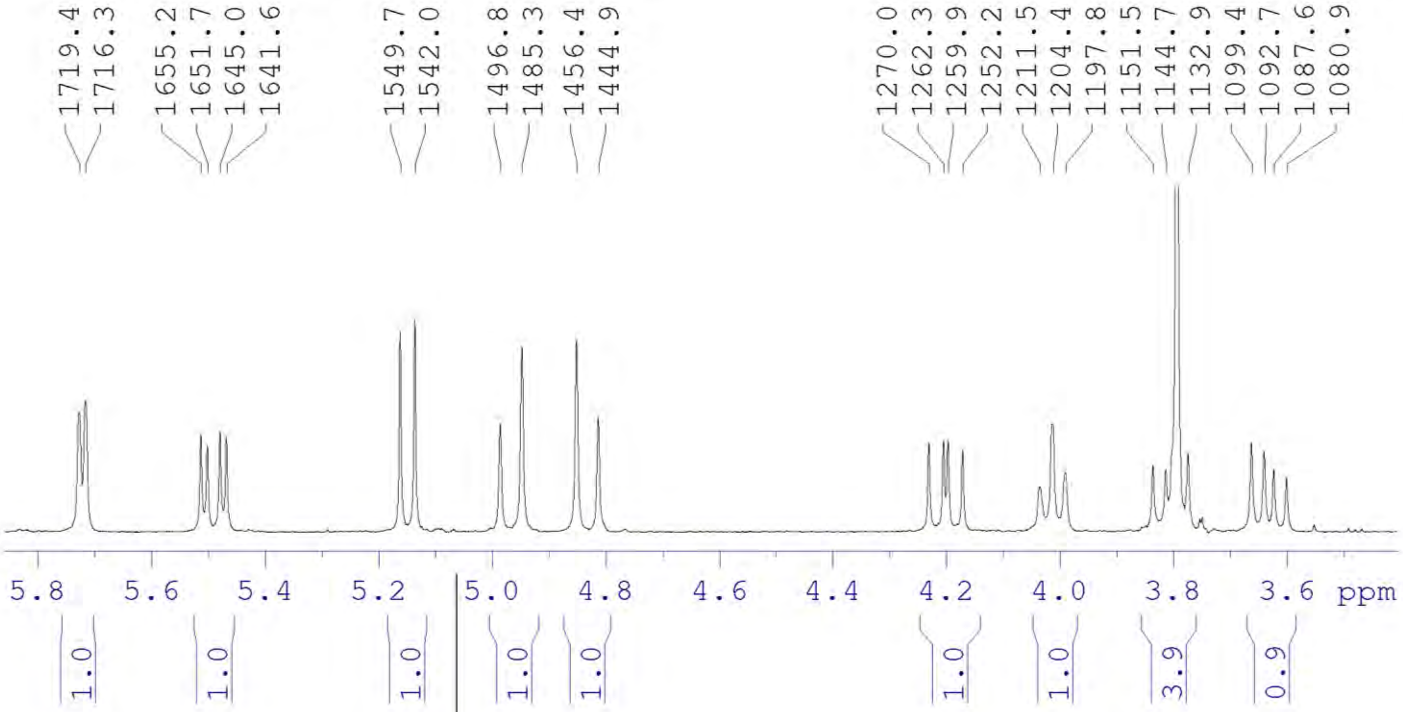

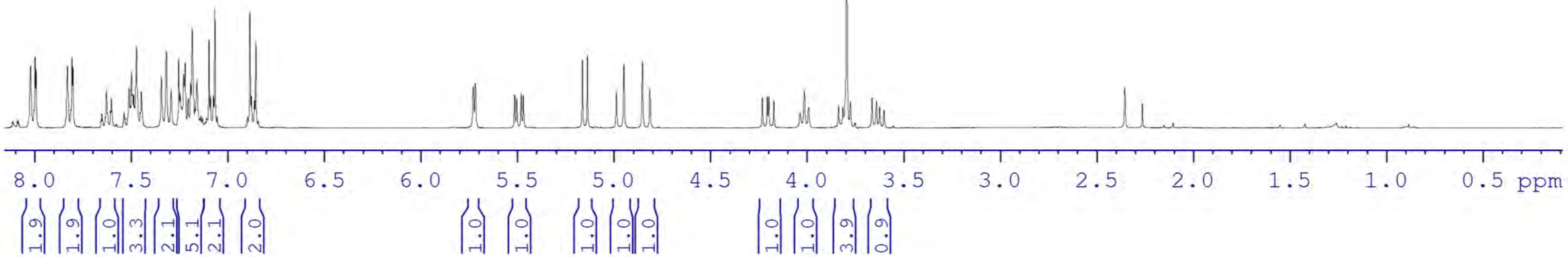

^{13}C-NMR of **15** (75 MHz, $CDCl_3$)

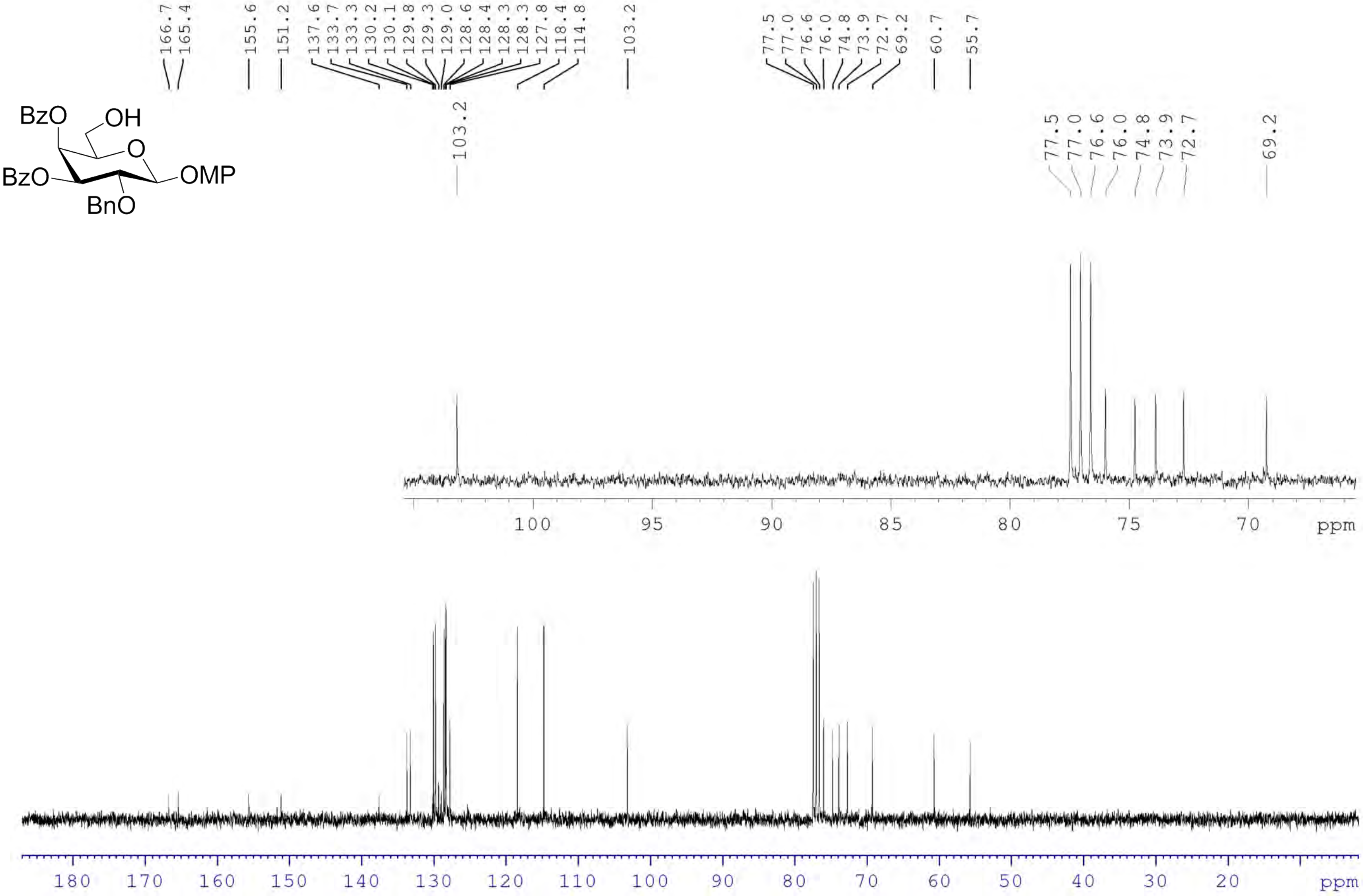

^{1}H-NMR of **16** (300 MHz, $CDCl_3$)

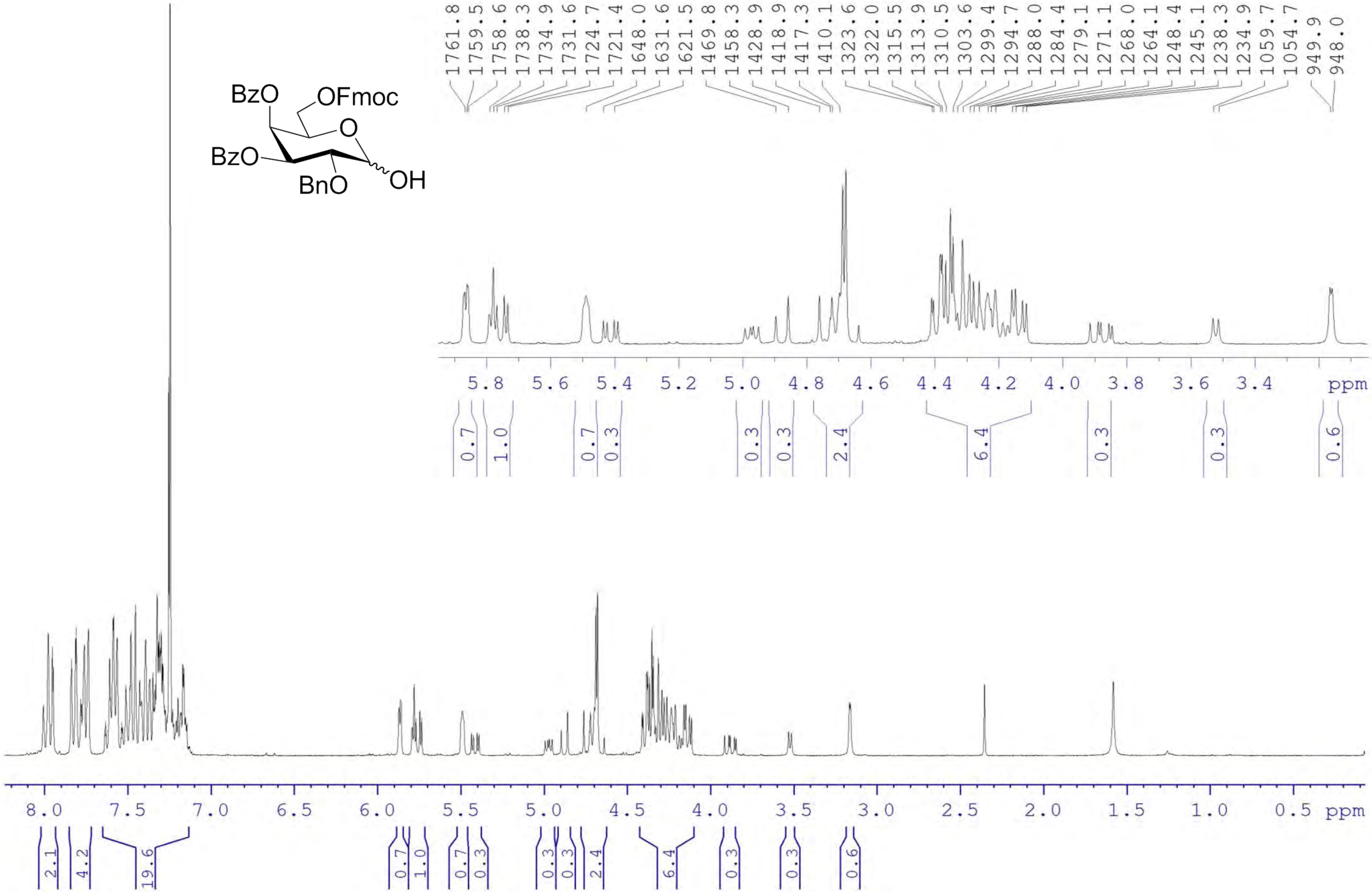

^{13}C-NMR of **16** (75 MHz, $CDCl_3$)

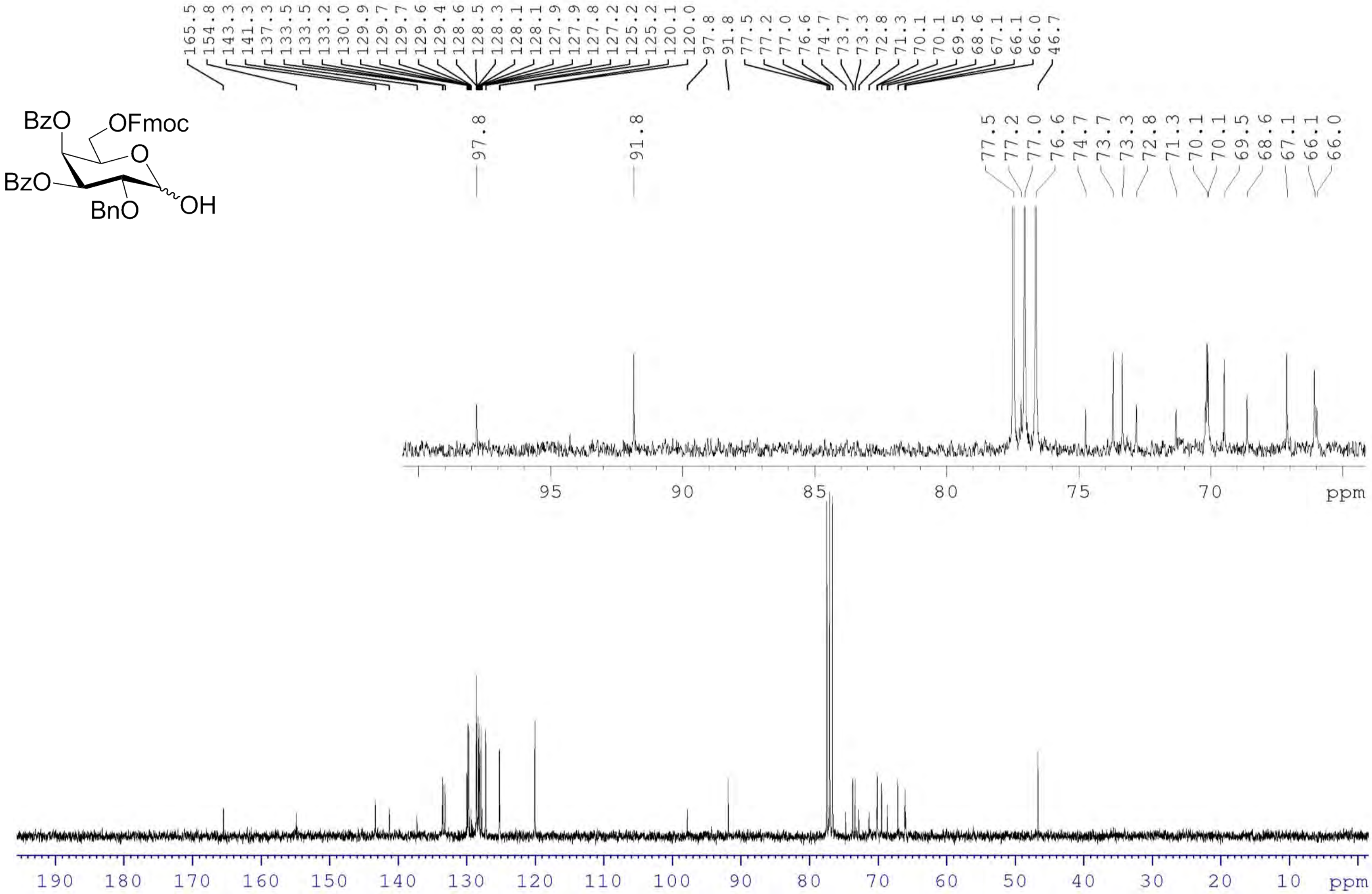

^{1}H-NMR of **17** (300 MHz, $CDCl_3$, 323 K)

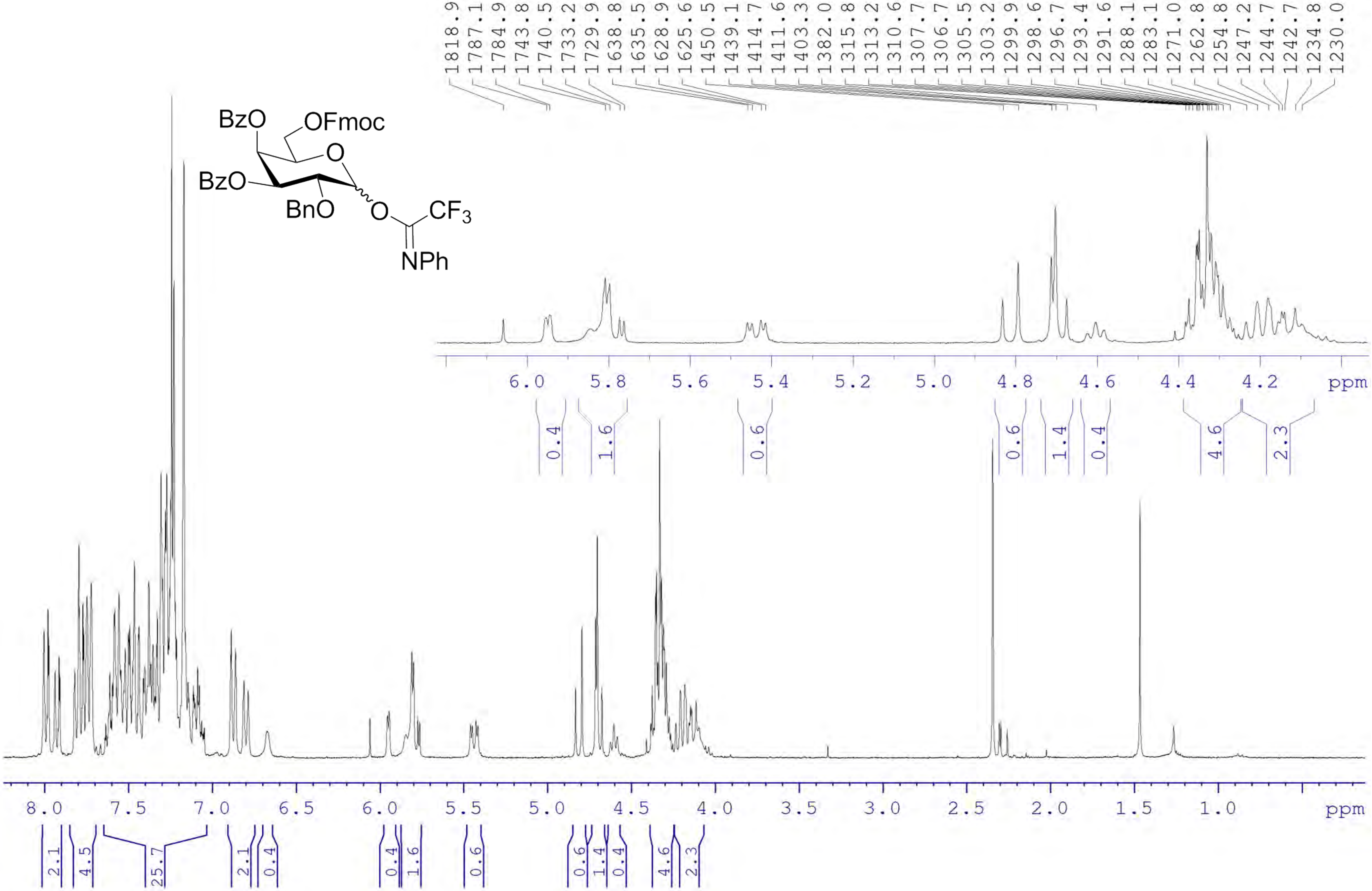

^{13}C-NMR of **17** (75 MHz, $CDCl_3$, 323 K)

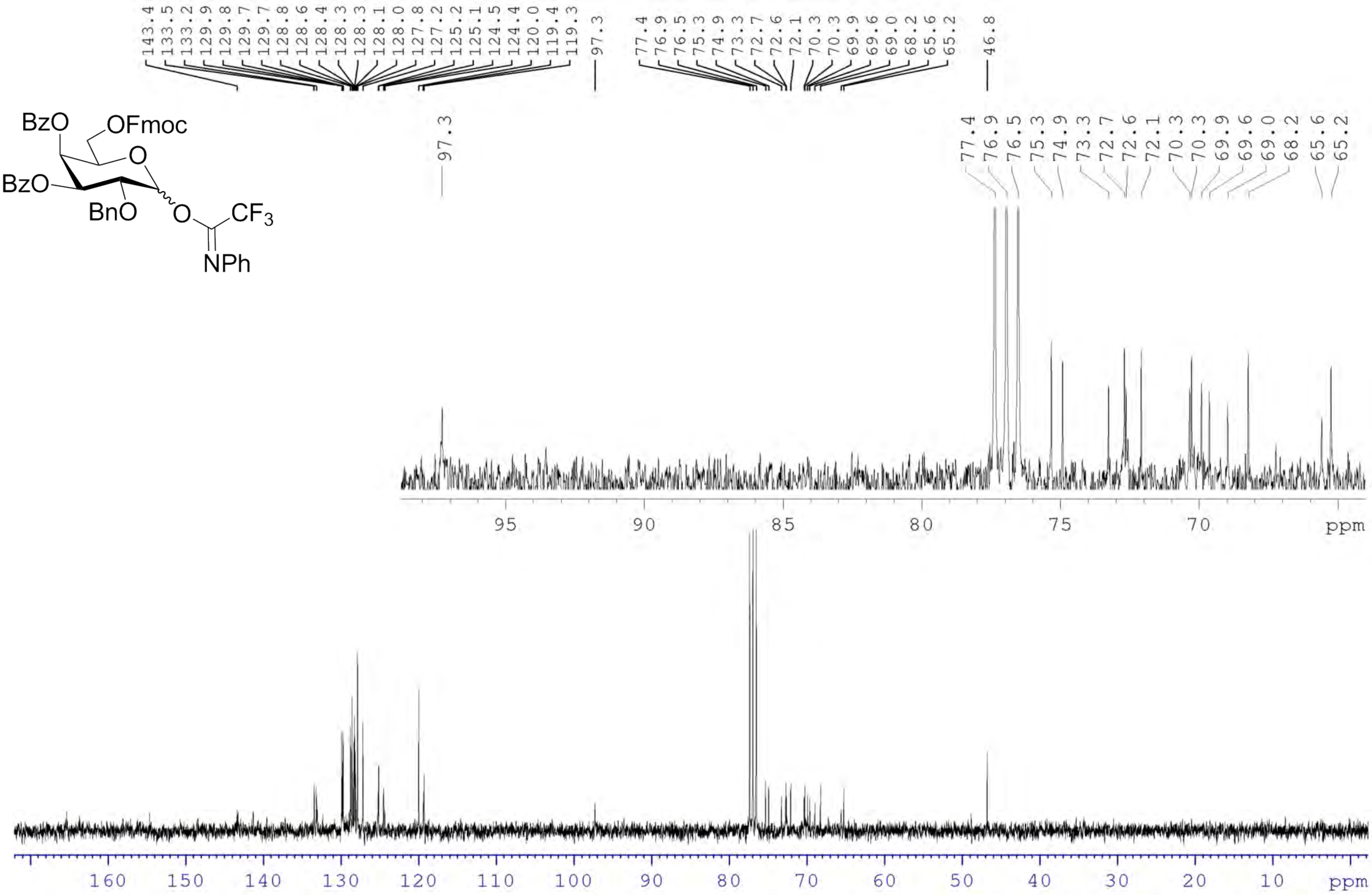

^{1}H-NMR of **18** (300 MHz, $CDCl_3$)

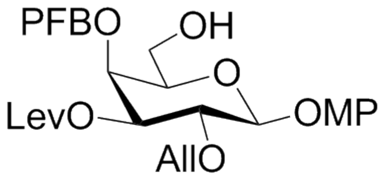

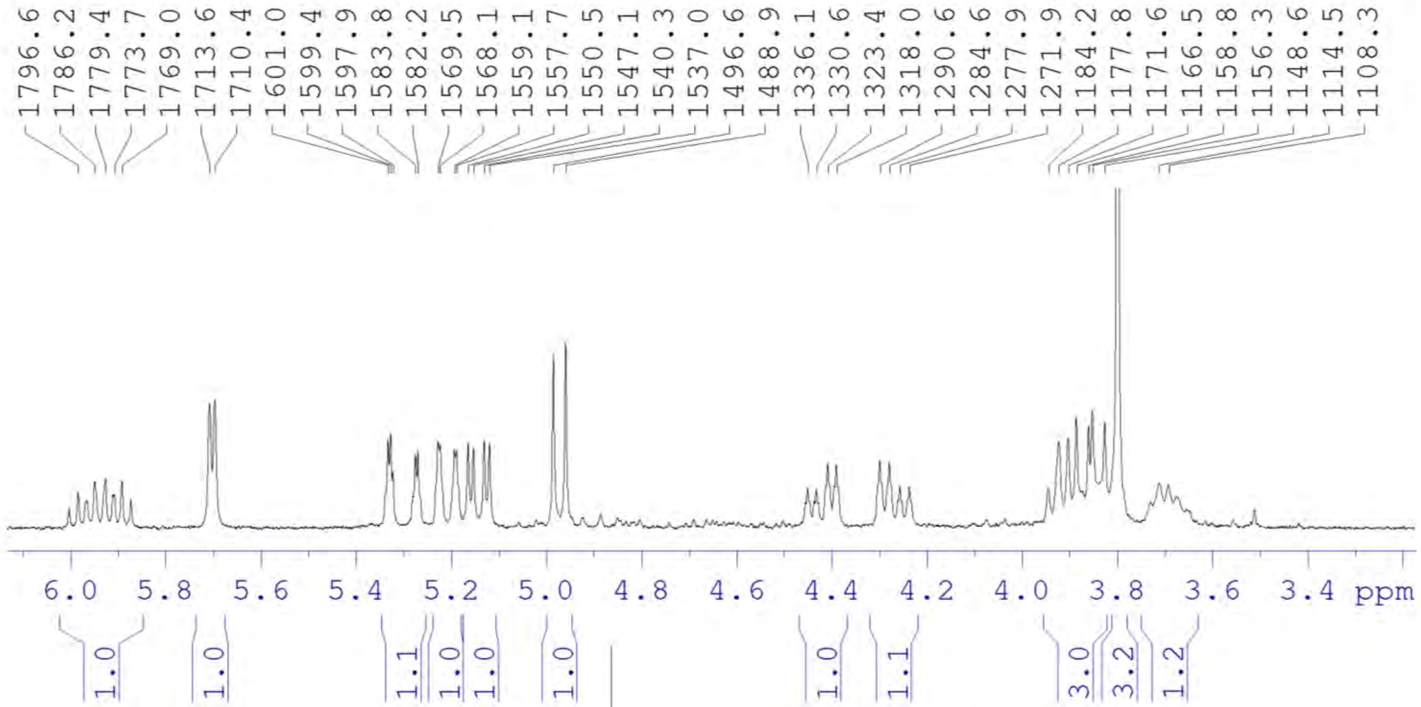

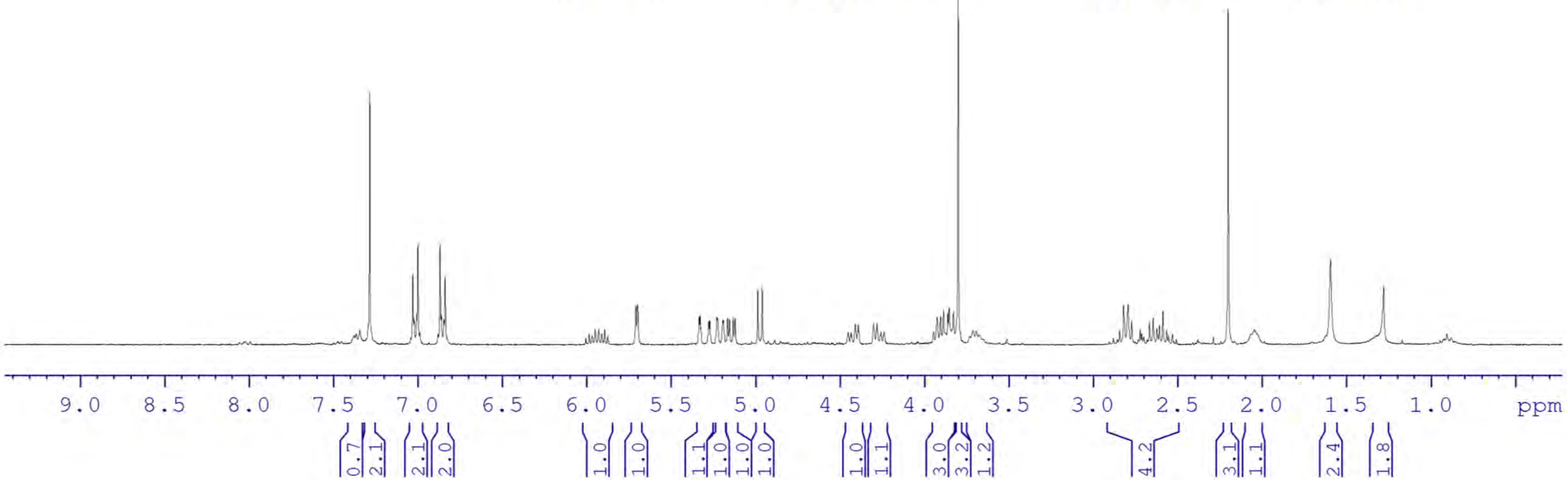

^{13}C-NMR of **18** (75 MHz, $CDCl_3$)

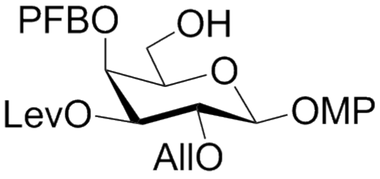

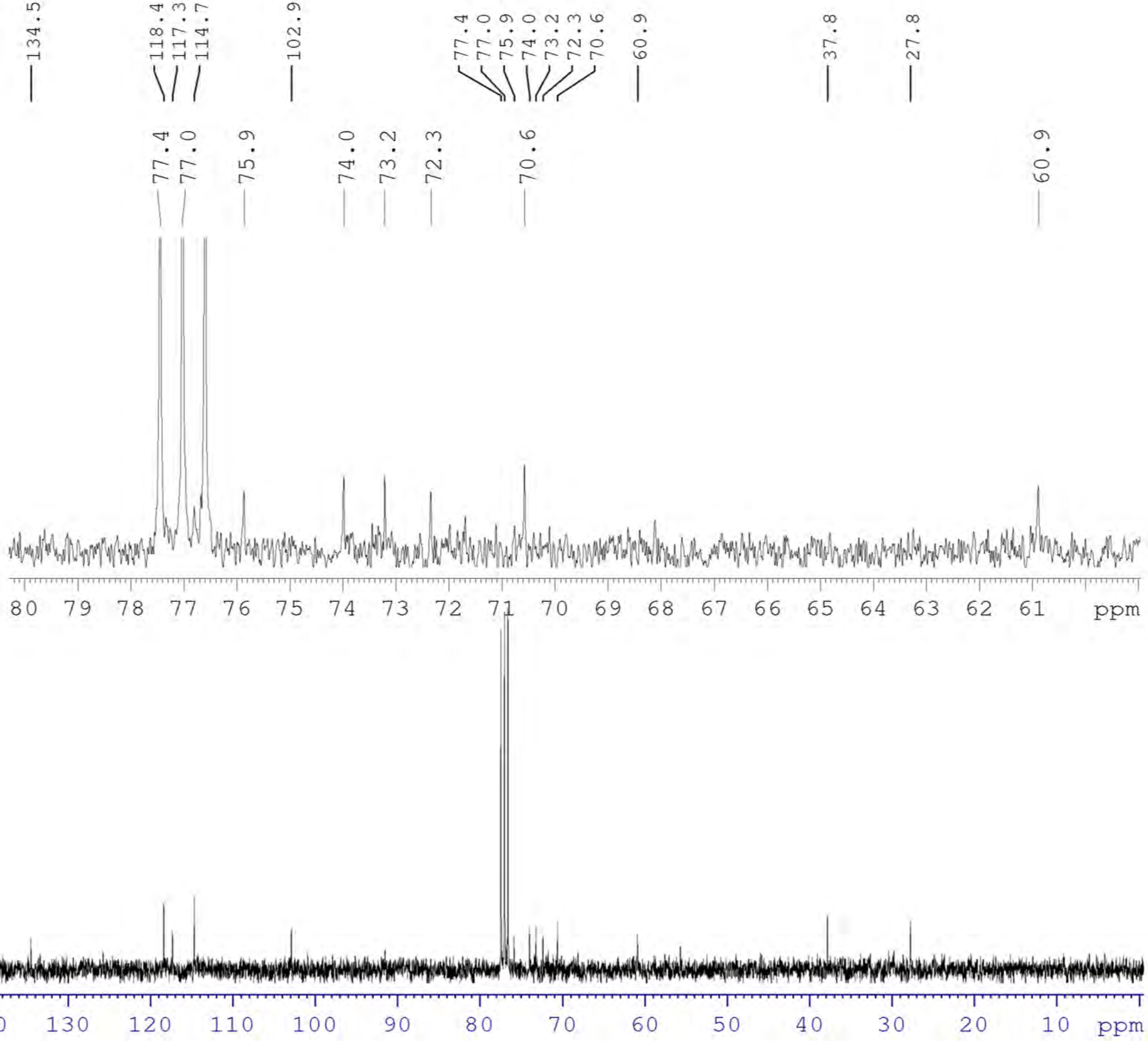

^{1}H-NMR of **19** (300 MHz, $CDCl_3$)

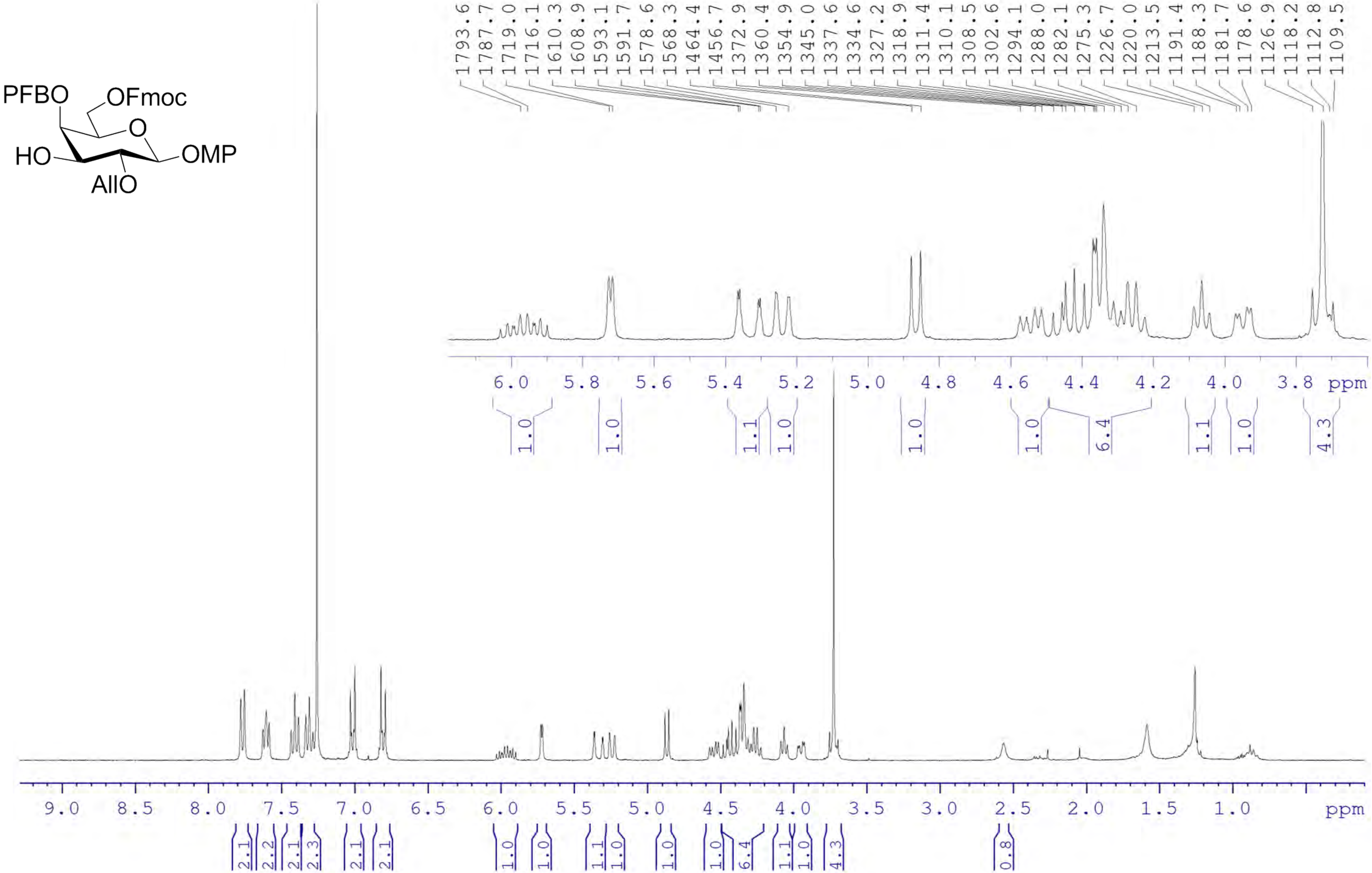

^{13}C-NMR of **19** (75 MHz, $CDCl_3$)

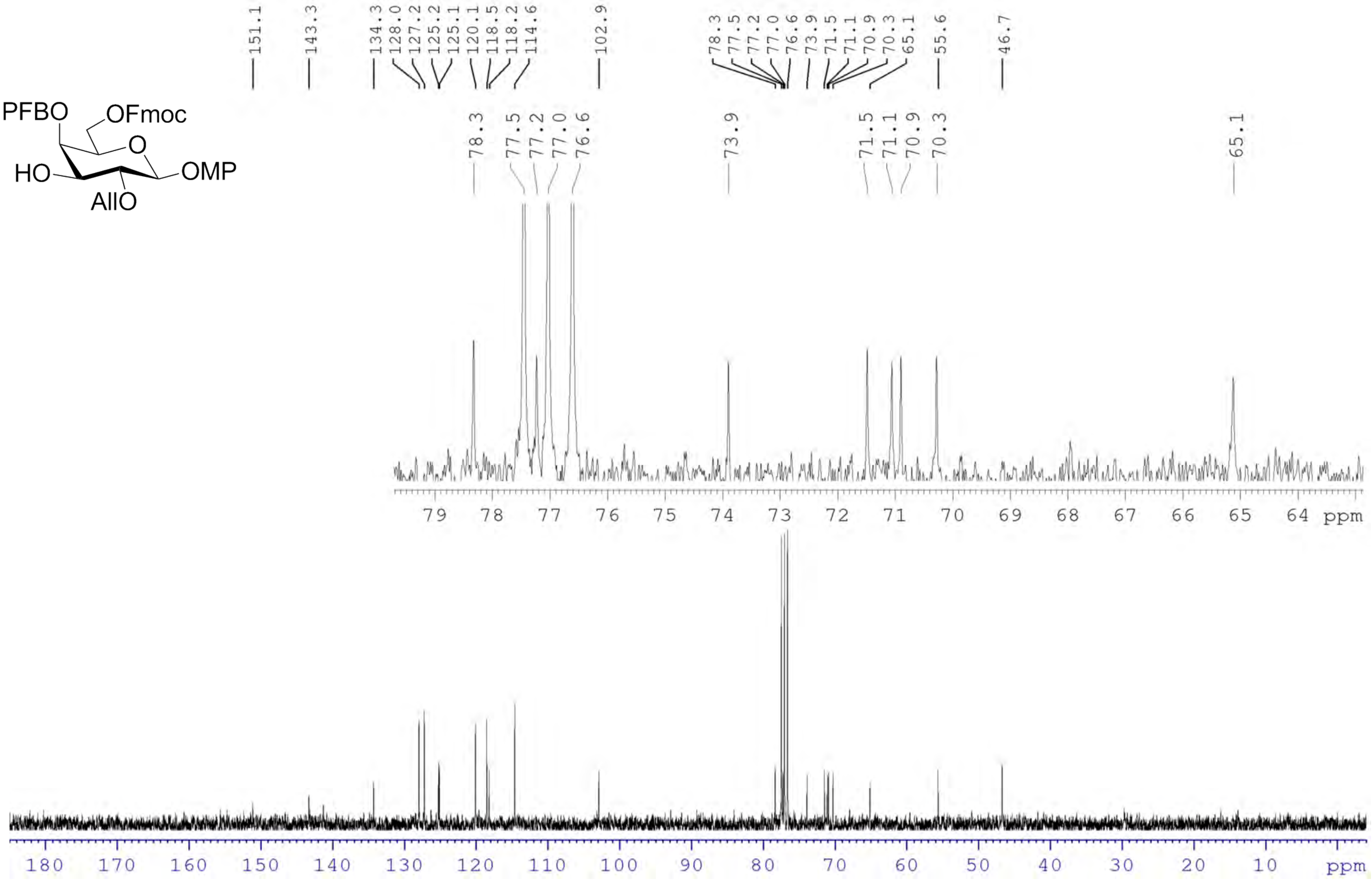

^{1}H-NMR of **20** (400 MHz, $CDCl_3$)

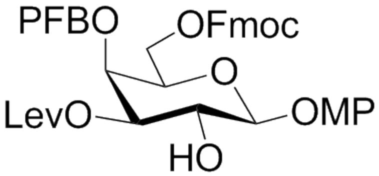

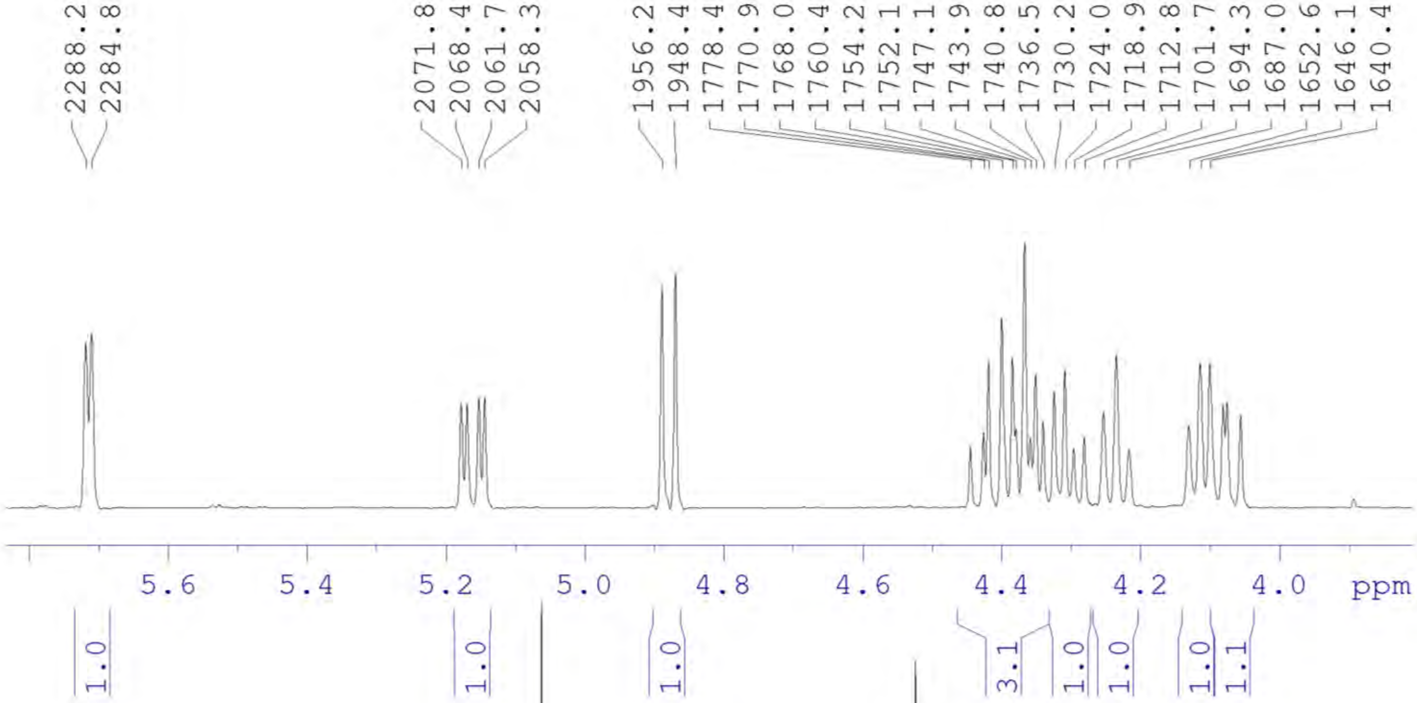

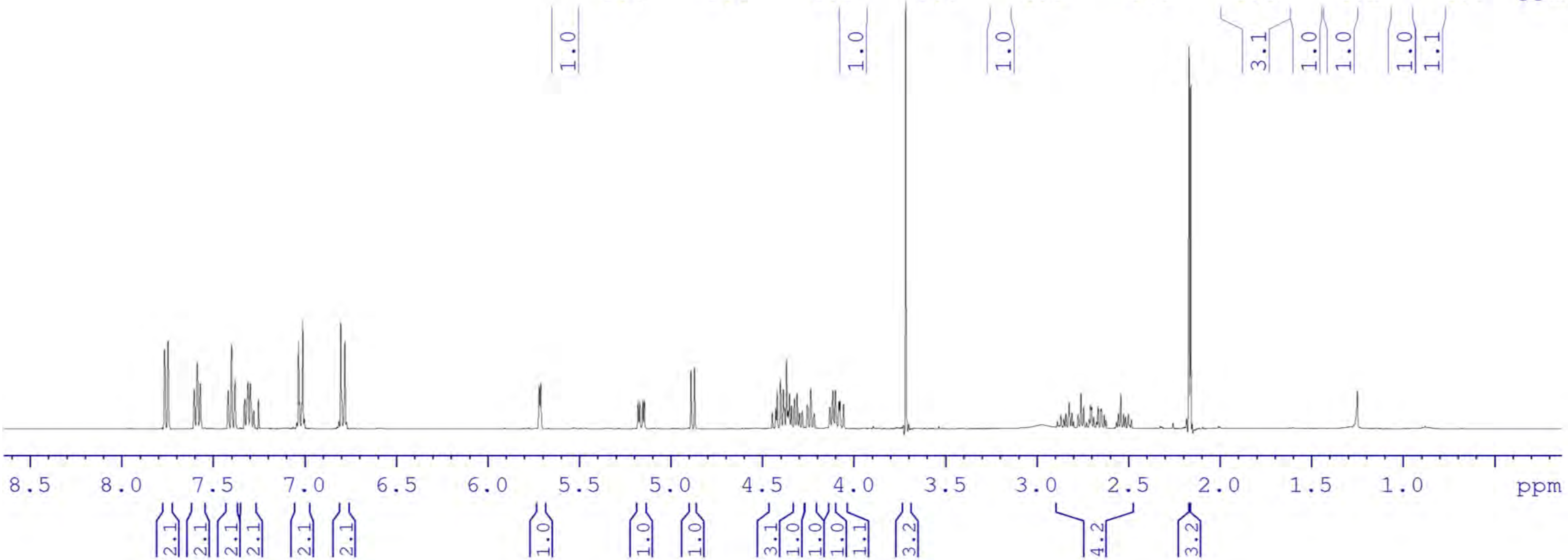

^{13}C-NMR of **20** (100 MHz, $CDCl_3$)

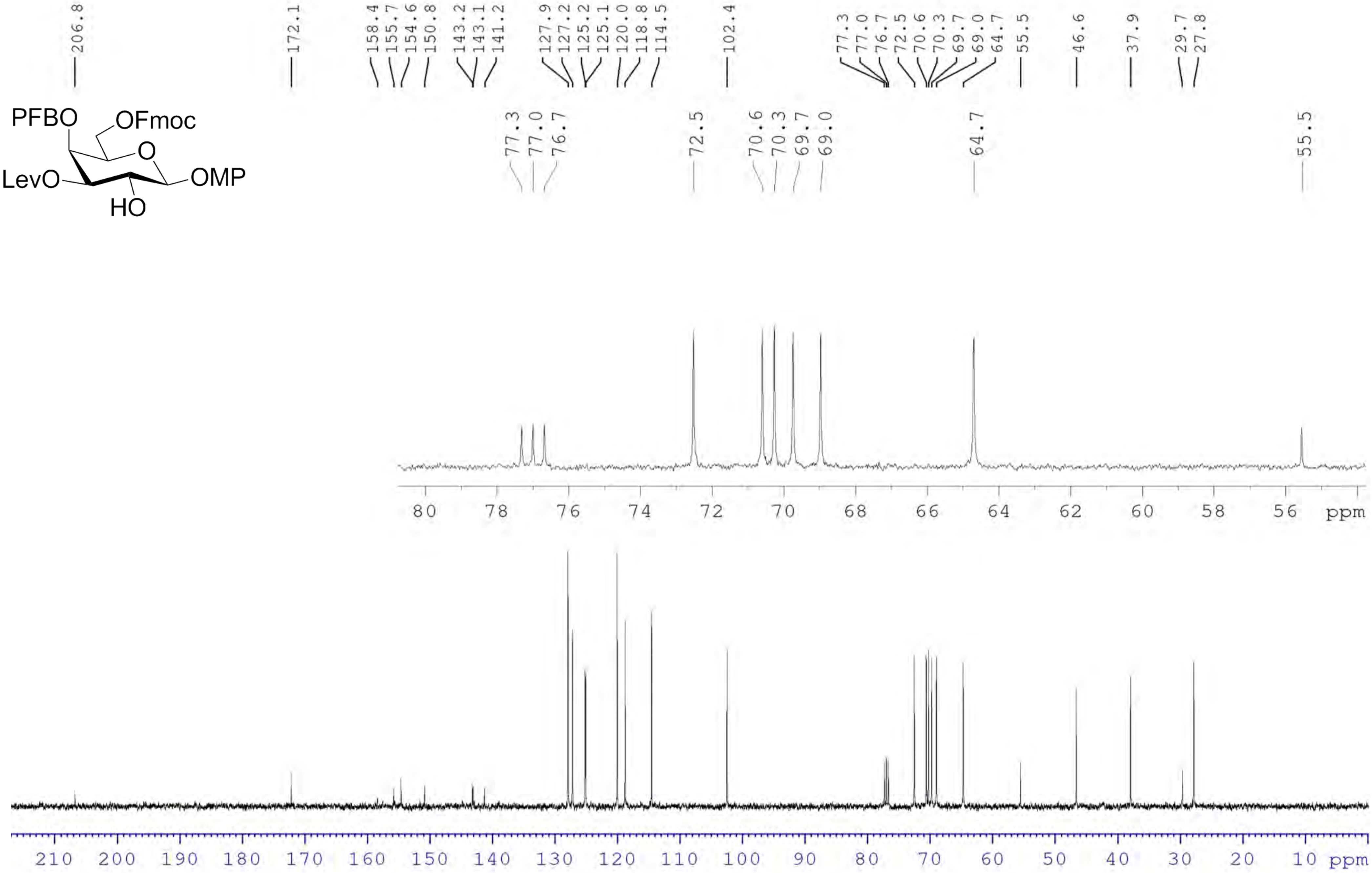

^{1}H-NMR of **S2** (400 MHz, $CDCl_3$)

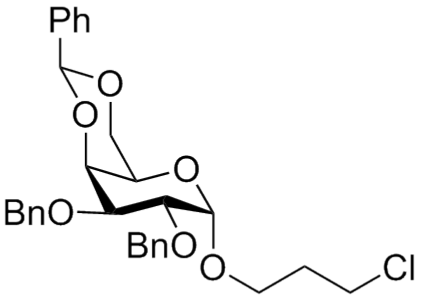

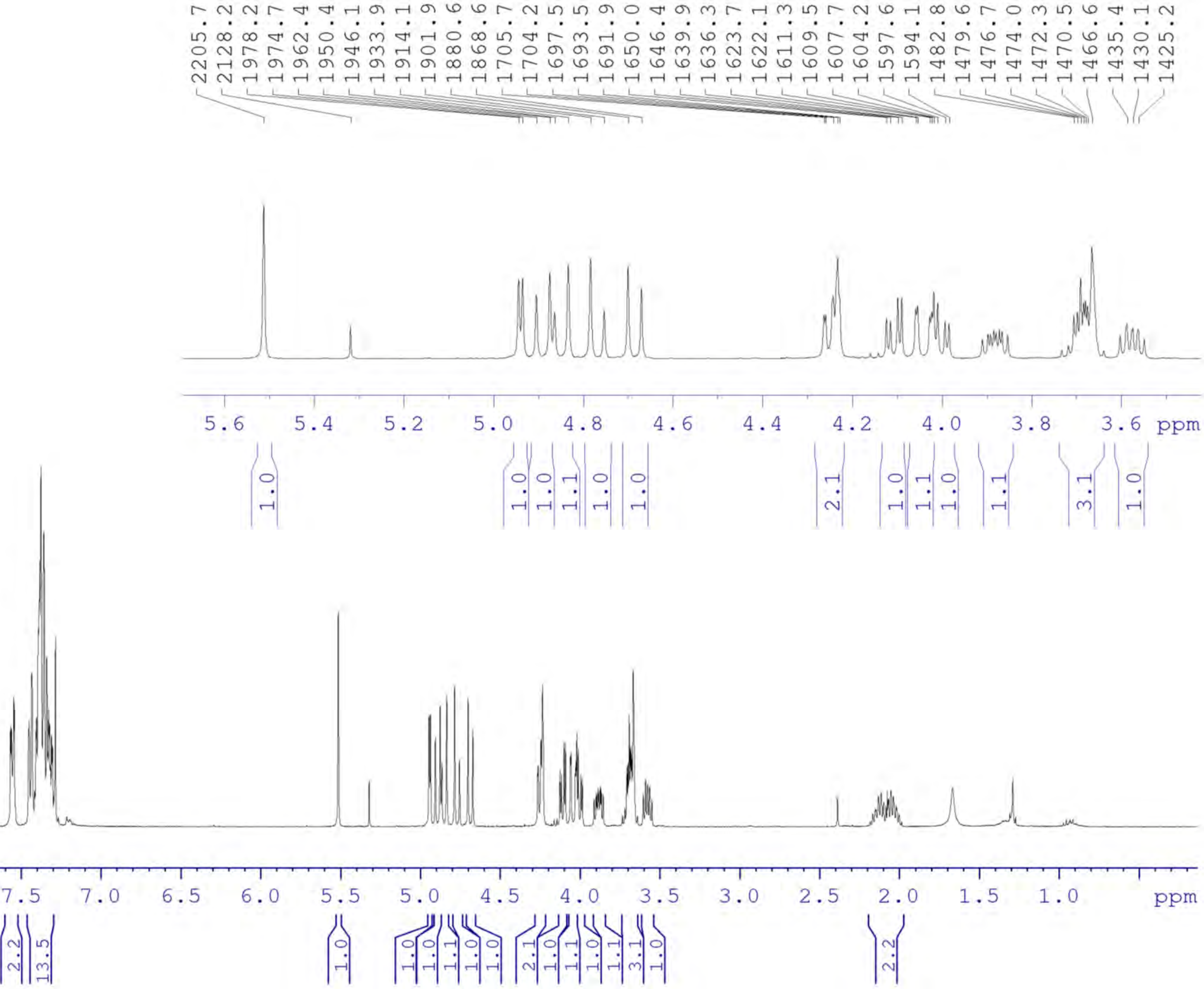

^{13}C-NMR of **S2** (100 MHz, $CDCl_3$)

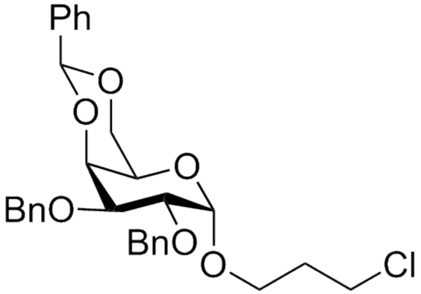

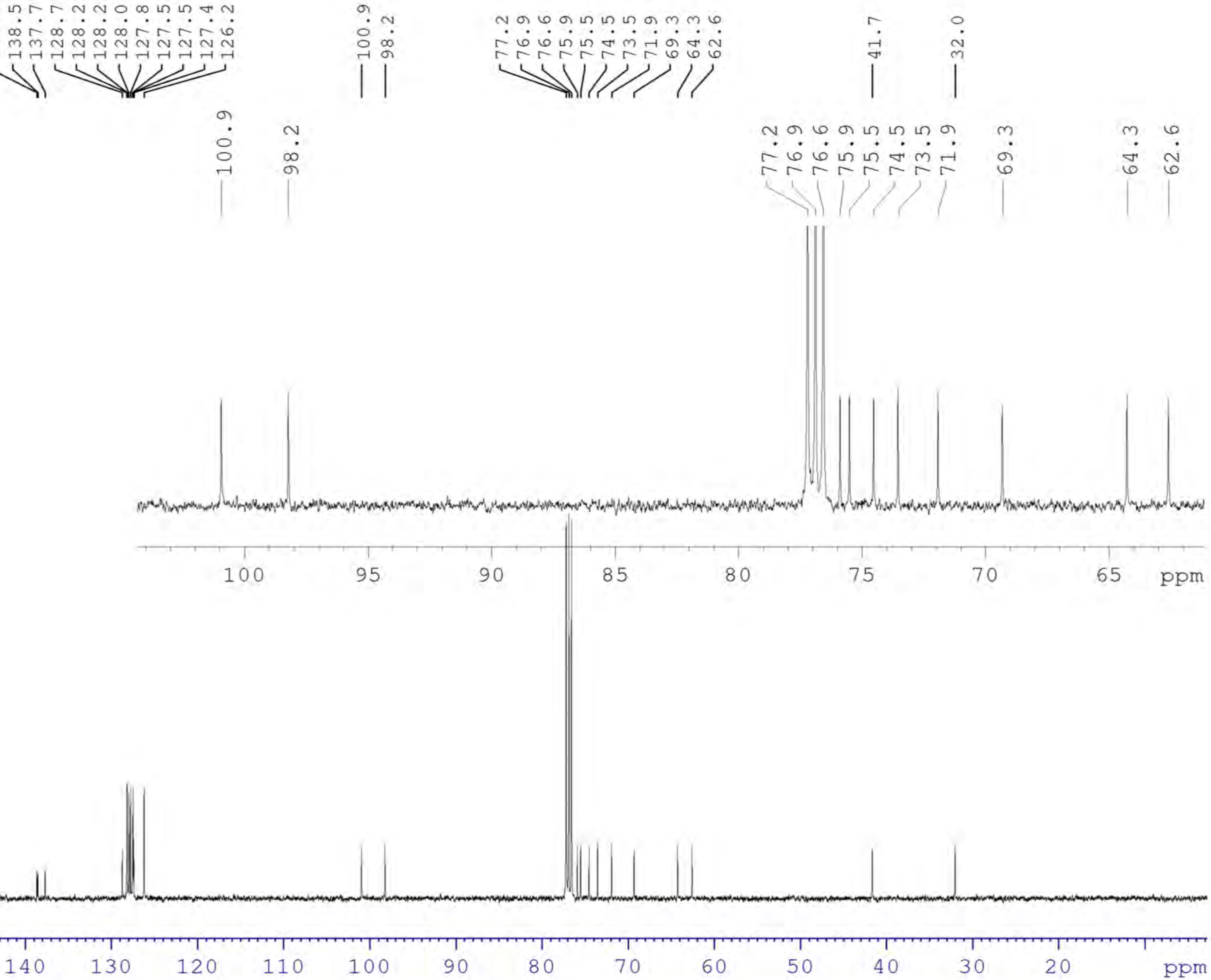

^{1}H-NMR of **S3** (500 MHz, $CDCl_3$, 323K)

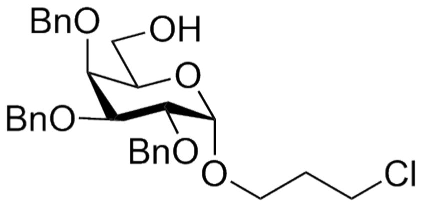

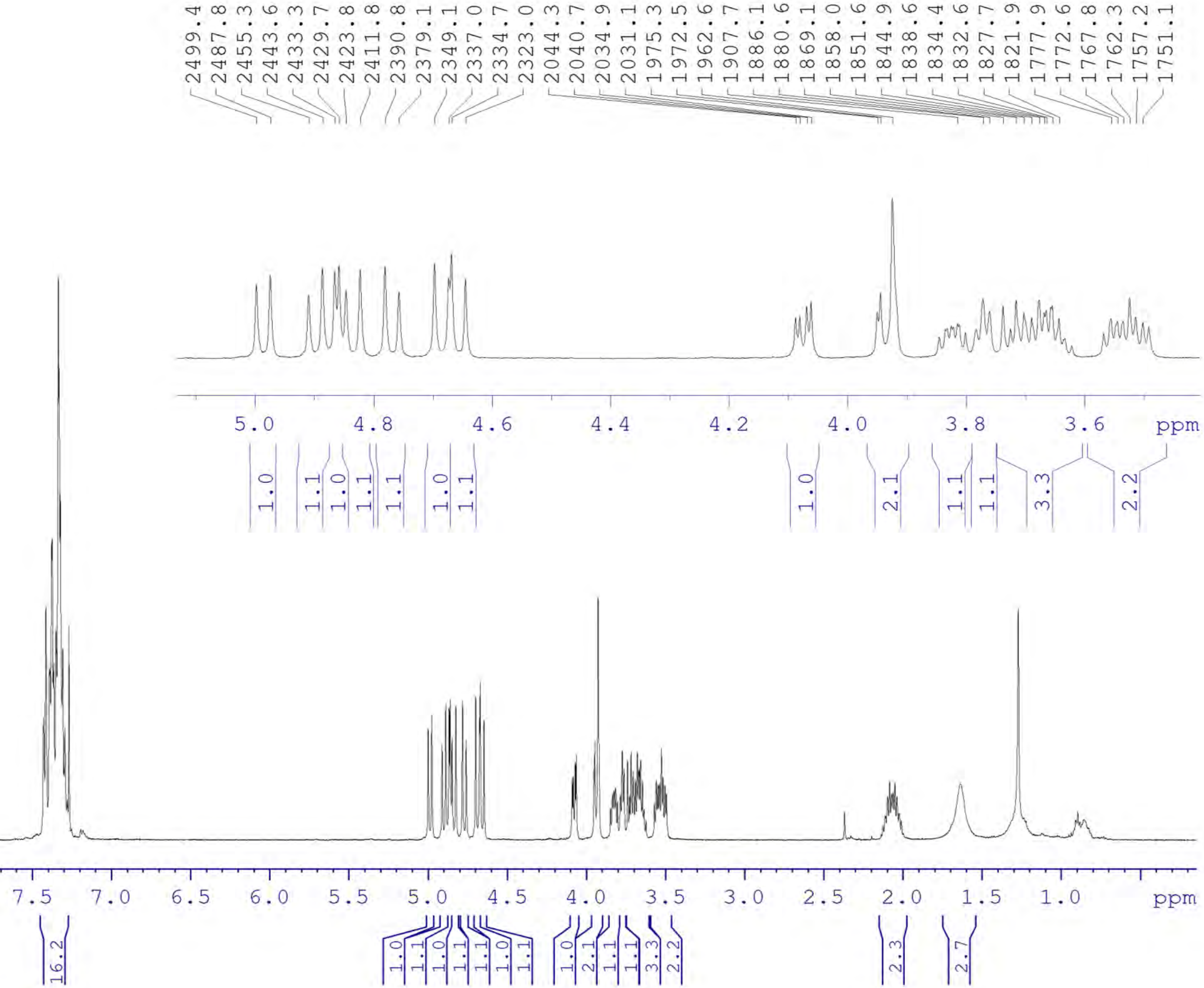

^{13}C-NMR of **S3** (100 MHz, $CDCl_3$)

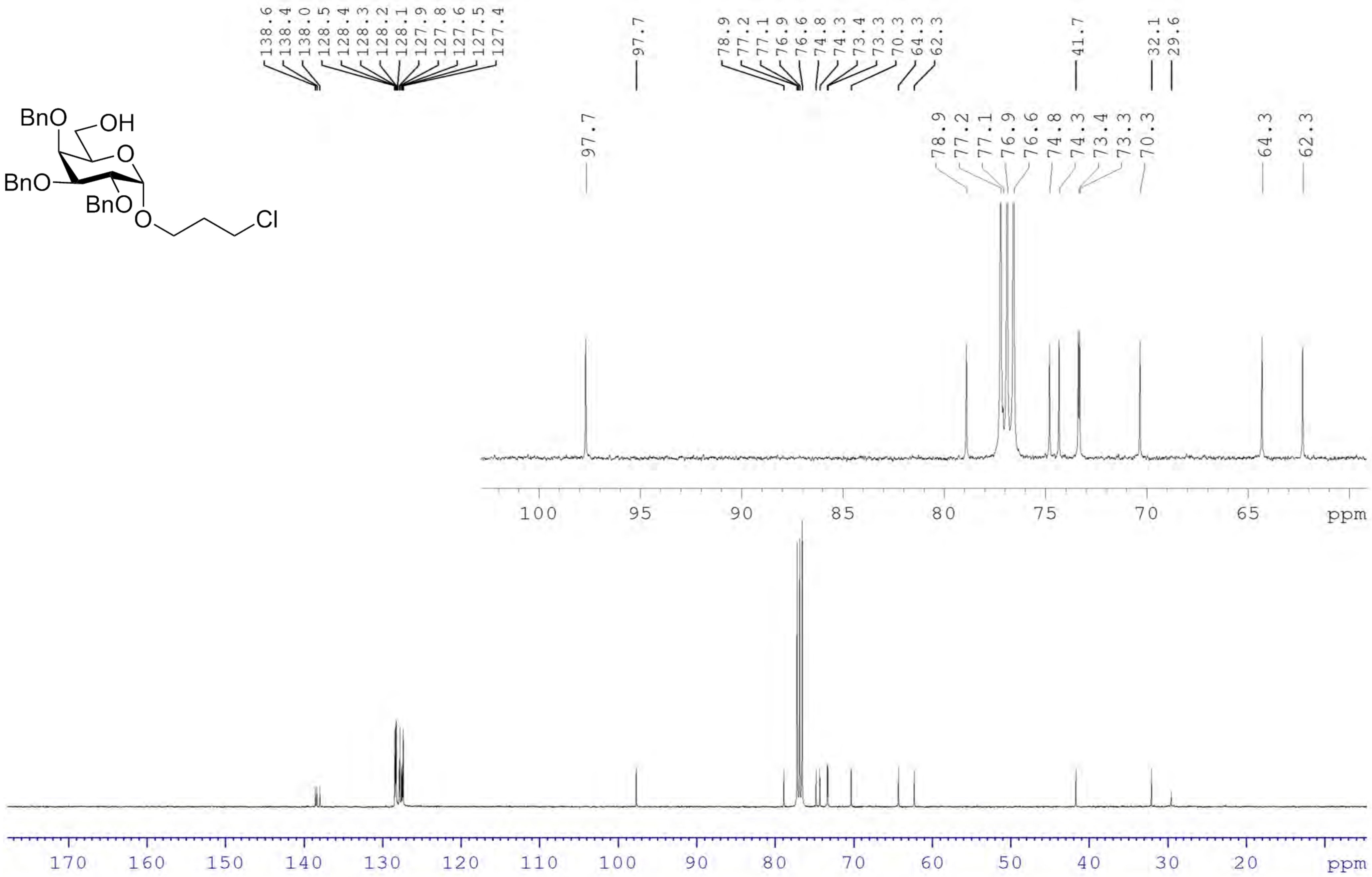

^{1}H-NMR of **21** (600 MHz, $CDCl_3$)

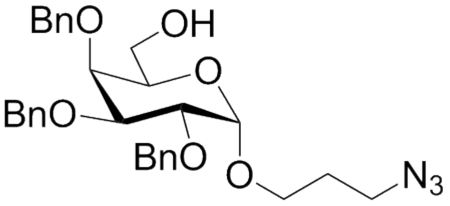

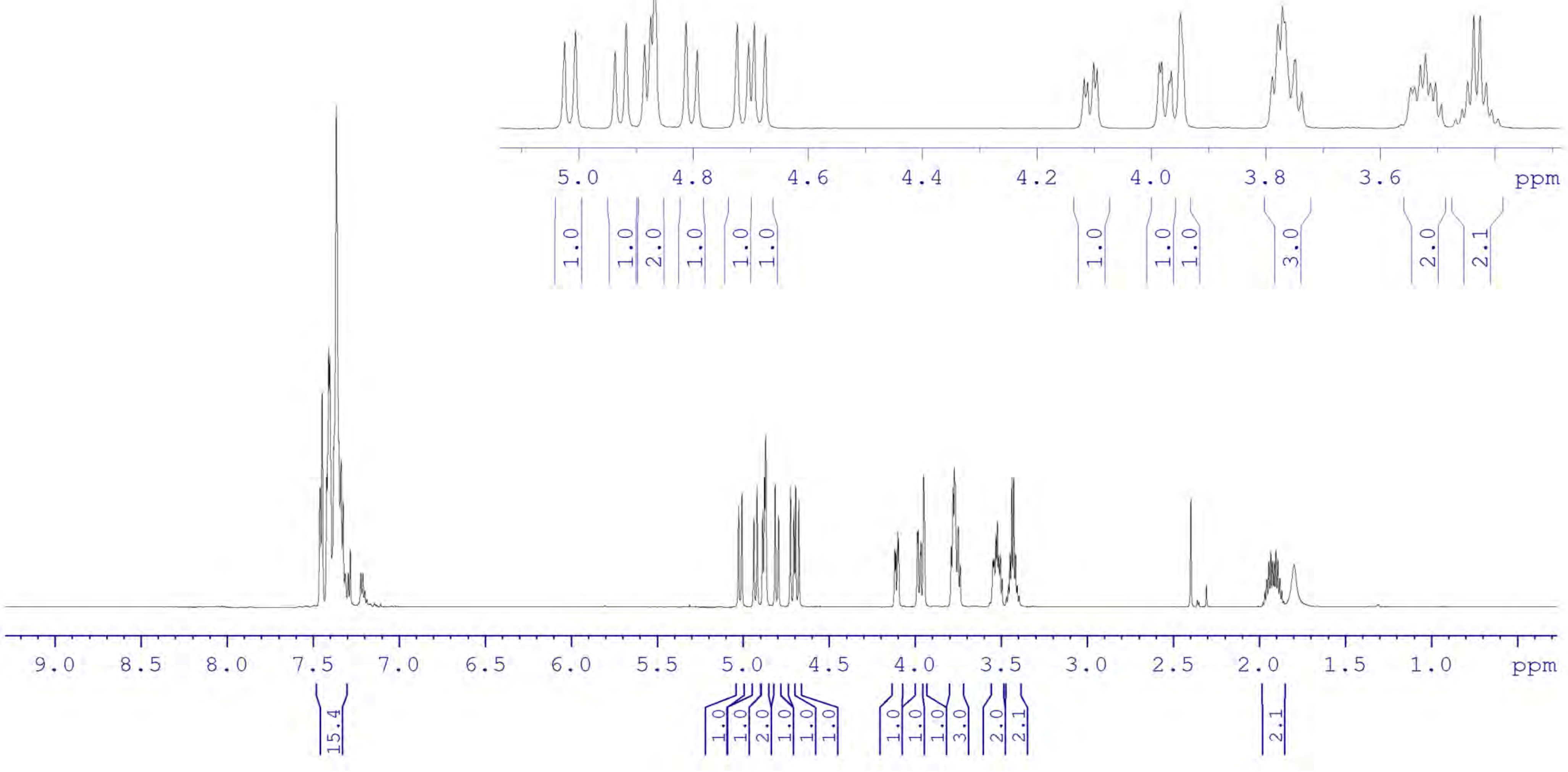

^{13}C-NMR of **21** (150 MHz, $CDCl_3$)

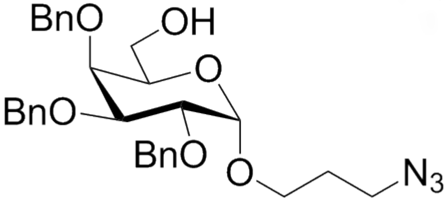

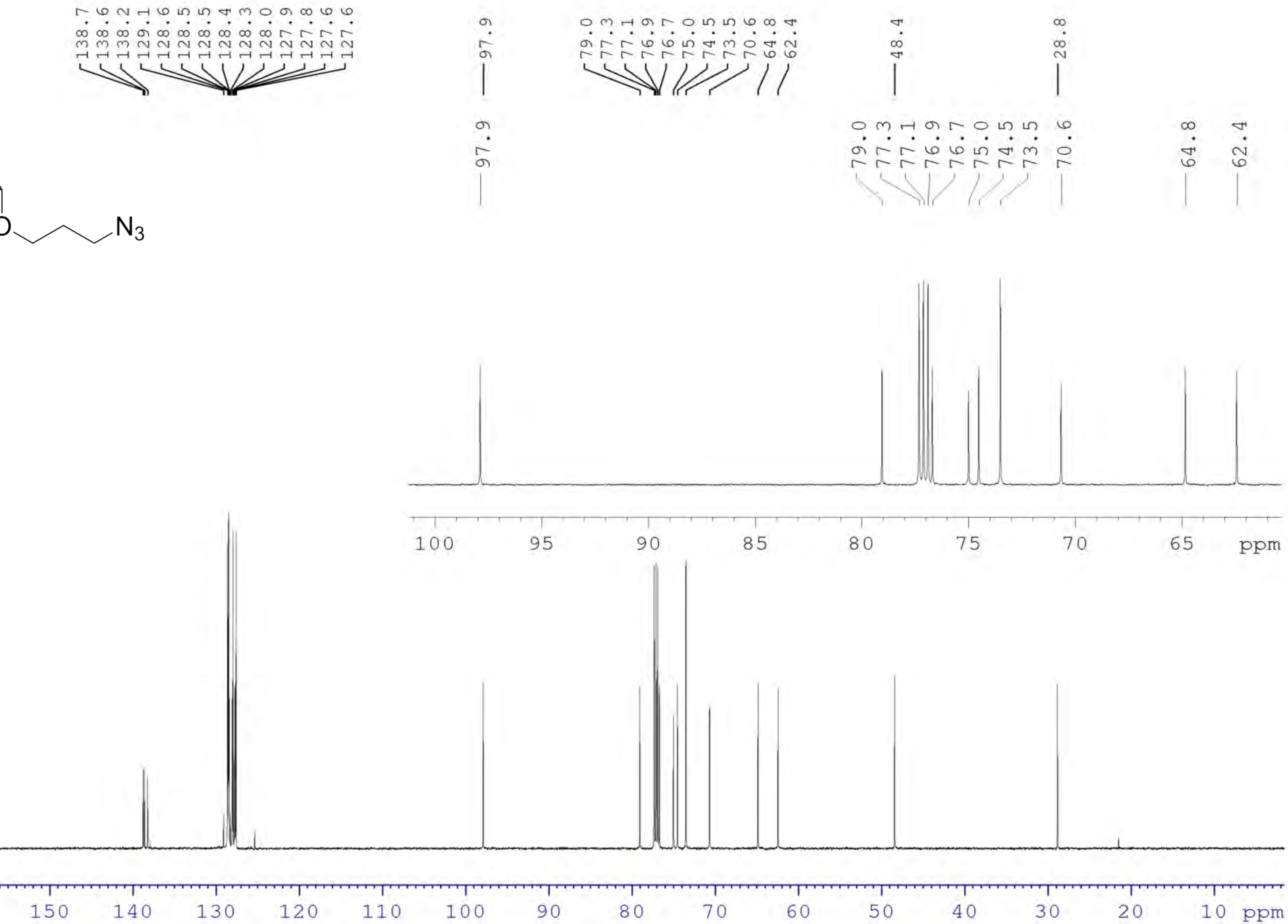

^{1}H-NMR of **23** (400 MHz, $CDCl_3$)

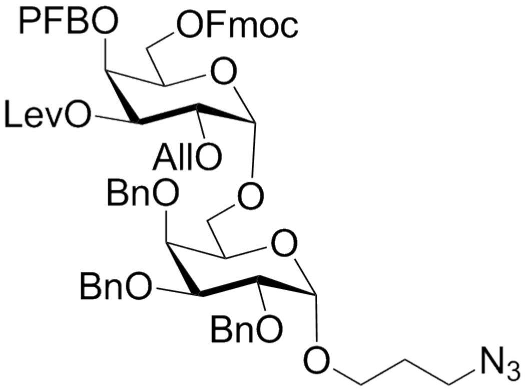

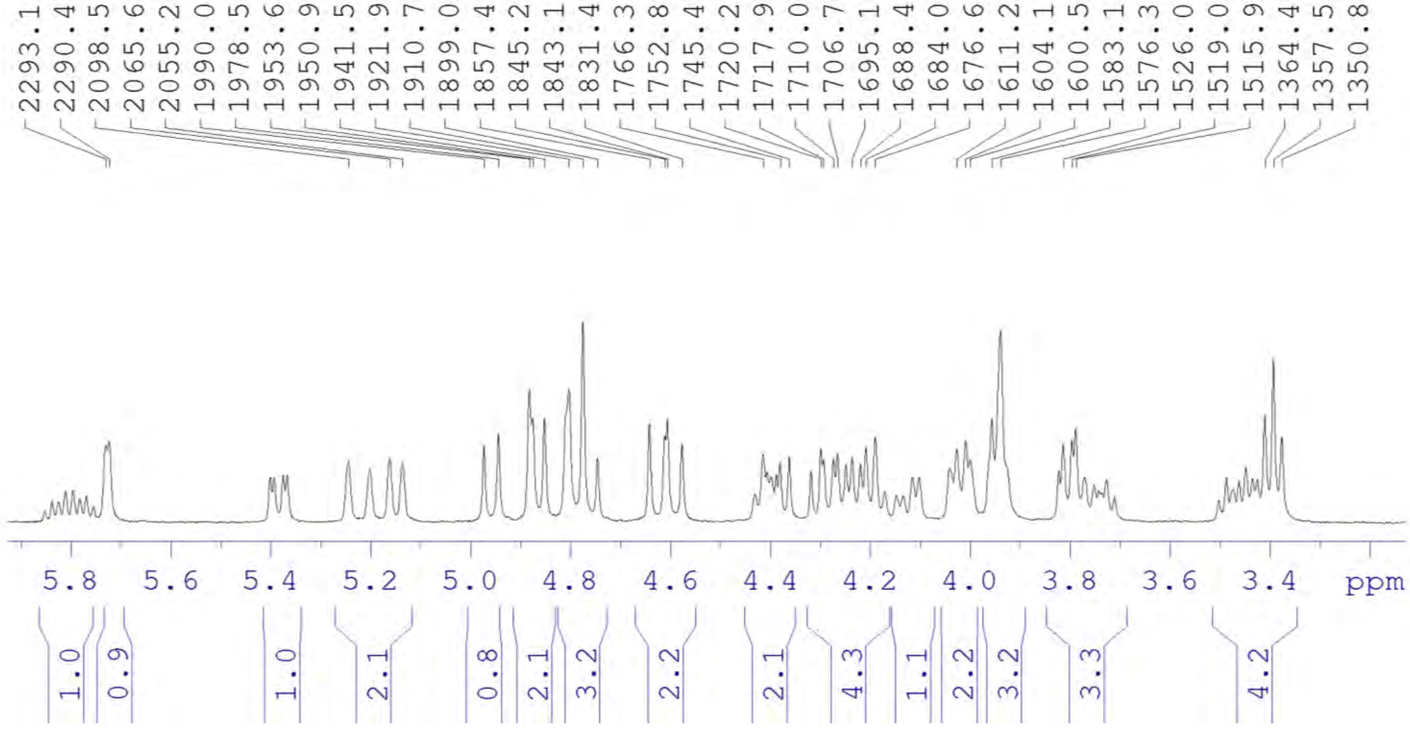

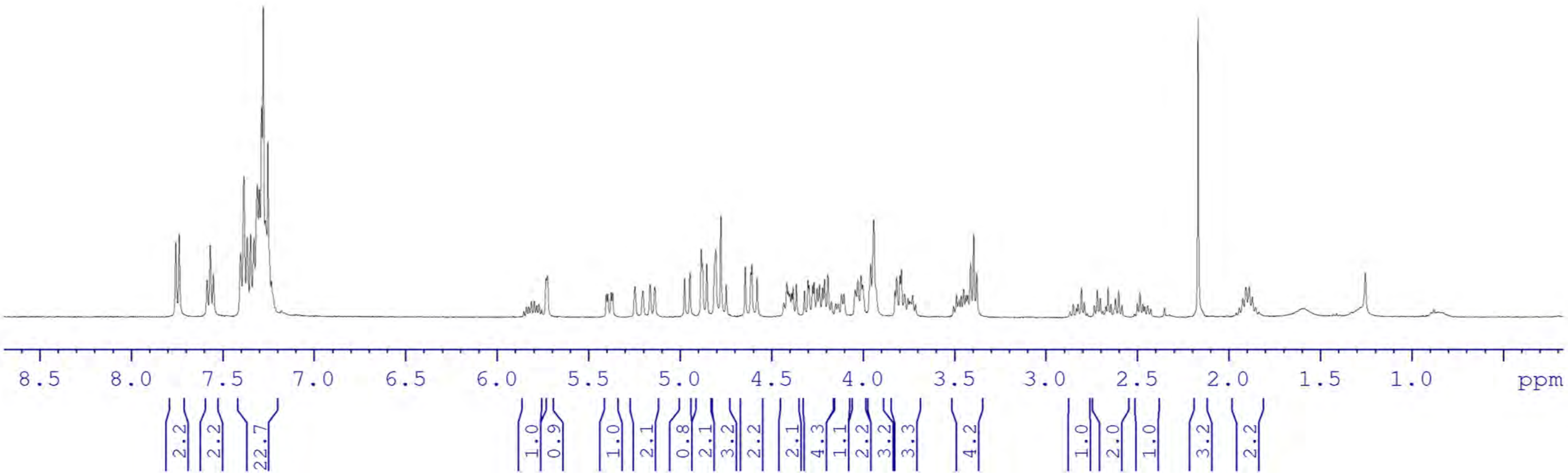

^{13}C-NMR of **23** (100 MHz, $CDCl_3$)

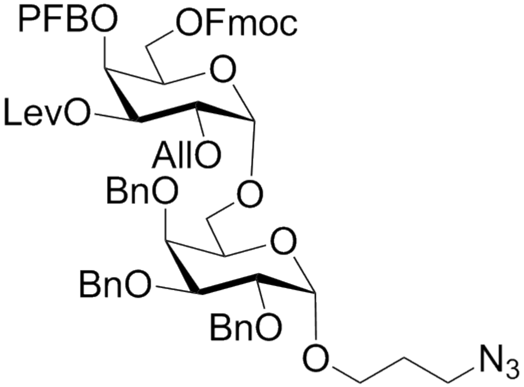

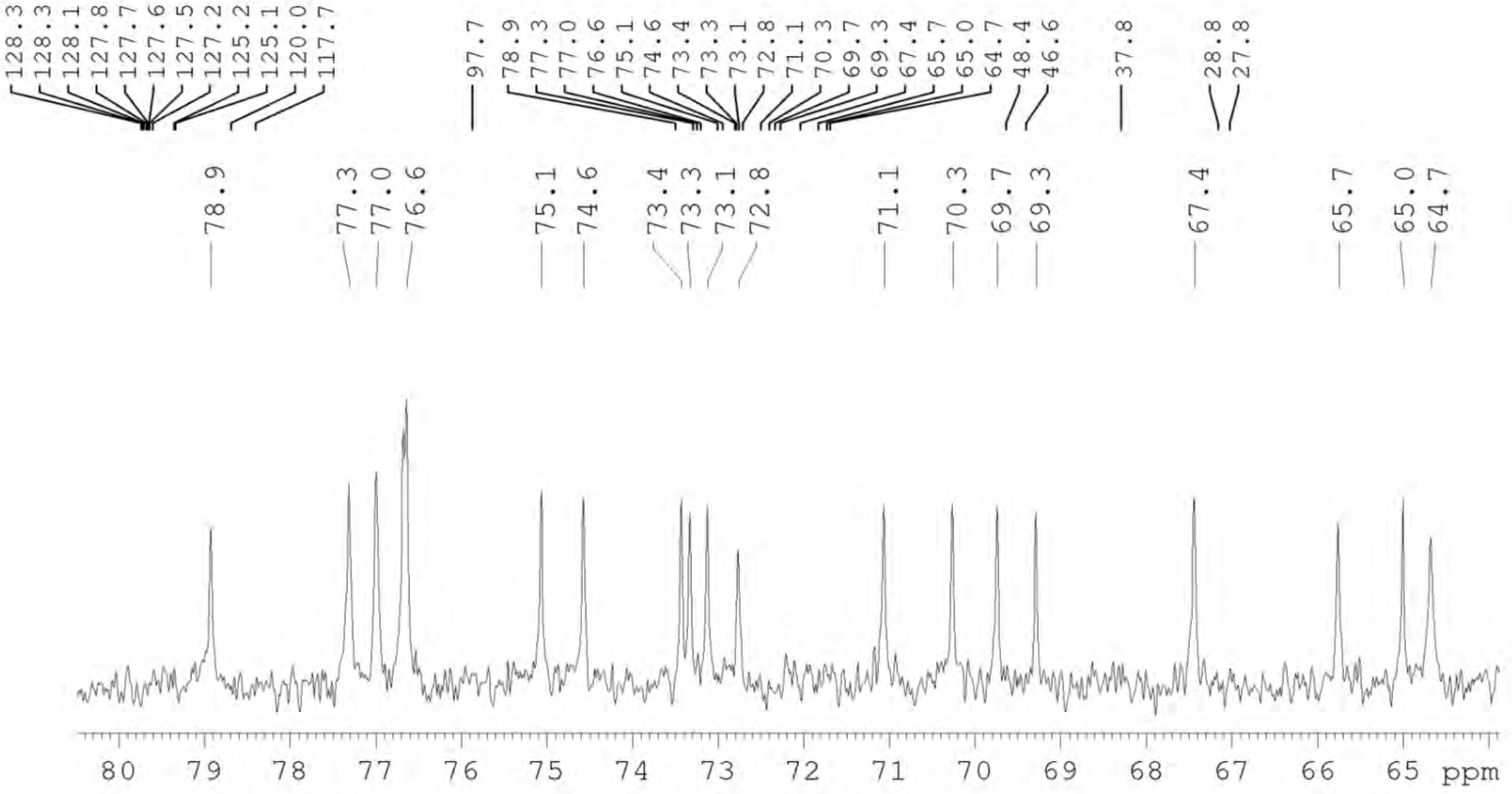

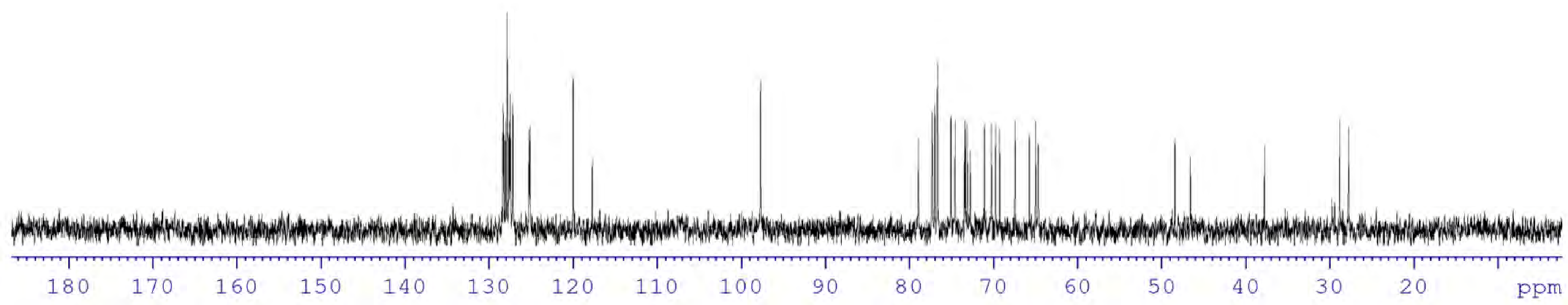

^{1}H-^{1}H COSY of **23** (400 MHz, $CDCl_3$)

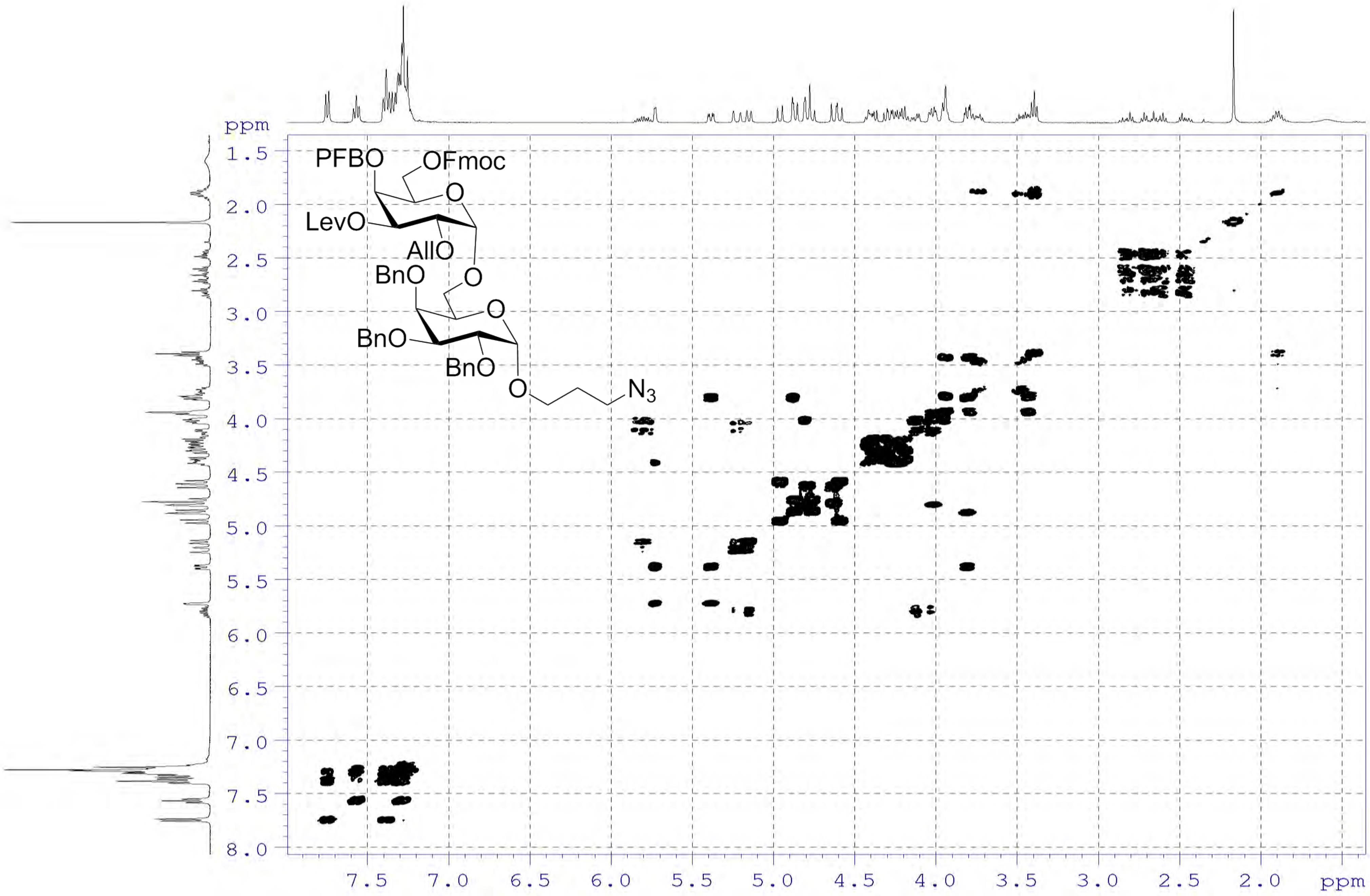

^{1}H-^{13}C HSQC of **23** (400 MHz, $CDCl_3$)

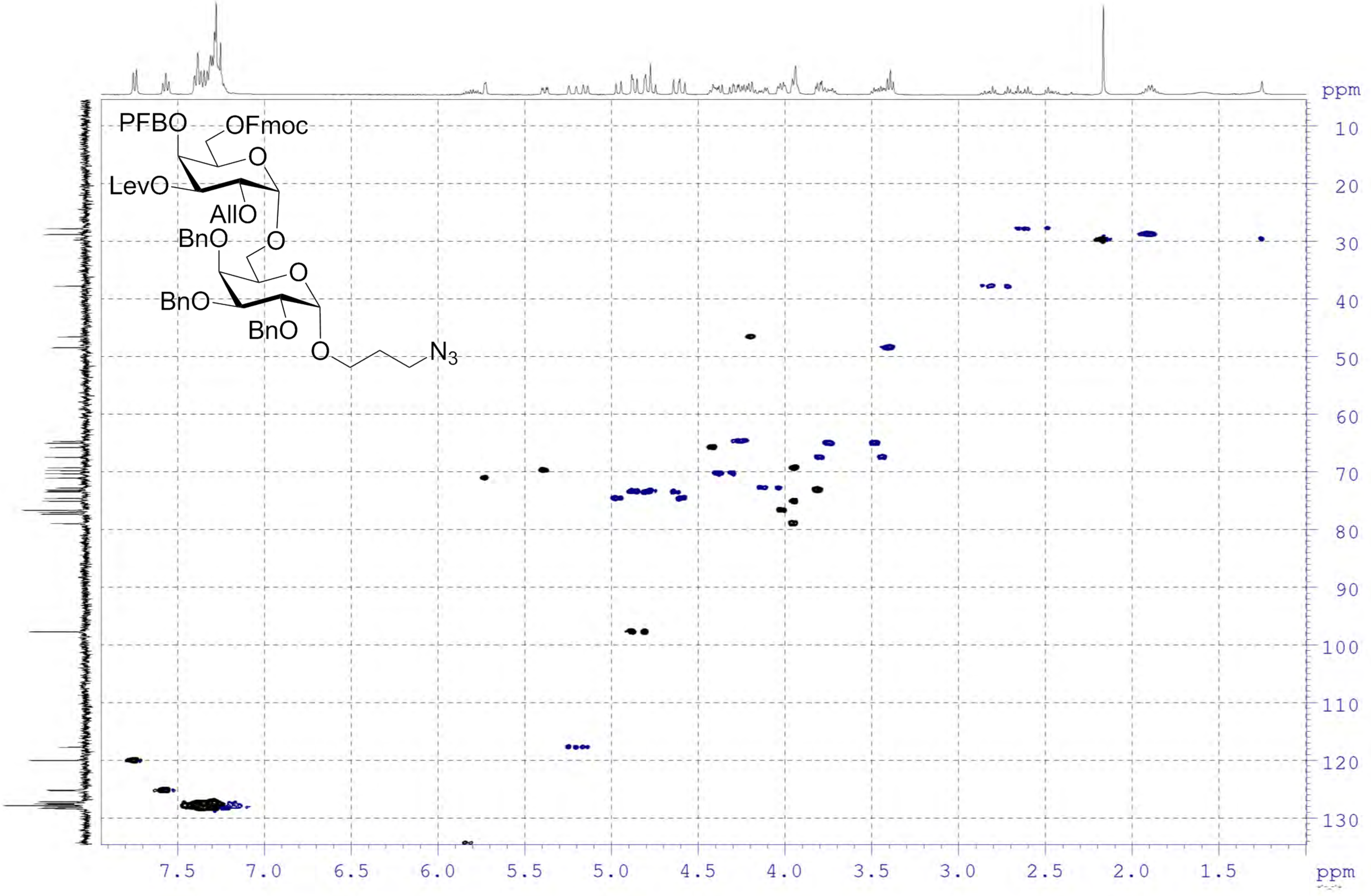

^{1}H-NMR of **24** (300 MHz, $CDCl_3$)

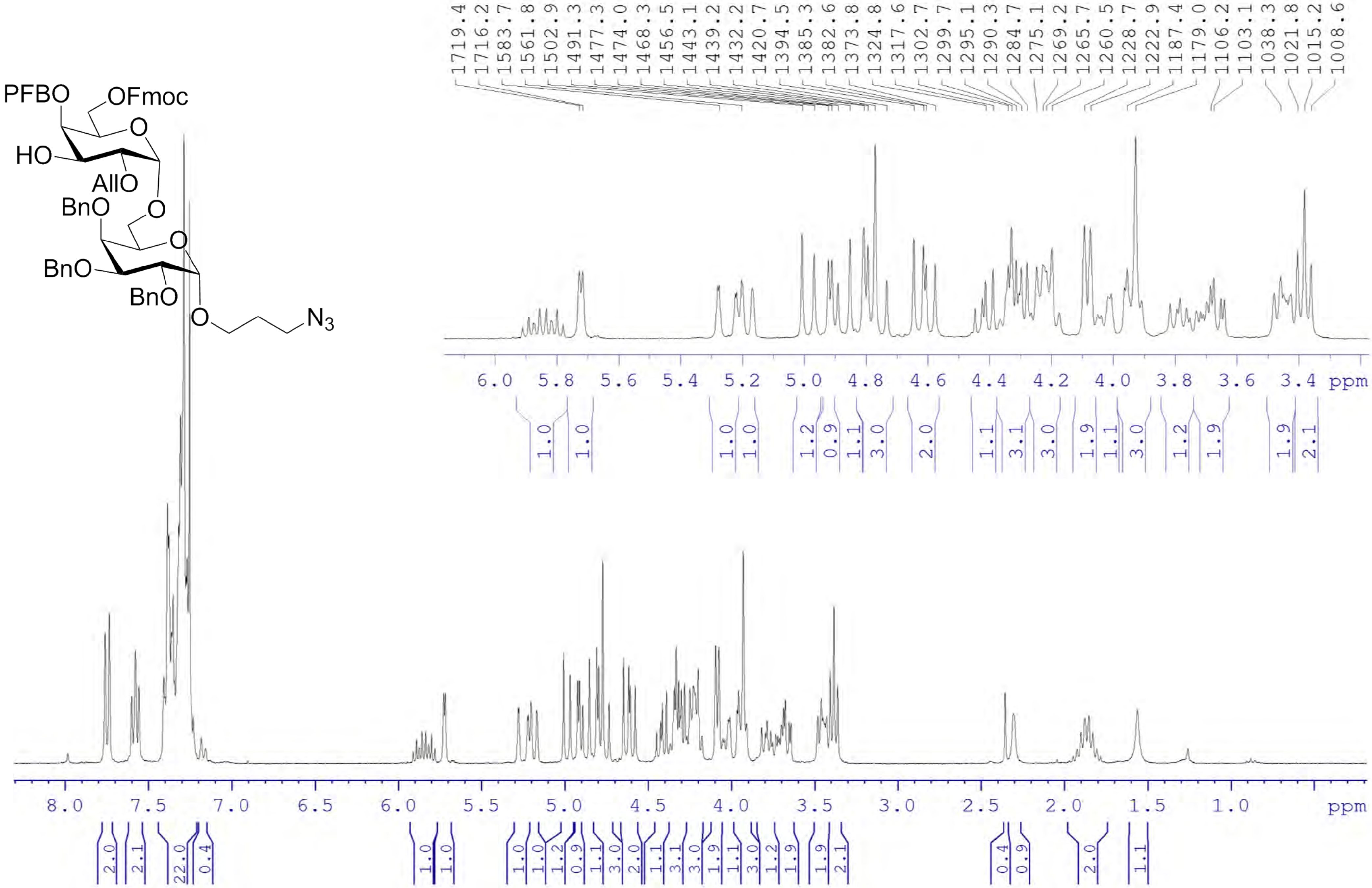

^{13}C-NMR of **24** (75 MHz, $CDCl_3$)

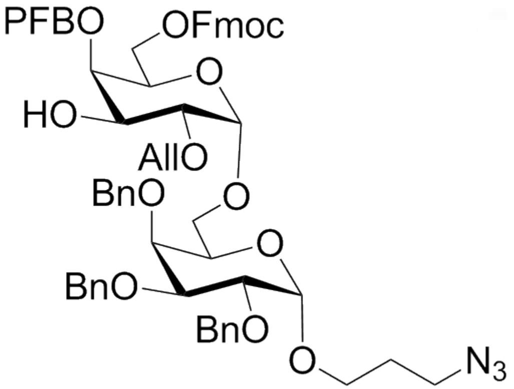

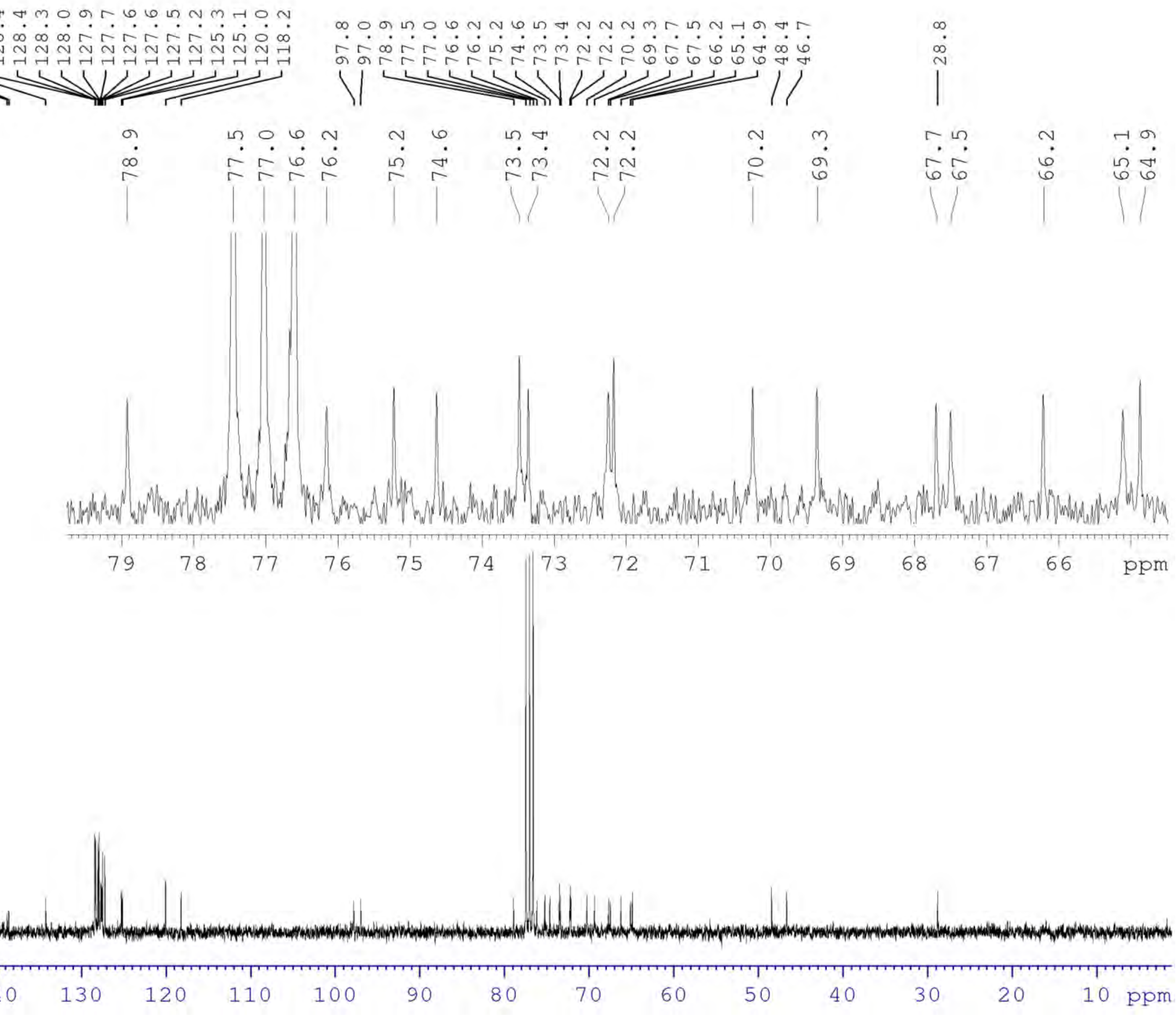

^{1}H-^{1}H COSY of **24** (300 MHz, $CDCl_3$)

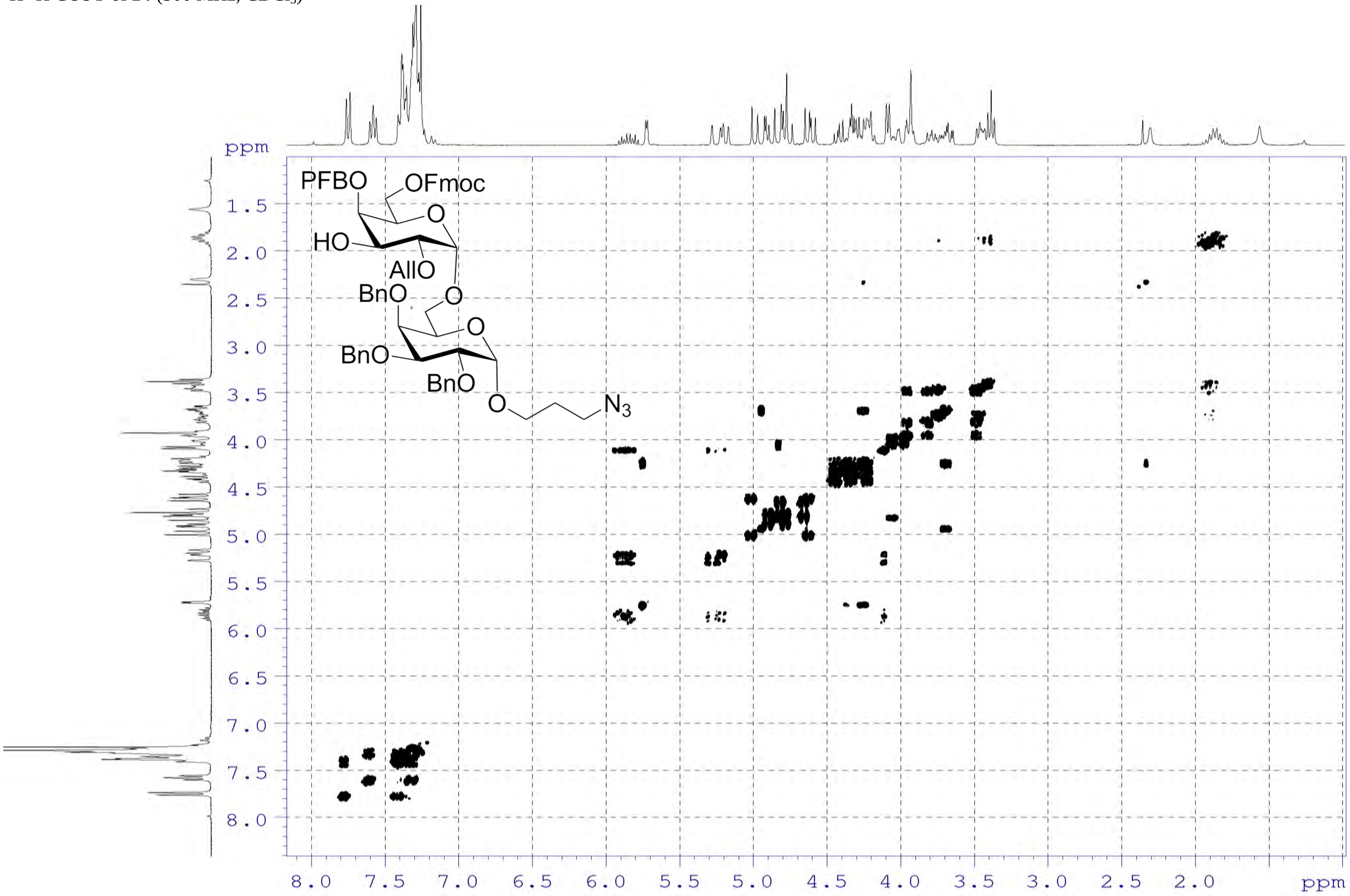

^{1}H-^{13}C HSQC of **24** (300 MHz, $CDCl_3$)

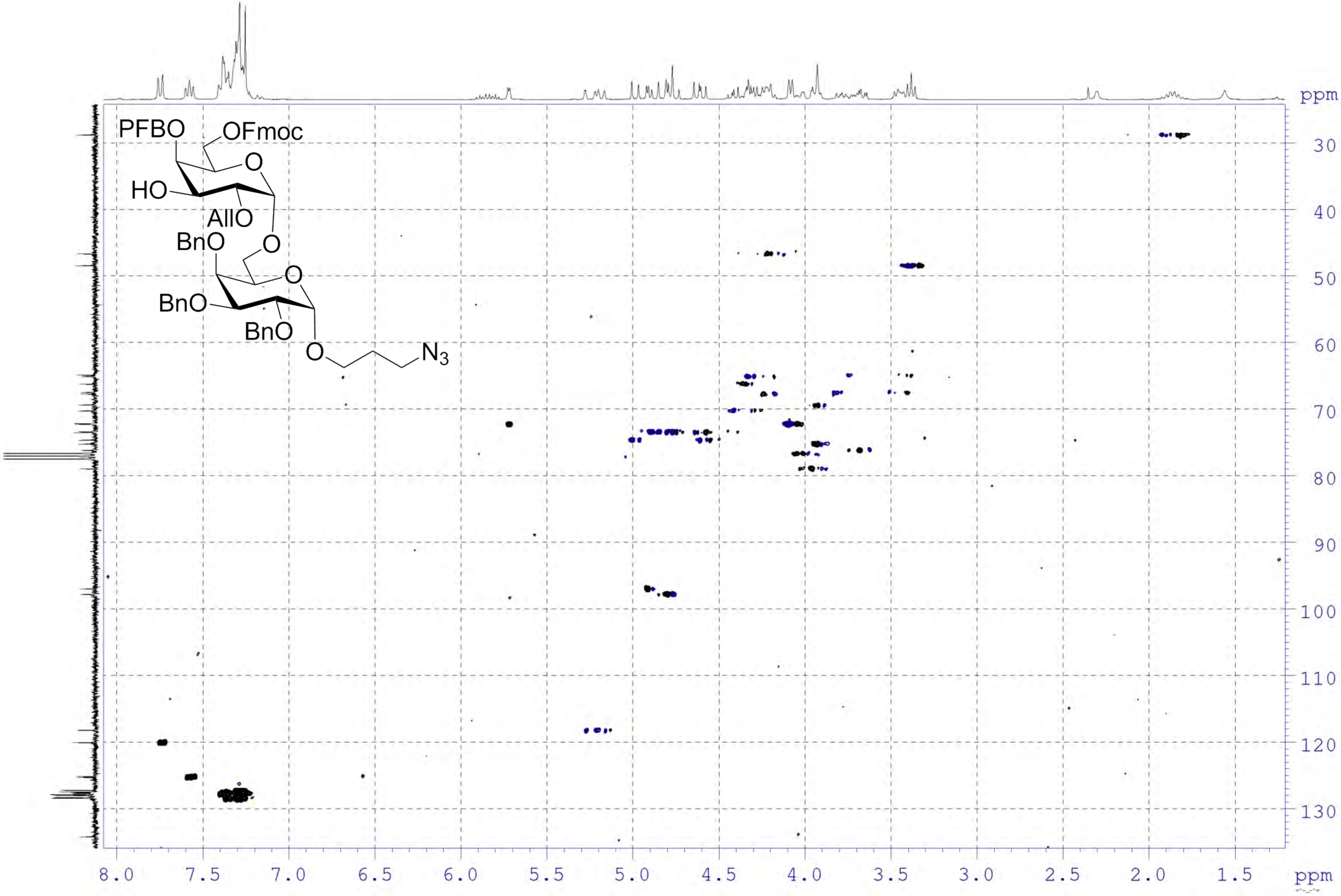

^{1}H-NMR of **25** (300 MHz, $CDCl_3$)

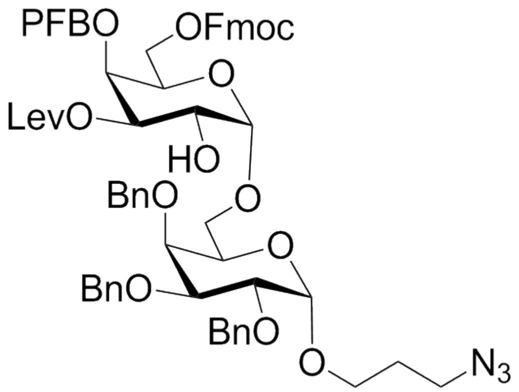

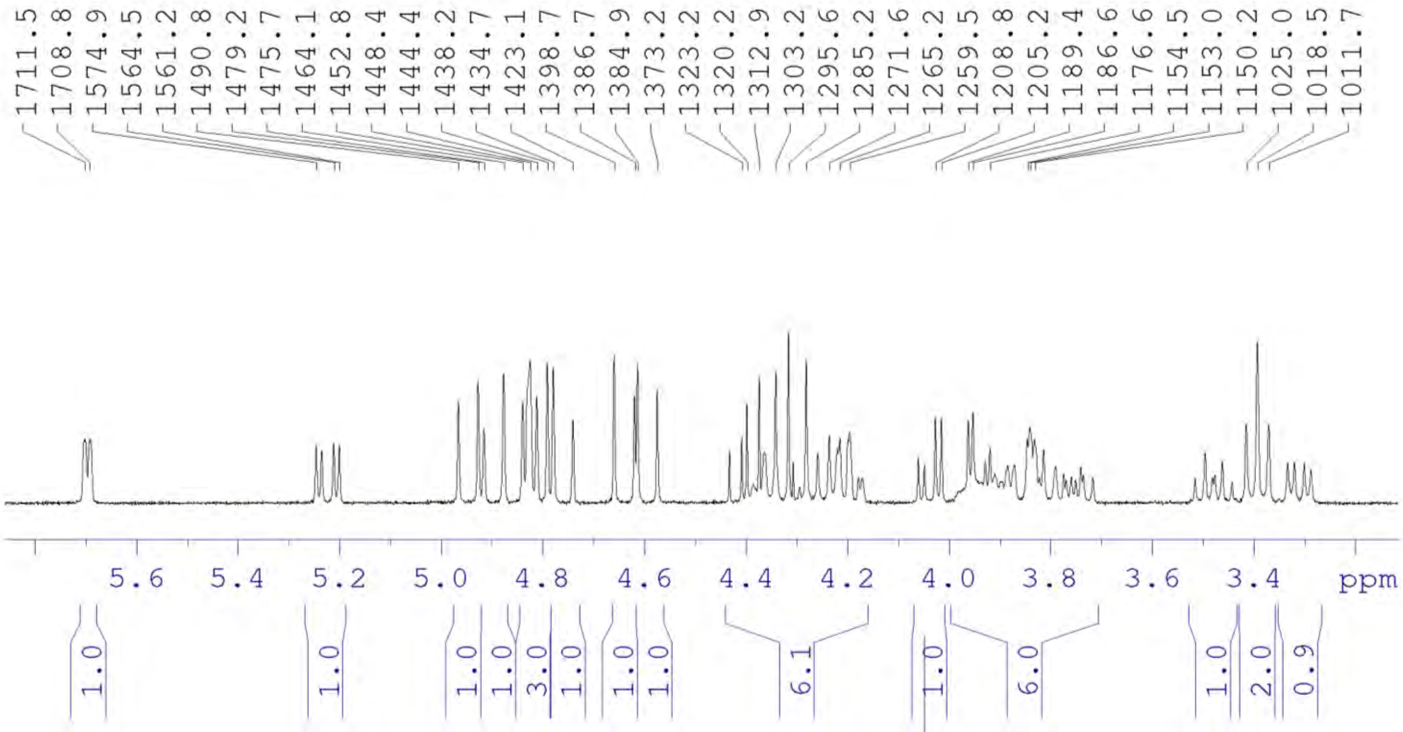

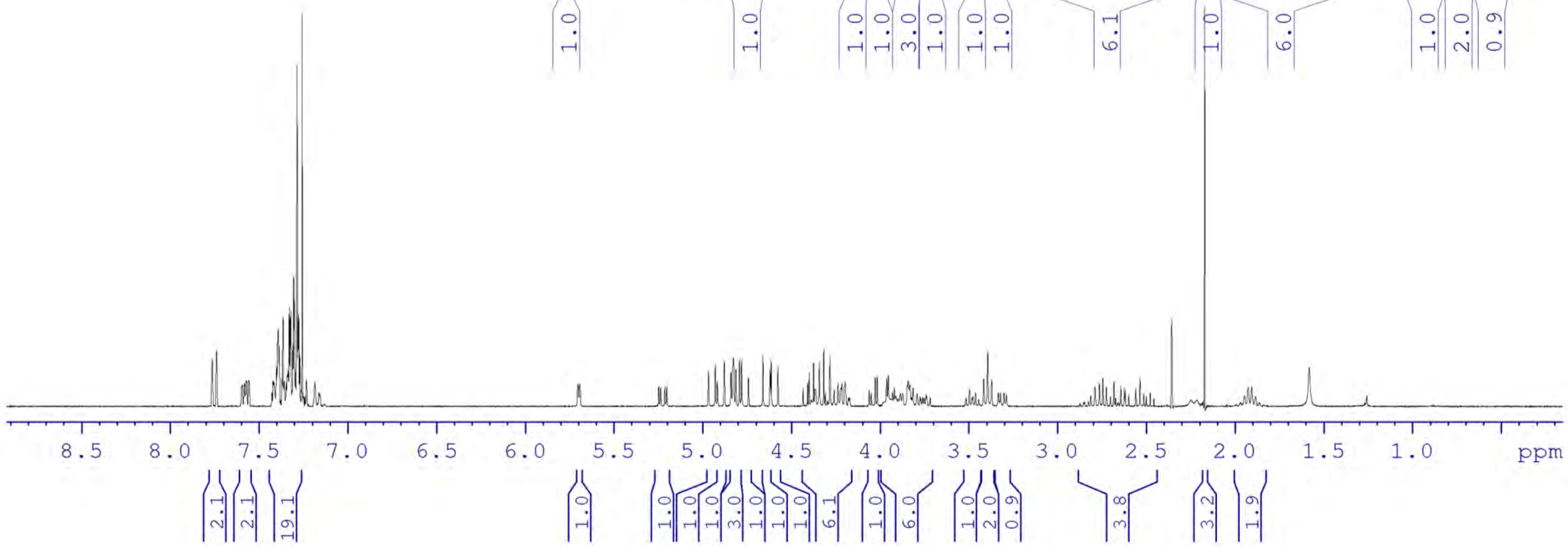

^{13}C-NMR of **25** (75 MHz, $CDCl_3$)

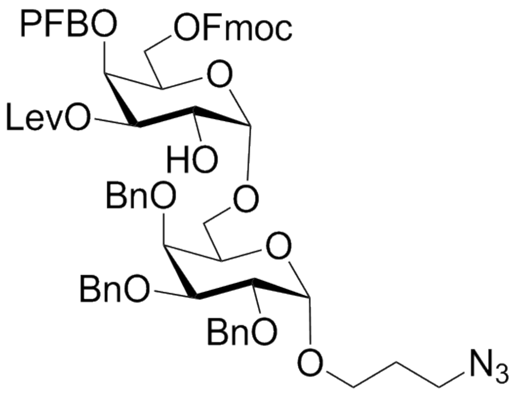

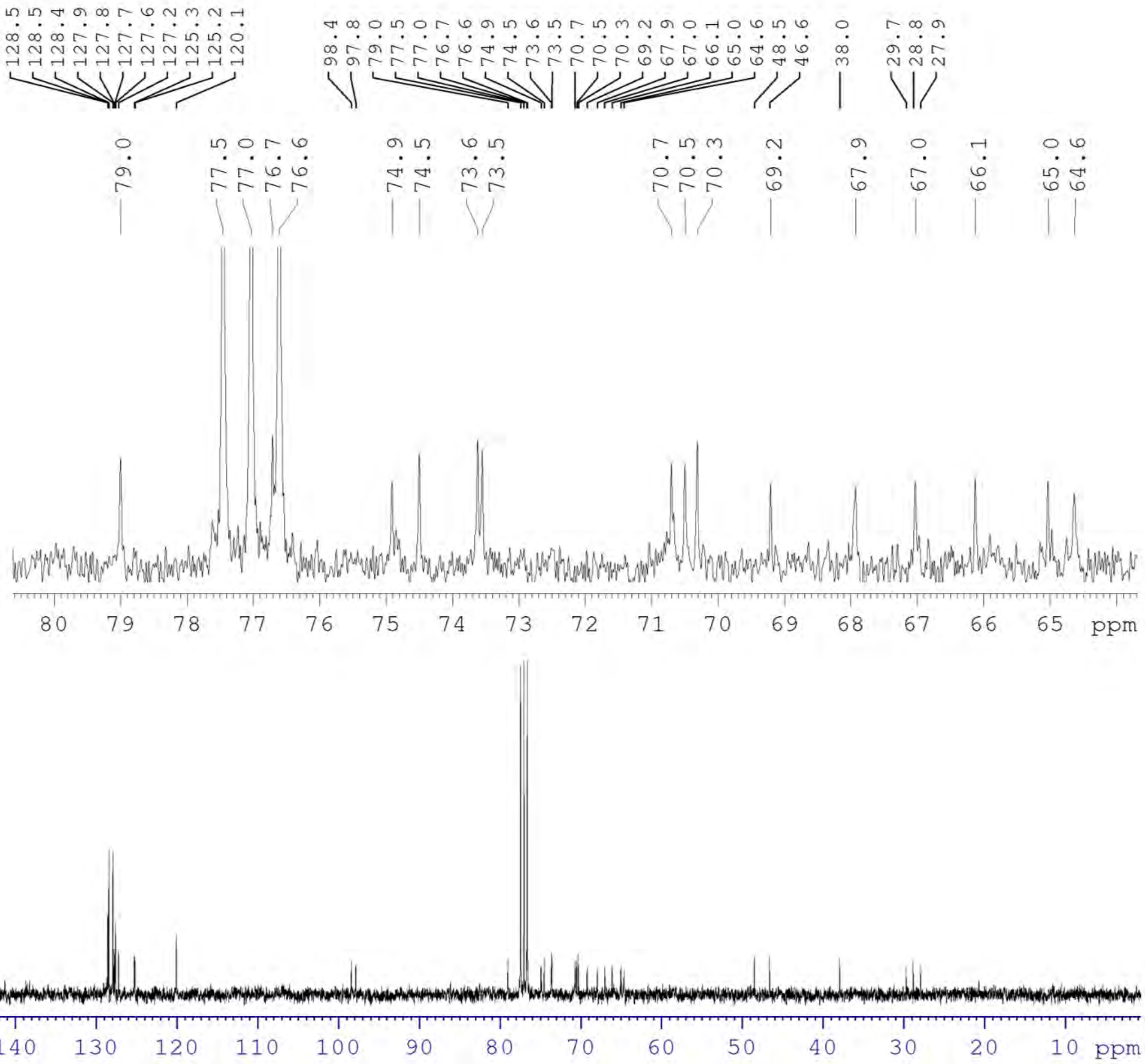

^{1}H-^{1}H COSY of **25** (300 MHz, $CDCl_3$)

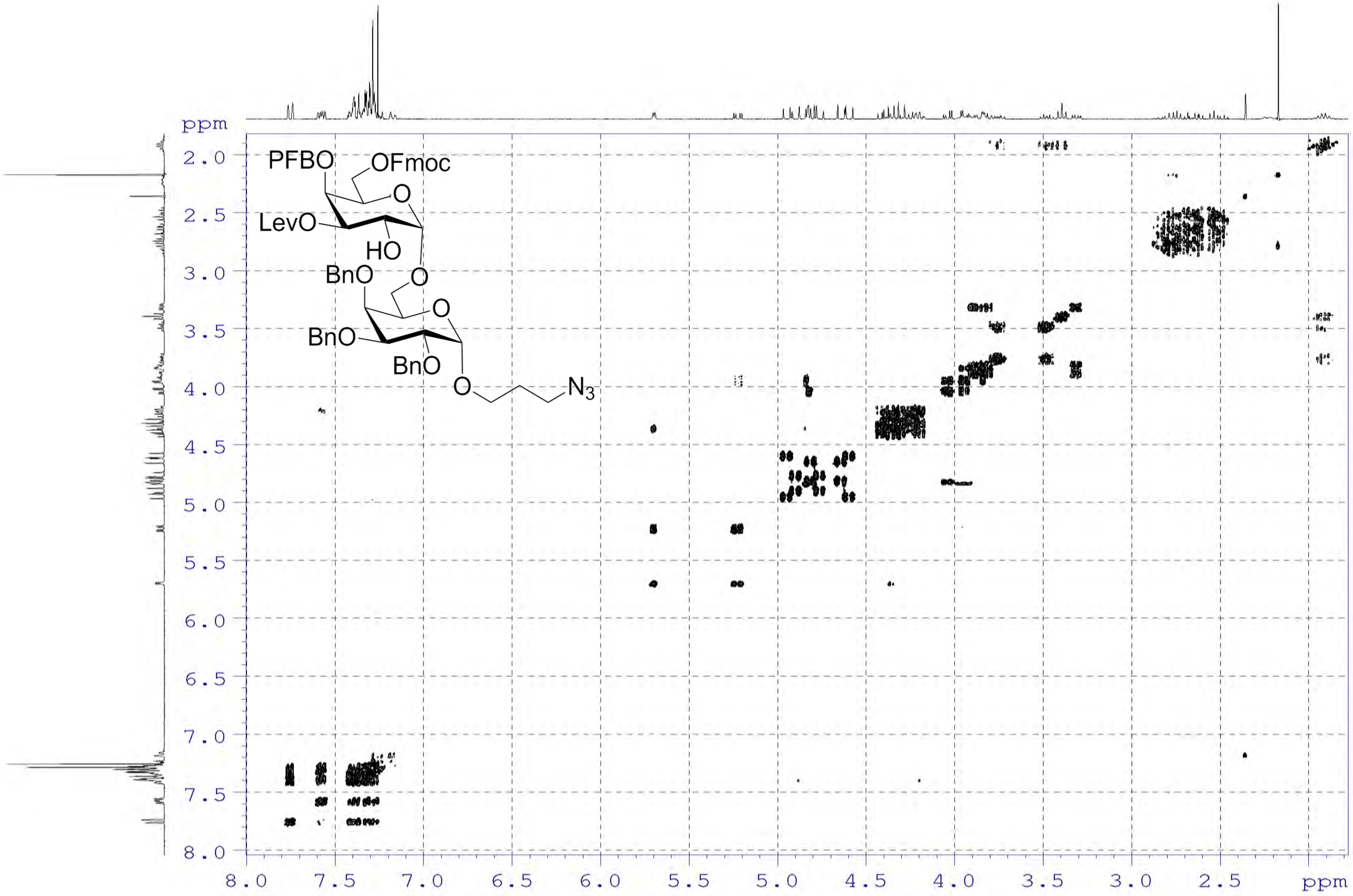

^{1}H-^{13}C HSQC of **25** (300 MHz, $CDCl_3$)

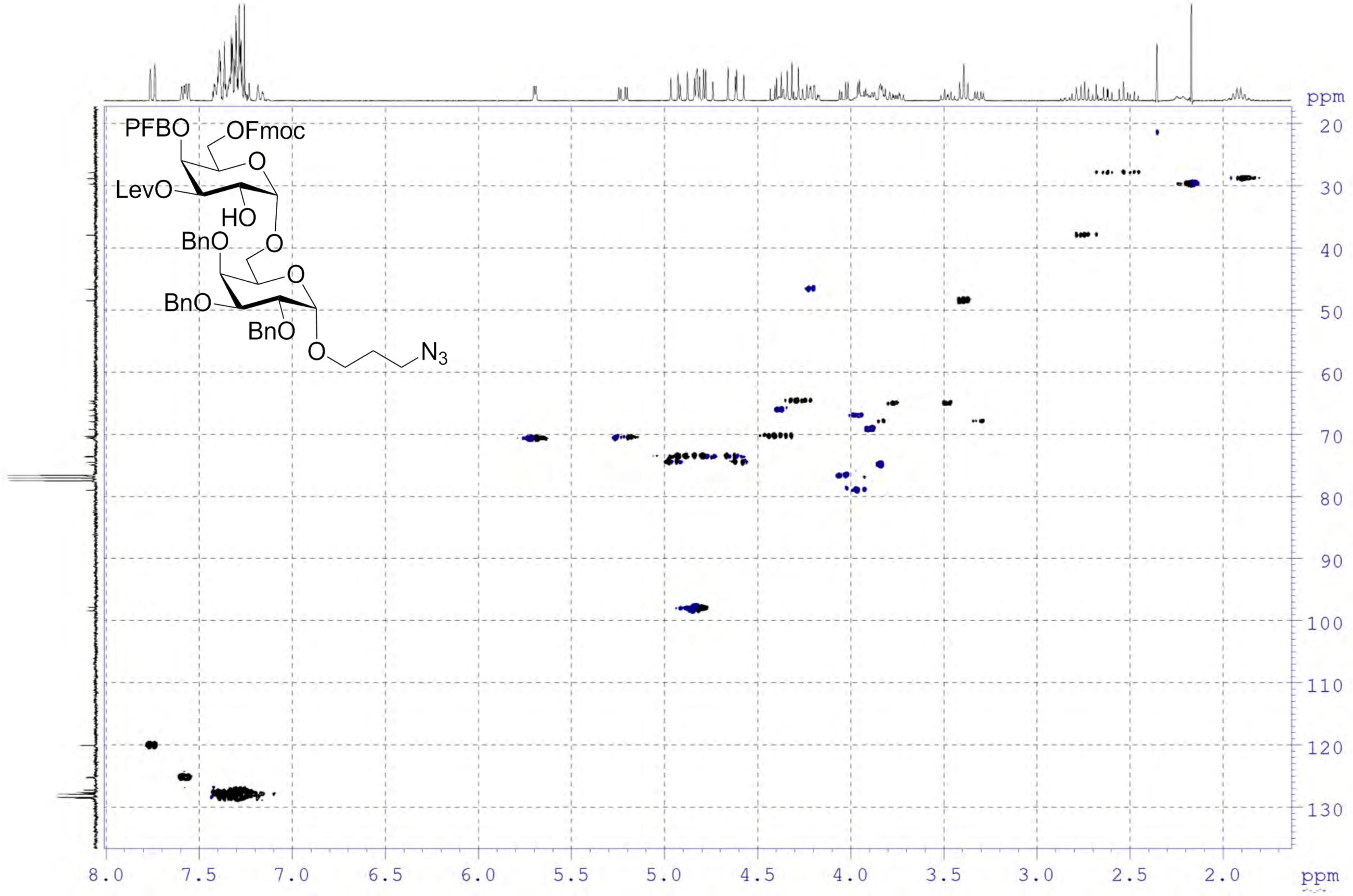

^{1}H-NMR of **26** (600 MHz, $CDCl_3$)

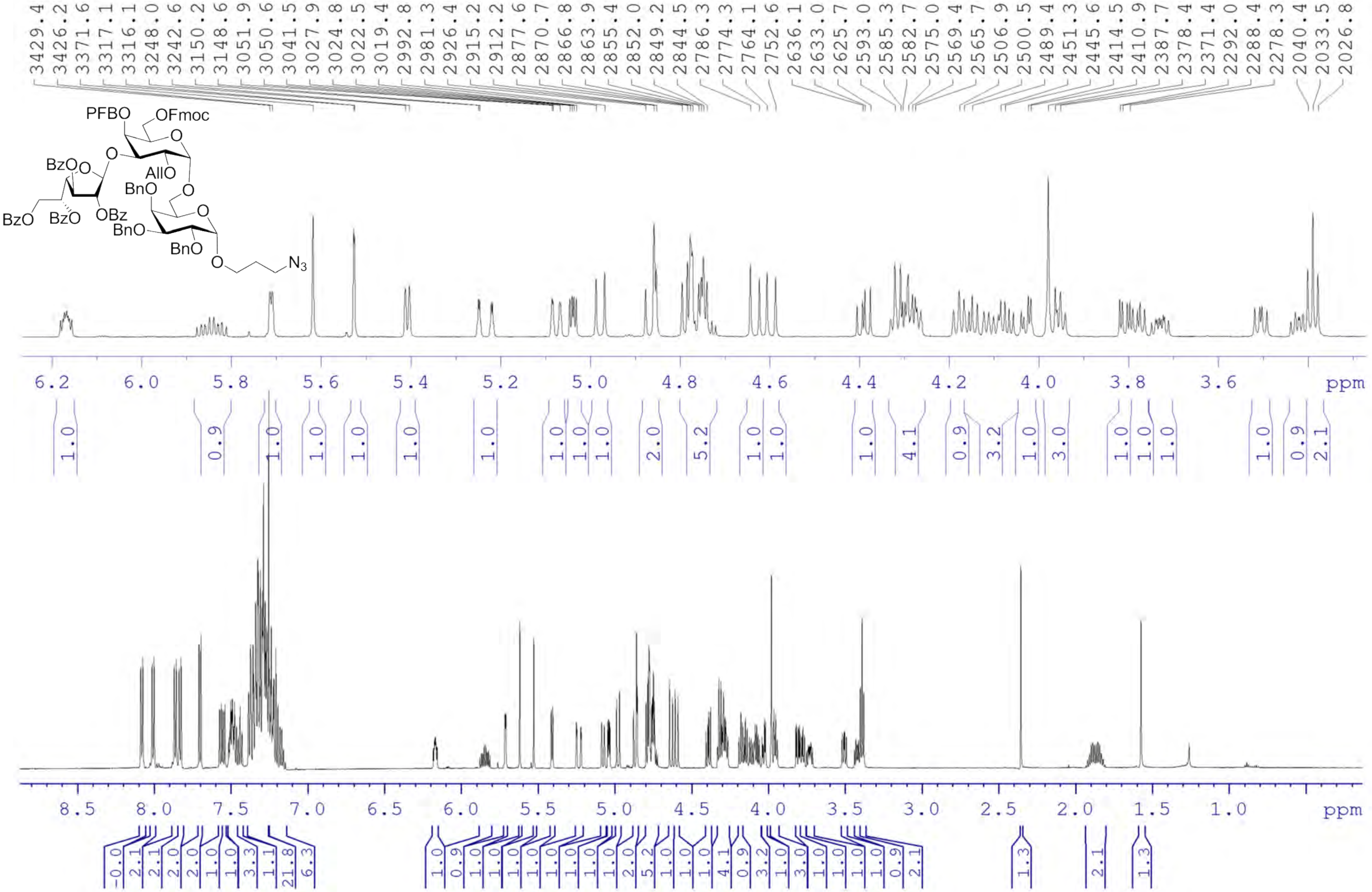

^{13}C-NMR of **26** (150 MHz, $CDCl_3$)

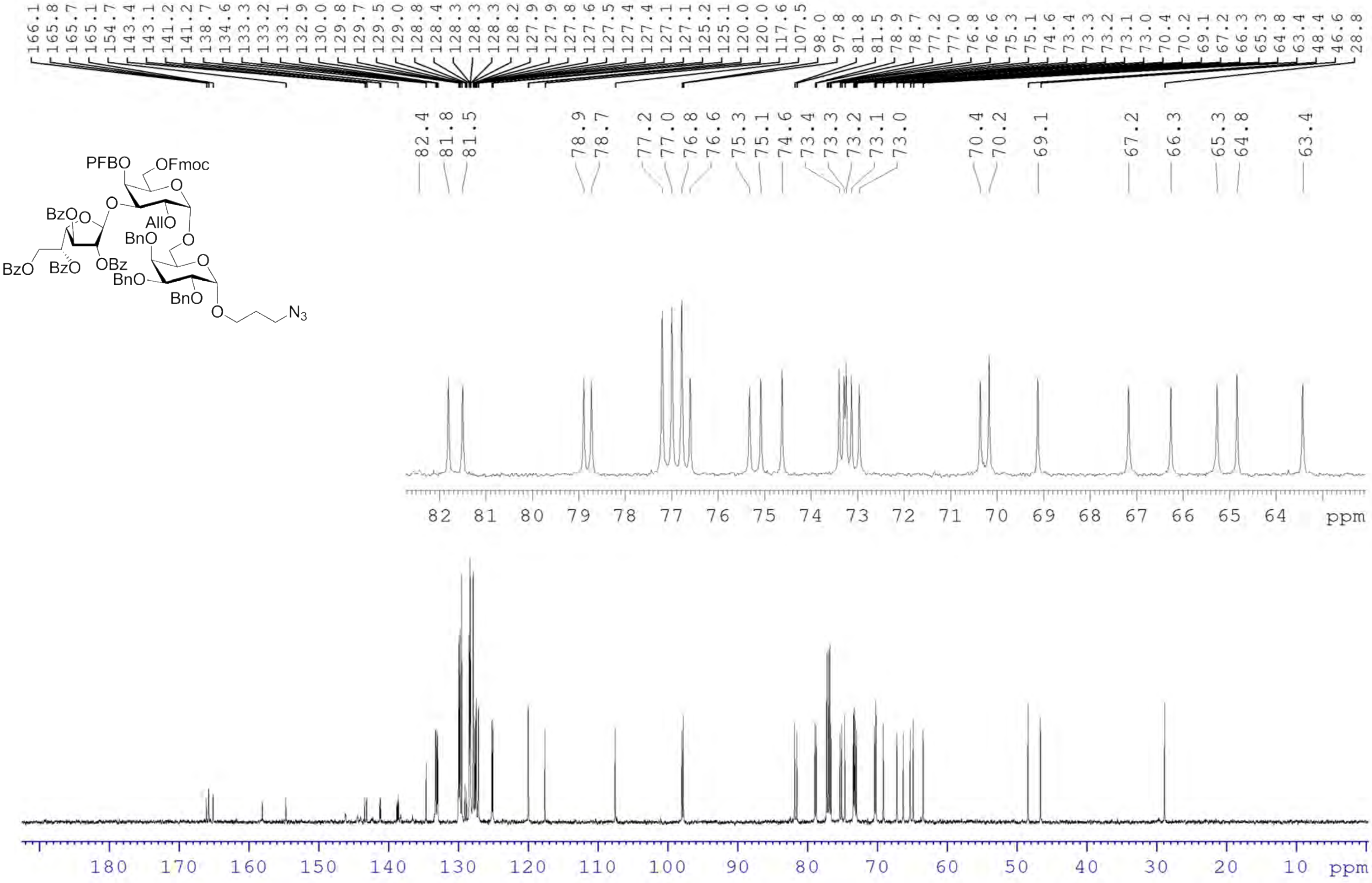

^{1}H-^{1}H COSY of **26** (600 MHz, $CDCl_3$)

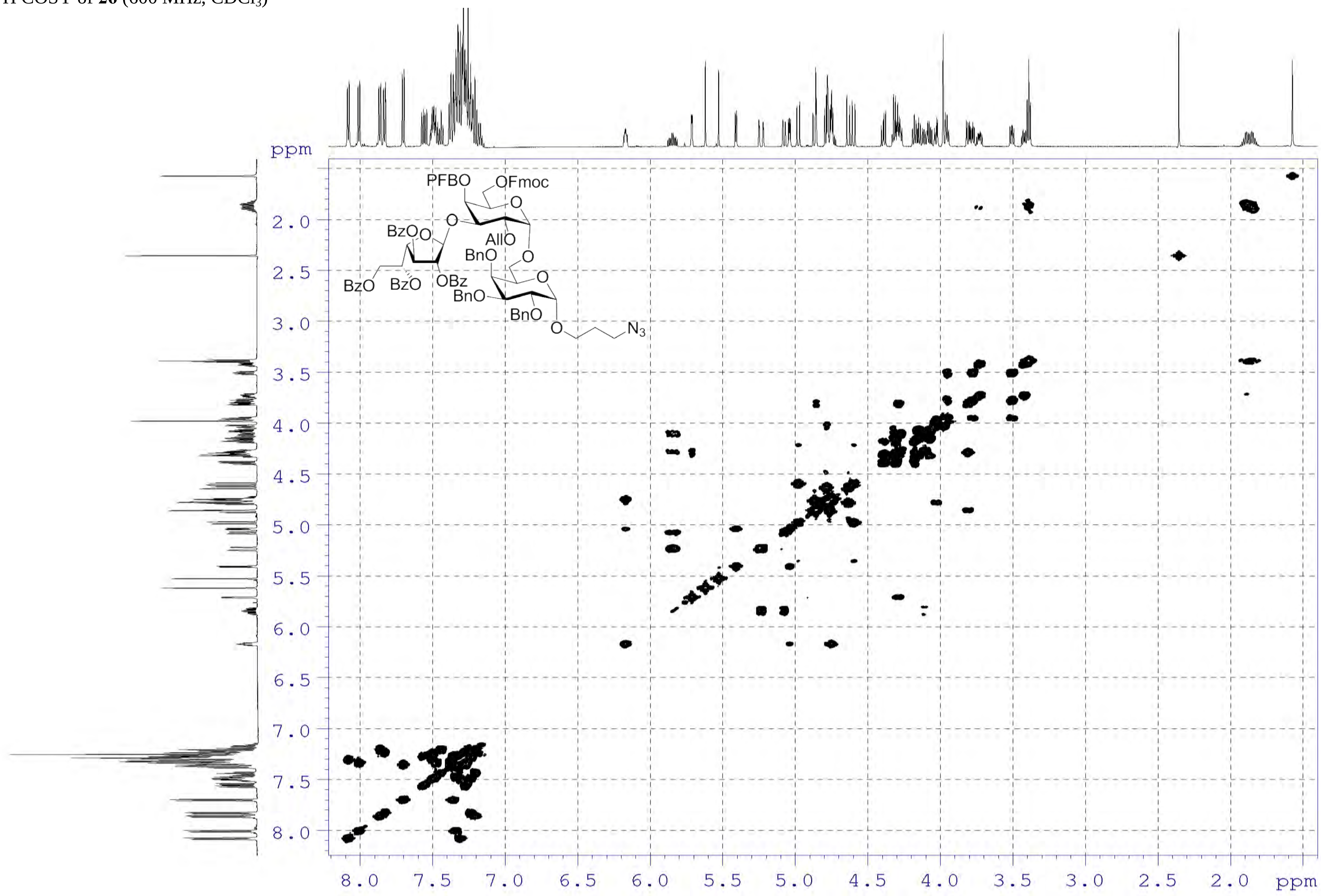

^{1}H-^{13}C HSQC of **26** (600 MHz, $CDCl_3$)

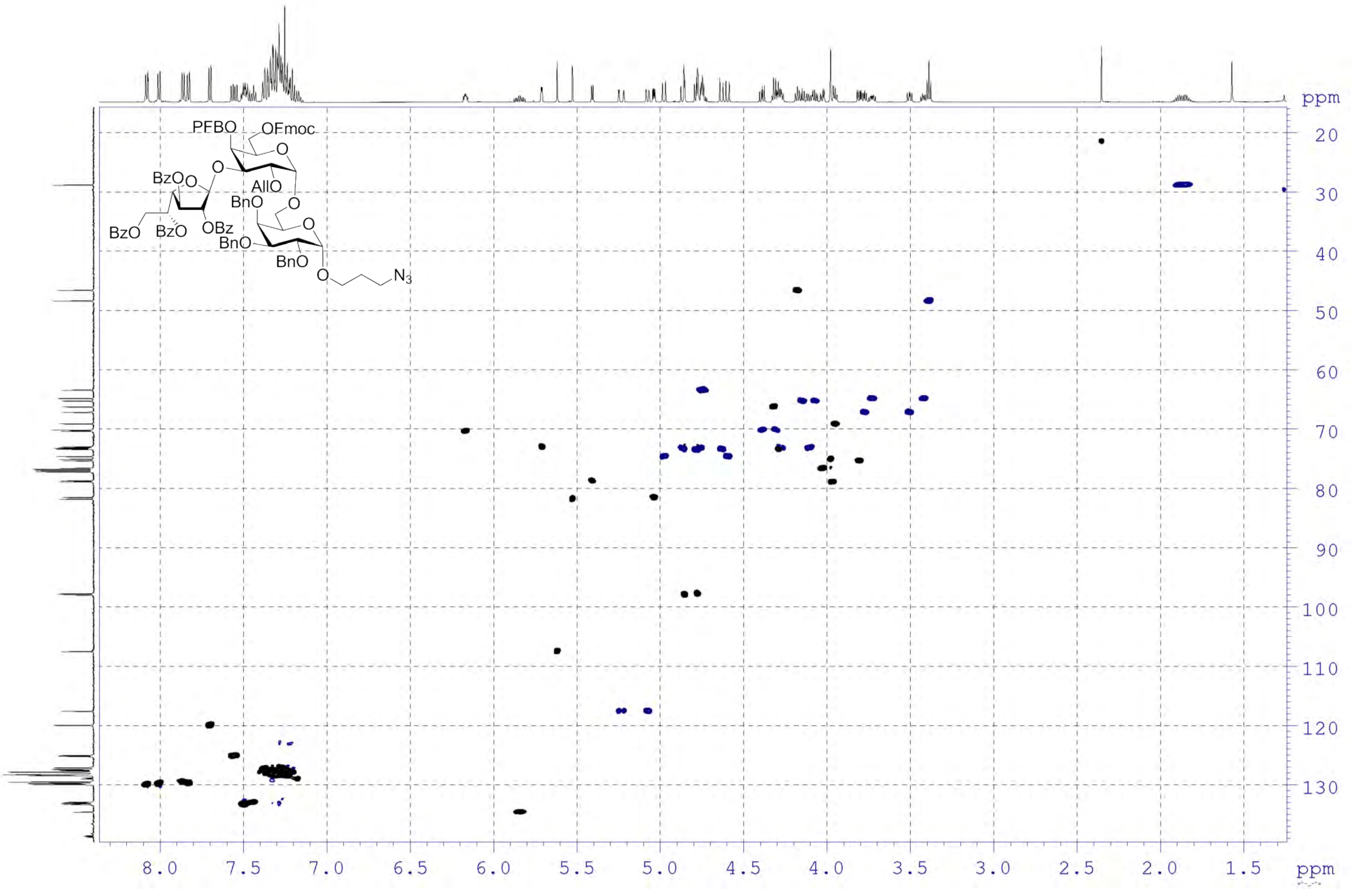

^{1}H-NMR of **27** (300 MHz, $CDCl_3$)

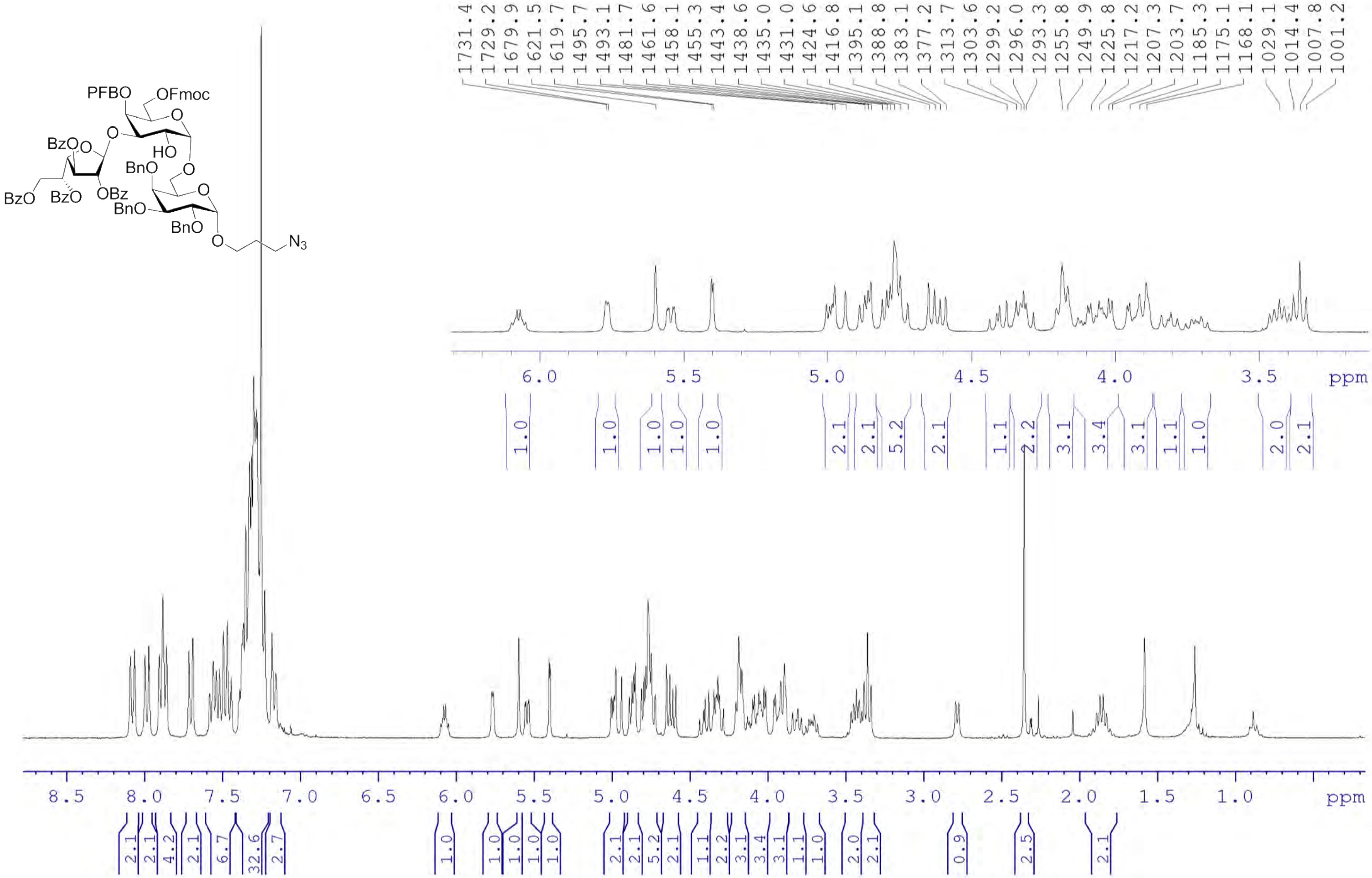

^{13}C-NMR of **27** (75 MHz, $CDCl_3$)

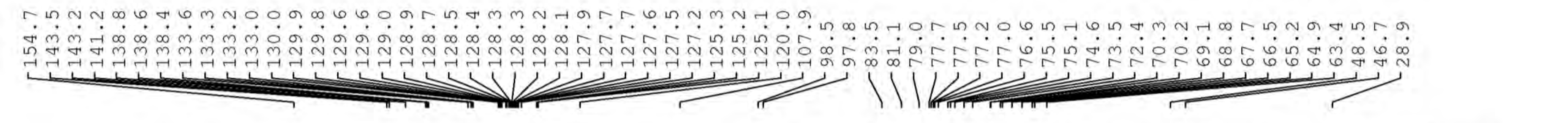

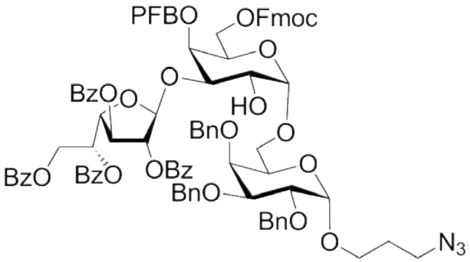

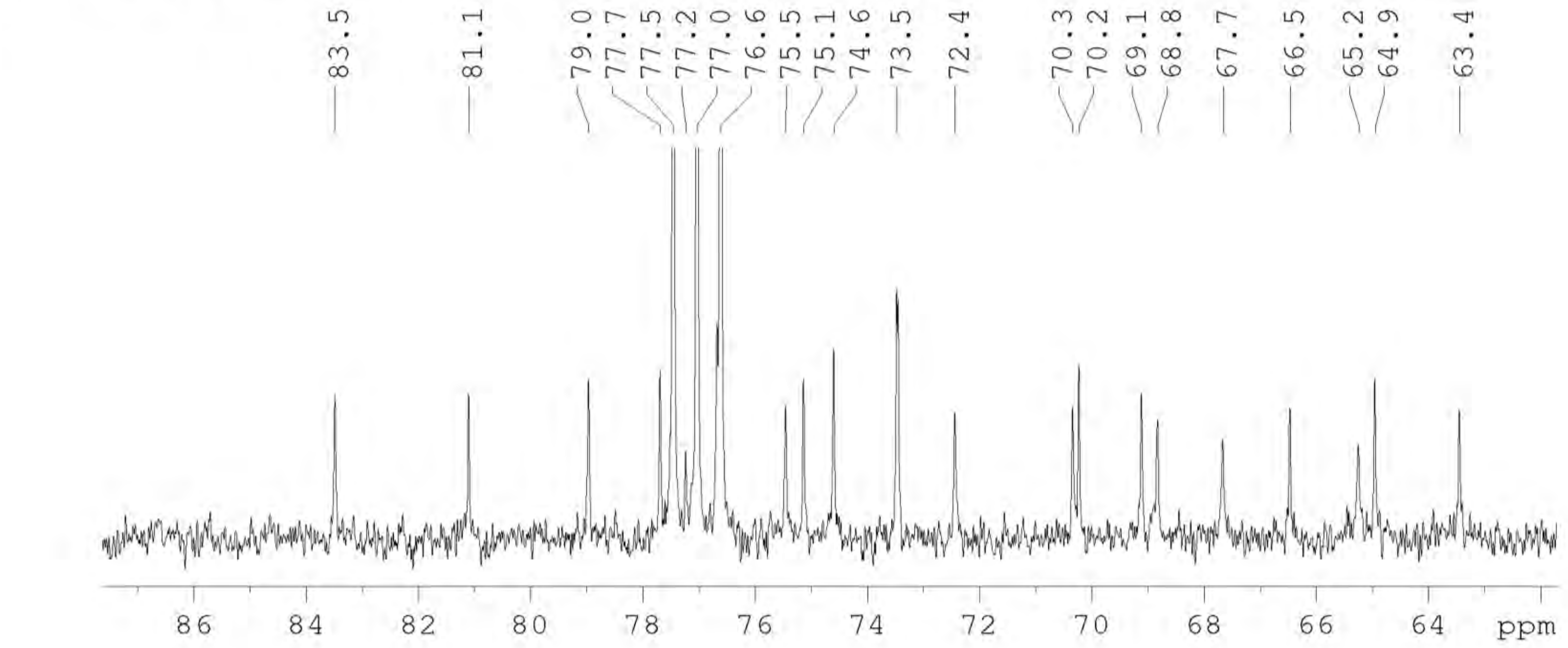

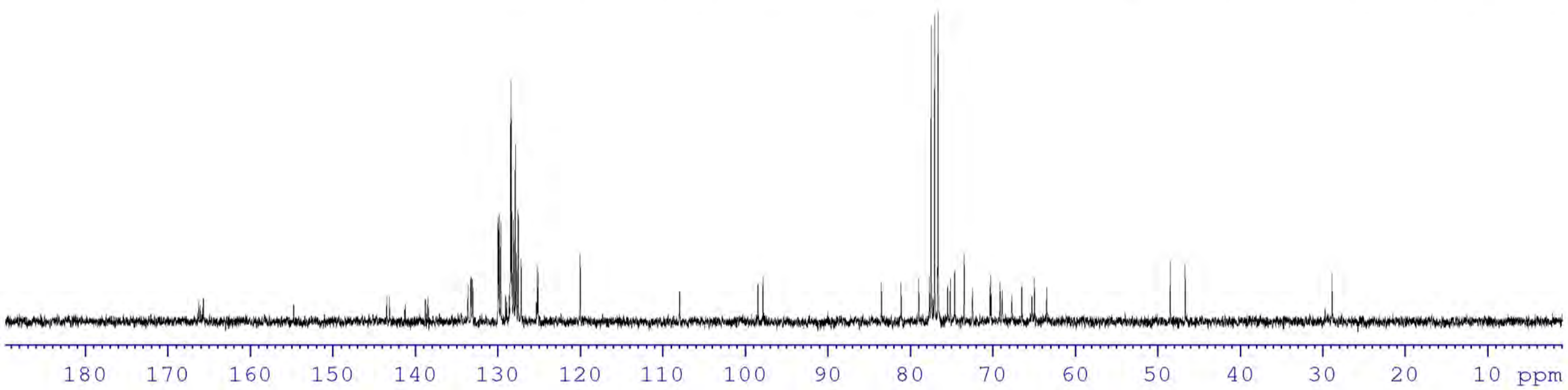

^{1}H-^{1}H COSY of **27** (300 MHz, $CDCl_3$)

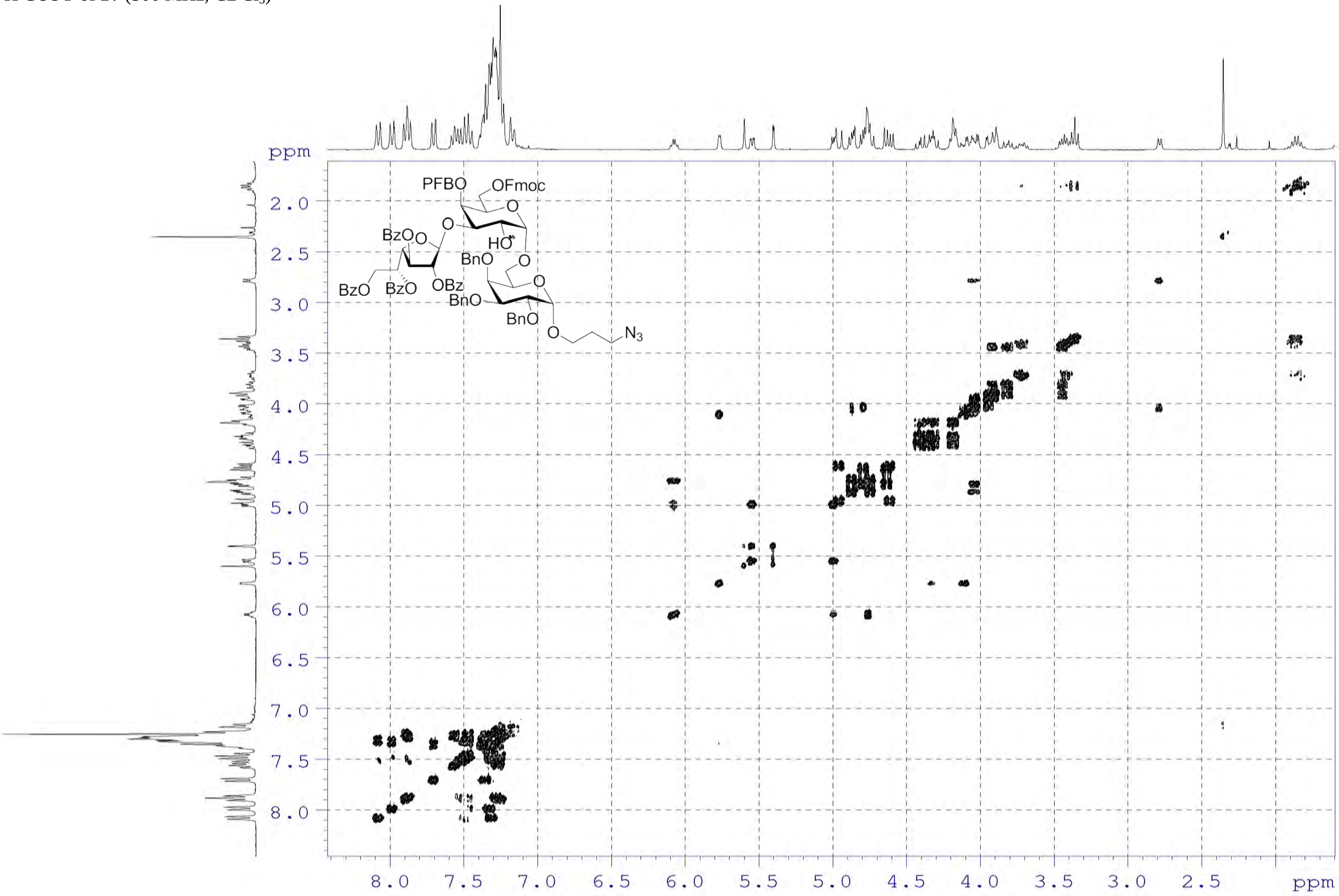

^{1}H-^{13}C HSQC of **27** (300 MHz, $CDCl_3$)

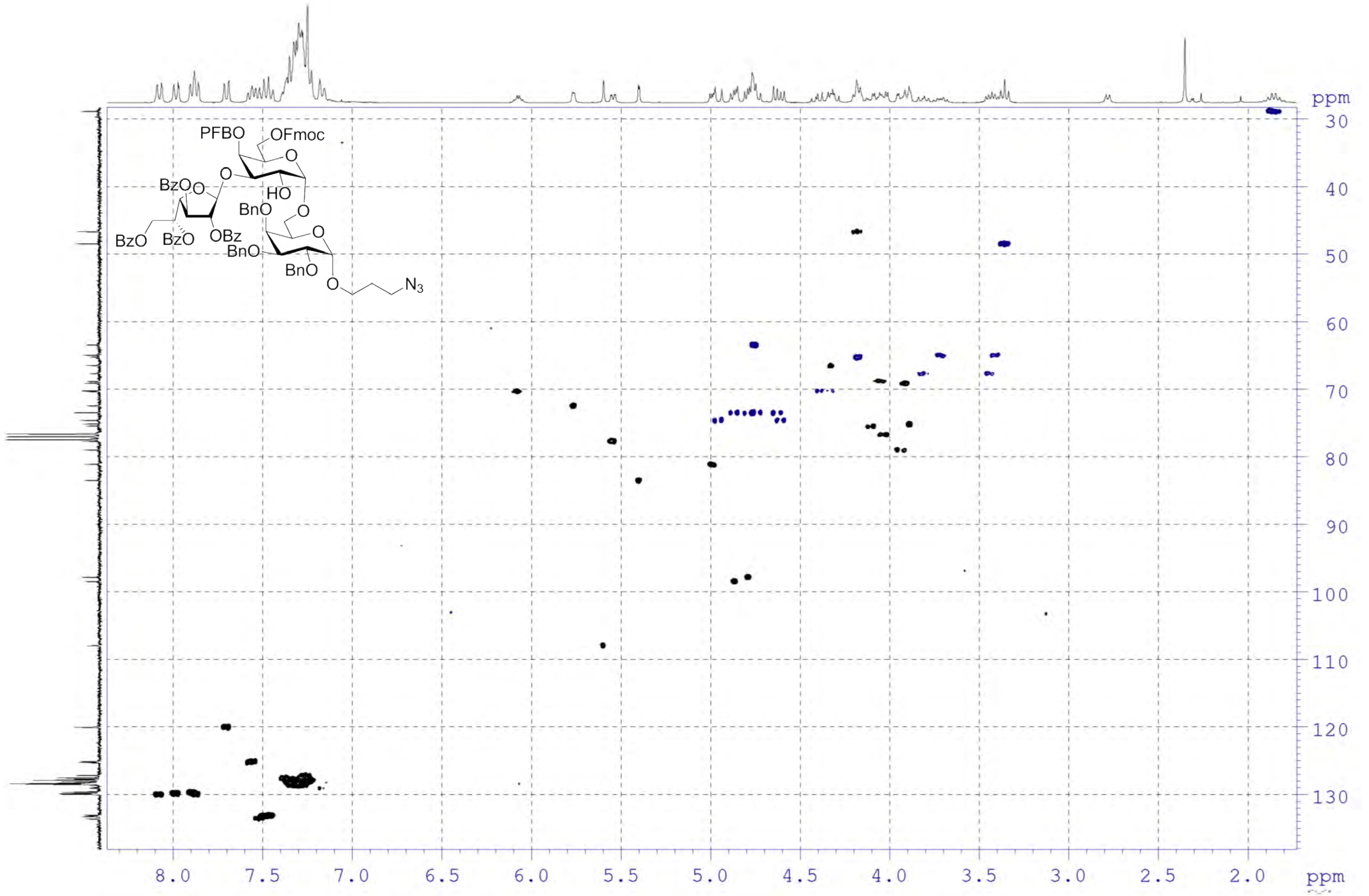

^{1}H-NMR of **28** (600 MHz, $CDCl_3$)

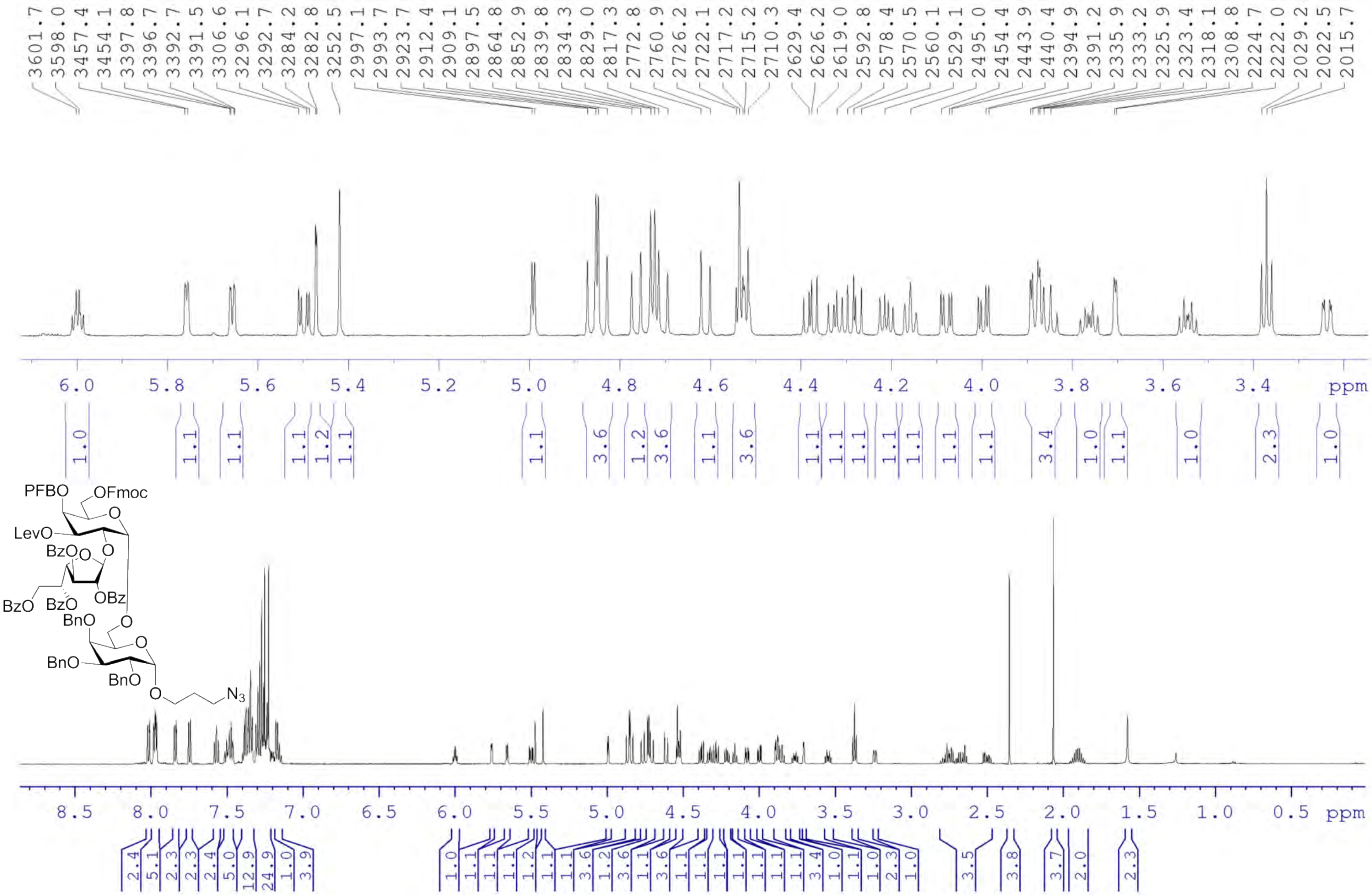

^{13}C-NMR of **28** (150 MHz, $CDCl_3$)

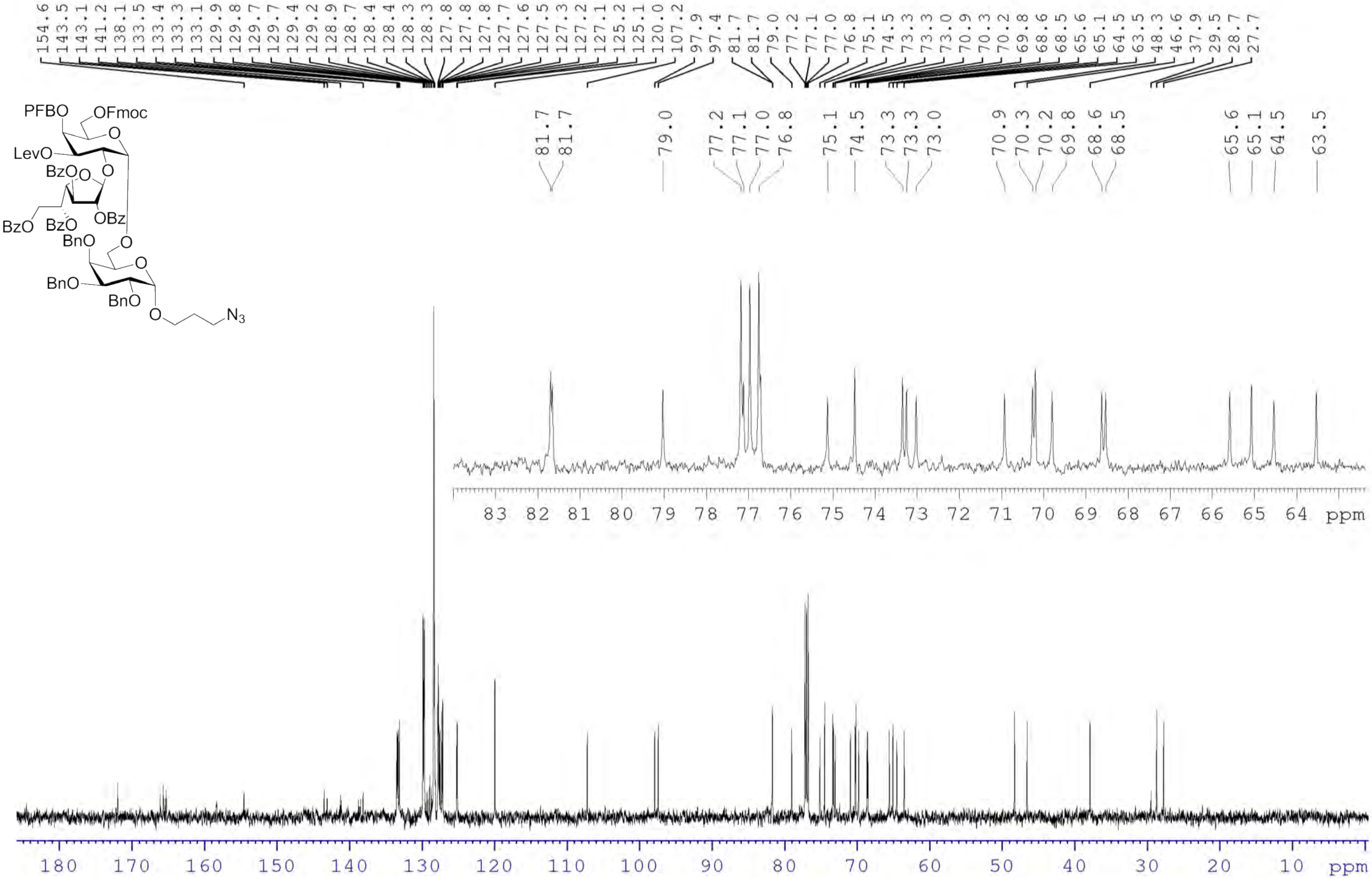

^{1}H-^{1}H COSY of **28** (600 MHz, $CDCl_3$)

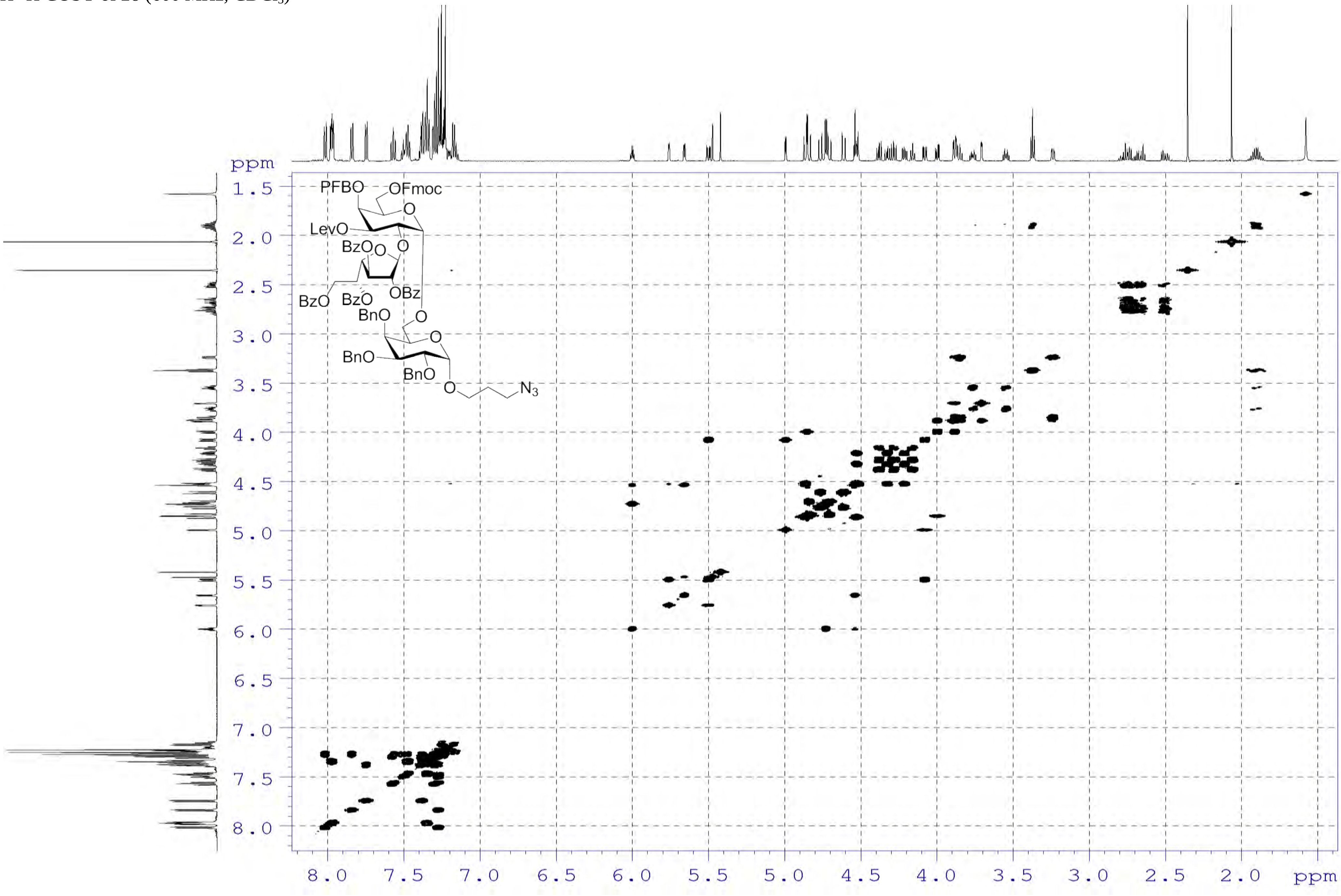

^{1}H-^{13}C HSQC of **28** (600 MHz, $CDCl_3$)

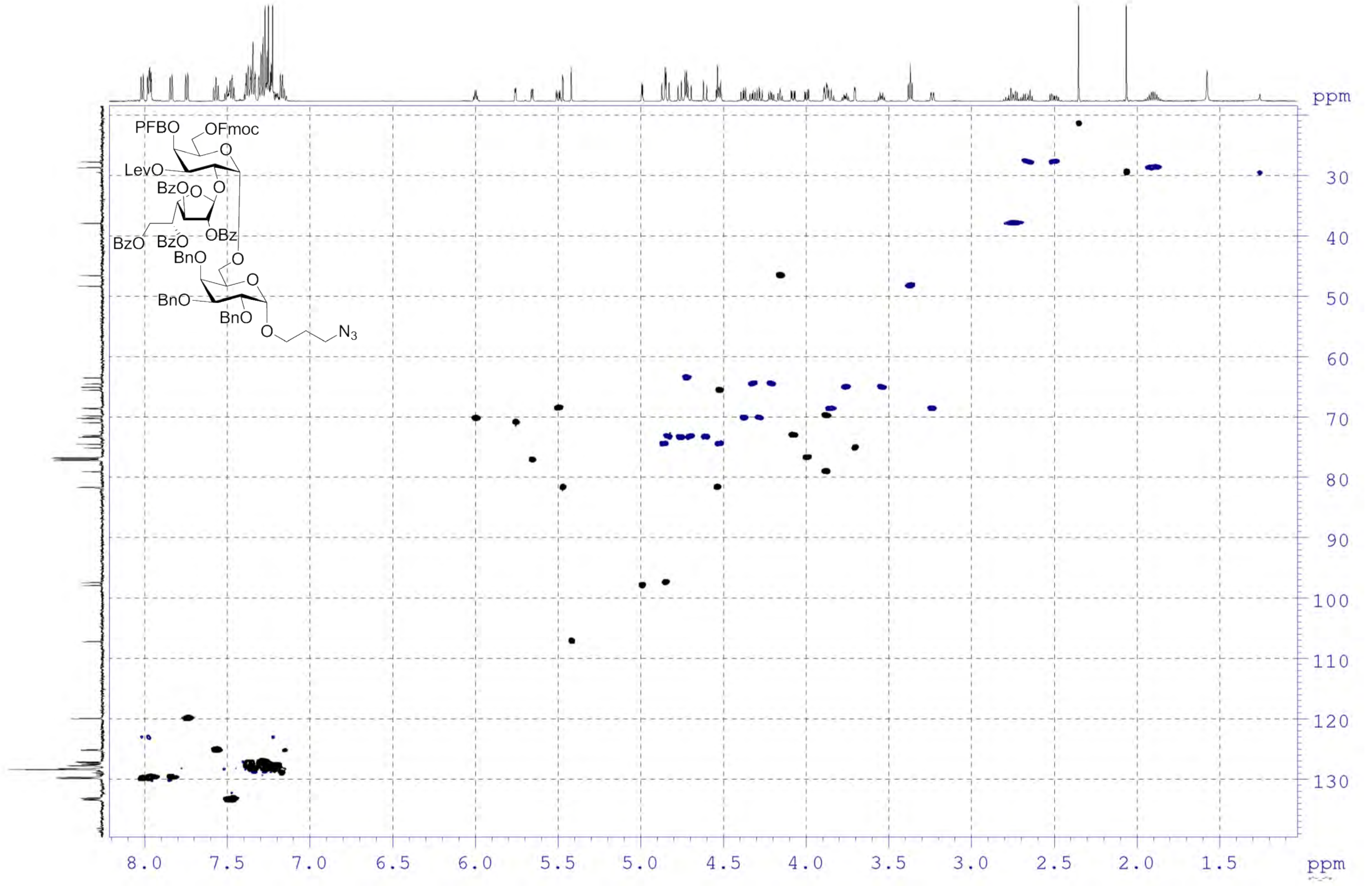

^{1}H-NMR of **29** (300 MHz, $CDCl_3$)

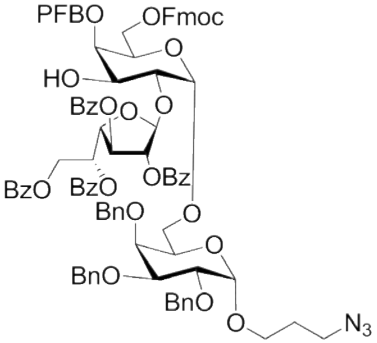

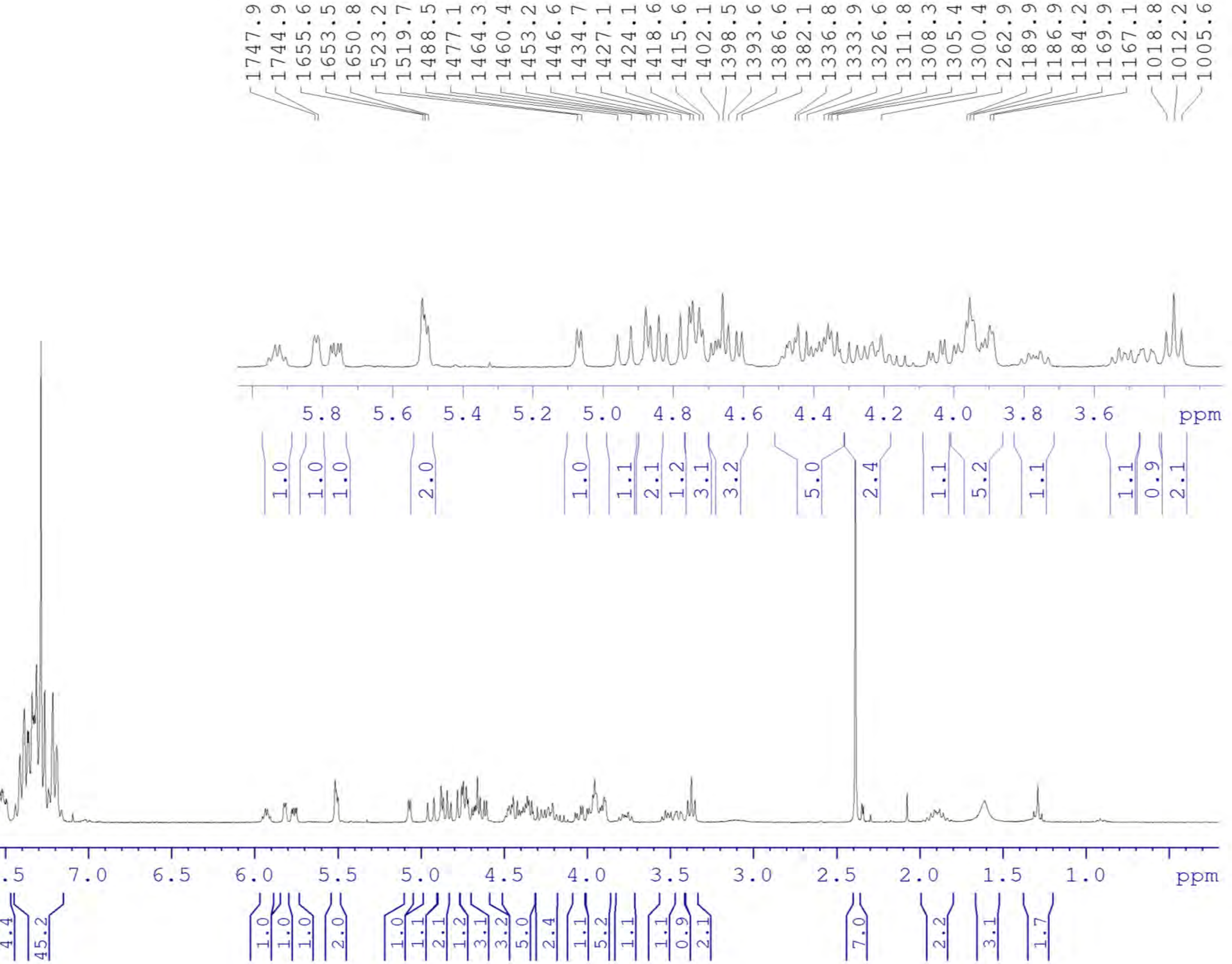

^{13}C-NMR of **29** (75 MHz, $CDCl_3$)

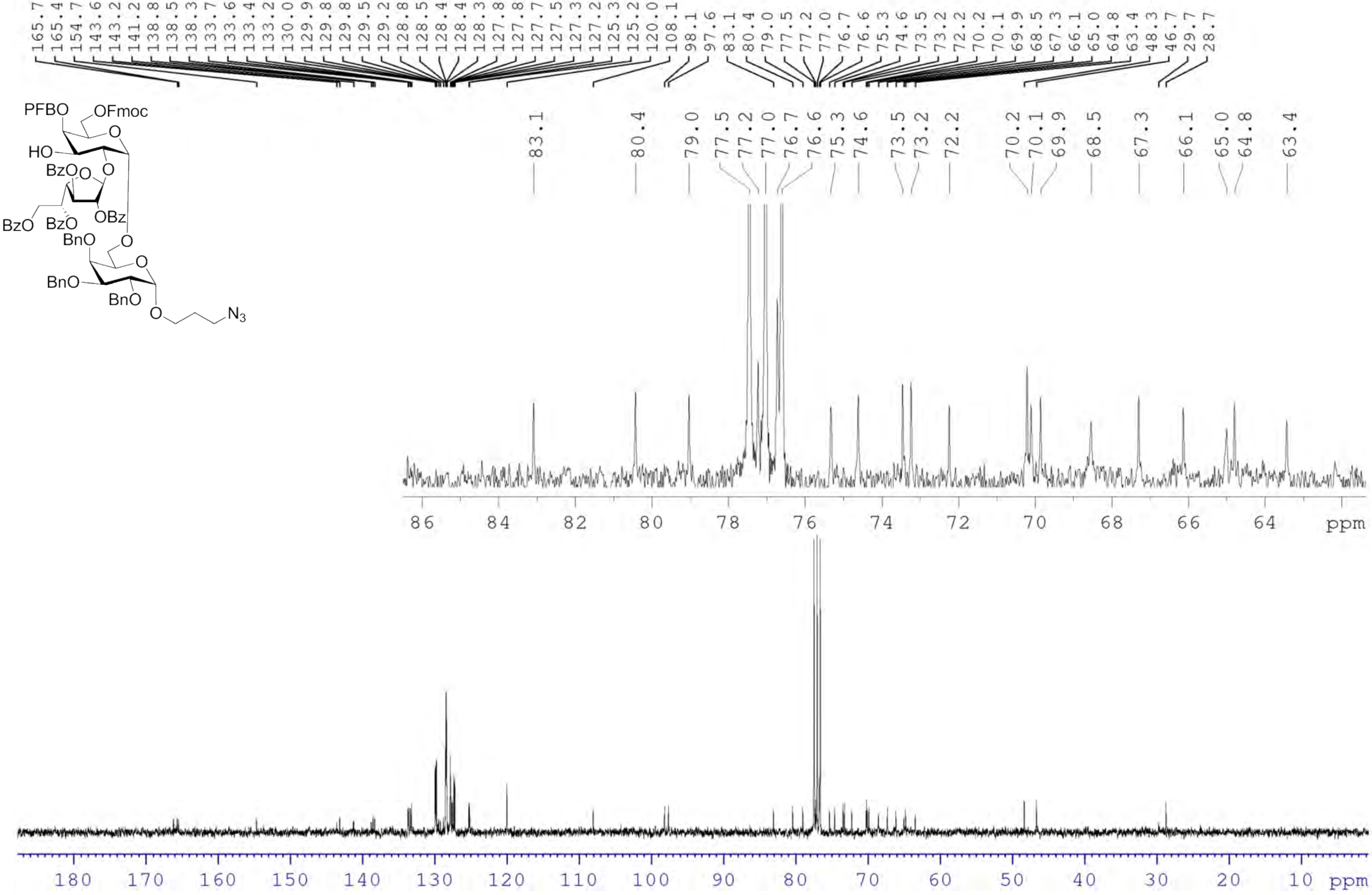

^{1}H-^{1}H COSY of **29** (300 MHz, $CDCl_3$)

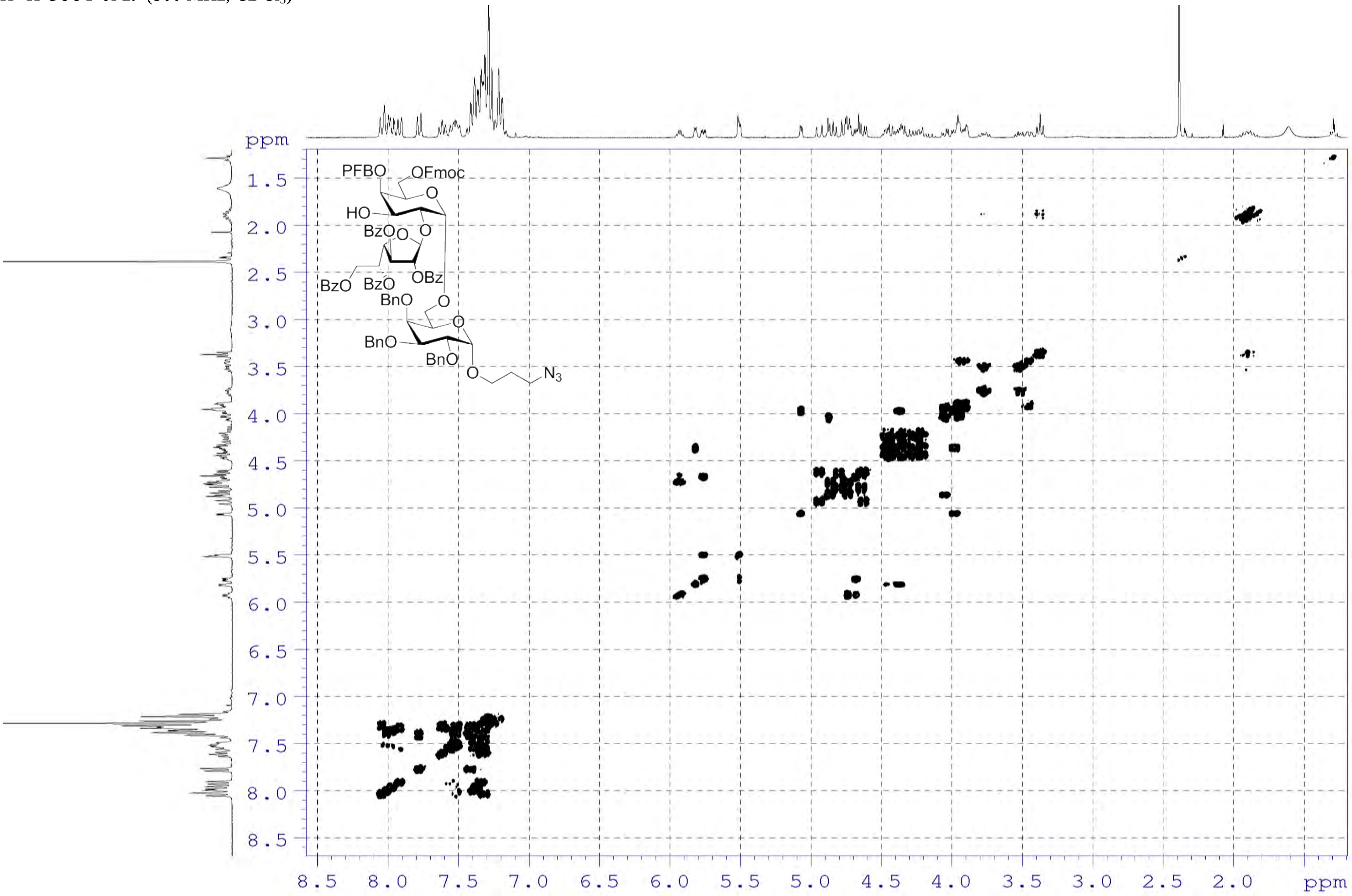

^{1}H-^{13}C HSQC of **29** (300 MHz, $CDCl_3$)

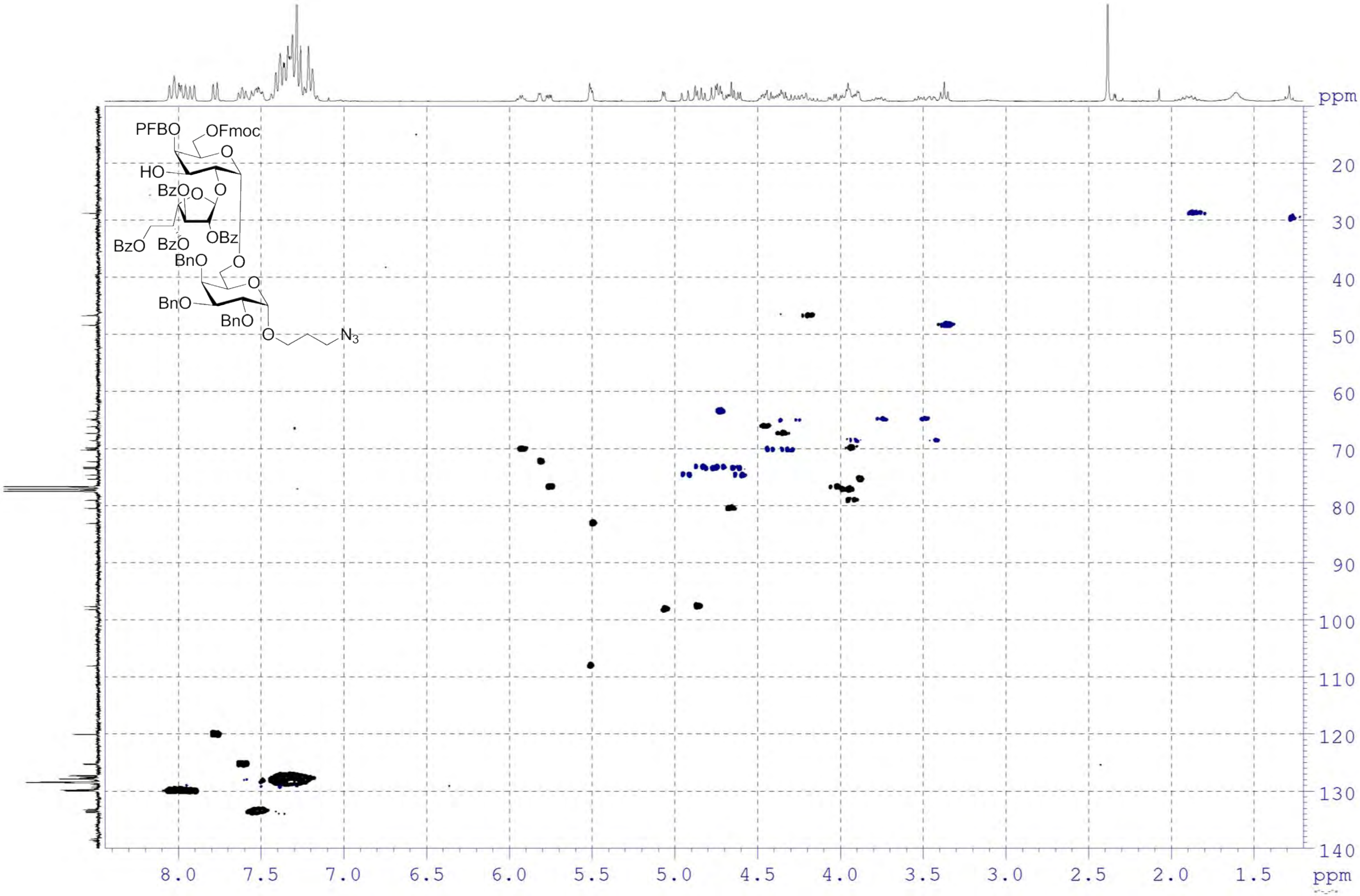

^{1}H-NMR of **29i** (300 MHz, $CDCl_3$)

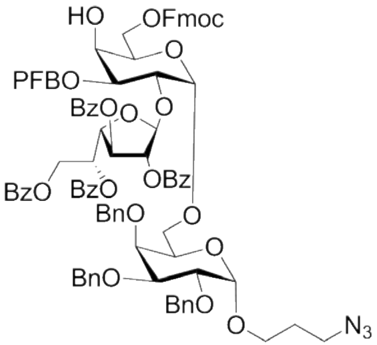

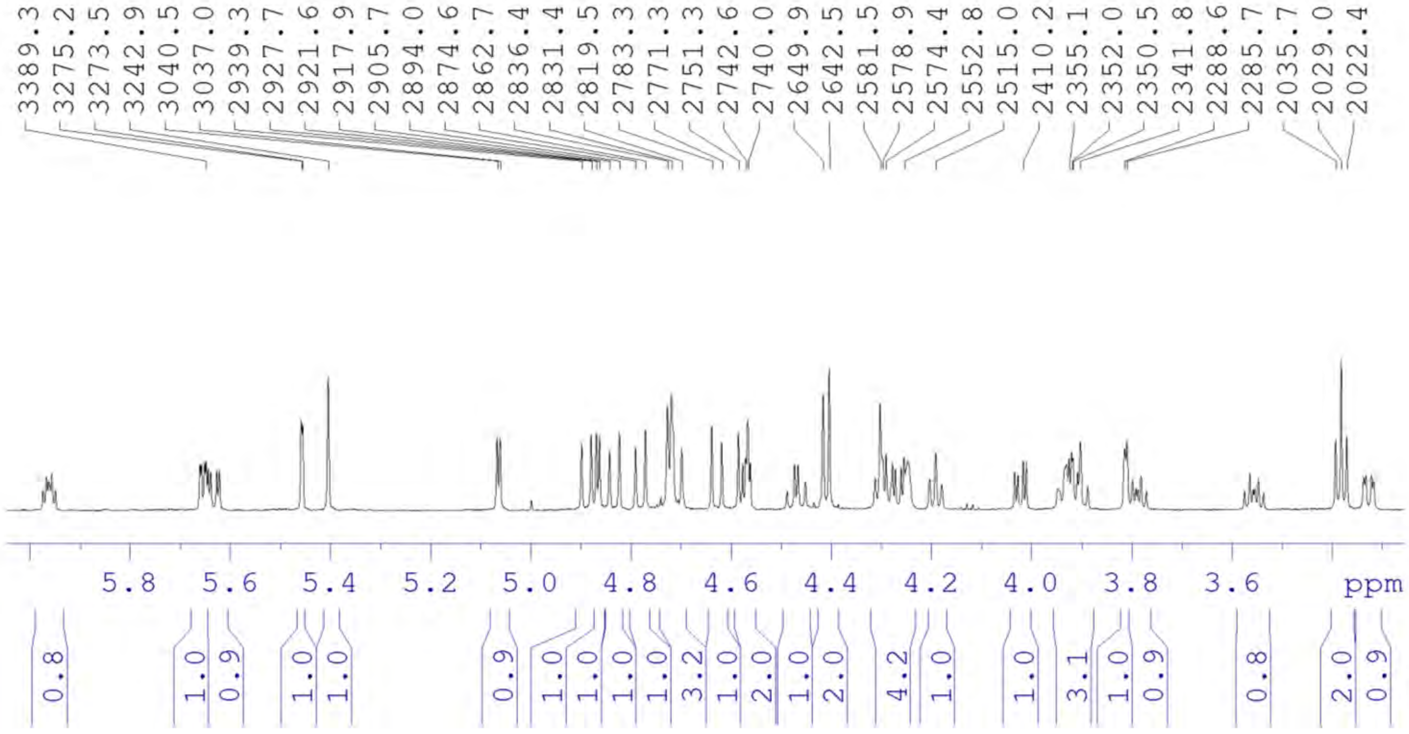

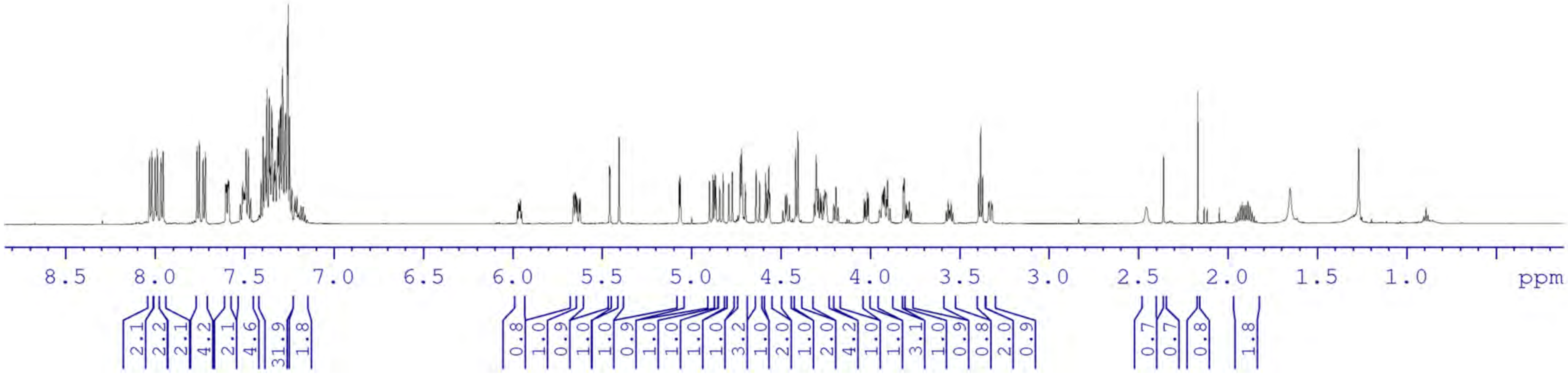

^{13}C-NMR of **29i** (75 MHz, $CDCl_3$)

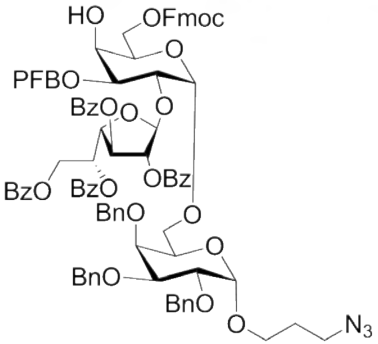

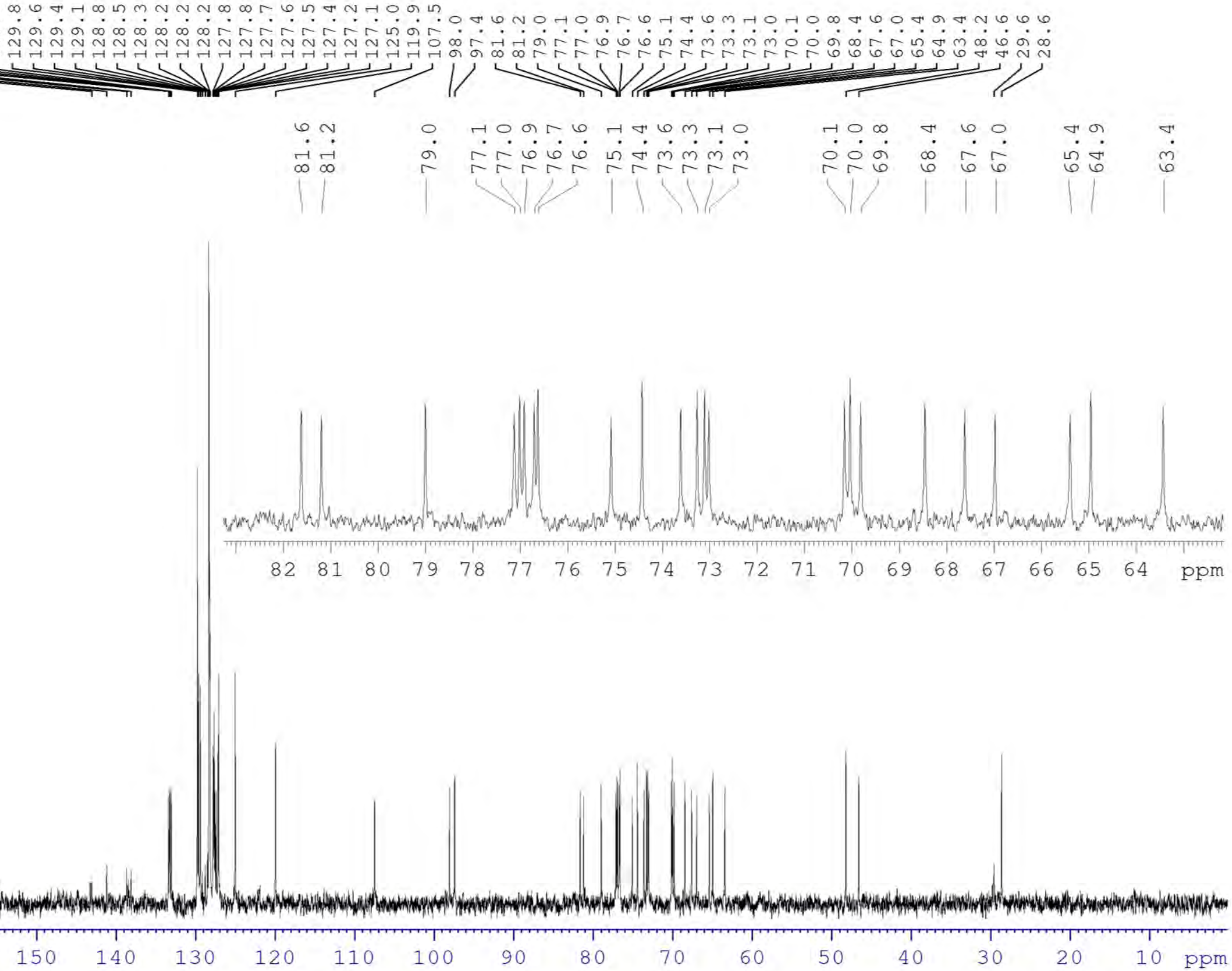

^{1}H-^{1}H COSY of **29i** (300 MHz, $CDCl_3$)

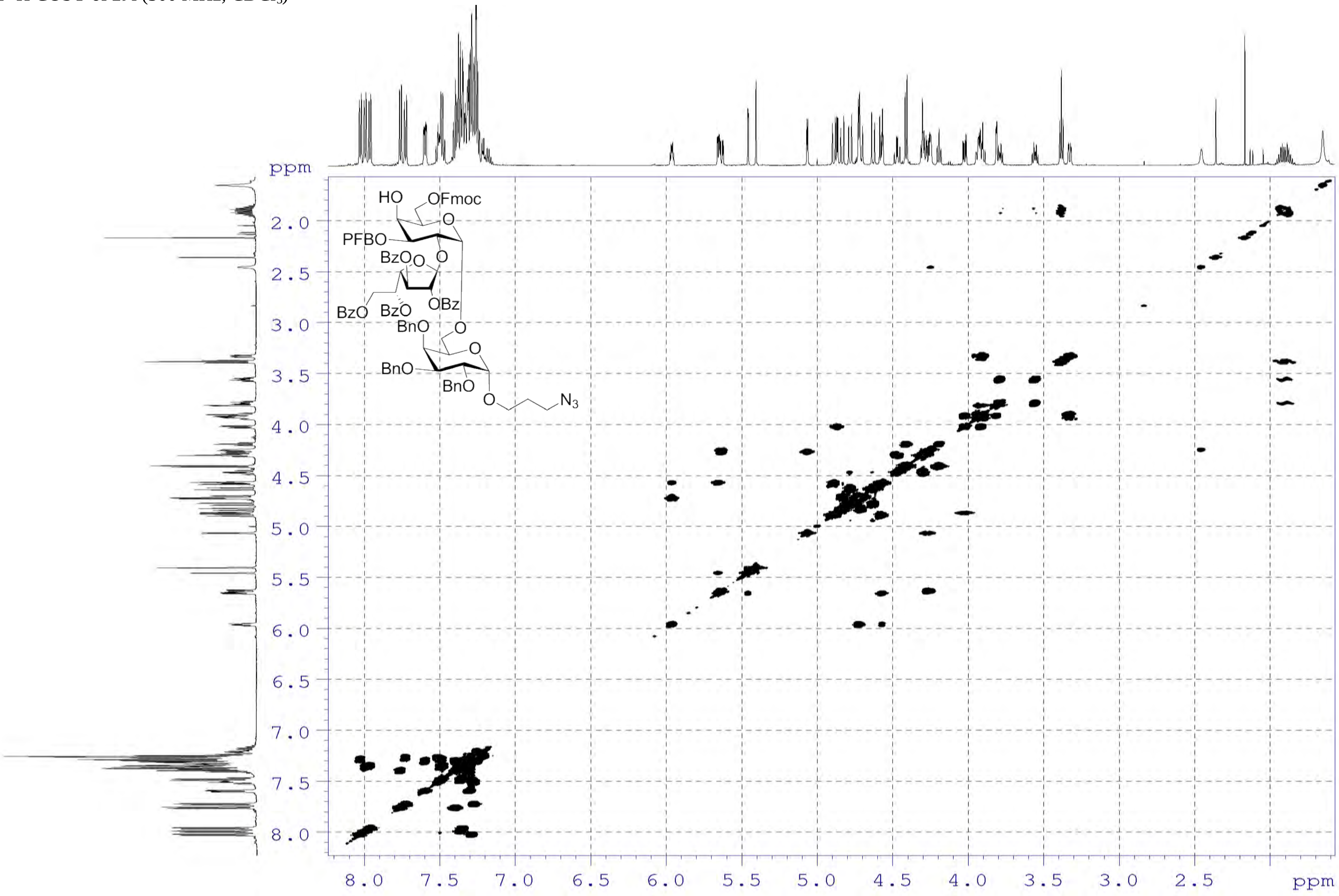

^{1}H-^{13}C HSQC of **29i** (300 MHz, $CDCl_3$)

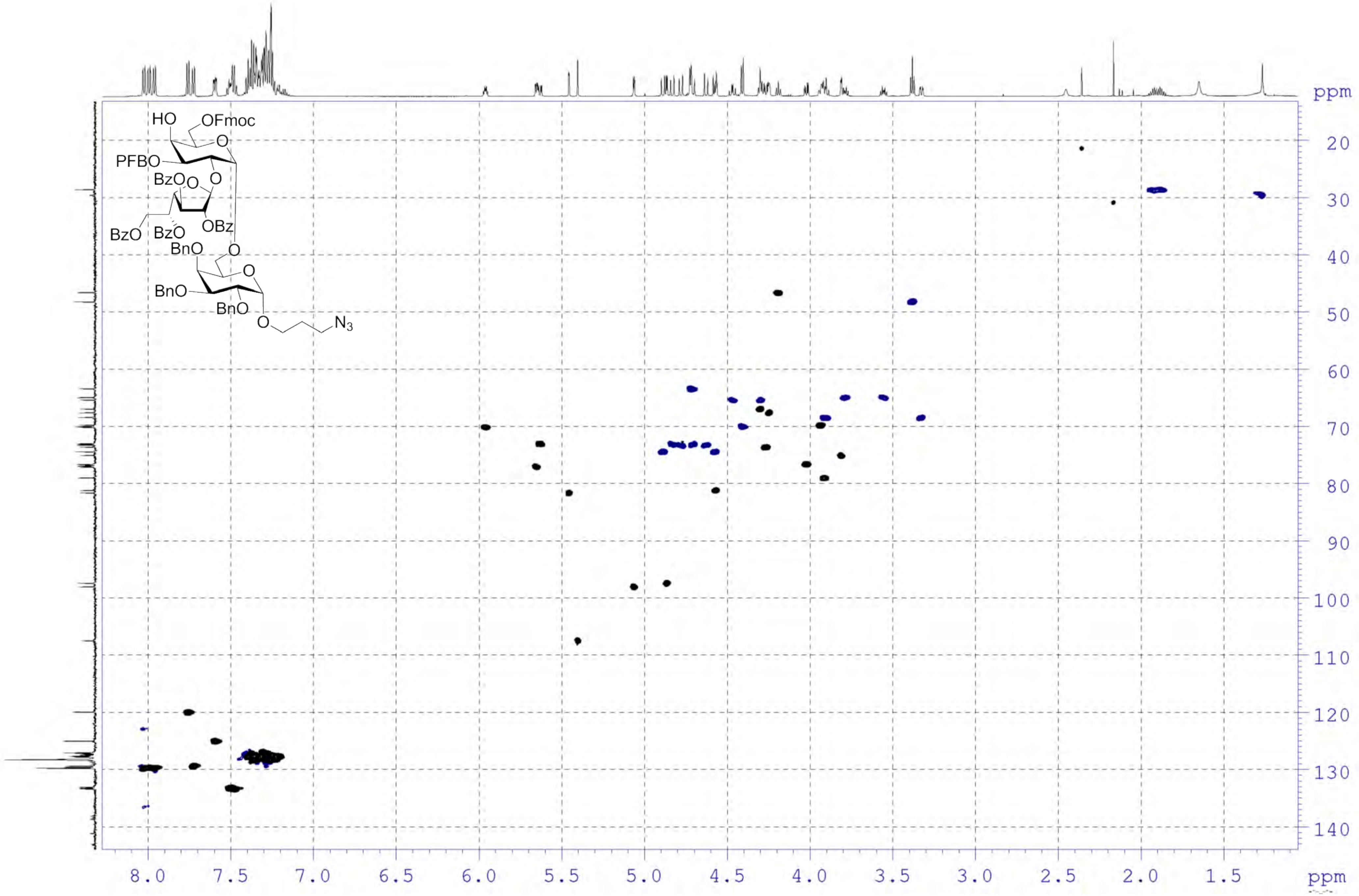

^{1}H-NMR of **30** (300 MHz, $CDCl_3$)

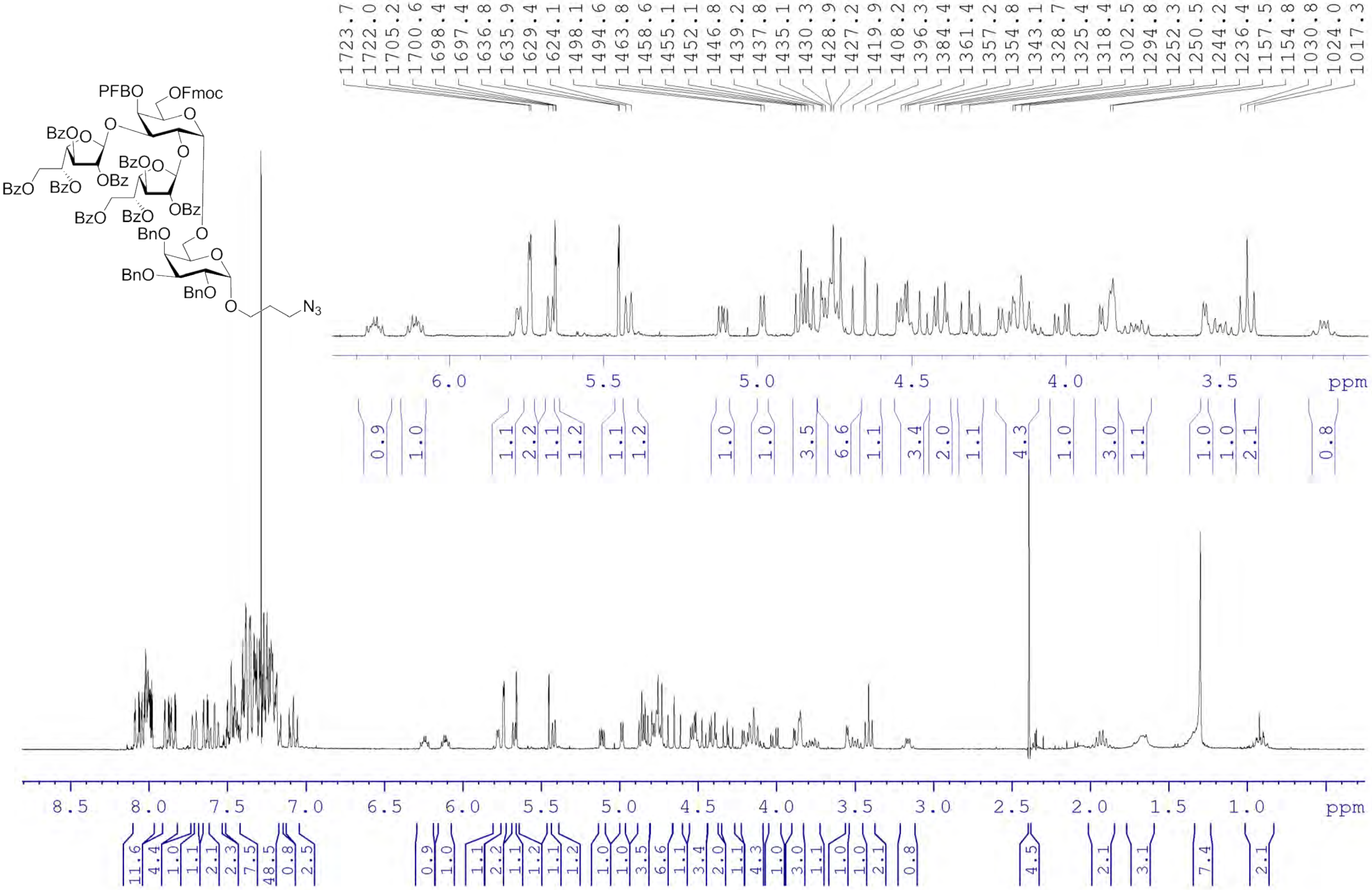

^{13}C-NMR of **30** (75 MHz, $CDCl_3$)

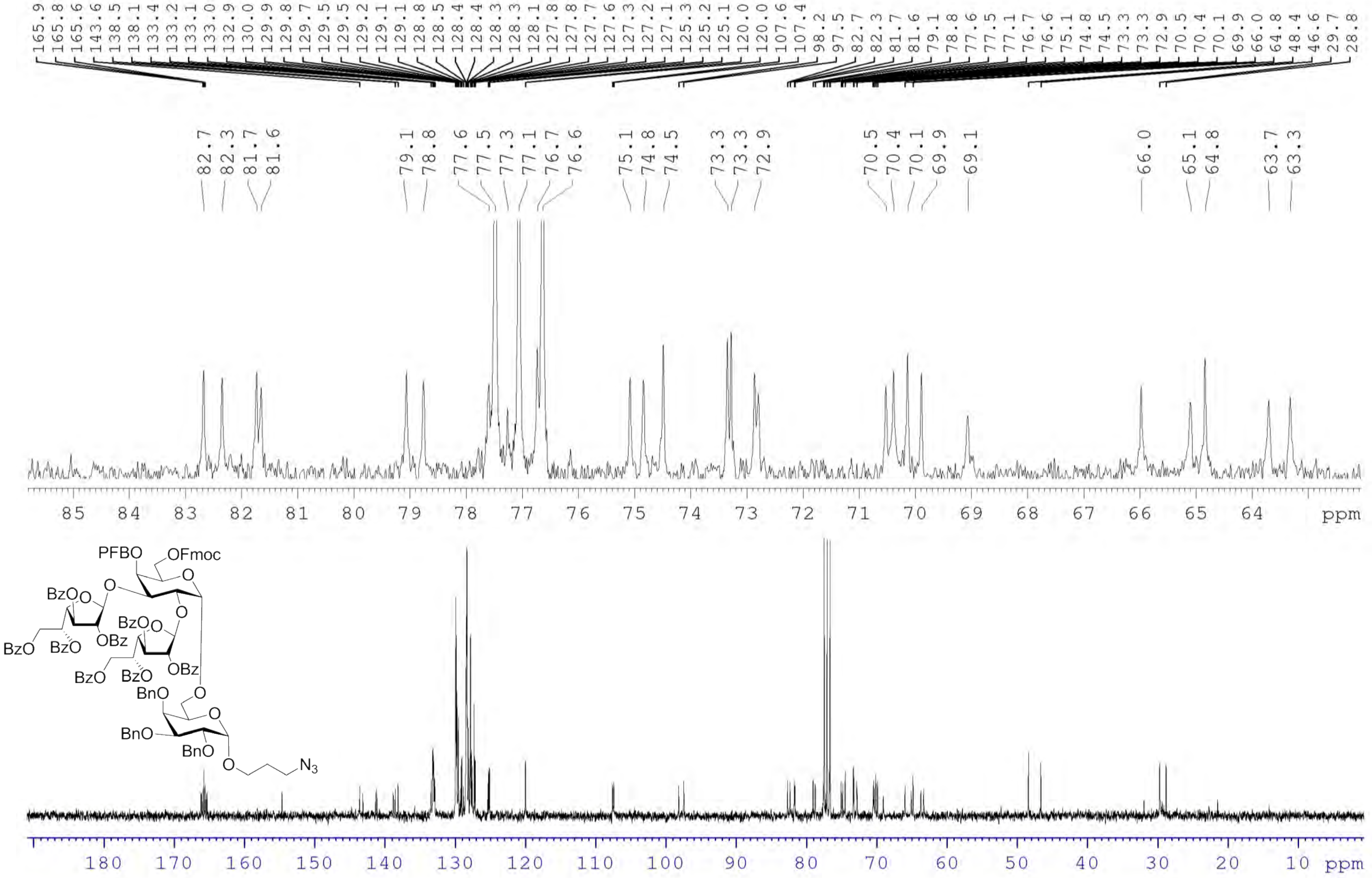

^{1}H-^{1}H COSY of **30** (300 MHz, $CDCl_3$)

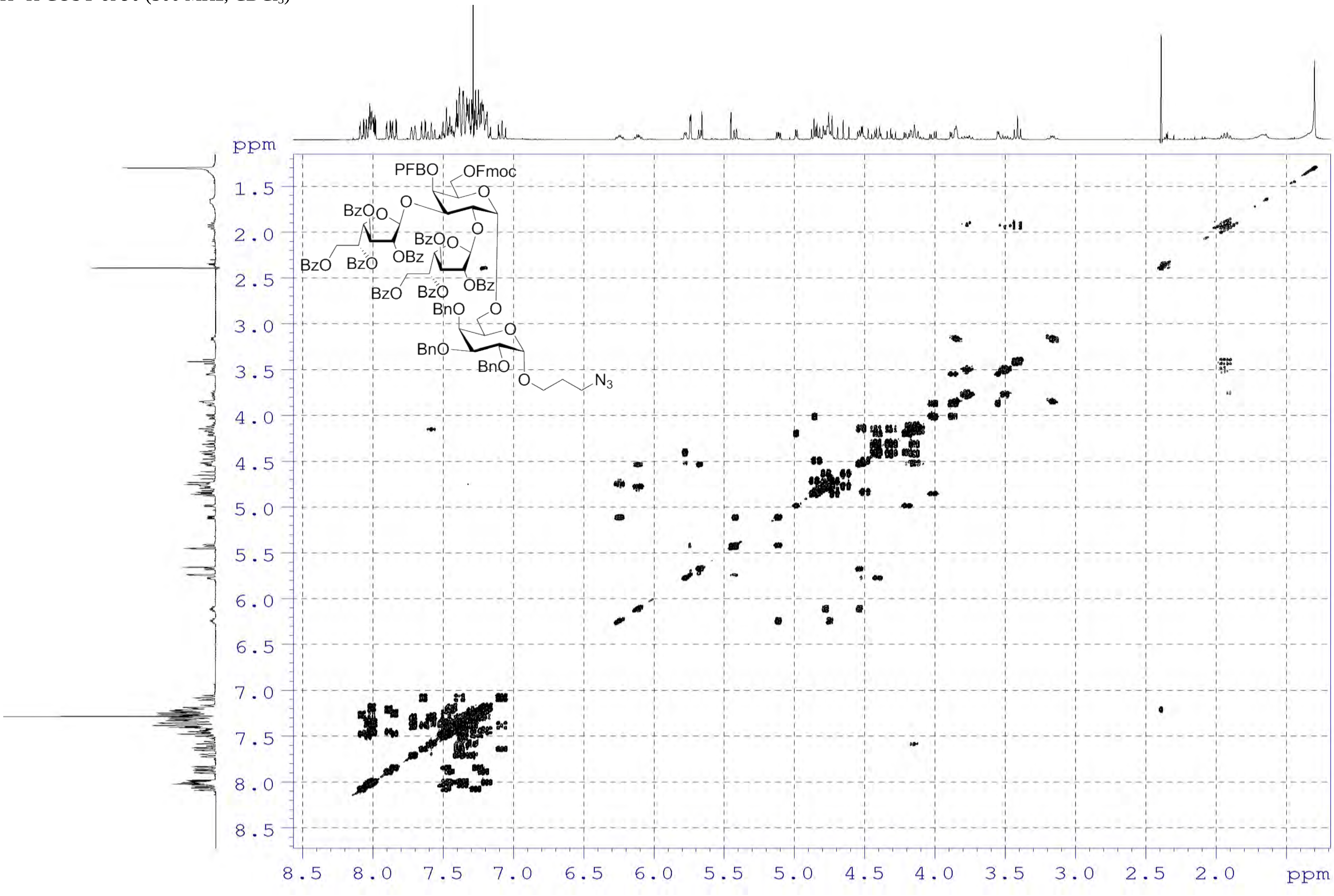

^{1}H-^{13}C HSQC of **30** (300 MHz, $CDCl_3$)

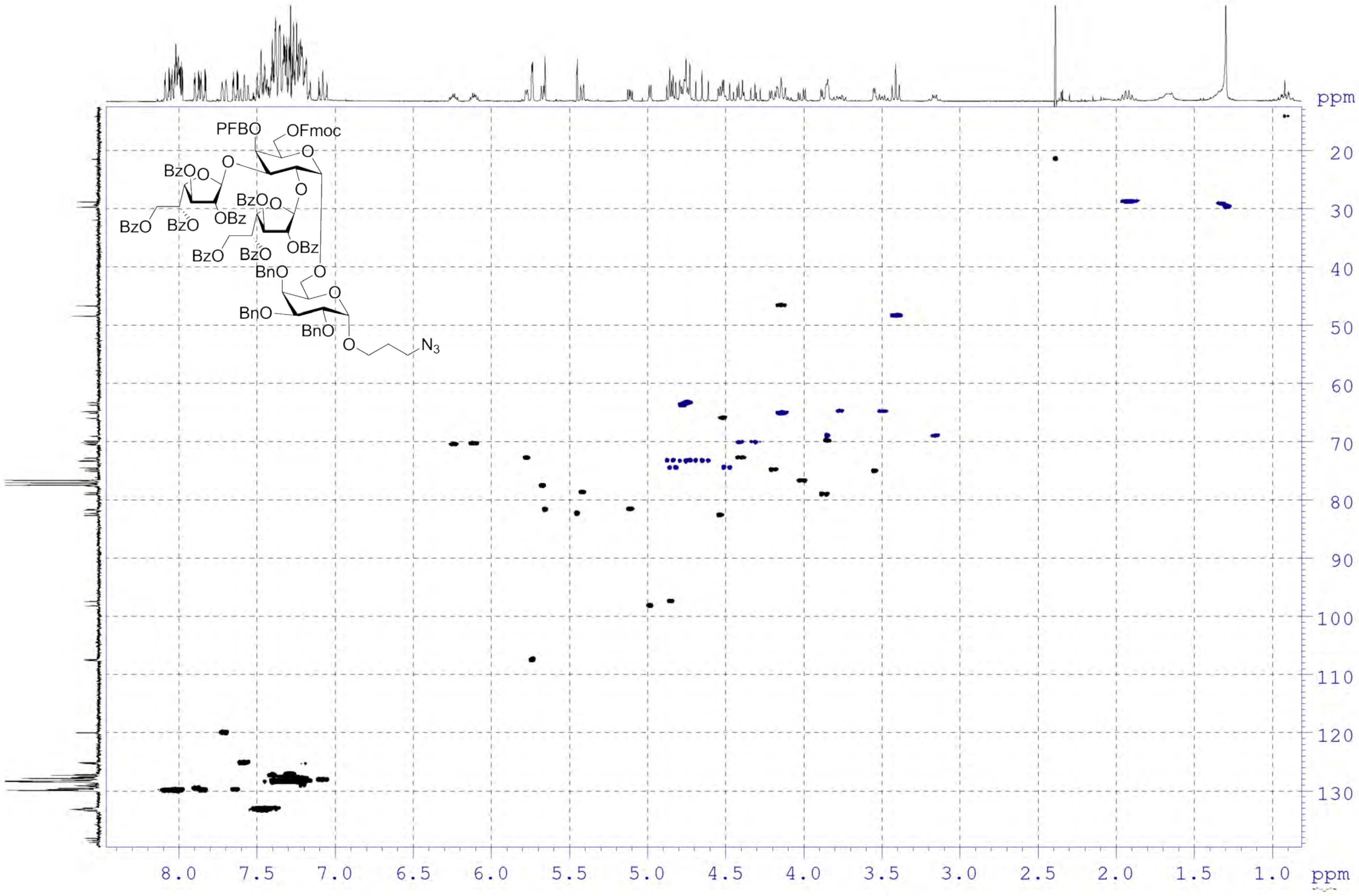

^{1}H-NMR of **S4** (300 MHz, $CDCl_3 + CD_3OD$)

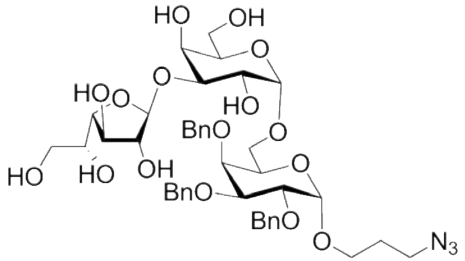

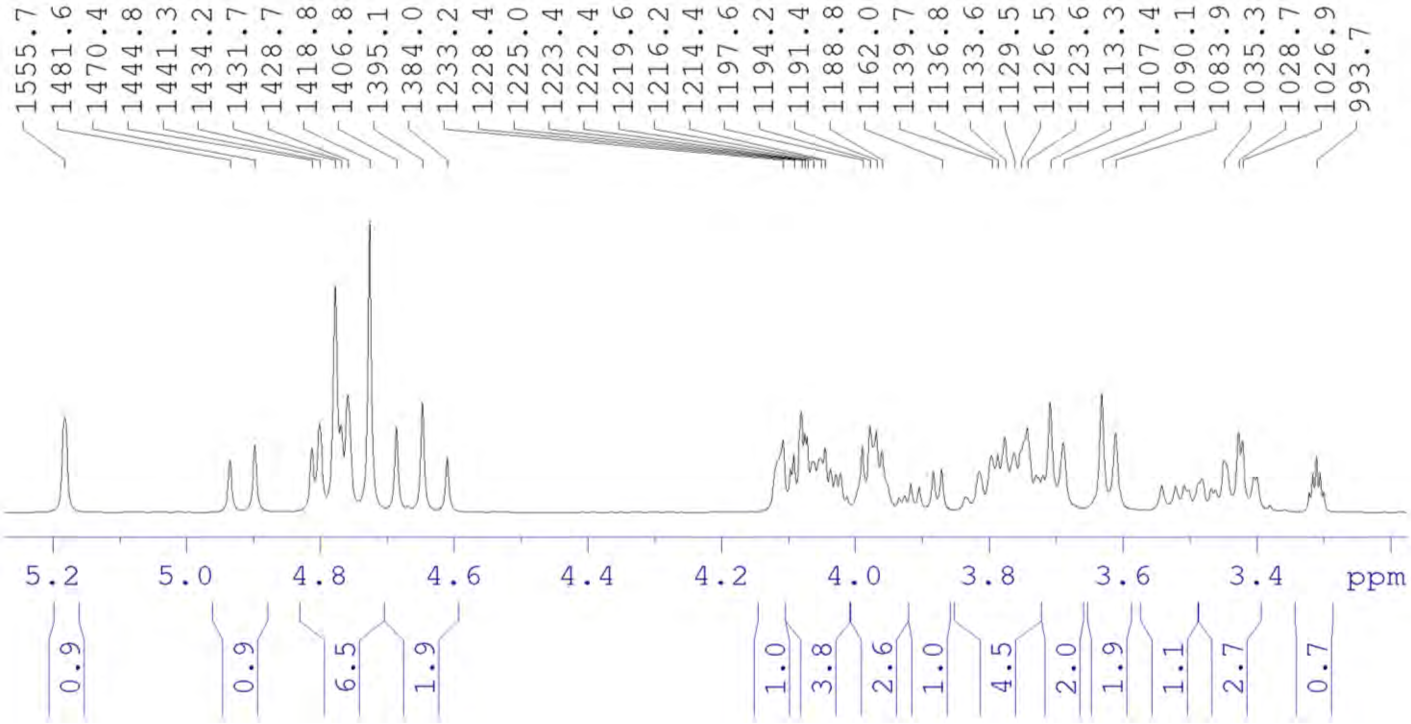

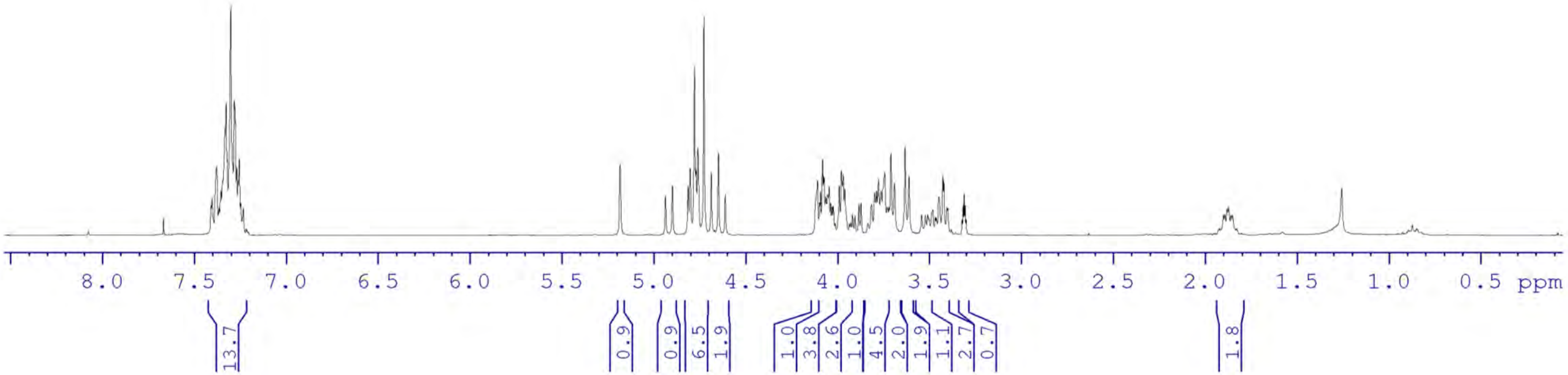

^{13}C-NMR of **S4** (75 MHz, $CDCl_3$ + CD_3OD)

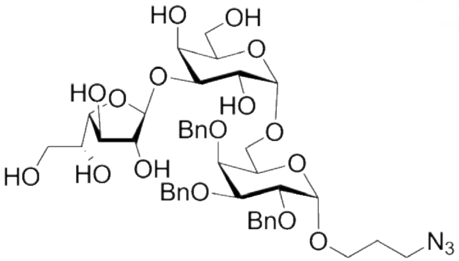

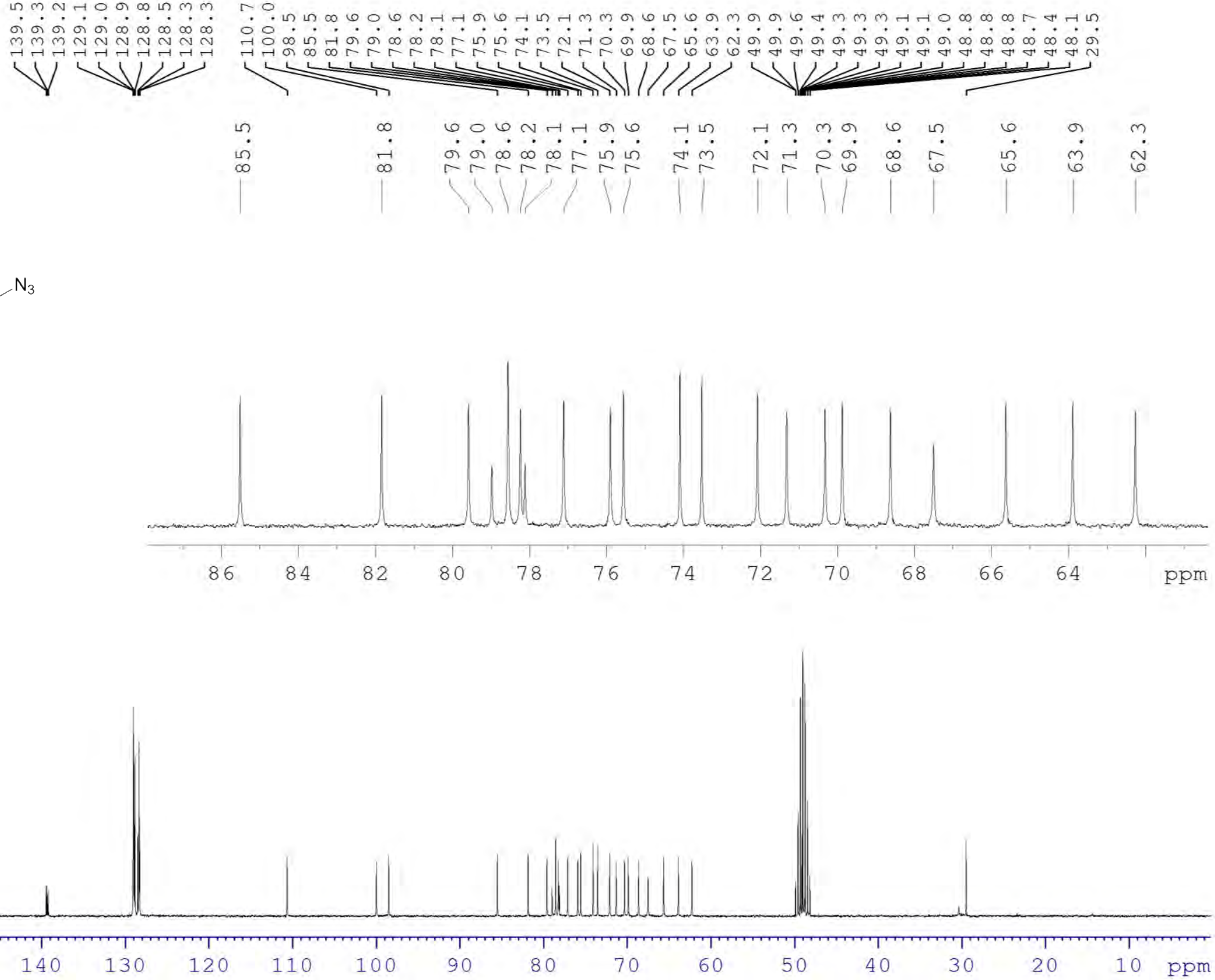

^{1}H-^{1}H COSY of **S4** (300 MHz, $CDCl_3 + CD_3OD$)

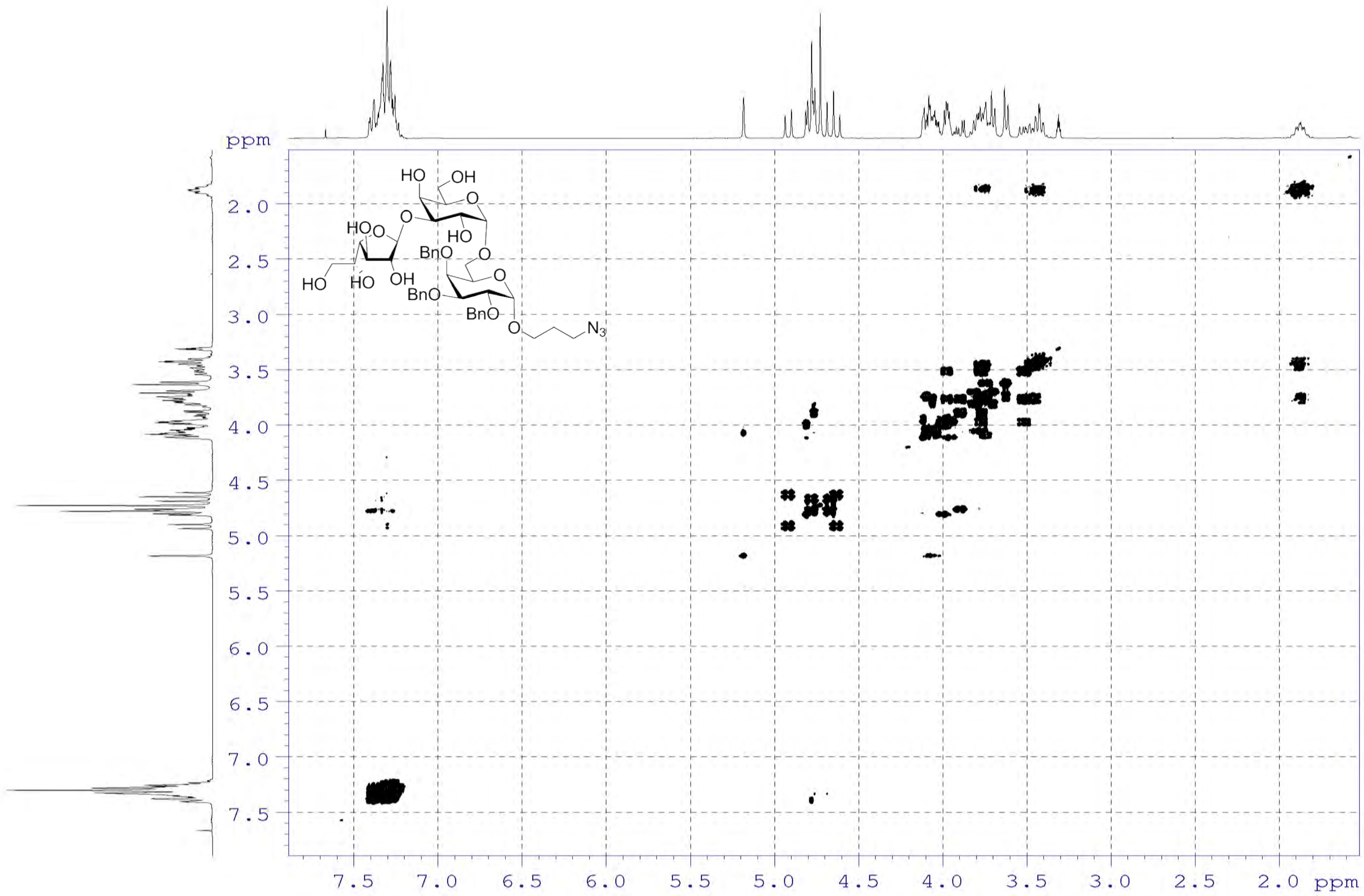

^{1}H-^{13}C HSQC of **S4** (300 MHz, $CDCl_3$ + CD_3OD)

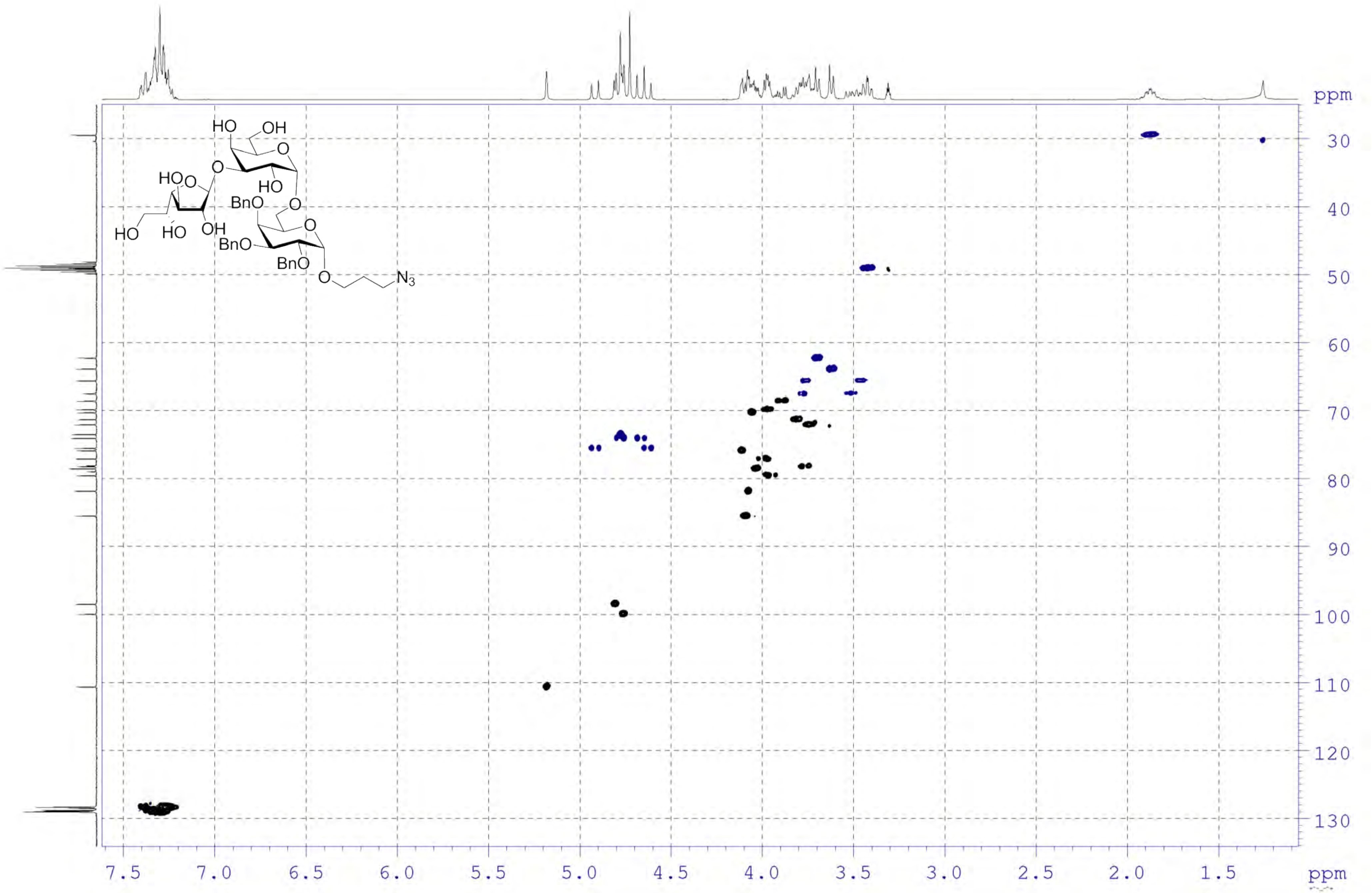

^{1}H-NMR of **2a** (600 MHz, D_2O, 303K)

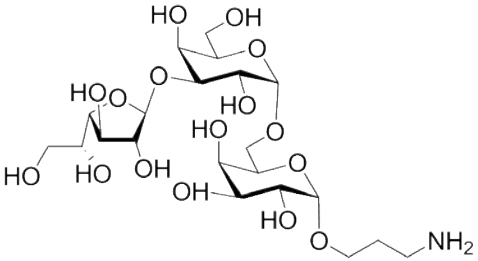

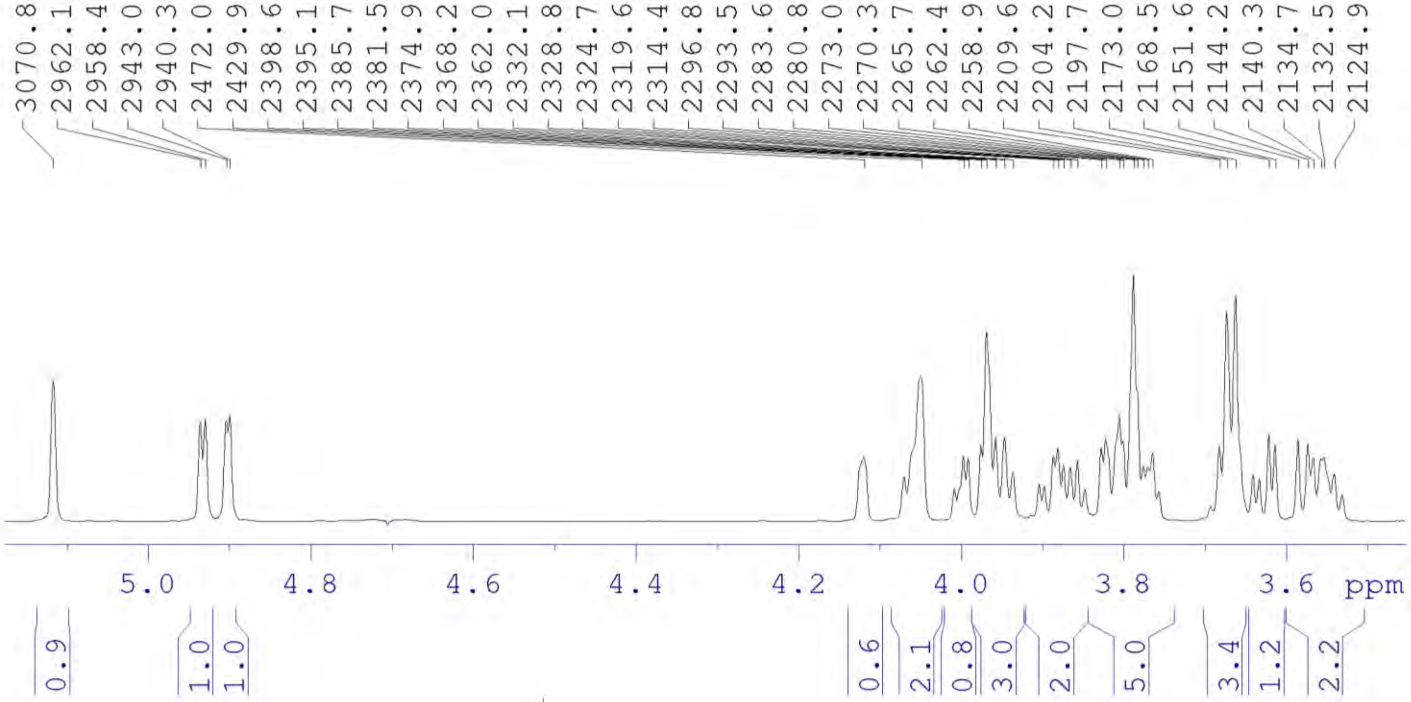

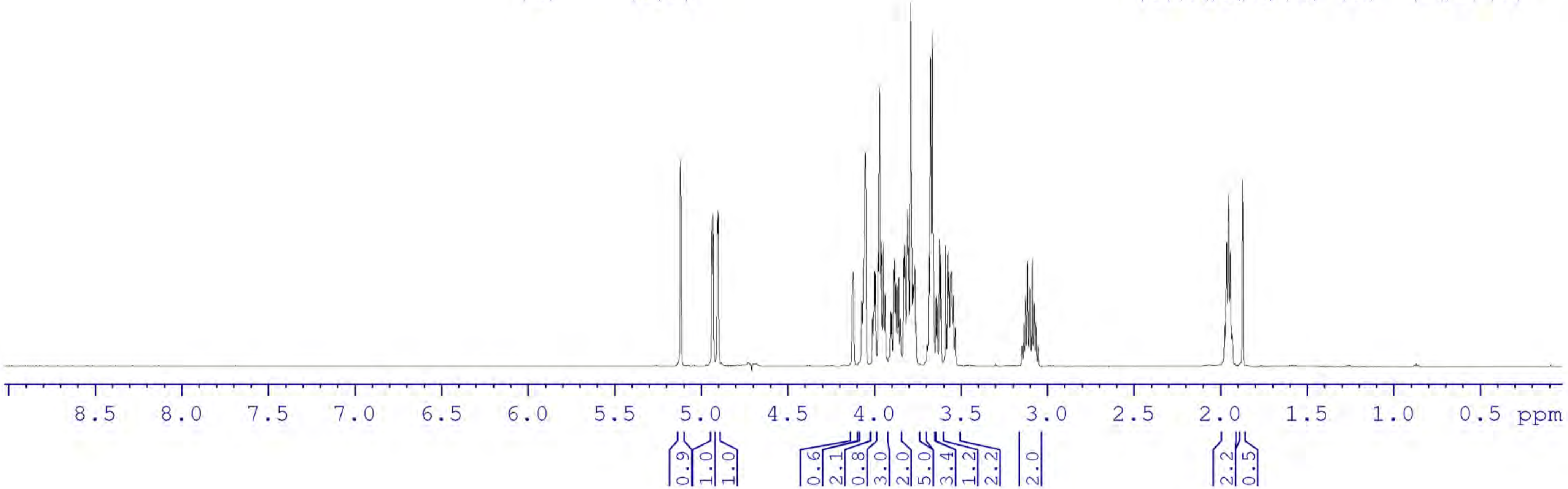

^{13}C-NMR of **2a** (150 MHz, D_2O, 303K)

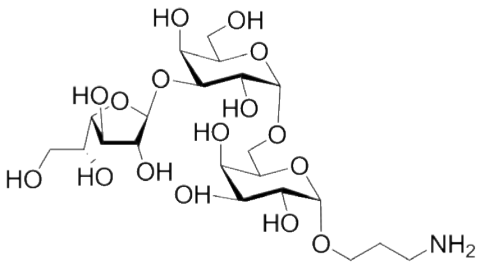

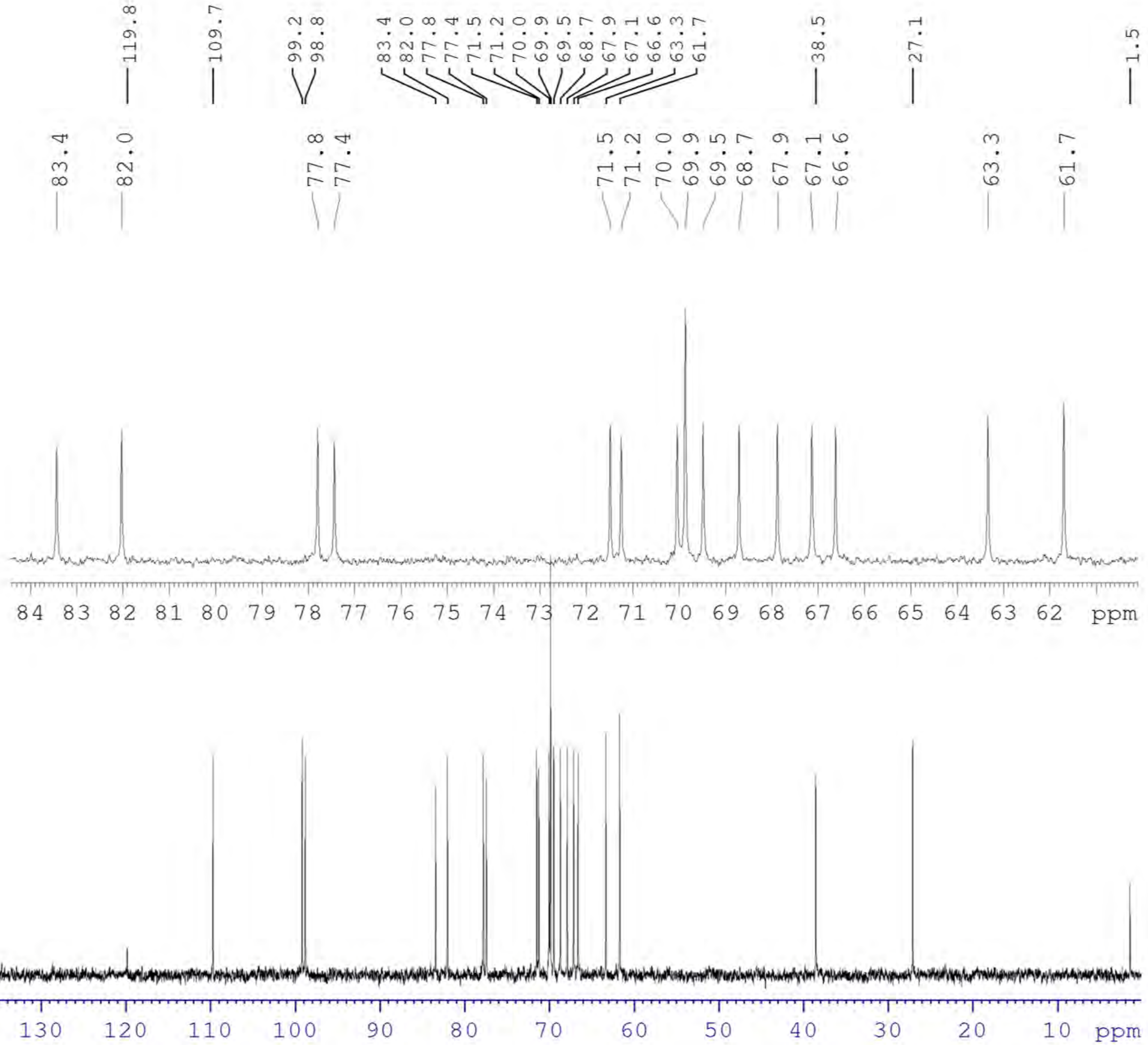

^{1}H-^{1}H COSY of **2a** (600 MHz, D_2O, 303K)

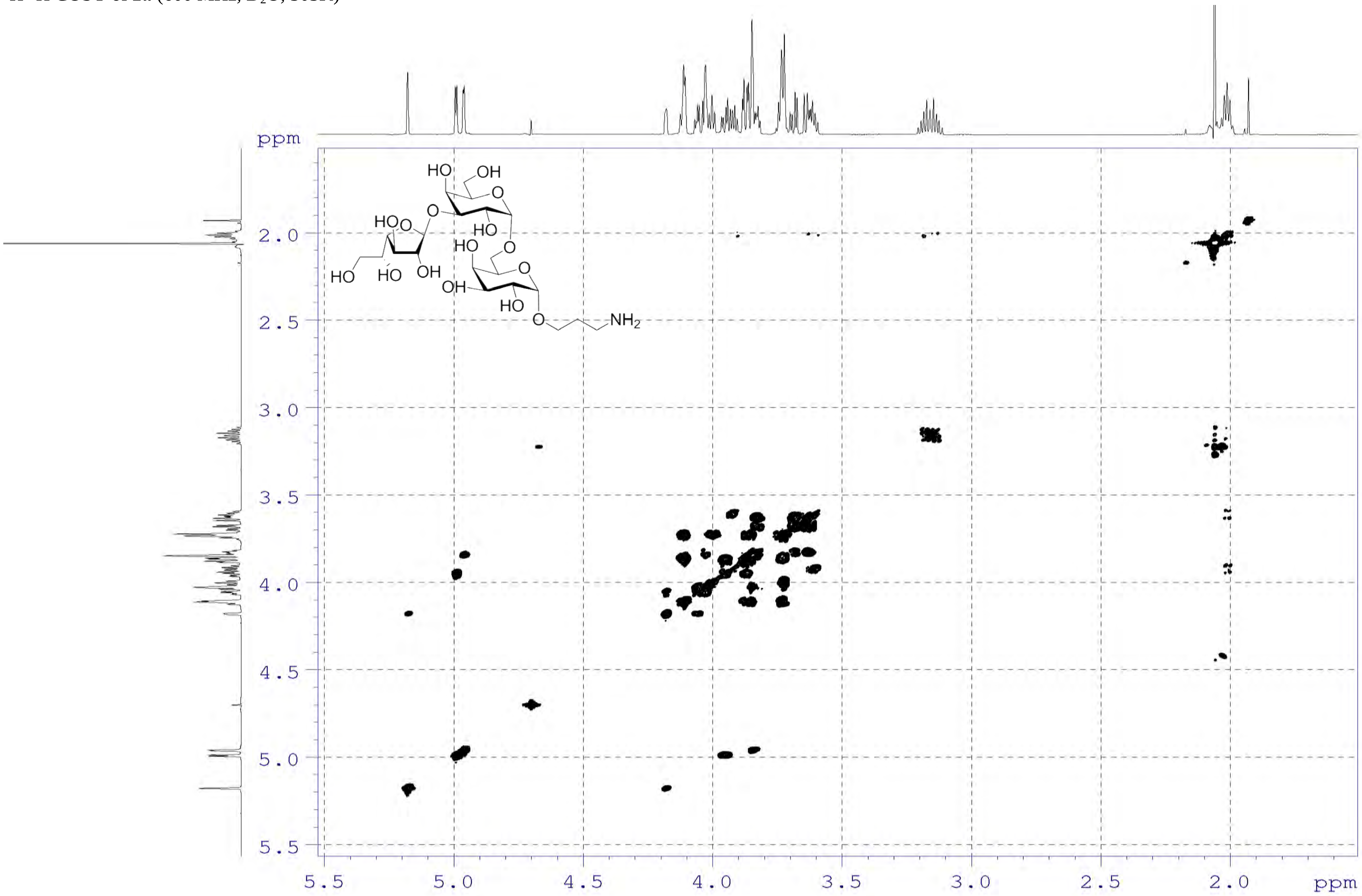

^{1}H-^{13}C HSQC of **2a** (600 MHz, D_2O, 303K)

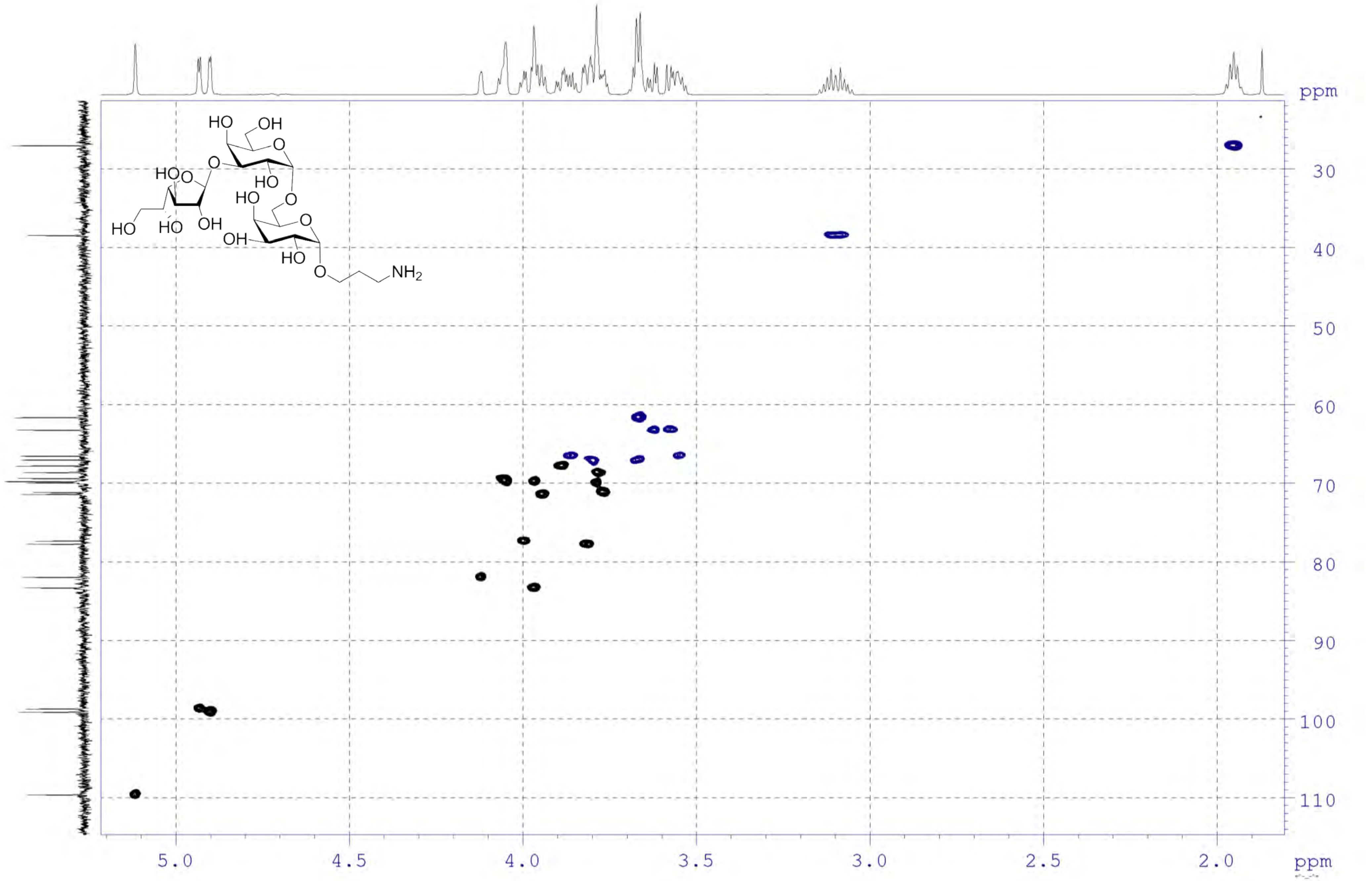

HRMS for **2a**

Comment	CH3CN : H2O 50/50 %, dil. 2000, calibrant added				

Acquisition Parameter

Source Type	ESI	Ion Polarity	Positive	Set Nebulizer	0.4 Bar
Focus	Not active			Set Dry Heater	180 °C
Scan Begin	50 m/z	Set Capillary	4500 V	Set Dry Gas	4.0 l/min
Scan End	3000 m/z	Set End Plate Offset	-500 V	Set Divert Valve	Waste

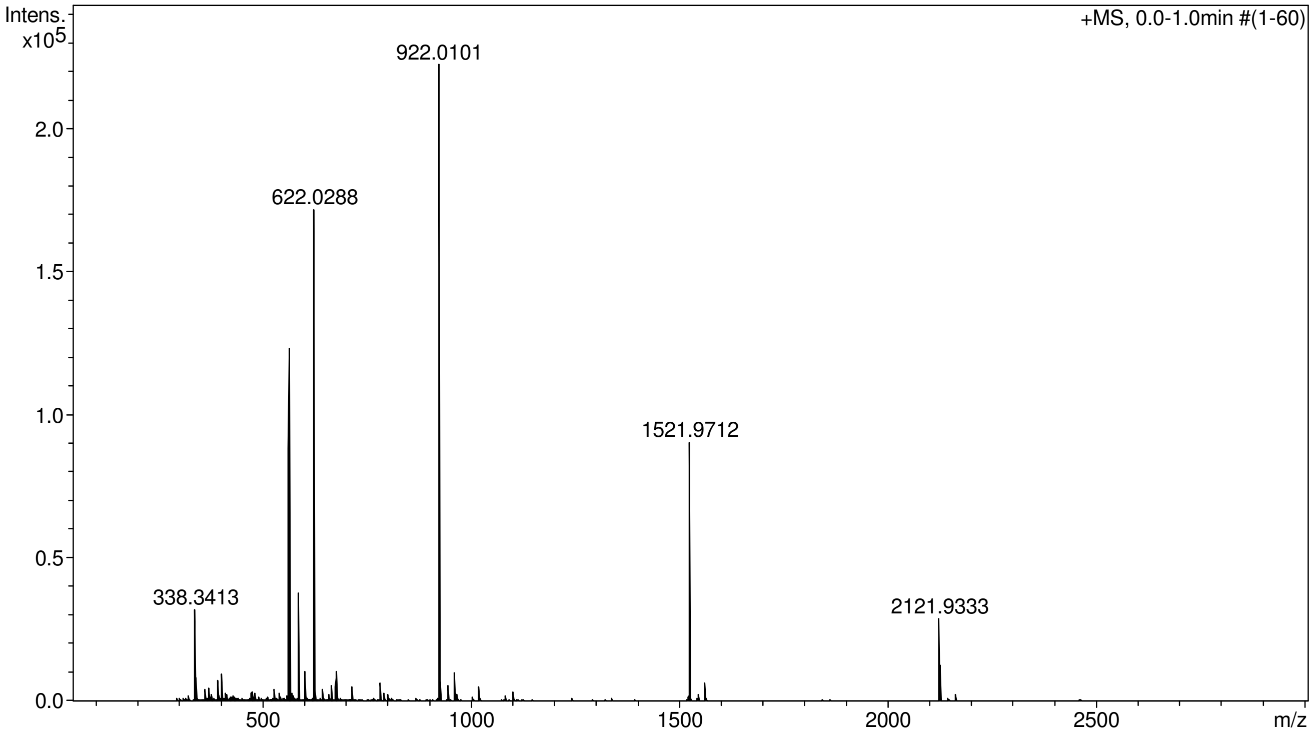

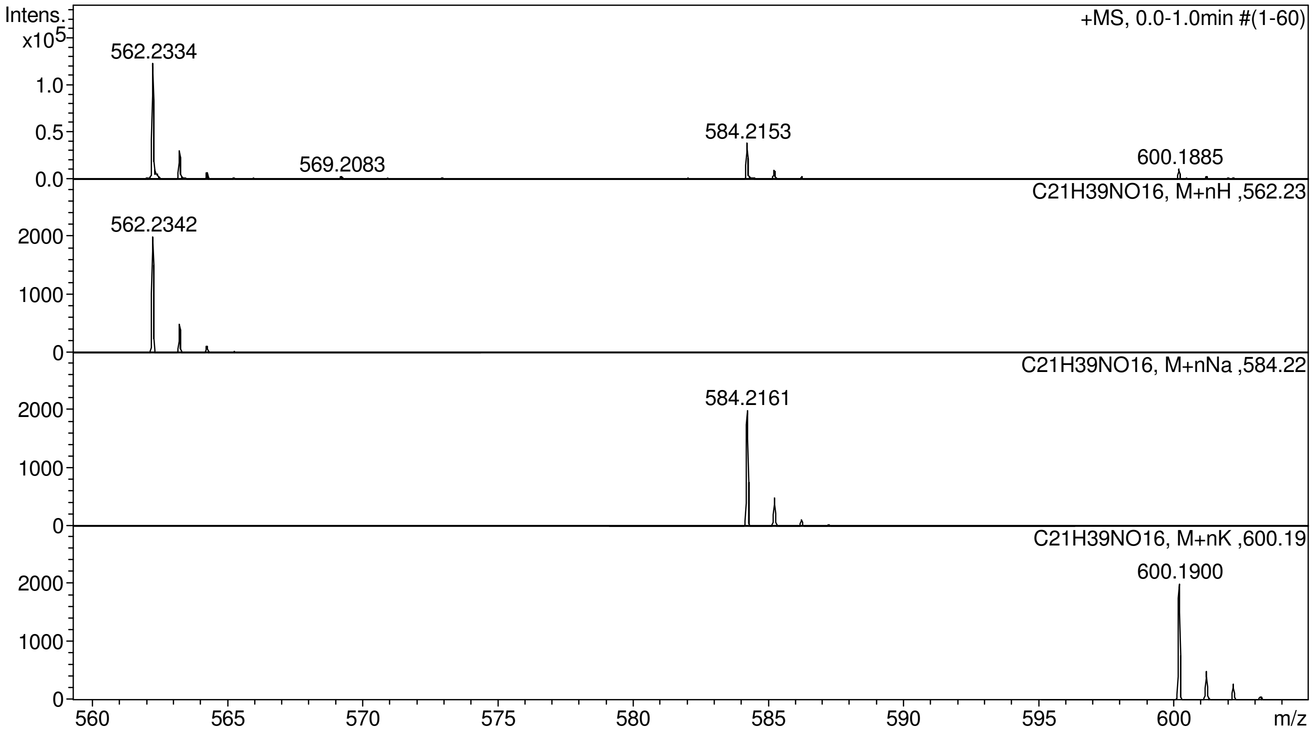

^{1}H-NMR of **2b** (600 MHz, D_2O, 303K)

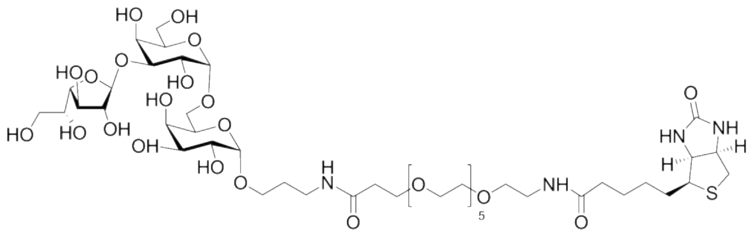

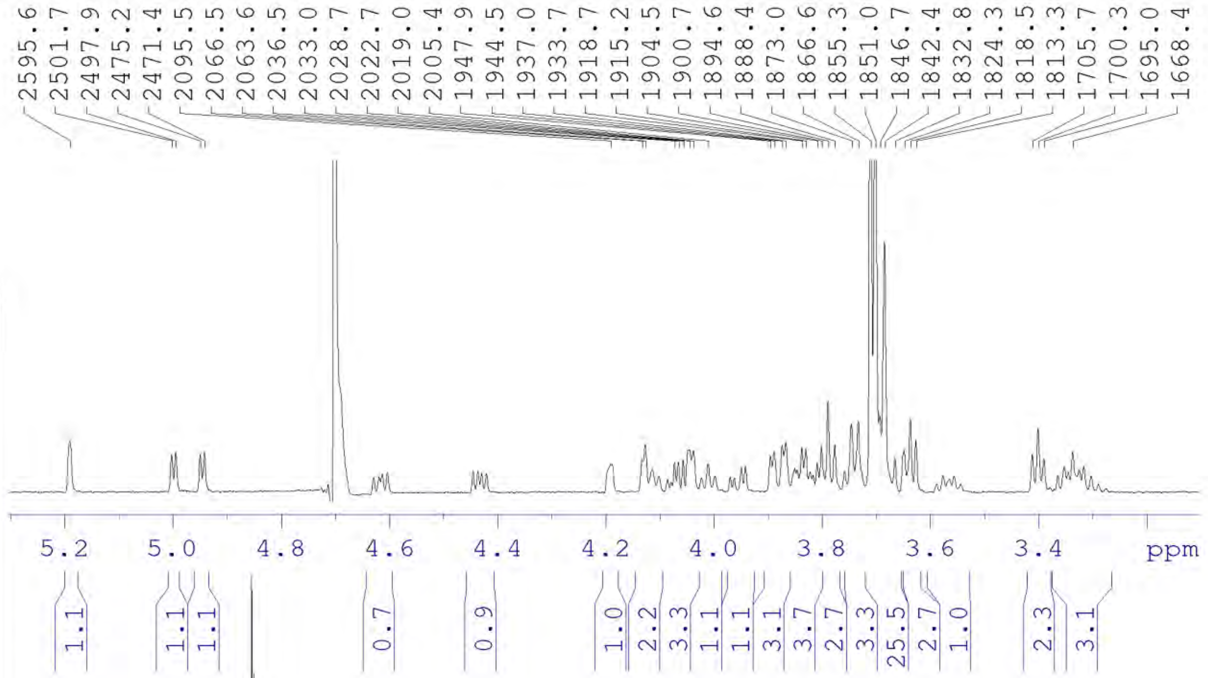

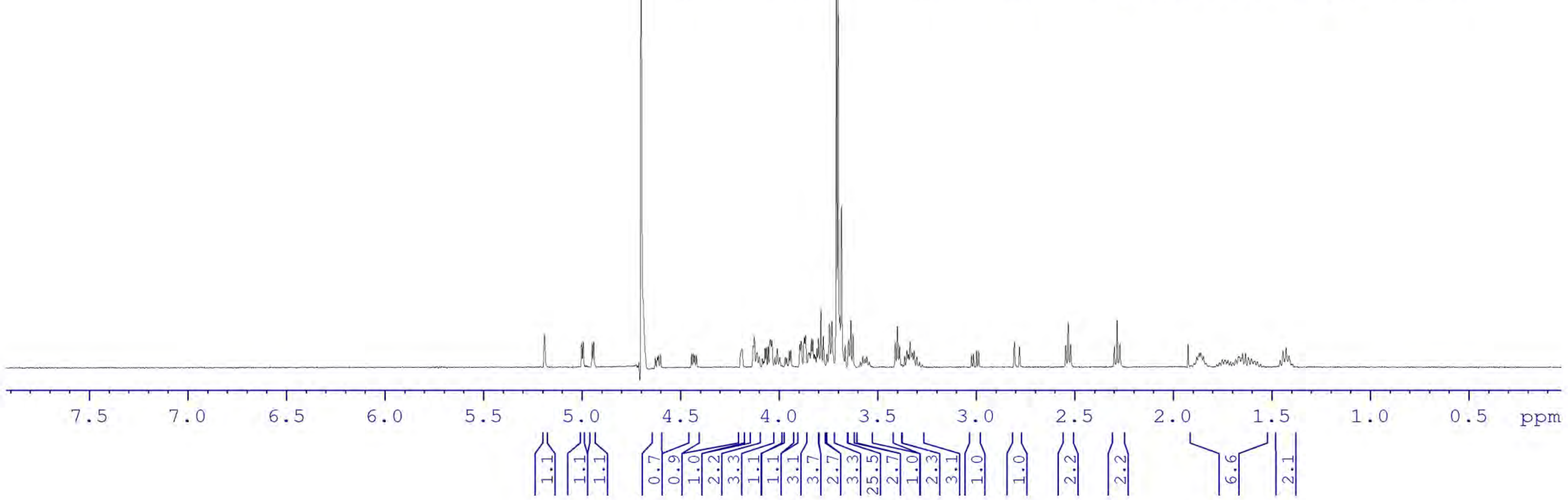

HRMS for **2b**

Comment	CH3CN : H2O 50/50 %, dil. 200, calibrant added				

Acquisition Parameter

Source Type	ESI	Ion Polarity	Positive	Set Nebulizer	0.5 Bar
Focus	Active			Set Dry Heater	180 °C
Scan Begin	50 m/z	Set Capillary	4500 V	Set Dry Gas	4.0 l/min
Scan End	3000 m/z	Set End Plate Offset	-500 V	Set Divert Valve	Waste

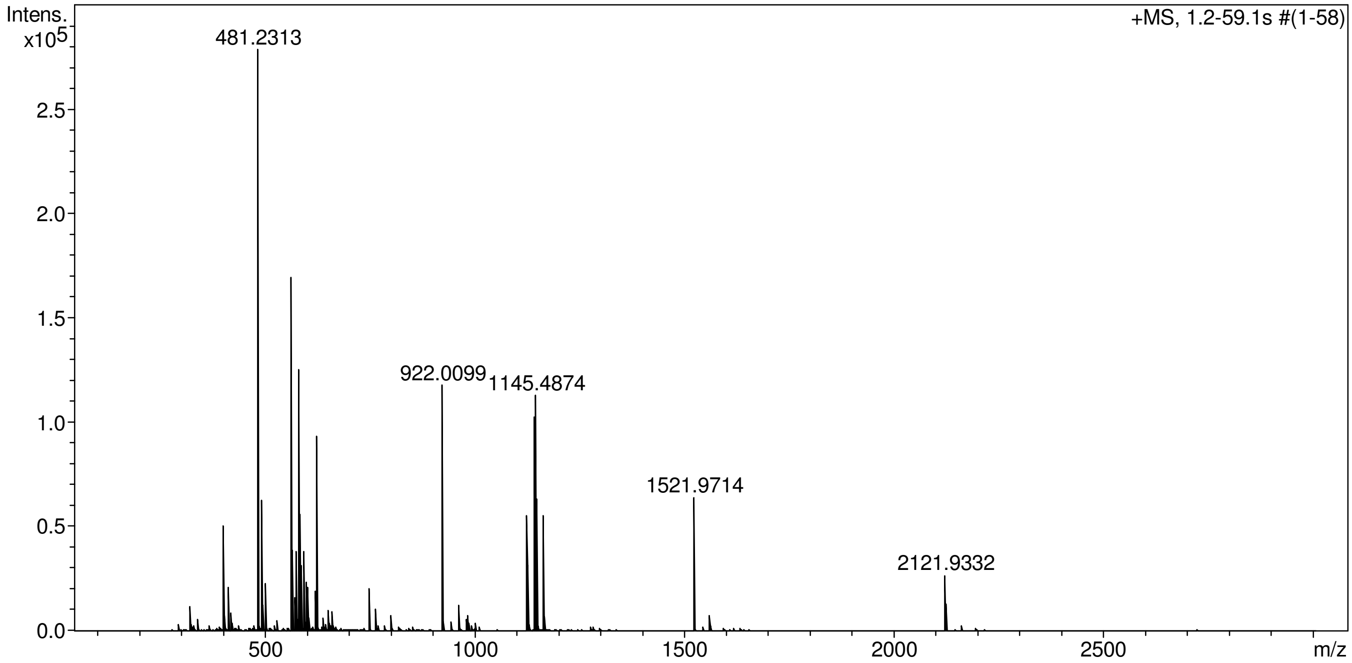

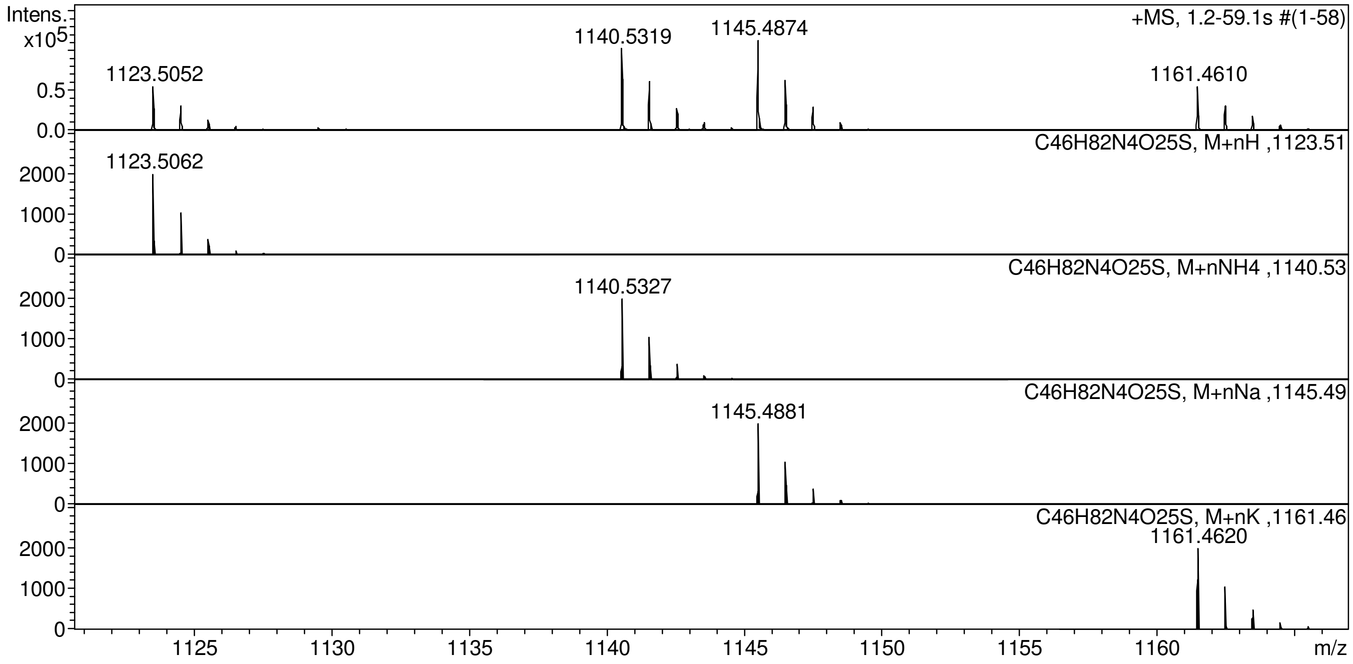

^{1}H-NMR of **S5** (600 MHz, $CDCl_3$)

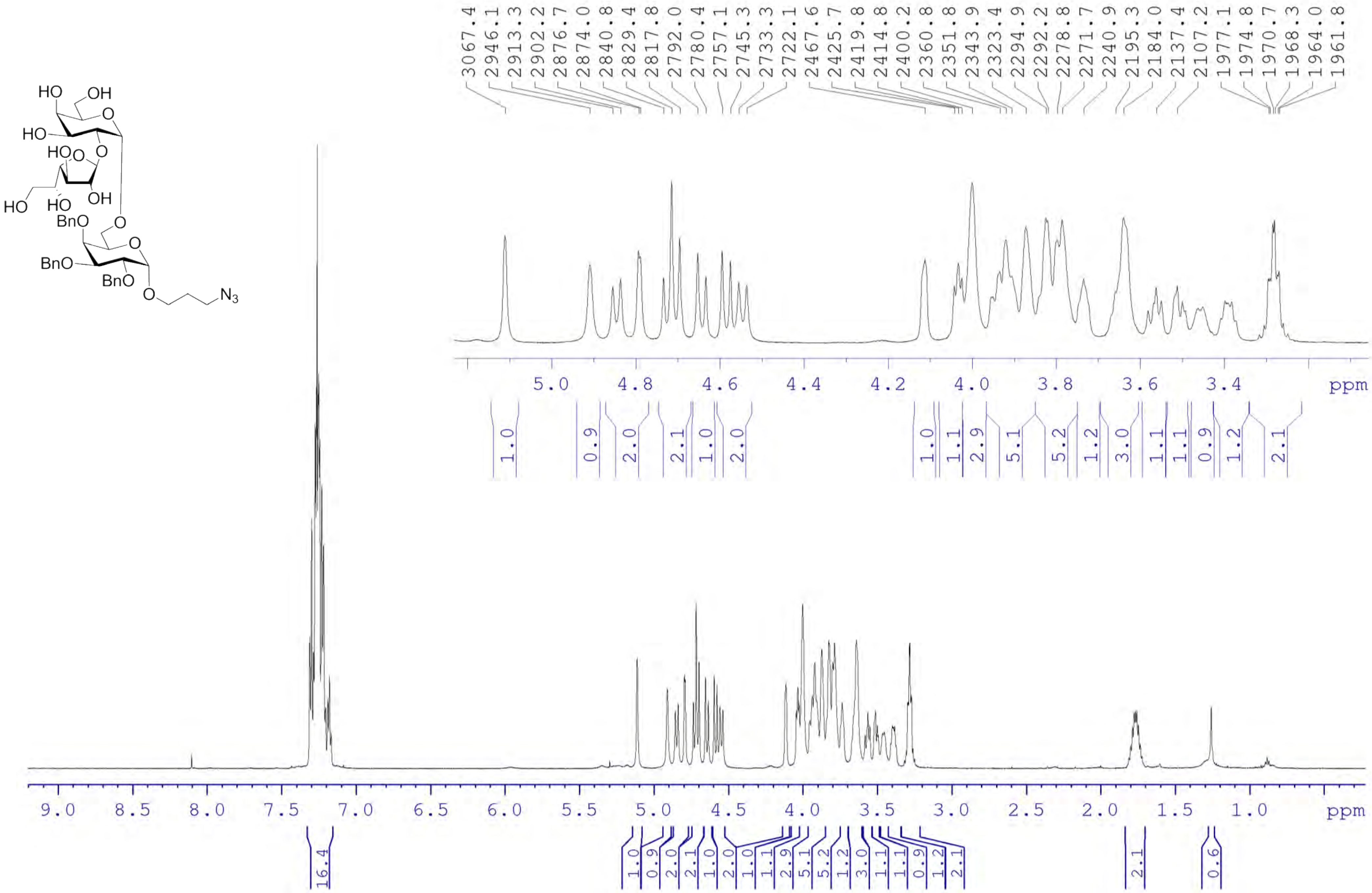

^{13}C-NMR of **S5** (150 MHz, $CDCl_3$)

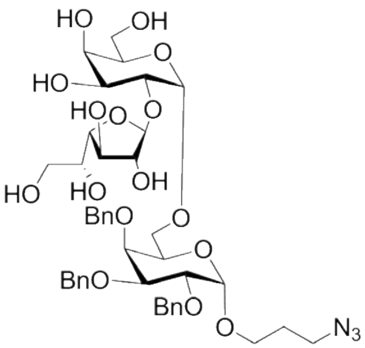

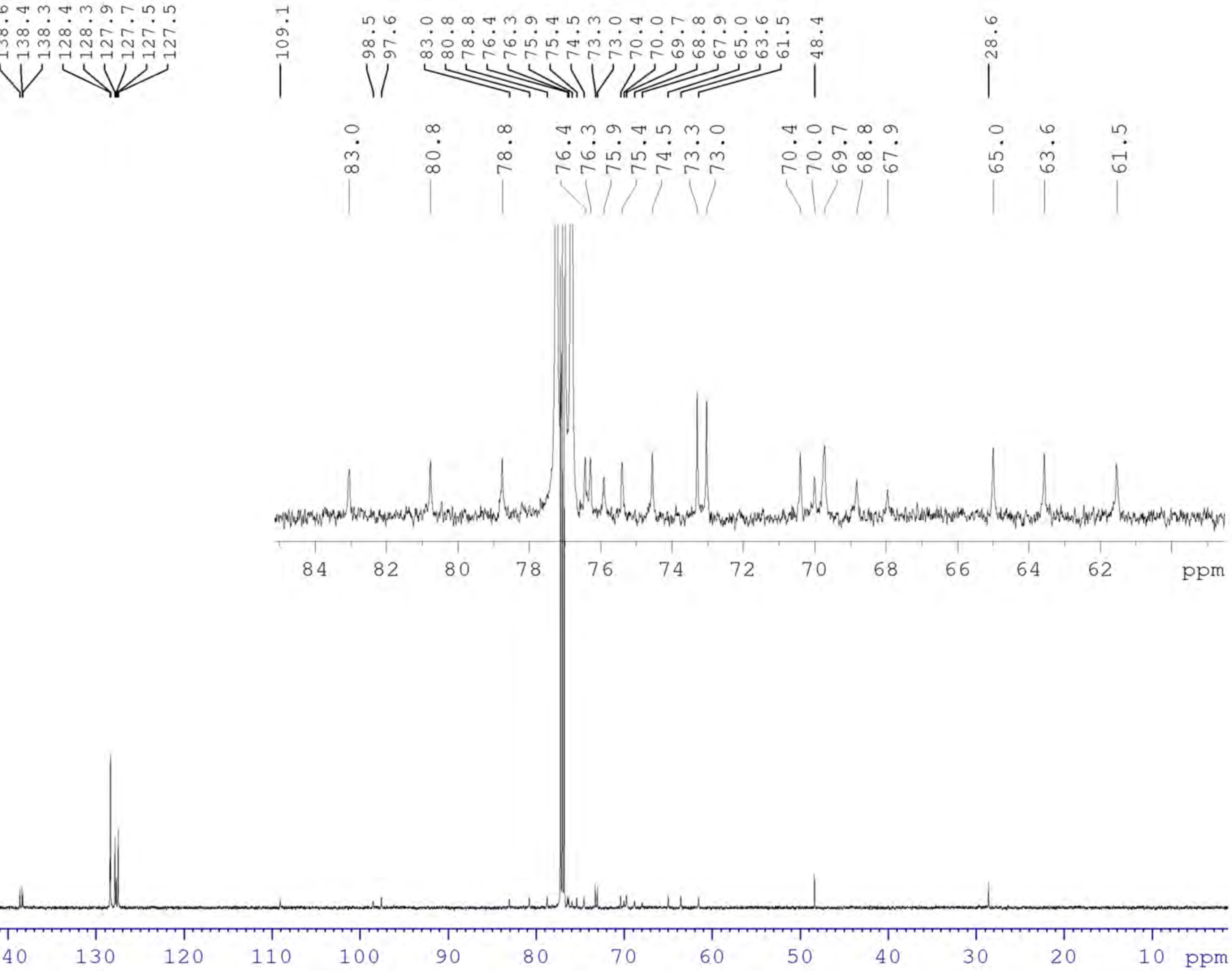

^{1}H-^{1}H COSY of **S5** (600 MHz, $CDCl_3$)

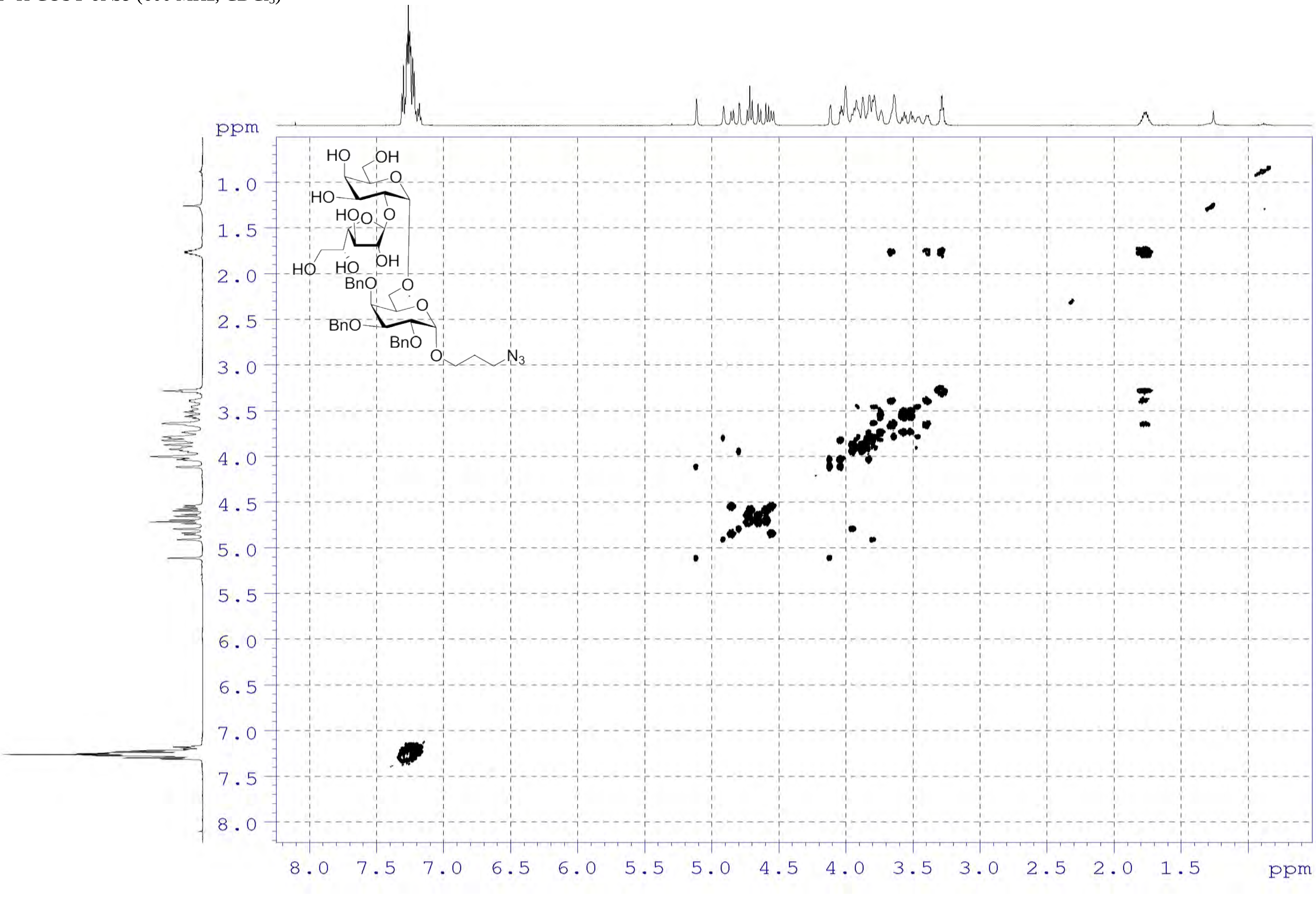

^{1}H-^{13}C HSQC of **S5** (600 MHz, $CDCl_3$)

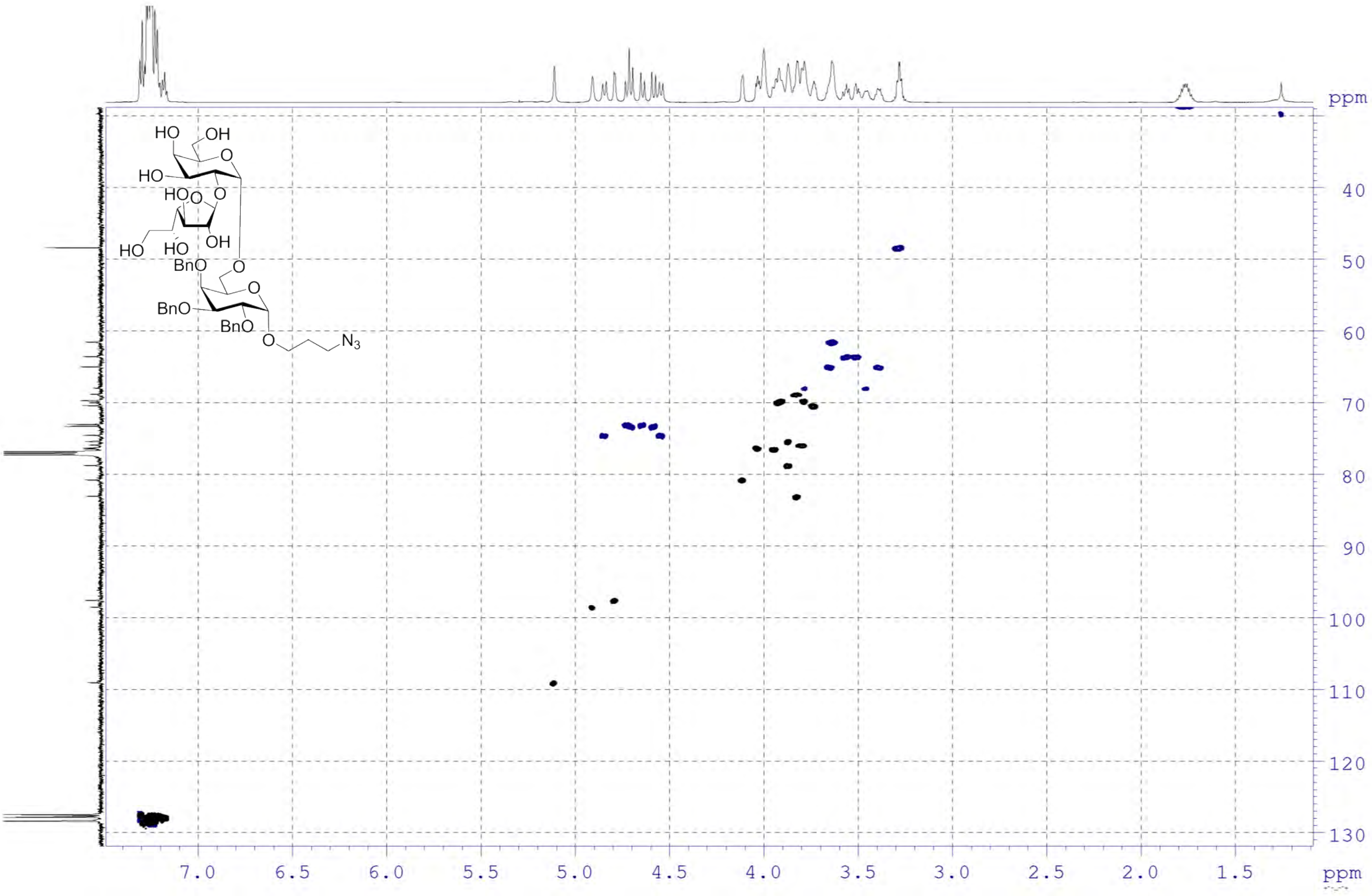

^{1}H-NMR of **1a** (600 MHz, D_2O, 303K)

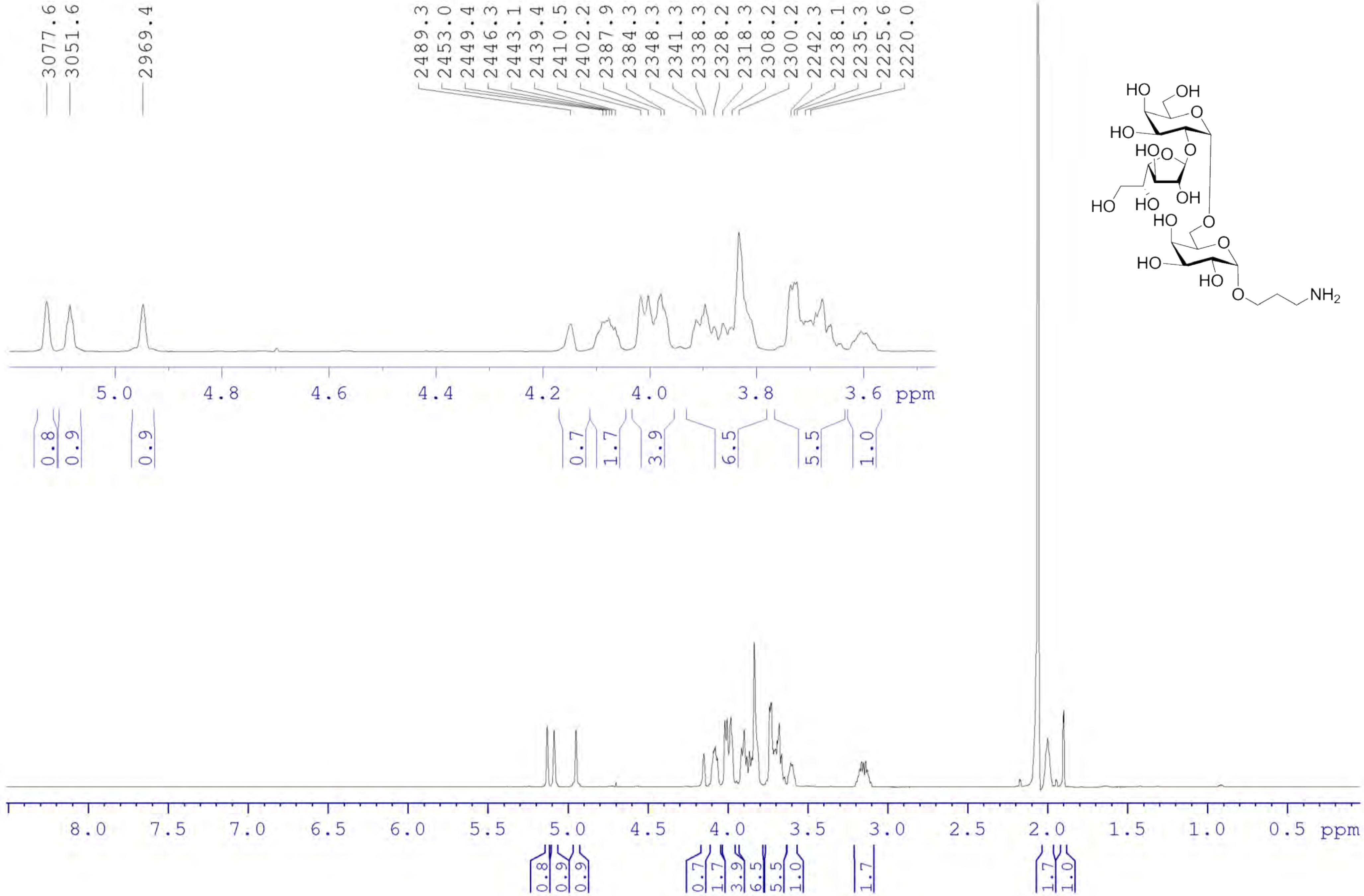

^{13}C-NMR of **1a** (150 MHz, D_2O, 303K)

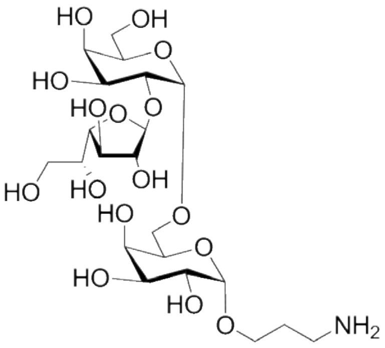

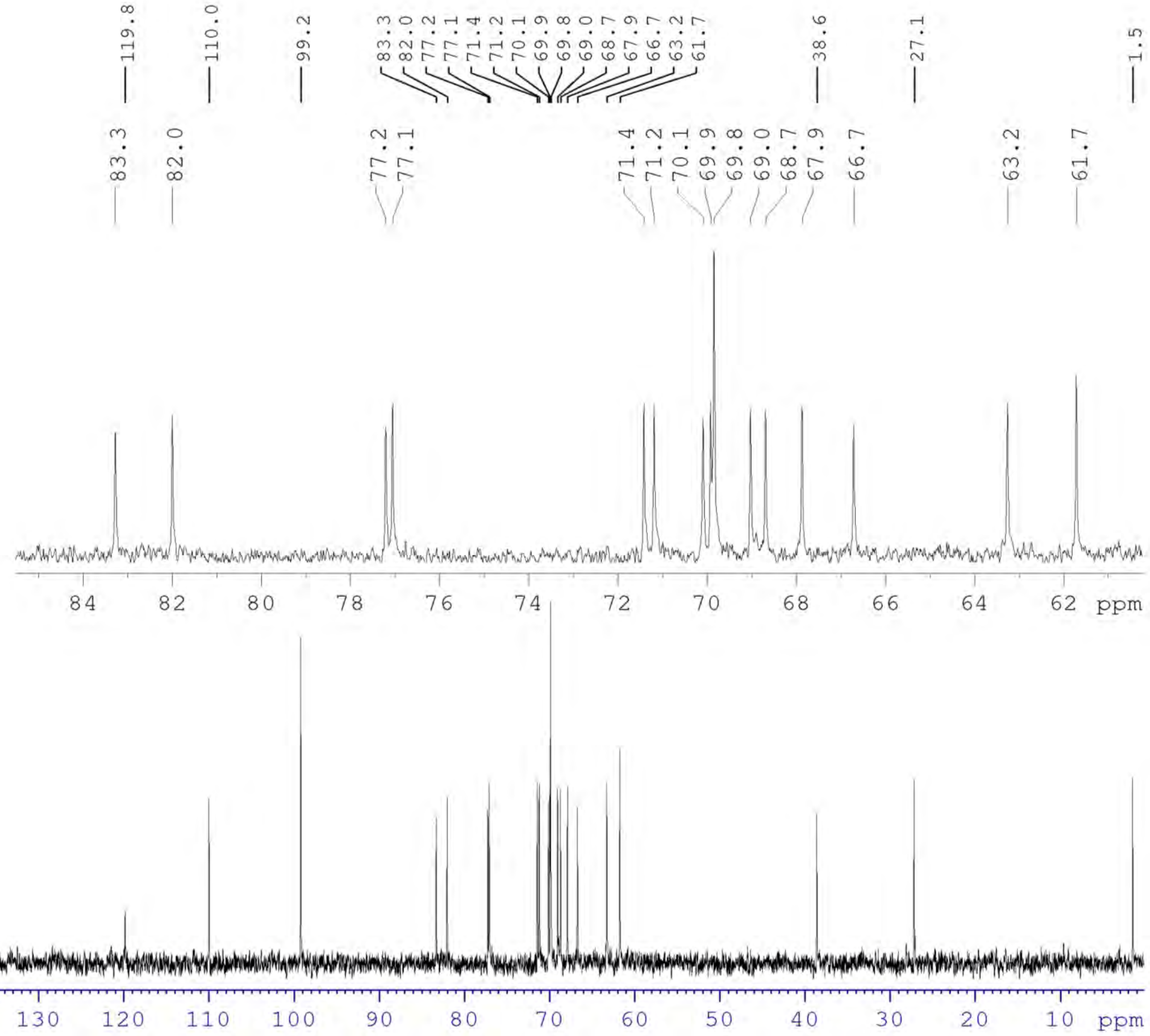

^{1}H-^{1}H COSY of **1a** (600 MHz, D_2O, 303K)

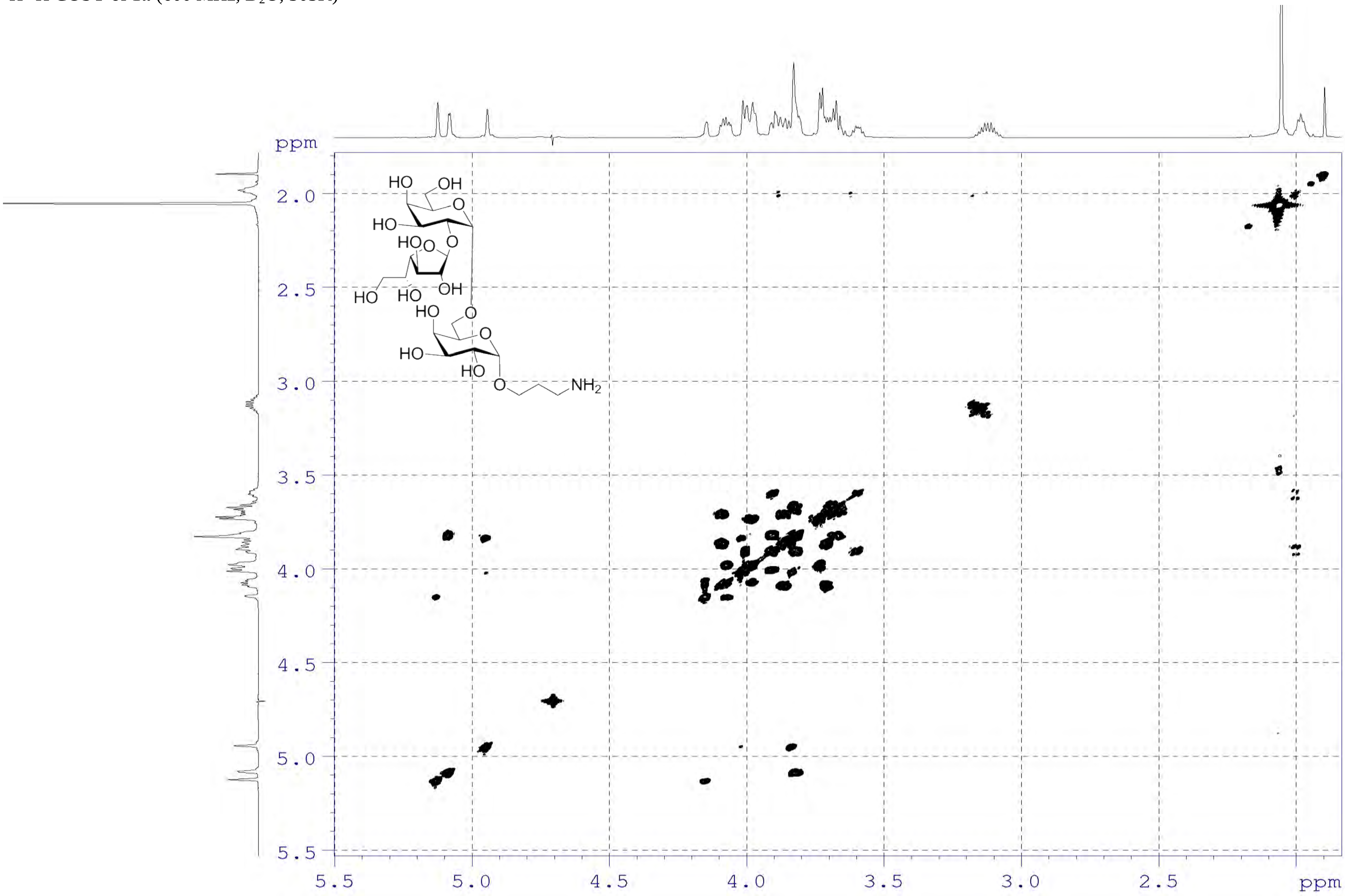

^{1}H-^{13}C HSQC of **1a** (600 MHz, D_2O, 303K)

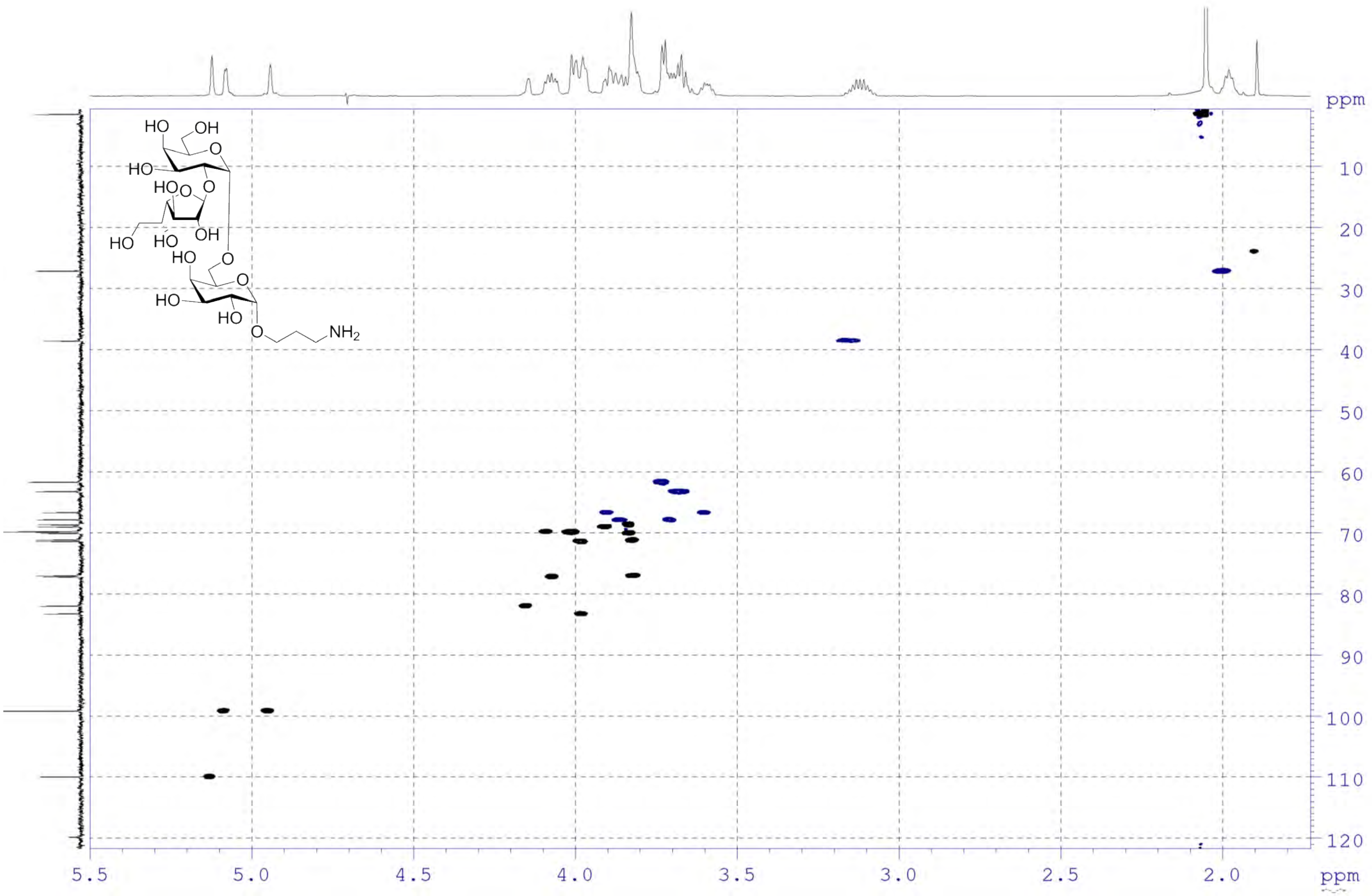

HRMS for **1a**

Comment	CH3CN : H2O 50/50 %, dil. 2000, calibrant added

Acquisition Parameter

Source Type	ESI	Ion Polarity	Positive	Set Nebulizer	0.4 Bar
Focus	Not active			Set Dry Heater	180 °C
Scan Begin	50 m/z	Set Capillary	4500 V	Set Dry Gas	4.0 l/min
Scan End	3000 m/z	Set End Plate Offset	-500 V	Set Divert Valve	Waste

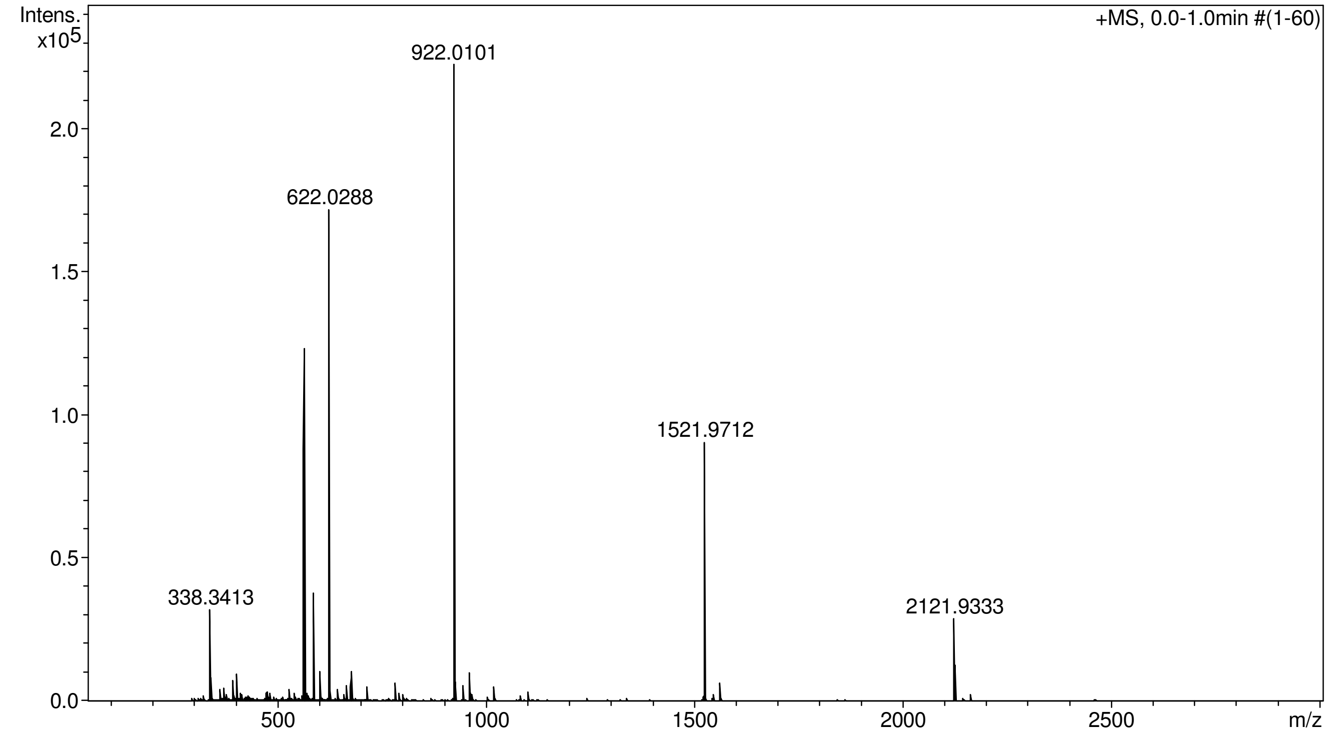

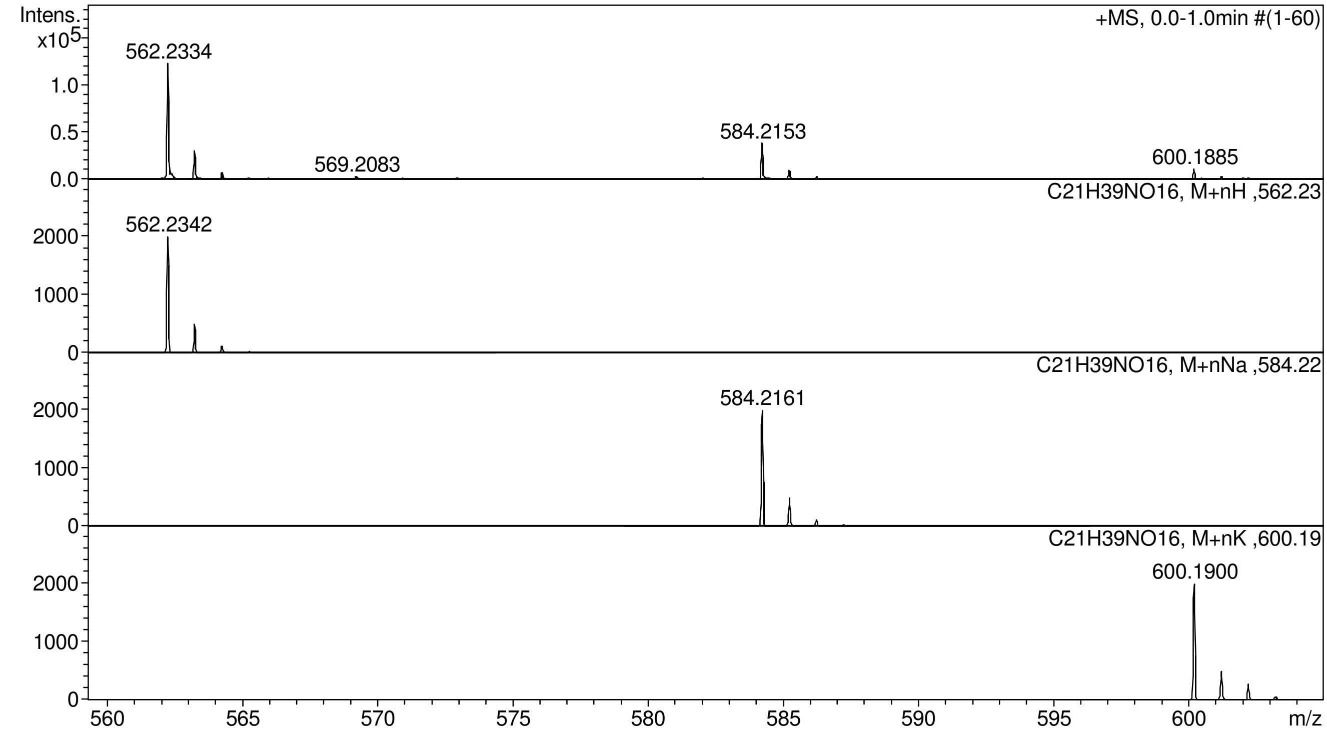

^{1}H-NMR of **1b** (600 MHz, D_2O, 303K)

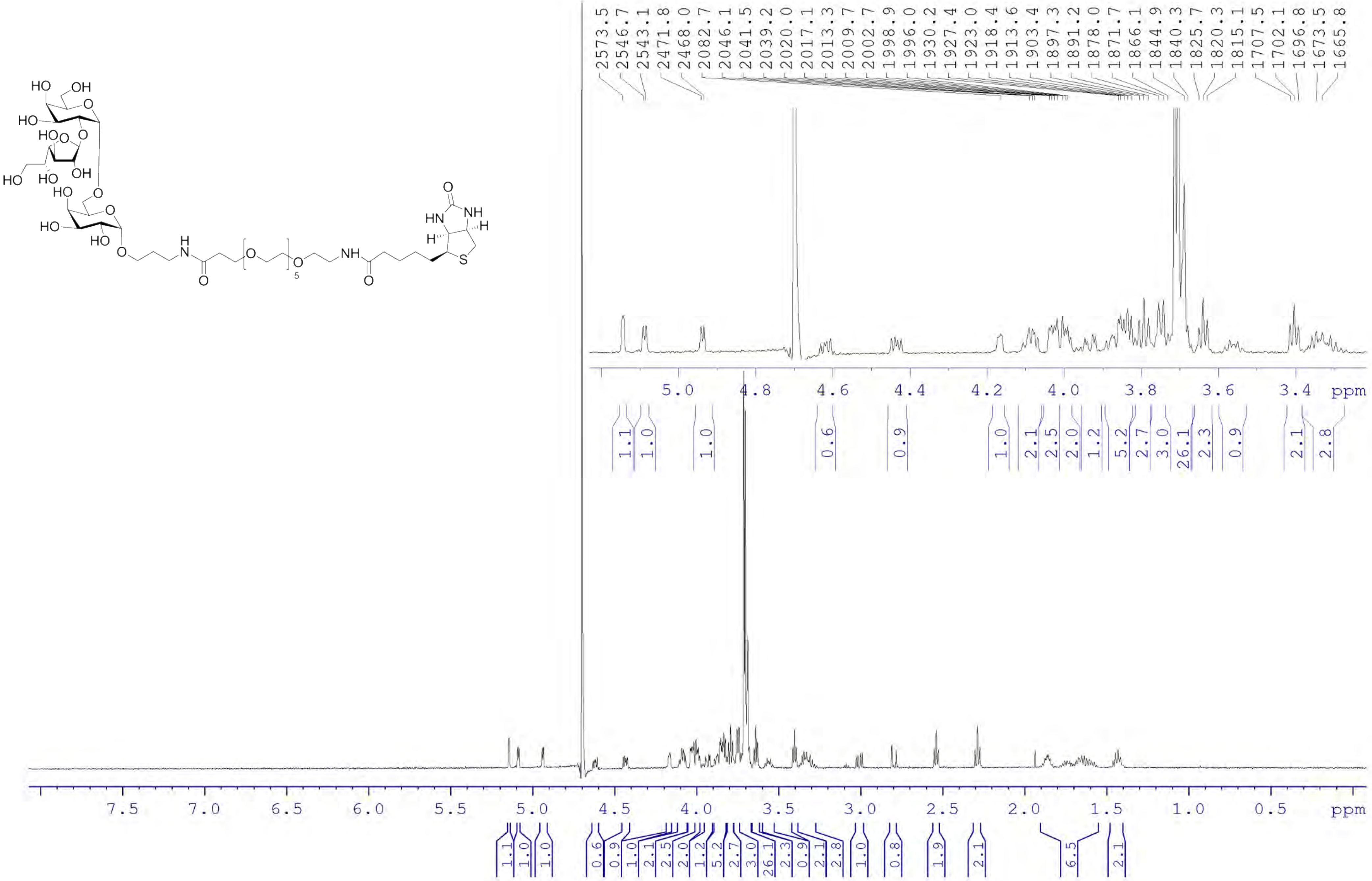

HRMS for **1b**

Comment	CH3CN : H2O 50/50 %, dil. 200, calibrant added				

Acquisition Parameter

Source Type	ESI	Ion Polarity	Positive	Set Nebulizer	0.5 Bar
Focus	Active			Set Dry Heater	180 °C
Scan Begin	50 m/z	Set Capillary	4500 V	Set Dry Gas	4.0 l/min
Scan End	3000 m/z	Set End Plate Offset	-500 V	Set Divert Valve	Waste

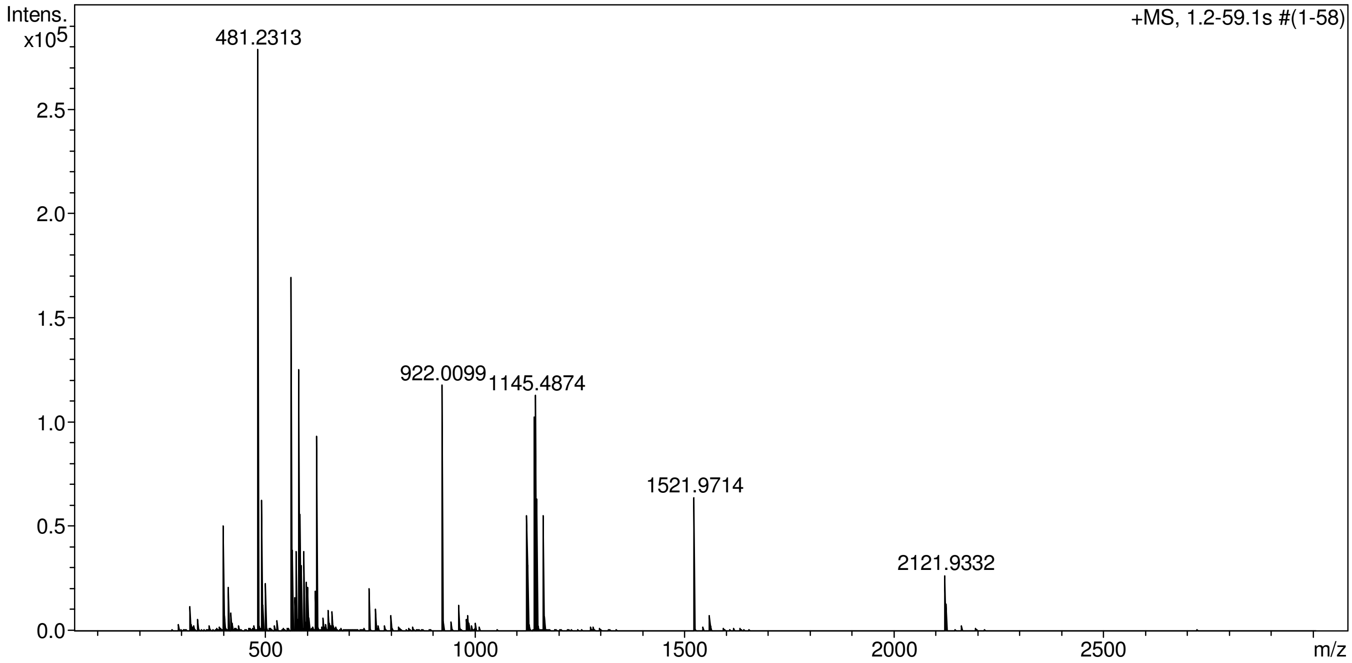

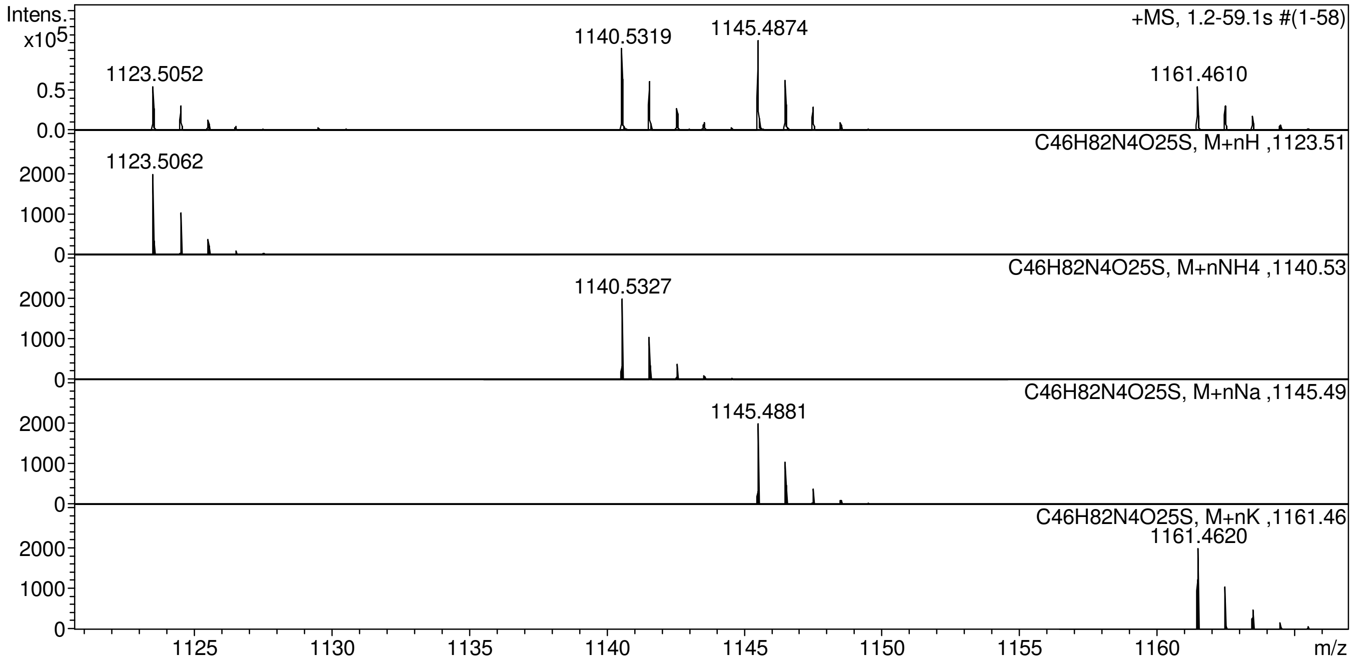

^{1}H-NMR of **S6** (600 MHz, $CD_3OD + CDCl_3$, 303K)

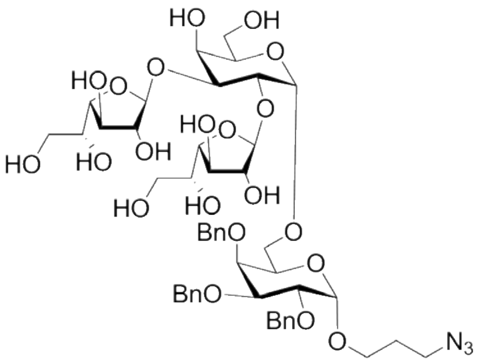

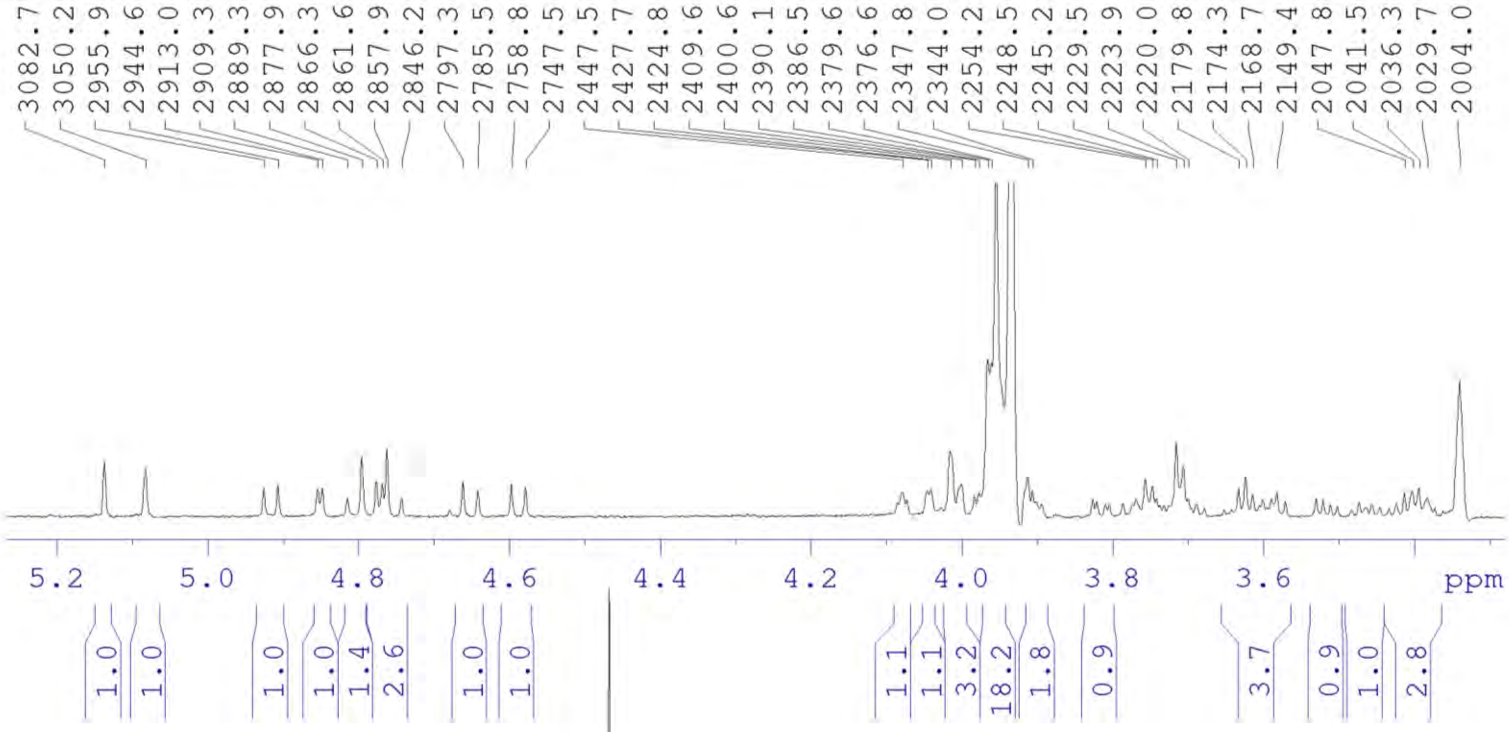

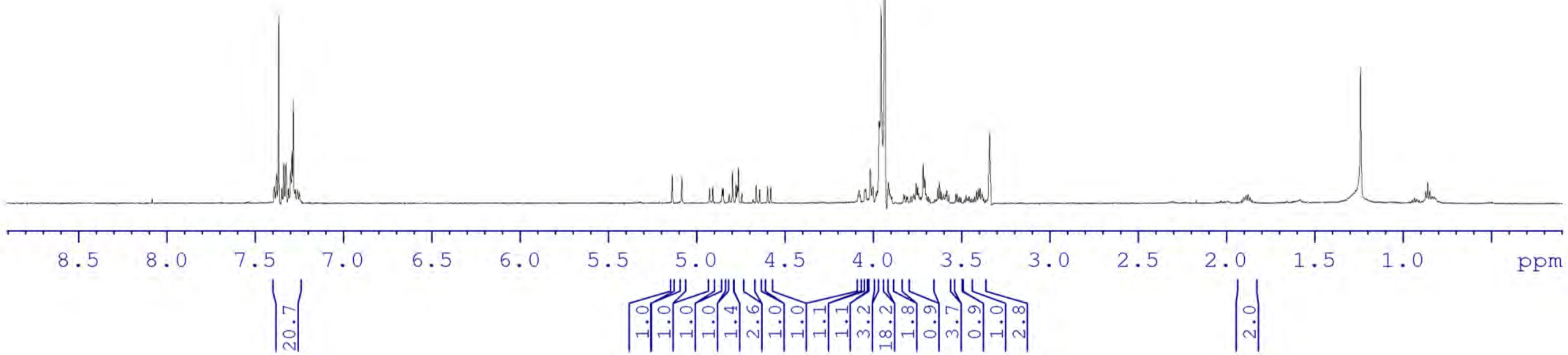

^{13}C-NMR of **S6** (150 MHz, CD_3OD + $CDCl_3$, 303K)

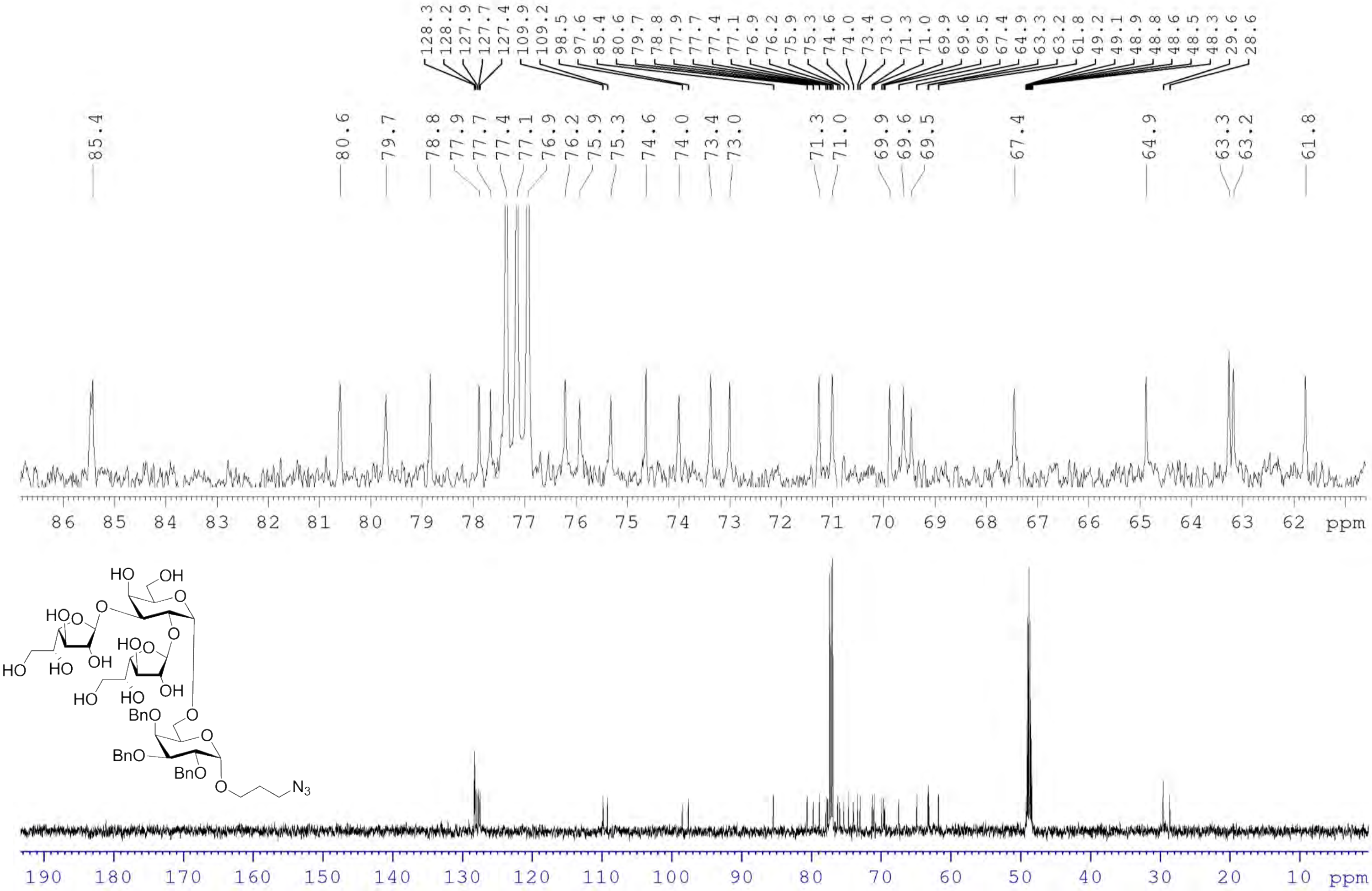

^{1}H-^{1}H COSY of **S6** (600 MHz, CD_3OD + $CDCl_3$, 303K)

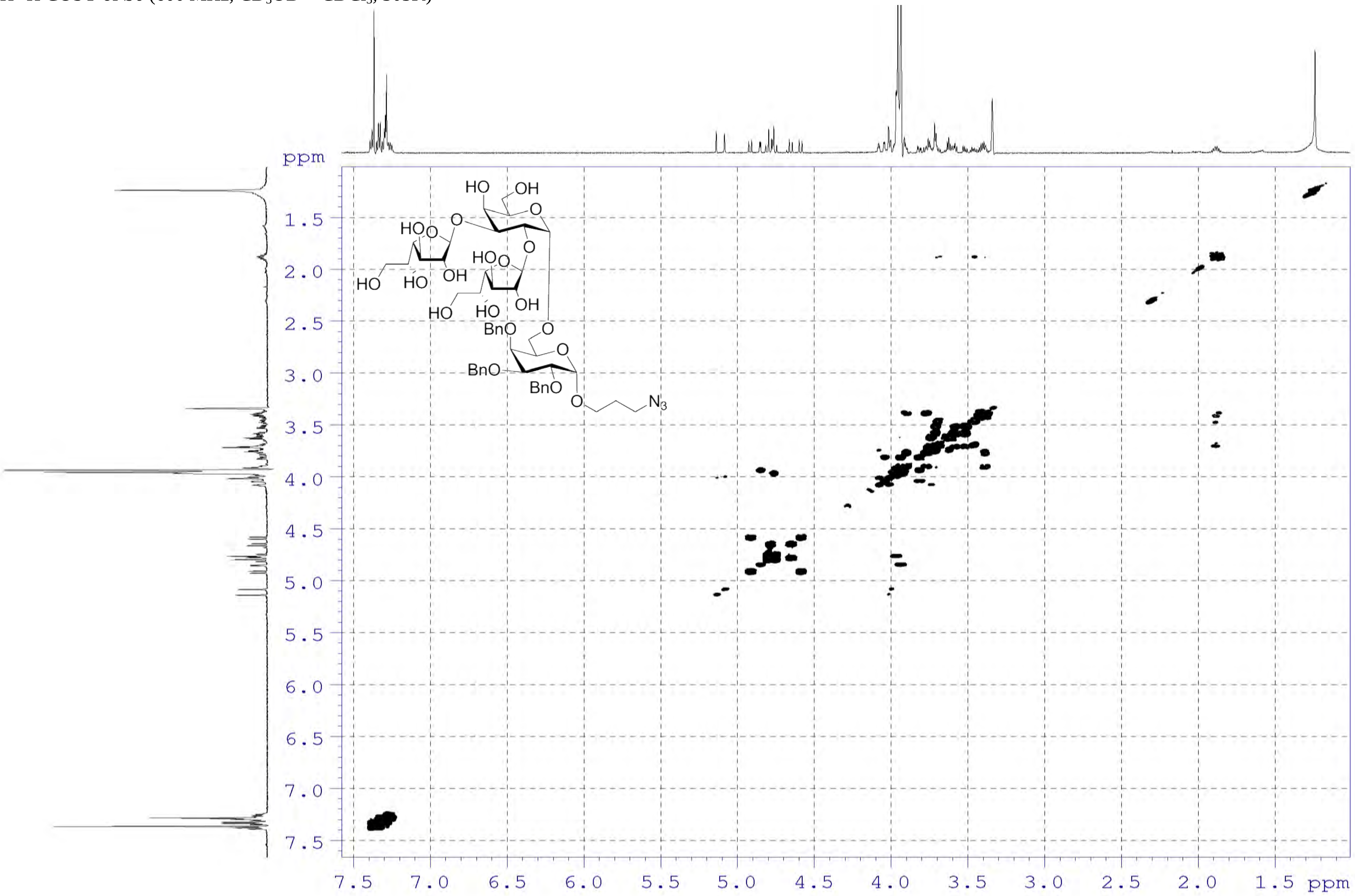

^{1}H-^{13}C HSQC of **S6** (600 MHz, $CD_3OD + CDCl_3$, 303K)

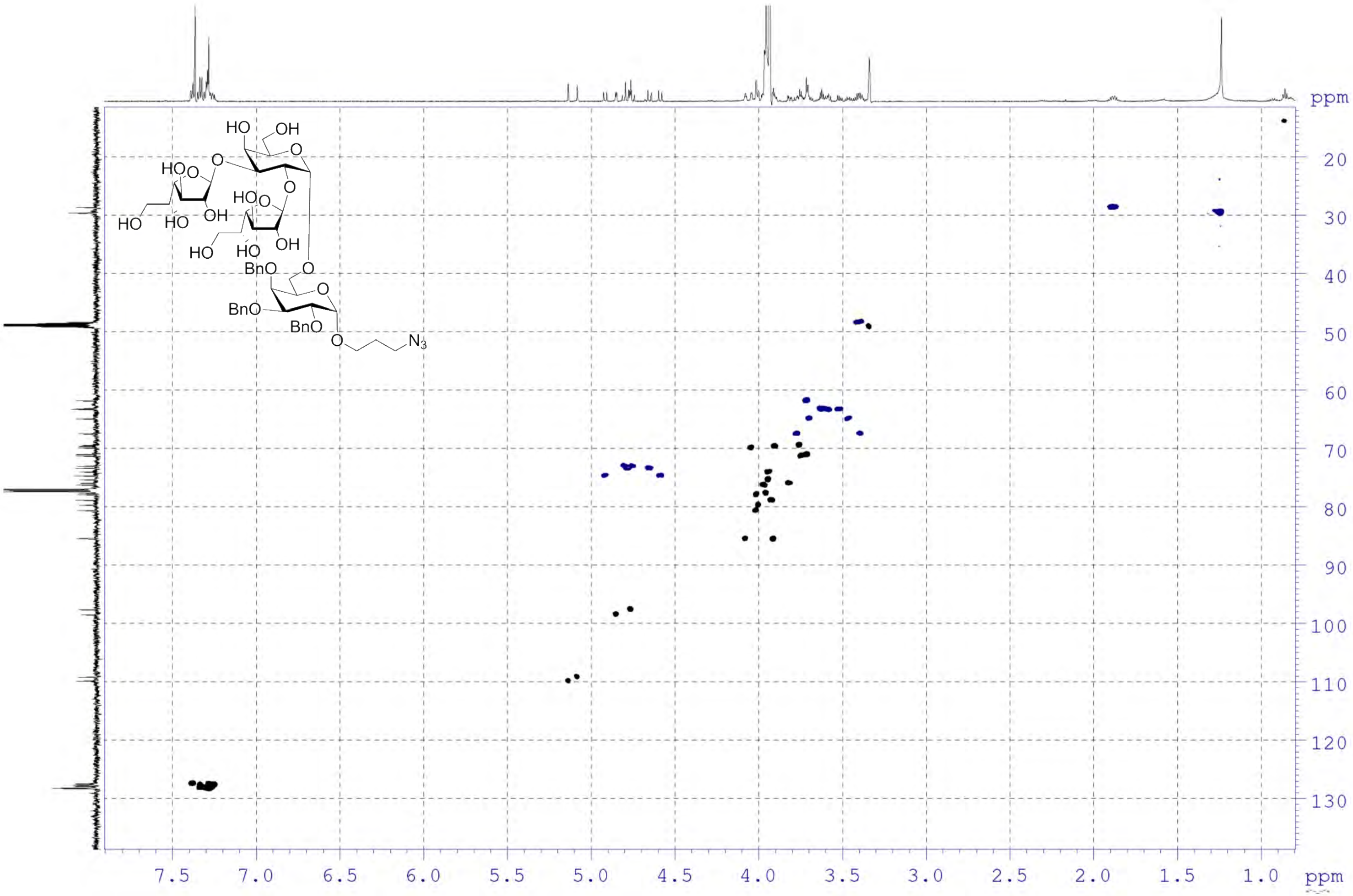

^{1}H-NMR of **3a** (600 MHz, D_2O, 303K)

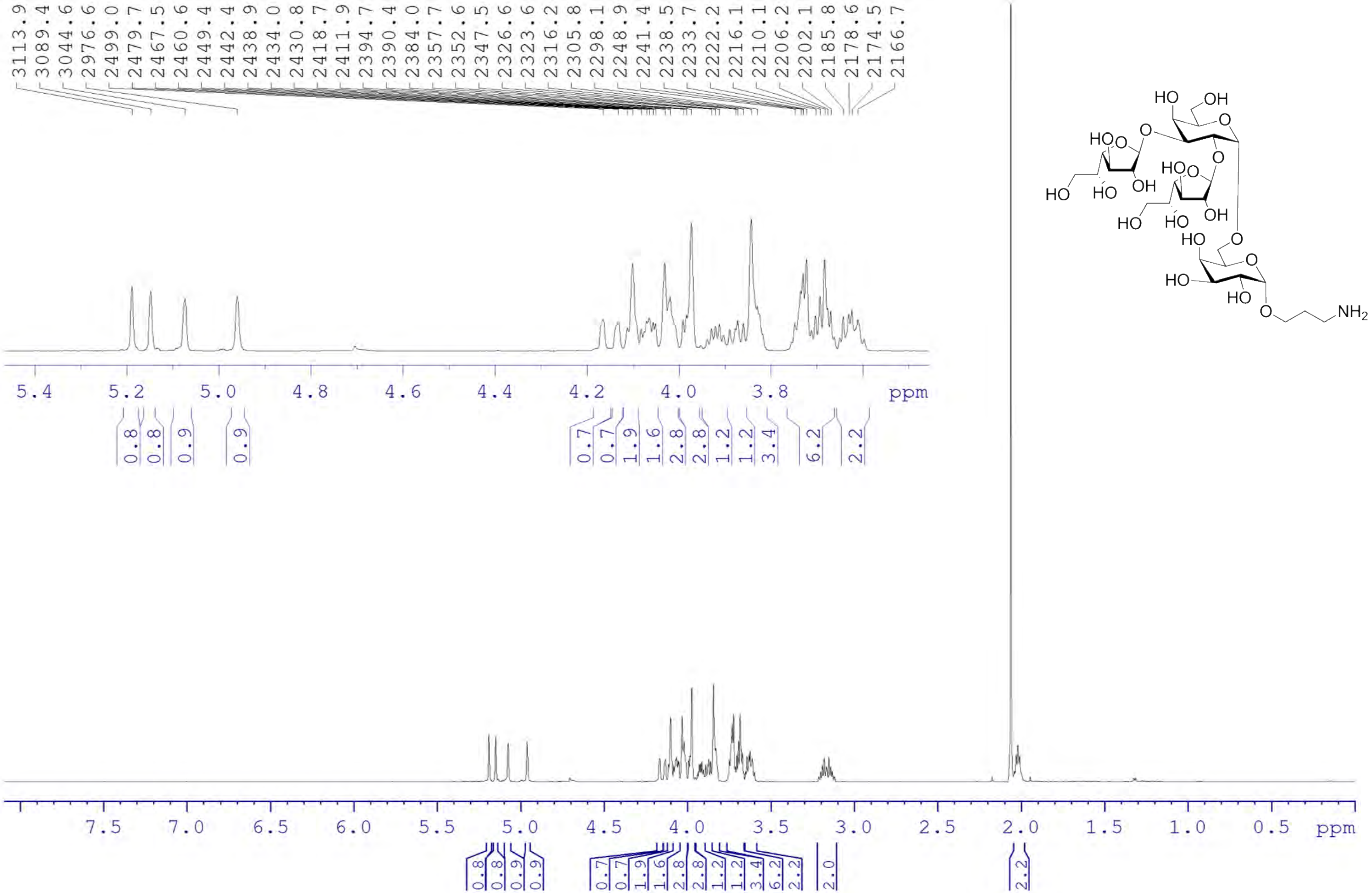

^{13}C-NMR of **3a** (150 MHz, D_2O, 303K)

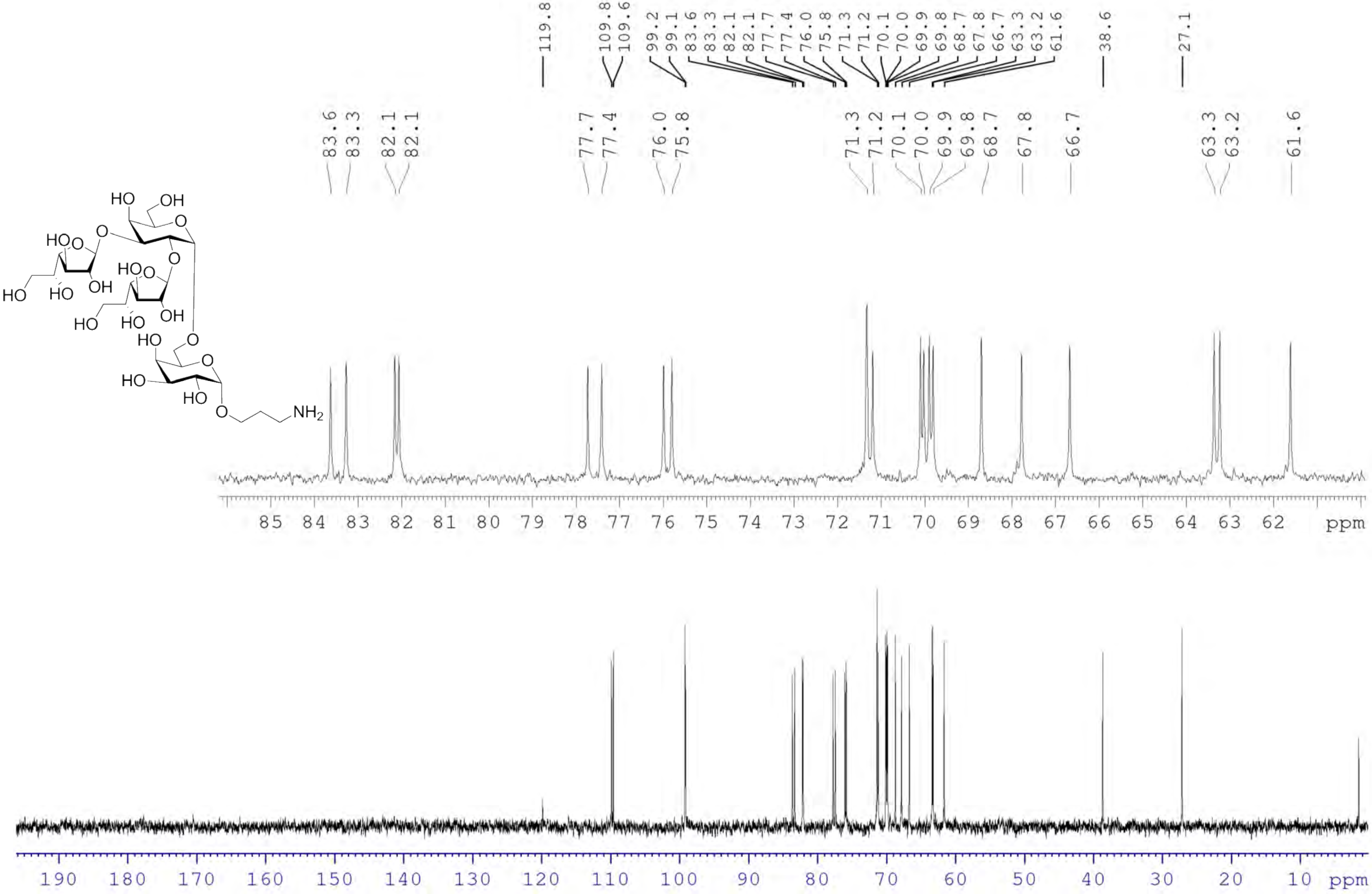

^{1}H-^{1}H COSY of **3a** (600 MHz, D_2O, 303K)

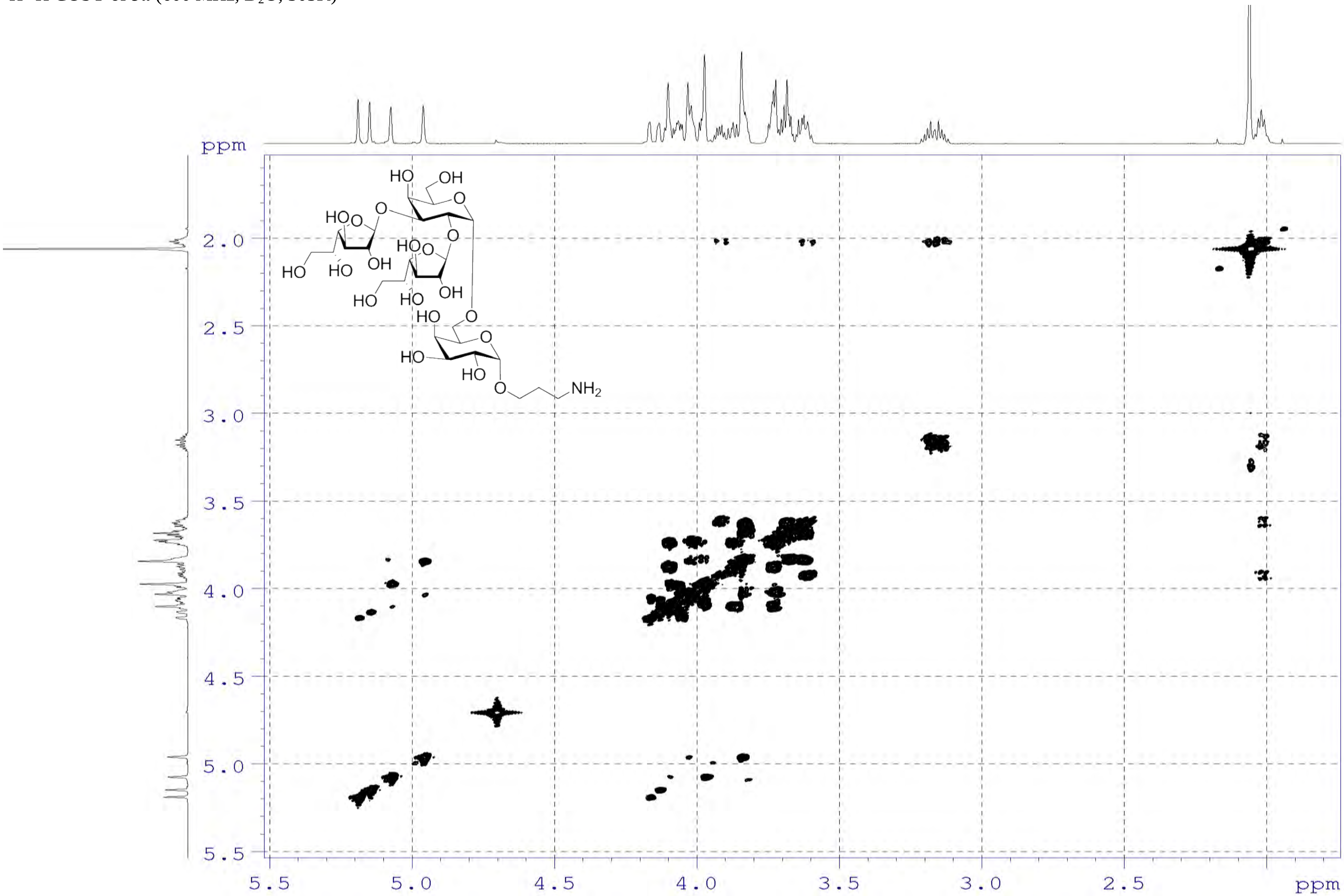

^{1}H-^{13}C HSQC of **3a** (600 MHz, D_2O, 303K)

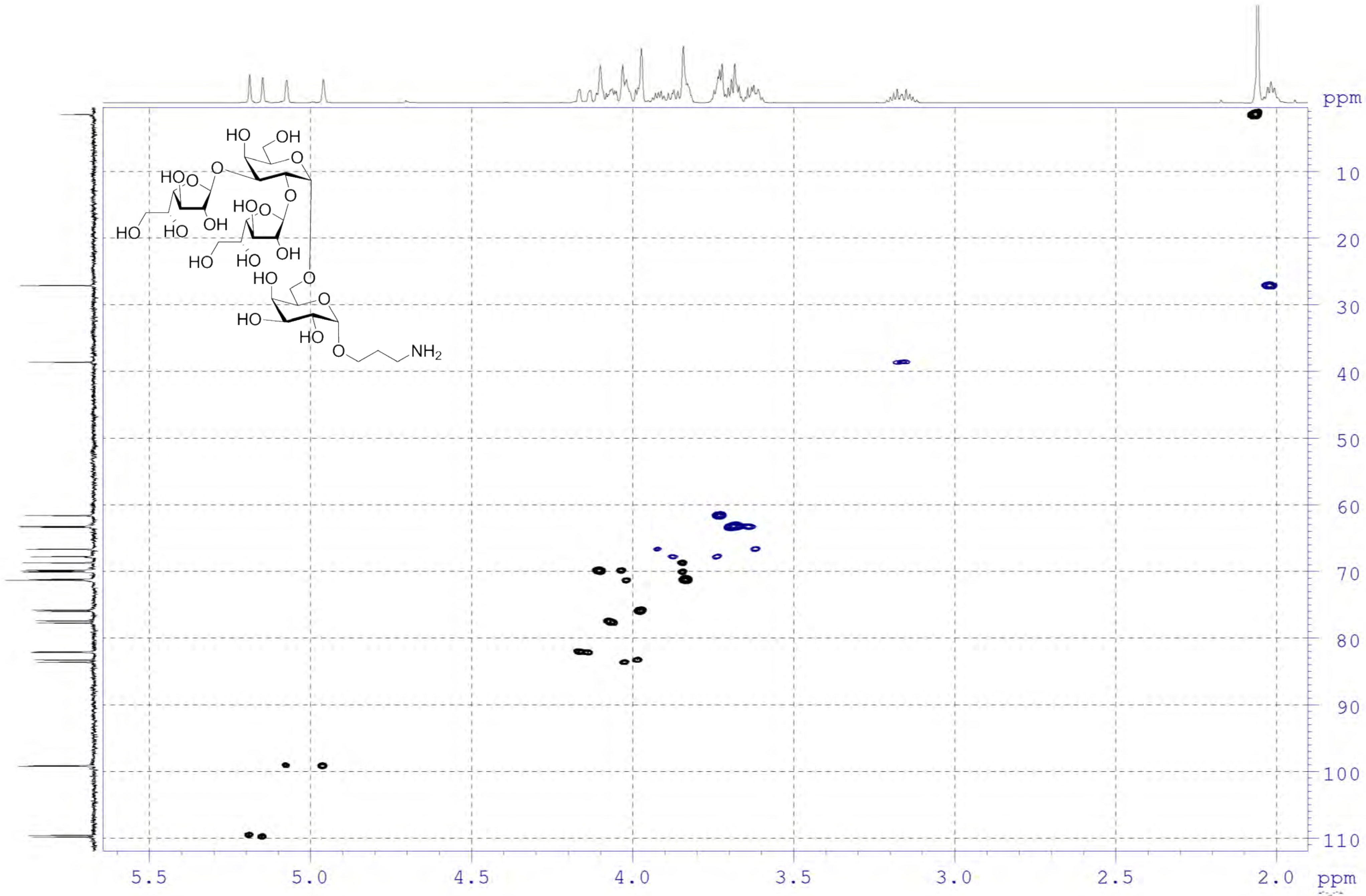

HRMS for **3a**

Comment	CH3CN : H2O 50/50 %, dil. 2000, calibrant added

Acquisition Parameter

Source Type	ESI	Ion Polarity	Positive	Set Nebulizer	0.4 Bar
Focus	Not active			Set Dry Heater	180 °C
Scan Begin	50 m/z	Set Capillary	4500 V	Set Dry Gas	4.0 l/min
Scan End	3000 m/z	Set End Plate Offset	-500 V	Set Divert Valve	Waste

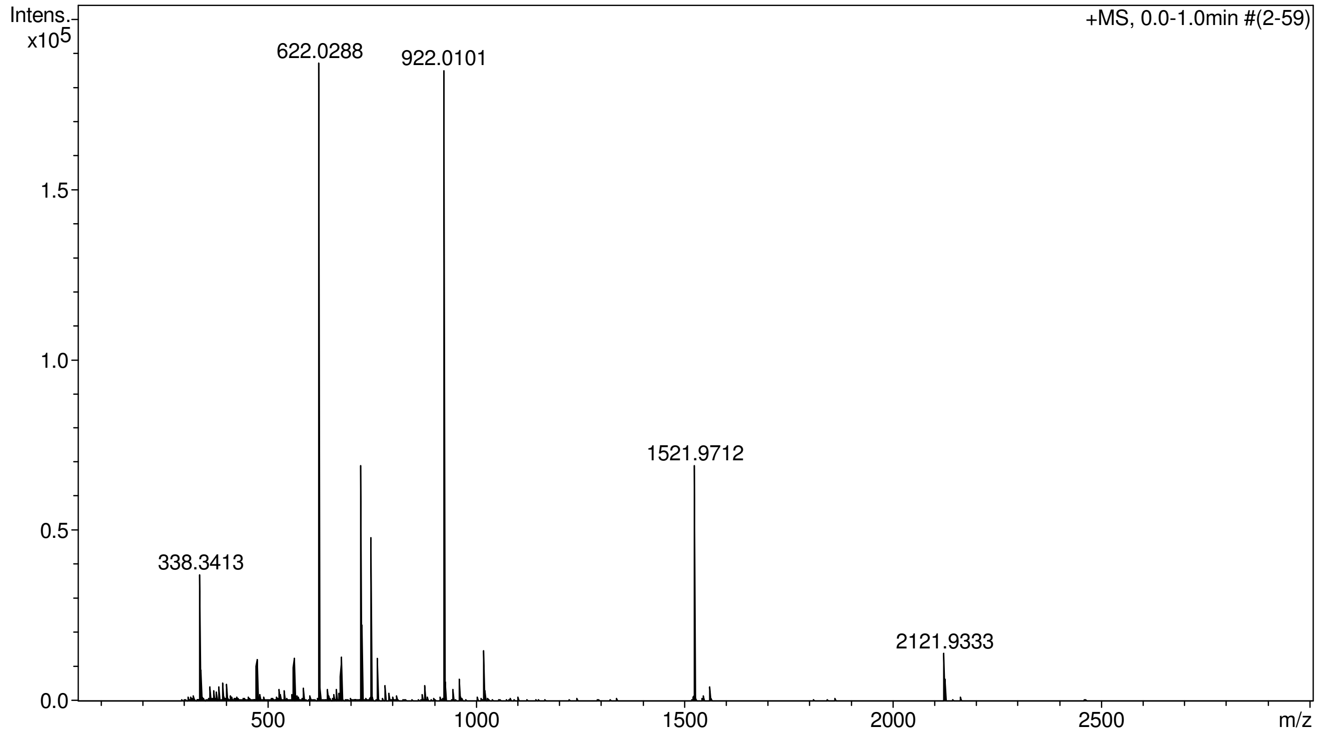

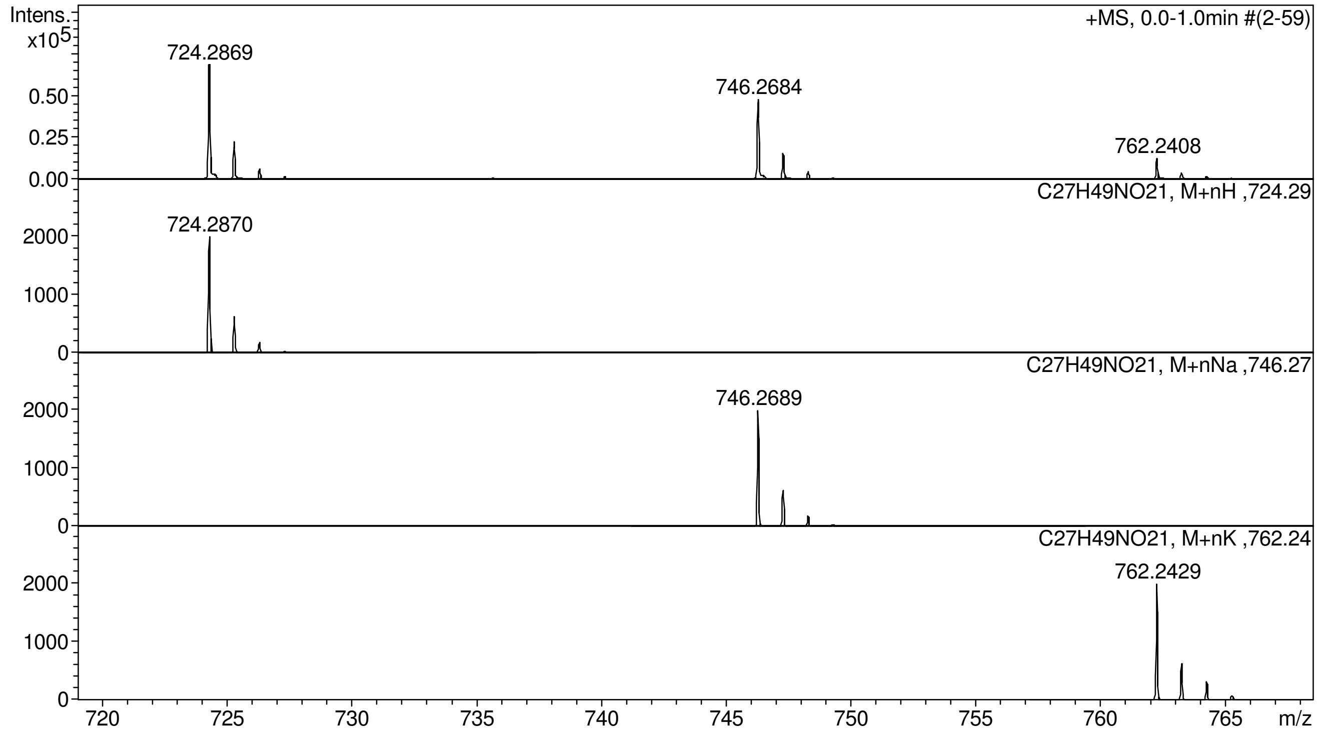

^{1}H-NMR of **3b** (600 MHz, D_2O, 303K)

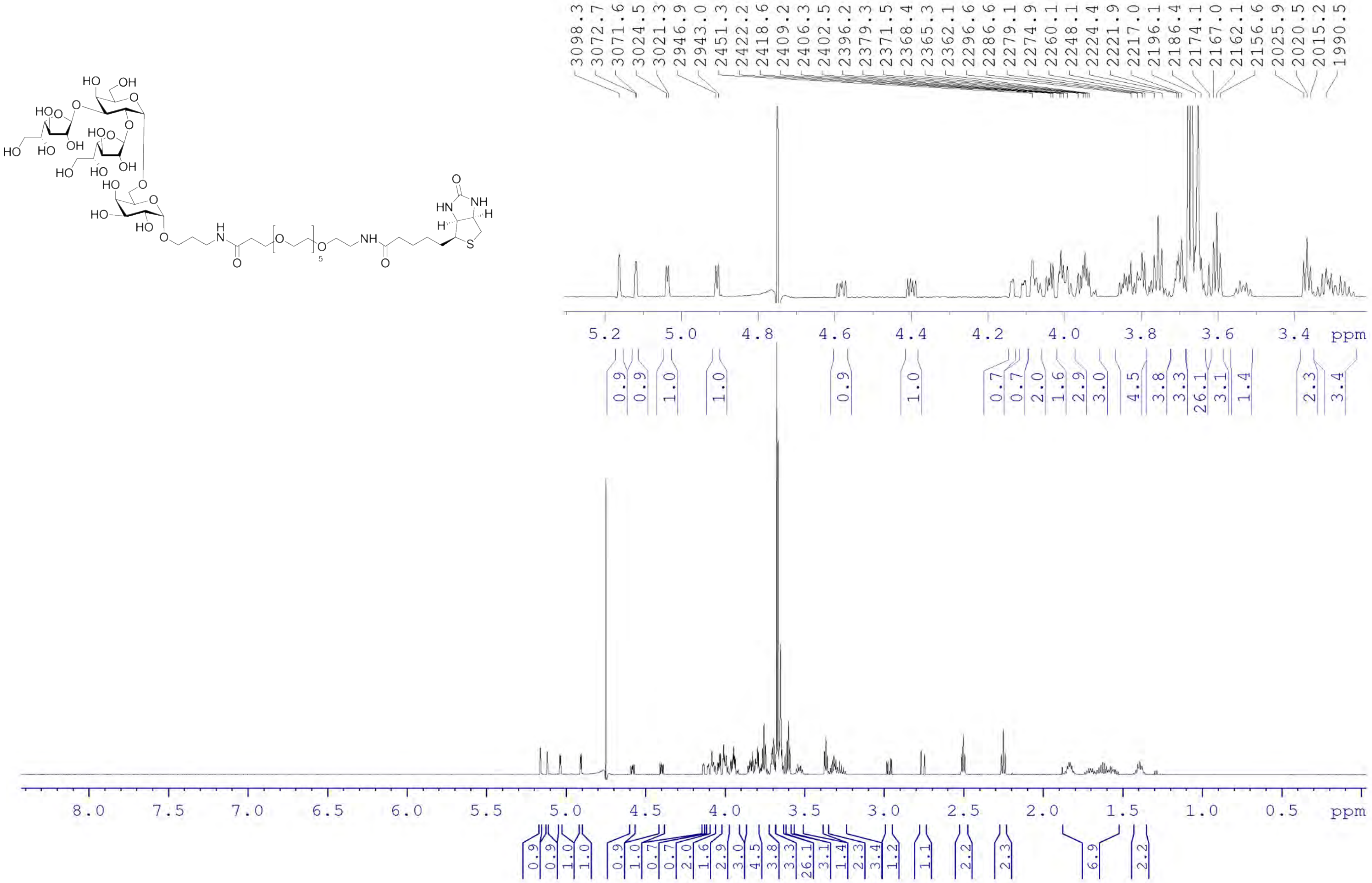

HRMS for **3b**

Comment CH3CN : H2O 50/50 %, dil. 200, calibrant added

Acquisition Parameter

Source Type	ESI	Ion Polarity	Positive	Set Nebulizer	0.5 Bar
Focus	Active			Set Dry Heater	180 °C
Scan Begin	50 m/z	Set Capillary	4500 V	Set Dry Gas	4.0 l/min
Scan End	3000 m/z	Set End Plate Offset	-500 V	Set Divert Valve	Waste

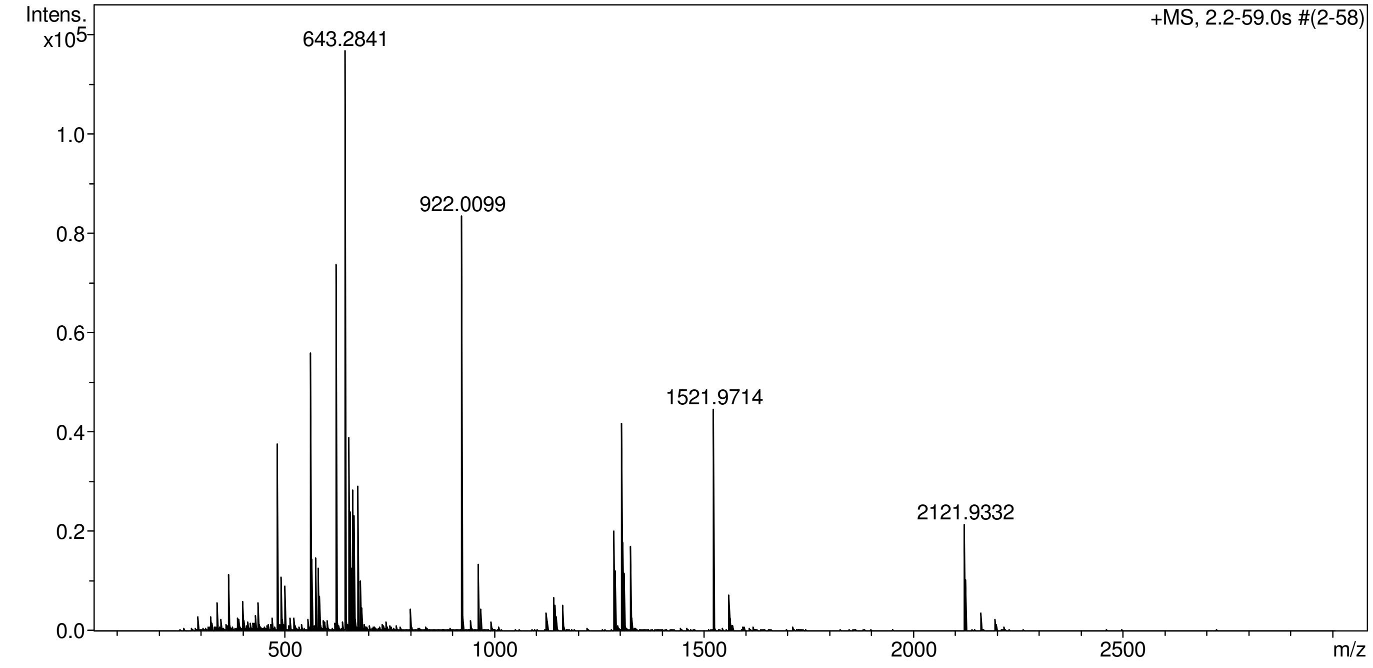

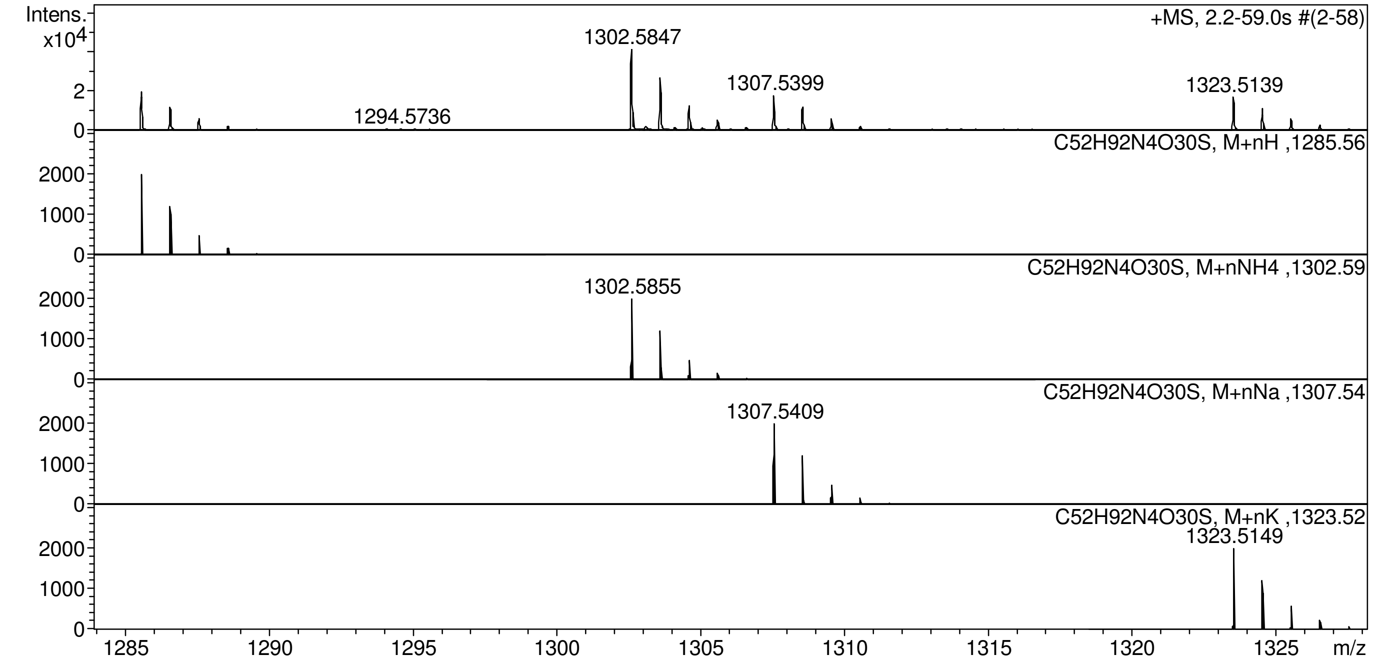

^{1}H-NMR of **31** (300 MHz, $CDCl_3$)

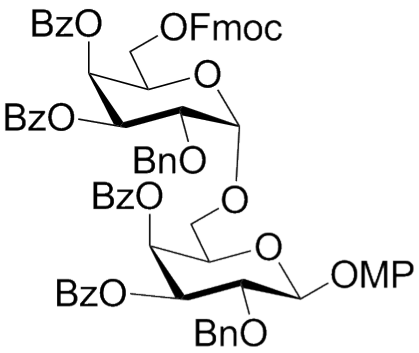

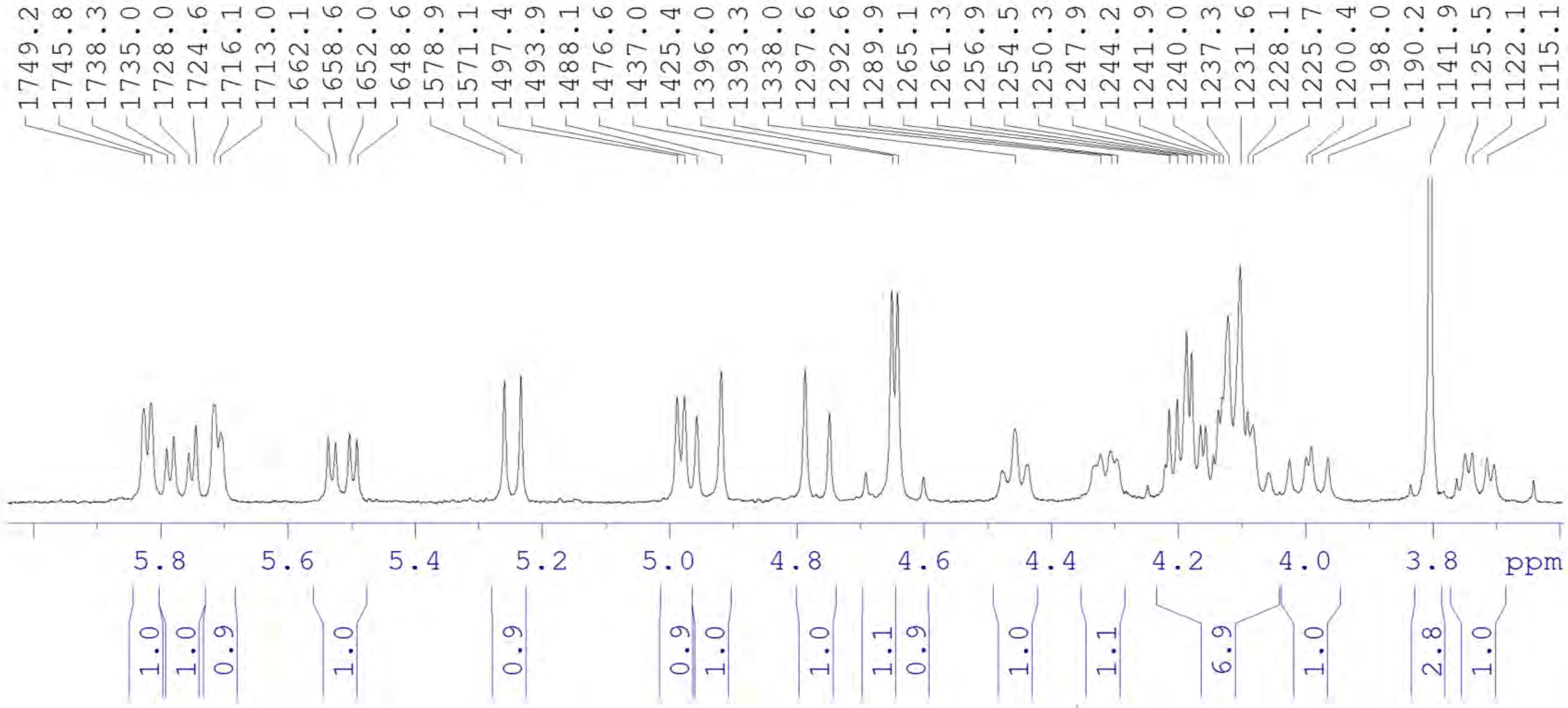

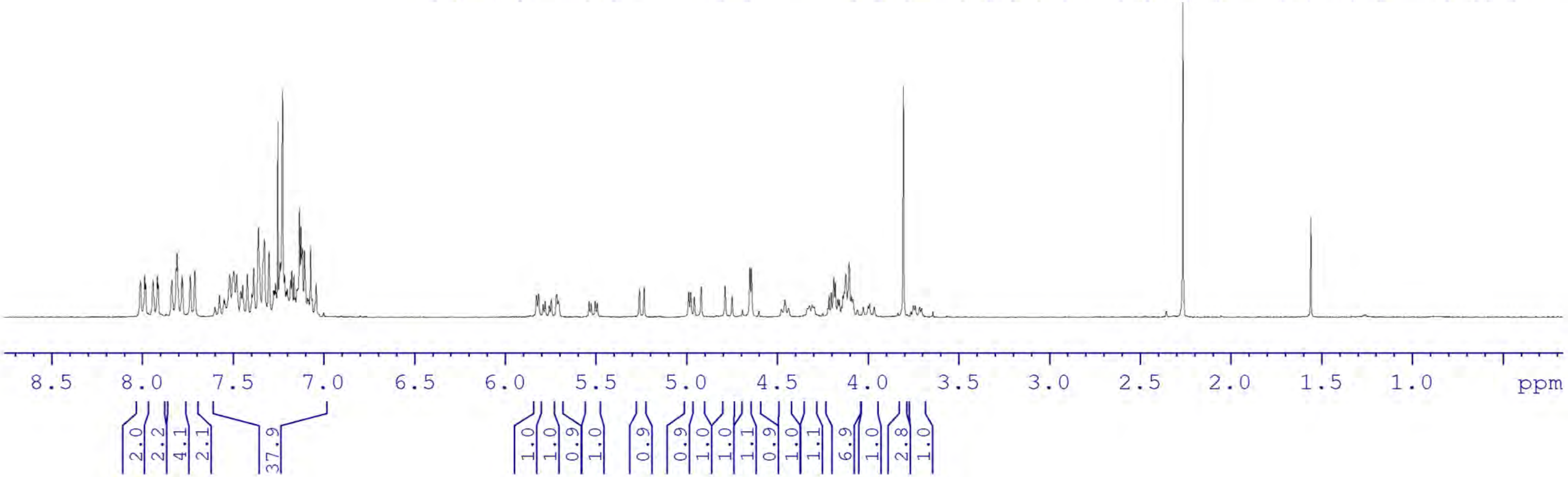

^{13}C-NMR of **31** (75 MHz, $CDCl_3$)

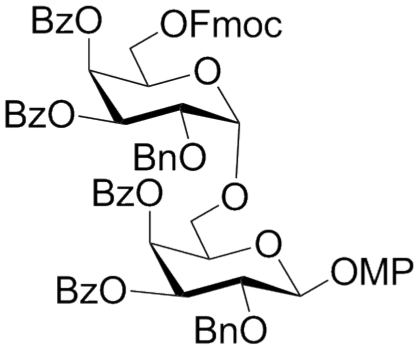

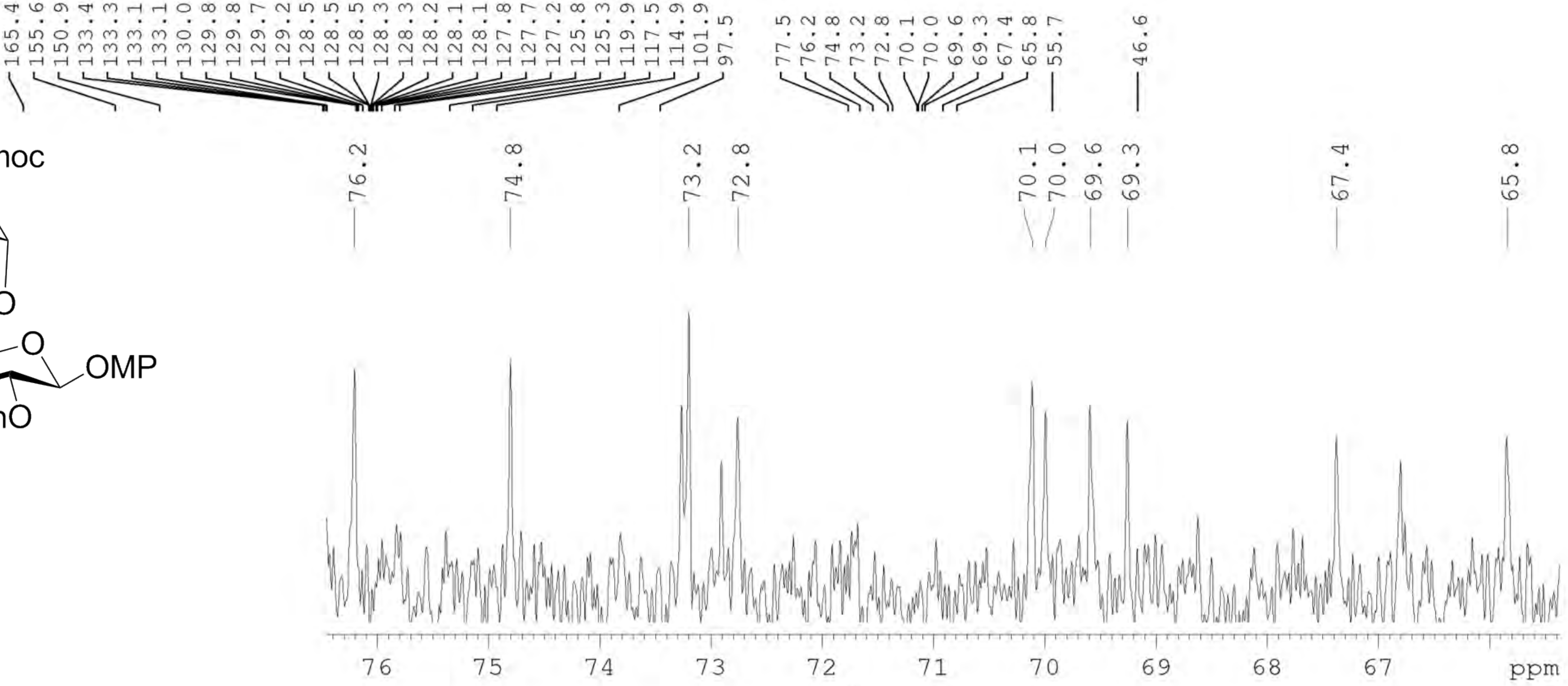

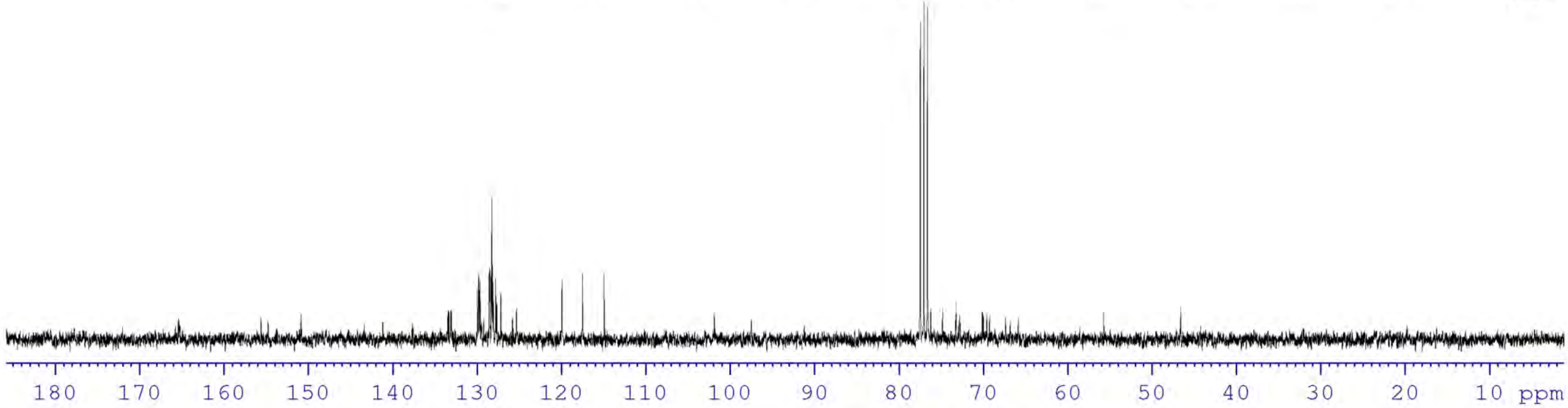

^{1}H-^{1}H COSY of **31** (300 MHz, $CDCl_3$)

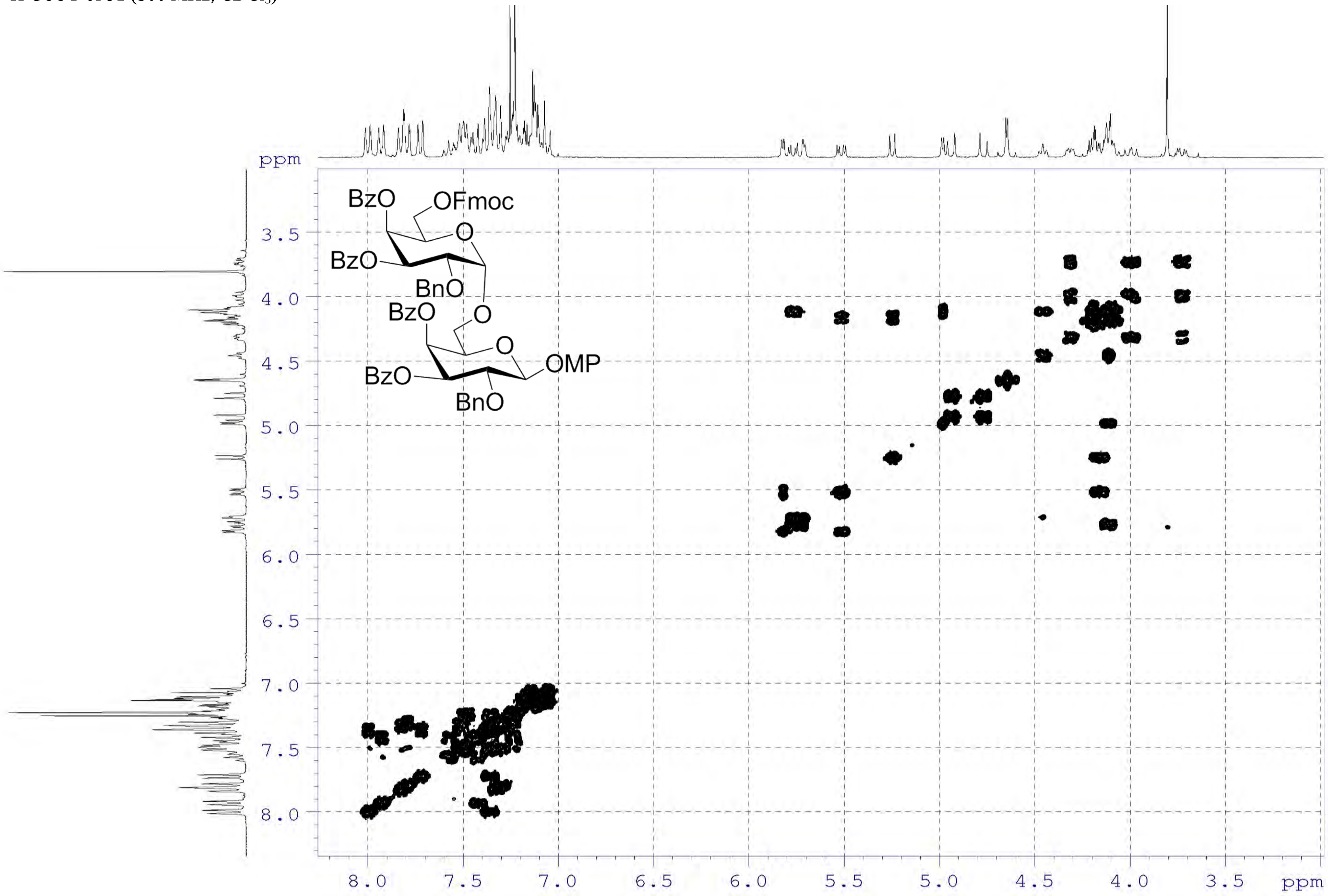

$^{1}H-^{13}C$ HSQC of **31** (300 MHz, $CDCl_3$)

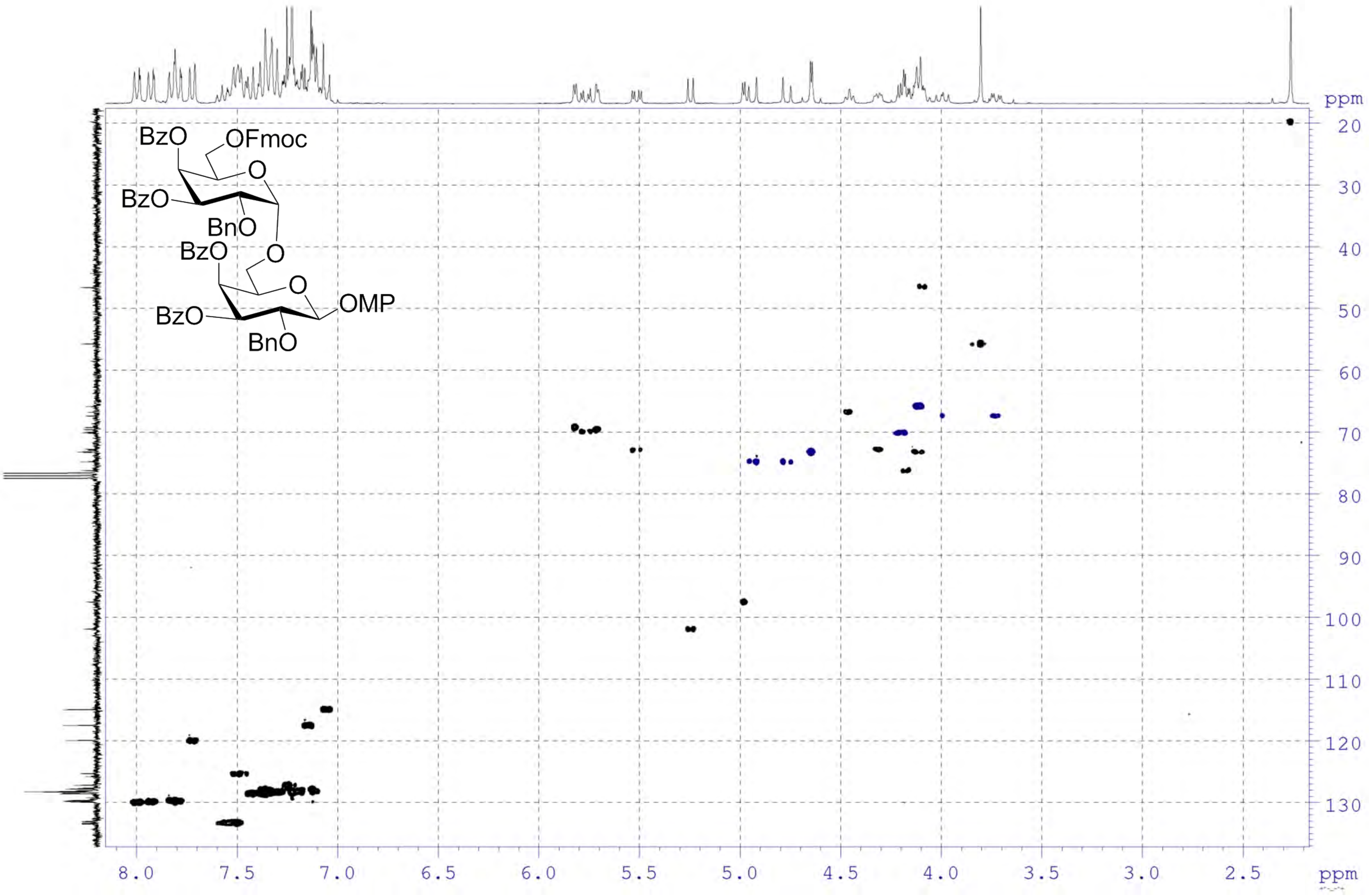

^{1}H-NMR of **32** (300 MHz, $CDCl_3$)

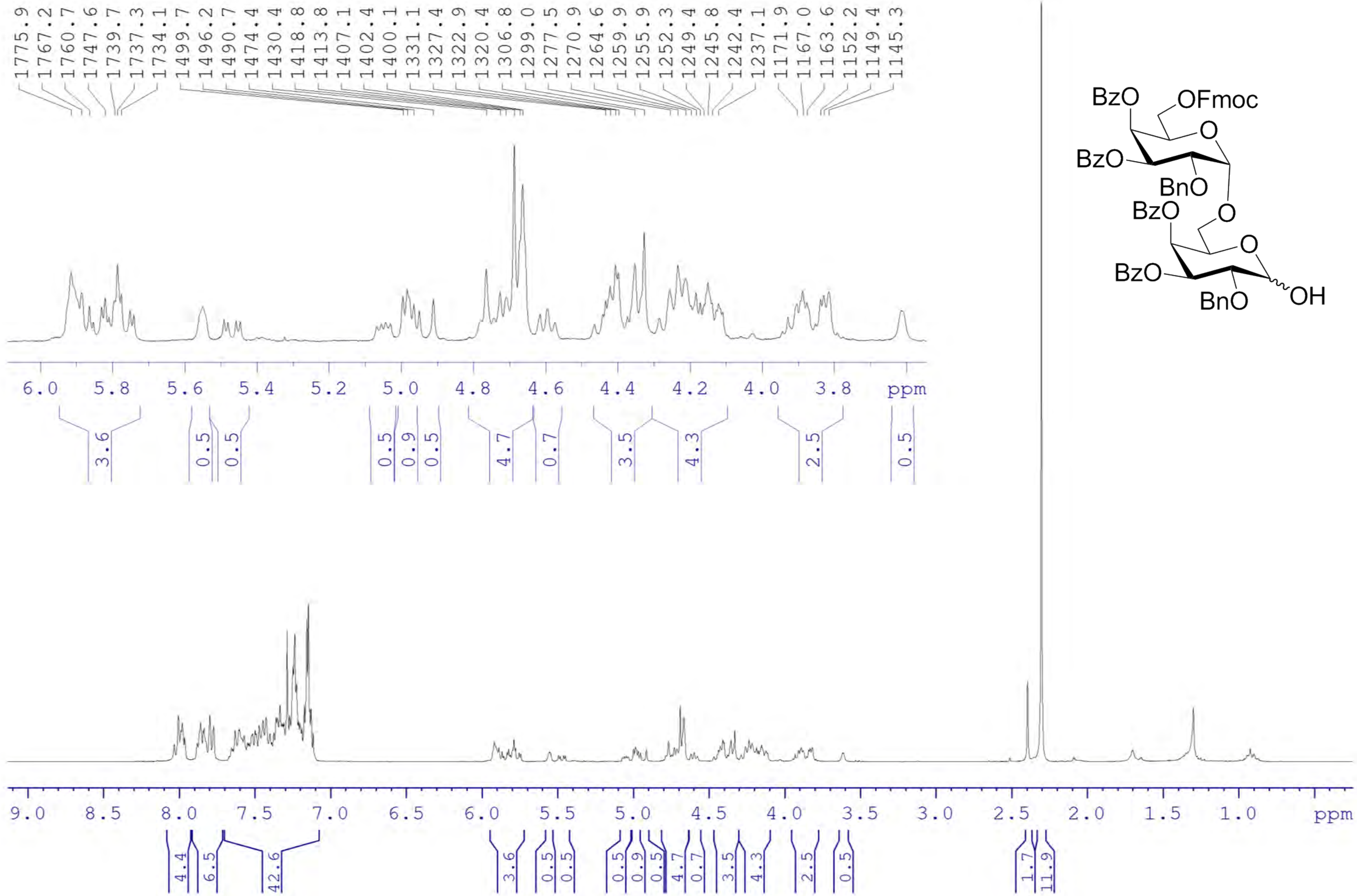

^{13}C-NMR of **32** (75 MHz, $CDCl_3$)

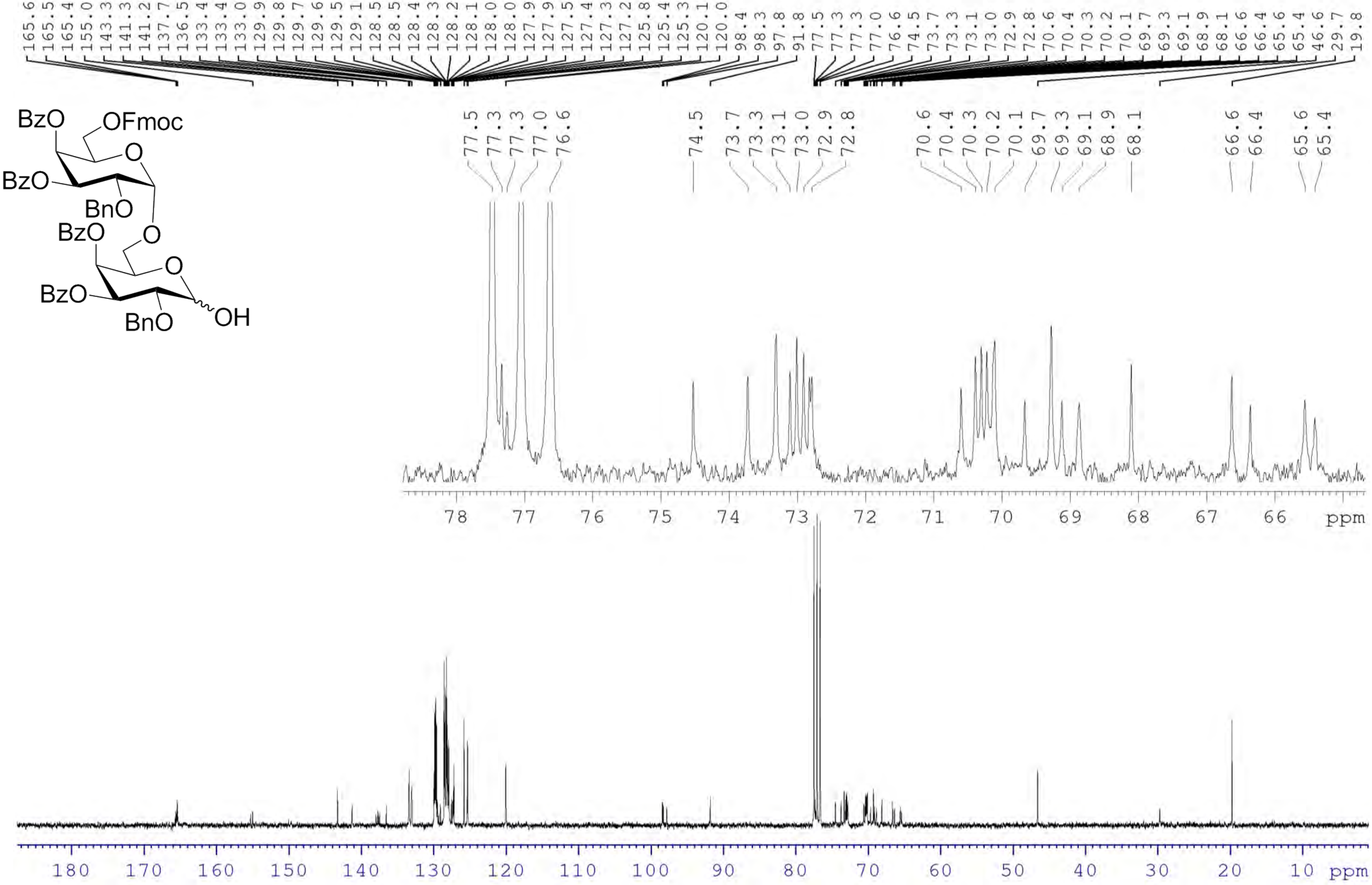

^{1}H-^{1}H COSY of **32** (300 MHz, $CDCl_3$)

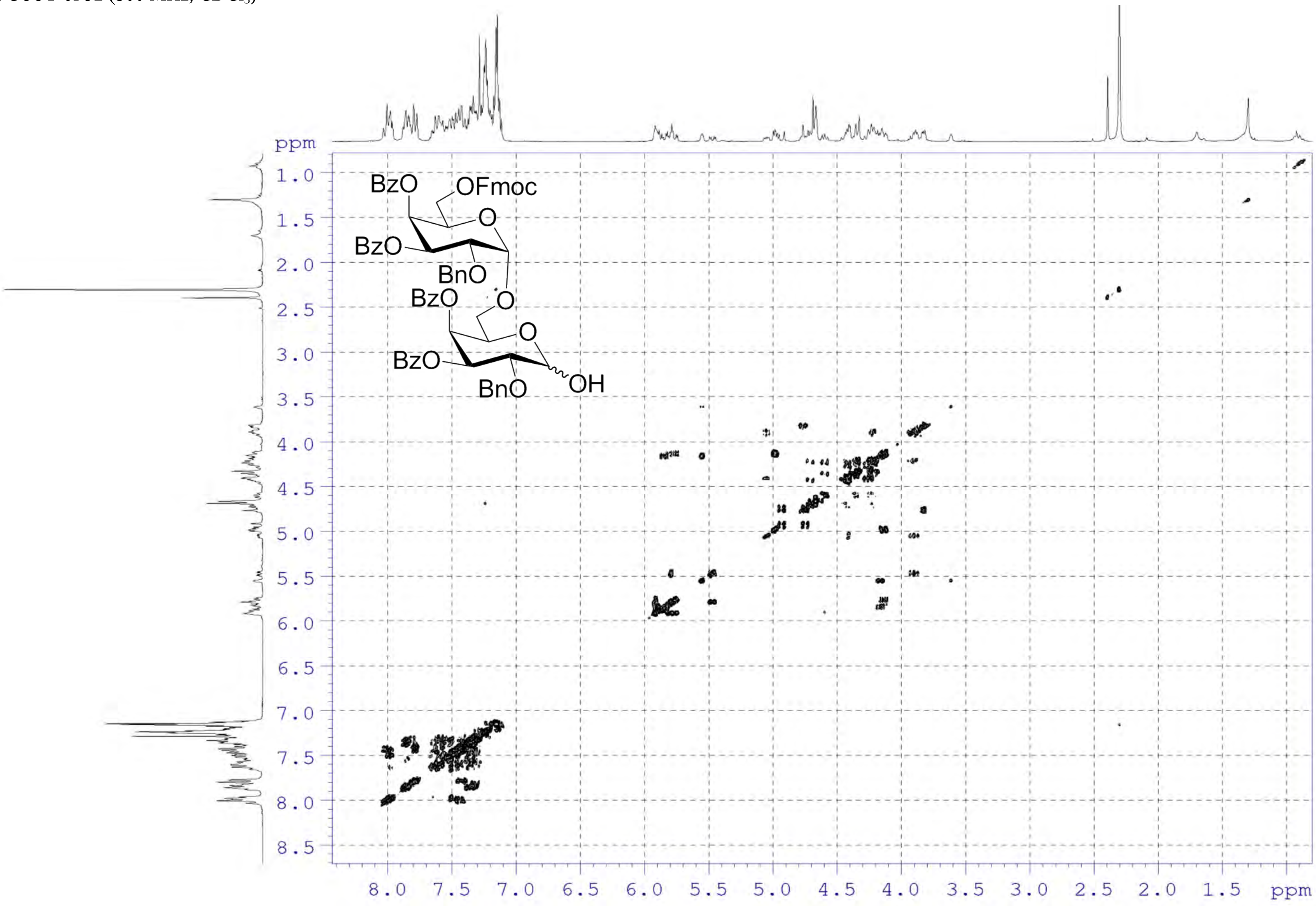

^{1}H-^{13}C HSQC of **32** (300 MHz, $CDCl_3$)

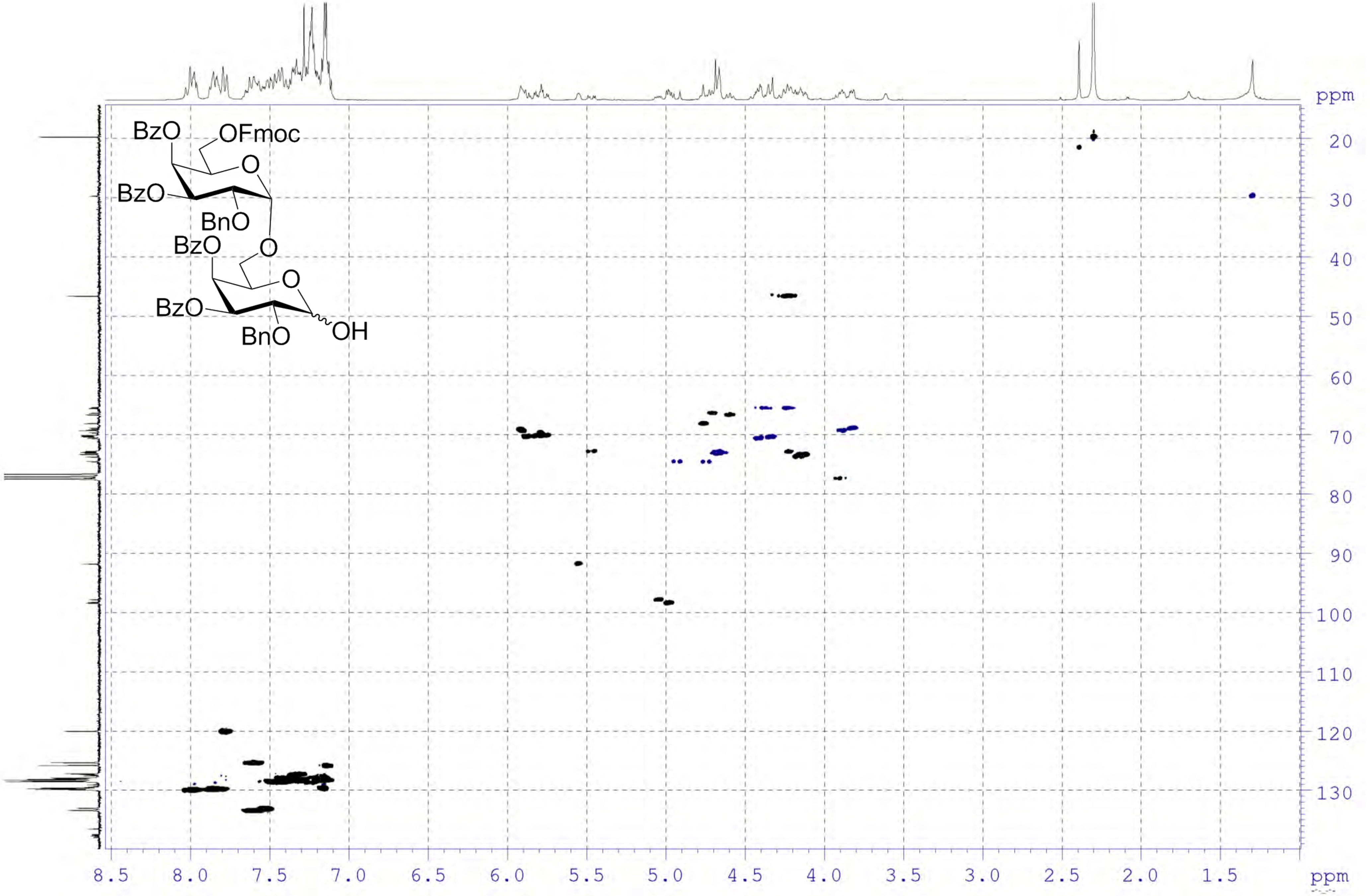

^{1}H-NMR of **33** (600 MHz, $CDCl_3$, 323K)

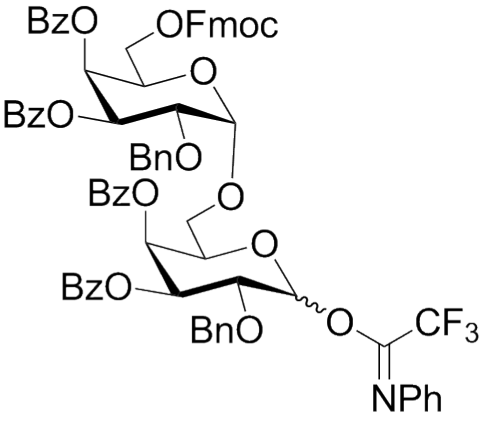

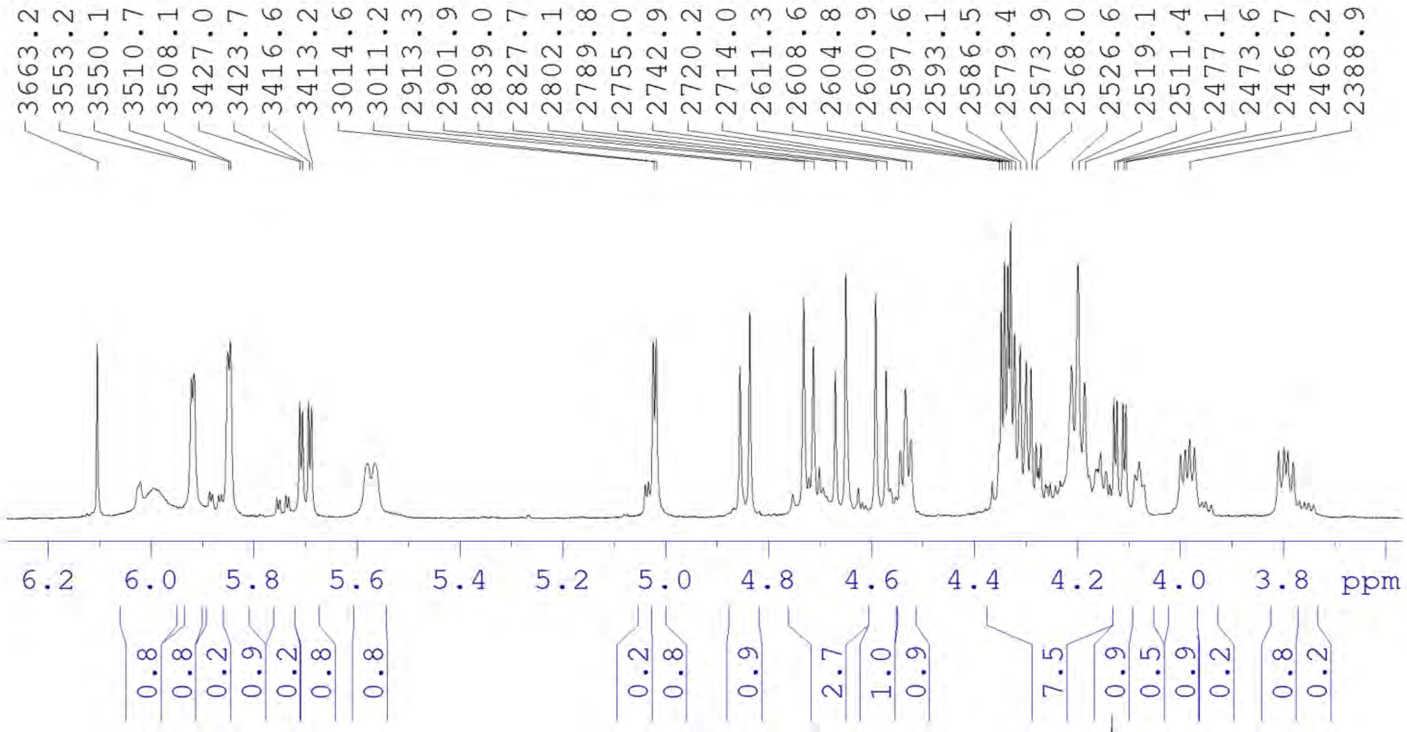

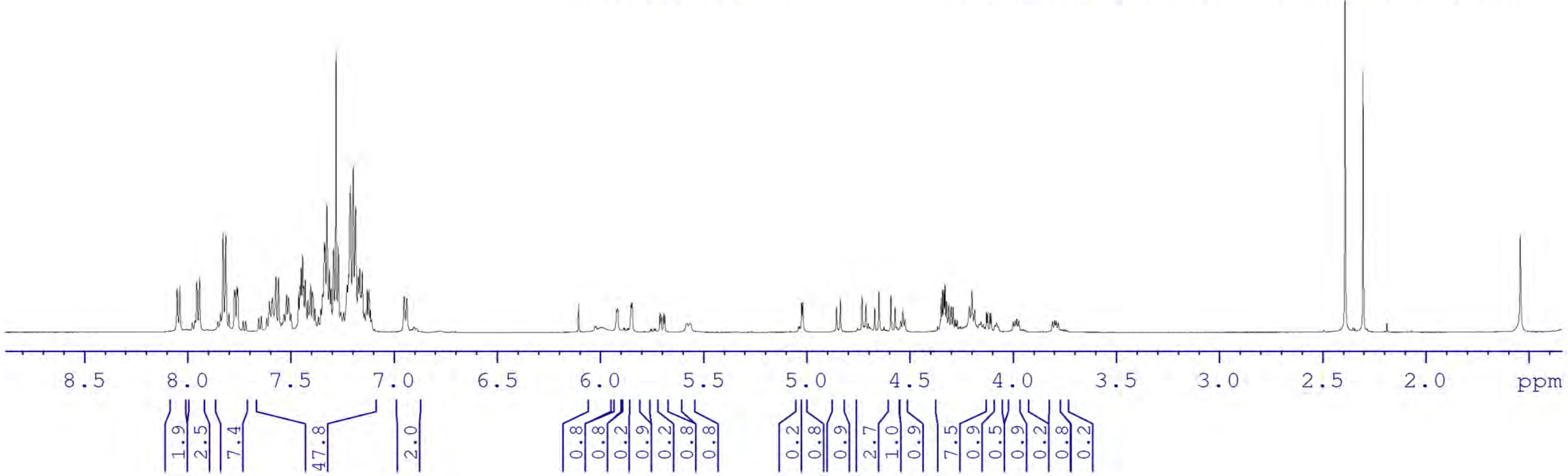

^{13}C-NMR of **33** (150 MHz, $CDCl_3$, 323K)

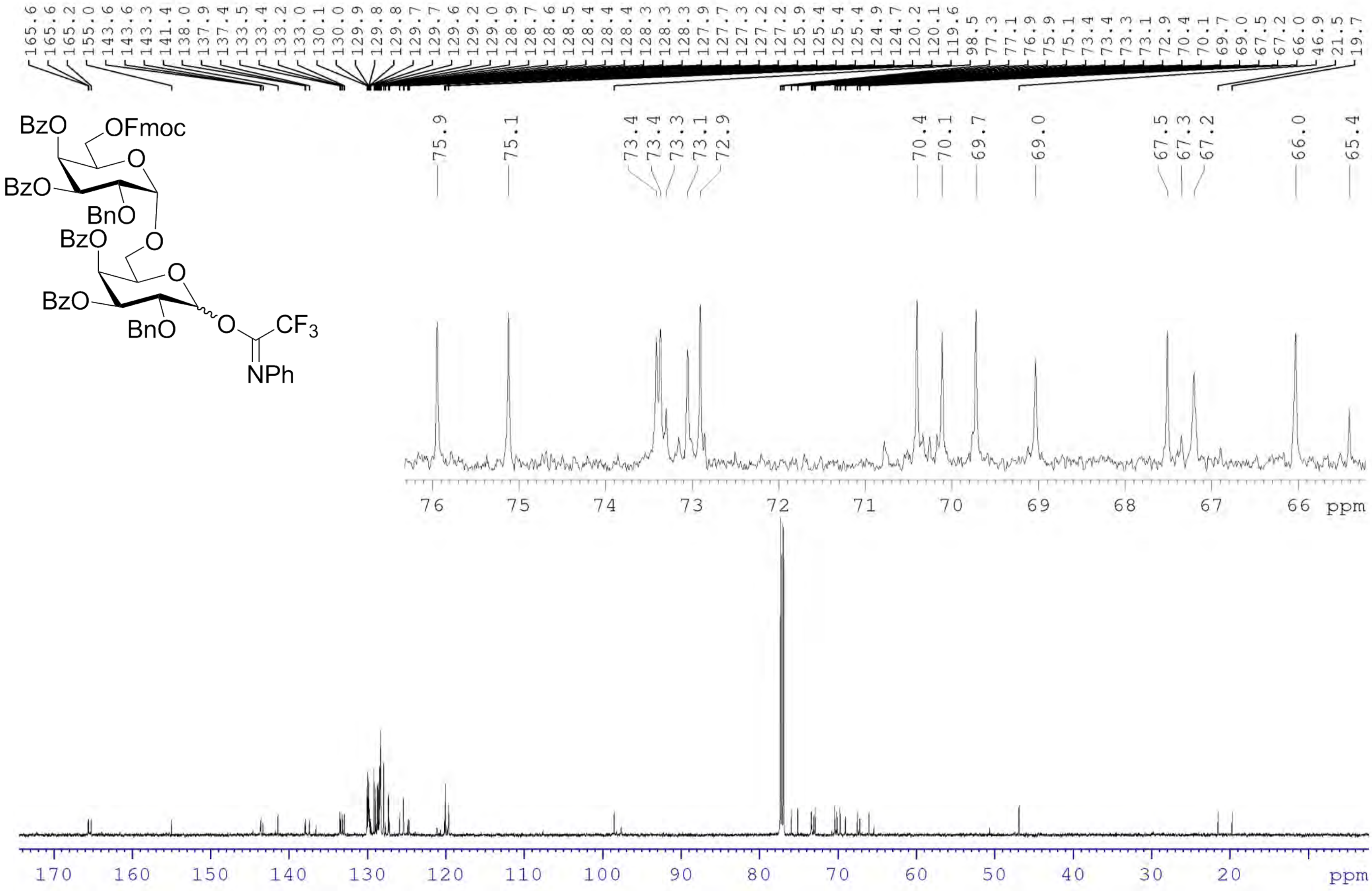

^{1}H-^{1}H COSY **33** (600 MHz, $CDCl_3$, 323K)

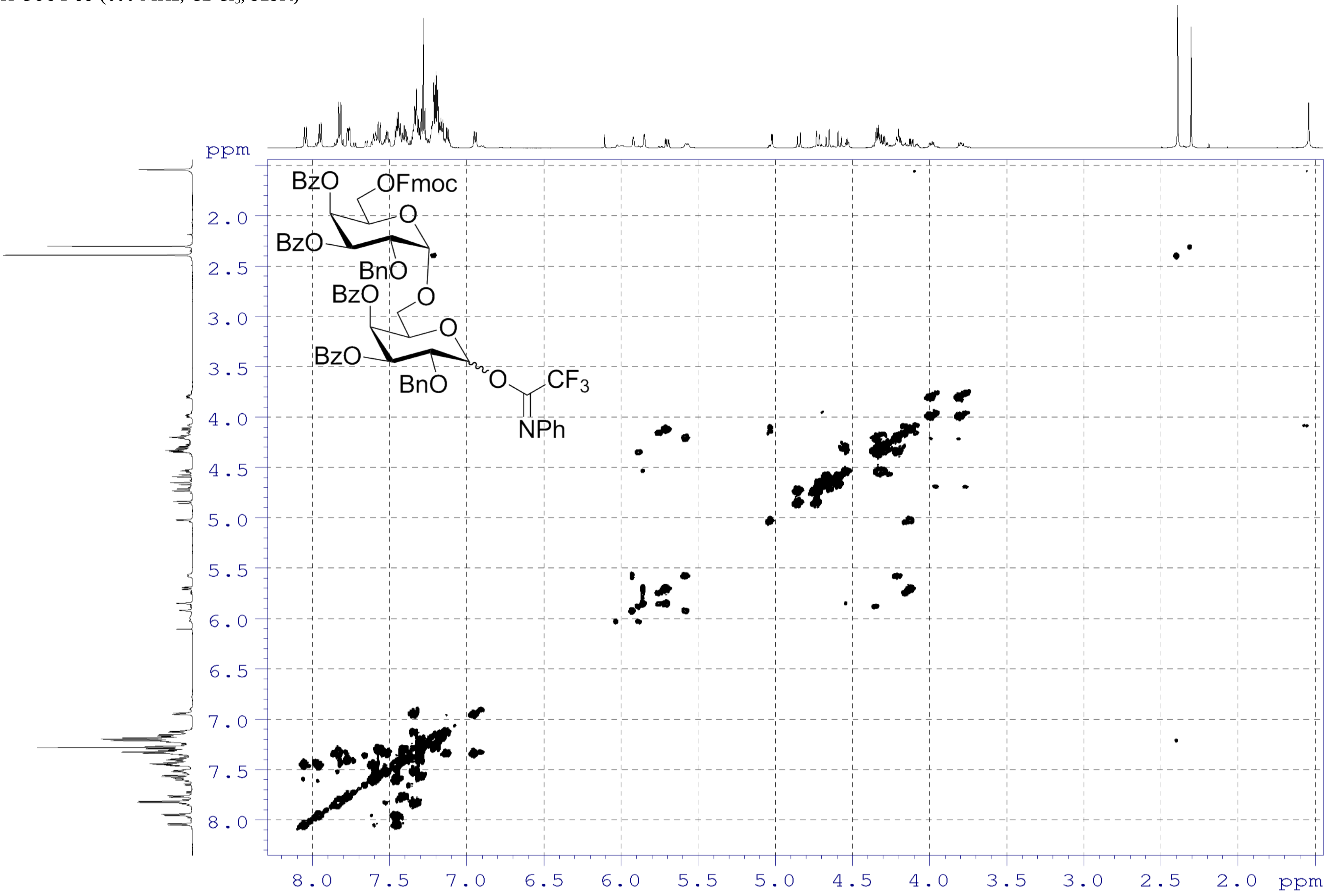

^{1}H-^{13}C HSQC of **33** (600 MHz, $CDCl_3$, 323K)

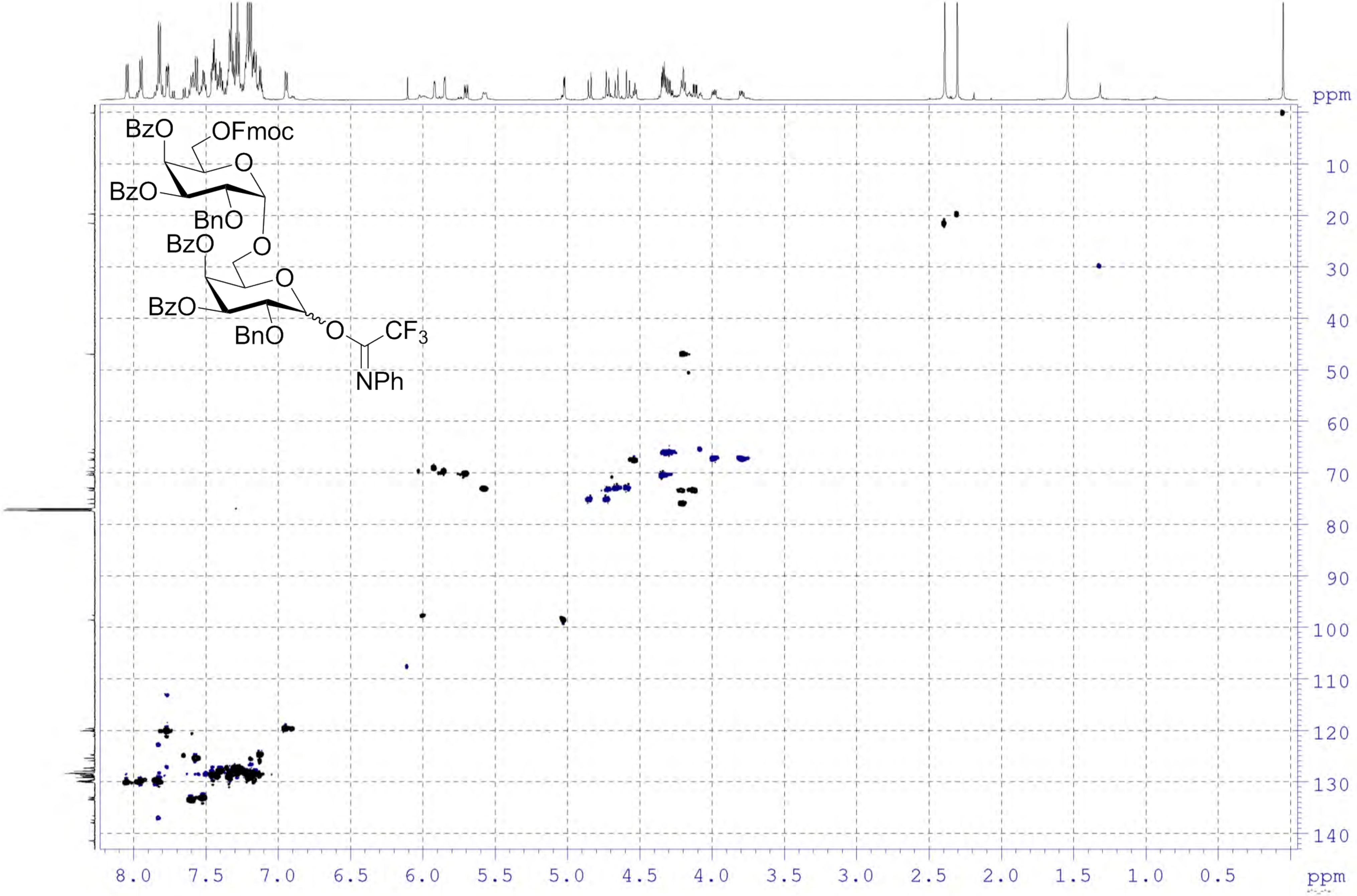

^{1}H-NMR of **34** (600 MHz, $CDCl_3$)

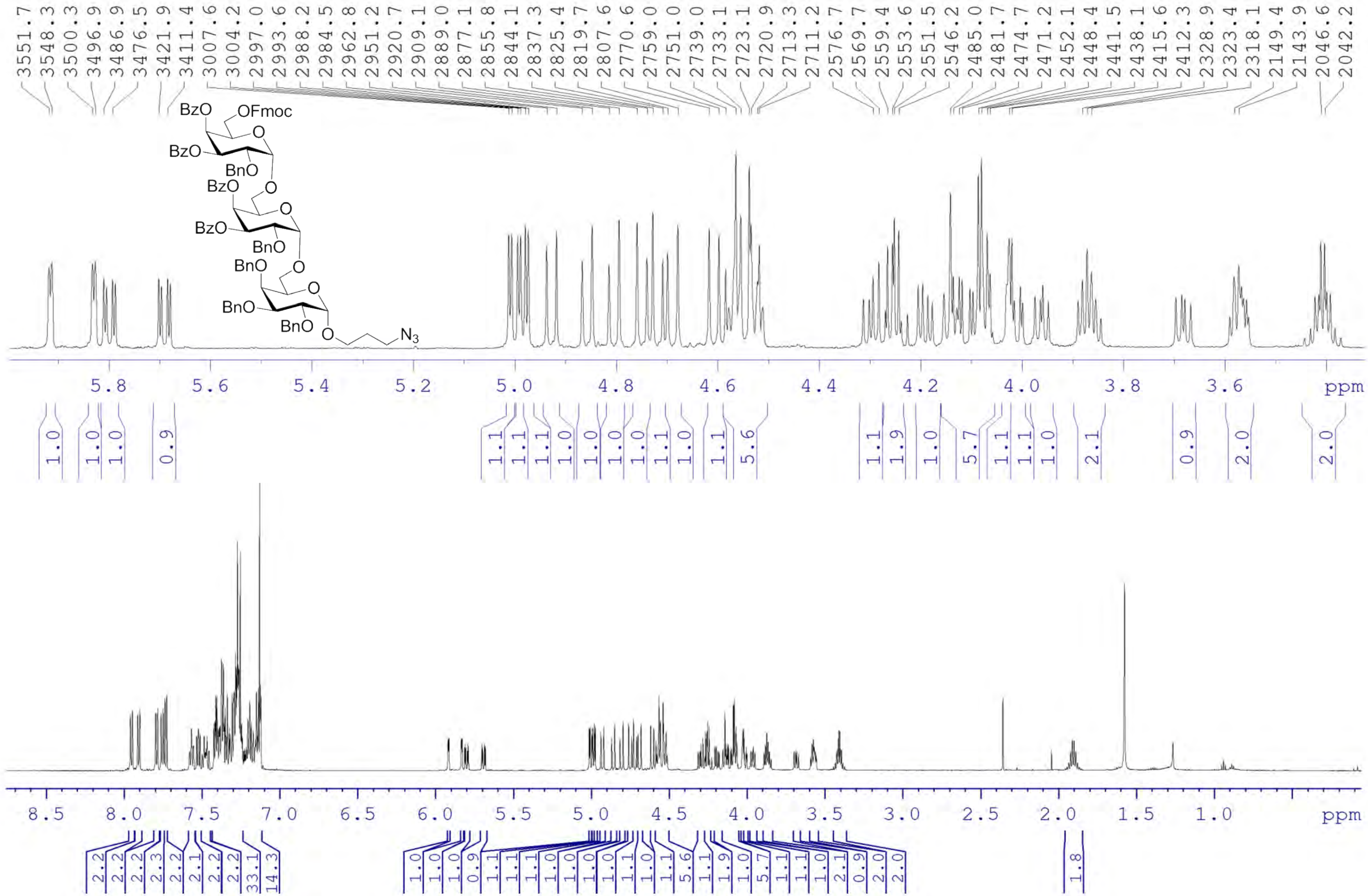

^{13}C-NMR of **34** (150 MHz, $CDCl_3$)

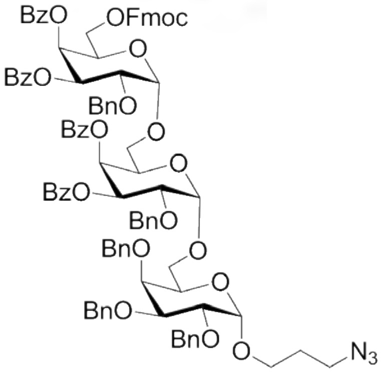

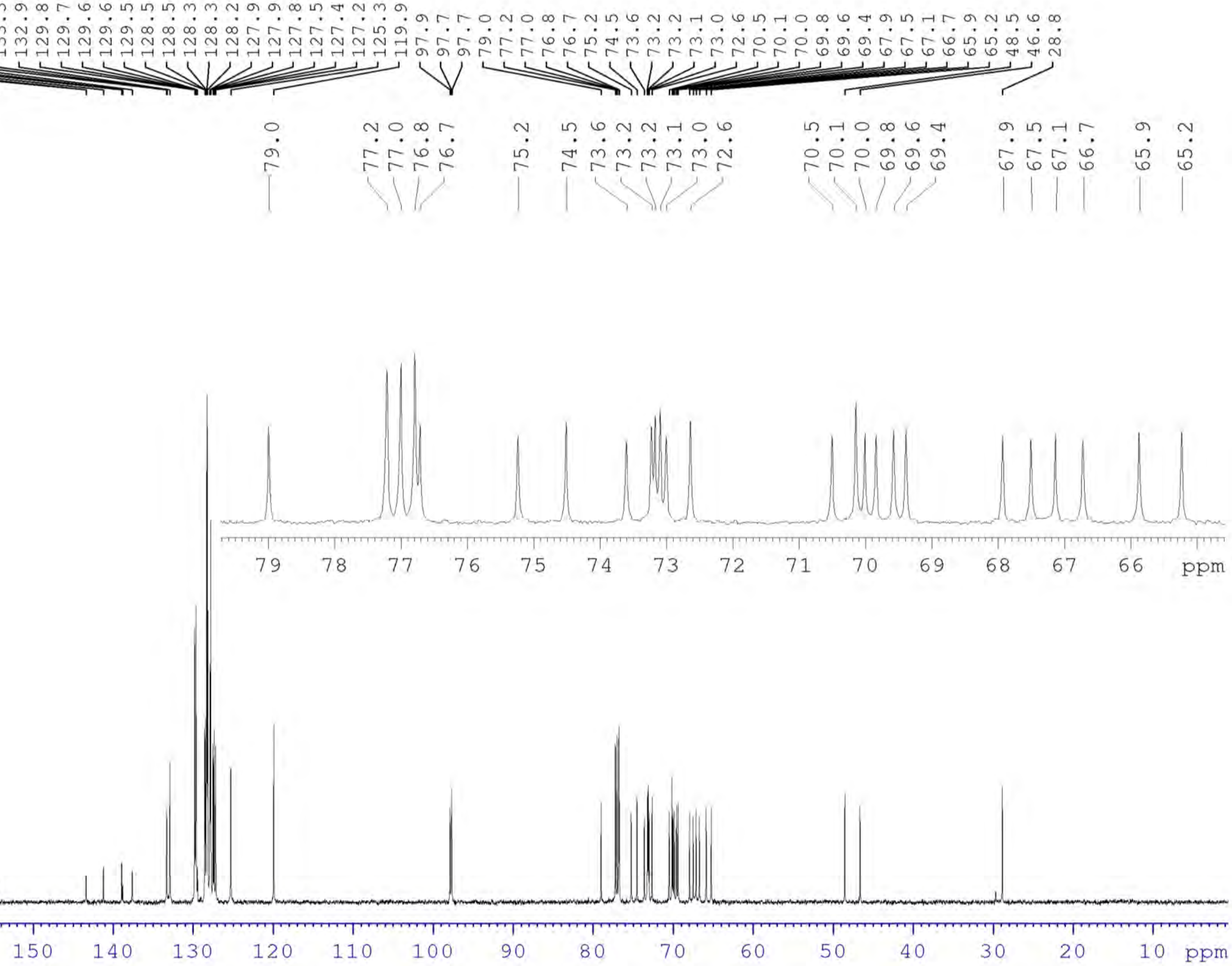

^{1}H-^{1}H COSY of **34** (600 MHz, $CDCl_3$)

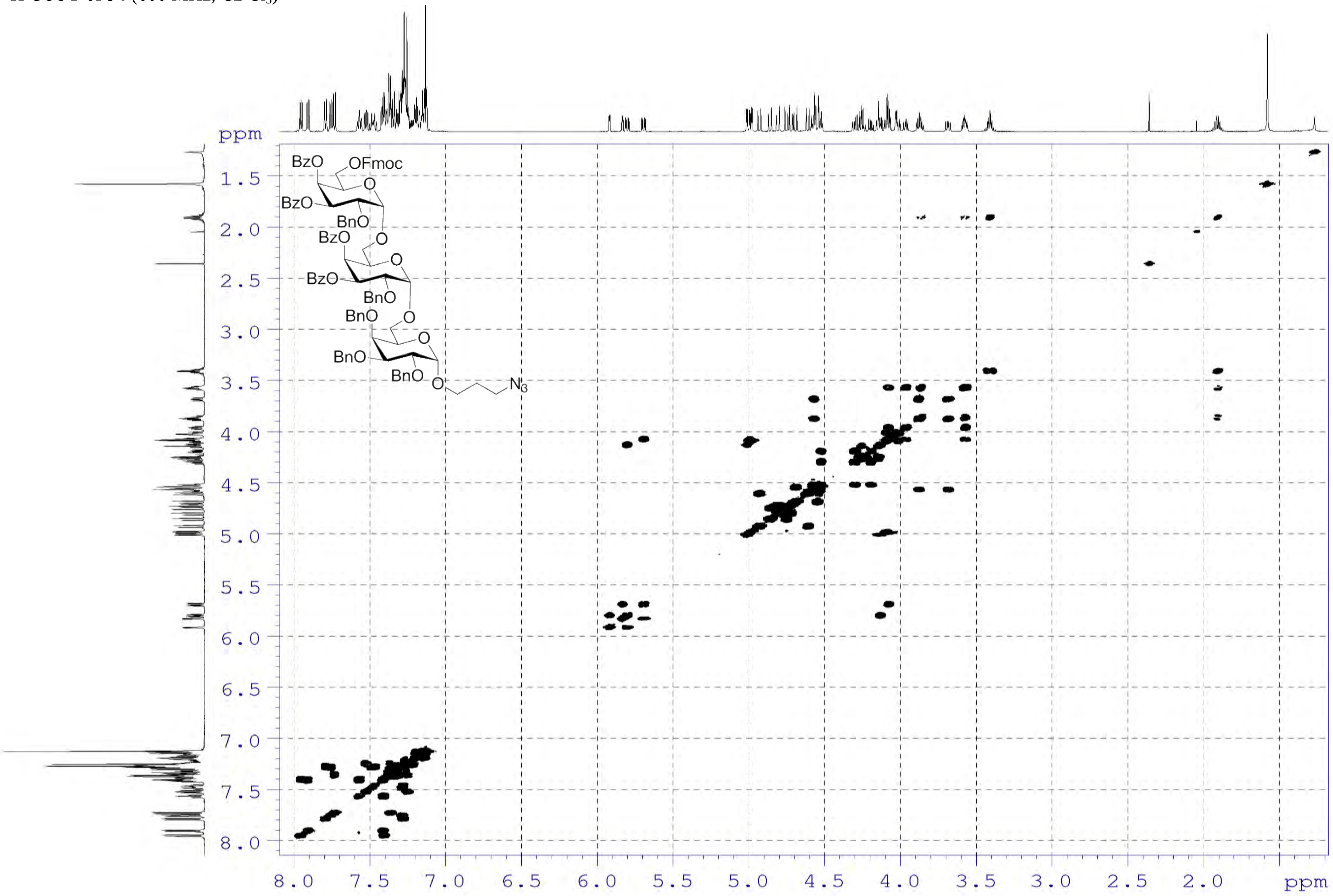

^{1}H-^{13}C HSQC of **34** (600 MHz, $CDCl_3$)

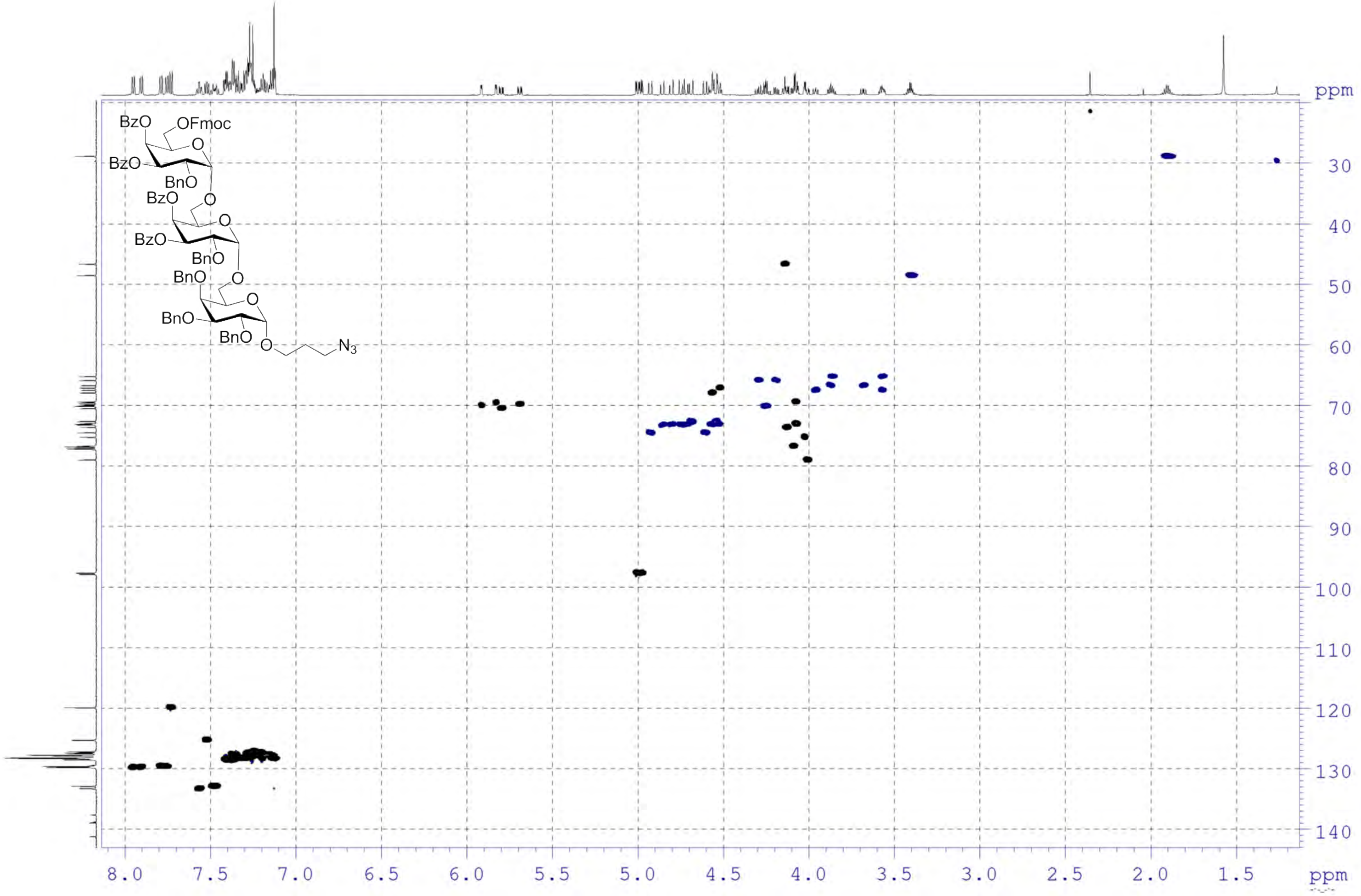

^{1}H-NMR of **35** (600 MHz, $CDCl_3$)

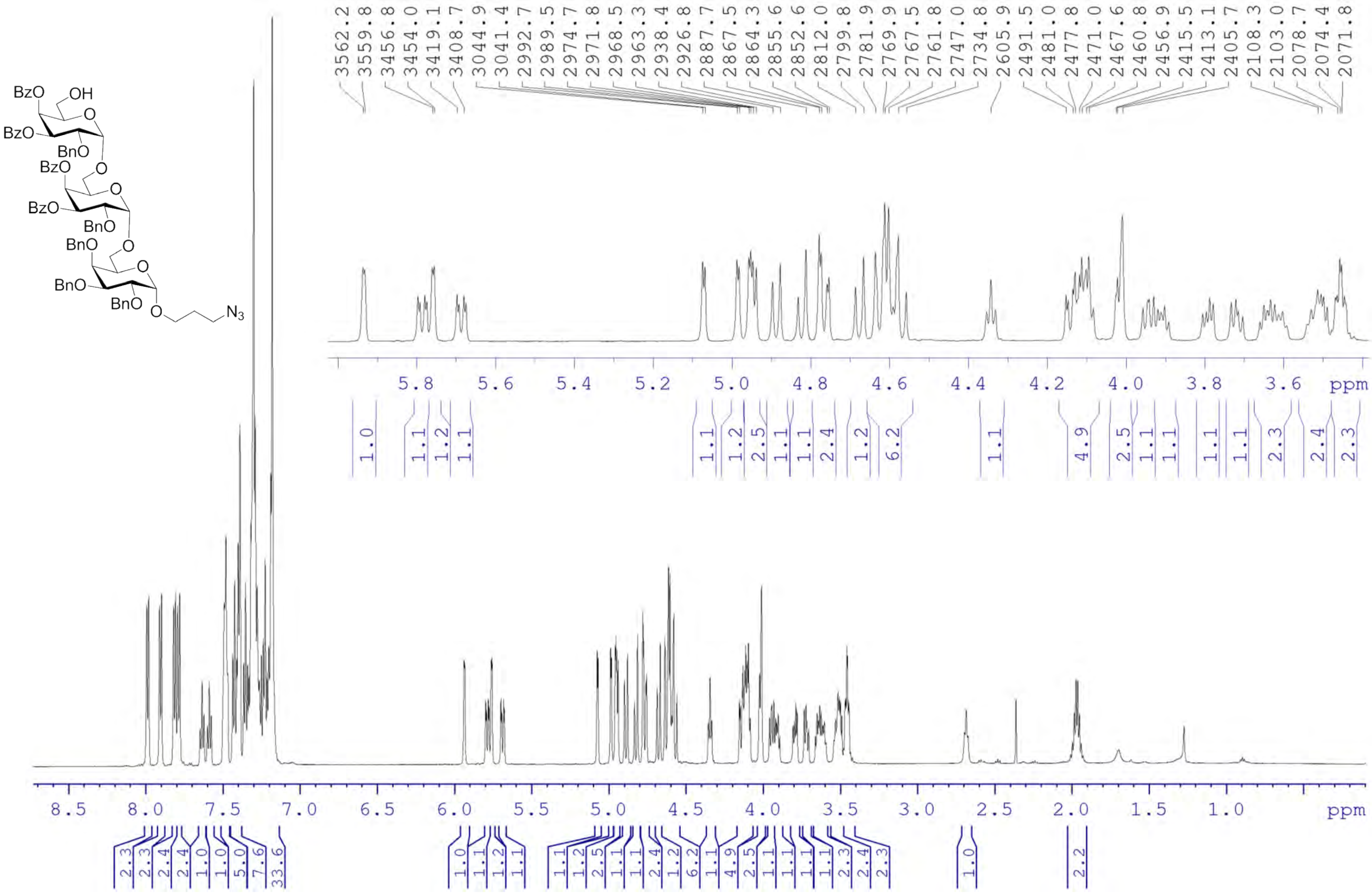

^{13}C-NMR of **35** (150 MHz, $CDCl_3$)

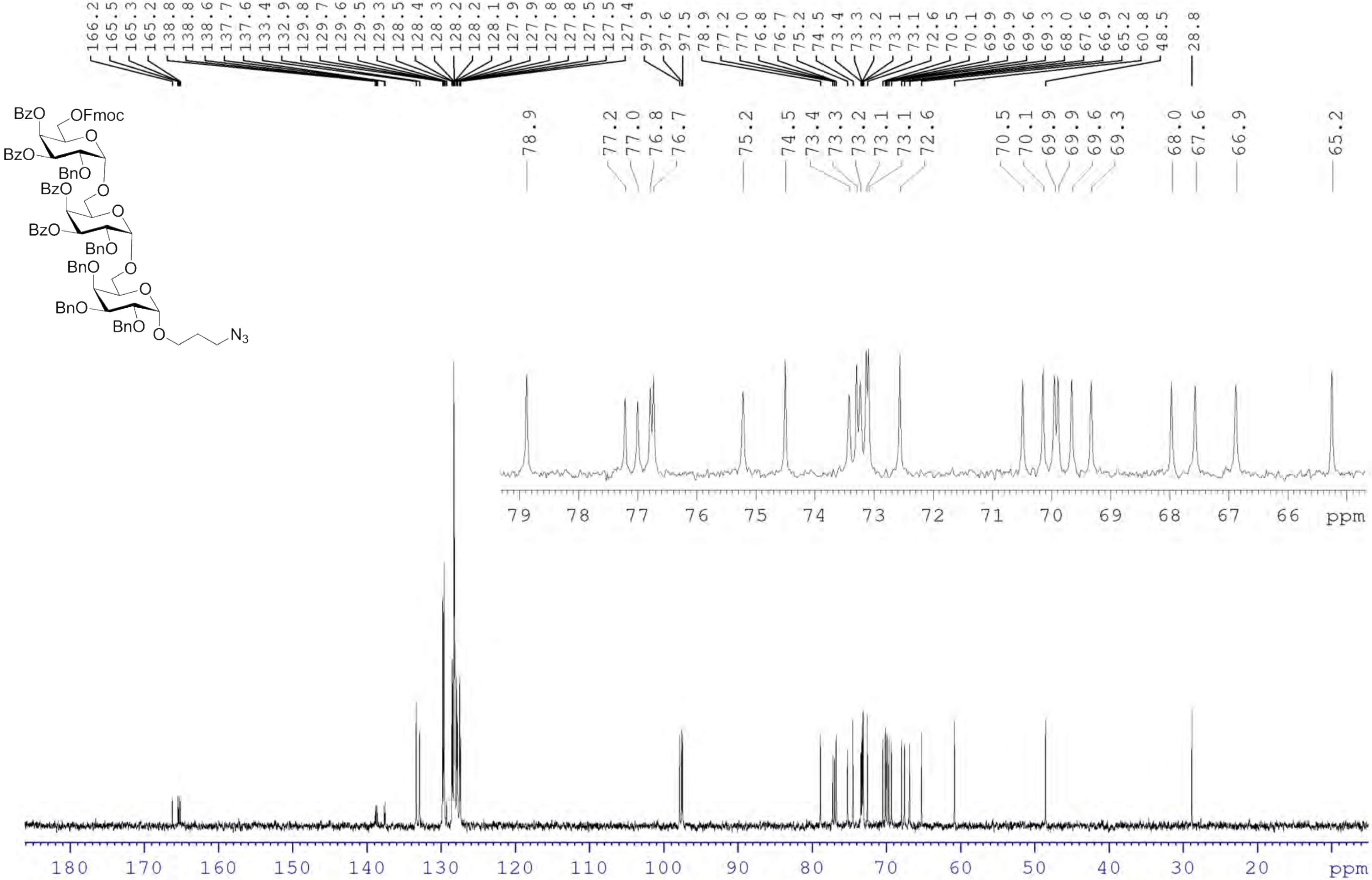

^{1}H-^{1}H COSY of **35** (600 MHz, $CDCl_3$)

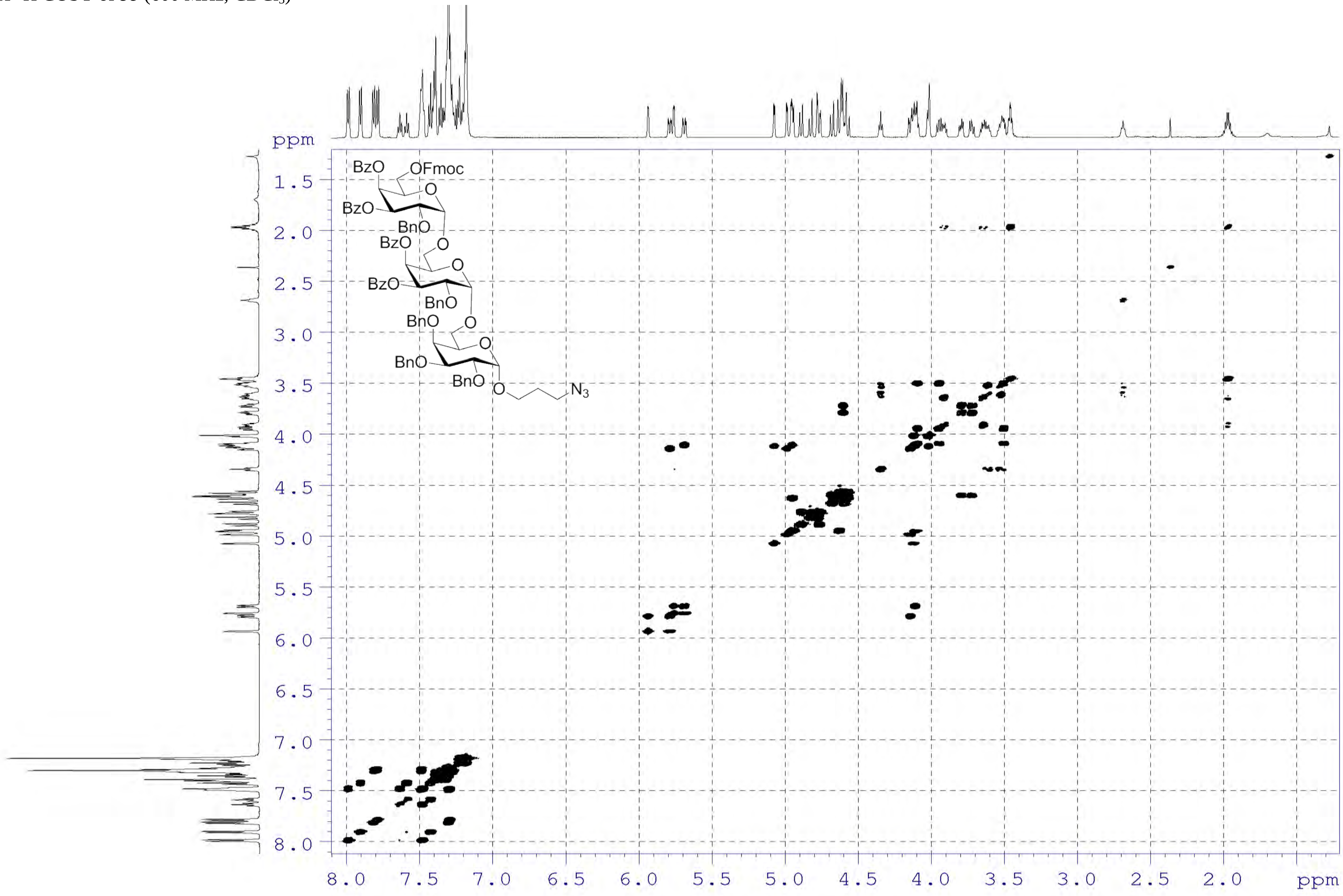

^{1}H-^{13}C HSQC of **35** (600 MHz, $CDCl_3$)

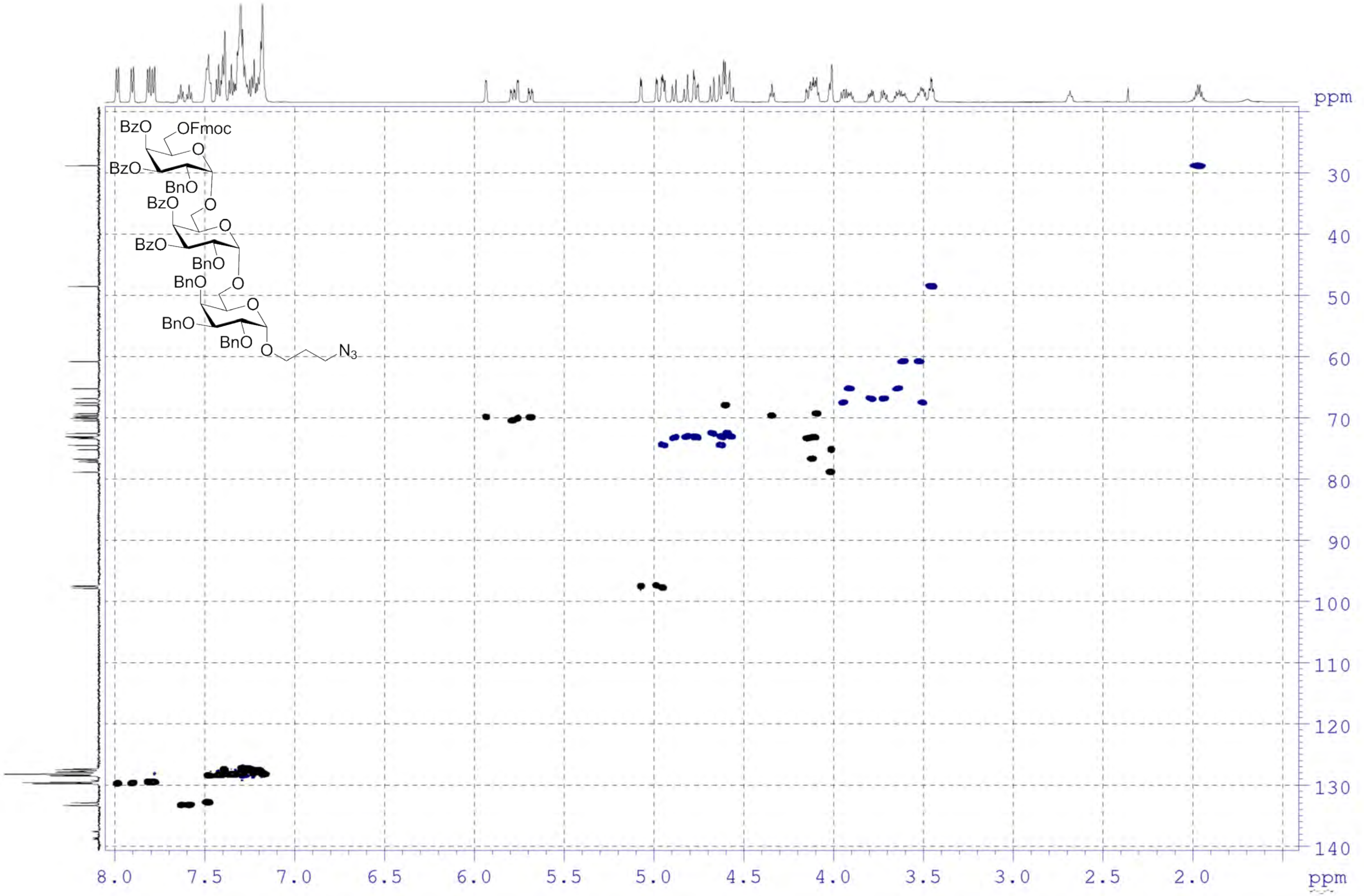

^{1}H-NMR of **36** (600 MHz, $CDCl_3$)

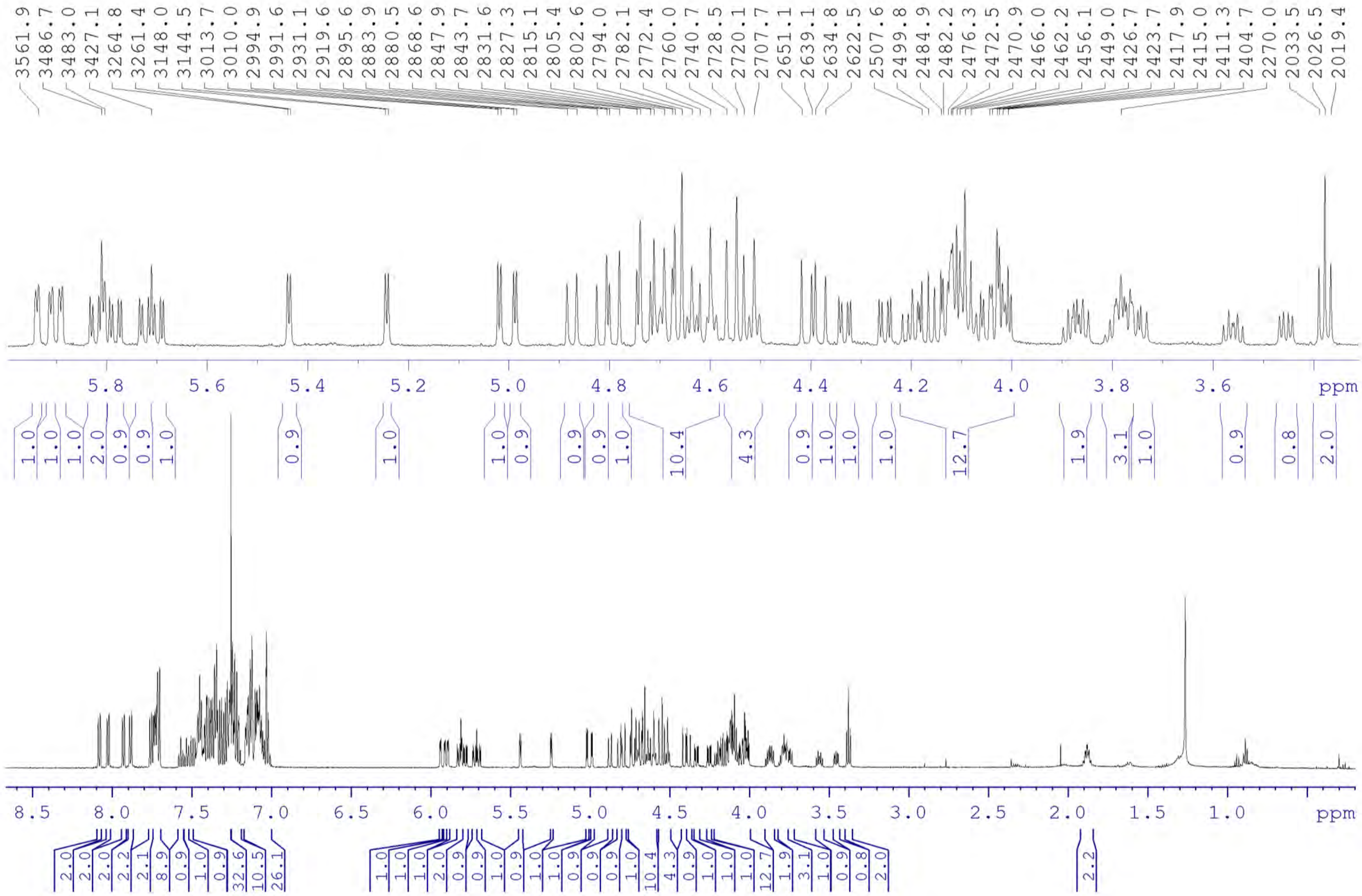

^{13}C-NMR of **36** (150 MHz, $CDCl_3$)

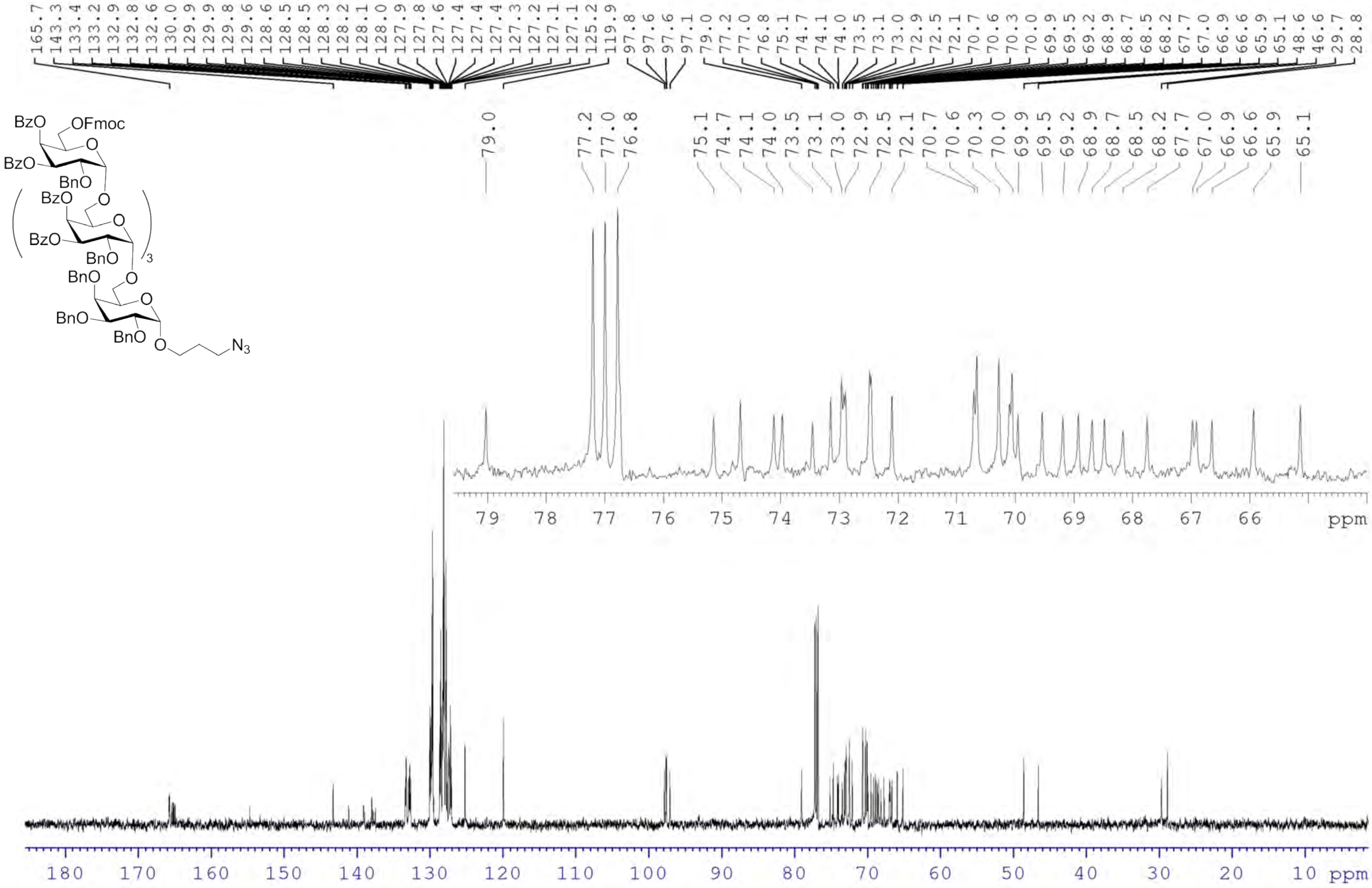

^{1}H-^{1}H COSY **36** (600 MHz, $CDCl_3$)

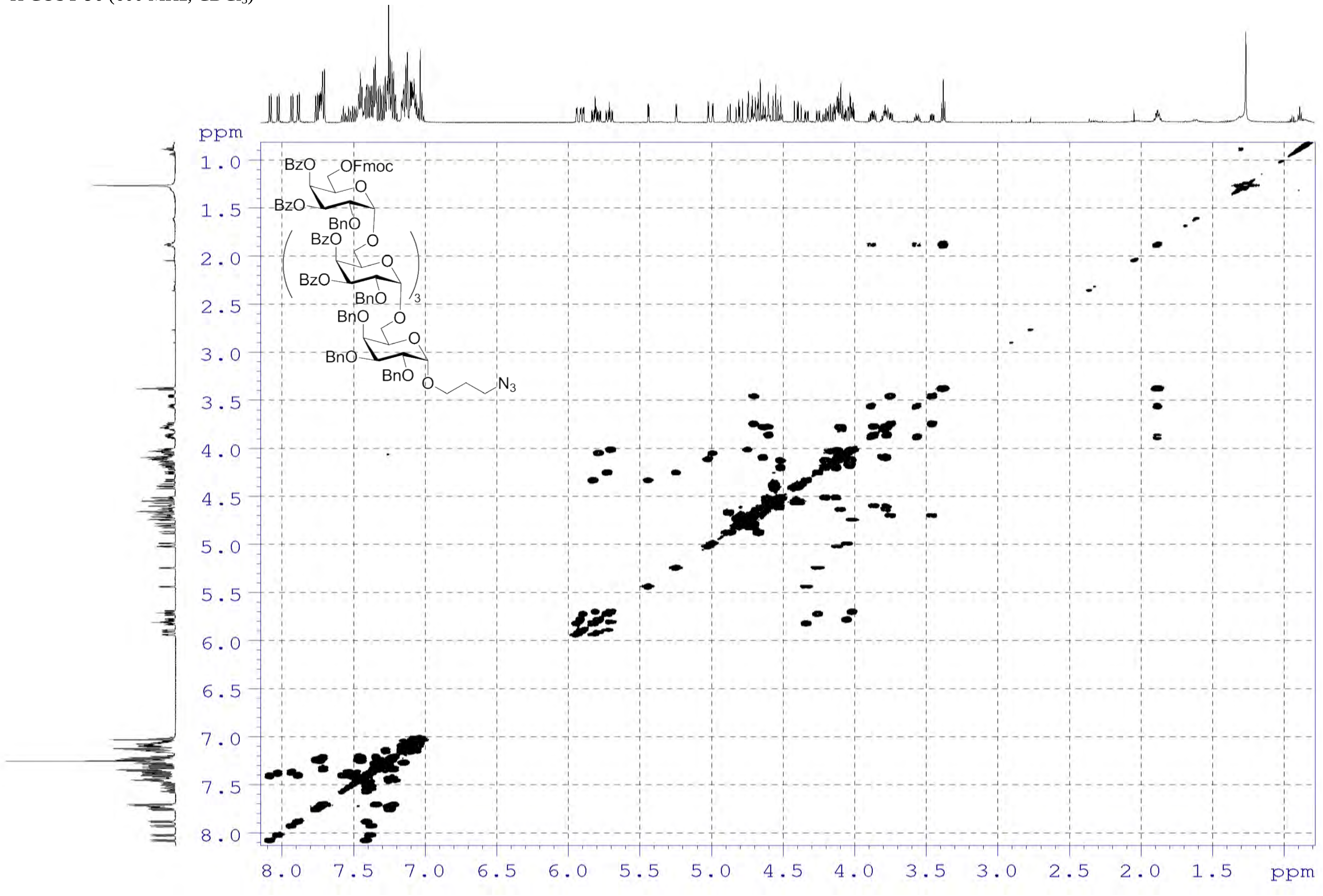

^{1}H-^{13}C HSQC of **36** (600 MHz, $CDCl_3$)

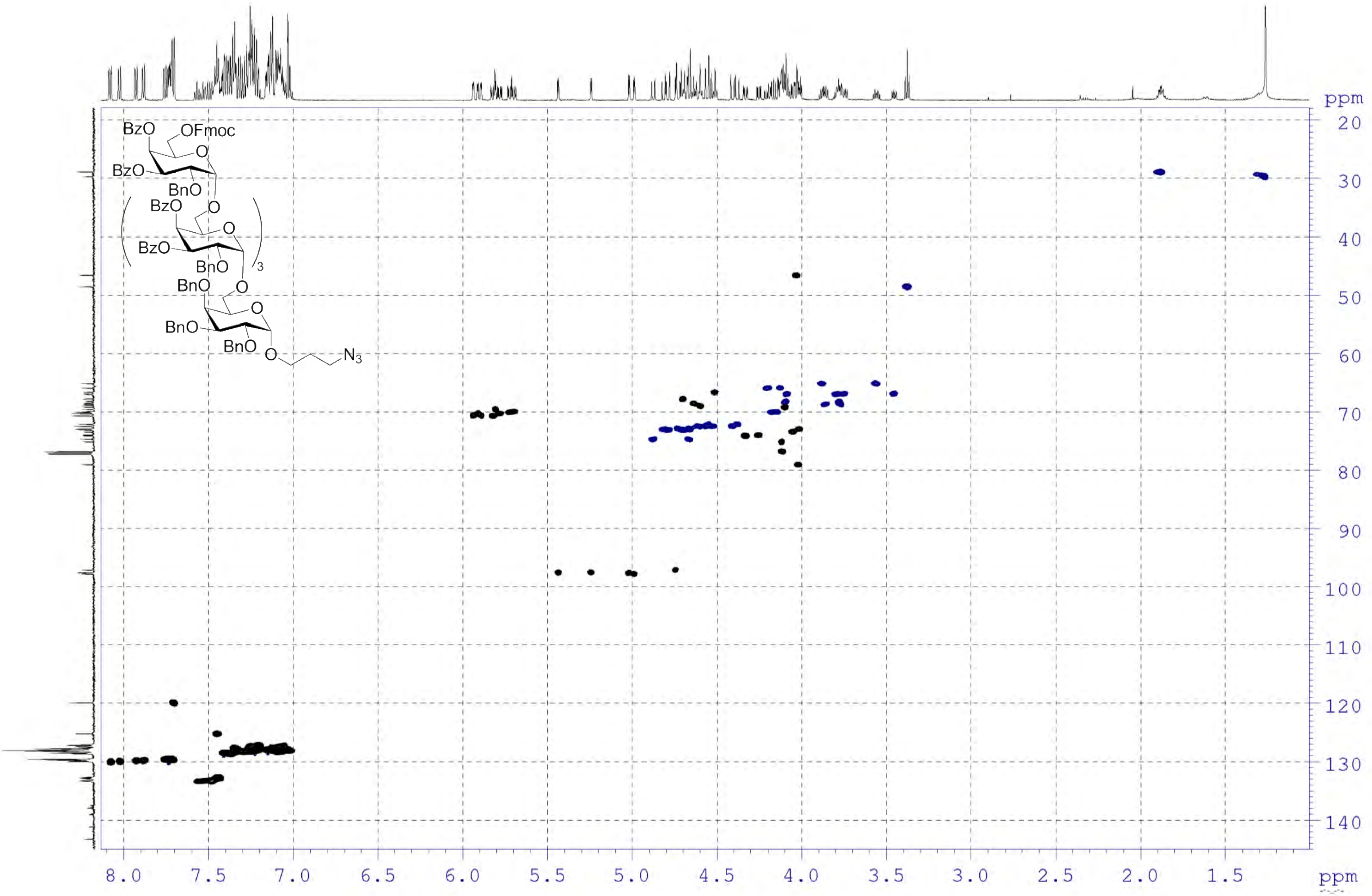

^{1}H-NMR of **S7** (600 MHz, $CDCl_3$)

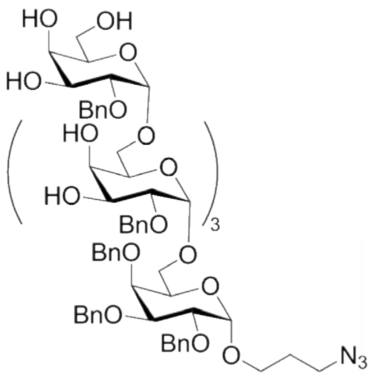

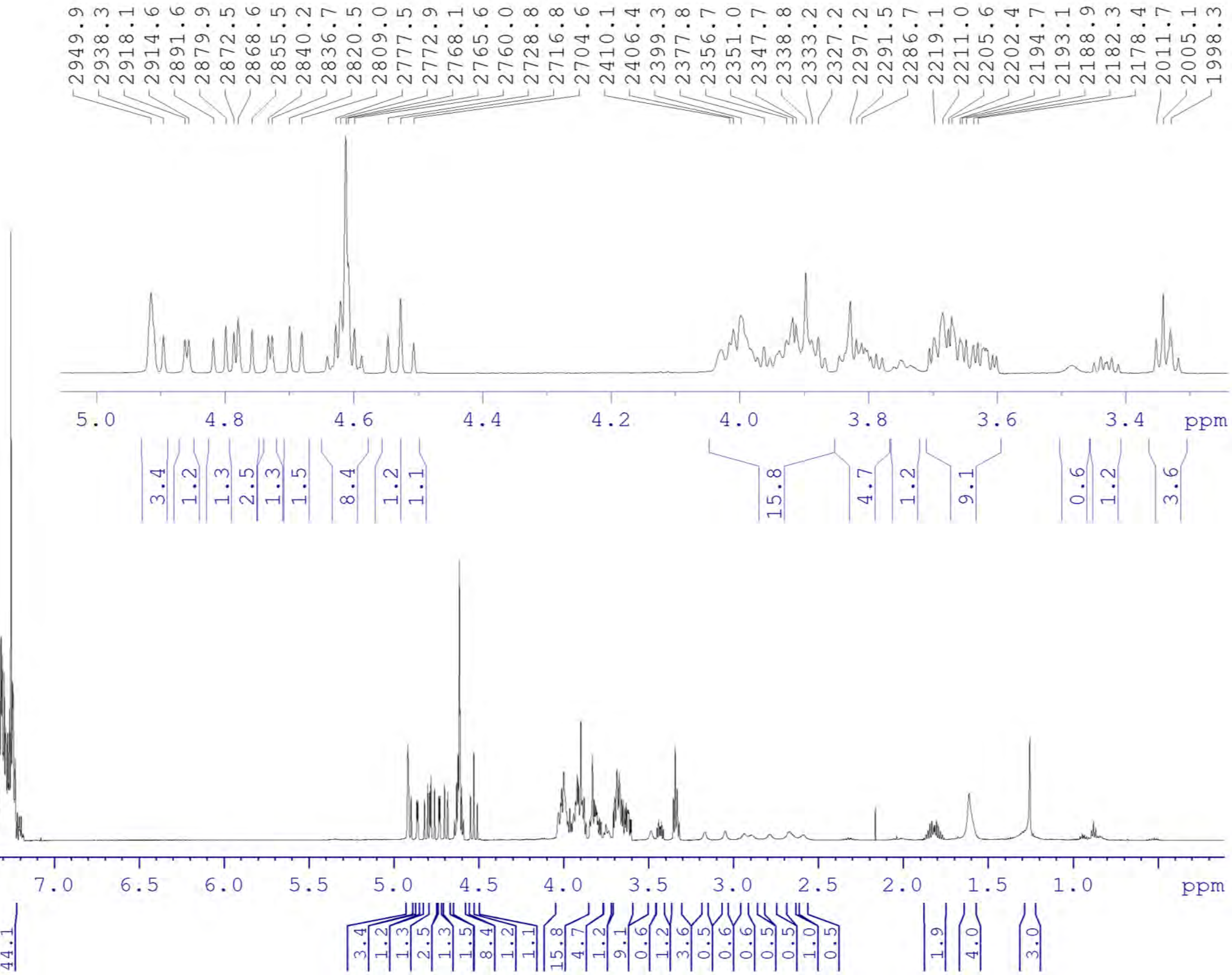

^{13}C-NMR of **S7** (150 MHz, $CDCl_3$)

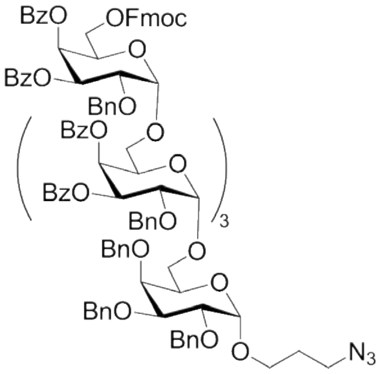

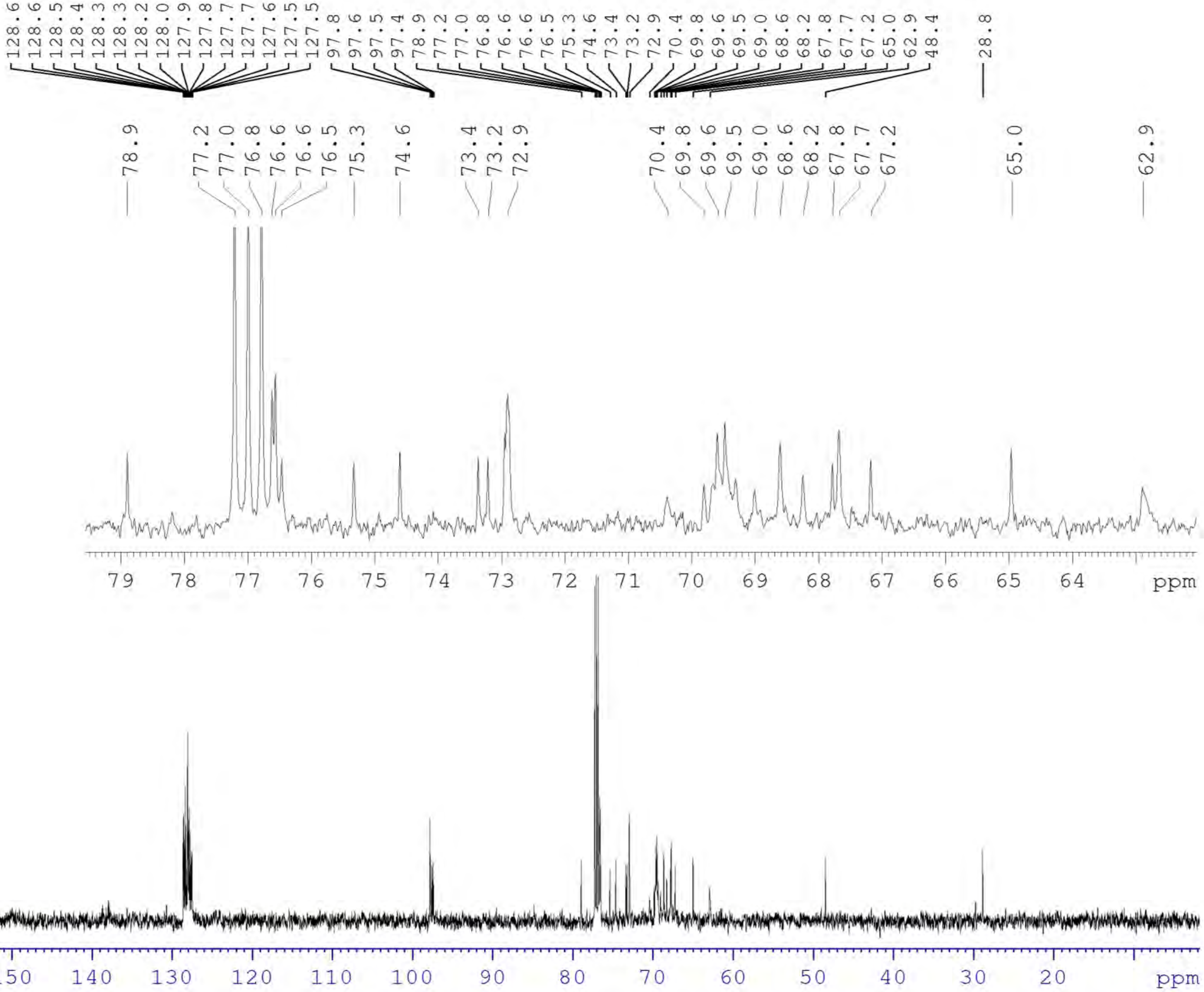

^{1}H-^{1}H COSY of **S7** (600 MHz, $CDCl_3$)

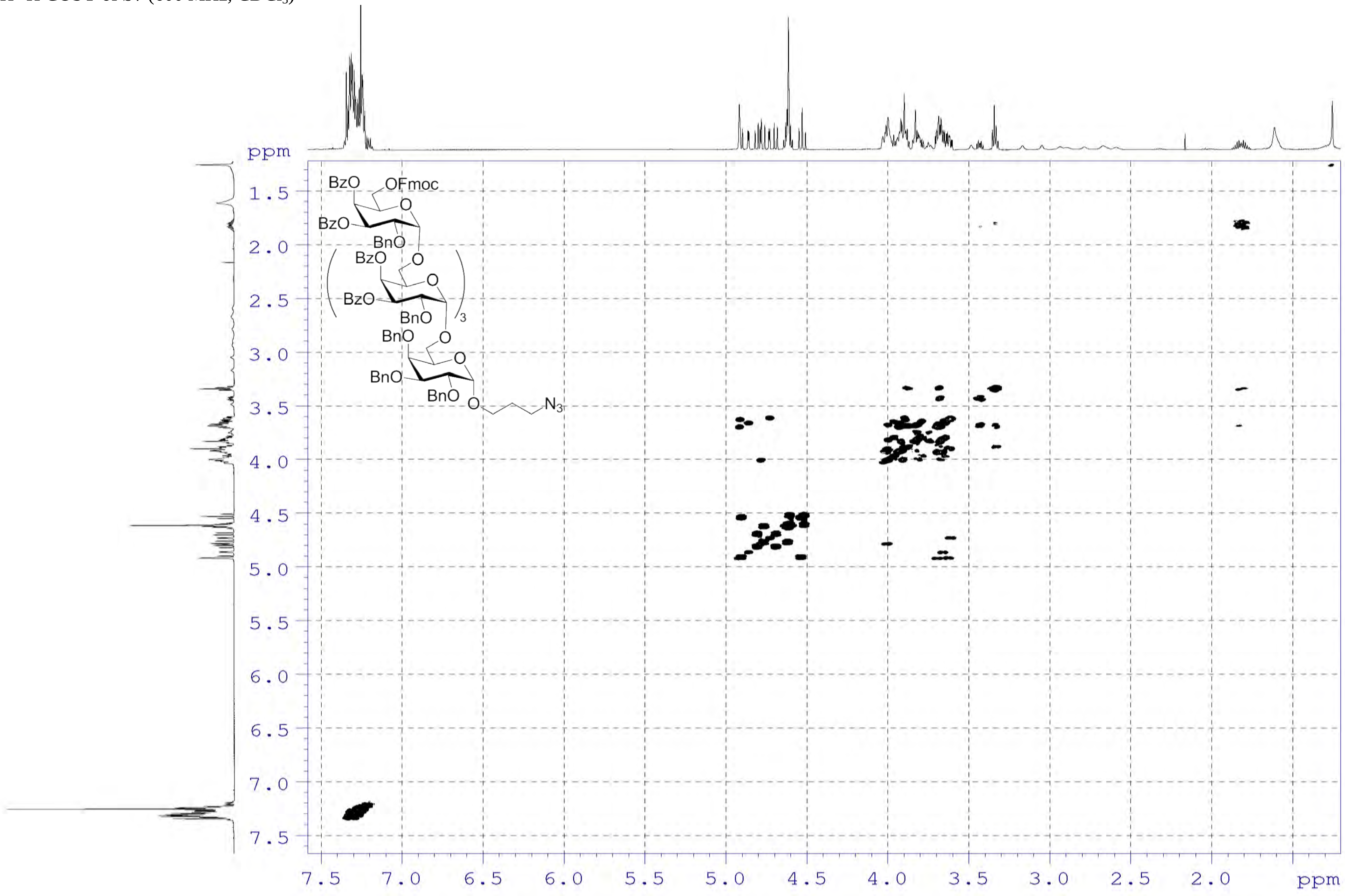

^{1}H-^{13}C HSQC of **S7** (600 MHz, $CDCl_3$)

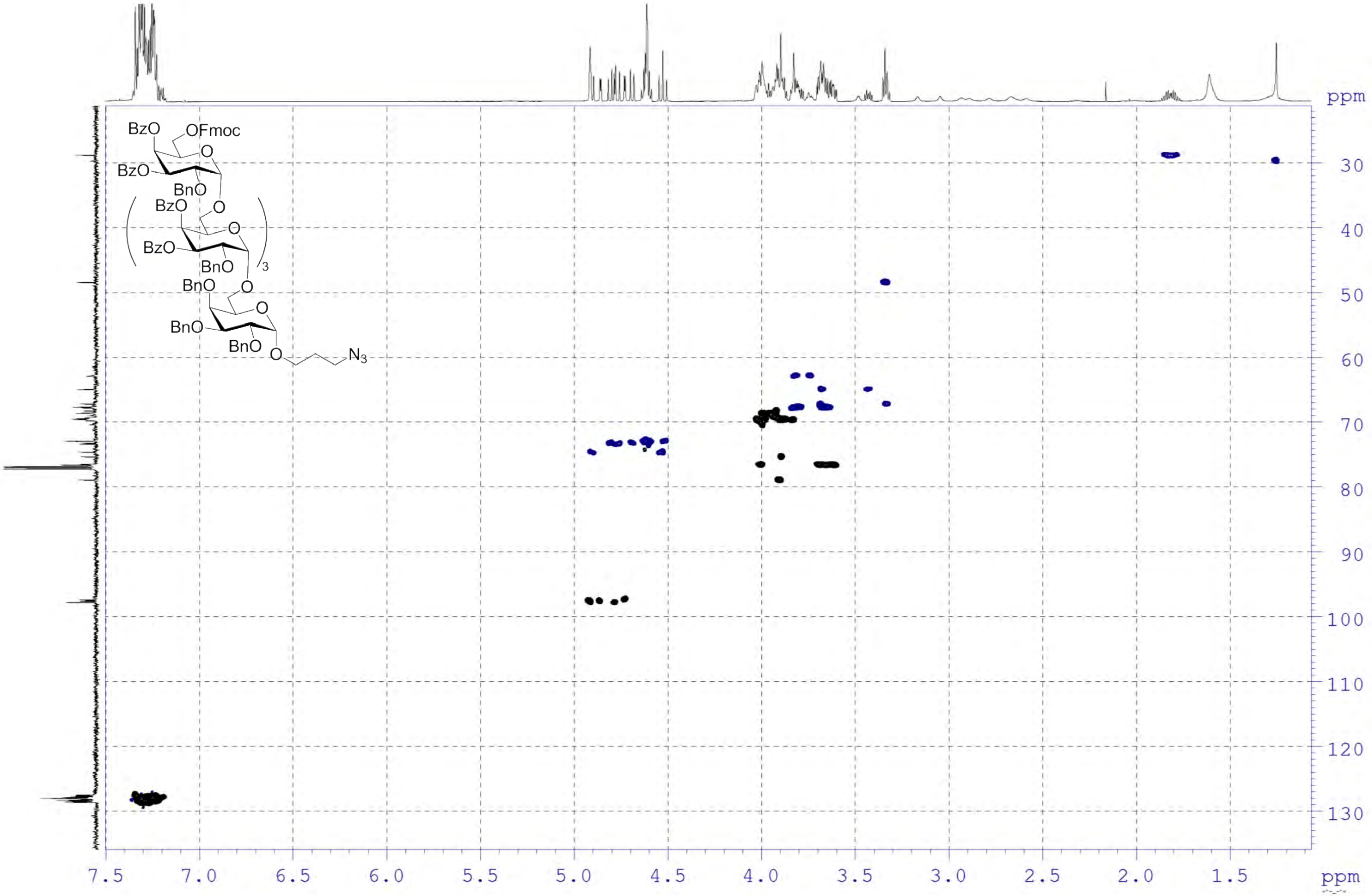

^{1}H-NMR of **4a** (600 MHz, D_2O, 303K)

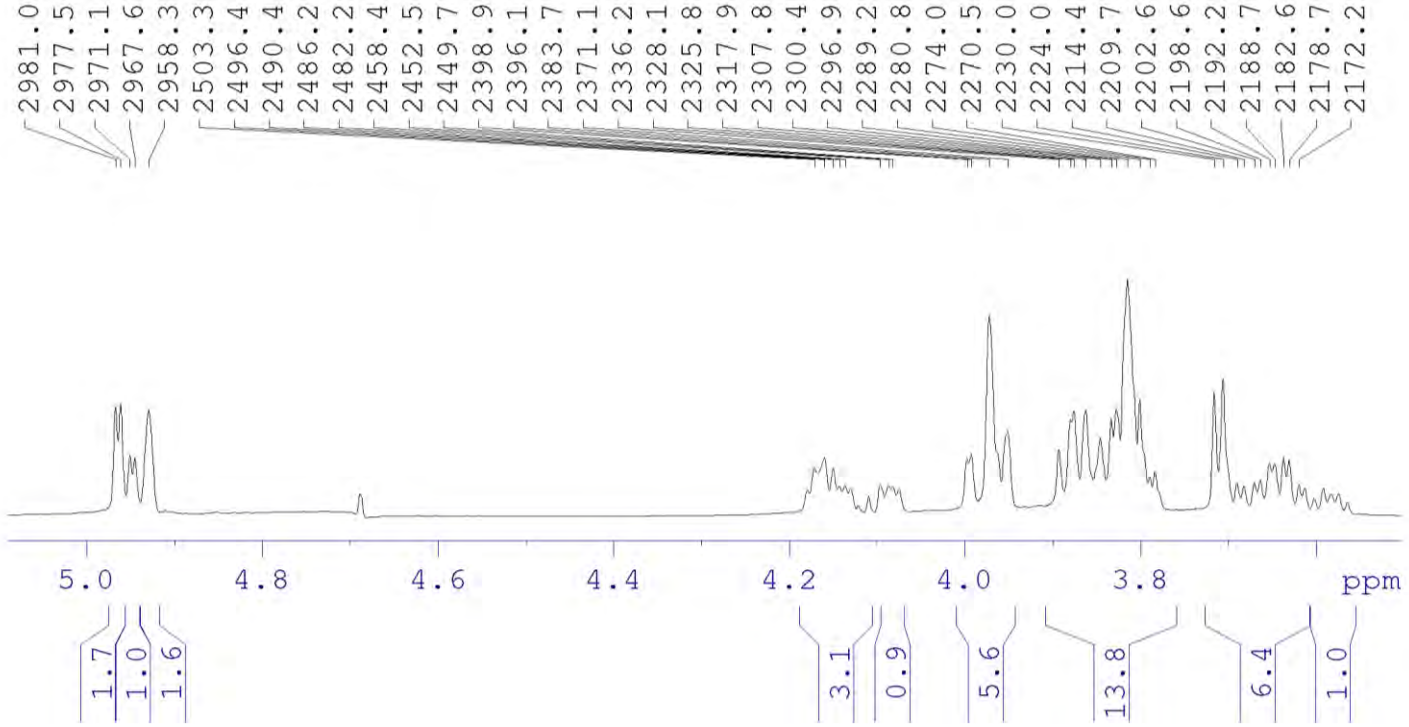

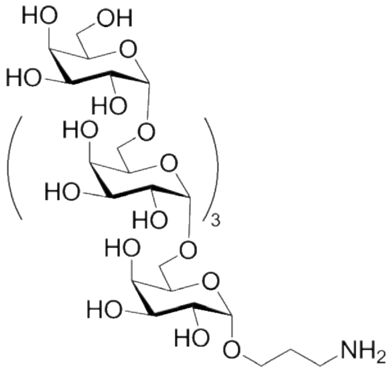

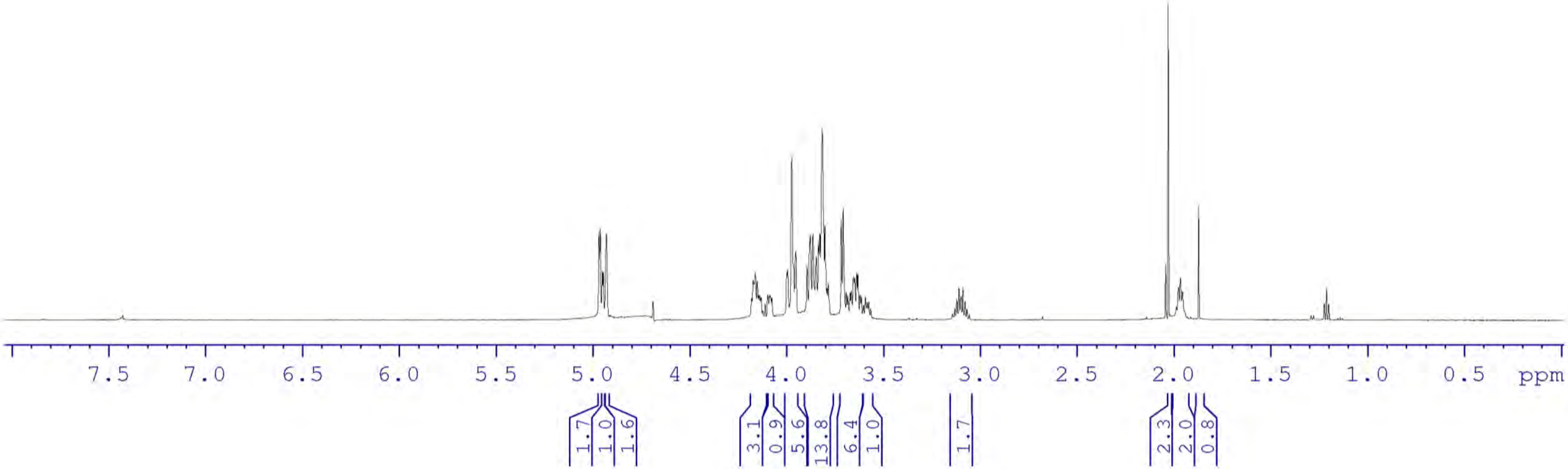

^{13}C-NMR of **4a** (150 MHz, D_2O, 303K)

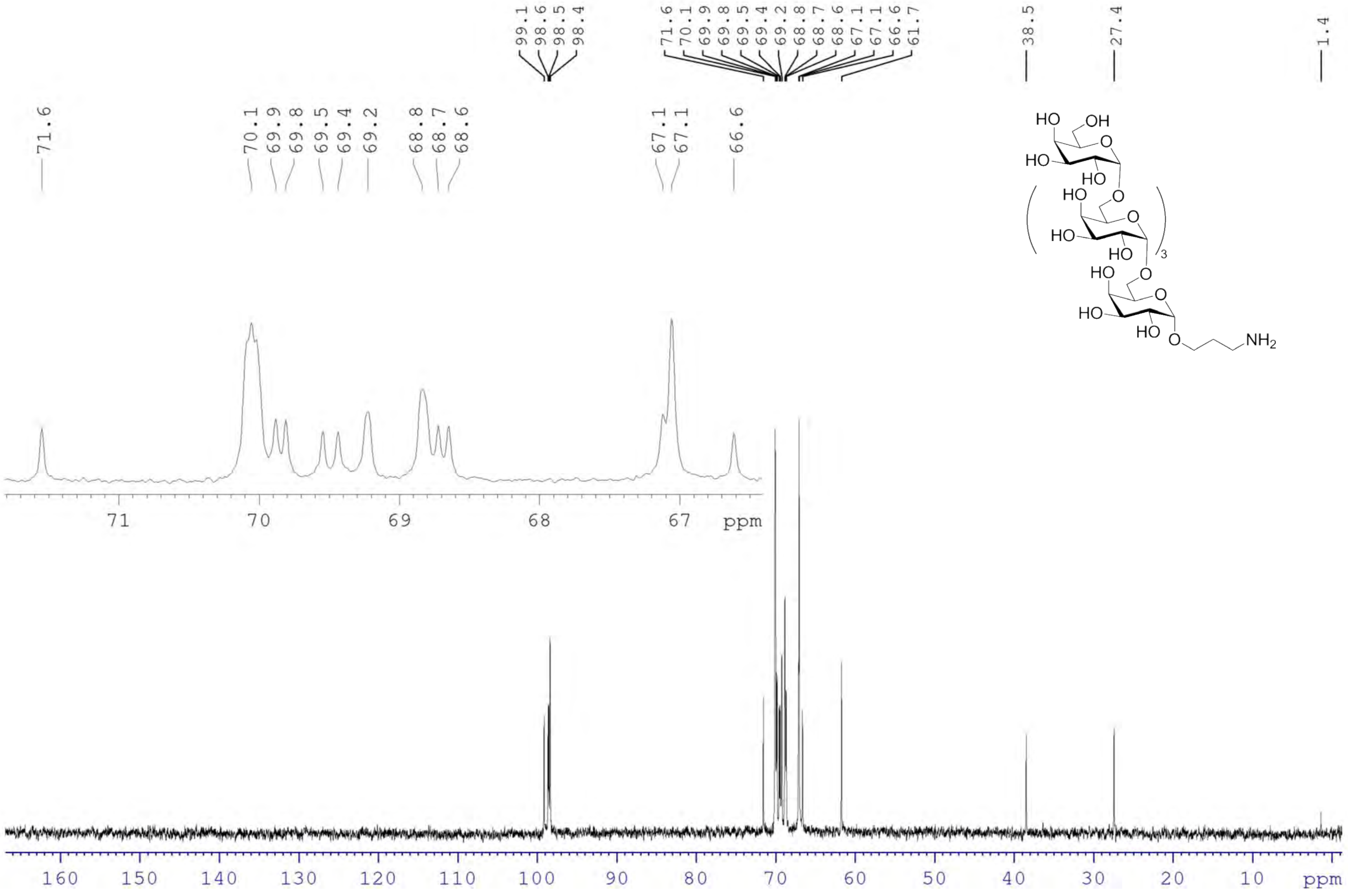

^{1}H-^{1}H COSY of **4a** (600 MHz, D_2O, 303K)

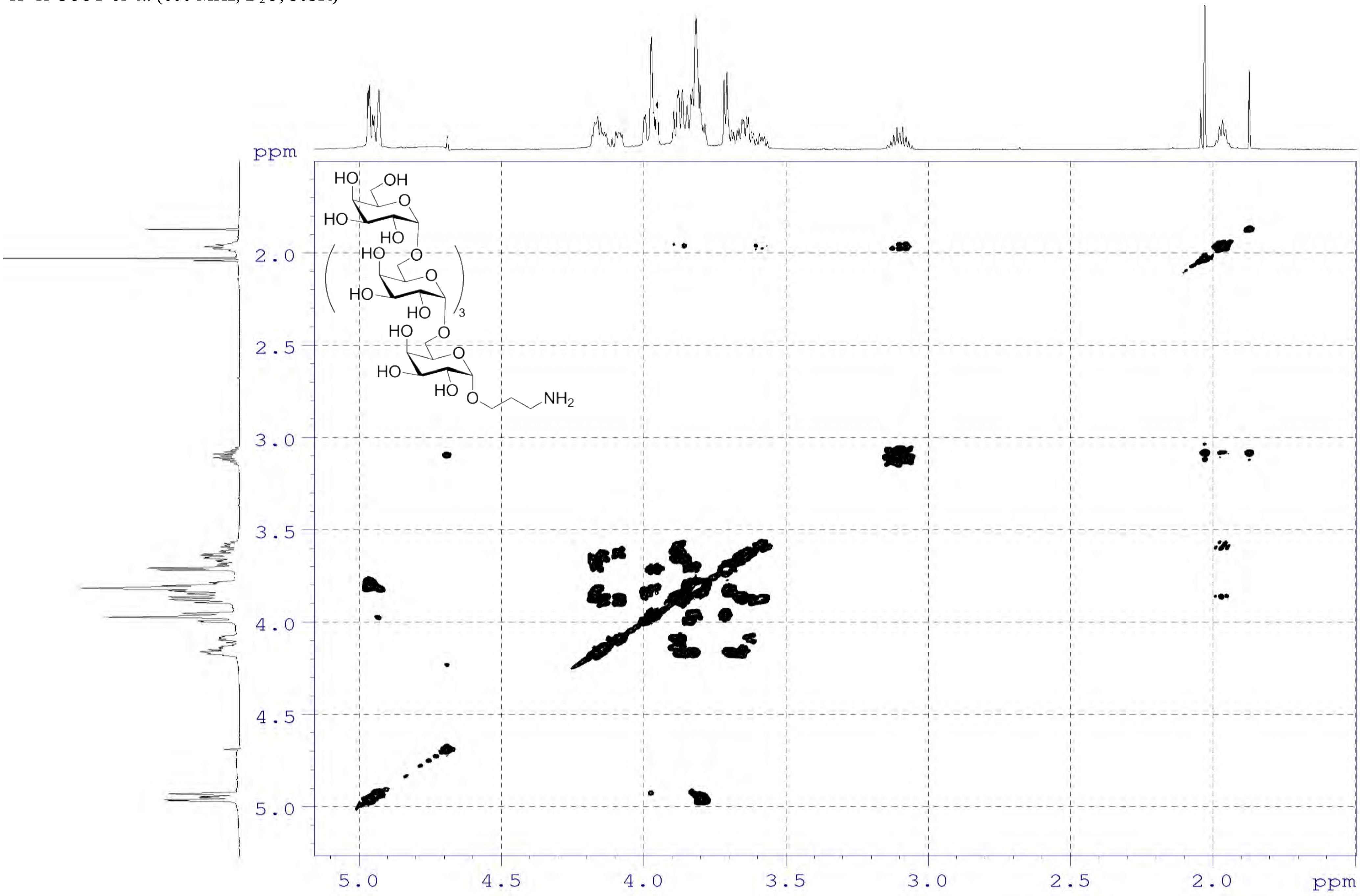

^{1}H-^{13}C HSQC of **4a** (600 MHz, D_2O, 303K)

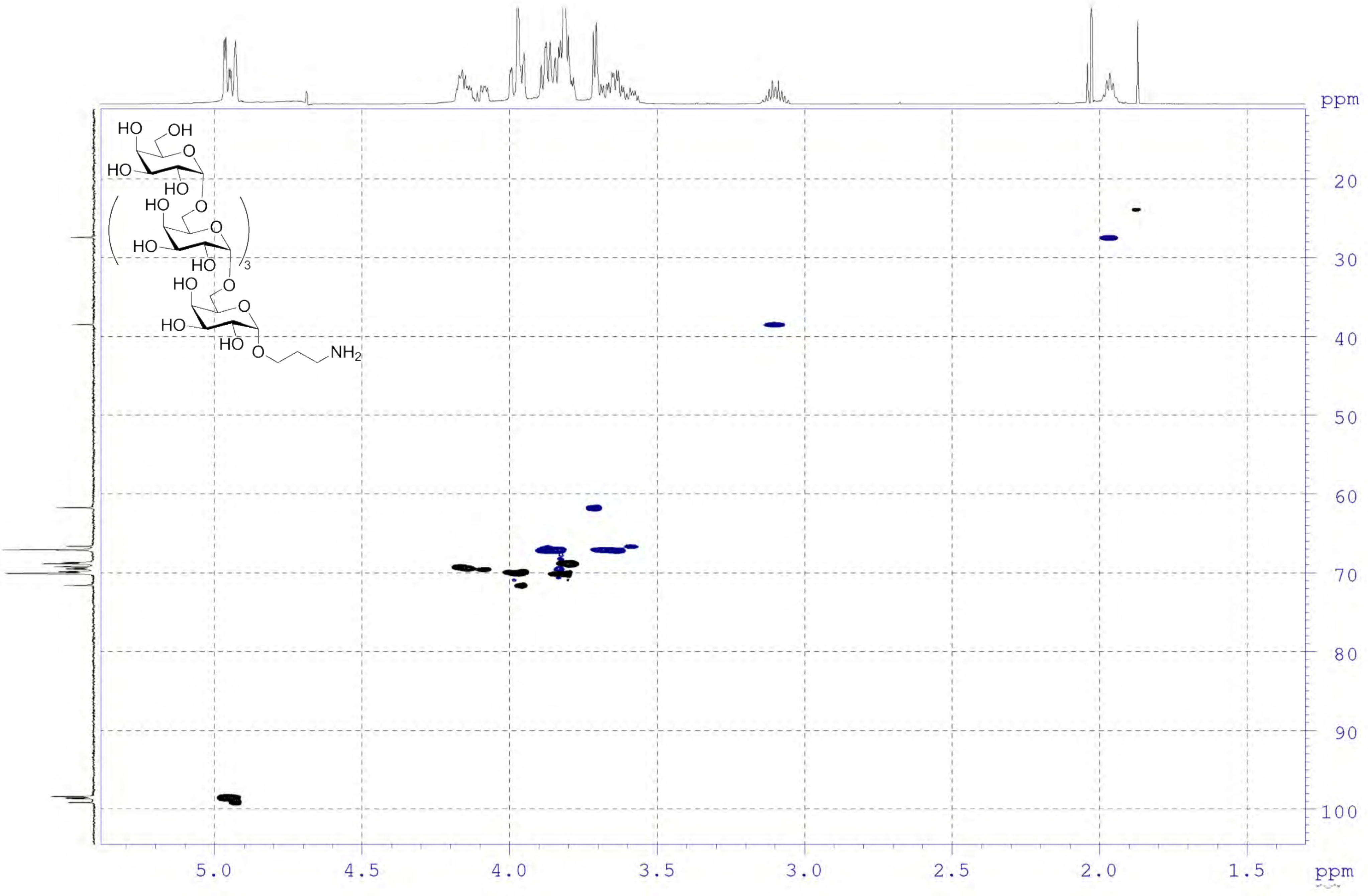

HRMS for **4a**

			Instrument / Ser#	micrOTOF	10248
Comment	CH3CN : H2O 50/50 %, dil. 2000, calibrant added				

Acquisition Parameter

Source Type	ESI	Ion Polarity	Positive	Set Nebulizer	0.4 Bar
Focus	Not active			Set Dry Heater	180 ℃
Scan Begin	50 m/z	Set Capillary	4500 V	Set Dry Gas	4.0 l/min
Scan End	3000 m/z	Set End Plate Offset	-500 V	Set Divert Valve	Waste

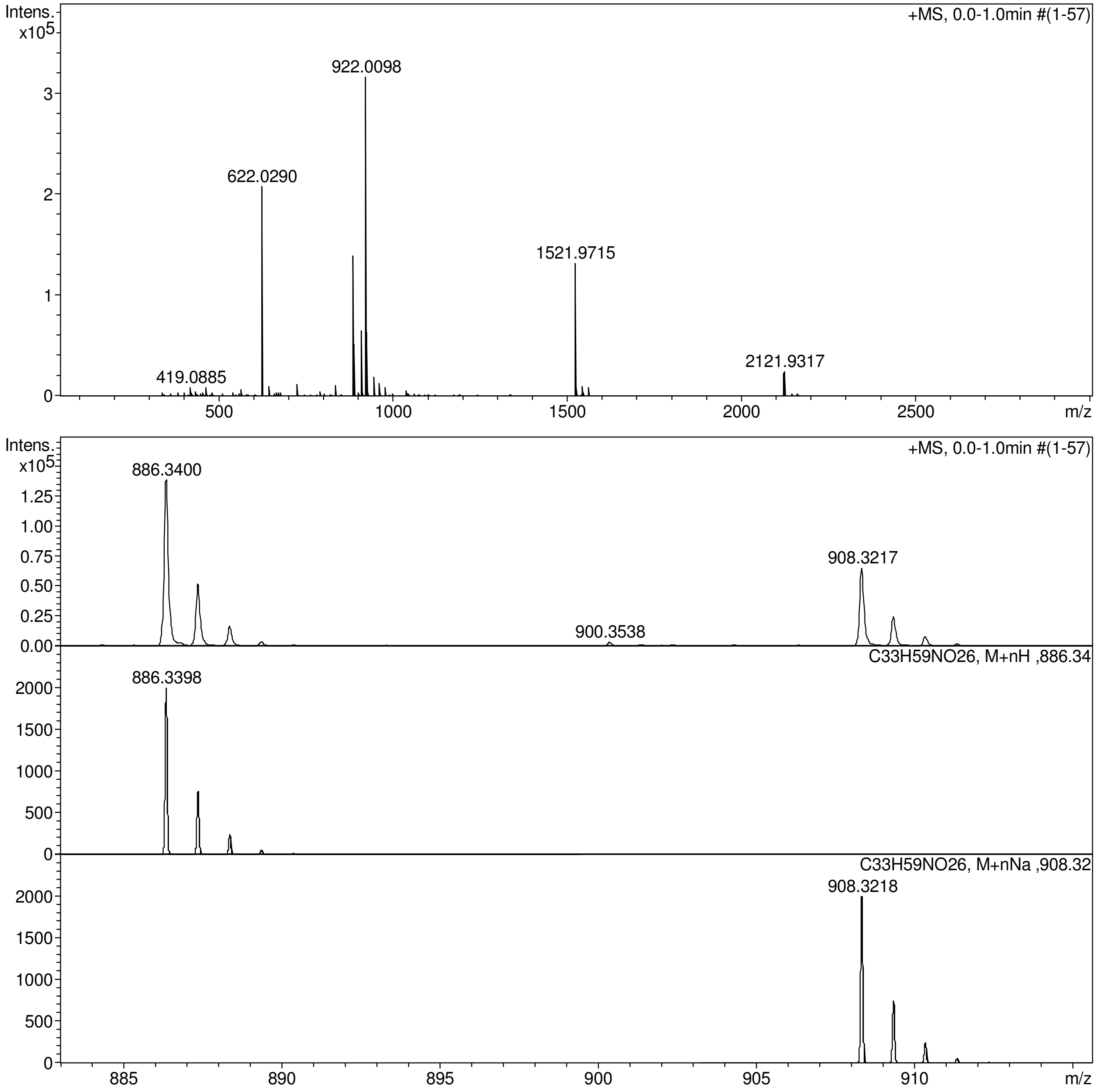

^{1}H-NMR of **4b** (600 MHz, D_2O, 303K)

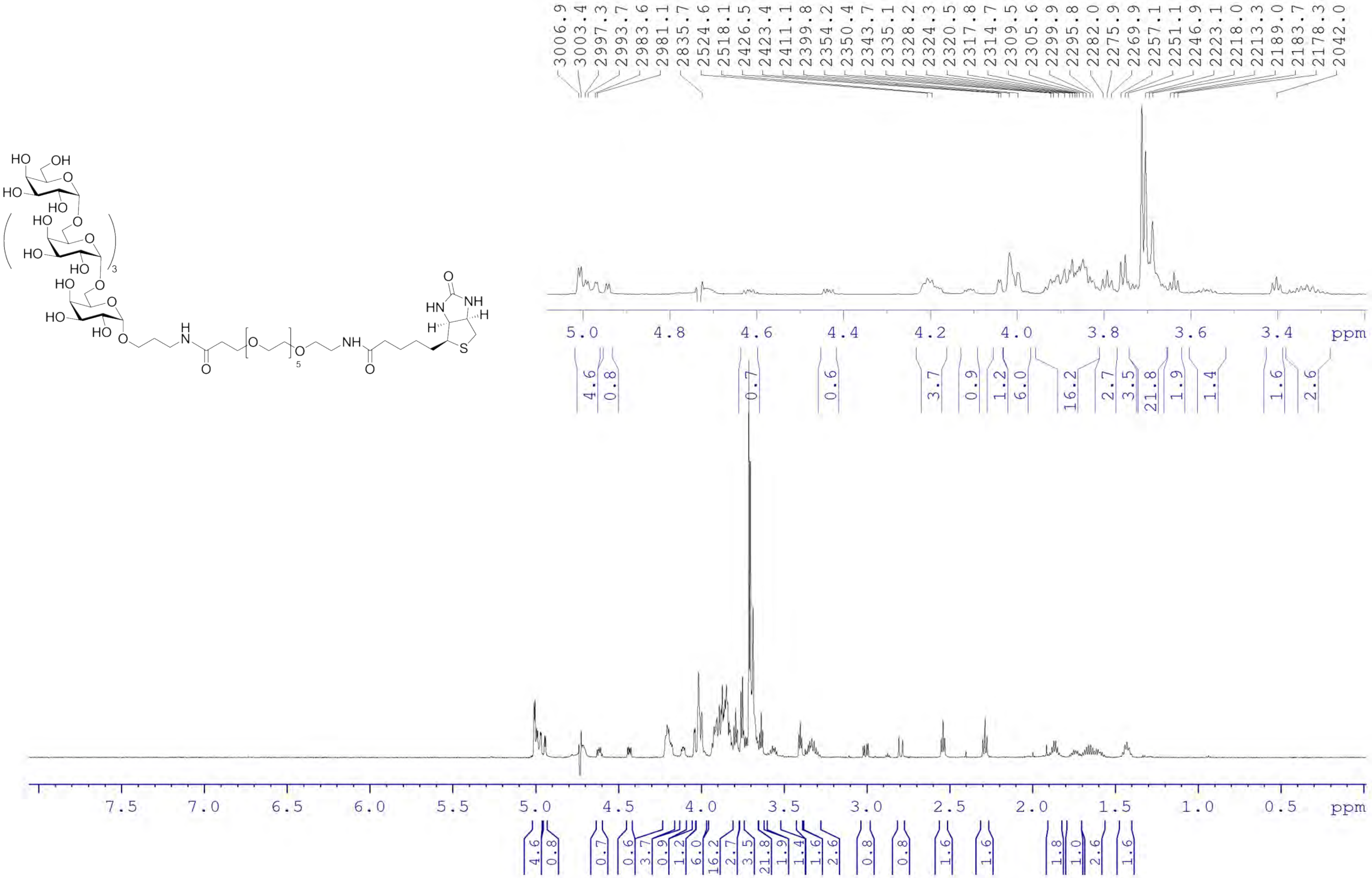

^{13}C-NMR of **4b** (150 MHz, D_2O, 303K)

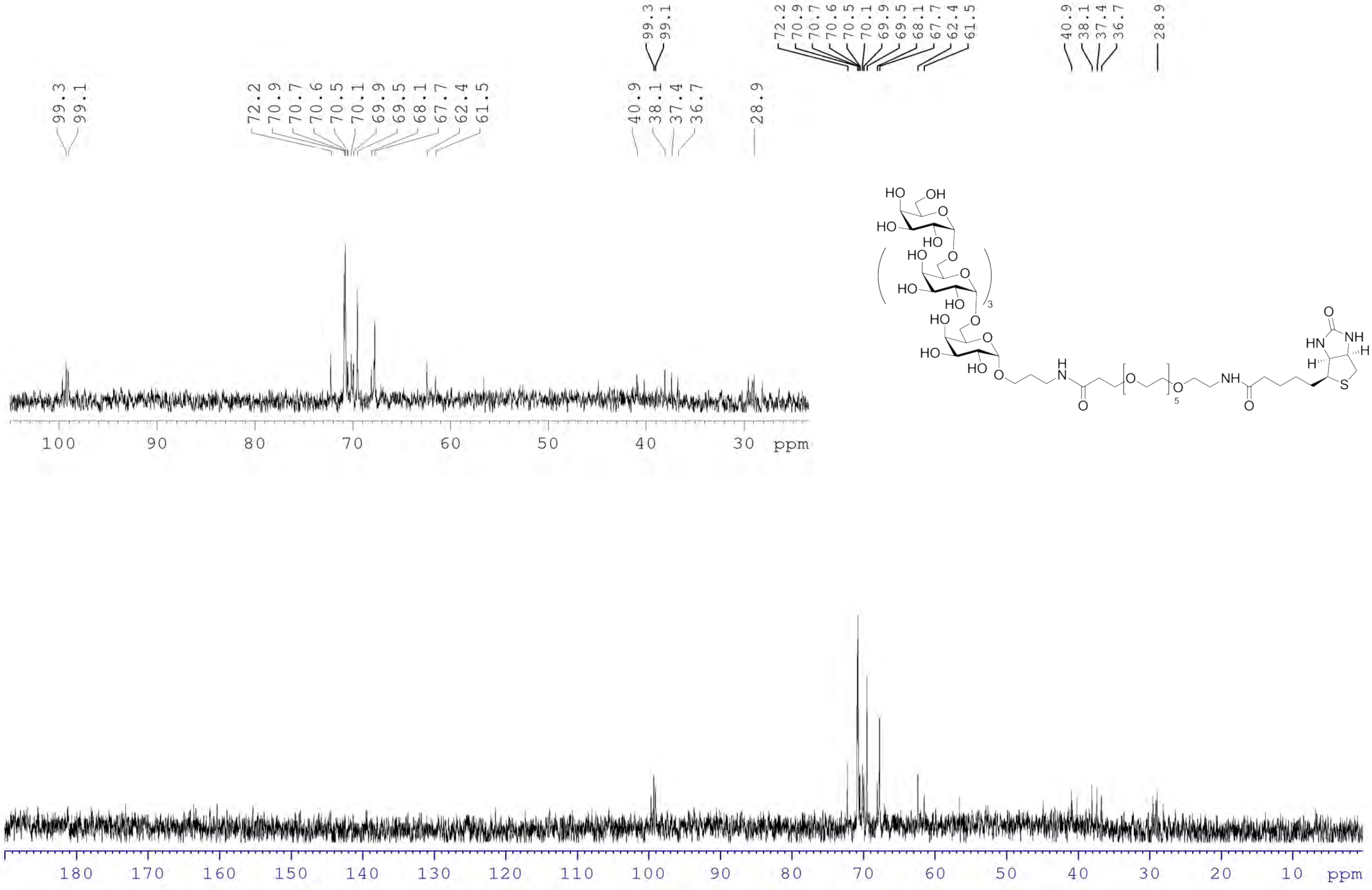

HRMS for **4b**

Comment CH3CN : H2O 50/50 %, dil. 200, calibrant addded

Acquisition Parameter

Source Type	ESI	Ion Polarity	Positive	Set Nebulizer	0.5 Bar
Focus	Active			Set Dry Heater	180 ℃
Scan Begin	50 m/z	Set Capillary	4500 V	Set Dry Gas	4.0 l/min
Scan End	3000 m/z	Set End Plate Offset	-500 V	Set Divert Valve	Waste

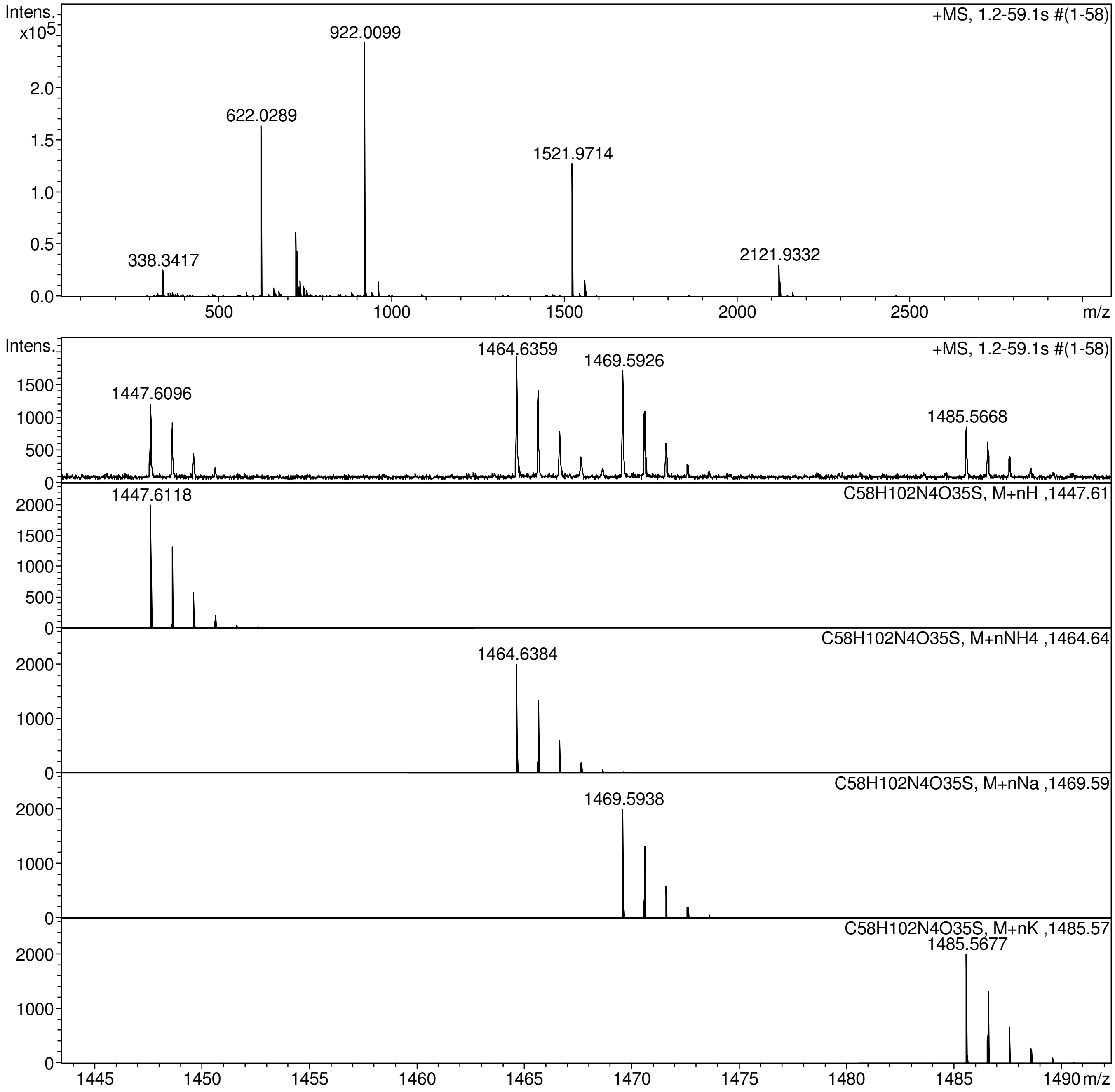

^{1}H-NMR of **38** (300 MHz, $CDCl_3$)

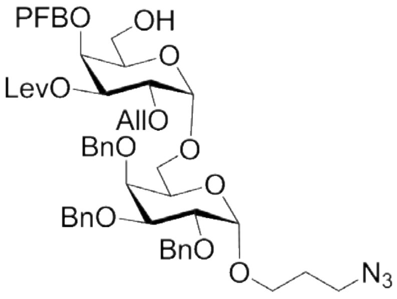

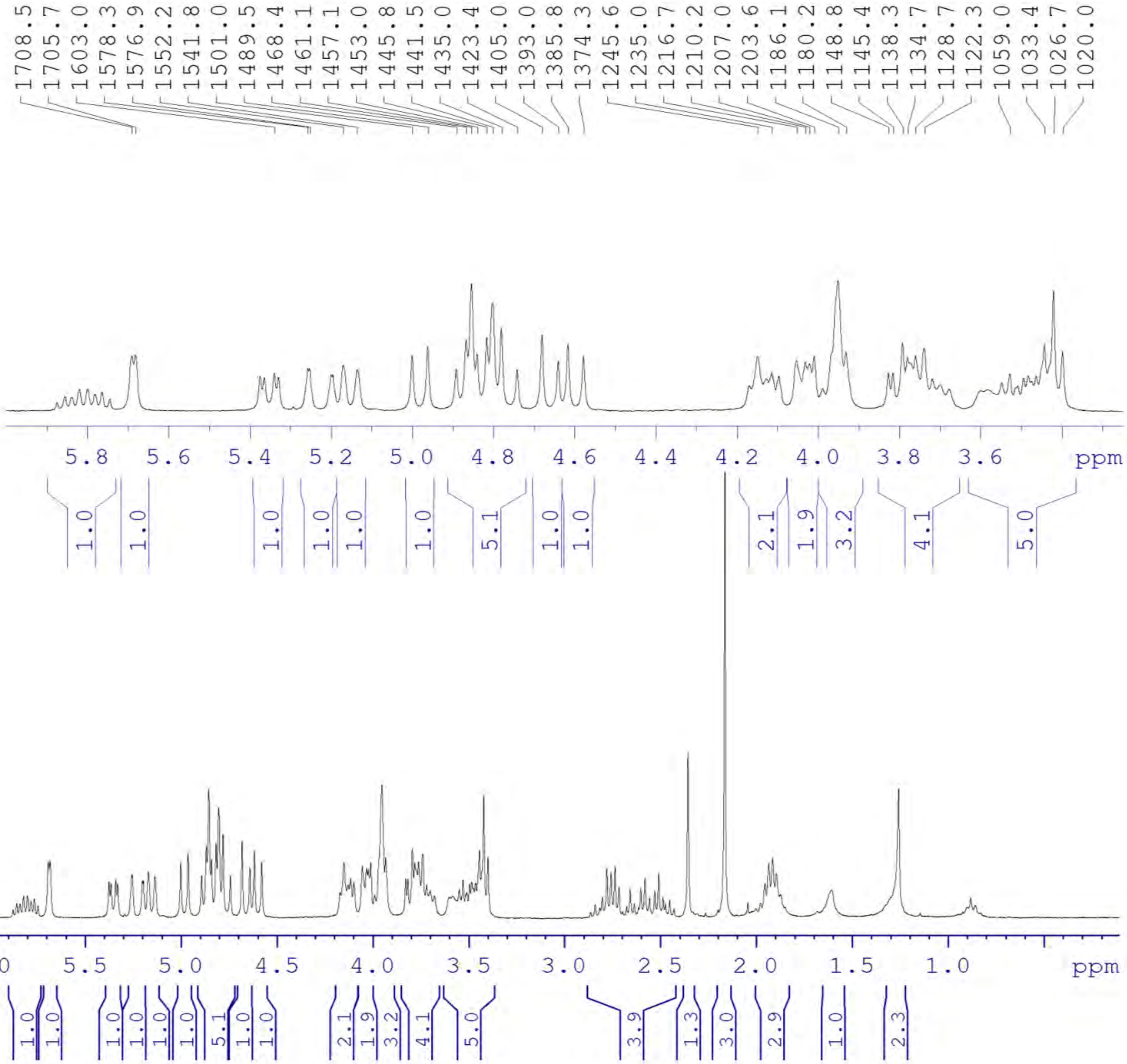

^{13}C-NMR of **38** (75 MHz, $CDCl_3$)

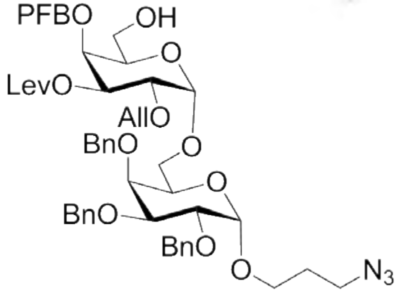

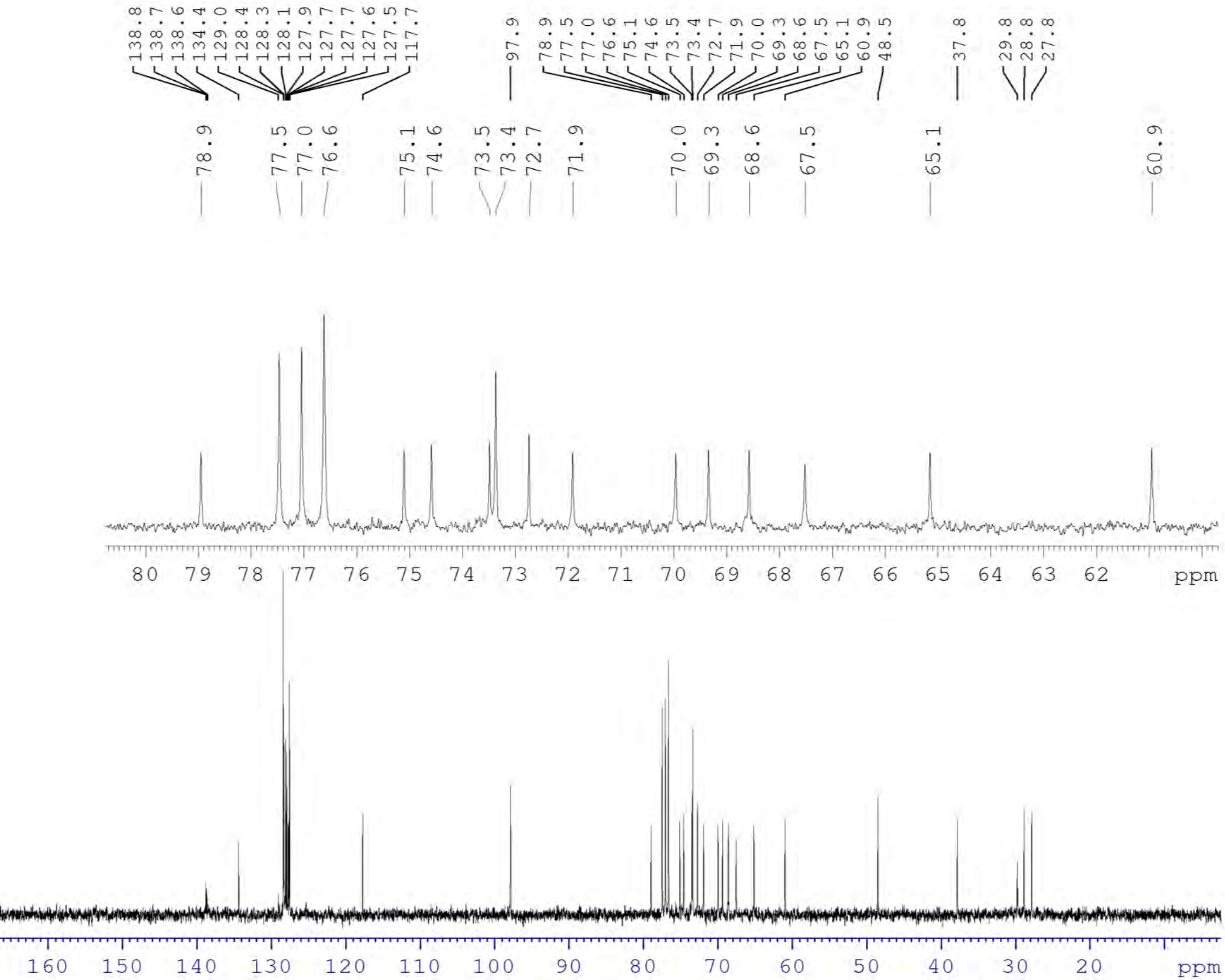

^{1}H-^{1}H COSY of **38** (300 MHz, $CDCl_3$)

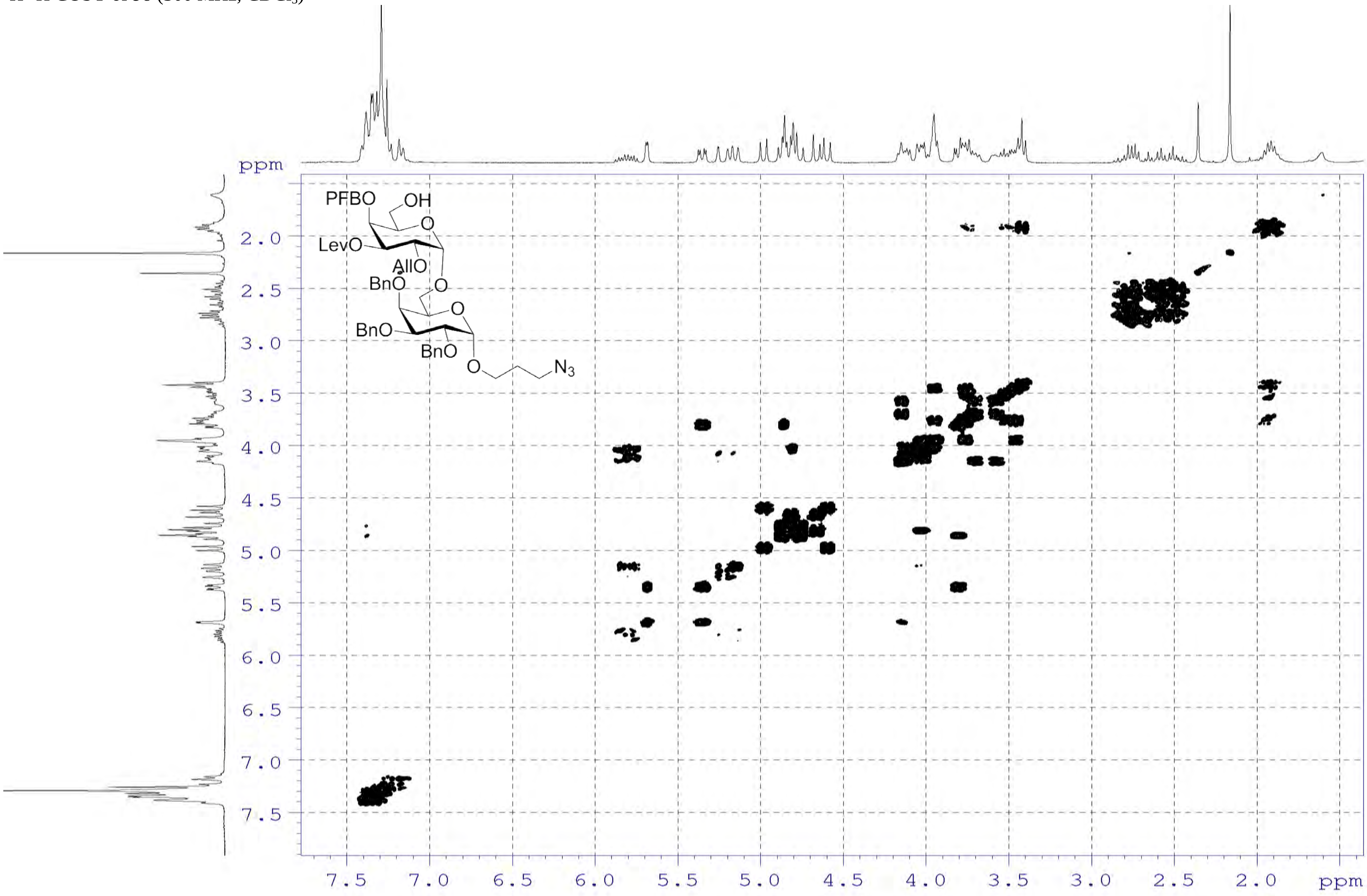

^{1}H-^{13}C HSQC of **38** (300 MHz, $CDCl_3$)

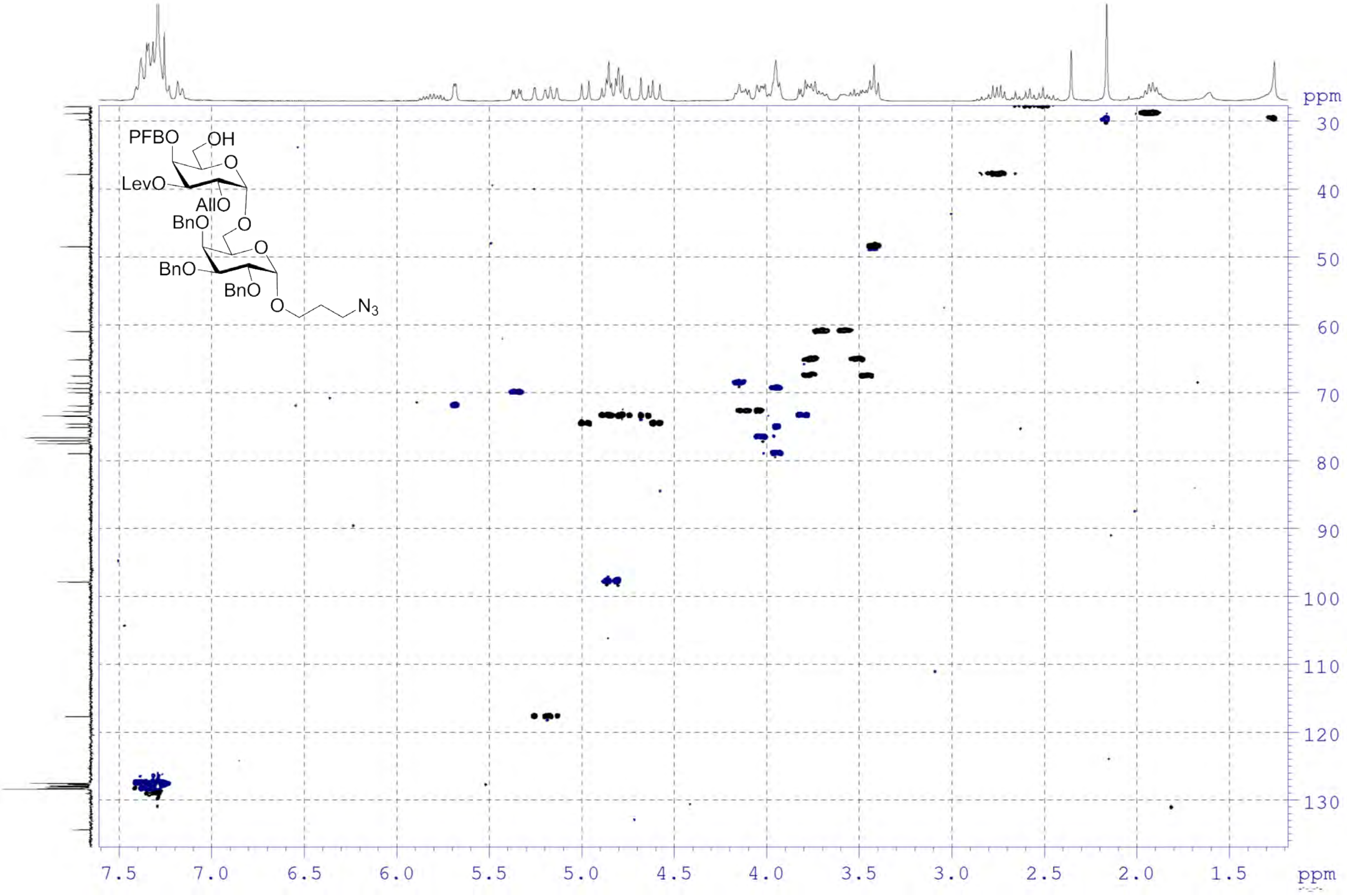

^{1}H-NMR of **39a** (600 MHz, $CDCl_3$)

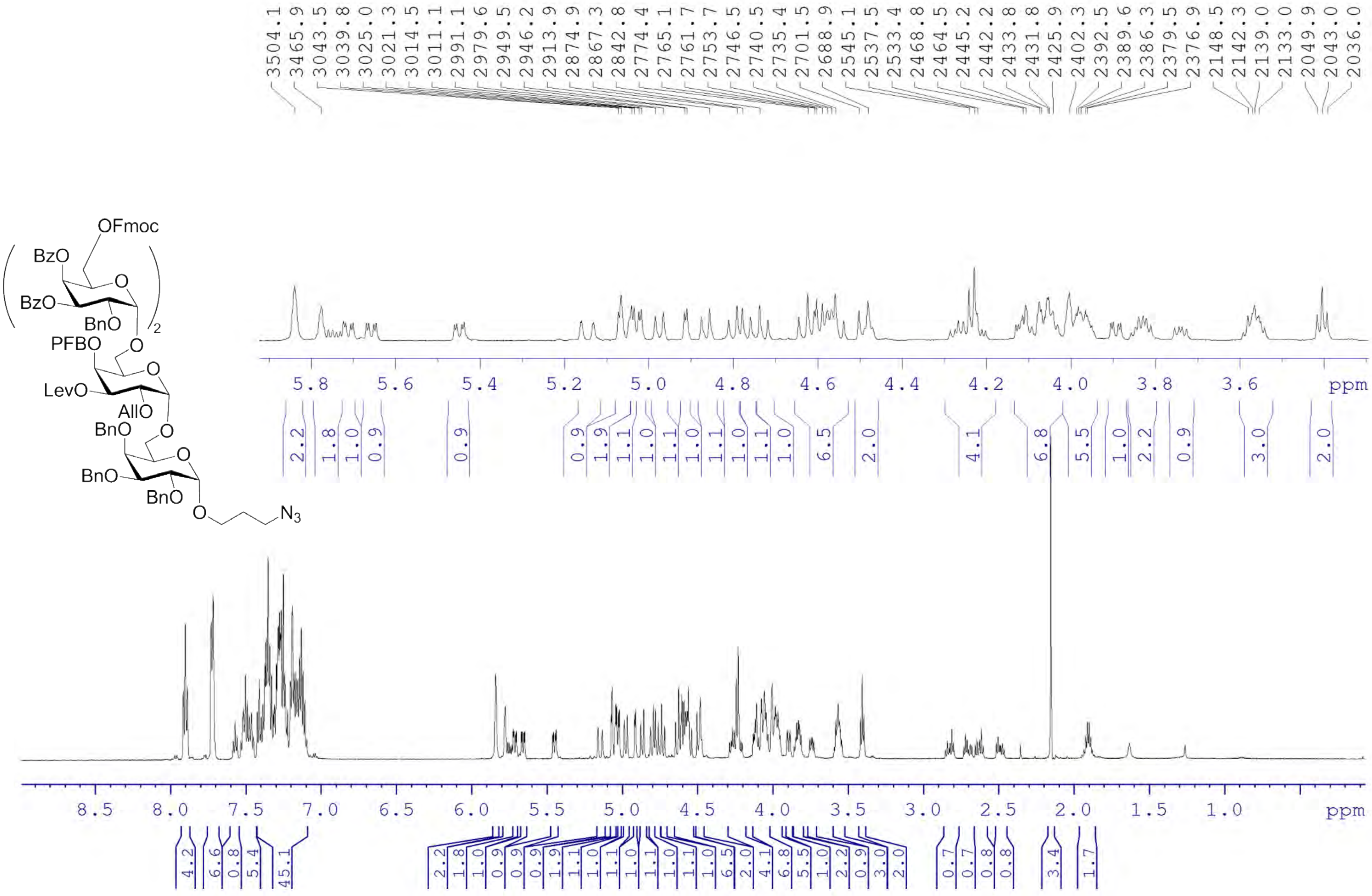

^{13}C-NMR of **39a** (150 MHz, $CDCl_3$)

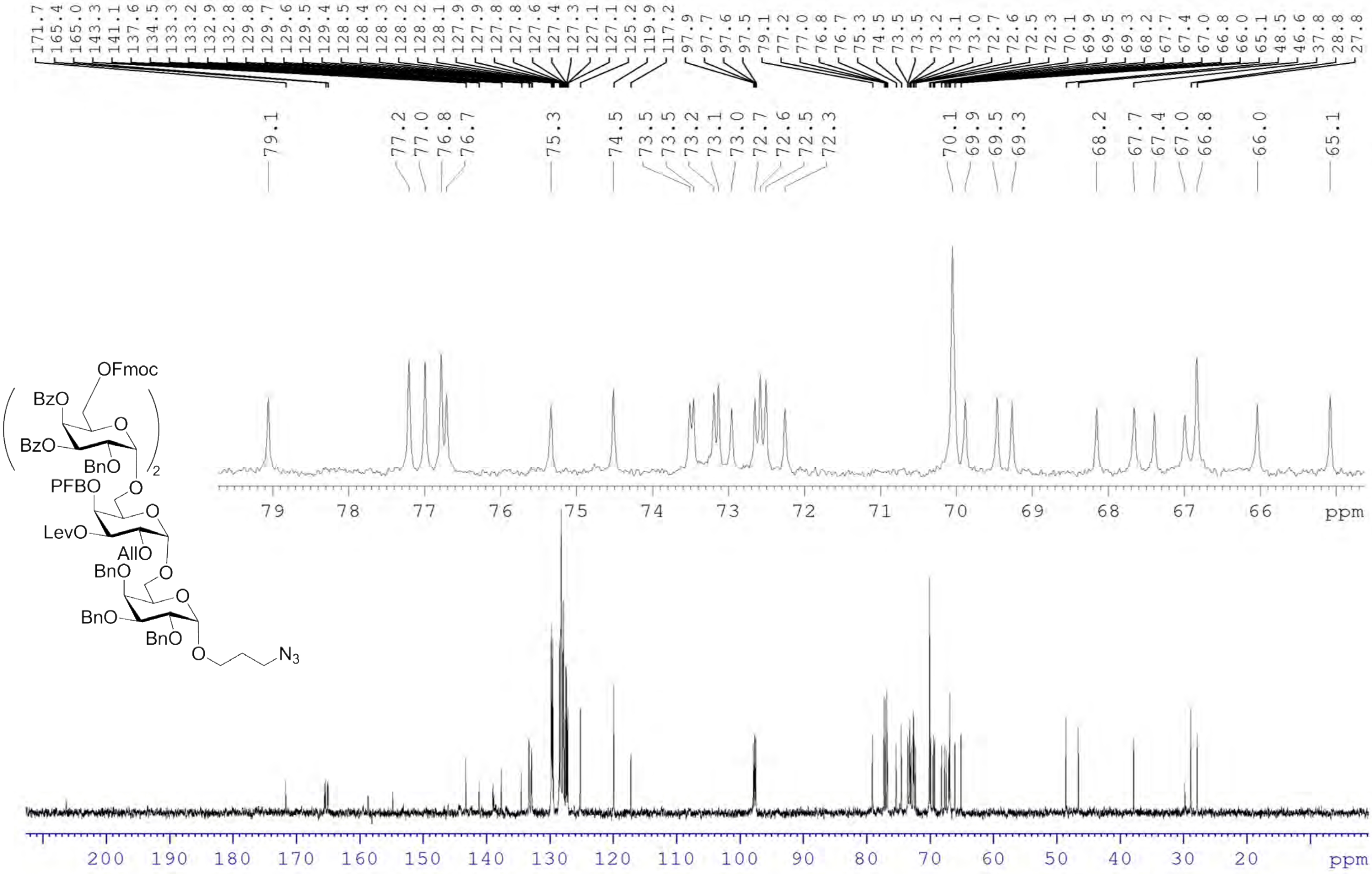

^{1}H-^{1}H COSY of **39a** (600 MHz, $CDCl_3$)

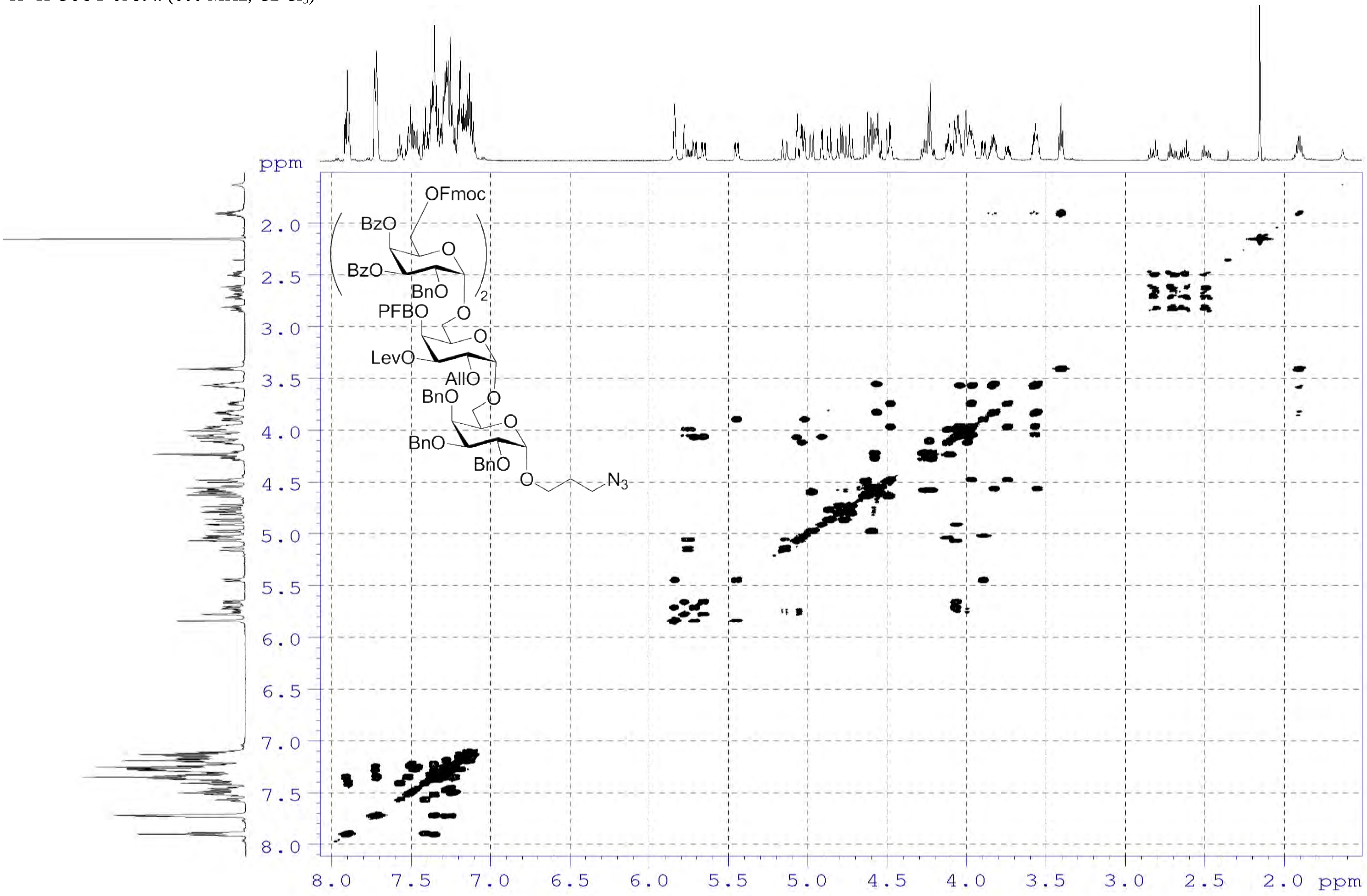

^{1}H-^{13}C HSQC of **39a** (600 MHz, $CDCl_3$)

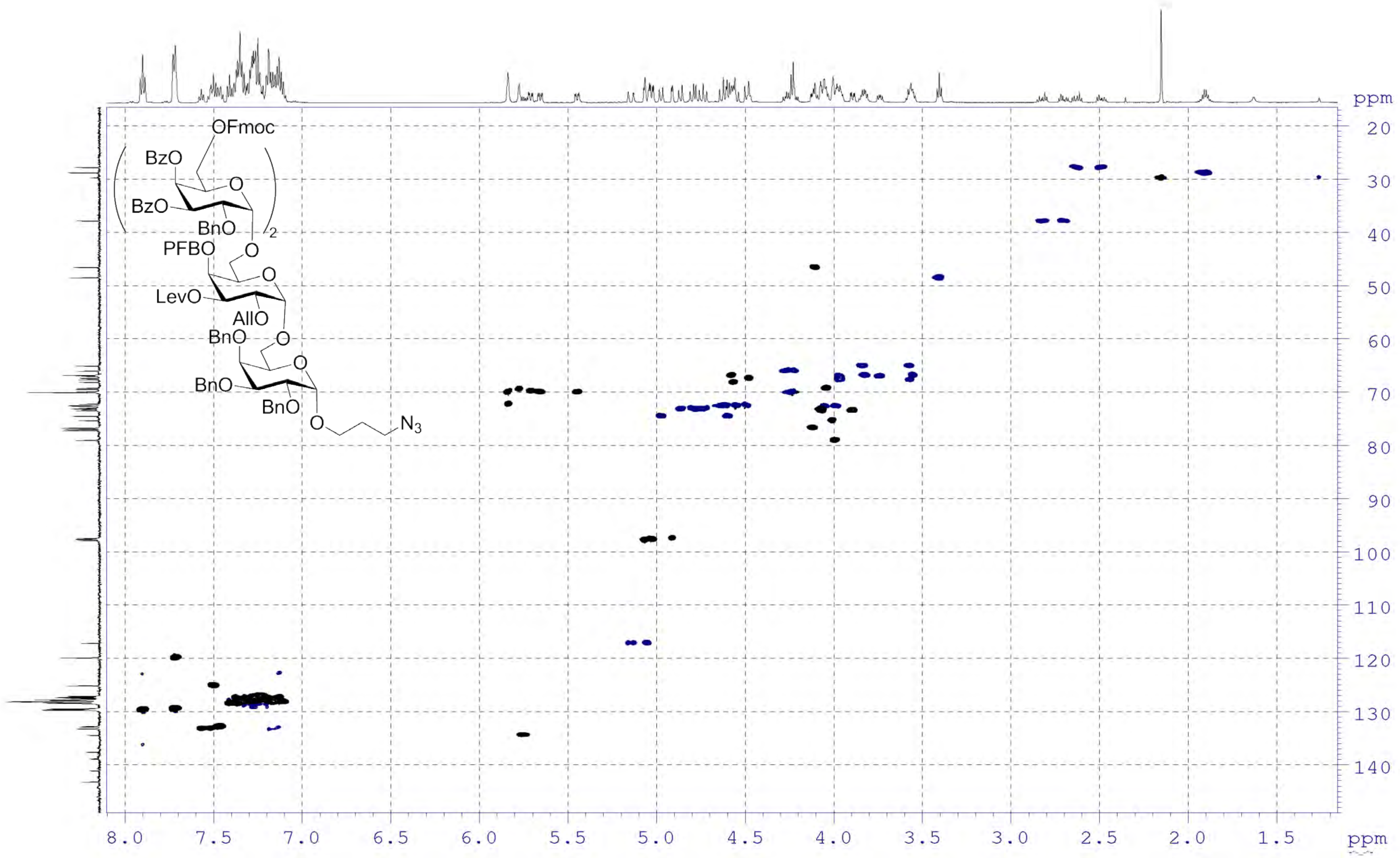

^{1}H-NMR of **39b** (600 MHz, $CDCl_3$)

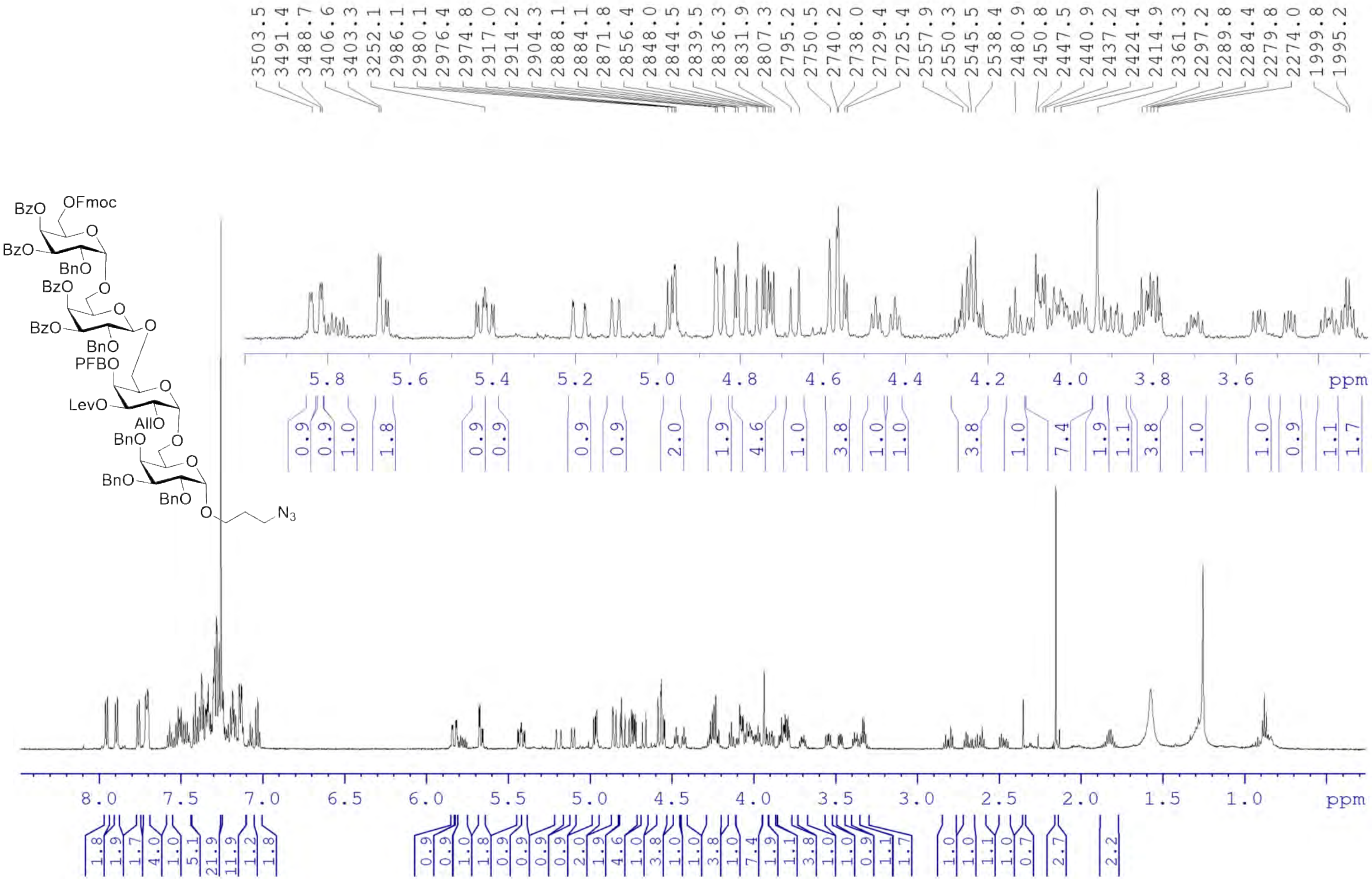

^{13}C-NMR of **39b** (150 MHz, $CDCl_3$)

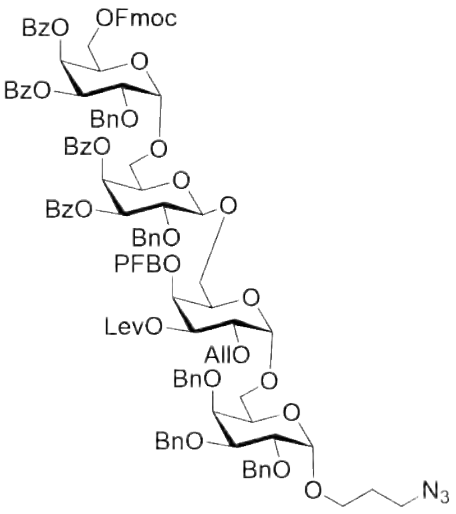

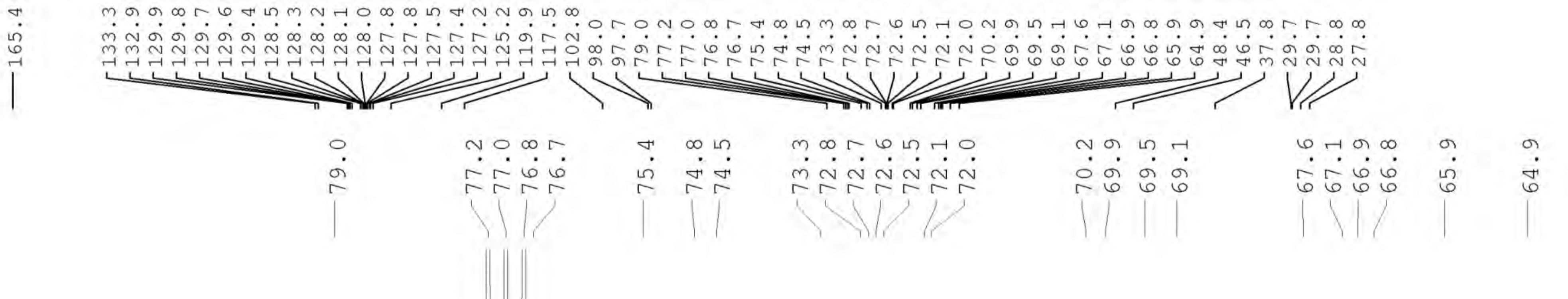

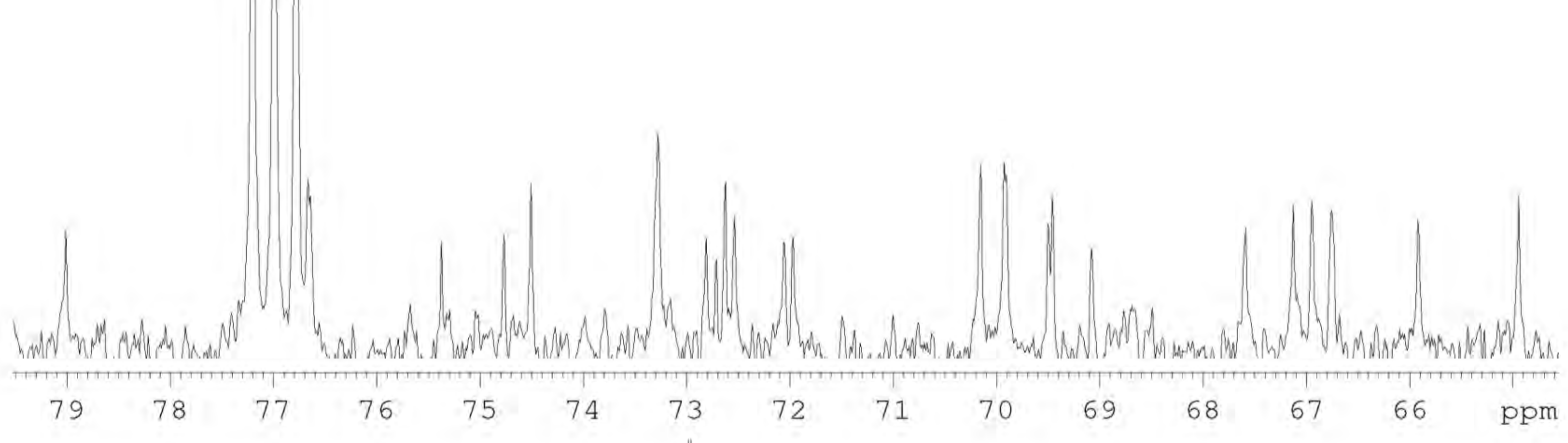

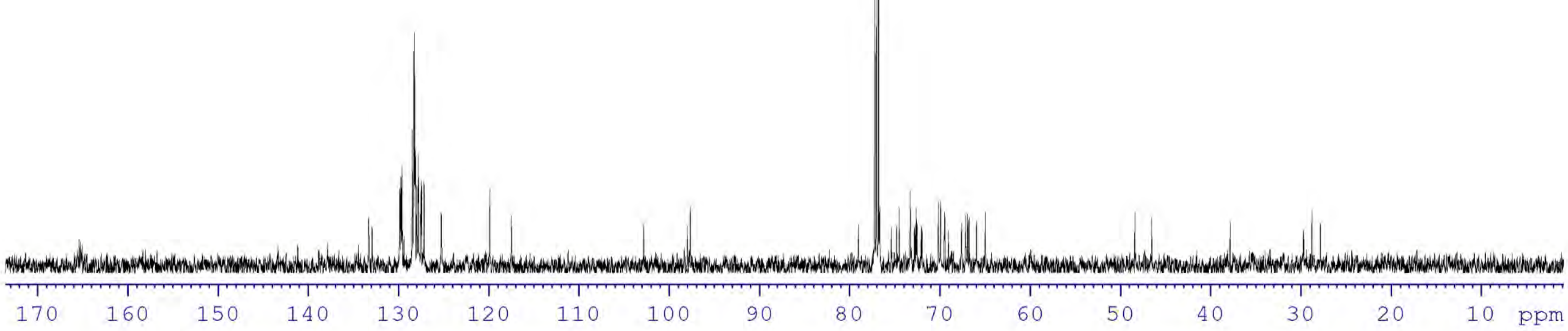

^{1}H-^{1}H COSY **39b** (600 MHz, $CDCl_3$)

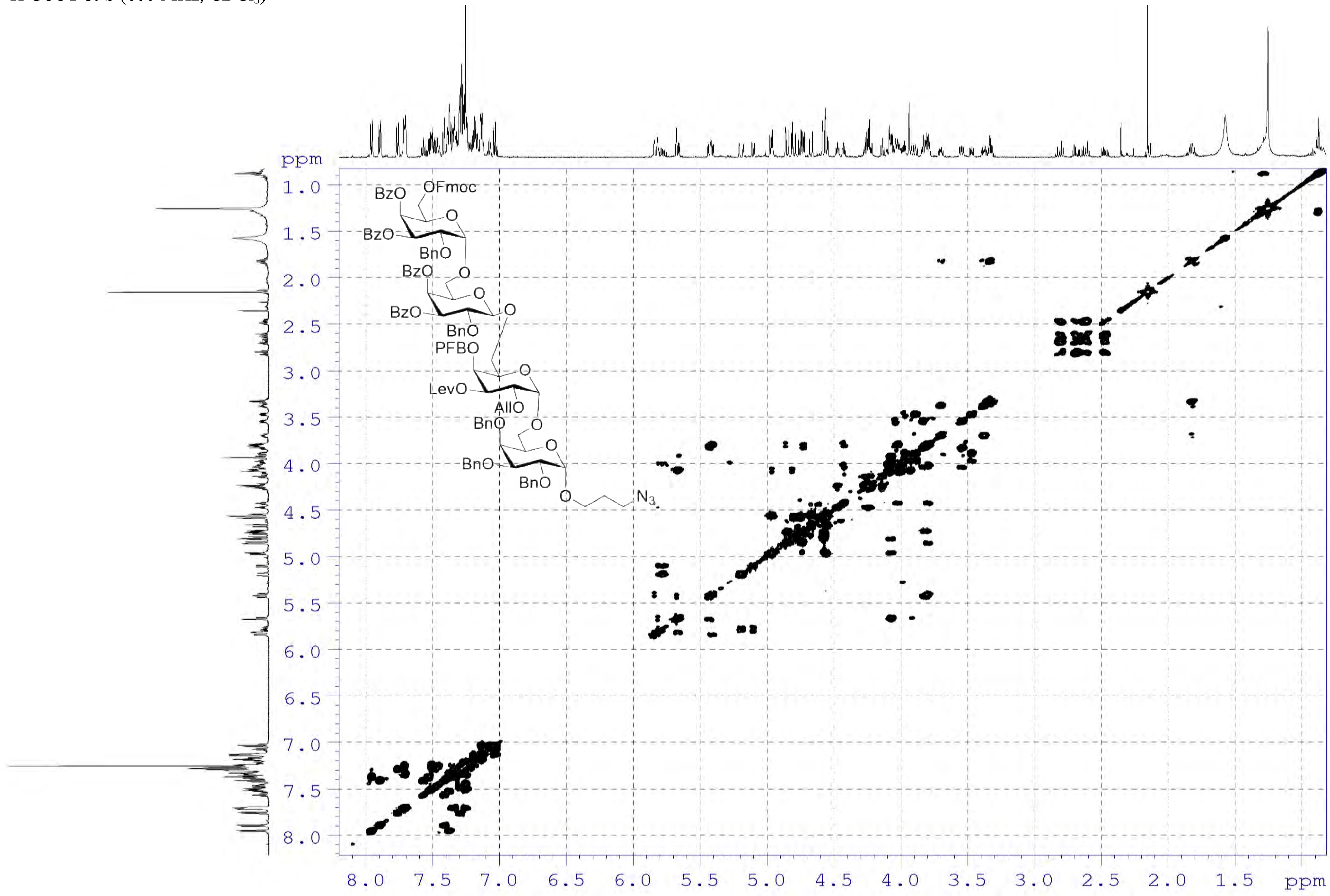

^{1}H-^{13}C HSQC of **39b** (600 MHz, $CDCl_3$)

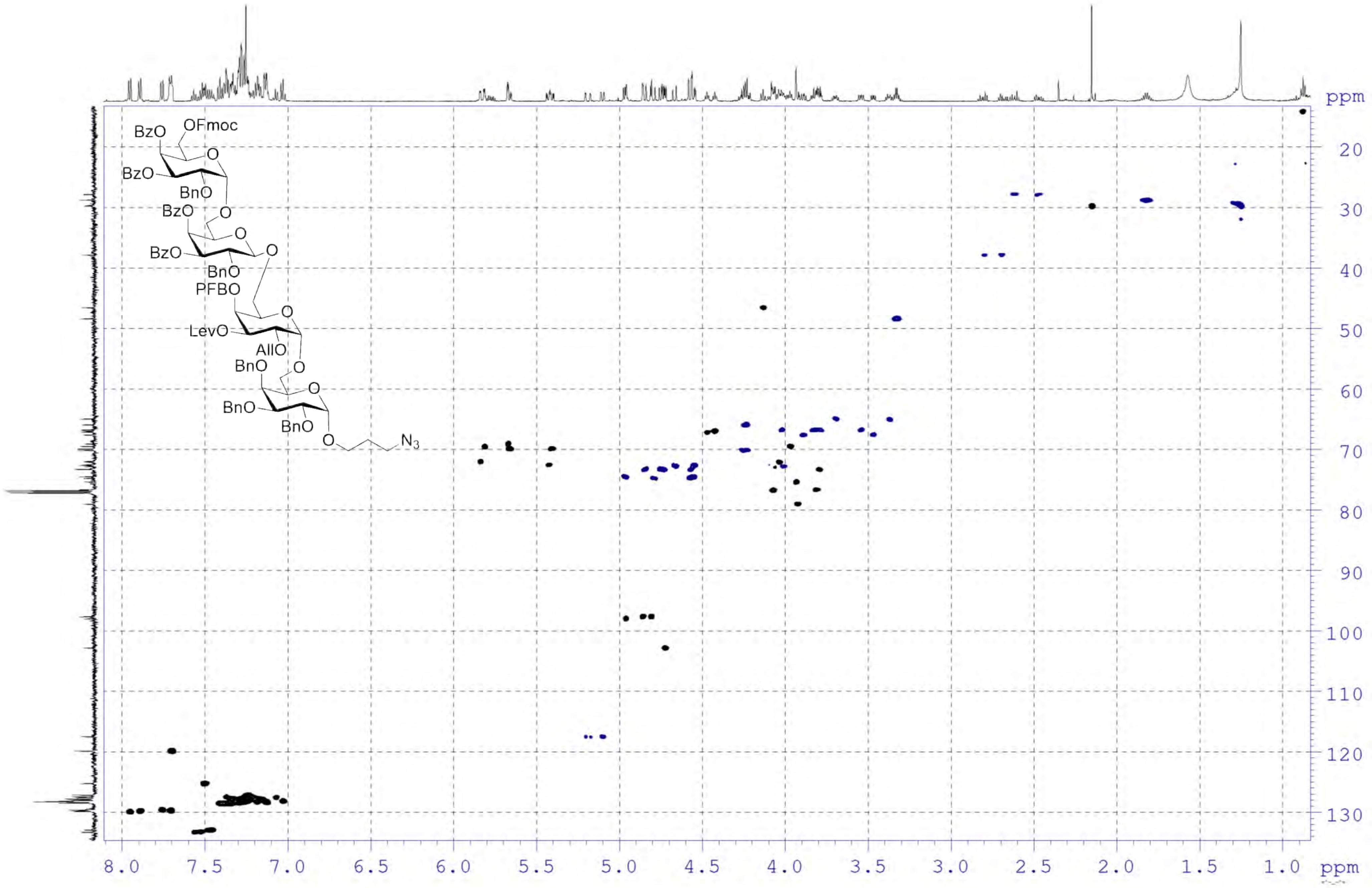

^{1}H-NMR of **40** (600 MHz, $CDCl_3$)

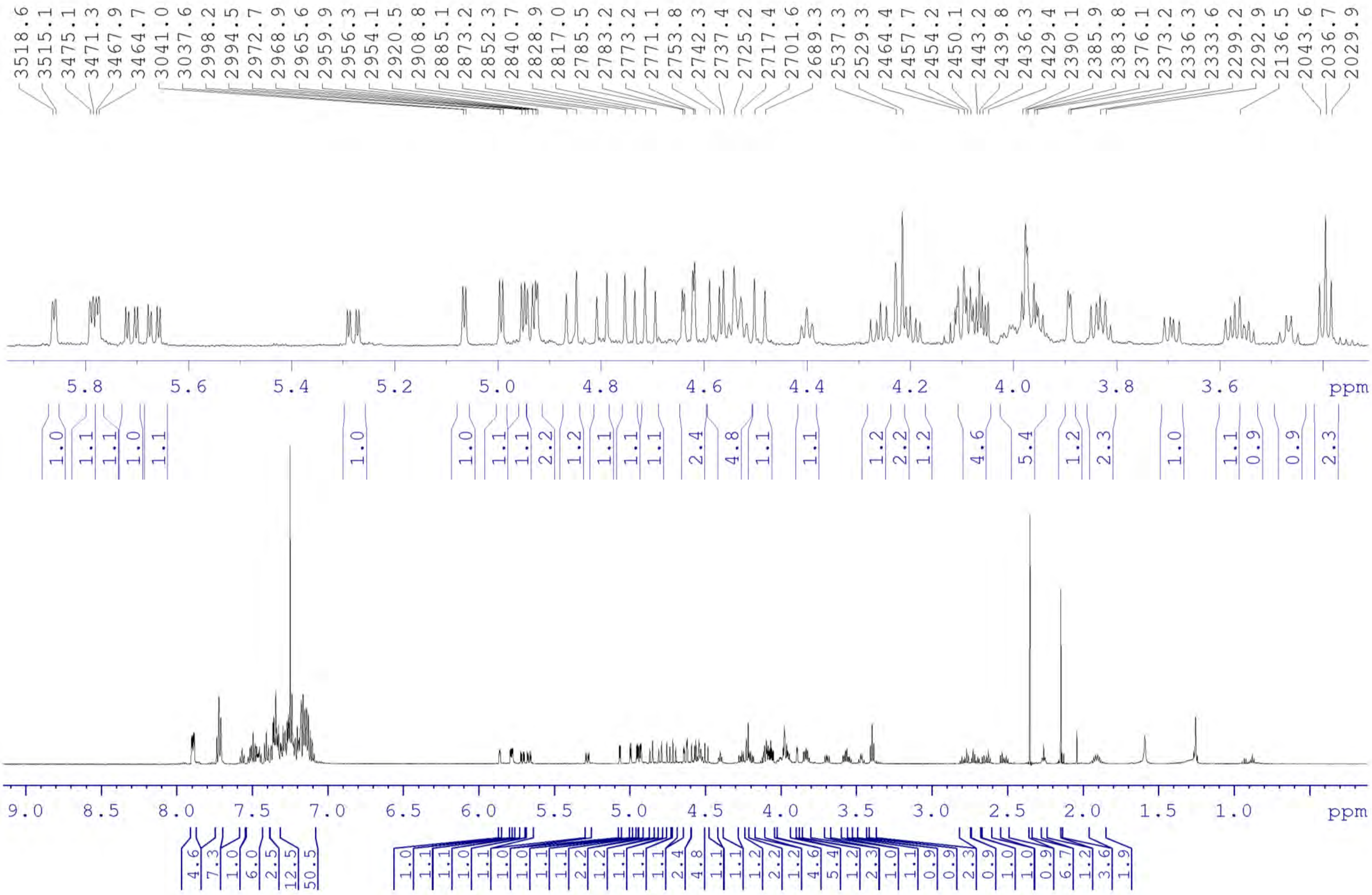

^{13}C-NMR of **40** (150 MHz, $CDCl_3$)

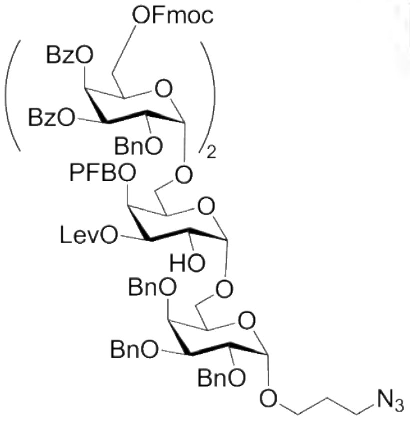

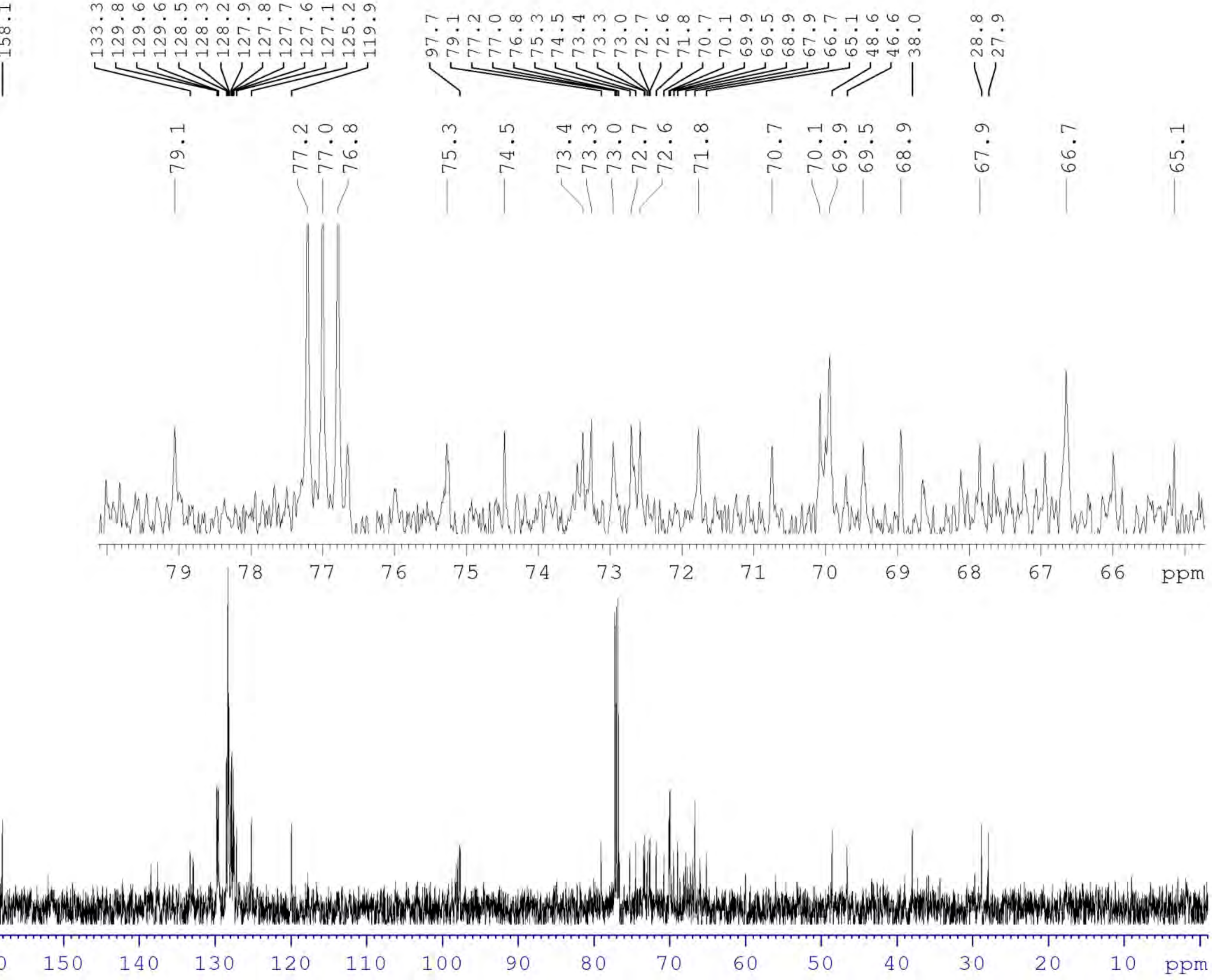

^{1}H-^{1}H COSY of **40** (600 MHz, $CDCl_3$)

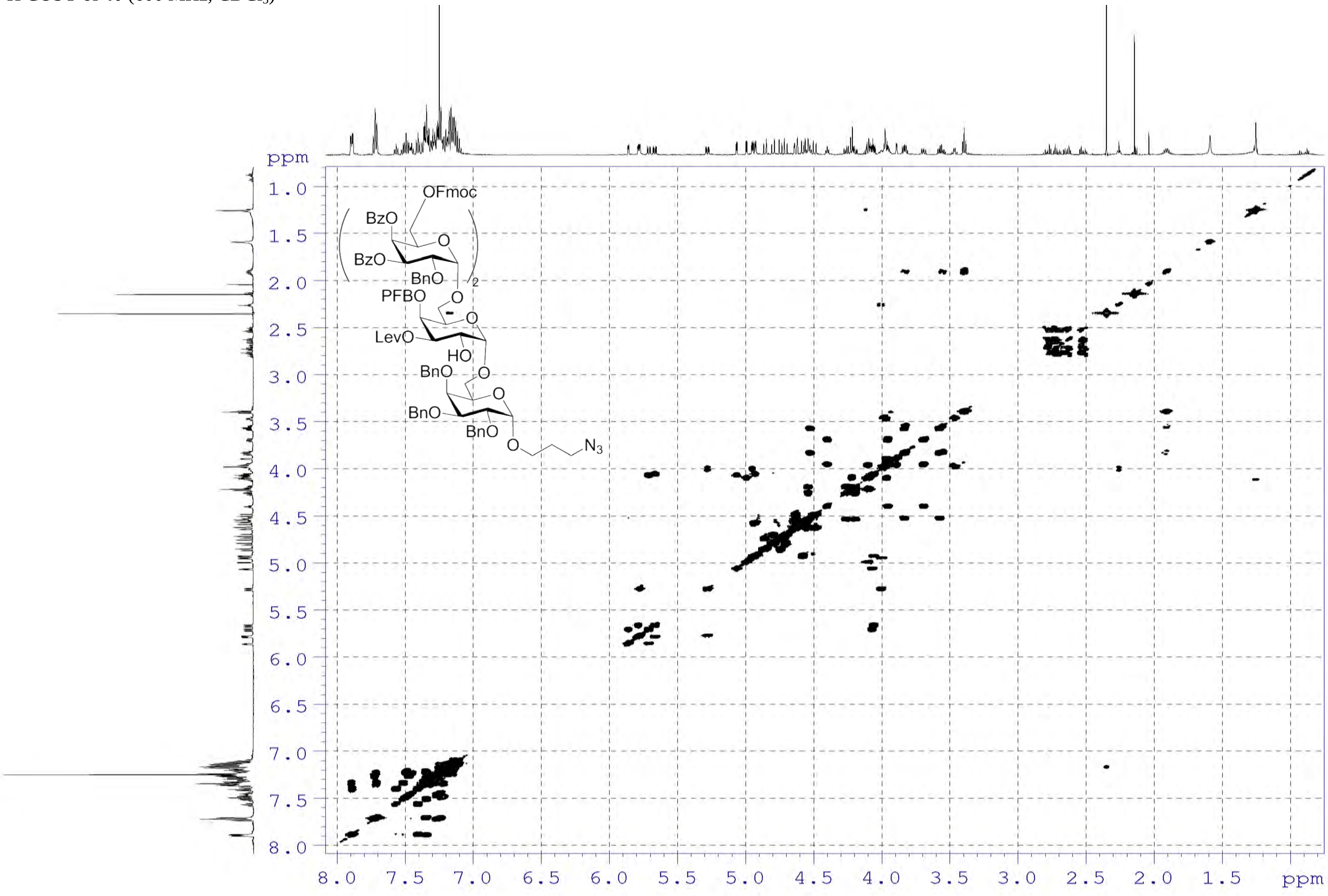

^{1}H-^{13}C HSQC of **40** (600 MHz, $CDCl_3$)

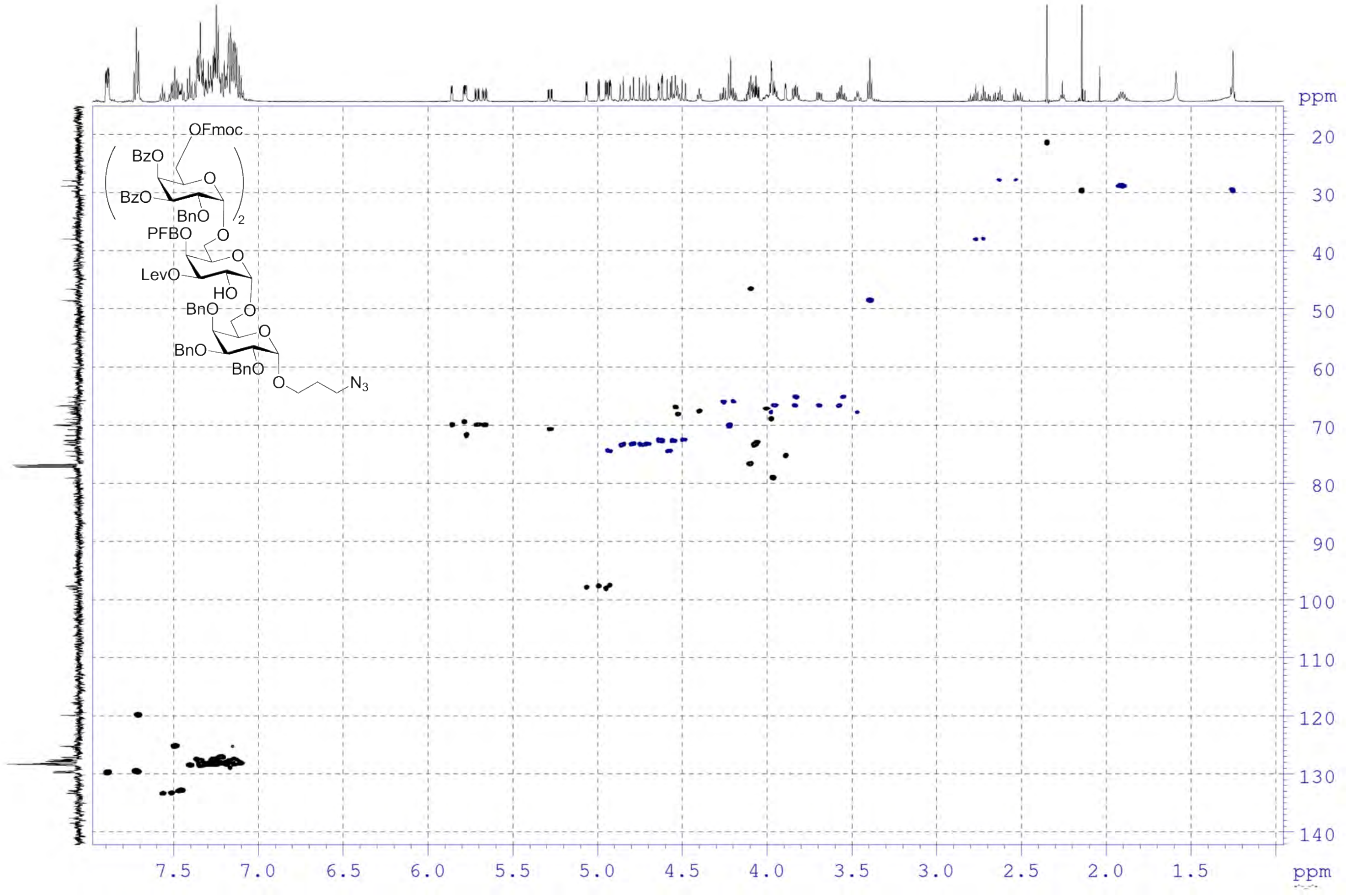

[1] H-NMR of **41** (600 MHz, $CDCl_3$)

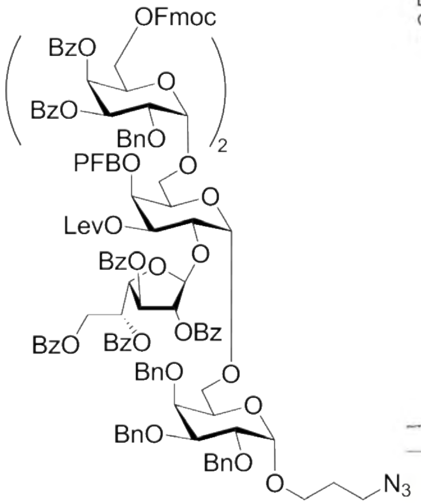

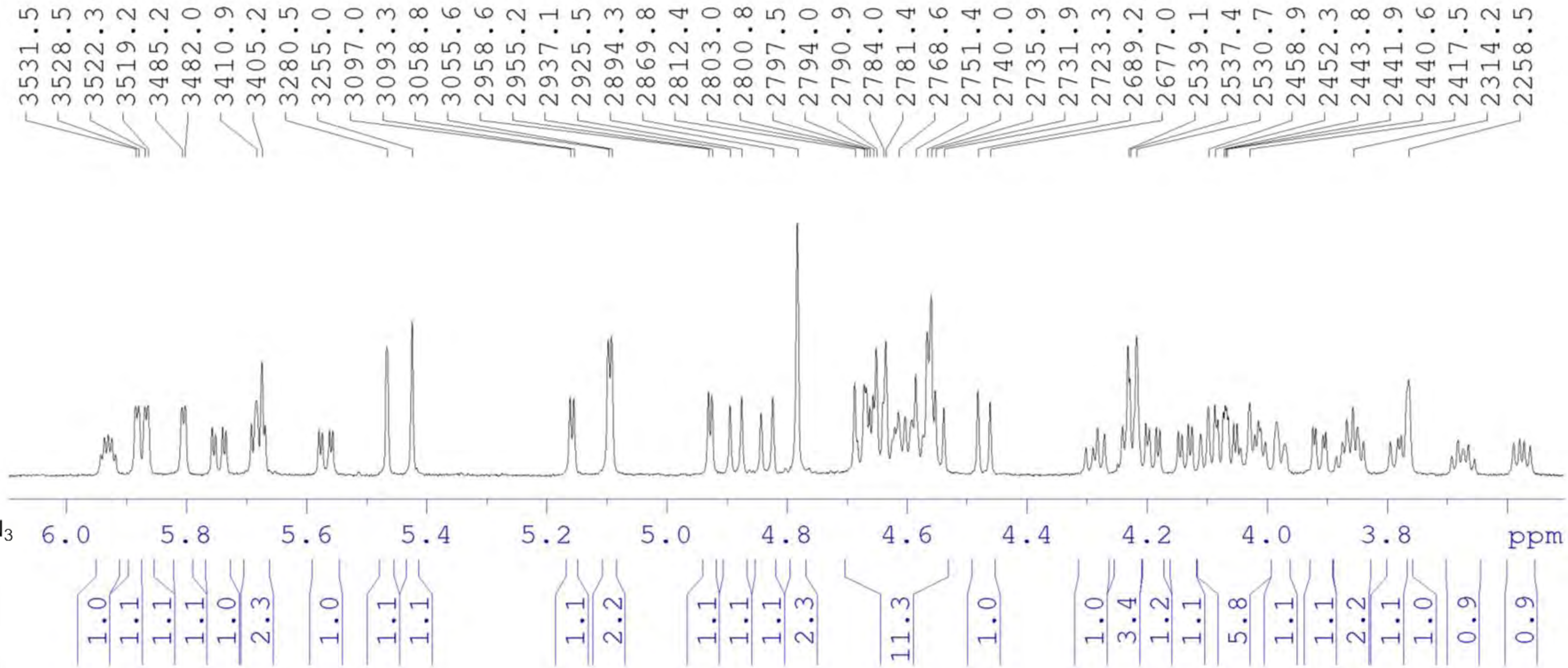

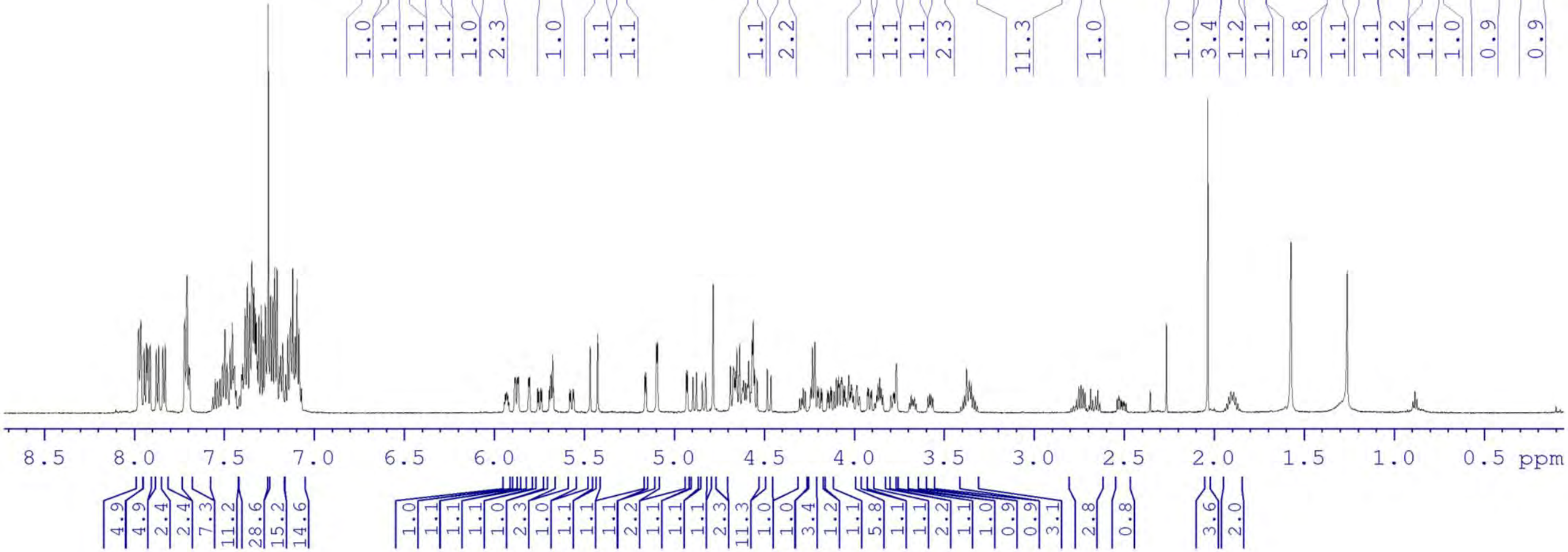

^{13}C-NMR of **41** (150 MHz, $CDCl_3$)

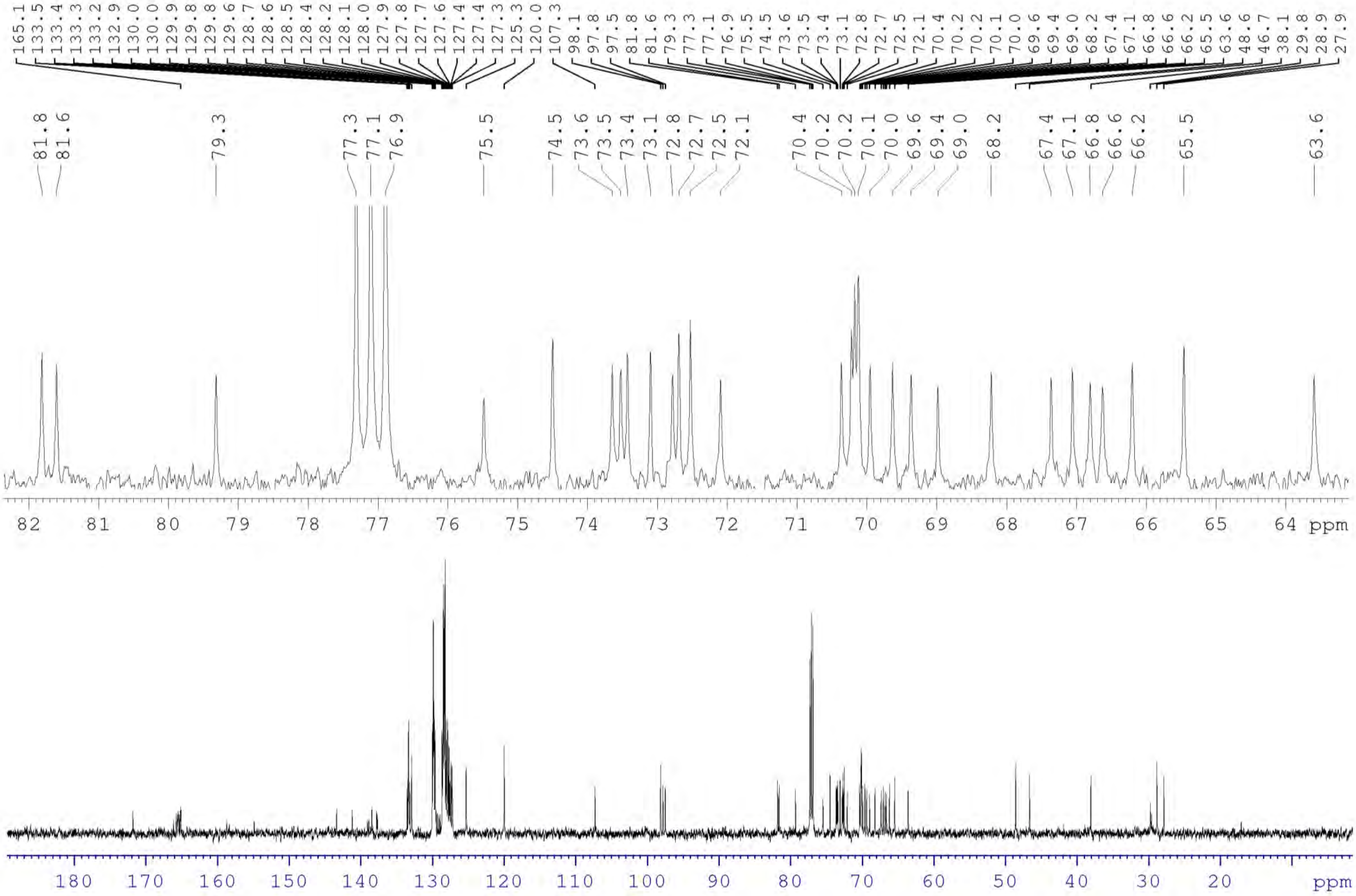

^{1}H-^{1}H COSY of **41** (600 MHz, $CDCl_3$)

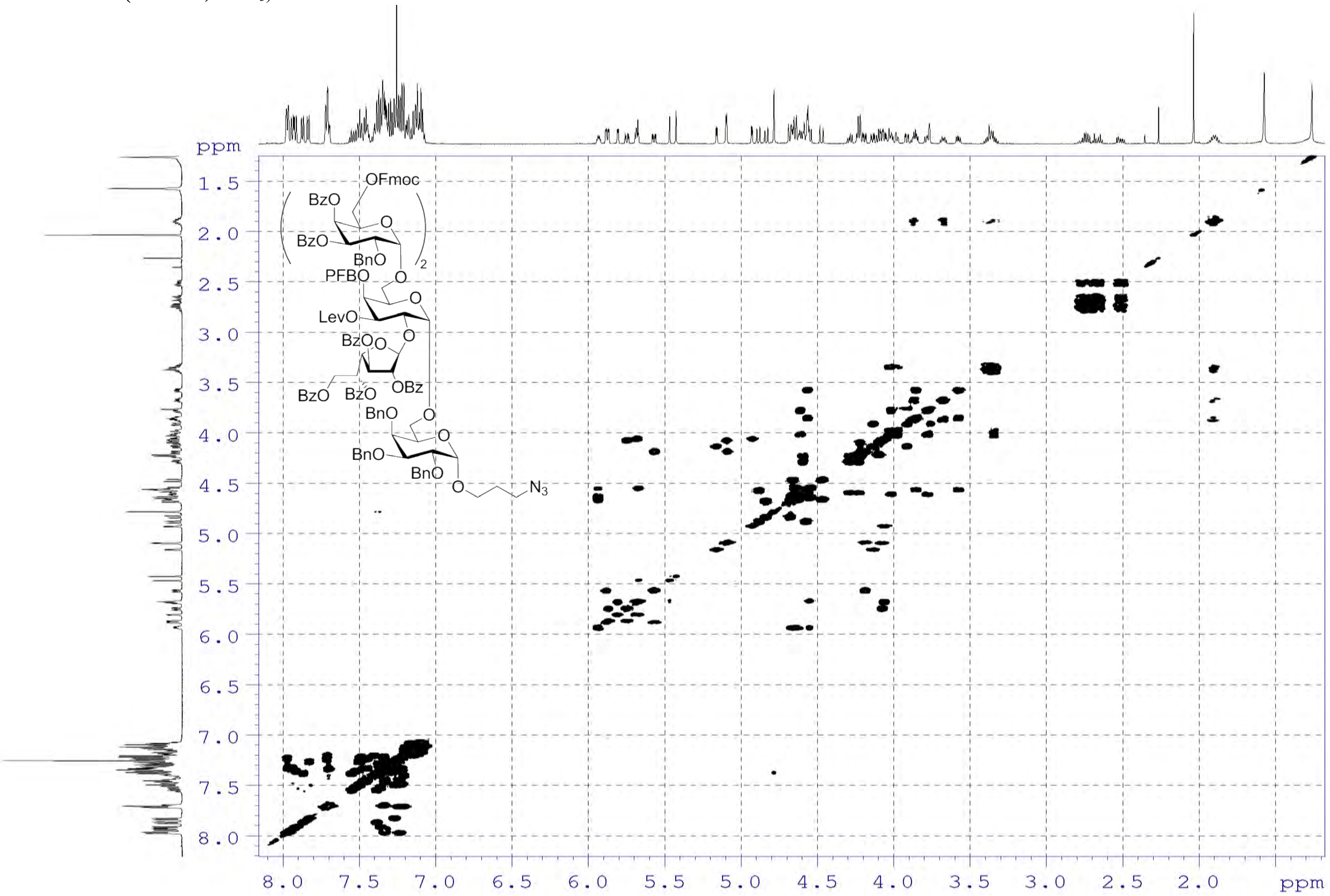

^{1}H-^{13}C HSQC of **41** (600 MHz, $CDCl_3$)

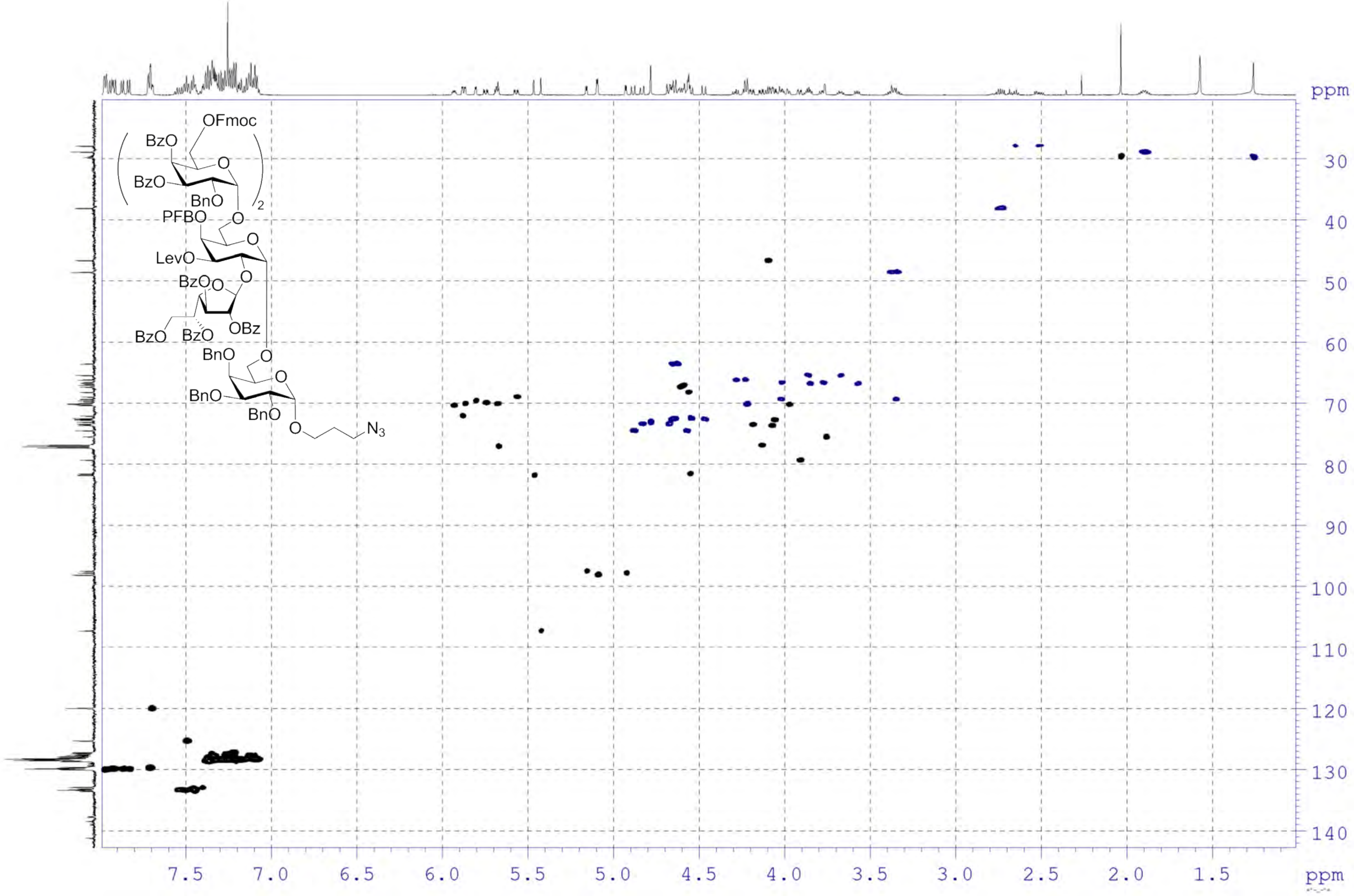

^{1}H-NMR of **42** (600 MHz, $CDCl_3$)

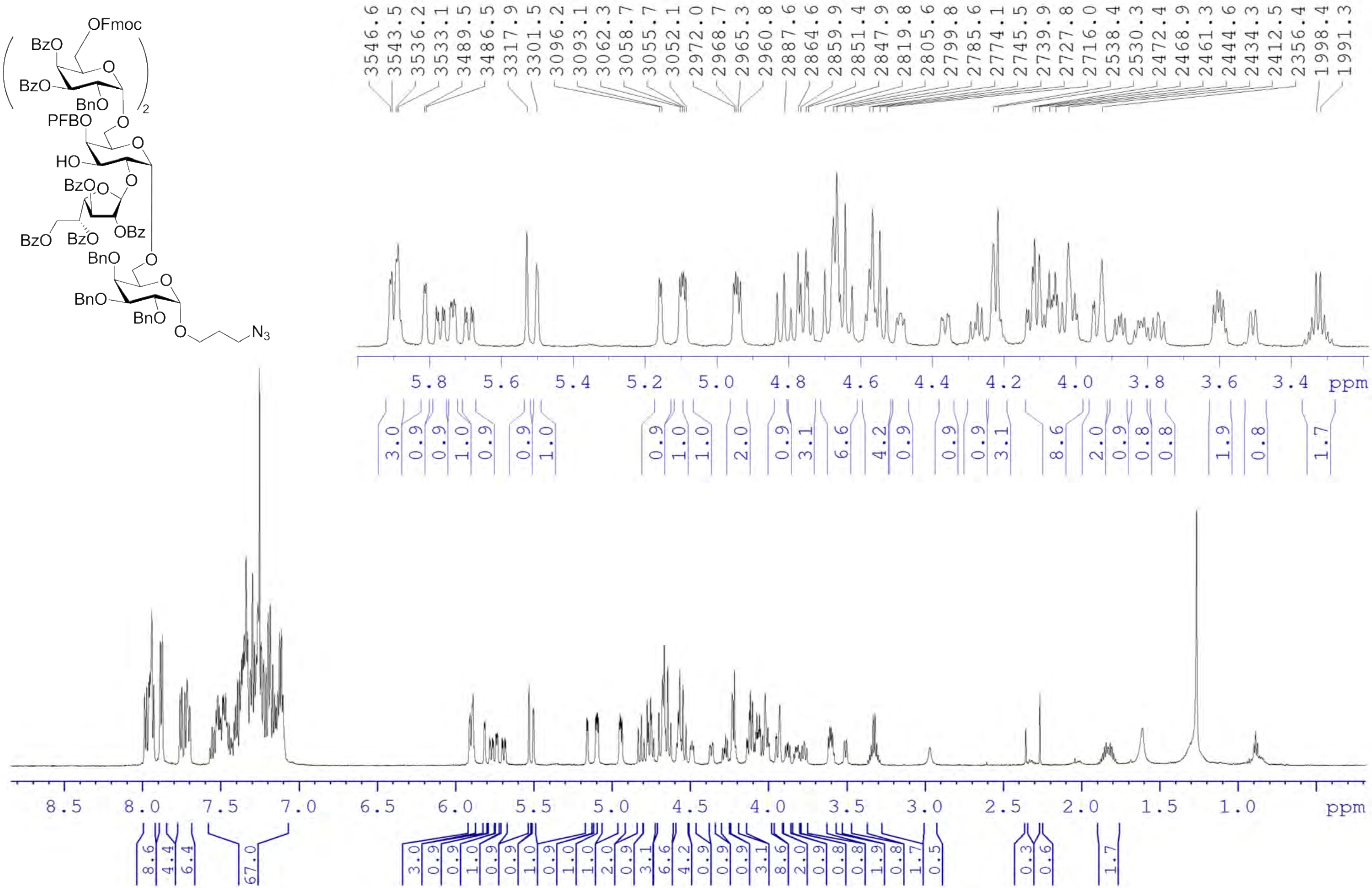

^{13}C-NMR of **42** (150 MHz, $CDCl_3$)

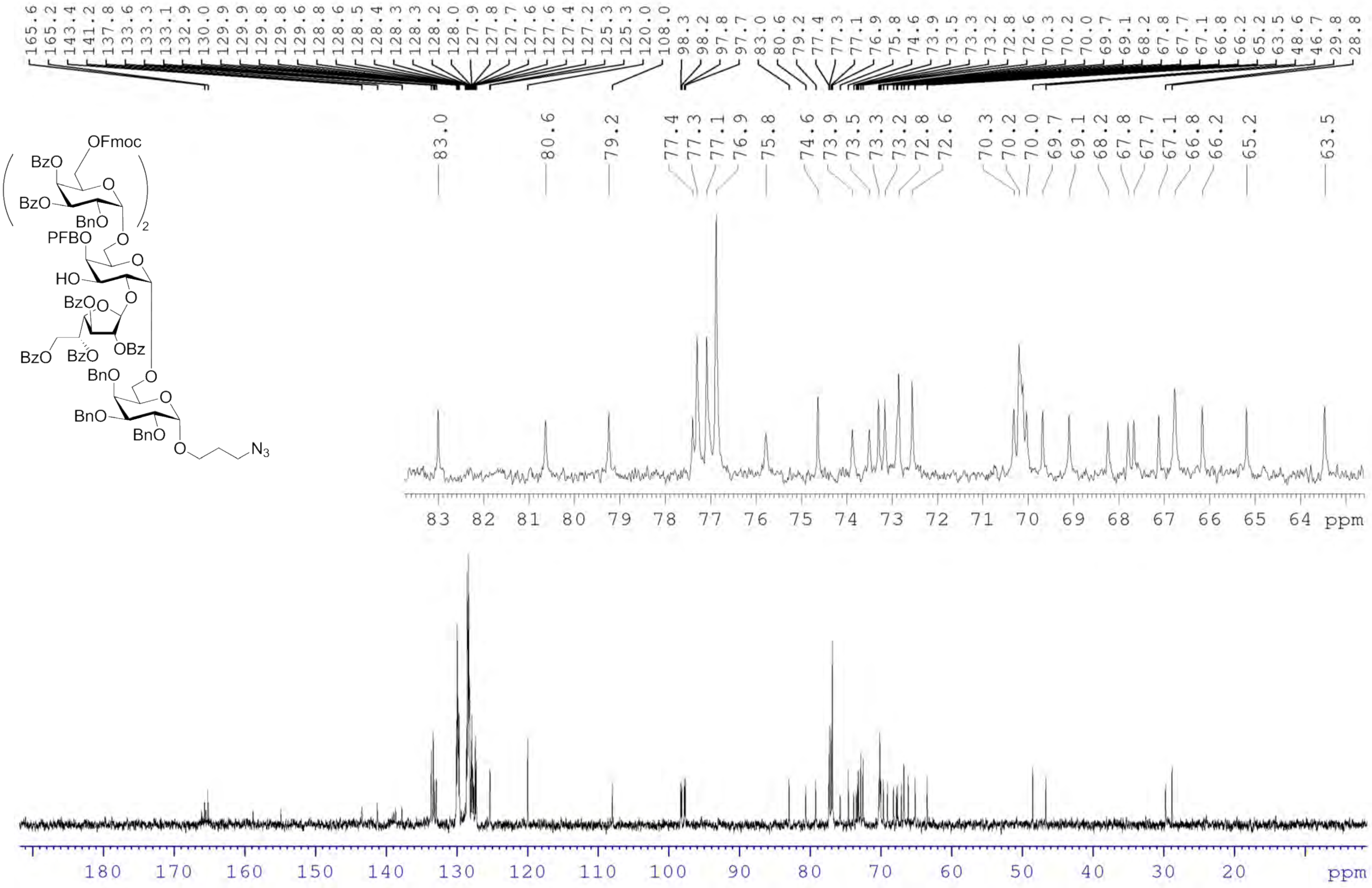

^{1}H-^{1}H COSY of **42** (600 MHz, $CDCl_3$)

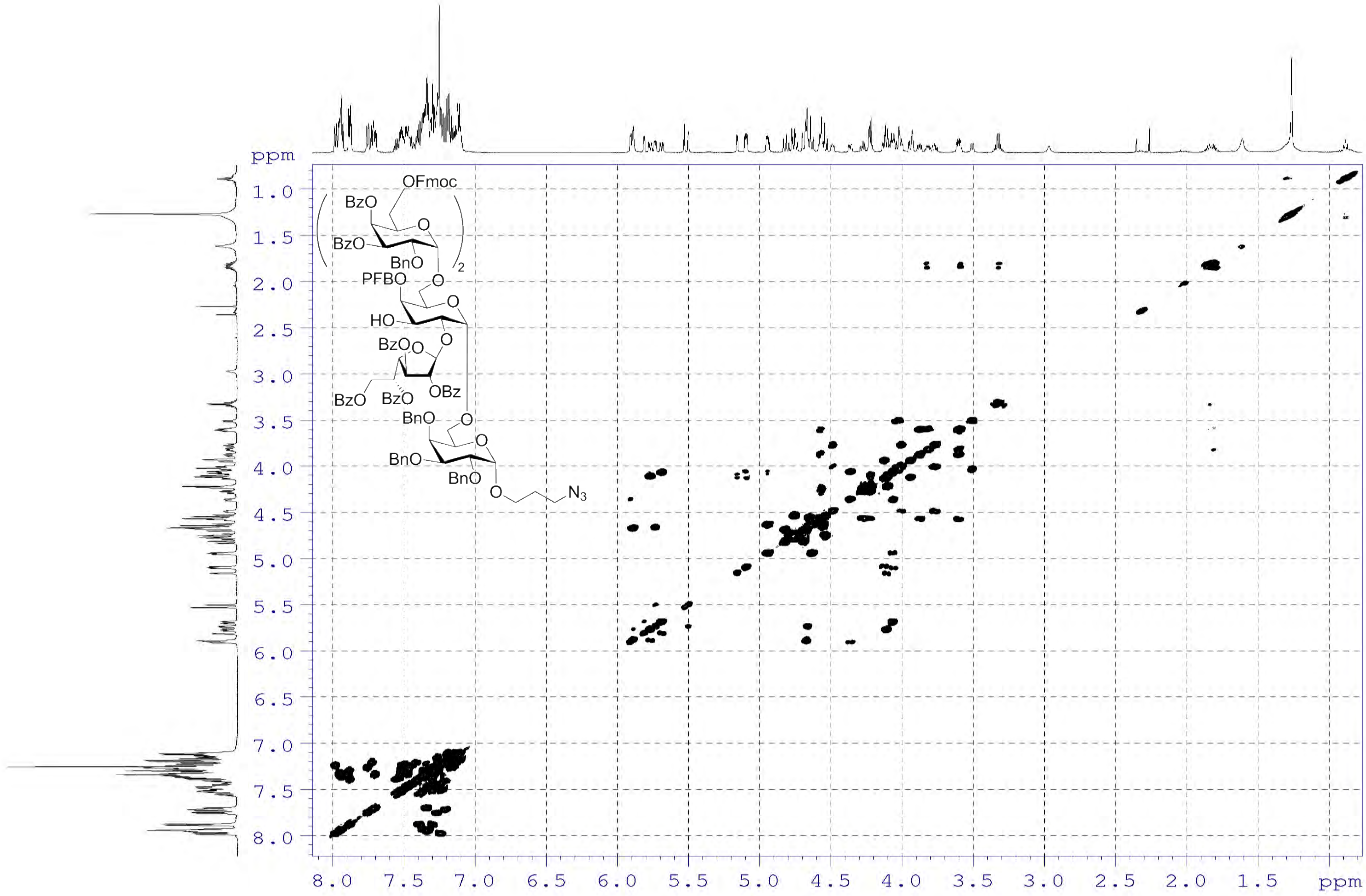

^{1}H-^{13}C HSQC of **42** (600 MHz, $CDCl_3$)

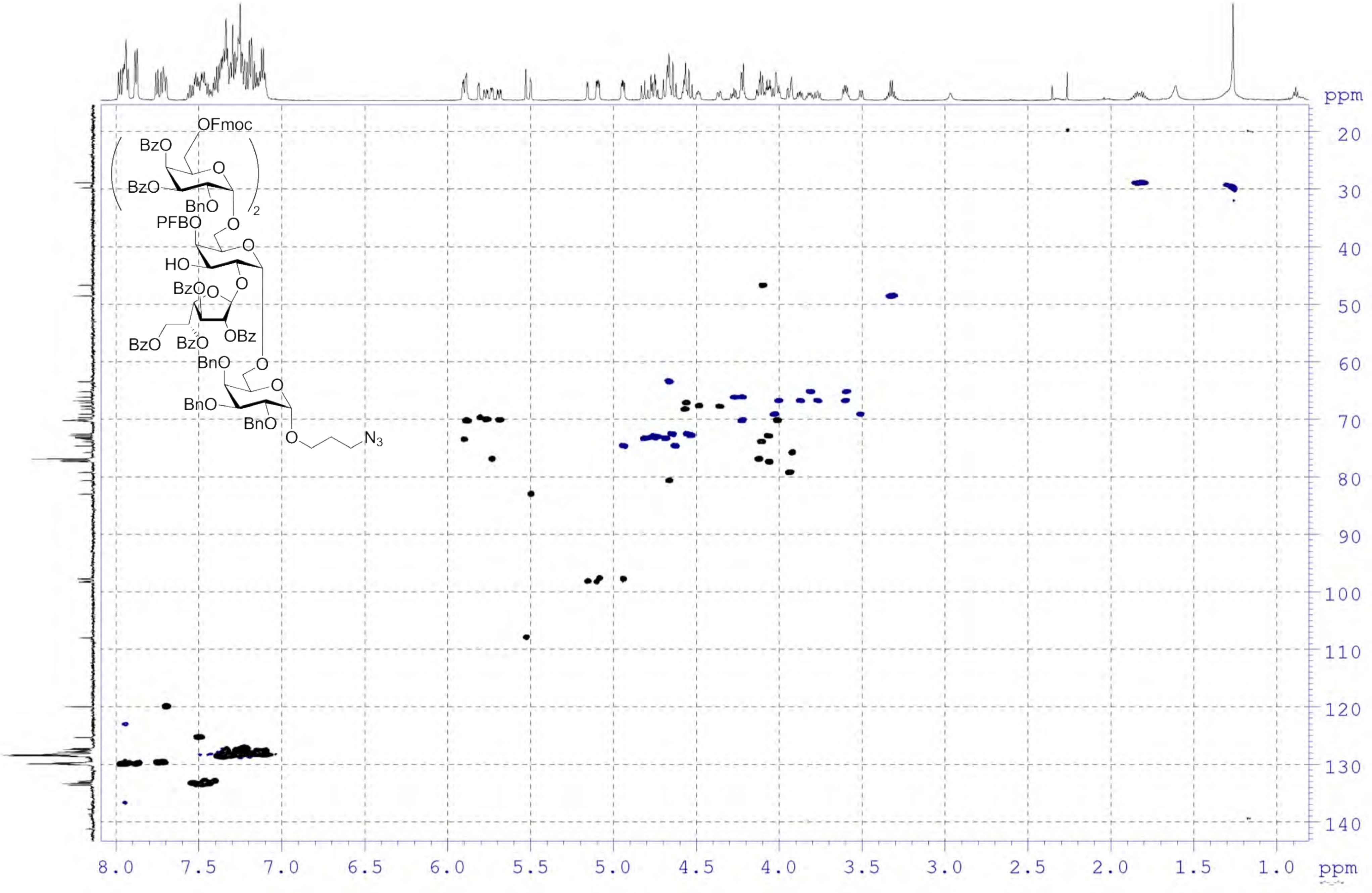

^{1}H-NMR of **43** (600 MHz, $CDCl_3$)

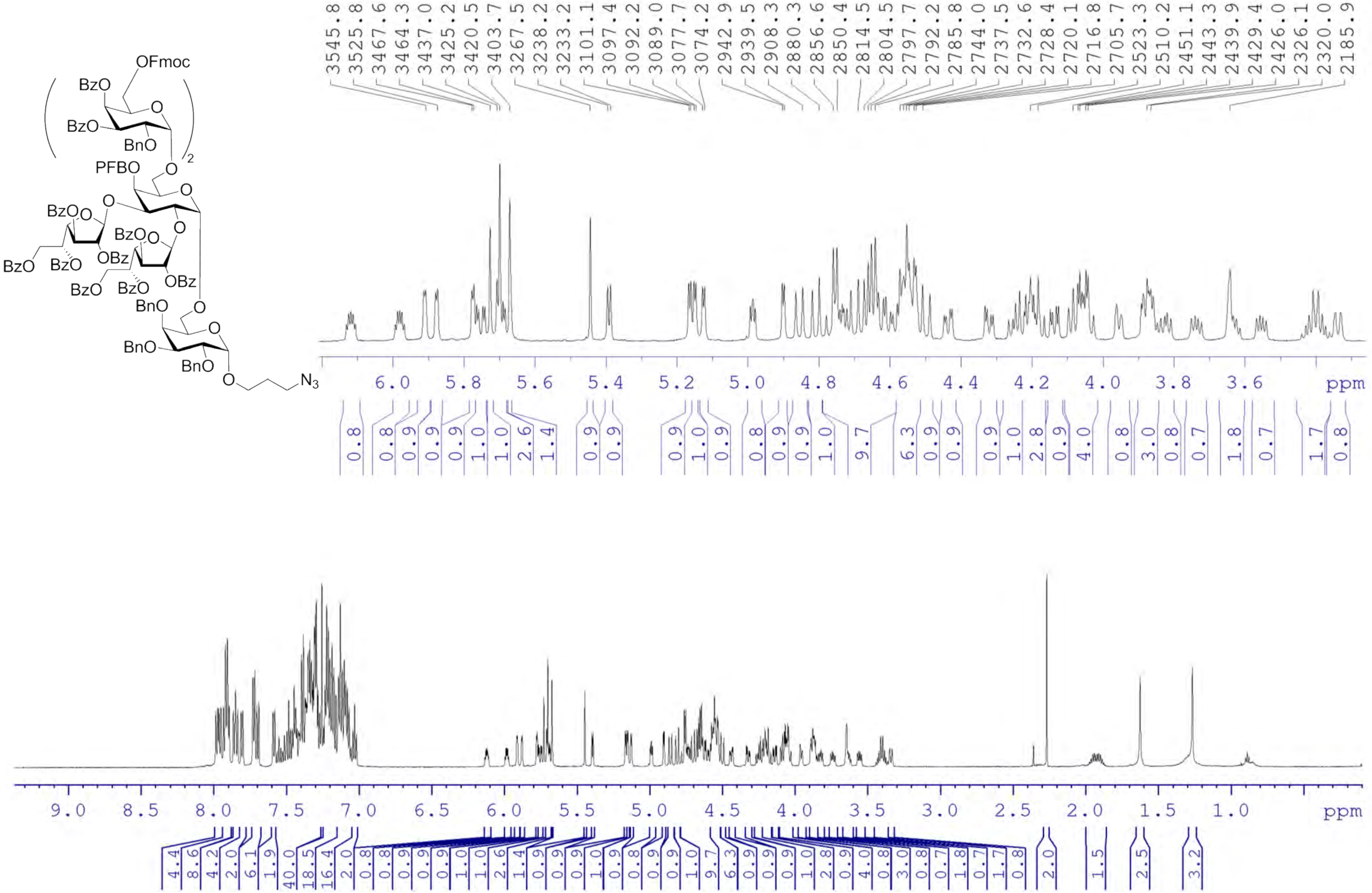

^{13}C-NMR of **43** (150 MHz, $CDCl_3$)

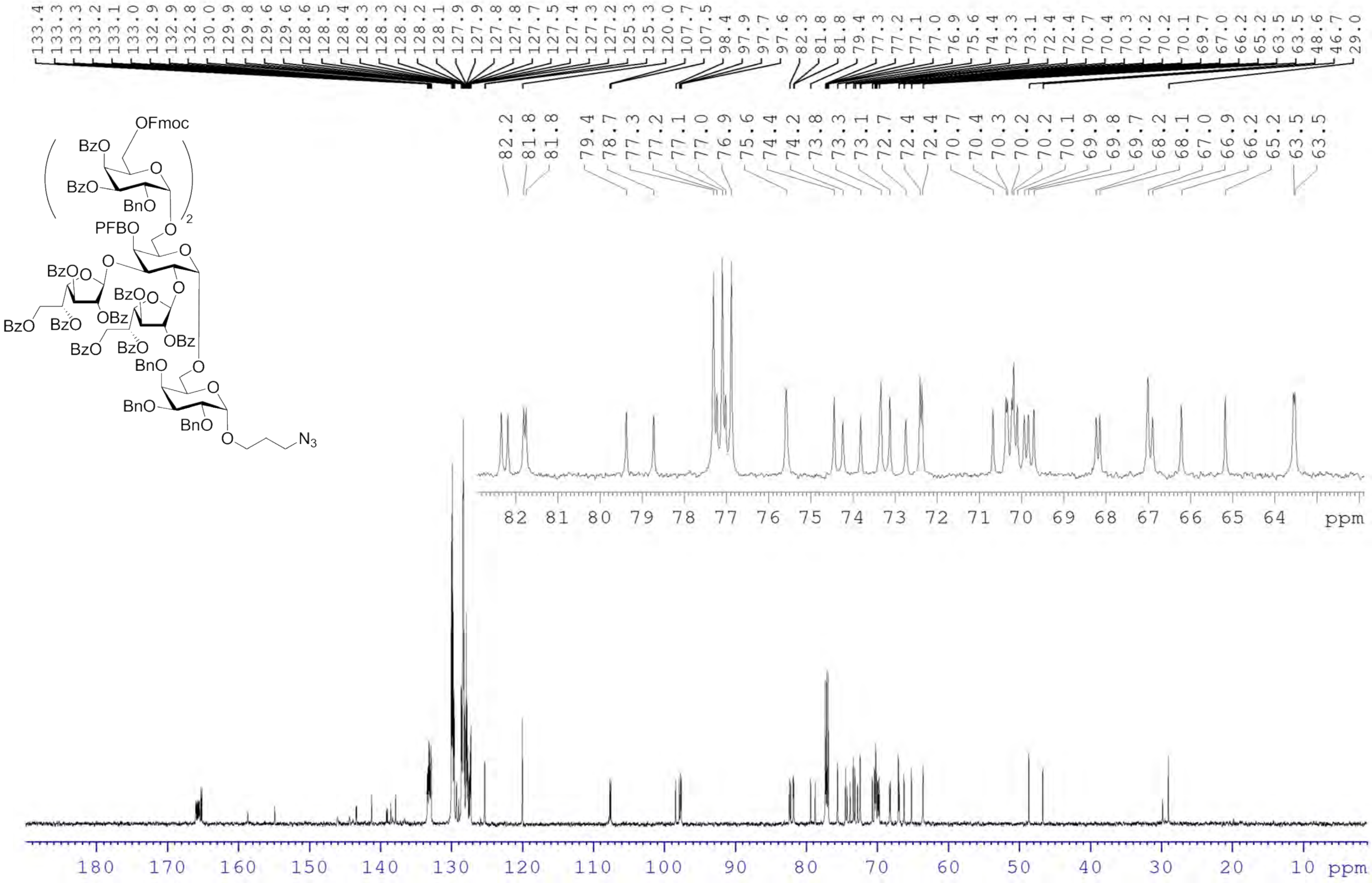

^{1}H-^{1}H COSY of **43**(600 MHz, $CDCl_3$)

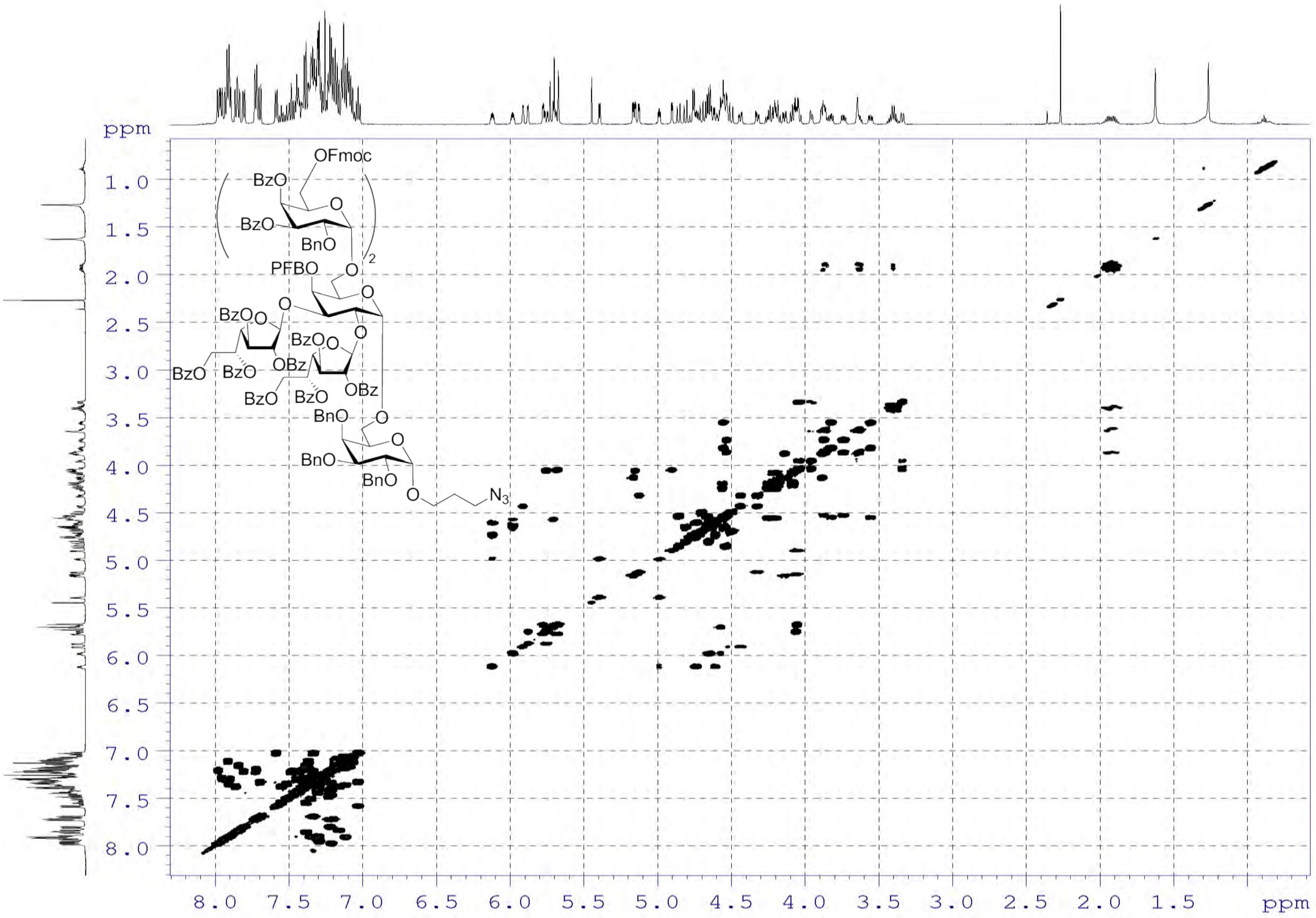

^{1}H-^{13}C HSQC of **43** (600 MHz, CDCl$_3$)

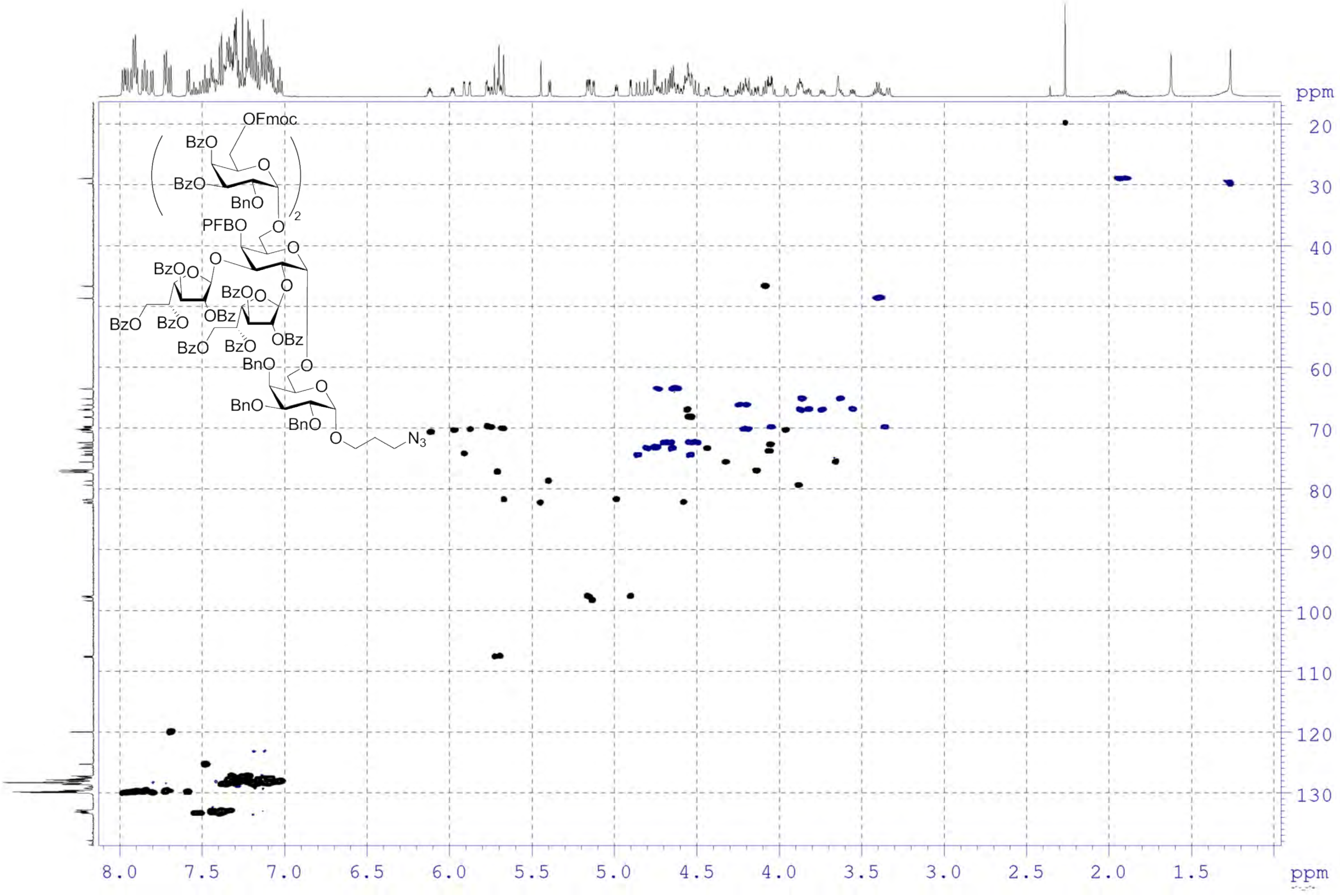

^{1}H-NMR of **S8** (600 MHz, $CDCl_3 + CD_3OD$)

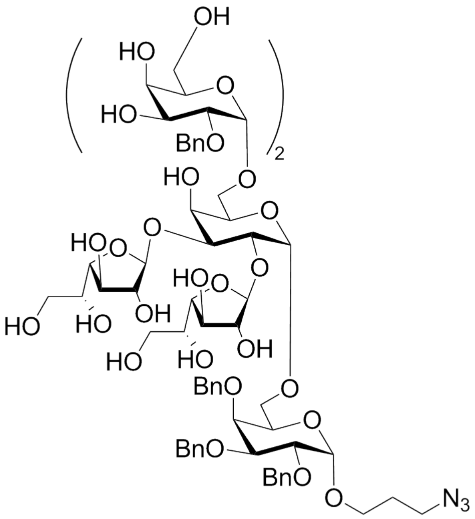

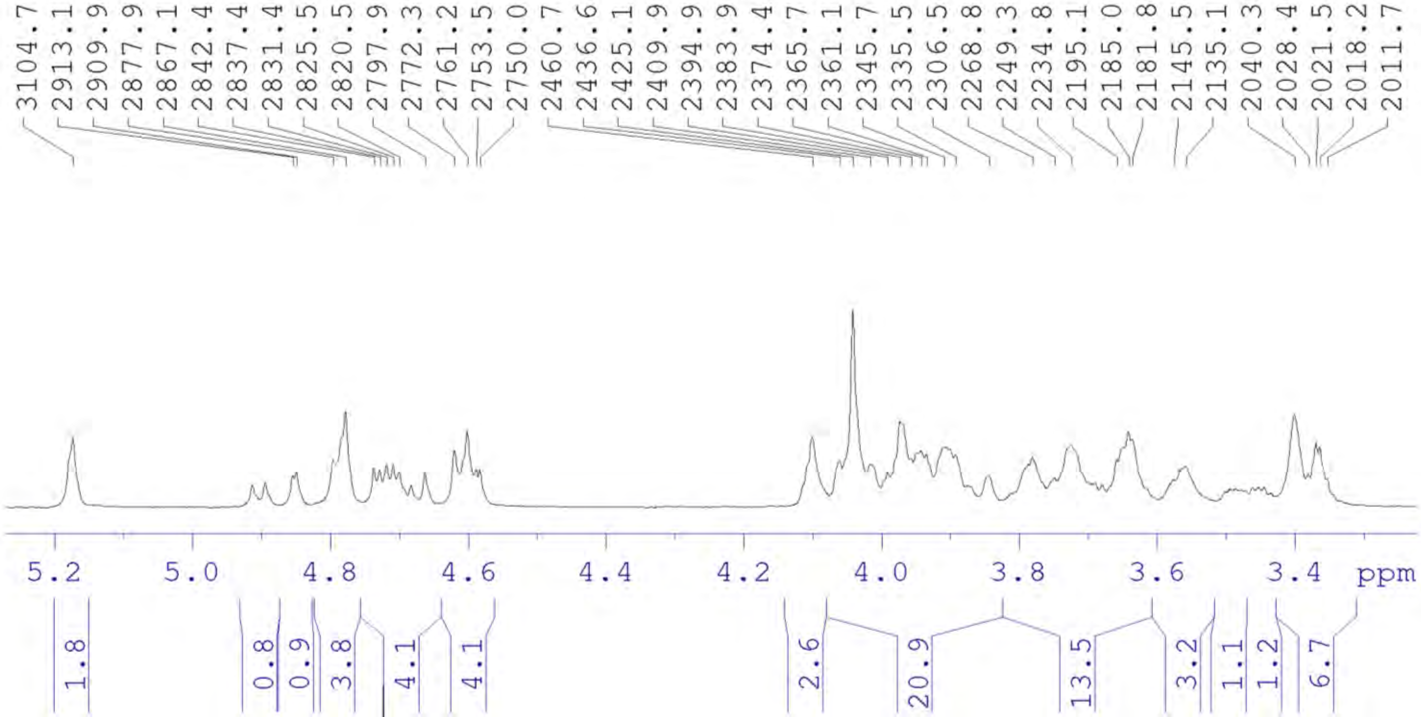

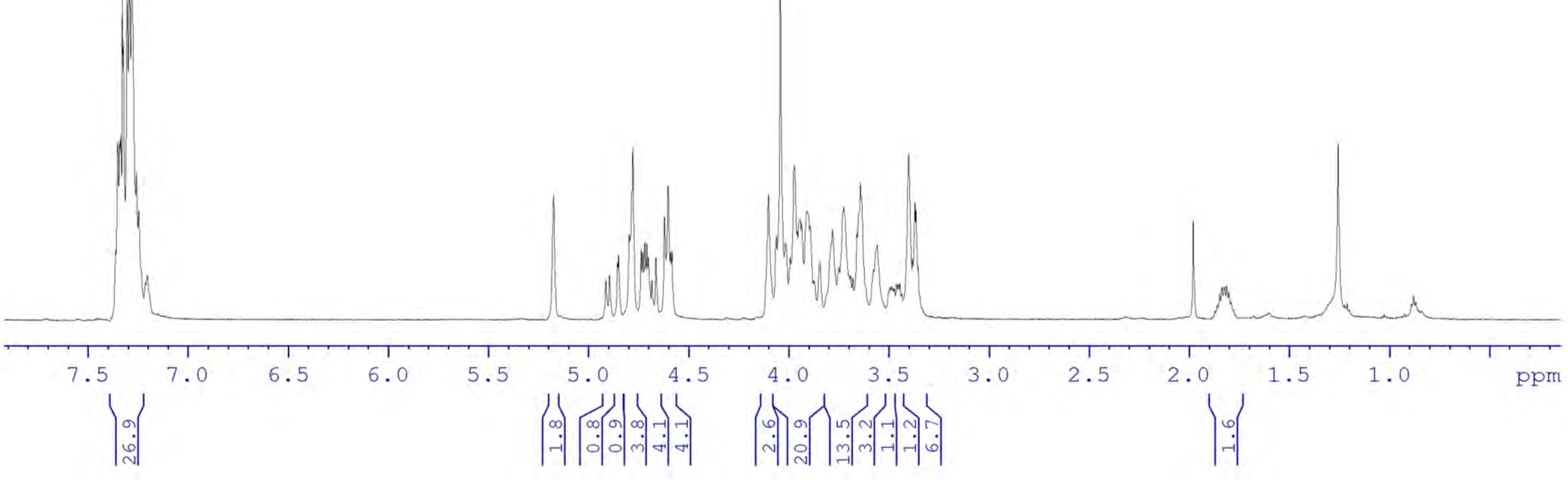

^{13}C-NMR of **S8** (150 MHz, $CDCl_3$ + CD_3OD)

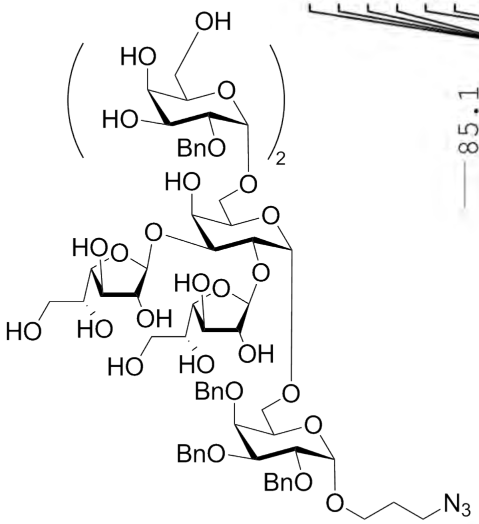

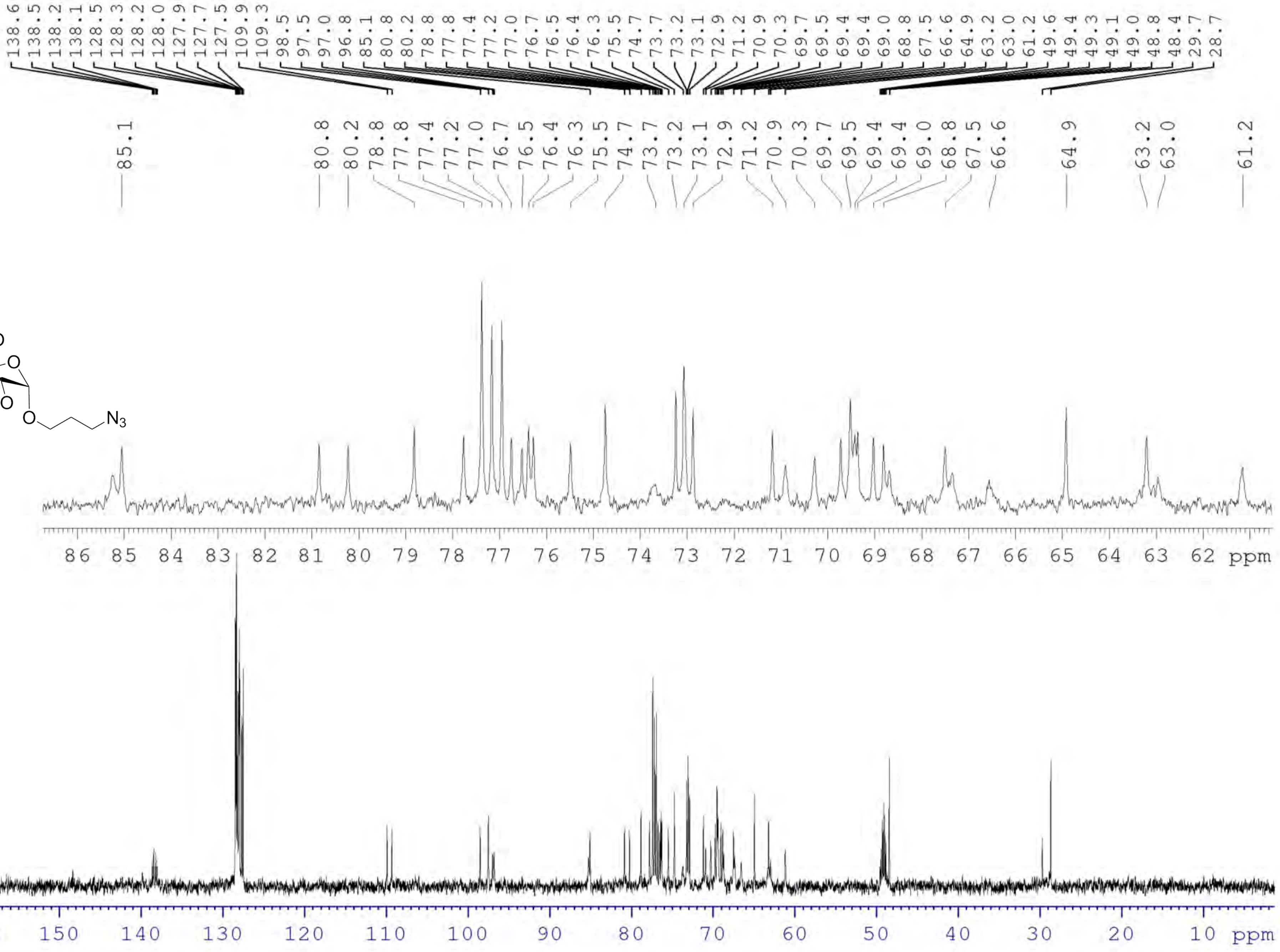

^{1}H-^{1}H COSY of **S8** (600 MHz, $CDCl_3$ + CD_3OD)

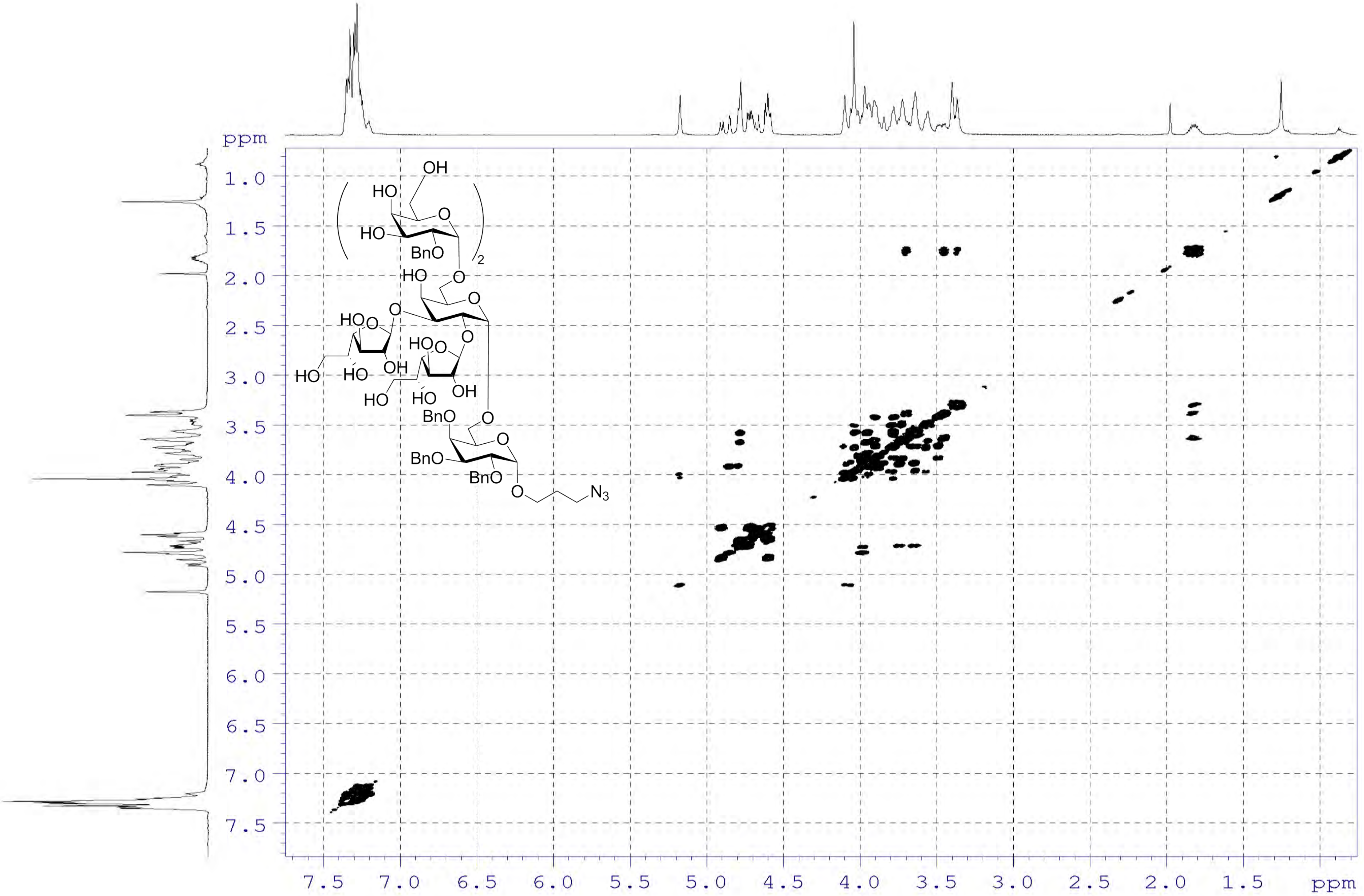

^{1}H-^{13}C HSQC of **S8** (600 MHz, $CDCl_3 + CD_3OD$)

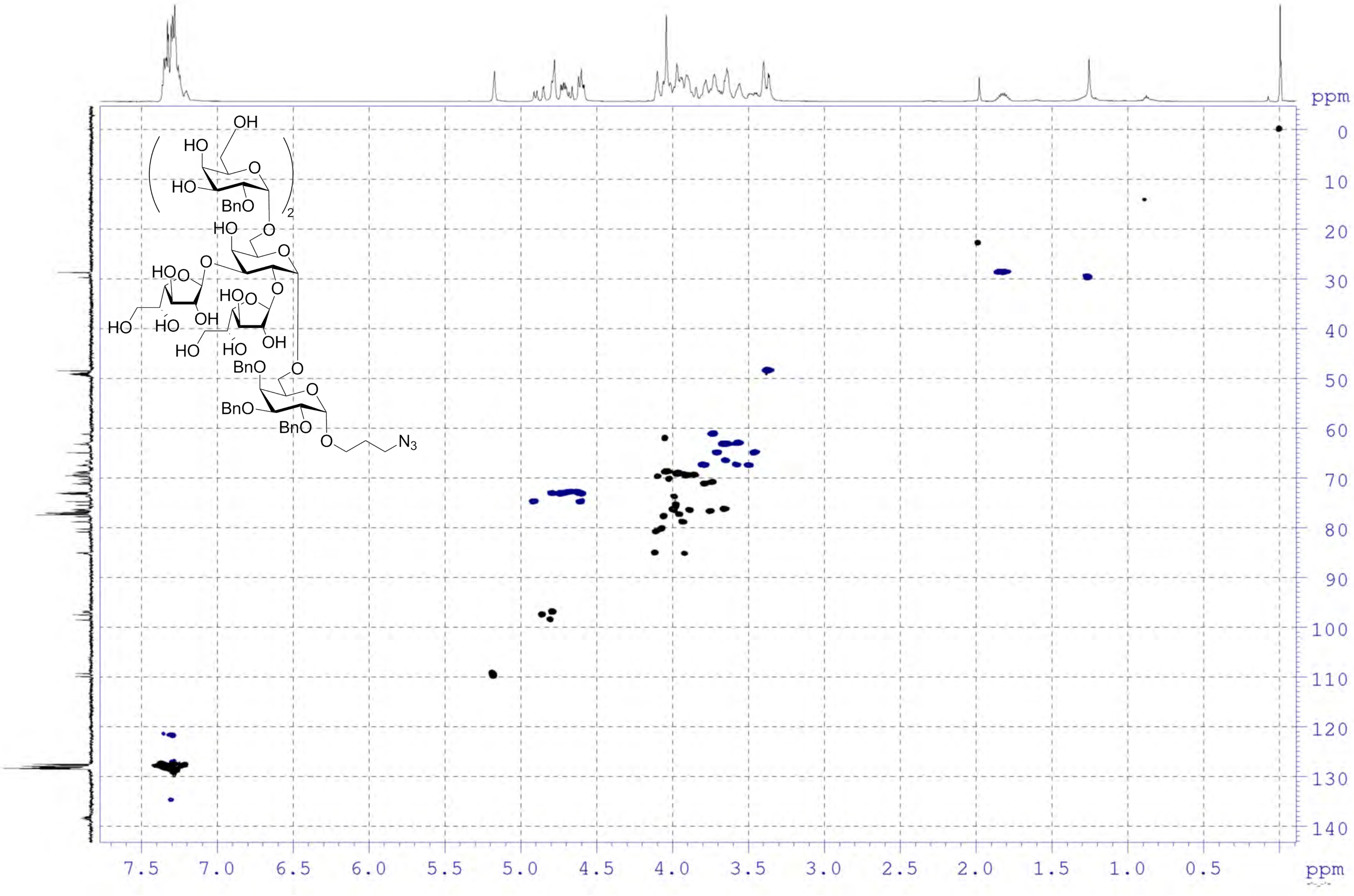

^{1}H-NMR of **5a** (600 MHz, D_2O, 303K)

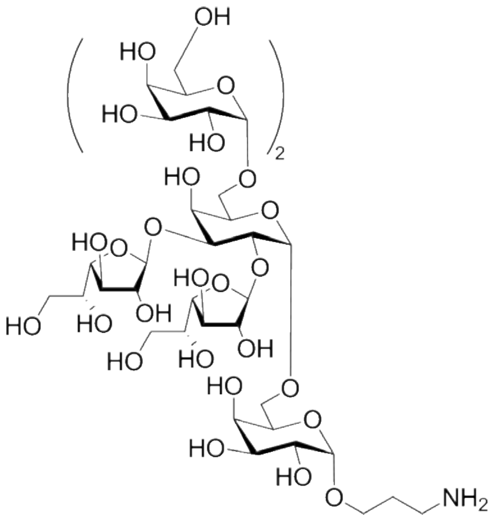

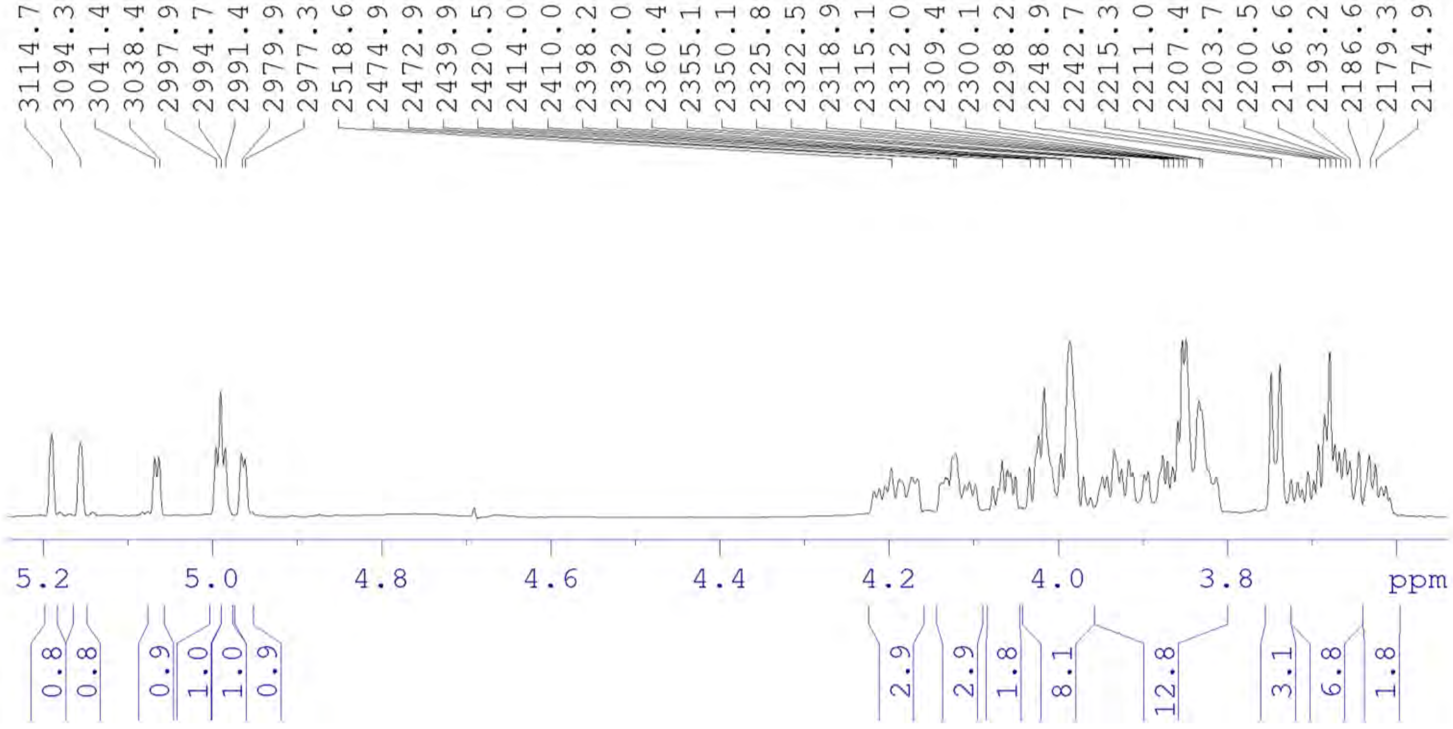

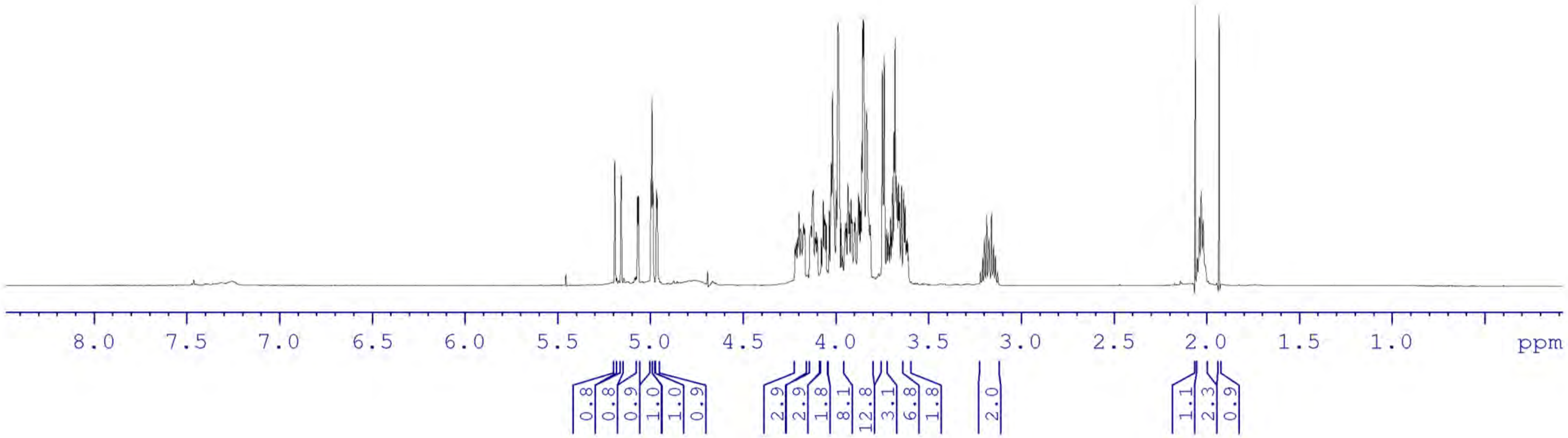

^{13}C-NMR of **5a** (150 MHz, D_2O, 303K)

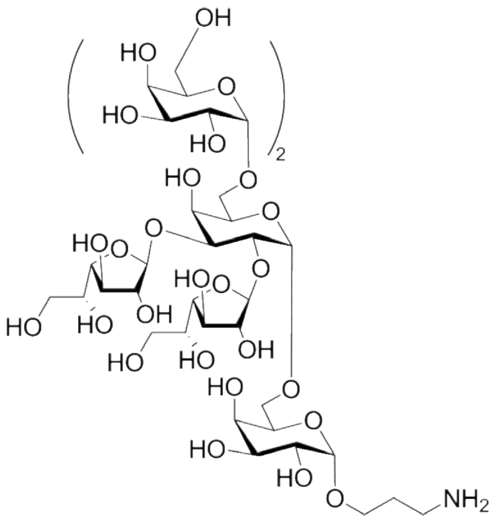

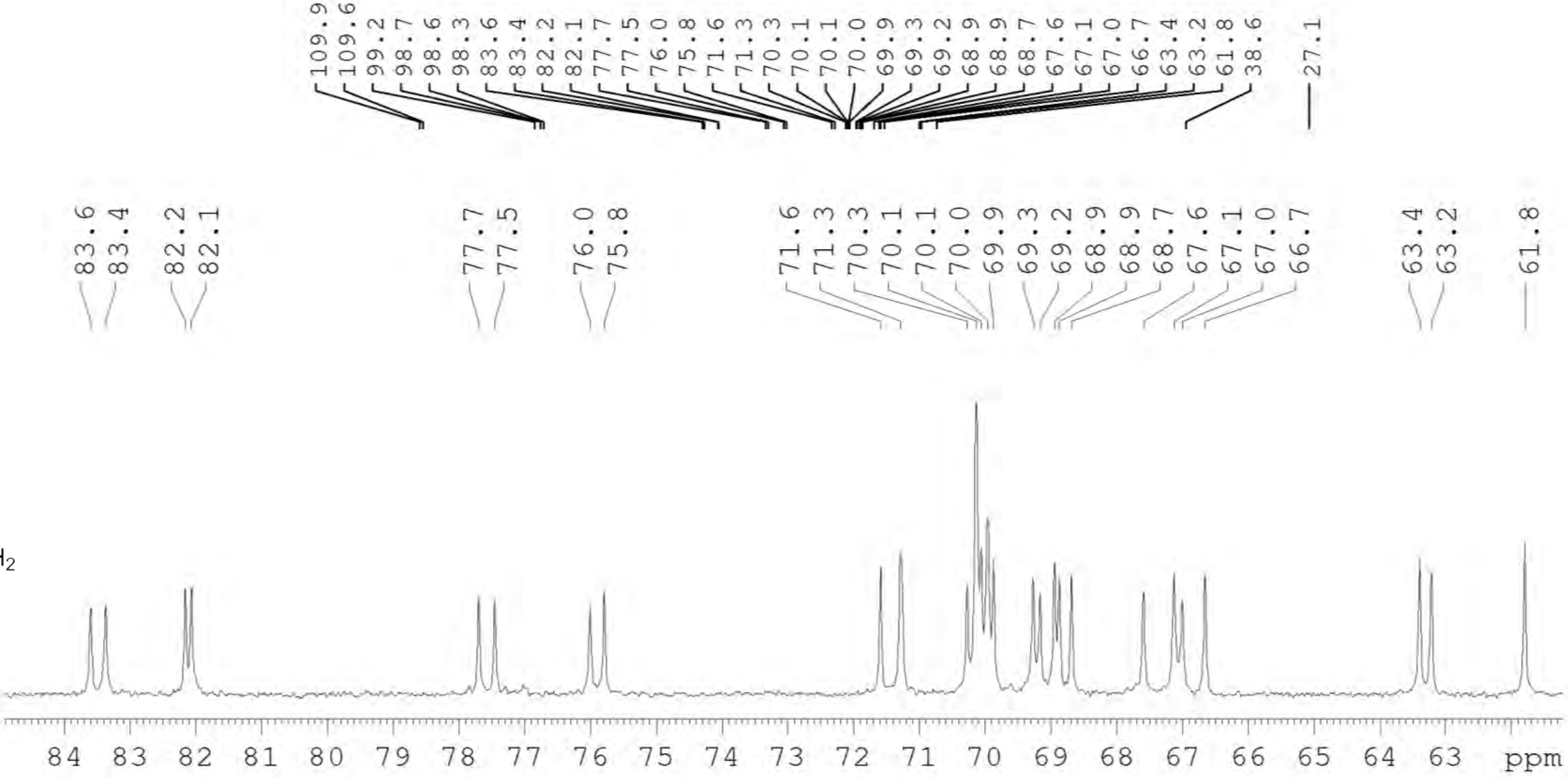

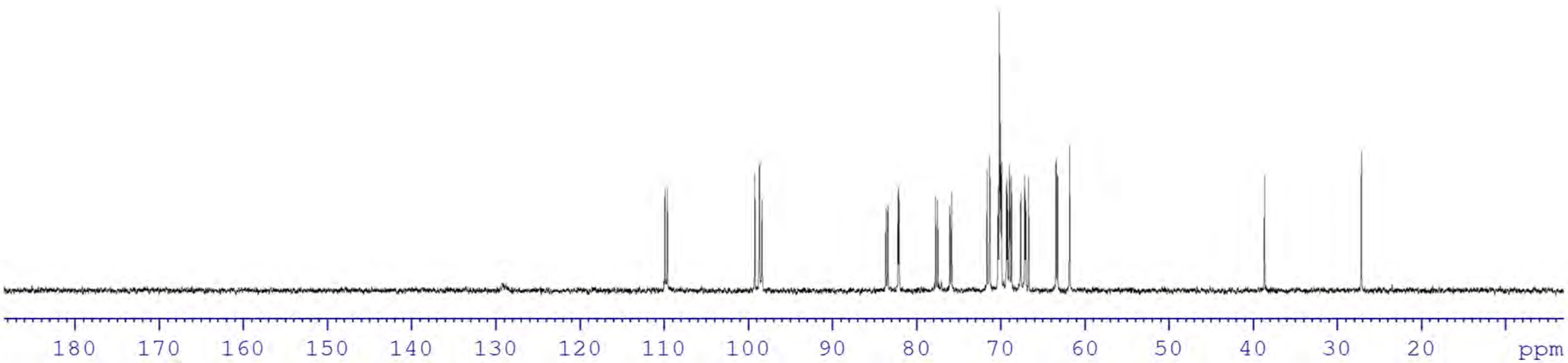

^{1}H-^{1}H COSY of **5a** (600 MHz, D_2O, 303K)

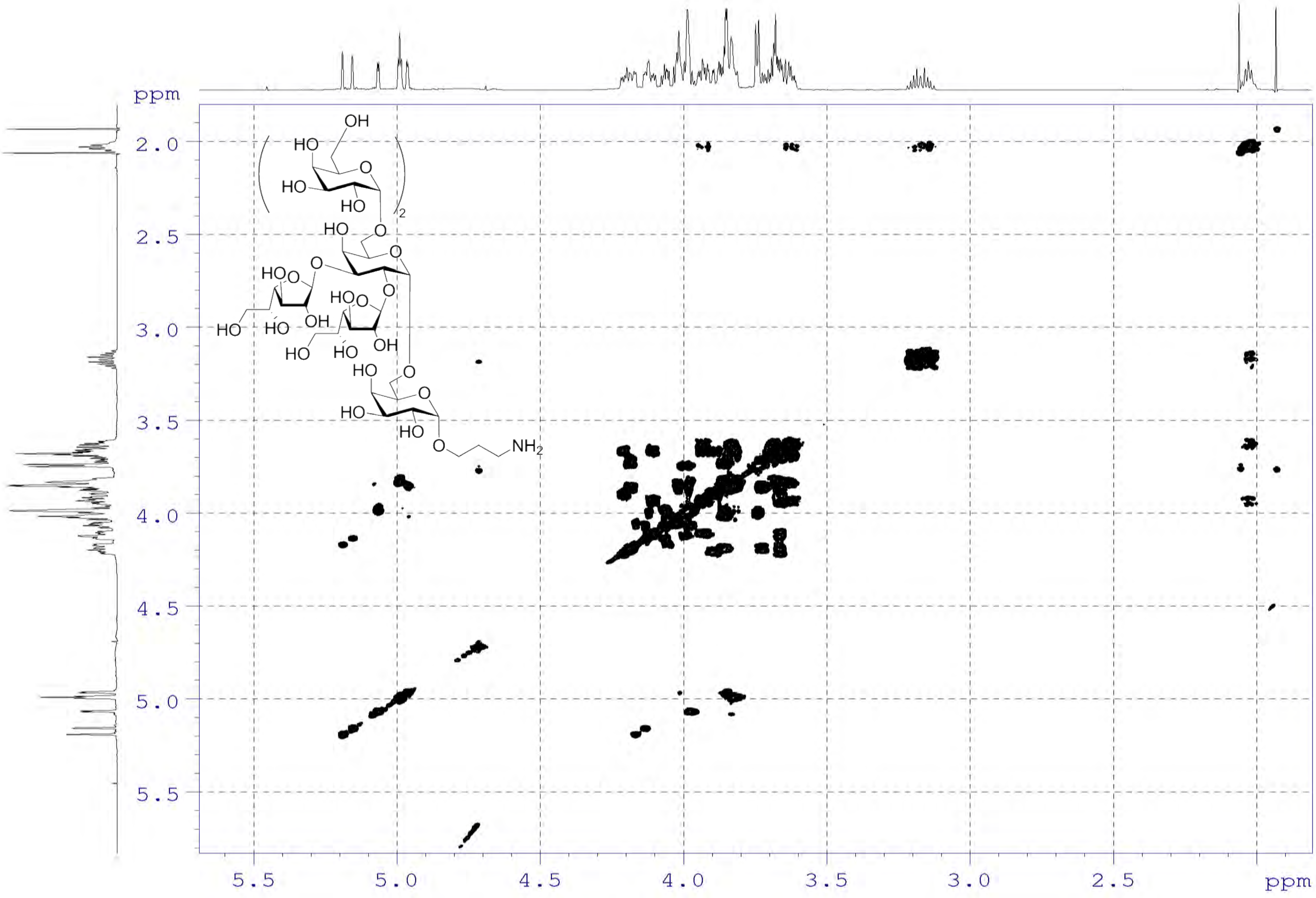

^{1}H-^{13}C HSQC of **5a** (600 MHz, D_2O, 303K)

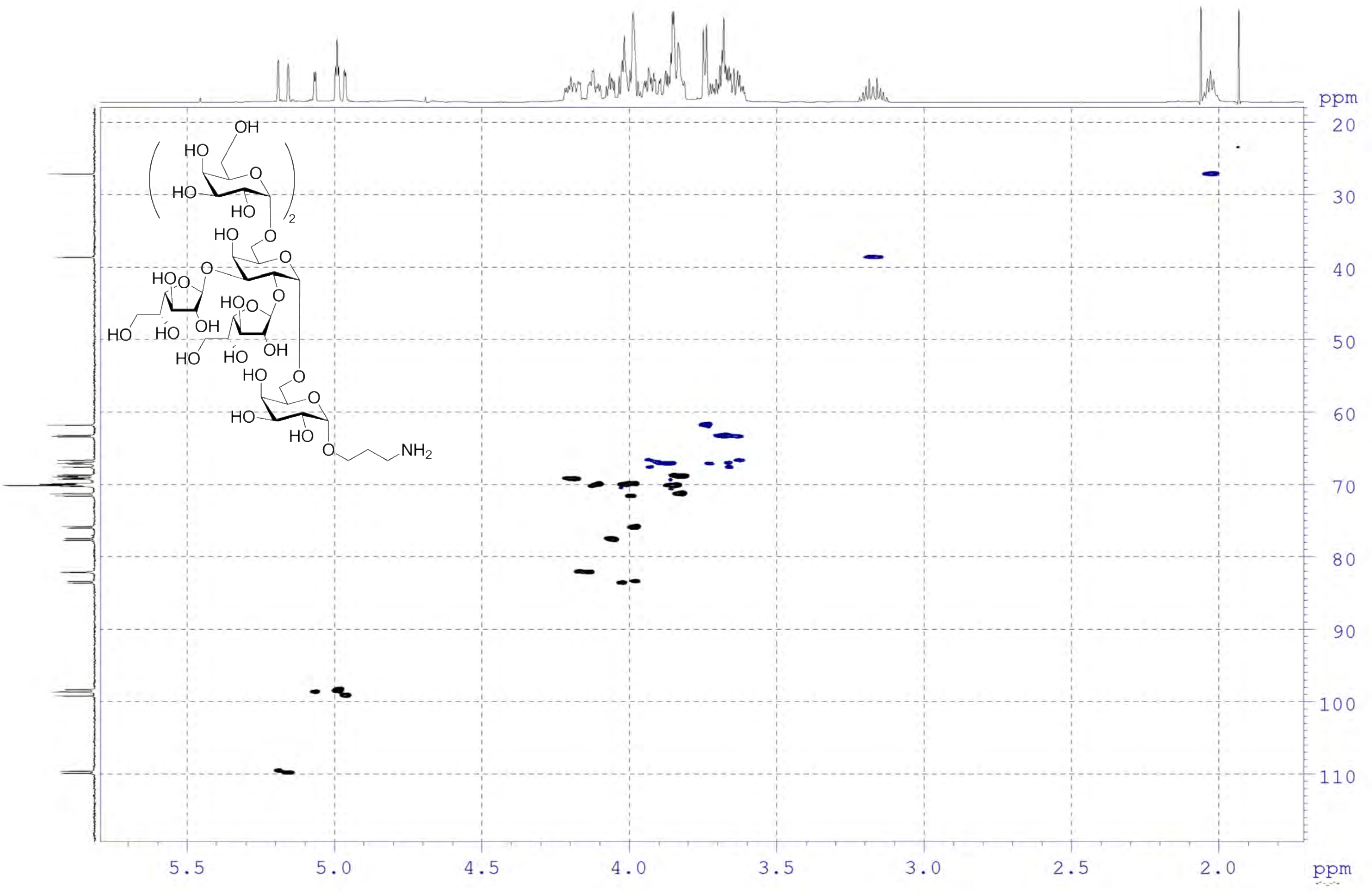

HRMS for **5a**

Comment CH3CN : H2O 50/50 %, dil. 200, calibrant addded

Acquisition Parameter

Source Type	ESI	Ion Polarity	Positive	Set Nebulizer	0.5 Bar
Focus	Active			Set Dry Heater	180 °C
Scan Begin	50 m/z	Set Capillary	4500 V	Set Dry Gas	4.0 l/min
Scan End	3000 m/z	Set End Plate Offset	-500 V	Set Divert Valve	Waste

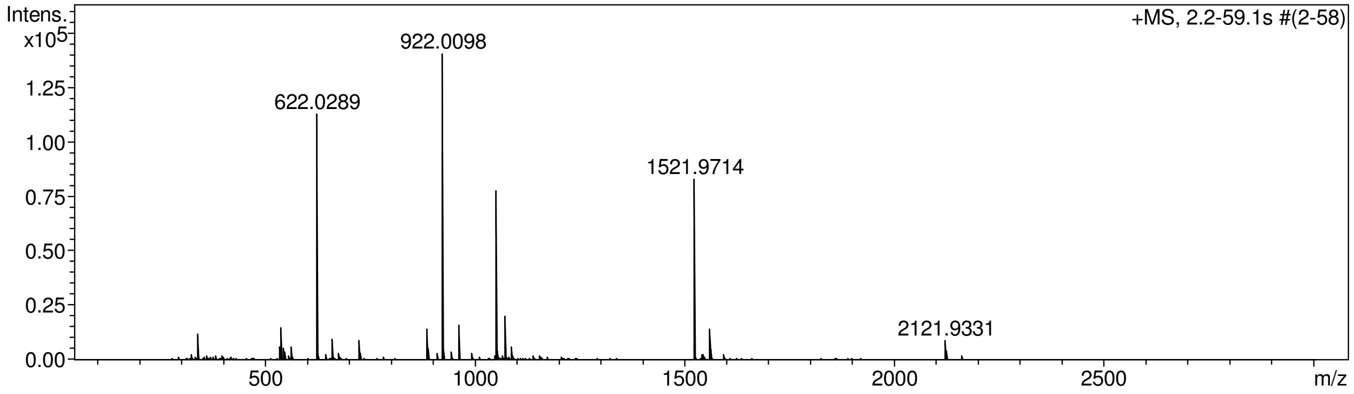

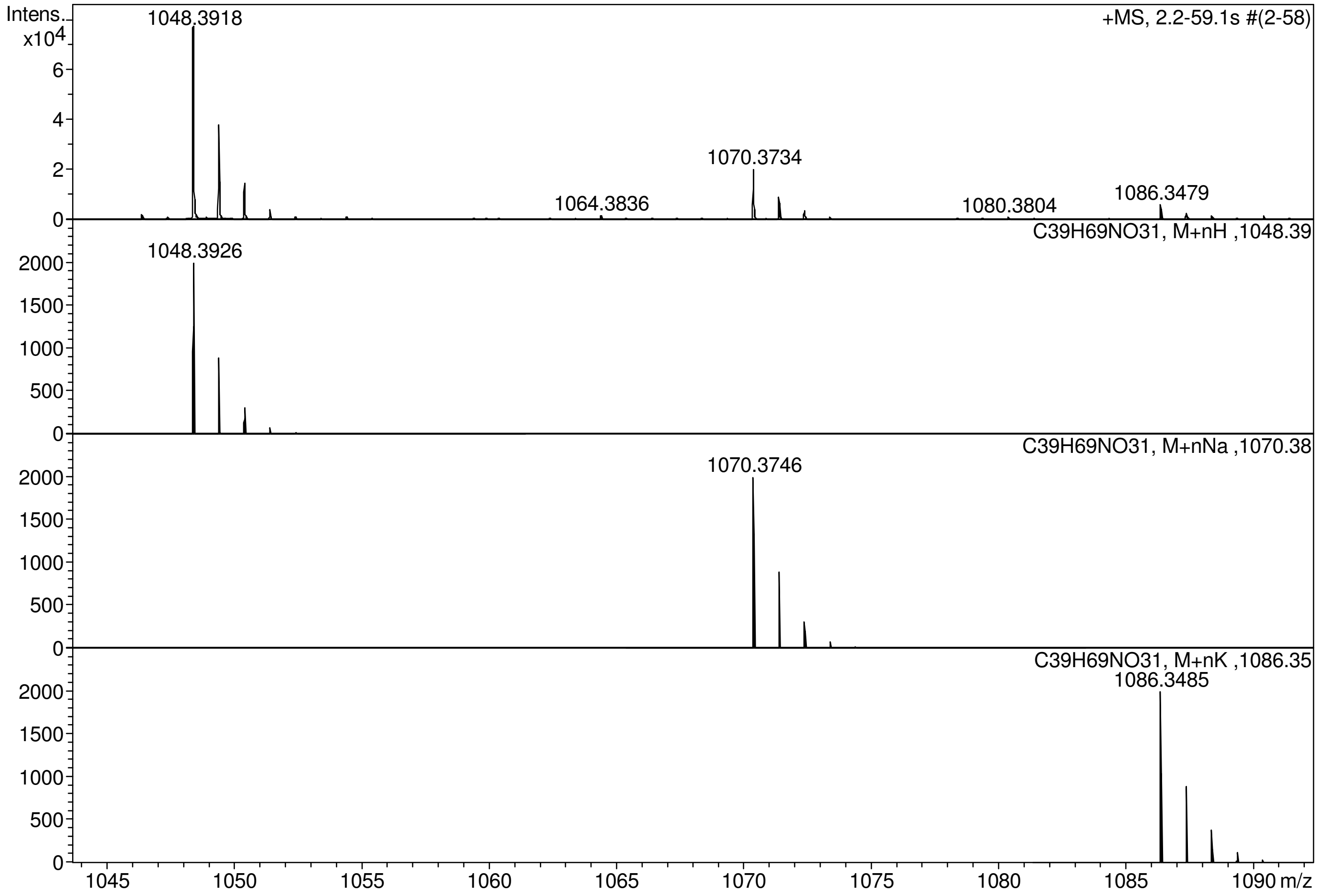

^{1}H-NMR of **5b** (600 MHz, D_2O, 303K)

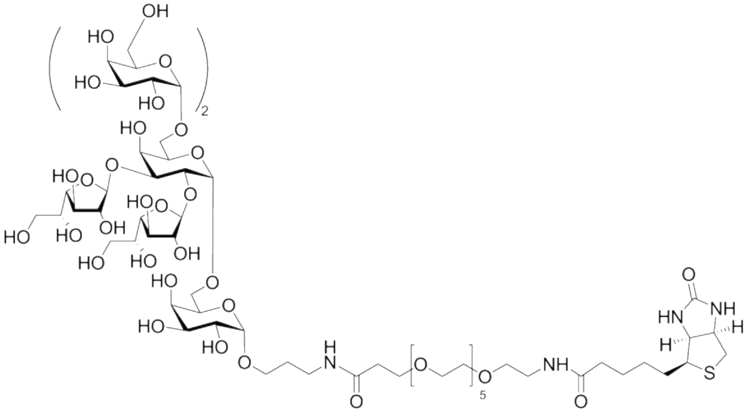

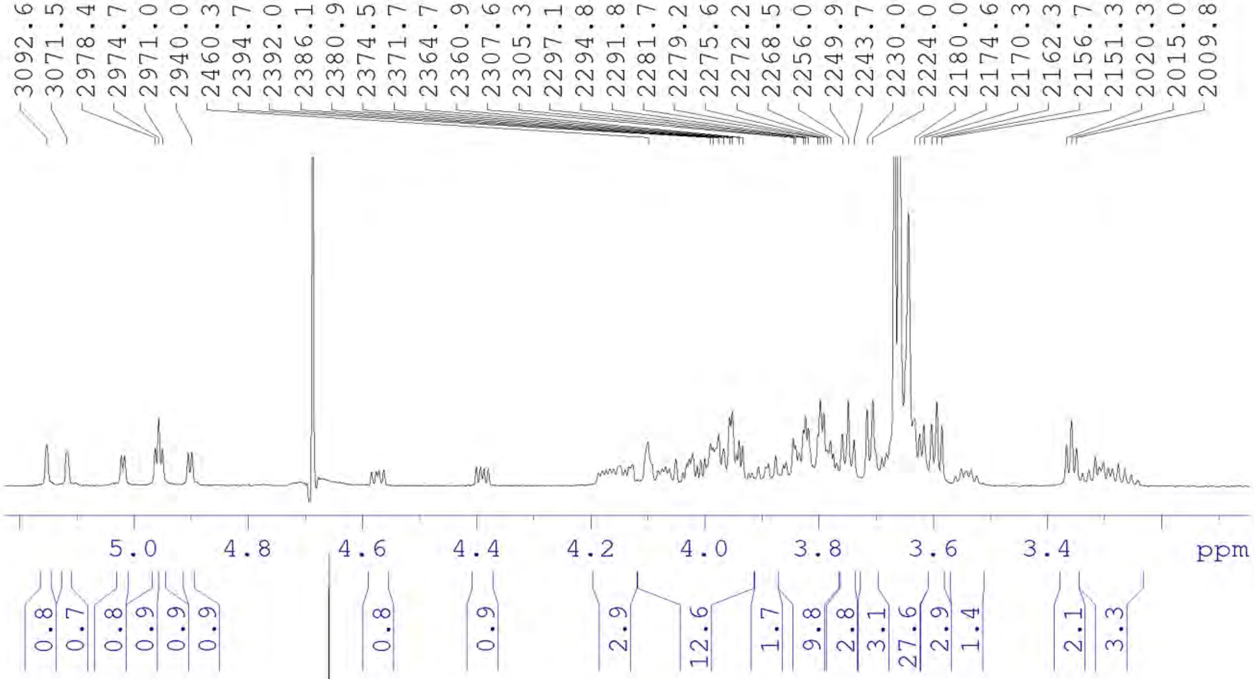

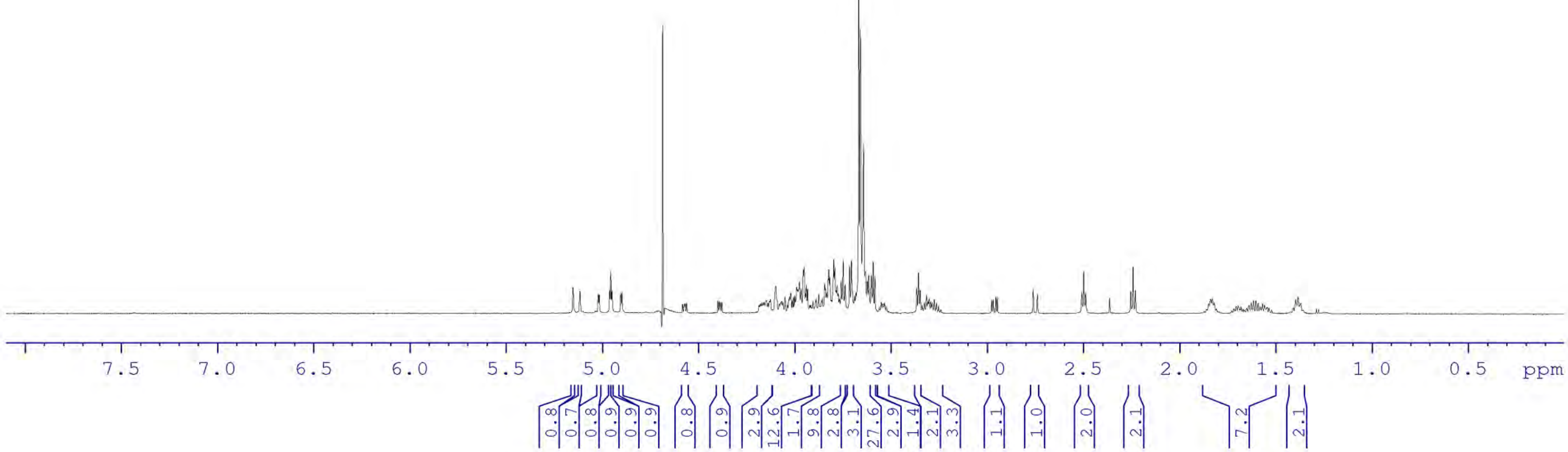

^{13}C-NMR of **5b** (150 MHz, D_2O, 303K)

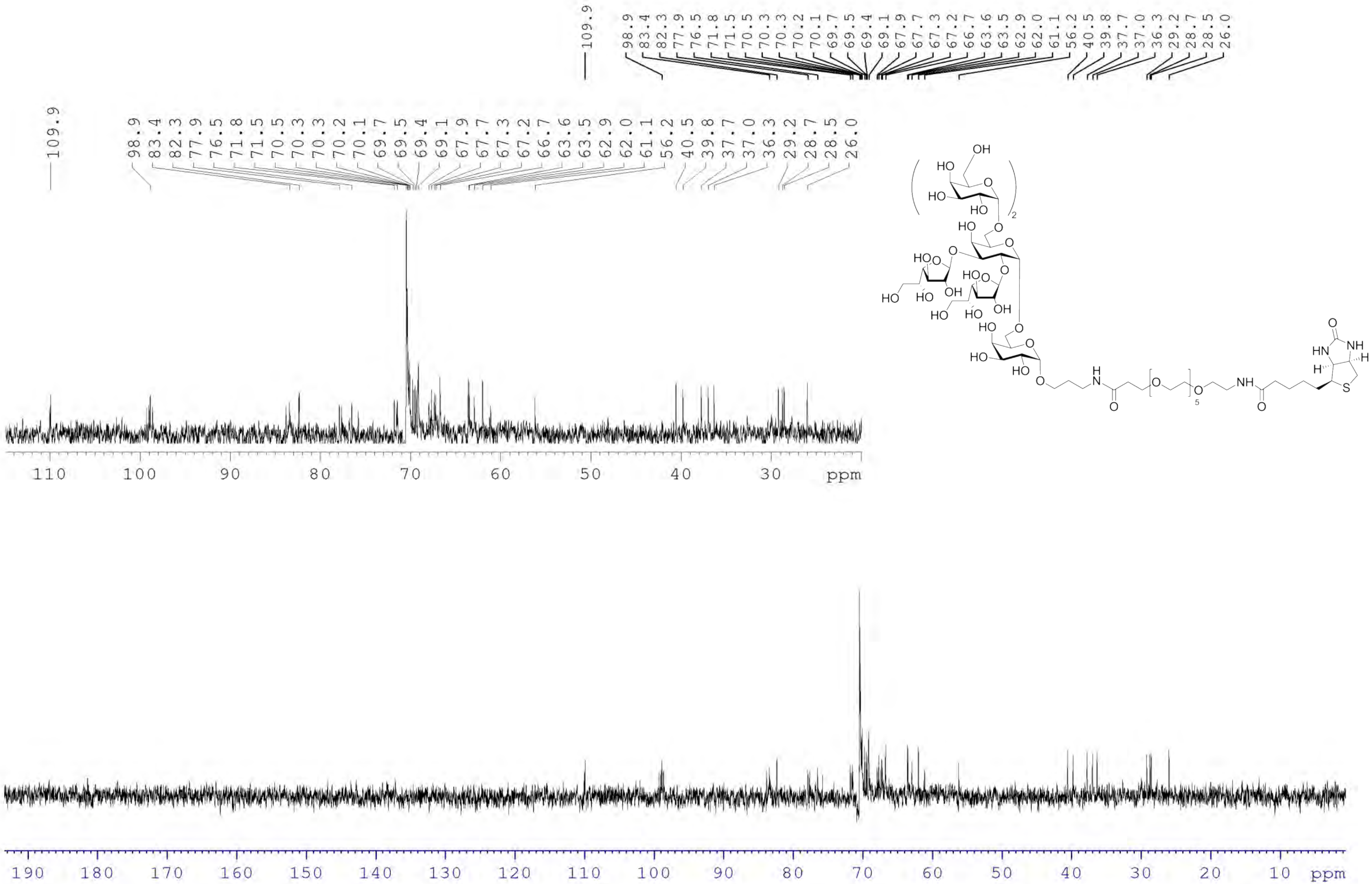

HRMS for **5b**

Comment	CH3CN : H2O 50/50 %, dil. 200, calibrant addded

Acquisition Parameter

Source Type	ESI	Ion Polarity	Positive	Set Nebulizer	0.5 Bar
Focus	Active			Set Dry Heater	180 °C
Scan Begin	50 m/z	Set Capillary	4500 V	Set Dry Gas	4.0 l/min
Scan End	3000 m/z	Set End Plate Offset	-500 V	Set Divert Valve	Waste

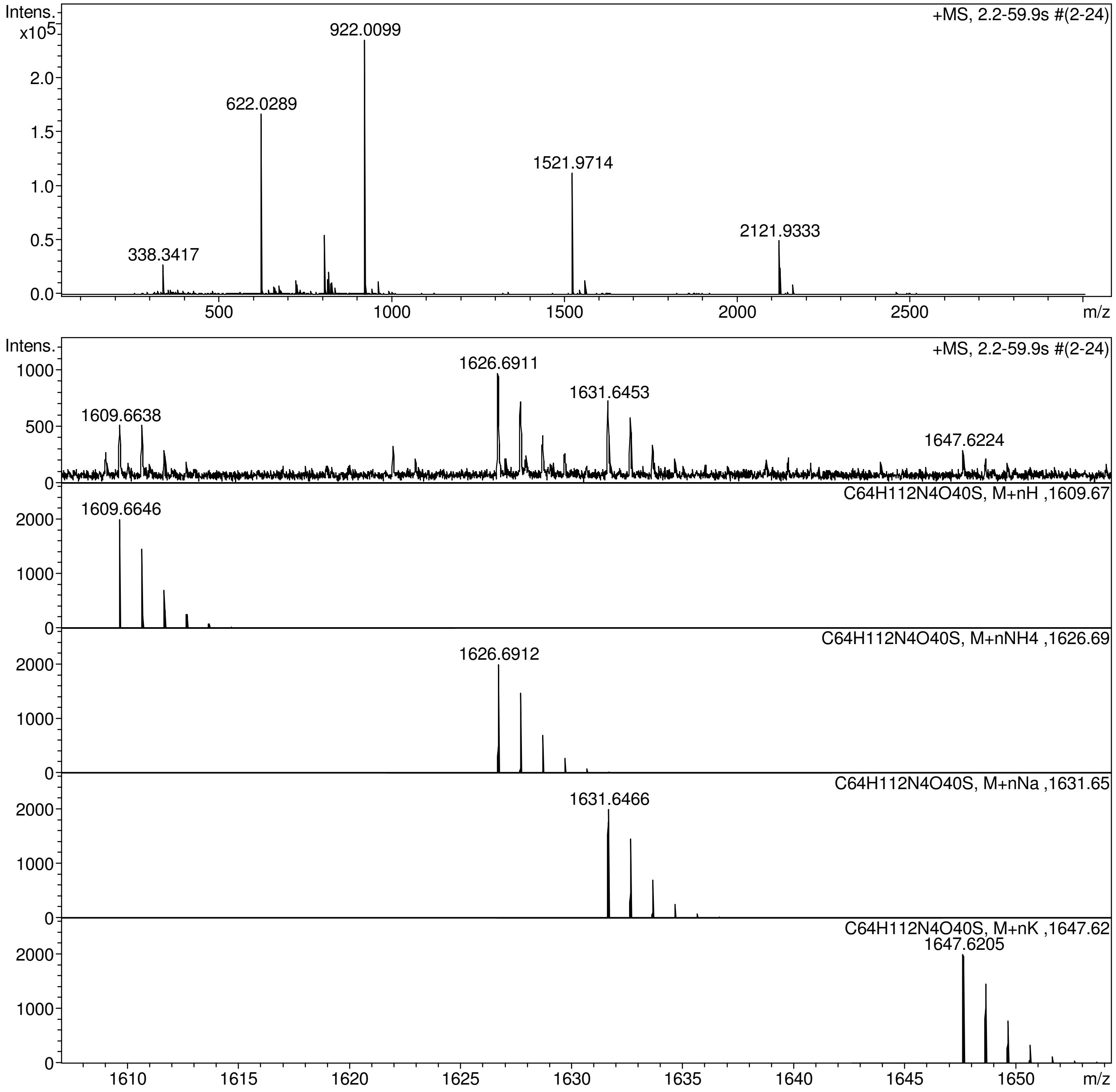

Supplement: Supplementary file 1 [file DataSheet1.PDF]
